# The central exons of the human MUC2 and MUC6 mucins are highly repetitive and variable in sequence between individuals

**DOI:** 10.1038/s41598-018-35499-w

**Published:** 2018-11-30

**Authors:** Frida Svensson, Tiange Lang, Malin E. V. Johansson, Gunnar C. Hansson

**Affiliations:** 1 Department of Medical Biochemistry, University of Gothenburg, Gothenburg, Sweden; 2 Big Data Decision Institute, Jinan University, Tianhe, Guangzhou, P. R. China

## Abstract

The DNA sequence of the two human mucin genes *MUC2* and *MUC6* have not been completely resolved due to the repetitive nature of their central exon coding for Proline, Threonine and Serine rich sequences. The exact nucleotide sequence of these exons has remained unknown for a long time due to limitations in traditional sequencing techniques. These are still very poorly covered in new whole genome sequencing projects with the corresponding protein sequences partly missing. We used a BAC clone containing both these genes and third generation sequencing technology, SMRT sequencing, to obtain the full-length contiguous *MUC2* and *MUC6* tandem repeat sequences. The new sequences span the entire repeat regions with good coverage revealing their length, variation in repeat sequences and their internal organization. The sequences obtained were used to compare with available sequences from whole genome sequencing projects indicating variation in number of repeats and their internal organization between individuals. The lack of these sequences has limited the association of genetic alterations with disease. The full sequences of these mucins will now allow such studies, which could be of importance for inflammatory bowel diseases for MUC2 and gastric ulcer diseases for MUC6 where deficient mucus protection is assumed to play an important role.

## Introduction

The epithelial surfaces throughout the gastrointestinal tract are covered by mucus. Mucus has an important role in maintaining the homeostasis between the gut microbiota and the host as well as to act as a protective barrier against pathogens, dehydration and physiological or chemical injury. The main components of the mucus are the mucins. These are heavily glycosylated proteins with domains that are rich in Proline, Threonine and Serine, called PTS domains or sequences^1^. These are often, but not always, composed of tandem repeats (TR) that can vary in number and was thus previously called VNTR for variable number of TR^2^. The sequences of the PTS domains are highly variable also between closely related species. The reason for this could be that the actual amino acid sequence is less important as long as it contains a sufficient number of anchor sites for *O*-glycans interrupted by prolines. With the glycans attached these form mucin domains that are extended rods, similar to a bottlebrush.

The *MUC2* and *MUC6* genes are located together with the *MUC5AC* and *MUC5B* near the recombination-rich telemetric end of a 400 kb gene cluster on the human chromosome 11p15.5^3,4^. These four all have central PTS domains and are gel-forming mucins that protect the epithelial cells of the human mucosal surfaces. The MUC2 protein is expressed in the small and large intestine^5^, MUC6 is mainly expressed in the glands of the stomach^6^, and MUC5B and MUC5AC in the respiratory tract^7^. MUC5AC is also the main surface mucin in the stomach.

The *MUC5AC* and *MUC5B* mucin genes have been fully sequenced and annotated in databases^8,9^. Variations in gene length between individuals has been noted for MUC5AC, MUC2, MUC6 but less so for MUC5B^4,8,10,11^. The MUC5AC and MUC5B PTS domains are frequently interrupted by small cysteine rich regions, called CysD, of which only two are found in the MUC2 PTS domains and none in the known MUC6 sequence. Changes in the organization and number of TR have been reported for respiratory diseases, specific allele-length polymorphisms in the PTS domain of MUC5AC was associated with severity of cystic fibrosis lung disease and asthma^8,12^. A specific SNP in the promotor of the *MUC5B* gene has recently been identified to be closely linked to lung fibrosis^13^.

The *MUC6* gene has only one TR region consisting of a 169 amino acid (aa) long repeat units^4^, with the estimated number of repeats varying between individuals from 15 to 26^10^. The reference sequence (GRCh38) for *MUC6* contains an imperfect TR region in the 3009 bp long sequence of exon 31. This sequence contains five TR units but this is not in agreement with the size range reported previously indicating an error in the assembly^10^.

The large central exon 30 of the human *MUC2* gene has two PTS domains where the larger one (PTS-TR2) is highly repetitive and has been extremely difficult to sequence^11,14^. The PTS-TR2 encodes 23 aa long repeat units and the number of repeats have been estimated to range from 50–115 with a mean size of approximately 100 TR^11^. The PTS region of the previous human genome reference sequence (GRCh37) was based on the original sequence published 1991 where a short part was sequenced and the deduced repeat unit was used to extend the sequence to the estimated size^11^. The new reference sequence (GRCh38) contains a PTS with more degenerated repeat sequence of 105 repeat units which would represent one of the longer alleles. The current Uniprot sequence (Q02817) still contains the sequence composed of 100 copies of the originally identified 69 nucleotide repeat sequence. The degenerated repeat sequence shows the importance of sequencing more variants to get a better reference library and to deduce the protein sequence.

The allele variation in the mucin genes has been indicated to have an ethnic link^11^, but no definite association between specific alleles from different mucin genes has been observed^10^. The number of TR in the *MUC2* and *MUC6* mucins was estimated from bands on Southern blots. Thus specific sequence details of the variability in these polymorphic alleles were not elucidated. The reason for this is technical as conventional sequencing methods have not been able to read-through these long TR. New sequencing strategies, such as Single Molecule Real-Time (SMRT) sequencing^15^ can generate the required long sequencing reads, enabling sequencing across the entire PTS repeat. Concentrated high quality DNA is still required, however through the use of Bacterial Artificial Chromosome (BAC) clones containing the full PTS sequence this is achievable. To have a number of different TR sequences will be important as references for human whole genome sequencing (WGS) projects where this region has and will likely continue to have low coverage^16–19^. As the MUC2 mucin is important in intestinal protection and the MUC6 mucin protects the gastric glands genetic variation could play a role in related diseases such as inflammatory diseases and gastric ulcers.

Here we report sequencing of a BAC clone spanning the *MUC6* and *MUC2* genomic region by SMRT sequencing technology. The repeat region of *MUC6* reveal a longer sequence compared to the reference sequence of 24 TR units matching the estimated length from Southern blot experiments and MUC2 reveals a different variant compared to the reference sequence with 98 TR matching the most common allelic lengths identified. We also compared the MUC2 and MUC6 TR units from all available MUC2 and MUC6 TR sequences from WGS projects using our sequence as template and found both sequence variation and length polymorphism.

## Results

### Spontaneous recombination occurs within *MUC2* PTS-TR2 region in bacteria

**Figure 1:**
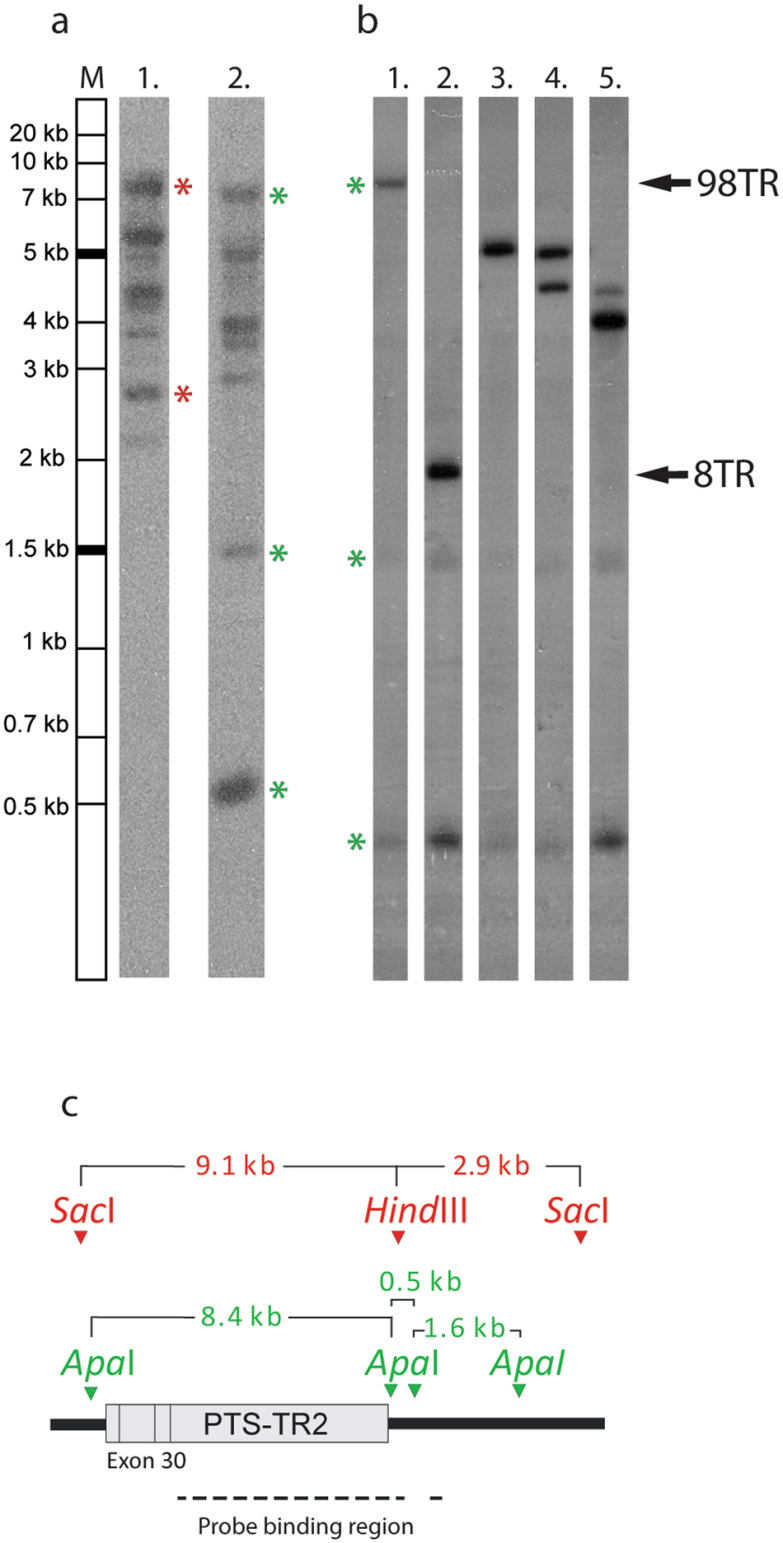
Southern blot analysis of MUC2 PTS-TR2 integrity shows variable number of tandem repeats. Southern blot using SMUC41 probe on small-scale purified RP13-870H17 BAC DNA (5–10 μg/lane). Expected bands are indicated by * and sizes are given in kilo bases (kb) (**a**) *Sac*I*/Hind*III-digested, *Lane 1*, and *Apa*I-digested, *Lane 2*, original RP13-870H17 BAC clone showed more bands than expected. (**b**) *Apa*I digest of different clones, *Lanes 1*–*5*, of the BAC showed PTS-TR2 variants from 98TR, *Lane 1*, down to 8TR, *Lane 2*. (**c**) Schematic picture of the DNA detected in the Southern blot with the multiple binding sites for the probe indicated and the sites for the used restriction enzymes. The original autoradiograms are shown in the Supplement as Figure X1.

The *MUC2* exon 30 is known to contain the PTS-TR domains of the central part of MUC2^11^. The integrity of this region was analyzed by Southern blot using a probe specific against the *MUC2* PTS-TR2 repeat region. When the original BAC bacterial stock (from scraping) was grown, more bands than expected were observed by Southern blot, using both *Apa*I and *Sac*I*/Hind*III digested DNA (Fig. 1a,c). These bands were smaller than expected and suggested recombination with loss of repeats. When single colonies from this stock were cultured and *Apa*I digested, single or double bands were observed on the Southern blot (Fig. 1b). The BAC showed at least six different *MUC2* PTS-TR2 lengths with sizes ranging from approximately 8 kb down to 2.5 kb (some of these variants are shown in Fig. 1b). The number of smaller bands increased over time when the BAC was propagated in bacteria and attempts to optimize the culturing conditions did not alter the recombination frequency. Sanger sequencing analysis of the smallest of these variants (Fig. 1b, Lane 2) revealed loss of TR within the PTS-TR2 where only 8 TR remained (Fig. S1). The recombination had occurred between repeat 1 and repeat 91 (Fig. S1). The first PTS domain and the two CysD domains also localized within exon 30 were not affected by the recombination. It can be concluded that the PTS-TR2 region of *MUC2* exon 30 is unstable and recombines spontaneously when propagated in the DH10B bacterial strain.

### PacBio SMRT Sequencing of BAC clone

**Figure 2:**
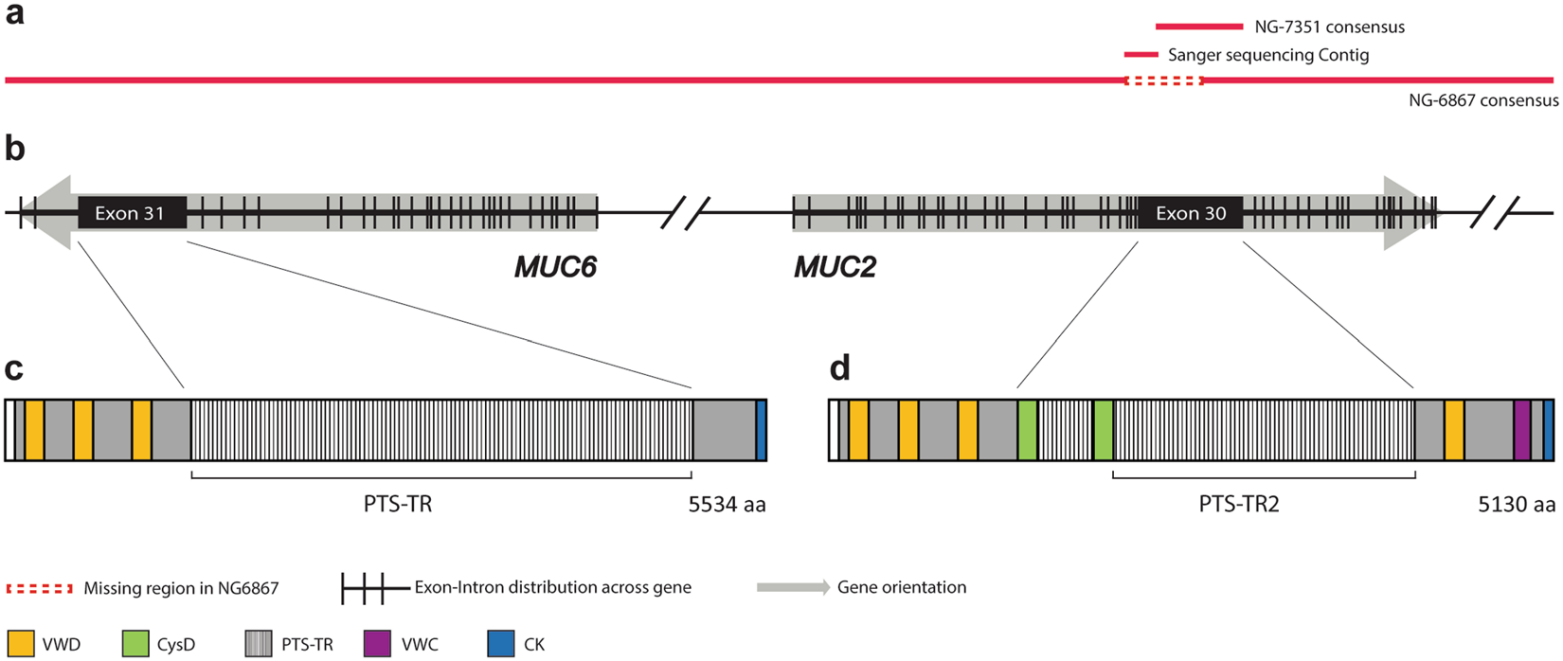
Schematic presentation of the assembled sequence for RP13-870H17 produced from PacBio SMRT and Sanger sequencing. (**a**) The RP13-870H17 assembly from SMRT sequencing results of whole BAC clone (NG-6867) and *Hinf*I MUC2 PTS-TR2 fragment sequence (NG-7351) assembled with Sanger sequencing. (**b**) Schematic picture of *MUC6* and *MUC2* gene organization showing the gene orientation and exon and intron distribution. (**c,d**) Resulting protein domain organization for MUC6 and MUC2. VWD = von Willebrand like domain type D, VWC = von Willebrand like domain type C, CK = *C*-terminal cystine knot.

The BAC clone RP13-870H17 containing full length *MUC2* PTS-TR2 as shown by Southern blot (Fig. 1) was sequenced by Pac Bio SMRT sequencing. Statistical summary can be found in Table S1. The results revealed a consensus sequence of 149,943 bp (including vector, Fig. S2) with 99.85% consensus concordance and mean depth of 1,168x. This sequence lacked *MUC2* exon 27 through to part of exon 30 (NG-6867 dotted red line, Fig. 2a). Mapping of raw reads against the missing sequence showed considerable coverage by reads, but only 14 reads covered the complete length of PTS-TR2 and only 3 reads were found to cover the complete exon 30.

*Hinf*I restriction endonuclease digestion is known to cut just outside PTS-TR2 of *MUC2*^11^ and has been used to analyze allelic length variation. To complete the sequence, the 7 kb *Hinf*I PTS-TR2 fragment was excised from the cleaved BAC DNA and submitted for SMRT sequencing. The sequencing result (Fig. S3) gave a consensus sequence of 7,034 bp (NG-7351) with 99.90% consensus concordance and mean depth of 483×, covering the entire *MUC2* PTS-TR2. This size agrees with the expected size of the PTS-TR2 as observed by Southern blotting (Fig. 1). Comparing the results of the two SMRT assemblies showed that the last 56 repeats of NG-7351 where identical to the last repeats in NG-6867. To complete the full-length BAC DNA sequence the remaining gap was analyzed by Sanger sequencing and was assembled into a 147,780 sequence (156,584 bp including vector of 8,804 bp) (Fig. S4). The BAC clone also contained the MUC6 gene and this was well covered by the SMRT sequencing.

### The *MUC2* and *MUC6* genes in BAC assembly

The *MUC2* gene contained 49 exons including the large central exon 30 (8,793 bp), revealing 98 TR composed of the 69 nucleotide long repeat units, with some degeneration of +/− 3 bp in two of the first four TR units (Fig. S5).

The previously annotated DNA sequence for BAC clone RP13-870H17 (GenBank: AC139749.4) is in addition to the unresolved region corresponding to exon 30 of *MUC2* also lacking most of the *MUC6* exon 31. Using the obtained sequences, we were able to resolve the large exon 31 containing a 12,294 bp sequence including 24 TR units, the majority of which corresponds to the previously known 507 bp repeats units (Fig. S6)^6^. The *MUC6* gene contained 33 exons and was considerably larger than previously understood and has a length similar to the other gel-forming mucins. Our sequencing assembly results thus confirms the organization of the two genes^4^ showing that both the *MUC2* and *MUC6* PTS-TR regions were different in length and sequence from previous and current reference sequences (Figs 2, S5 and S6).

### The amino acid composition of the PTS domains of the MUC2 and MUC6 mucins

**Figure 3:**
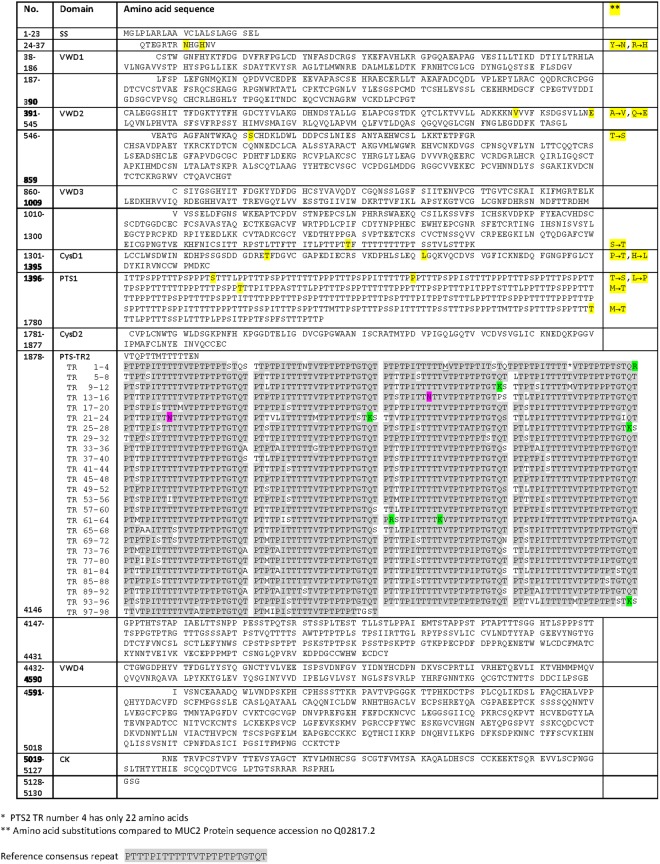
Amino acid sequence of MUC2. Translation of the *MUC2* mRNA from the RP13-870H17 assembly. Amino acid differences compared to the reference sequence are shown in yellow. Specific rare aa as K and R is marked as well as potential N-glycosylation sites.

**Figure 4:**
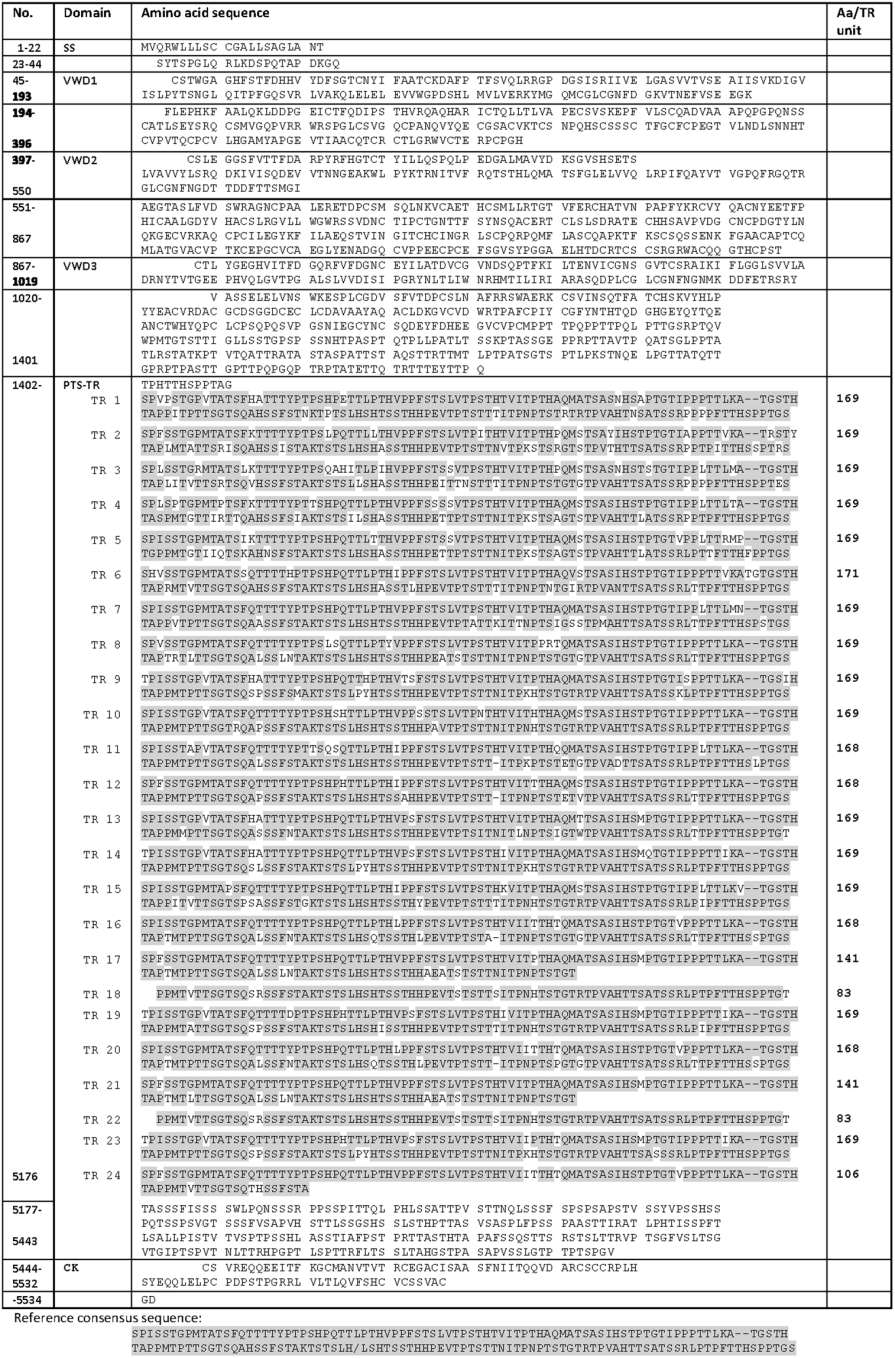
Amino acid sequence of MUC6. Translation of the *MUC6* mRNA from the RP13-870H17 sequence assembly.

The precise amino acid sequence of one MUC2 variant was only recently deduced and the variability of the PTS of MUC2 was previously undefined. The full amino acid sequence of MUC6 had not been previously elucidated. The mRNA sequence for *MUC2* (Fig. S7) and *MUC6* (Fig. S8) were translated into 5,130 aa for MUC2 and 5,534 aa for MUC6 (Figs 3 and 4).

Our results show that the translated MUC2 PTS-TR2 domain (2,254 aa) is composed of 98 units of 23 aa repeats (+/− 1 aa) with at least 78% sequence homology to the consensus repeat (Fig. 3). The aa composition within every tandem repeat unit varied and the previous theoretical assumption of identical repeats has been proven wrong. Out of the total 98 repeats, 32 were unique (Figs 3 and S9). The most abundant amino acids were Thr (55.5%) and Pro (21.7%) (Table S2). It should be noted that the PTS-TR2 contain both Arg and Lys that are potential cleavage sites for digestive enzymes and that two N-glycosylation consensus sequences were present. Alignment of MUC2 with UniProtKB Q02817.2 shows that the two *N*-terminal sequences vary by single amino acid polymorphism (Fig. 3).

For MUC6 no differences in amino acid sequence flanking the PTS region were observed when aligned to the UniProtKB Q6W4X9 sequence. Translation gave a 3,763 aa long repetitive PTS-TR sequence composed of 24 TR composed of 21 non-identical units, primarily of 169 aa^6^, two shorter identical repeats of 83 aa, two identical 141 aa repeats and one shorter final repeat unit (Fig. 4). This is 3,095 aa longer than the UniProtKB Q6W4X9 sequence for MUC6 that consists of only 668 aa in its PTS-TR domain. The sequence homology between repeats was at least 82% to the consensus repeat (Fig. S10) with the most abundant aa being Thr (30.1%), Ser (17.8% and Pro (13.7%) (Table S2).

### MUC2 and MUC6 tandem repeat sequence diversity and length polymorphism

Until now, only polymorphic length variation of the mucin genes has been studied^10,20,21^ and polymorphic sequence details have been lacking. Recently, PacBio SMRT sequencing of a hydatidiform (haploid) mole BAC library has been annotated in the NCBI sequence database^16^. A BAC clone, CH17-246P12 (GenBank: AC256300.1), derived from the CHORI17 PacBio BAC library, was shown to contain a part of the mucin cluster on Chr 11p15. This sequence is also used as the RefSeq for MUC2 (GRCh38), but not for MUC6. The clone contained the full *MUC6* and *MUC2* genomic sequences and their PTS-TR units were extracted from this BAC clone. *MUC2* was shown to contain 105 TR (Fig. S11) and *MUC6* 20 TR. The 98 TR of RP13-870H17 were aligned to the 105 TR of CH17-246P12 with respect to shared unique repeats (Fig. S11). The two sequences shared 61 *MUC2* repeats and 14 *MUC6* repeats. Comparison of our 98 PTS-TR2 repeats from RP13-870H17 with the 105 MUC2 PTS-TR2 repeats of the CH17-246P12 showed nucleotide polymorphisms leading to aa differences. The overall aa composition was very similar, but two histidine residues were present only in the CH17-246P12 repeats (Table S2). The overall aa composition of MUC6 was very similar between the two clones (Table S2).

**Figure 5:**
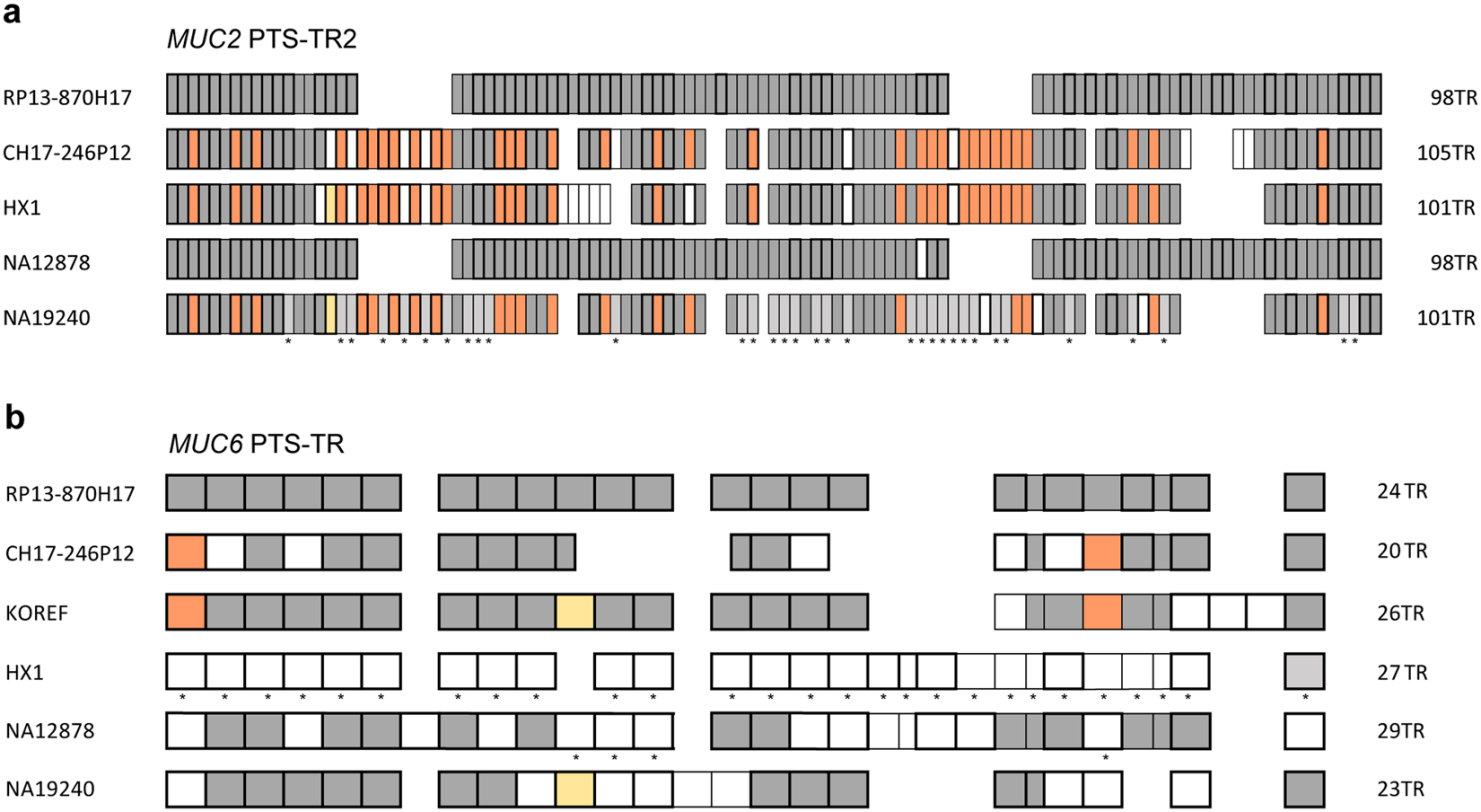
Schematic alignment of amino acid TR units derived from *MUC2* and *MUC6* genomic sequences. Aligned DNA sequences from MUC2 PTS-TR2 (**a**) and MUC6 PTS-TR (**b**) from different sequencing projects. Each box represents 1 TR unit (as outlined in Figs 3 and 4). Highlighted boxes indicate unique repeats. TR units homologous to RP13-870H17 are marked in grey and to CH17-246P12 are marked in orange. White boxes indicate non-identical TR units. Yellow boxes show identical repeats. *Indicate incomplete sequence (inserted/deleted bases). The KOREF *MUC2* sequence was incomplete and misassembled and was therefore not used.

Human WGS projects using the PacBio SMRT sequencing platform have increased during the past years. To further address individual differences of this polymorphic region, the *MUC2* and *MUC6* PTS-TR regions were compared to sequences from four additional WGS projects, KOREF^17^, HX1^18^, NA19240, and NA12878^19^. These were selected as they contain PTS sequences, which were assembled for the analysis, but contained features recognized as errors from the methods used indicating low coverage of this region. The general repeat patterns were compared showing variation in the numbers of repeats and their actual sequences in both the *MUC2* and *MUC6* genes (Fig. 5). Overall, the alignment of both *MUC2* and *MUC6* tandem repeat units indicate that loss or duplications of tandem repeats are the main reasons for the observed differences.

## Discussion

Long and highly repetitive GC-rich regions have been difficult to sequence by commonly used DNA sequencing methods. However, Single Molecule Real-Time (SMRT) sequencing methods has changed this. One group of proteins, the mucins, typically has long central coding sequences that are difficult to sequence due to their repetitive nature and high GC content. The first example using SMRT sequencing to elucidate PTS sequences of mucins was for MUC5AC^22^. The PTS sequences of MUC5AC, regularly interrupted by CysD domains making the PTS sequences shorter than for *MUC2* and *MUC6* genes, was successfully sequenced. In this study a BAC clone containing both the *MUC2* and *MUC6* gene was successfully sequenced using Pac Bio SMRT sequencing. The highly repetitive *MUC2*PTS-TR2 sequence was very unstable when the BAC was propagated in bacteria with an impaired recombination system, illustrating another serious problem for the analysis of the sequence of these molecules. Recloning and growth of the bacteria had to be continuously monitored to verify PTS-TR2 sequence integrity by Southern blot analysis. Our first attempt to fully sequence the complete BAC clone was unsuccessful as only few reads covered *MUC2* exon 30. This exon is rich in GC and it could be speculated that the missing sequence was lost due to technical problems such as secondary structure of the DNA. By isolating the *MUC2* PTS-TR2 region after cleavage of the BAC, we were able to obtain the full nucleotide sequence. Long-read sequencing from Human WGS projects also show low coverage for the *MUC2* PTS region, especially for PTS-TR2, suggesting that analysis of these sequences still remains highly challenging despite new technologies.

One of the sequences from the newly sequenced genomes discussed is from a haploid genome (CH17). Sequencing both alleles at once pose special problems as the coverage of the PTS regions is low and the high error rate of the method require high number of reads to obtain a reliable consensus. The degree of recombination during propagation of this BAC is not described and could also contribute to lack of sequences. The low sequence coverage of the TR regions in combination with large allelic variation will make the assembly of diploid sequencing challenging. It is thus important that future work include haploid resolution when studying these regions, allowing correct sequences of TR regions to determine contributing effects to disease. Most likely, higher sequence coverage requires the isolation and specific sequencing of these regions as outlined in this study.

We found that our BAC clone contained 98 PTS-TR2 units in the *MUC2* gene, something that corresponds with the most common length estimated by Southern blot analyses and to the first sequence prediction containing 100 copies of the general repeat sequence^11,14^. The *MUC6* gene in our BAC encodes 24 TR units which gives a longer sequence than what was provided by previous sequencing as well as in the reference assembly (GRCh38), but the length fits with what was estimated as a common allele by Southern blot^10^. This longer sequence also makes MUC6 similar in length to the other gel-forming mucins. All this argues that the assemblies provided here are of full length PTS regions and likely represents normal variants.

Our sequence also reveals that the repeats are not well conserved. The aa sequence of MUC2 PTS-TR2 contained several aa variations between repeat units and when compared to the extracted MUC2 PTS-TR2 from CH17-246P12 a similar variability could be observed. The repeats seemed to be fairly conserved especially at the ends of the TR region indicating more genetic instability in the central part of the repeat regions. Histidines were only found in the 105 TR from the CH17 BAC clone as well as in HX1 and NA19240, but not in the 98 TR from the RP13 BAC clone. This could be normal variation as seen in many of the other differences but methodological errors such as the systematic errors often seen with SMRT sequencing could result in variation and thus very good coverage of the sequences is crucial. In contrast to *MUC2*, the *MUC6* TR showed high identity between the compared sequences, and there was less diversity in sequence compared to *MUC2*. The main variability in the *MUC6* gene is in the length of these tandem repeat regions. This could indicate that the degree of variation differs between the different mucin genes.

With a better sequencing strategy, we were able to generate full and reliable sequences of genomic region containing the MUC2 and MUC6 gene which has proven to be extremely difficult to resolve in the past. The revealed sequence variation within the PTS regions and their known length variation indicate the need for several good sequences in a reference library to map data with less good coverage produced in other studies including WGS projects. The possibility to decipher these sequences in larger populations will likely be important for genetic associations with mucus related diseases such as ulcerative colitis^23^. Improved SMRT sequencing methods enabling region specific sequencing and multiplexing could also help in generating information from larger cohorts, but this would likely need optimization and is something for future studies.

## Materials

### BAC clone and bacterial strains

A human BAC clone, RP13-870H17 (GenBank: AC139749.4), known to carry a segment of chromosome 11p15.5 including *MUC2* and *MUC6* genomic sequences, was purchased from BACPAC Resources. It was cultured in the *E. coli* DH10B strain in LB medium at 37 °C less than 15 h.

### Southern blot analysis

Southern blot analysis was performed using SMUC41 probe for *MUC2*^14^. Purified BAC DNA was digested with *Apa*I restriction enzyme (Thermo Fisher Scientific) or double digested with restriction endonucleases *Sac*I*/Hind*III (Thermo Fisher Scientific) over night at 37 °C according to supplier instructions. Digested DNA was separated on a 1% 0,5x TBE agarose for 20 h at 2 V/cm (50 V). The gel was soaked in denaturating buffer [1,5 M NaCl/0,5 M NaOH] 2 × 30 minutes and then in neutralizing solution [3 M NaCl/0,5 M Tris-HCl pH 8.0] for 60 minutes. DNA was transferred by capillary transfer to a Zeta-Probe GT membrane (Bio-Rad) in 10x SSC [1.5 M NaCl/150 mM trisodium citrate] for 24 h at room temperature. The membrane was air dried and baked at 80 °C for 2 h. The membrane was pre wet in 2× SSC buffer and pre-hybridized in Church buffer [0.5 M KPO_4_ pH 7.4/1% BSA/1 mM EDTA/7% SDS]. The probe, 25 ng, was labelled with ^32^P αdCTP (Perkin Elmer) using RediPrimeII (GE Healthcare) according to manufacturer’s protocol. The probe was added and hybridized overnight at 65 °C. The membrane was washed with wash buffer 1 [40 mM KPO_4_ pH7.4/1 mM EDTA/5% SDS/0.5% BSA] 2 × 15 min. at 65 °C and wash buffer 2 [40 mM KPO_4_ pH 7.4/1 mM EDTA/1% SDS] 4 × 10 min. at 65 °C and was then exposed to a BioMax MS film (Kodak) 2–20 h. The relative sizes of the fragments were determined by comparing measured distances between fragment and wells with GelRed (Biotinum) post-staining of a GeneRuler 1 kb Plus DNA ladder (Thermo Fisher Scientific) included on the gel.

### BAC DNA purification methods

For sequence analysis, large-scale purifications of total BAC DNA was purified from bacterial cultures using the Large Construct Kit (Qiagen) according to manufacturer’s appendix A protocol. Isolation of BAC DNA from small-scale cultures, for Southern blot analysis, was performed using 5–10 ml overnight cultures that were centrifuged 2000 × g for 5 minutes. The pellet was dissolved in 250 µl cold Buffer 1 [50 mM Tris-HCl/10 mM EDTA/10 µg/ml RNase A] and transferred to a 1,5 ml tube. After adding 250 ul of Buffer 2 [200 mM NaOH/1% SDS] the tube was inverted 6–8 times and 300 μl of Buffer 3 [3.0 M Potassium acetate pH 5.5] was added. Centrifugation was performed for 5 minutes at 16,000 × g at 4 °C, the supernatant with DNA was precipitated by adding 800 μl of 95% ethanol. Tube was gently mixed and centrifuged at 12.000 × g at room temperature for 5 min. The DNA pellet was washed in 500 μL of 70% ethanol, air dried and dissolved in 25 μl 5 mM Tris-HCl pH 8. The DNA was quality controlled and quantified by NanoDrop (Thermo Fisher Scientific) and on 0.7% TAE or TBE agarose gels as well as on Sothern blot.

### Purification of *Hinf*I digested *MUC2* PTS-TR2 fragment

*Hinf*I is known to cut on either side of the *MUC2* PTS-TR2 domain^11^. Total BAC DNA was digested with *Hinf*I (New England Biolabs) overnight at 37 °C according to supplier instructions. The 7 kb band of digested BAC DNA was gel purified from a 0.7% TAE agarose gel, without UV exposure or DNA stain, using the Nucleo Spin Gel Purification kit (Macherey-Nagel) according to supplier recommendations for low concentration DNA. Lanes with a DNA ladder, GeneRuler 1 kb Plus (Thermo scientific), and a reference of *Hinf*I digested BAC DNA was cut from the gel and post stained with GelRed™ nucleic acid stain (Biotium). The distance from well was measured and gel pieces were accordingly cut at 7 kb +/− 5 mm on the non-stained gel. The DNA was eluted with buffer NE [5 mM Tris-HCl, pH 8.5] at 70 °C. DNA precipitation was performed using 0.1 volumes 3 M Sodium Acetate and 2.5 volumes of 95% ethanol for 3 h on ice. The DNA pellet was washed in 70% ethanol, air dried and dissolved in EB buffer (10 mM Tris, pH 8.5). The DNA concentrations and purity were measured using NanoDrop and on a 0.7% TAE agarose gel.

### SMRT sequencing and Bioinformatics

BAC DNA (NG-6867) (10 µg) was fragmented with G-tube (Covaris) and a SMRT bell DNA template library with 8–12 kb insert size was prepared according to standard protocol (Pacific Biosciences) followed by size selection using AMPure PB magnetic beads. SMRT sequencing was performed on the PacBio *RS* II according to standard protocol using MagBead loading with P4-C2 chemistry and a 120 min movie.

SMRT bell DNA template library of the *Hinf*I MUC2 PTS-TR2 fragment (NG-7351) (3.2 µg) was performed without further fragmentation, through direct adaptor ligation using a standard protocol for 10 kb template preparation and sequencing with low-input DNA (Pacific Biosciences). SMRT sequencing was performed as described above.

Long-read *de novo* assembly was performed using algorithms and software’s with parameters recommended in the Hierarchal Genome-Assembly Process (HGAP)^24^ using the PacBio HGAP software preset assembly protocol HGAP 2.0 (NG6867) (SMRT-Portal 2.1.0, SMRT-Analysis version 2.1.0) and HGAP 3.0 (NG-7351)(SMART-Analysis version 2.2.0. Library construction’s, SMRT Sequencing and assembly were performed at Eurofins genomics, Germany. Library quality was analyzed by Qubit (Thermo Fisher Scientific), and average fragment size was estimated using a Fragment Analyzer (Advanced Analytical).

### PCR amplification and Sanger sequencing

The DNA sequence containing the *MUC2* PTS-TR1 region was amplified from the BAC clone (1,237 bp), with PTS-TR1 Forward (F) and reverse (R) primers (Table S3), using Pfu Turbo (Agilent technologies) according to supplier’s instructions, with additional dimethyl sulfoxide (DMSO) to a final concentration of 10% (vol/vol). PCR conditions consisted of an initial denaturation step of 94 °C for 2 min., followed by 30 cycles of a three –step amplification cycle of 94 °C denaturation for 30 s, 62 °C annealing for 30 s and 72 °C extension for 90 s. The PCR product was analyzed on a 1% TBE agarose gel, visualized by GelRed (Biotinum) and the 1.2 kb band was purified from the gel using NucleoSpin ExtractII kit (Macherey-Nagel). Purified DNA was sequenced by the Sanger method (Eurofins genomics) using the PTS TR1 R primer. BAC DNA was sequenced using primers MUC2 exon 30 F and R, MUC2 exon 28 F and R and MUC2 intron 28 (Table S3). The 8 TR of the BAC containing *MUC2* PTS-TR2-8TR was sequenced using primer CysD2 F.

### Sequence consensus assembly of RP13-870H17

A contig was made by aligning all *MUC2* Sanger sequences from exon 27 to the beginning of exon 30. The full DNA sequence for RP13-870H17 was assembled by aligning SMRT consensus sequences from NG-6768 and NG-7351 and sequences from Sanger contig using Vector NTI 9 AlignX (Life Technologies).

### Whole genome sequences and TR sequence alignment

*MUC2* and *MUC6* genomic sequences were downloaded from three different WGS projects (3–5) from NCBI. NA12878 was downloaded from BioProject PRJNA253696 (44x coverage) and PRJNA323611 (NBMU01000704.1; 75x coverage). Missing/additional bases were corrected by aligning the two. KOREF was downloaded from BioProject PRJNA294231 (154.7x coverage). Only the sequence for MUC6 was useful since the sequence for MUC2 was incomplete. NA19240 was downloaded from BioProject PRJNA288807 (73x coverage). The assembly of HX1, BioProject PRJNA301527 (103x coverage), representing a Chinese genome was shown not to contain any of the two mucin genes. Therefore these sequences were assembled by downloading the raw data (Unpublished results, T. Lang).

*MUC2* TR sequence alignments were made manually and by using AlignX (Life Technologies) by aligning shared unique repeats (referred to as unique repeats only present one time). *MUC6* TR units were aligned based on individual TR unit clustering using AlignX. All sequences can be found in the Mucin database www.medkem.gu.se/mucinbiology/databases.

## Electronic supplementary material

Supplementary Tables and Figures

## Data Availability

The final version of the sequence for RP13-870H17 consensus has been submitted to the NCBI GenBank under accession number MH593786. The nucleotide, mRNA and protein sequences are available at the Mucin Database at www.medkem.gu.se/databases.
