# Supplementary material for: The central exons of the human MUC2 and MUC6 mucins are highly repetitive and variable in sequence between individuals
Source: Sci Rep. 2018 Nov 30;8:17503. doi: 10.1038/s41598-018-35499-w (PMC6269512; doi:10.1038/s41598-018-35499-w)

Supplementary Figures and Tables to

The central exon of the human MUC2 and MUC6 mucins are highly repetitive and variable in sequence between individuals

Frida Svensson, Tiange Lang, Malin E. V. Johansson, and Gunnar C. Hansson

Tables 1 - 3

Table S1. Statistics summary of filtered subreads, **page 2**
Table S2. Amino acid composition of the TR in the different PTS. **page 3**
Table S3. Sequencing and amplification primers. **page 4**

Figures S1 - S11

Figure S1. Recombination of 1st and 91st repeat of MUC2 generates a 8TR variant. **page 5**
Figure S2. NG-6867 consensus nucleotide sequence. **page 6**
Figure S3. DNA sequence of *Hinf*I MUC2 PTS-TR2 fragment. **page 48**
Figure S4. Genomic sequence for BAC RP13-870H17. **page 50**
Figure S5. Complete MUC2 genomic sequence for Rp13-870H17. **page 93**
Figure S6. MUC6 genomic sequence (Reverse Complement). **page 103**
Figure S7. Complete MUC2 mRNA sequence. **page 113**
Figure S8. Complete MUC6 mRNA sequence (reverse complement). **page 117**
Figure S9. MUC2 PTS-TR2 units showing common and unique repeats. **page 122**
Figure S10. MUC6 Tandem repeats. **page 123**
Figure S11. Comparison of MUC2 PTS-TR2. **page 124**

Figures X1

Figure X1. Original autoradiograms of Southern gels presented in Figure 1. **page 125**

Table S1. Statistics summary of filtered subreads

Material	# Cells	Number of filtered Reads	Number of filtered Bases	N50 read length	Mean subread length	Mean filtered read score Polymerase read quality	Mean Covarage across reference	Poliched assembly consensus
**BAC DNA**	1	**68,011**	**353,937,792**	**7,678**	**5,204**	0,833	**1,168.21**	**149,943**
***HinfI* DNA Fragment**	1	**3,372**	**6,806,139**	**2,383**	**2,018**	0,83	**483.05**	**7,034**

For full statistics, see Supplementary Figure S3. N50, N such that 50% of the bases in the assembly are cantained in contigs≥ N.

Table S2. Amino acid composition of the TR in different PTS.

RP13-870H17

	MUC2 PTS-TR2		MUC6 PTS	
Amino acid	Number of residues per molecule of protein	Percentage of residues per molecule of protein	Number of residues per molecule of protein	Percentage of residues per molecule of protein
T	1251	55.5	1135	30.1
P	491	21.8	519	13.7
I	107	4.7	131	3.5
V	99	4.4	126	3.3
G	92	4.1	134	3.5
Q	92	4.1	81	2.1
S	71	3.1	672	17.8
A	15	0.7	217	5.7
L	13	0.6	132	3.5
M	12	0.5	66	1.7
K	5	0.2	56	1.5
N	4	0.2	43	1.1
R	2	0.1	45	1.2
H	-	-	276	7.3
F	-	-	86	2.3
Y	-	-	27	0.7
E	-	-	26	0.7
D	-	-	2	0.1
W	-	-	1	>0.1 (0.03)
C	-	-	-	-
Total	2254		3775	

CH17-246P12

	MUC2 PTS-TR2		MUC6 PTS	
Amino acid	Number of residues per molecule of protein	Percentage of residues per molecule of protein	Number of residues per molecule of protein	Percenmtage of residues per molecule of protein
T	1352	56.0	923	29.9
P	522	21.6	421	13.6
I	110	4.6	112	3.6
V	108	4.5	105	3.4
G	98	4.1	110	3.6
Q	98	4.1	64	2.1
S	73	3.0	553	17.9
A	13	0.5	176	5.7
L	13	0.5	106	3.4
M	13	0.5	56	1.8
K	4	0.17	47	1.5
N	5	0.2	37	1.2
R	2	0.1	39	1.3
H	2	0.1	227	7.3
F	-	-	68	2.2
Y	-	-	23	0.7
E	-	-	21	0.7
D	-	-	1	>0.1 (0.03)
W	-	-	1	>0.1 (0.03)
C	-	-	-	-
Total	2413		3090	

Table S3. Sequencing and amplification primers.

Primers, forward (F) and reverse (R)			
Location		Primer sequence	Application
MUC2 exon 30 (TR1)	F	5’- GTGCAGTGTGATGTCTCTGTT G -3’	Sanger sequencing
	R	5’- ACACTCACAGCACTGAACG -3’	Sanger sequencing
MUC2 exon 28	F	5’- CTGATGAGGTGATTCTTGGC -3’	Sanger sequencing
	R	5’- GTGTCGTAATGGAACAGATG -3’	Sanger sequencing
MUC2 intron 28	F	5’- GTAAGTGACGGTGATGATATTC -3’	Sanger sequencing
MUC2 PTS-TR1	F	5’- AGATACGTGTCAATTGTTGCTGGCC- 3’	PCR
	R	5’- CCAGAATCCAGCCAGCCAGTCC- 3’	PCR/ Sanger sequencing
MUC2 exon 30 (CysD2)	F	5’-GACTGGCTGGCTGGATTC- 3’	Sanger sequencing

Figure S1

Recombination of 1st and 91st repeat of MUC2 generates a 8TR variant

a

TR No.

VTQPTTMTTTTTEN
1. PTPTPITTTTTTVTPTPTPTSTQS
2. PTPTAITTTTTTVTPTPTPTGTQT
3. PTSTPITTTTTTVTPTPTPTGTQT
4. PTPTPISTTTTTVTPTPTPTGTQT
5. PTTTPITTTTTTVTPTPTPTGTQT
6. PTTVLITTTTTTMTPTPTPTSTKS
7. TTVTPITTTTTTVTATPTPTGTQT
8. PTMIPISTTTTTVTPTPTPTTGST

GPPTH

b

PTS-TR2 RP13-870H17 assembly																								
Repeat No																								
1	P	T	P	T	P	I	T	T	T	T	T	V	T	P	T	P	T	P	T	S	T	Q	S
	CCA	ACT	CCG	ACA	CCA	ATC	ACC	ACC	ACC	ACT	ACG	GTG	ACC	CCA	ACC	CCA	ACA	CCC	ACC	AGC	ACA	CAG	AGT
91	P	T	T	T	P	I	T	T	T	T	T	V	T	P	T	P	T	P	T	G	T	Q	S
	CCA	ACC	ACG	ACA	CCC	ATC	ACC	ACC	ACC	ACT	ACG	GTG	ACA	CCA	ACC	CCA	ACA	CCC	ACC	GGC	ACA	CAG	TCC
PTS-TR2 Repeat of 8TR variant																								
1	P	T	P	T	P	I	T	T	T	T	T	V	T	P	T	P	T	P	T	S	T	Q	S
	CCA	ACT	CCG	ACA	CCA	ATC	ACC	ACC	ACC	ACT	ACG	GTG	ACC	CCA	ACC	CCA	ACA	CCC	ACC	AGC	ACA	CAG	TCC

Figure S2. NG-6867 consensus nucleotide sequence.

```
   1 CTTCCCACCC ATACACTCAG CTCTCCATTT ATTCAGGATT AATTCAGCTG TTTGGGGAAT
  61 AAAGATGTGA AAAAGTTACC ATCTTTAACA ATCCCCTTGA CTGACTTCAG AATAGTGTTC
 121 CCTTTCATCC CCTCTGTCCA CCTGGGAATG CTTCTGATGC TGCCTGCATT ATCAGGAGGG
 181 GAAGAGGCCC AGCCAGTCTG CAGTGAACAT GCCGTGAAAG CCTGGACTCA ACATTTGCTA
 241 TTTGAAGTTA CAGACAGACT TTTAAGTACC TGACTGGATA TACCATGTGA ACATGCCGTG
 301 AAAGCCTGGA CTCAACATTT GCTATTTGAA GTTACAGACA GACTTTTAAG TACCTGACTG
 361 TATATACCAT CCAACCTGGG ACCCGGGGAT GGGAGAGCCC ACGCCCCTGA CTGGAGAGTT
 421 CCAGAGGCCT GGAAAGAACA CCCACAGTAG AAAACCCGCG GAAGAGGGGC CTAGCAAAAG
 481 ATTCATTAGG TATTATTATT ATTATTTTGA GATGGAGTCT CGCTCTGTTG CCCAGGCTGG
 541 AGTGCAGTGG CTTGATCTCA GCTCACTGCA AGCTCTACCT CCCGAGTTCA CGCCATTCGC
 601 CTGCCTCAGC CTCCTGAGTA GCTGGGACTA CAGGCGCCTG CCACCACACC TGGCTATTTT
 661 TTGTATTTTC TTATAGAGAT AGGATTTTGC TATGTTGCCG AGGCTGGTCT CAAACTCCTG
 721 GCCTTAAGGG ATCTGCCCAC CTTGGCCTCC CAAAGTGCTG GGATTACAGG CATGAGCCAC
 781 CATGCCTGGC CTTCATTAGA TATTATTATT ATTATTTTTT TAAAAAGTTT CTCCTGAAAG
 841 GCAGAGAGAA CACATAGTTT TATTTAAAAA ATACATTAGA AACAAGTATA TTTAGAAAAT
 901 ATATGCTTTT ATTATTTATG AATTTAAAAA AACGACTCAA TGAAACAAAA AGTGAAAGAC
 961 GAATAAAAAA GGTAACAGAC TGAGAGGAGA GAATCAGCCT GAGGCAGAGA TGAGGGGGAA
1021 CTAGATAATG TAAAGAAACC ACGATGGGAG AGTTAATATC TGTACTCAAA ACAGTAAAAC
1081 GGCAAGTAAC AAAAAACACC ATGTAAAATT GAGTAAGTAA TGTGGAAGAC AATTAAAGTT
1141 CTTTGAGAAT AGGAGGGAAA GAACAGAGAG ATTAAACTGG TGTGAAAGGC TCCTTCAGTG
1201 TTTGGGGACT AGCTCATATC AGAATAACAT GCTTGCCCTT CATAATCATA AACTCAAGAT
1261 GAAATAGAAA TGACACCTGT TTTGAAGACT GGTGAGTGGC TGGGCATGGT GGCTCATTCA
1321 TGTAATCCCA GCACTTTGGG AGGCTGAGGC AGGTGGATCA CCTGAGGTCA GGAGTTCGAG
1381 ACCAGCCTGG CCAACATGGT GAAACCCCAT CTGTACTAAA AATACAAAAA TTAGCTGGGT
1441 GTGGTGGCGC ACACCTGTAG TCCCAGCTAC TAGGGAGGCT GAGGCACCAG AGTCACATGA
1501 ACCCAGGAGG CAGAGGTTGC AGTGAGCTGA GATCGCACCA CTGCACTCCA ATCTGGACAA
1561 CAGAGTGAGC CTCAGTCACA CATACATACA CACACACACA CACACACACA CAACCCAAAA
1621 CAACAAAAAA CCACTGGTGA GAAATCAGAG GCAGGCAGCA TCAGAGGGGA TGCAAGCCTG
1681 GAAAAGGGAA ATAAGTGAGG TACGATCCCC ATTCAGCTGA CTTTCCACTG GAGGGAGCTT
1741 CCCTGTTTAC AGGAATGTGA GGGAGAGGGT GCCAGCTGAC TACAGCTCCT GAGCTGGGCT
1801 GAGAAGACTG AGGTCCAAGC TTAAGGTCTC CAGAGGCTGG AAATTAGGAA GGATGTTACA
1861 GGAAGAATAG ATCCACAAAT TTTTGACTGA GTTAAAAGTC ACATATAGGA TGAGACCTTA
1921 AACATCTGAG TGAAAGGAAA CAGCTGGAAG GCTGAAAGAT AGGGGCATGG GGTCACAGAG
1981 TTTGGAGTTC GAGTTGGCCC AGCTAGACCT CCTGGAGAAA CACCTTGCTT CCCATTACGA
2041 CTCTGTAAGA ACTGTATGTT AGGAGCAAGG ACCATCTGCT AGGACTAAAT GCTGTGCCCC
2101 AGGAACAAGG GCTAAACTGA AAGAGACCCA GATAATAAAG CCTGAGAAGA ATCCTCCTCA
2161 GGATCAAGTT GATCCACCAA AAATTTAACC ACCTGCTAAA CAAAACTTAG CATTCTCCTT
2221 CGGAAGATCA CAGAATCTAA AGTCTCCACA GTGTATCATA CACAATGCAC AGTGTACAAT
2281 AAAAATTACC ACAGAGGACA ATATAGCCCA CAATCAAAGG AACAAACCAA CCATCAGAAG
2341 CATATCCACA GAGGCCCATA TGTTGAAATT AGGAGATAAG TATTTTAAAT TAACCATTAT
2401 AAAAATGTGA CATAACATAC ATTGGAAAAT GAATATAATG GAAAAGAGGT GGGGAATGTC
2461 AGGAGAGAGA TGGAAACTCT AAAACAGAAC CAAATGAAAA TCTTGAAACT GAAAACAAAC
2521 AACACAACCA AATATCTGAA ATGAAATAGT CATTGAATTG GTTTAACAGA AGCTGGGAAA
2581 CTACAGAGGA AAAGATCAAG AAACTTGAAG AGAGGTTGAT AGAAACAATC CACACTGAAG
2641 AACAGACAAA ACAGTTTTTT GAAAAAGTCT CAGTGACTGG TGGGATGATA TTAAGTTGTC
2701 TGAGGAAGGA GAAGAAAGAG AAAAGGGGGT AAAAAAATATT AAAGAAAAAA TAGCCAAATG
2761 TTTTAAAATT TGACTAAAAA CATCAACTCT GAATTCCAAG AAGCAAATCT TCAGTGCAAT
2821 AAATATGAAG AAAACTACAC TTACGCATGT AATAGTCAAA CTACTGAAAA GAAAAGGTAA
2881 AAGAAAATCT TTAAAGCATC CAGGAAGAAA AAGACATAGC ACACAGAGGG AATAAATGAT
2941 AAAATGGTAG CTATAAATGC AGTCATATCA AAATTTAAAT TAATGTAAGT GGACTATACA
3001 CTCCAATTAA AAGACAGACT TTTAGACTGA ATCAATGAGC AAGACCCAAA GATATGCTGC
3061 TTACAAGAGA CATGTTGTGA CTATAGACAC AGATAAGGTT AAAAGTGAAA GGATGGAAAA
3121 ATGTATGTCA TGCAAGCAGT AATGATAATA AAGCTAGAGT GGTTACATCA ATATCAGACA
3181 AAGTAGATGT CAAGACAAGG AATATTACCG AGATAAAAGG ACATTTTATA ATTATAGAAA
3241 TGACAATTCA TTGAGAAAGC ACAATATTAC TGTGTATACA TCTAATAACA AAGCTTCAAA
3301 GTATACAAAG CAAAAATAGA CAAAACTTAA GGGTGAAACA GGCACAGATT TTAACATCAT
3361 TTTCTTGATC ATTGATAAAG CAAATAAACA CTCAGGAAGG CTGTTAGAAG TTCTGAAACC
3421 ACTATCAACC AACTTGAGCT AATTGACATT TATGGAACCC TAGTCAATGA CTGTAGAATA
3481 CACCATTCTT CAAAAGACCA CATGGATTGC TCACCGAAAT GGAACAGTGC TGGGCTATAA
```

```
3541 AACAAGTCTC AATAAACTTC TAAGGATTGA AATAATCAAC ATATGTTCTC TCACTAAAAT
3601 ACAAATTAGT TAAAAGCAGT AATGATAAGA TATTAAGAAA AACCTCAAAT ATTTAAAAAT
3661 CAAGAAGCAC AATGCATTCC AAACAACCTA TGGGTCAAAA AAGTCACATA AGAATTACAA
3721 CCAACTTCTC ATAGAAACAA AGGTGGCCAG AAGATGCCAG AAAGACATCT TTATAGTGTT
3781 GAAAGAAAAA AAAAAAAGCC TGTCAGTCAA CACTAAAATT TAGGTCTACA GGAAGGAATG
3841 AGGAGCACTA GAAATAACAA ATATGAGGGT AAGTACAAGT AAAATGTTCT TTTTTAAAAA
3901 CTTTCTTTAA GGGACTGTTT AAAGCAAAAA ATTAATTGTG GAAGTTTATA ACACATGGAG
3961 TAATTAAAAA CATCACACCA AATAGCACCA ACAATGGAGA AAAAATGAAA TTAGACTGTT
4021 CAAAGGTTCA TATATATACA TATATATACA CATATATATA CATATATACA TATATACACA
4081 TATATACATA TATACACATA TACACATATA TACATATATA CATATATACA CATATATACA
4141 TATATACATA TATACACATA TATATACATA TATACATATA TATATACACA TATATATATA
4201 CATATATATA TACATATATA ATTTTTTTTT TTTTGAGACA GTCTCACTTA CTCTGTCACC
4261 CAGGCTGGAG TGCAGTGGTG CGATCTTGGC TCACTGCAAC CTCCACCTCC TGGGTTCCAG
4321 CAATTCTCCT GCCTCAGCCT CCCAAGTAGC TGGGAGTACA GATCTGTGCC ACCACGCCTG
4381 GCTAATTTTT ATATTTTTAG TAGAGATGGG GTTTCACCAT GTTGGCCAGG CTGGTCTCGA
4441 ACTCCTGAAC TCAAGTGATC CACATGCCTT GGCCTCCCAA AGTGCTGAGA TTACAGGTGT
4501 GAGACACTGC ACCCGGCCAG GTTCTTACAT TTTACGTGAT GTGATATAAT ATTAATTCAA
4561 GATAGACTGA TAAGGATGCA TACTATAACT TTCAGAGAAA CTACTAAAAA TATAATGATA
4621 TCTAGATAAA AAGCAAATAG AAGTATTAAA ACTGAATATT GAAAATACTT AACTGACCAT
4681 ACTAAGTGTT GGCAAGAATG TGGCACAGCC GGAGCTCTCA GACAGTGCAG ACAAGACATA
4741 ATGTGGTACA ATCACTTTGG AAGGCTGCTT GAAAGTTTCT TATGAAATTA AACACATGCT
4801 TAGCATAGGA CTCAGCAATT CTACTCCTAG ACATTTACTC AAAAGAAATG AAAACGTGTG
4861 CTTACAAAAA GACTCGCACA AAAATGTTCC TGGCAGCATT ATTCGTAACT GTAAAAGACT
4921 GGGAAAGGCT CAGGTATTCC TCAAGAGGTG TATGGAATAA ATACATTGTG GTATAGCCAT
4981 ATGATGGAAT ACTATCCAGC AATAACAAGA AACAGTGAAA ACACACCACA ACATGGGTAA
5041 ATCTCAAAAA CATGATGTGT GAAAAAGGCC AGACACAGAA GAGTTAATAT TTTATGACTC
5101 CATTTGTCTG AAGTTCCATA ATAGACCAAA CTAATGTACA GTGACAAGAC TCAGACCCAT
5161 AGTTGCCTGG GTCAGGAATA AGAGTGGGGC CAGGCATGGT GGCTCAGGCC TGTAATCCCA
5221 GCACTTTGGG AGGCCAAGGT GGGCAGATCA CCTGAGGTCA GGAGTTTGAG ACCAGCCTGG
5281 CCAACATGGC AAAACCCCAT CTCTACTAAA AATACACAAA AAAGTAGCCA GGTGTGGTCG
5341 TGGGCACCTC TAGTCCCAGC TACTCAGGAG GCTGAGCCAG GAGAATCACT TGAACCCAAG
5401 AGGTGGAGGT TGCAGTGAGC CAAGATGATG CCACTGCACT CCAGCCTGAG CGACAGAGCG
5461 AGACTCCATC TTAAAAAACA AAACAAACAA AAAAGAGTAG GGTTGACTGG AAAGGGGCAT
5521 GAAACTCTTT GGGTGATGGG AACATTTTCT GGCTTGATCG CGGTCATGGT TCCATGGGTG
5581 TATACGTTTG CCAACACTCA TTGAATACCA TCATCCCTTG GCATCTGTTG GGGATTGGTT
5641 CCAGGACCTC CTGGAATACC TAGACAGTAT TTGCATAAAA ACTGTGCACA CCGTCCTGTG
5701 TACAGTTGGC CCTTGAACAA CATGGGGGCC AAGAGTGCCA GCCCCTCATG CAGGTGAAAA
5761 TCCAAGTATA ACTTTTGATT TCCCCCAAAC TTAACTACTA ATAGCCTCCT GTTGGCTGGA
5821 AACCTTACTA ACAACATAAA CAGTTGGTTA ACACATATTT TGTCTGTTAT ATGTATCATA
5881 GACTGTATTC TTATAATAAA GTAAGCTAGA GGAAAGAAAA TGTTATTATG AAAATCATAA
5941 GGAAGAGAAA ATATATTGAC TATGTATAAA GTGGAAGTGG GTCATCCCAA ACGTCTTCAT
6001 CCTGGTTGTC TTCTTGTTGA GTAGGCTGAG GAGGAGGAGA AGGAGGAGGA GGGGTTGGTT
6061 TCAAAGGTGG CAGAGGTGGA AGAGATGGAG GAGGTGGAAG GGGAGTCGGG AGAGGCAGGC
6121 ACACTTGGTG CAACTTTCGT GGAAAAAACT CCATGCATAA GAGGACACAC ACAGTTACAA
6181 CCCCTGTCAT TCAAGGGTTG ACTGCACTGT AAGTCATCGC TGGATTACTC ATAACACCAA
6241 AGACAACACC CACACACCCC TTCATTCCCG TGGATTCAGC GTGGAGCTCG GCTGTGTGAC
6301 CTCAACGTGT GACCTCAGTG GCAGATTCAG GTTTTGCTTT TTTGGAACAT TGTGACTTTT
6361 TTTTTCCTGA ATATTTTTGA TCAGCCGTTG GTTGAGTCAA CAGATGCAGA AGCCACAGAC
6421 ATGCATGGCT GGCTGTATTA ACTTAAAATG AATTCATCTT AAGCCAGGCA TGGCTTATGC
6481 CTGTAATCCC AGCACTTCGG GATAATAAGT GCTGGGATTA CAGCACTTAT TATAAGCCAG
6541 GCTCATGCCT GTAATCCCAG CACTTCGGGA GGCTGAGATG GGTGGATCAC TTGAGGTCAC
6601 GAGTTCAAGA CCAGCCTGGT GACCATGGCG AAACCCCGTT TCCAATAAAA ATACAAAAAT
6661 TAGCCAGGCG TGGTGGCGCG TGCCTGTAGT CCCAGCTACT CAGGAGGCTG AGGCAGGAGA
6721 ATCACTTGAA CCTGGGAGGC AGAGGTTGCA GTGAGCTGAG ATTATGCCAC TGCATTCCAG
6781 CCTCATGACA GAGCGAGACT CCATCTCAAA AAAAAAAAAA AAAAGAGTTG ATTTTATTAT
6841 GGGTAAATTA TATCTCAACA AATTTTATTT CAAAAGAAAA CAAGAATATC GATTCACTGA
6901 AAGGAAAGCA GGCAAAGAAG ATCGAAGGAA CAAAGAATGC ATGGGTTACA CATAAAGCAA
6961 AGTGCAAGAC GGTGGGCTGA ACTTGCCCTA CCTGTAAAAC AGCAATATTG AAGCGATGAT
7021 AAAATAAAAC ATGTCTGAGT CAAAGCAAGA CTTCATTTAT TCGGCTAGTT GAGAGTCTCT
7081 CACGTGTCAT TCATCAAGGT GCACAAAACC AGACAAGATC CTTCTTGTCA GTGGGATAAG
7141 AGTTTAGTGG GATCATAATA TTTCAGTTGT ATAATCTCAT CAATCAATGG GTAACTGCAG
```

7201 AAGGGAACAC ATTTCTGTGG GGAAATTCAA TACGGGGAGC TGAGCGGGGT GTAGGCAGGG
7261 GAGATGGCCT TGCACGGGGT GGCTGGGGGA GGCGGGGGTG GCTGGGGGAG GGAGGGGGTA
7321 GCTGGGGGAG GCGGGGGTGG TGGGAGGAGG CGGGGTGCTC AGACAGAAGC AGCTGCAGGG
7381 AAGCCCTGGG GTGGGGGAAC AGGCCCTGGA AAACTGCTAA GGCAATCAAT TAGAAAGAAT
7441 ATTTCCCATG CATTTTTCCT GCAAACAGAA AGGACTGGAA GACATACATC AGCCCATCAA
7501 GATACAATCT CAGCAAAAAA CCACAAAAGC GGCAGACAAA ACCAAACCTC AAGAAAGGCA
7561 AAGTTGTACA TTCAGAAGGT CTGGAATGAC TGGGTTTAGG GGTCATCCCG GCCATTTGCT
7621 GCAATGGCTG CCACAGATTG CAAAGAGCAC AGTCAATTCT TGGAGGAGGA CAAGGGGAGG
7681 TGTCATGTTT ACAATCCAGA GTGCACAGCC CAGAACTGAG ACCTGGAAAG GAGGGGCCAG
7741 CAGGAGGAAG GGAAGGGGTA GTGACGTCTC TTCCACACGC ACAAGCAAAT CAGGGTCACT
7801 CATAGAAACA CAGATCCCAG GGTTAAAAGG ATGACCTCTA TAGTCATGAA AACAGAGTGA
7861 AGACCTGCAA GAGGAAACGG CAAAGGGAGC ACAAACCCGG CTGCACAAGA ACAGACCGGA
7921 GAAAGGTCAG GACTCTCAGG CCAGGGACTG AGGAGCCAGC GATAAGCCAT GGCAGTAAAG
7981 GCTGACTGAA TGCCCCTATG AAAAGGAGGC TGGAGATGGG GCTGAAAAGA AAAGAGCACG
8041 CCCACAGCCT CACTGCACGT TGTTTGAAAG AGACACAGCT AAAACGAATG CCCCAGCAAA
8101 GTTAAAATCG AAAGGCAACA TGGCCGGGCA CAGTGGCTCA CGCCTGTAAT TCCAGCACTT
8161 TGGGAGGCCA AGGTGGGTTT GAGGTCAGGA GTTTGCGACC AGCCCGGCCA ACATGGTGAA
8221 ACCCCGTCTC TACTAAAAAT ACAAGAATTA GCCGGGCGTG GTGGCGCGTG CCTATAATCC
8281 CAGCTACTCG GGCAGGAGAA TTGCTTGAAC CTGGGAGGTG GAGGTTGCAG TGAGCTGAGA
8341 TTGCAGTGAG CTGAGTGCCA CTGCACTCCA GCCTGGGTGA CAAGAGCACA ACTCCATCAA
8401 CAACAACAAC AACAAAAAGC AACAGGATAT GAGACAAAAG CCAGCAGAGG CAGAGCTGTG
8461 GCCGTAGTAT TAATAAATCG AAAGGCGGGG TTAAGATATG AACACCGAAC ACGACAAAGA
8521 GGATCAGTTT GAGTTTATAG AAGAAGCAGA AGAAACAATG GTGATTTATT TTGATTTTTA
8581 CTCATTAAAA AACATAGTGC TGCTGCTCCC AGATGATGGC ATAGCTCTTC TTTTCCCTGT
8641 CTTCCTCCTA AGTACAAAGA AGAATCCTGA ATATTAGACA TGAAAGAAAT GTAGGAAGGC
8701 TCCGGAAGGT GGAGAGGAGA GGGCAGACCA GTCGGGGACC TCGGGCCCCA GGAAAGACCC
8761 ATTGCAAGTT CCCTGGGTTT TGCTTTTGCC TCATGGATCC CAGGCCTGGA GCTGGAGAAG
8821 CTGGCAGTGT GGACACTTCA ACACATGTAC ACCAAAAAAA GGCCCCACCA GTCCCCGCCT
8881 CCTGGAGCCA GCGTGATCAG GAAAGTAGCT GCCCGGCAAA GCAGAAGGCT TTTAGGTGAT
8941 AACTGCCCTG CTCCAGCTGA ACACCACGGA AAACCTGCAC TCCACTGGAG CAAAGTCTGG
9001 GCAGGGCTGG GCAGGGCTGG GCACCCACCC TGGTGGCTGG AATGAGGCGC CCCAGGTGGC
9061 CCCAGGGCTG GTGTCCAAGT GGGCACAGCA GGAGGCTGGG GTCATCCCAG TGGGTGGTTC
9121 TGAACCGCTT TCTCTGTGAC CAGTGATGGG GATGGTGGAG ACCACACGGG GTTAGGGAGC
9181 TTGGATTTCC ACCCCATCCC TGGTGTTGAA ACACTCCTCC TCTTCCCCAT GAGGAGTGTC
9241 AGAGGAGGCT GAGCCGAGAG TCATGACTTT GACCACTGCT CAGTGTAACA GGGCCCCCTC
9301 CATCATGCCC GTGGAGGGAA TGAGAGCAGC AACGAGGCGC CATGCCCCTC CCAGCCAGAA
9361 GGCATCGGTG AGGGCTCCAC CCTGCCCGGC AGGCATGGAC AAAACCCCTG GGGTGTTAAG
9421 AGAGGGCCGG TGGGGAATGA GCACTTCCAC ACCACAAGCT GCCCCTTCCC TTCATTAATC
9481 TGTGCCAGAG GAAGTCAACG AGAAAGGTTT AAATAAGATC CAGAGTCTCG TAGCAATCTC
9541 CAAAATGCCC AGGTTTCAAT AACAAATTGC TCATCATCCC AAGACCCAGG AAGATCTCAG
9601 ACTGAATTCA AAAGGACAAT CAGCAGAGGC TGACCCTGAG ACAAAGAGGT GTTAGAATGA
9661 TCTGGAAAGG GTTTTTGAGA AGCCCATAAA GTGCTTCATT TGTGAACAAA TTGAAACCAA
9721 TTTAAAAATA GAAAGTCTCA ACAGATAATA GAAAATCCCA GCACAGAAAC AGAACACACA
9781 AAGACGAACT GAATGGAAAT GGTAGAACTG AAAATTACAA TAATTGAAGA GATAAAAAAC
9841 AAAAACAAGA CTTCAATGAA TGGGCTCAAC ACAGAGTAGA GGGAGAGTCC AGTGAGAGGA
9901 AGAAACAGTG AGCAGGGAGG TACAATAATA GACGTTATCA GACCCATCAG CGAAGAGAAA
9961 ACAGACTGAA CAGGATGAGA GAAACACACA GAGCCTCAGG AACCATAGGG CTAGAACAAG
10021 AGACCCAGCA TTCCAGGTGT TGGGGTCATG GGAGGAGAGA AAGAGGATGG GGATGGAAAA
10081 GAGCTCAAAG ACCTAAGGTT GAAAACTCCC CAAACTTGCC AAAACACCCA CAGATTCAAG
10141 AAGCTCGATG AACCCCTAAC AGGATAAACC CAAGAAAATC CACAGCAAAA CATCTCGTAG
10201 TCAAACTTTT GAAAACTATA AAAACAAAAA ATTTTGGAAG TAGCAGGAGA GAAACAACAT
10261 TTTACATATA AGGGAAAGAC AGTTTGAGTG GCATTTGATT CCTCATCAGA AACCCCAAAG
10321 GCCAAAAAGA GCACAACATT TTTCAAATGC TGACAGAAAG GAACTGTCAG CCCAGAATGC
10381 TATATTCAGT GAAAGTATCC CCGAAGAAGG AAGAGGAAGT TAAGGCATTT TCAGATGAAG
10441 GAAAAGTAAG AGAAATTTCC ACCAGAAGAT GTACCCTGAA AGCTTCAAAA TAGTTGAACC
10501 AAATCTGATA AAACTGAAAG GAGAAATGCA CCCATTTCTA ATTATAGCTA GAGACATCAA
10561 CACCCCTGTC TAAACAATTA ATAGAACAAC TAGGCAGAAA ATCAACAAAG ATGTGGAAAA
10621 ACTTAACACC ATCAACCAAC AGGACCAACA TTTGTAGAGC TCCACCCAAC CACAGAACAC
10681 ACATTCTTTT CAAATGTGCA CGGAACACAT TCCAGGAGAG GCCGTGTCCT GGGCCTGAAC
10741 ACAAACTTTA GCACATTTCA CATACTGACA TCACACTCCA GCATGTTCTC TGACAACAAT
10801 GACATCAAAT TAGAAATGAA CAACAGAAAG TTAACAGGAA AATCTCCAAA CATTCAGAAA

10861 CTAGAGAACA GACCTCCAAA TAATCTGTGG GCCAATGAGG AAGTCTCAAG GGAAGTTTTA
10921 AAAAACAACA ACATTGAACT CAATGAAAAC GAAAATGCAA CATATCAAAA ATTGAGGGAC
10981 ATAGCCAGGC ATGGAGACAT GCAACTGGGG TCCCAGCTTC TCAGGAGGTT GAGGTGGGAG
11041 CATCACTAGA GGCTAGGAGT TTGAATCTAG CCTGGGCAAC ATAGCAAGAC CCTATCTCTG
11101 AAAAATAAAA ATAAAAAAAA TTAGAGAGAT ACAGTCAAAG CAGTGCTGAG GGAAATTTGT
11161 AGCAGTAACA GAACACACTA GAAAAAAGGA TGAGTCAAGT CAGTAATCTA TGCCACCCAC
11221 TGAAGAAATG AGAAAAAGAA GCACAGGTTG CACACAGAGC AAACTGAAGG AAGGAAAGAG
11281 TAAAGACCTC AGTGAAACCG AAAGCAGGGA GACAGTGGCA GAAAGAAAAA TACCAATAAC
11341 ACGGACAAGG CTCTAGCATT ACAGCAAGAA ACAAGAGCAA AGACACAAAT GACCAAGGAC
11401 AGGACTAAAA CAGGAGCAAT CACTACAGAC CCTGCAGATA TCATGAGGGT GACAAGGGGC
11461 TGTGGCACAC AACTCTGCAC ACAGAACTTT GGCACCTTAG ATGAAATATG CCAGTTCCTC
11521 AAAAAGCACA AGTGATCACA ACTCAGCTAA CACAAATAGA TAACTGGGCA GCCCAATAAA
11581 TGGTTGAGAA CATTGAATTT ATAATTTTAA ACTCCAAAAG AAGAAATTCC GGGCTCAGAT
11641 GAGTTCAGCG GTGAATTCTG TAAAAACTTT AAAGAAGAAT TAACACCAAT CATACATAAT
11701 TCTTCCAGAA TGTAGAAGAG GAGGGCGTCT GTATGACCCA GATACTGAAA TCATACAGGC
11761 AGGAGAAAAA CAAAGCAAAT CCAAACCAGA ACTATGGACC AACACCCCTC ATGAAGATGG
11821 ATACAGACTC TAACAAAATA ACAGCAAAGA GAATTCAGTG CTATATGGAG CATTATCCAT
11881 CTTAACCAAG AGGGATCTAT TCCAGAAATG CAAGGTGGCC AGGTCAATAT TTAAAAAATT
11941 AATGCAACCA GCCATATTAA CAGGCCAAAG AAGAAAAATT GCACAAGCCT ATCAATCAGA
12001 GCAGCAAAAA CATTTGAAAA GACTCAACAT TCATTCTTAT AAATTCTCAA AAAATAAGAA
12061 TACAGGGAAT TTCCTCAATG AGATAGAGAA CAGCTACAAA AAAAGGAAAC CAAAAAGCAA
12121 AAACCACTCC TGCTAACATT GTACCTAATG GTGAAGGCTG AGTTCTTCCT GTCTGACACT
12181 GGGAGCACGG TGAGGGTGTC CCCTCTCATC GCTCTTATTC AACGTACTGC TGGGAGTTCT
12241 GCCAGTGCAA TAAAGCAAGA AAAGGAAATA AAAGGCAATT CGTTTGGAAG AAATAAAGCC
12301 GTCCCTATTT TTAAGTGACA TGATTGTCTA TATAGAAAAT TCCAAAGATA CTGAAATTTA
12361 AAAAAATCCA AGACTAATGT CTGTGCAGAA AGGTCAAAGG ATACAGGATA AACACACAAC
12421 AATGAATAGT ATTTCTATTT AGTAGCAATG ACCATGACTT CATCGACTTT AAAAATATAC
12481 CACCATTCTC AATCACGCAA ATAACTTGAA ACATGTAGGC GTAAATCTAA CCAAACGTGG
12541 AGGACTTGTG TGCTTAAAAC TACAAAATGC CGATGAAAGA AATCTTTCTT TTTTTTCATT
12601 TAGAATCCAT TTTTATTCCC ACAAACAGTT CTGAAAAATA TTAGAATTGG CAAATGGTTT
12661 ACCATGAATG GAAAAAAAAA ACCCATTGAT TTTCCTTTTT TCTTTTTTTT TGCTTAAATA
12721 ATTTTATTAT TTTTATTTTA TTTTTCCATA AGTTACTGGG TTGTATTTGG GTATATGAGT
12781 AAGTTCTTTA GTGGTGATTT GTGAGATTTT GGTGCACCCA TTACCTGAGC AGTATACACT
12841 GCACCATATT TTTTGTCTTT TATCCCTCAC CCCCTCCCAC TCTTCCCCCC AAGTCCCCAA
12901 AGTCCATTAT ATCATTCTTA TGCCTTTGCA TCCTCATAGC TTAGCTCCCG CATATCAGTG
12961 AGAACATATG ATGTTTGGTT TTCCATTCCT GAGTGACTTC ACTTAGAATA ATAGTCTCCA
13021 ATCTCATCCA GGTCACTGCA AAAGCTGTTA ATTCATTCCA TTTTATGACT GAGTAGTATT
13081 CCATCAGATC TATATCTATA TGTATATCTA TACCTATATC TATATCTATA TACCATCACA
13141 GAGCAGGTAA ACCTGTAGGG GTGCAAAACA GATATGTGGT GGCTGGAGAC TGGGAGAGGG
13201 GACGGGGTTG GCTGTACAGG GCACGGGGGA CCTCGTGGGG TGACCGAGCG ACTGTCTCTG
13261 AGTGTGGTGA TGGTTACCTG ACTGTGTGTT CATCACAGCT CCCAGAACTG TAACCCTAAC
13321 AGGAGTGGAT CTTACTGTAT GCAAATTATA TCTTAATAAA AAATGAAAGA TATATGTAAA
13381 GTTGATGTCG GAAAAAGTGC AGAGAAATTT TAAAAAGTTC AGTTATAGTA TCTTACTCCA
13441 TGACAGATAG AGTAGGCAAG AGGTGGAGAG AAGATTTGAA TTATGTAATT AATAAGATTT
13501 ATTTACACAT TTATTTCAAA CTTTGTTCTC TAATACAGAT AATACTCCTT CATCTTAGGC
13561 ATCCATAGAA TAGTTACAAA AATTAATCTT TTTTACATGT ATTTTTTATT TTCAAGGTAT
13621 AATTTGCATA CAGTAAAATT CACTCTTGTT AGTGTGTAAT TCTGTCCAAT TTGTTGTCTA
13681 CGGAATTGGC TGCAGTTTTC TCTGGGGCCT AATGTGAGAT TAATTTTTGT GGCTATGGTC
13741 TCTAACAAAA TACAACTGAA AATTGATAAC AAACATGTGA ATAAGACAAA AGAGGCTGGG
13801 CACAGTGGTT CACGCCTGTA ATCCCAGCAC TTCGGGAGGC CGAGGCGGGC GGATCACGAG
13861 GTCAGGAGAT CGAGACCATC CTGGCTAACA CGGTGAAACC CTGTCTCTAC TAAAAATACA
13921 AAAAATTAGG TGGGCGCGGT GGTGGGCGCC TGTAGTCCCA GCTACTCGGG AGGCTGAGGA
13981 AGGAGAATGG CATGAACTCG GGAGGCAGAG GTTGCAGTGA GCCGAGATCG GGCCACTGCA
14041 CTCCAGCCTG GGCGACAGAG CGAGACTCCG TCTCAAAAAA AAAAAAAAAA AAAAAGACAA
14101 ACAGCCTAAG CGGCTGGGGG TGGACACGGC AGGGCTGTCT CTGGCCTCTT TCCTGTCTCT
14161 GGCGTCTCTC CTCTCCCCTC CCCTGGACCC TCCTTATTCT GCAAAACGGG CCATGGGCAC
14221 TTTCTGGGAA ACGTCACTTT GCTCAAACCG TGTTTGCAGA TCCAGGACCC CTTAAGAGAT
14281 GTTACTTTGC ATAAAGTGTT TGCCACTTAG GTTTTCCCTA AGGGATGTTA TGTGTTTAAG
14341 CTGCCATTAA GGTGCAGAGT GGTAAACCTG TTACAGTCAC GTGTGGTCCG CAATTGCCAC
14401 CAAGACCATC CTGGCTGTGT GGAGCCGGGC TGCTGCCGTG GCAAAGGATC ACAGCCCTGT
14461 GGCTGAGCAC AGCACGCTCA CGCCGTGCGG TCCGGGGGTG CCAAGTCTCC AGGGAGCTCG

14521 CAGGACTGCG TTCCTTCCAG AGGCTCCAGG GGAGGGTTCA CGTCCTCGCC TCTTCCCGCT
14581 CTGGAGGCCG CGTCTCTTCC CCGGCGGACC GTCCTCTACC TGCAGAGCCA TCGGGAGCCT
14641 CTGCTCTCCC CGACTGCCTC CCTCTCCTCC CGCTGTCTCT GTCTGCCTGA CCTCTTTCTC
14701 TGACCCTCTT GCTTCTTTCA CAAGAGCCCT TGTGGTTACT TCGGGCCCAT CCAGATAATC
14761 CAGGACAACC TCCCCACCCA AGGCCCATCA CTCTGTCCAC CAGGTCCCTT CCCCCATGGC
14821 TCCAGGGTTT AGAATGCGGG CATCTCTGGG GCCATCATTC AGGCTAATGC TGTCAGTATA
14881 TTAAAAACTG CATTTCTATA TGCTAGCAAC AAACTAGTAG AAAAGAGCAT CAAAAAACTG
14941 CTACTGCCTA GAAATAAATT TAACAAAATG TCCCCATGAC CTCTACACTA AAAACTGCAA
15001 AGAATTGCAG AAATTCAAAA GGACCAAAAT ATGTGGAGAT GTATTAATAT AGTTGAAGAC
15061 TCAATATTGT TAAAATGTCA TTTTCCTCCA AACTGATCGG TAAATTCAAT CCCAATAAAA
15121 AATCCAGTTT TTTTTGGGTG GACATTGACA AGCGCCTTCT AAAATTTATA CGGCCATGGA
15181 AAAGACCTGG GATAGCCATG ACGATCTTGA CGAAGCAGAA AAAAACATTT CCAAGACTTA
15241 GAATGAAGCT CTTAGTCGAG ACGGAGTGAC ATTGATATAA ACTTAGACAT TCAGATTAAT
15301 GAGACGAACT GAGAGCACAG AAATAGGCTG TCACCTGTAC CGGGCAAACA CACCGAGGAA
15361 AACCAAGAGT GCCAGTGTGT GTGTGTGTGT GTGTGTGTGT GTGTGTTCAC AGGGTTATGT
15421 GTCTGTATGT GTATGTATTT GTAGGCTTGT GTATGTGTTT CTGTGTTTGT GTGTTTGCAG
15481 GGTTCTATAT GTGTTTGCAT GTGTGTGTAT GCAGGTTGTG AGTATGTGCT TGCGTGTTTG
15541 TGTGTTTGAA TGGTTGCATG TGTTTGCATG TGCGTGTGTT TGCAGGGTTC TGTGTGTGCA
15601 CATCTGCTTG TTTGCATGTG TGTTTGCAGG GTTGTGTCTC TGTTTGCATG TGTGTGTGTT
15661 TGTGTATGTA TTTGCAGGGT TGTGTGTGTG TCTGTTTGCA TGTGTGTGTT TGCAGAGTTA
15721 TGTATGTGTG TTTCCATGTT TGTGTGTTTG CAAGGTTCTG TGTGTGTTTG CATGTATGTG
15781 TATGCAGGTG TGTGTGGGCT TGTGTGTTTG CATGGTTGTG TGCGTGTTTG TGTGTGTGCT
15841 TGCATGTTGT GTGTGCTTTG TGTGTGTGCT TGCATGGTGT GTGTGCTTGA TGTGTGTGTG
15901 CGCTTGTGTG TGTGTGTTTG CATGGTACGT GTGTGTGCTT GTGTGTGTGT TTGCATGGTA
15961 TGTGTGTGTG CTTGCATAGT GTGTGTGTGC TTGCATTGTG TGTGTGTGCT TTGTGTGTGT
16021 GCTTACATGG TGTGTGTGTG CTTTGTGTGT GTGCTTGCAT GGTGTGTGTG TGTGTTTGCA
16081 TGGTGTGTAT GTGCGTGTGT GTGTGTTTGC ATGGCATGTG TGTGTGCTTG TGTGTGTGTG
16141 CTTGCATGGT GTGTGTGTGC CTGTGTGTGT GTGCTTGCGT GGTGTGTGTG TGTTTGCATG
16201 GCGTGTGTGT GTGCTTGTGT GTGTGTGCTT GCATGGCGTG TGTGTGTTTG CATGGTGTGT
16261 GTGTGCTTGC ATGGCGTGTG TGCTTGTGTG TGTGTGCTTG CATGGTGTGT GTGTGTGTTT
16321 GCATGGTGTG TGTGCGCTTG TGTGTGTGTG TTTGCATGGT GTGTGTGTGC TTGCATGGTG
16381 TGTGTGTGCT TGTGTGTGTG TGTTTGCATG GTGTGTGTGC TTGTGTGTGT GTGTTTGCAT
16441 GGTGTGTGTG TGTTTGTGTG TGTGTGTTTG CATGGAGTGT GTGTGTGTTT GCATGGTGTG
16501 TGTGTGTTTG CATGGTGTGT GTGCGCTTGT GTGTGTTGTT TGCATGGAGT GTGTGTGTGT
16561 TTGCATGGTG TGTGTGTGTG GACTGCTACT GCTGCCATGT CGCTGCAGTT CAATGGGTTT
16621 GTGCGTTGTG ATCACACGGT CCTGCCCACA GAGCTGAATG GGCTATTGGG GTTTCCTTGG
16681 GTGAGTCCCA GGCTGTGTGT GGGGTGAGTG TGGGGCCTTT CTTGCACCGT TTAGCCTGGC
16741 ACGCGTTGCC CTCTCTCTCT CTCCCAGGAC CACATTCAGG TGCCAGGGCC TTTAATCCTG
16801 TGTCCCTTGT CCTACTTACT CCCTCTCCCC AGACTGTGGG ACAGGCACCT GGGGCGCACT
16861 GTGGGGGCTC TGAGATGCAG GTGGGTGGGC CAGGCCTGCC GAGGGGAGGT GAGGCGAGCA
16921 GAGCCCTTTG AGGGTGATGG AGCTTGGCTG GCCCCATGGC CCGAACCCAC CTGGCCTAGG
16981 GGCGTGGCTC AGAGCAGGTG CACACAGGGC TTATTCACCT GTGCATTCTT CAAACAGTCC
17041 AGGTGTGAGT GTGAGGCTGT GAACACACCG GGGGTGTGTC TGCCAGGTGG GGCCCTGGAC
17101 GGGGTGGCCC CCAGCACCTC TGGGTTTCCA GCCCCCAAAA GTGGGTGTCT CTGGGCCCAC
17161 CTGCTCCTGG AGCTGGGCTC GCACTGACCC TGGGAAGACA GCCTCCCCTG GGCATTTTGG
17221 GGACGCTGGG CCCAGCCCAT GCCTCGTGGC TCAGCTTCCC TCAGCCAATA CTTTCGAGAC
17281 ATGTATTTCG AGACATCCCT GGAGACATGG CCAGTATATT TGTATTTTAT ATGAACCCGG
17341 AAGGCTGGAC CTGAAGAGGC CCCCTGGGTG AGGGGGCCAG GCTGAGCTCG AGTTCCCCAG
17401 GGCCTCCAGC AAGAAAGGCA CCCAATGCCT CCTTACAGGA GGAGCAGGCG CATGTCCACA
17461 GAAGATCTGA AAAGACCCCA TCAAAATATT CGTGGGTGTC TTCTCAAGAC GAGGGGGGCA
17521 ATTTTCGCTT TACATTTTGT TCCAATTTTT TATTGTTGTA AAAAACACAT AACATAAAGT
17581 TGACGTTTTT TGCCACTGCT GGGTGCACAG TGCAGTGCTG TTAGGACATT CACACTGCCG
17641 TGCGGCCCGC AGAGCCACCA TCTCCAGAAC ATCTTCGTCT TGCAGAACTG AAGCTCTGTC
17701 TTGTTAAACA CCAGGTCCCC ACTCCCCGTG CCCAGCCCCT GCCCAGCCCT GGCGGCCACC
17761 ATCTGCTTTC TGTGTGTGTG GATTTGGCGG CCCCAGCCGC TGGTGTAGTG GAACCACGTG
17821 GCGTTTGCCC TTCCGTGGAT GGCTTGTCTC TCCAAGCCAA TGTCCTTGGA TCCGCCTACA
17881 CTGCCGCCTA TGCCAGGGCC TCCTTCCTTT TGGGGGCTGC TTGGTGCTCC CCGGCACAAT
17941 GGGCCTCACC TTGTTTTCCC CCAGATTCGT GGACAGACAC CAGGCTGCTT CCACCTCTTG
18001 GCTGTGGTCA ACAAGACTGC TGACCCTGCT TTCAATTCTT TGTGATTTTG CACTGAGAAA
18061 AATACCTTTT AAATGCACGG TCAGAATTTG GTGGACGACA CCCCACCCTG CGTATGCATT
18121 TGGTGGACGC CCCACCCTGC GCATGCATTT GGTGGACGCC CCACCCTGCG TATGCATTTG

18181 GTGGATGACG CCCCACCCTG TGTTGATGCC CCTCTGTCCT CCCAGGGGTC AGAGGTGCTA
18241 ACCTGTGCCC CAATGCCCTG GTTGGTCTGG GCCCCTGGAA ACCACAGCGA GGGCAGCACG
18301 CTCTGGCTGG TCCTCTCCCC GAGGCCGACC CGGATGGGGA TCCTTCTATA GTGTCACCTA
18361 AATGTCGACG GCCAGGCGGC CGCCAGGCCT ACCCACTAGT CAATTCGGGA GGATCGAAAC
18421 GGCAGATCGC AAAAAACAGT ACATACAGAA GGAGACATGA ACATGAACAT CAAAAAAATT
18481 GTAAAACAAG CCACAGTTCT GACTTTTACG ACTGCACTTC TGGCAGGAGG AGCGACTCAA
18541 GCCTTCGCGA AAGAAAATAA CCAAAAAGCA TACAAAGAAA CGTACGGCGT CTCTCATATT
18601 ACACGCCATG ATATGCTGCA GATCCCTAAA CAGCAGCAAA ACGAAAAATA CCAAGTGCCT
18661 CAATTCGATC AATCAACGAT TAAAAATATT GAGTCTGCAA AAGGACTTGA TGTGTGGGAC
18721 AGCTGGCCGC TGCAAAACGC TGACGGAACA GTAGCTGAAT ACAACGGCTA TCACGTTGTG
18781 TTTGCTCTTG CGGGAAGCCC GAAAGACGCT GATGACACAT CAATCTACAT GTTTTATCAA
18841 AAGGTCGGCG ACAACTCAAT CGACAGCTGG AAAAACGCGG GCCGTGTCTT TAAAGACAGC
18901 GATAAGTTCG ACGCCAACGA TCCGATCCTG AAAGATCAGA CGCAAGAATG GTCCGGTTCT
18961 GCAACCTTTA CATCTGACGG AAAAATCCGT TTATTCTACA CTGACTATTC CGGTAAACAT
19021 TACGGCAAAC AAAGCCTGAC AACAGCGCAG GTAAATGTGT CAAAATCTGA TGACACACTC
19081 AAAATCAACG GAGTGGAAGA TCACAAAACG ATTTTTGACG GAGACGGAAA AACATATCAG
19141 AACGTTCAGC AGTTTATCGA TGAAGGCAAT TATACATCCG GCGACAACCA TACGCTGAGA
19201 GACCCTCACT ACGTTGAAGA CAAAGGCCAT AAATACCTTG TATTCGAAGC CAACACGGGA
19261 ACAGAAAACG GATACCAAGG CGAAGAATCT TTATTTAACA AAGCGTACTA CGGCGGCGGC
19321 ACGAACTTCT TCCGTAAAGA AAGCCAGAAG CTTCAGCAGA GCGCTAAAAA ACGCGATGCT
19381 GAGTTAGCGA ACGGCGCCCT CGGTATCATA GAGTTAAATA ATGATTACAC ATTGAAAAAA
19441 GTAATGAAGC CGCTGATCAC TTCAAACACG GTAACTGATG AAATCGAGCG CGCGAATGTT
19501 TTCAAAATGA ACGGCAAATG GTACTTGTTC ACTGATTCAC GCGGTTCAAA AATGACGATC
19561 GATGGTATTA ACTCAAACGA TATTTACATG CTTGGTTATG TATCAAACTC TTTAACCGGC
19621 CCTTACAAGC CGCTGAACAA AACAGGGCTT GTGCTGCAAA TGGGTCTTGA TCCAAACGAT
19681 GTGACATTCA CTTACTCTCA CTTCGCAGTG CCGCAAGCCA AAGGCAACAA TGTGGTTATC
19741 ACAAGCTACA TGACAAACAG AGGCTTCTTC GAGGATAAAA AGGCAACATT TGCGCCAAGC
19801 TTCTTAATGA ACATCAAAGG CAATAAAACA TCCGTTGTCA AAAACAGCAT CCTGGAGCAA
19861 GGACAGCTGA CAGTCAACTA ATAACAGCAA AAAGAAAATG CCGATACTTC ATTGGCATTT
19921 TCTTTTATTT CTCAACAAGA TGGTGAATTG ACTAGTGGGT AGATCCACAG GACGGGTGTG
19981 GTCGCCATGA TCGCGTAGTC GATAGTGGCT CCAAGTAGCG AAGCGAGCAG GACTGGGCGG
20041 CGGCCAAAGC GGTCGGACAG TGCTCCGAGA ACGGGTGCGC ATAGAAATTG CATCAACGCA
20101 TATAGCGCTA GCAGCACGCC ATAGTGACTG GCGATGCTGT CGGAATGGAC GATATCCCGC
20161 AAGAGGCCCG GCAGTACCGG CATAACCAAG CCTATGCCTA CAGCATCCAG GGTGACGGTG
20221 CCGAGGATGA CGATGAGCGC ATTGTTAGAT TTCATACACG GTGCCTGACT GCGTTAGCAA
20281 TTTAACTGTG ATAAACTACC GCATTAAAGC TTATCGATGA TAAGCTGTCA AACATGAGAA
20341 TTGATCCGGA ACCCTTAATA TAACTTCGTA TAATGTATGC TATACGAAGT TATTAGGTCC
20401 CTCGACTATA GGGTCACCGT CGACAGCGAC ACACTTGCAT CGGATGCAGC CCGGTTAACG
20461 TGCCGGCACG GCCTGGGTAA CCAGGTATTT TGTCCACATA ACCGTGCGCA AAATGTTGTG
20521 GATAAGCAGG ACACAGCAGC AATCCACAGC AGGCATACAA CCGCACACCG AGGTTACTCC
20581 GTTCTACAGG TTACGACGAC ATGTCAATAC TTGCCCTTGA CAGGCATTGA TGGAATCGTA
20641 GTCTCACGCT GATAGTCTGA TCGACAATAC AAGTGGGACC GTGGTCCCAG ACCGATAATC
20701 AGACCGACAA CACGAGTGGG ATCGTGGTCC CAGACTAATA ATCAGACCGA CGATACGAGT
20761 GGGACCGTGG TCCCAGACTA ATAATCAGAC CGACGATACG AGTGGGACCG TGGTTCCAGA
20821 CTAATAATCA GACCGACGAT ACGAGTGGGA CCGTGGTCCC AGACTAATAA TCAGACCGAC
20881 GATACGAGTG GGACCATGGT CCCAGACTAA TAATCAGACC GACGATACGA GTGGGACCGT
20941 GGTCCCAGTC TGATTATCAG ACCGACGATA CGAGTGGGAC CGTGGTCCCA GACTAATAAT
21001 CAGACCGACG ATACGAGTGG GACCGTGGTC CCAGACTAAT AATCAGACCG ACGATACGAG
21061 TGGGACCGTG GTCCCAGTCT GATTATCAGA CCGACGATAC AAGTGGAACA GTGGGCCCAG
21121 AGAGAATATT CAGGCCAGTT ATGCTTTCTG GCCTGTAACA AAGGACATTA AGTAAAGACA
21181 GATAAACGTA GACTAAAACG TGGTCGCATC AGGGTGCTGG CTTTTCAAGT TCCTTAAGAA
21241 TGGCCTCAAT TTTCTCTATA CACTCAGTTG GAACACGAGA CCTGTCCAGG TTAAGCACCA
21301 TTTTATCGCC CTTATACAAT ACTGTCGCTC CAGGAGCAAA CTGATGTCGT GAGCTTAAAC
21361 TAGTTCTTGA TGCAGATGAC GTTTTAAGCA CAGAAGTTAA AAGAGTGATA ACTTCTTCAG
21421 CTTCAAATAT CACCCCAGCT TTTTTCTGCT CATGAAGGTT AGATGCCTGC TGCTTAAGTA
21481 ATTCCTCTTT ATCTGTAAAG GCTTTTTGAA GTGCATCACC TGACCGGGCA GATAGTTCAC
21541 CGGGGTGAGA AAAAAGAGCA ACAACTGATT TAGGCAATTT GGCGGTGTTG ATACAGCGGG
21601 TAATAATCTT ACGTGAAATA TTTTCCGCAT CAGCCAGCGC AGAAATATTT CCAGCAAATT
21661 CATTCTGCAA TCGGCTTGCA TAACGCTGAC CACGTTCATA AGCACTTGTT GGGCGATAAT
21721 CGTTACCCAA TCTGGATAAT GCAGCCATCT GCTCATCATC CAGCTCGCCA ACCAGAACAC
21781 GATAATCACT TTCGGTAAGT GCAGCAGCTT TACGACGGCG ACTCCCATCG GCAATTTCTA

21841 TGACACCAGA TACTCTTCGA CCGAACGCCG GTGTCTGTTG ACCAGTCAGT AGAAAAGAAG
21901 GGATGAGATC ATCCAGTGCG TCCTCAGTAA GCAGCTCCTG GTCACGTTCA TTACCTGACC
21961 ATACCCGAGA GGTCTTCTCA ACACTATCAC CCCGGAGCAC TTCAAGAGTA AACTTCACAT
22021 CCCGACCACA TACAGGCAAA GTAATGGCAT TACCGCGAGC CATTACTCCT ACGCGCGCAA
22081 TTAACGAATC CACCATCGGG GCAGCTGGTG TCGATAACGA AGTATCTTCA ACCGGTTGAG
22141 TATTGAGCGT ATGTTTTGGA ATAACAGGCG CACGCTTCAT TATCTAATCT CCCAGCGTGG
22201 TTTAATCAGA CGATCGAAAA TTTCATTGCA GACAGGTTCC CAAATAGAAA GAGCATTTCT
22261 CCAGGCACCA GTTGAAGAGC GTTGATCAAT GGCCTGTTCA AAAACAGTTC TCATCCGGAT
22321 CTGACCTTTA CCAACTTCAT CCGTTTCACG TACAACATTT TTTAGAACCA TGCTTCCCCA
22381 GGCATCCCGA ATTTGCTCCT CCATCCACGG GGACTGAGAG CCATTACTAT TGCTGTATTT
22441 GGTAAGCAAA ATACGTACAT CAGGCTCGAA CCCTTTAAGA TCAACGTTCT TGAGCAGATC
22501 ACGAAGCATA TCGAAAAACT GCAGTGCGGA GGTGTAGTCA AACAACTCAG CAGGCGTGGG
22561 AACAATCAGC ACATCAGCAG CACATACGAC ATTAATCGTG CCGATACCCA GGTTAGGCGC
22621 GCTGTCAATA ACTATGACAT CATAGTCATG AGCAACAGTT TCAATGGCCA GTCGGAGCAT
22681 CAGGTGTGGA TCGGTGGGCA GTTTACCTTC ATCAAATTTG CCCATTAACT CAGTTTCAAT
22741 ACGGTGCAGA GCCAGACAGG AAGGAATAAT GTCAAGCCCC GGCCAGCAAG TGGGCTTTAT
22801 TGCATAAGTG ACATCGTCCT TTTCCCCAAG ATAGAAAGGC AGGAGAGTGT CTTCTGCATG
22861 AATATGAAGA TCTGGTACCC ATCCGTGATA CATTGAGGCT GTTCCCTGGG GGTCGTTACC
22921 TTCCACGAGC AAAACACGTA GCCCCTTCAG AGCCAGATCC TGAGCAAGAT GAACAGAAAC
22981 TGAGGTTTTG TAAACGCCAC CTTTATGGGC AGCAACCCCG ATCACCGGTG GAAATACGTC
23041 TTCAGCACGT CGCAATCGCG TACCAAACAC ATCACGCATA TGATTAATTT GTTCAATTGT
23101 ATAACCAACA CGTTGCTCAA CCCGTCCTCG AATTTCCATA TCCGGGTGCG GTAGTCGCCC
23161 TGCTTTCTCG GCATCTCTGA TAGCCTGAGA AGAAACCCCA ACTAAATCCG CTGCTTCACC
23221 TATTCTCCAG CGCCGGGTTA TTTTCCTCGC TTCCGGGCTG TCATCATTAA ACTGTGCAAT
23281 GGCGATAGCC TTCGTCATTT CATGACCAGC GTTTATGCAC TGGTTAAGTG TTTCCATGAG
23341 TTTCATTCTG AACATCCTTT AATCATTGCT TTGCGTTTTT TTATTAAATC TTGCAATTTA
23401 CTGCAAAGCA ACAACAAAAT CGCAAAGTCA TCAAAAAACC GCAAAGTTGT TTAAAATAAG
23461 AGCAACACTA CAAAAGGAGA TAAGAAGAGC ACATACCTCA GTCACTTATT ATCACTAGCG
23521 CTCGCCGCAG CCGTGTAACC GAGCATAGCG AGCGAACTGG CGAGGAAGCA AAGAAGAACT
23581 GTTCTGTCAG ATAGCTCTTA CGCTCAGCGC AAGAAGAAAT ATCCACCGTG GGAAAAACTC
23641 CAGGTAGAGG TACACACGCG GATAGCCAAT TCAGAGTAAT AAACTGTGAT AATCAACCCT
23701 CATCAATGAT GACGAACTAA CCCCCGATAT CAGGTCACAT GACGAAGGGA AAGAGAAGGA
23761 AATCAACTGT GACAAACTGC CCTCAAATTT GGCTTCCTTA AAAATTACAG TTCAAAAAGT
23821 ATGAGAAAAT CCATGCAGGC TGAAGGAAAC AGCAAAACTG TGACAAATTA CCCTCAGTAG
23881 GTCAGAACAA ATGTGACGAA CCACCCTCAA ATCTGTGACA GATAACCCTC AGACTATCCT
23941 GTCGTCATGG AAGTGATATC GCGGAAGGAA AATACGATAT GAGTCGTCTG GCGGCCTTTC
24001 TTTTTCTCAA TGTATGAGAG GCGCATTGGA GTTCTGCTGT TGATCTCATT AACACAGACC
24061 TGCAGGAAGC GGCGGCGGAA GTCAGGCATA CGCTGGTAAC TTTGAGGCAG CTGGTAACGC
24121 TCTATGATCC AGTCGATTTT CAGAGAGACG ATGCCTGAGC CATCCGGCTT ACGATACTGA
24181 CACAGGGATT CGTATAAACG CATGGCATAC GGATTGGTGA TTTCTTTTGT TTCACTAAGC
24241 CGAAACTGCG TAAACCGGTT CTGTAACCCG ATAAAGAAGG GAATGAGATA TGGGTTGATA
24301 TGTACACTGT AAAGCCCTCT GGATGGACTG TGCGCACGTT TGATAAACCA AGGAAAAGAT
24361 TCATAGCCTT TTTCATCGCC GGCATCCTCT TCAGGGCGAT AAAAAACCAC TTCCTTCCCC
24421 GCGAAACTCT TCAATGCCTG CCGTATATCC TTACTGGCTT CCGCAGAGGT CAATCCGAAT
24481 ATTTCAGCAT ATTTAGCAAC ATGGATCTCG CAGATACCGT CATGTTCCTG TAGGGTGCCA
24541 TCAGATTTTC TGATCTGGTC AACGAACAGA TACAGCATAC GTTTTTGATC CCGGGAGAGA
24601 CTATATGCCG CCTCAGTGAG GTCGTTTGAC TGGACGATTC GCGGGCTATT TTTACGTTTC
24661 TTGTGATTGA TAACCGCTGT TTCCGCCATG ACAGATCCAT GTGAAGTGTG ACAAGTTTTT
24721 AGATTGTCAC ACTAAATAAA AAAGAGTCAA TAAGCAGGGA TAACTTTGTG AAAAAACAGC
24781 TTCTTCTGAG GGCAATTTGT CACAGGGTTA AGGGCAATTT GTCACAGACA GGACTGTCAT
24841 TTGAGGGTGA TTTGTCACAC TGAAAGGGCA ATTTGTCACA ACACCTTCTC TAGAACCAGC
24901 ATGGATAAAG GCCTACAAGG CGCTCTAAAA AAGAAGATCT AAAAACTATA AAAAAAATAA
24961 TTATAAAAAT ATCCCCGTGG ATAAGTGGAT AACCCCAAGG GAAGTTTTTT CAGGCATCGT
25021 GTGTAAGCAG AATATATAAG TGCTGTTCCC TGGTGCTTCC TCGCTCACTC GAGGGCTTCG
25081 CCCTGTCGCT CAACTGCGGC GAGCACTACT GGCTGTAAAA GGACAGACCA CATCATGGTT
25141 CTGTGTTCAT TAGGTTGTTC TGTCCATTGC TGACATAATC CGCTCCACTT CAACGTAACA
25201 CCGCACGAAG ATTTCTATTG TTCCTGAAGG CATATTCAAA TCGTTTTCGT TACCGCTTGC
25261 AGGCATCATG ACAGAACACT ACTTCCTATA AACGCTACAC AGGCTCCTGA GATTAATAAT
25321 GCGGATCTCT ACGATAATGG GAGATTTTCC CGACTGTTTC GTTCGCTTCT CAGTGGATAA
25381 CAGCCAGCTT CTCTGTTTAA CAGACAAAAA CAGCATATCC ACTCAGTTCC ACATTTCCAT
25441 ATAAAGGCCA AGGCATTTAT TCTCAGGATA ATTGTTTCAG CATCGCAACC GCATCAGACT

25501 CCGGCATCGC AAACTGCACC CGGTGCCGGG CAGCCACATC CAGCGCAAAA ACCTTCGTGT
25561 AGACTTCCGT TGAACTGATG GACTTATGTC CCATCAGGCT TTGCAGAACT TTCAGCGGTA
25621 TACCGGCATA CAGCATGTGC ATCGCATAGG AATGGCGGAA CGTATGTGGT GTGACCGGAA
25681 CAGAGAACGT CACACCGTCA GCAGCAGCGG CGGCAACCGC CTCCCCAATC CAGGTCCTGA
25741 CCGTTCTGTC CGTCACTTCC CAGATCCGCG CTTTCTCTGT CCTTCCTGTG CGACGGTTAC
25801 GCCGCTCCAT GAGCTTATCG CGAATAAATA CCTGTGACGG AAGATCACTT CGCAGAATAA
25861 ATAAATCCTG GTGTCCCTGT TGATACCGGG AAGCCCTGGG CCAACTTTTG GCGAAAATGA
25921 GACGTTGATC GGCACGTAAG AGGTTCCAAC TTTCACCATA ATGAAATAAG ATCACTACCG
25981 GGCGTATTTT TTGAGTTATC GAGATTTTCA GGAGCTAAGG AAGCTAAAAT GGAGAAAAAA
26041 ATCACTGGAT ATACCACCGT TGATATATCC CAATGGCATC GTAAAGAACA TTTTGAGGCA
26101 TTTCAGTCAG TTGCTCAATG TACCTATAAC CAGACCGTTC AGCTGGATAT TACGGCCTTT
26161 TTAAAGACCG TAAAGAAAAA TAAGCACAAG TTTTATCCGG CCTTTATTCA CATTCTTGCC
26221 CGCCTGATGA ATGCTCATCC GGAGTTCCGT ATGGCAATGA AAGACGGTGA GCTGGTGATA
26281 TGGGATAGTG TTCACCCTTG TTACACCGTT TTCCATGAGC AAACTGAAAC GTTTTCATCG
26341 CTCTGGAGTG AATACCACGA CGATTTCCGG CAGTTTCTAC ACATATATTC GCAAGATGTG
26401 GCGTGTTACG GTGAAAACCT GGCCTATTTC CCTAAAGGGT TTATTGAGAA TATGTTTTTC
26461 GTCTCAGCCA ATCCCTGGGT GAGTTTCACC AGTTTTGATT TAAACGTGGC CAATATGGAC
26521 AACTTCTTCG CCCCCGTTTT CACCATGGGC AAATATTATA CGCAAGGCGA CAAGGTGCTG
26581 ATGCCGCTGG CGATTCAGGT TCATCATGCC GTTTGTGATG GCTTCCATGT CGGCAGAATG
26641 CTTAATGAAT TACAACAGTA CTGCGATGAG TGGCAGGGCG GGGCGTAATT TTTTTAAGGC
26701 AGTTATTGGT GCCCTTAAAC GCCTGGTTGC TACGCCTGAA TAAGTGATAA TAAGCGGATG
26761 AATGGCAGAA ATTCGATGAT AAGCTGTCAA ACATGAGAAT TGGTCGACGG CGCGCCAAAG
26821 CTTGCATGCC TGCAGCCGCG TAACCTGGCA AAATCGGTTA CGGTTGAGTA ATAAATGGAT
26881 GCCCTGCGTA AGCGGGGCAC ATTTCATTAC CTCTTTCTCC GCACCCGACA TAGATAATAA
26941 CTTCGTATAG TATACATTAT ACGAAGTTAT CTAGTAGACT TAATTAAGGA TCGATCCGGC
27001 GCGCCAATAG TCATGCCCCG CGCCCACCGG AAGGAGCTGA CTGGGTTGAA GGCTCTCAAG
27061 GGCATCGGTC GAGCTTGACA TTGTAGGACT ATATTGCTCT AATAAATTTG CGGCCGCTAA
27121 TACGACTCAC TATAGGGAGA GGATCCTCCA GTTGATCCTA CCACAGATGG TGCAGAAGGG
27181 GCTCTCGGGC AGATGGGGGT GGCCCCCGGA GGACGGTGGG CTTCGCCAGA GACGGAACAG
27241 TCCTAATGGA GGGAGGGCAG GGGCCCAAAG CCAGAGGCAG CTTCTCTTAC CAGCCCAGAC
27301 CCTGCTGGGG GCAGCCCTGC CCAACTGCAG ATCCCAGGGG CCCACAGGGT GCAGATGTGG
27361 GTCGGGTGCC TACTAAGGGA CTGGGGAGAG GCAAAAGCAG TGCTCAGGGA CCAGCCTCTG
27421 TGCCTTGACA AGAACCGGTT TATTTGGAGT CTCTGACCGG GTGACGCCCT CTCCCAAGGA
27481 CAGGCTGCTG GGGATGGGTG TGGTGCTGGT GGGGGCCTTC TGCCTGGCTG GGAGCGGGGG
27541 CGGCTGCCCT GCCCTCGCTG CCACCCTCTG CCCTCCCTGA CTCTGGGGAT CTCCTCTTCT
27601 CTGTGCTGGT GGCTGCCTGG GCCTCTTGCC CCCATCTCGG CACAGGTTTC CGTGCCCTCC
27661 TCGCCCCTGA TGGGTCTGGT CGAGCGCCTG CTCTGTGGCT TCATCTGCAG GGTTCTGGGC
27721 CCAGCAGGGC TGGGGCCAAG TTCAGGGGCT GGGAGGTGTT GGACGGTGGG CAGGGGTGGT
27781 CGGGAGTGCC CTGTGTGCCT GGGAGGTCCG TGACCACTGT CCGCCTCAGT CCCTCTCTGC
27841 TGGCTCAGGG TCTGCAGGAG TGTGGTAGTC TGAGCCCCTG CTTGGCAGCC CCTCTCCAAC
27901 CGGTGCCCCA GGGAGCCACG GCCCAGGGCA CTTGGGGGCC AGGCCTGGTG ACCAGGAAAG
27961 CAGCTGCTGG CACAAGCGGG AAGGGGGCTG GTGGGTGTTT TCCTGTCTGT CATCTGCAGT
28021 CCTTCAGCCC CAGGAGAGCA GGCAGCGACC CTGCTAGTCT CCACAGGCCA CAGAGCTGCA
28081 CACGCAGTGG CTGAACACCT GCAGGGTGAG TACGAGCCGC CGGCCAGGCG TGCTGGGATC
28141 GGGGCAGGGC AGCTCCAGCT GCTGCTCATA GGAGTGGAGG GGGCGGCAGC AGCTGCAGCG
28201 GGCATCCACC TGCTGGGTGA TGATGTTGAA GCTGCCGAGA AGTCAAGACA GAGCAGGGTC
28261 ATGACTGCTG CAGGGGCATA AGGCCCCTCC CTCCCCAGGG CAGCTGCTCC GCAGAGGCCT
28321 GGACCTCCCC GCTGAGCTCC CGGCTCACAG GGGCCAGGGC GGTGTTGGGC AGATGGAGCG
28381 CAGAGTGGCT CACCTGATGG GGCAGCAGGC GCCTGGGTGG GGTGCCCGAA CCTCTCTGGG
28441 GTGGGGTTGT GAGCACCTTG GTGGACGTGG GGCAGGCCTC CCACCTGTGG ACTCACCTGG
28501 CAGCGGAAAT GCAGGCGCCC TCACAGCGGG TTACCGTCAC GTTCGCCATG CACCCCTTGA
28561 ACGTGATCTC CTCCTGCTGC TCCCGCACAC TGCAGACCCC TGGTAGCCGA GTGGACGGTC
28621 AGCAGCGCCC AGGGTGGGCA TGGAGCGAGG AGGGAGGGAA ACCCTGGCTA GAGACCGGGG
28681 TCCCCACCTG TCTCTGGACT CTCCTGCCCA AGGTGTGGTC TCCCCTTGTG GAGCCCCACA
28741 GAGCTCAGAC CTCAGCCATA CACAAAGGCA AAGGCCAGCC GCATCCTAGT TTGTTTTTTC
28801 CCCTCAAAGT CGCTCTGCTG GAGTCCATAA GGTAGAGAAT TTTCTCAGCG GACTCAGCAG
28861 ATACTGAGCC CTGGGCCTGT GTGAGCCACC CTTGGGCCCC AGCTTCCCTG ATGACCAACC
28921 CCAGGCCTCA ACACTGACCT GTGGGCATGG GCCTCTCTGG TTGGCCAGAC CCAGGACCTG
28981 CAGGGGGGCC GAGGACACCG TGCCAGTGAC CCCAGAGCGG GACATATGGT GTTGCAGGAG
29041 CTCCGTGTGC CCCAGCTGGG GAGGAGGGAA GTGGAGCAGG CCCCGTGGTC TGGGCTCCTG
29101 GAAGGGGCGG GGTGGGGGCC CGCGGGGAAG GCCCAGAGAC AGCCAGCCTC AGCGAGAGGC

29161 CTGGGTACGT GGCAGGCAGC ACAGAGCAGG GTGGACCGAT GCAGGCAGTG GCAGCGTTGG
29221 CATTAGAAAC CGCCACTGGA GAGCGTGGGT TTAAAAGTGG AAAAGGAGTC CTGGGGTAAA
29281 GGCTGGACTC TGCTGCCCGT GTGCCCTCCC CTGGCTGCTC CCAGCTTCGT TTCCTGGCTG
29341 CAGAGAAGAC AGGTCACCTG GAGCCTACCT GCCCGCTGCA GAGGGCTTTC TCCGGGAGGG
29401 TCAGGACTCT GGGTGCTTCC ATAGGCCCTG CCGTCCCCAG CTGCCCTCCC CTCCCAGCTG
29461 GTTCCTGCTC CCTCGCCTCT GCTTCTTGGG GTCACCTCCC TGGTTACACC TTGCACCCAA
29521 GTCCCGGTCT CAGGGAGGGA CCCGAACCAA GATTCCCTCA GCTTGCAGCA ACGAGCTGCC
29581 ACCCACGTGA GAGACCACGA AGGCAACTGT GGCGTTGTCG CCTGGTGGAG GTCGATGCAT
29641 GCGAGGGACC CAGTCCCTGG GTGGTTGCTG TGAGAGGAAC ACCCAGGAGG GAAGGGAGGG
29701 ACATCCCTGG AACGCGAGGT GAGGTGAGGT GGACATGGAT AAGGCCTGAG GTGTGCTCTG
29761 TCCCCAGGCA TCCCATGGCC TGGGGAGCAG TGGACTCGCT CACAGGCATG GGCCGGGAAG
29821 AGCCGTGGCC CAGAGAATTG GCATGAGCAC GAAGGAGCAG GTGCCCTGGT TGCCAGGTCC
29881 AGCTCCACCT TCAACAGGTC CTTTGGGGCG AGGCAGGGGA TGTGCCAGAC TTTTGTGTCT
29941 CTCTCTGCAC TTGGAGGAAG GGCTGTGCTG CTTGAACCCT GGGGTTGGGC CAGCCCTGAC
30001 ATCCTCTGAG CAACCAAAGG GGCAGCCAGG GAAGGCTTTT GGCAGGTAAC ACTCTGAGGG
30061 GAGGCCAGGA GTGCAGCAGT GACCGGGCAT GAGTGAGCCA GTGCAGGGAA AGGTCAAGGT
30121 TAGCTGGGGA ACAAGGCCCA ACGGGGAGGC CAGGCACTTG CTTGGCCAGG ACAGCGTTGG
30181 CATAGAGGGT GCCCAGGAGA GGAGAGTGGA AACGCCTGGC AGGTGTGTAG GGAAGGCAGG
30241 GAACAGGCGT GTGGTCCTGG GATCACAGGA CCATGAGGGG TAAGCTGAGG CAGGGGCTGC
30301 CTGGGAGTCA TGAGCACAGA GGTGAGGCCA GGAGAAGGGC CACAGAGATG CCACTTACCG
30361 GGTGAGGTGG GCGTAGGTGT CCCGAGAGAA GATACCGGGG CAGAAGCAGG GGTGCTTCCA
30421 TGGGCAGTGA GGGAGCTGGT CAGGAACCGT GTGGTAGGCG ACAAGGTGGG ACCAGGGTGC
30481 CTGGTGGTAA GGTTGGTGAC TGGAGAGGTG GGGATACCCG TCACCCCCGA GGTGAGTGAC
30541 ACAAAGCCTG ATGTGGGAAC TCGGGTGGTG AGAGAAGTGG ACCGCGAGGT GGTGGACTGA
30601 GAGGAGAAGG CAGGGGCGGT GTGGGTGCTG GCCGTGGTCC TGGGCGTGGA CGGAAATGCA
30661 ATGGTGCTGG AGGCTAGGTG GCTGGATGGG GTGGGAGACA CGGTAACAGT GGATATGGGG
30721 AGTAGAGCAG AGAGGGTGAA AGGAGAGGAG ATAGTGTGGG GGAGAGTGGC CCTAATGGTA
30781 GTAGAGGCAG CTGGAGAAGA AGGAAAAAGA GGAGATGCAG ACACTGATGC AGTCGTGGGA
30841 TGAGTGGACA ATGAGGAGTG TGACCCCGAG CTCAGGGTTG TGGAGTGCAC GGGGGCGGAC
30901 ACGAAAGAGG AAGATGTGCC AACAGAAGGC GATGAAGTCT GGGGAGAGGA GTGGGAGGAG
30961 GGCACATAAG AAGAAACAGT AGAGGGGGCA GAAGGACTGG GAGAAAATGA GGAGGACAGC
31021 TGATTAGTTG TGGAAACAGG AGTGGTTGCA GAACTCAAGT GGGGGAGTTG TGTGGTGATA
31081 GGTGATGACG GTGGCCTTGA GCTAGAGTTC TGAGGCAGCC AAGACGAGGA GGATATGAAG
31141 GAAGAAGAGG CTGTAGCTGT GCTGAATGAG CTGTGGGTTT GGCTGGTCCC ACTGGTGGTC
31201 ACTGTCATTG GTGGGGCTGT GTGGGTGGAC CCTGTGGCCT TGAGCGTTGT TGGTGGAGGA
31261 ACGGTGCCTG TTGGCGTTGA GTGGATGGAG GCAGAAGTGG CCATCTGTGT GTGGGTAGTG
31321 ATGATGACTG TGTGAGTACT TGGAGTCACC AAGGAGGTGG AGAAAGGTGG AACGTGAGTG
31381 GGAAGTGTGG TCTGAGGGTG TGATGGGGTT GGATAGGTAG TGGTGGTCTG GAAGGATGTT
31441 GCAGTCATAG GACCTGTGGA AGAGAAGGGA CTGCTCCCTG TAGGTGGGGA GTGTGTGGTG
31501 AAGGGTGTGG GTAGCCTGCT GCTGCTGGCC GAGGTGGTGT GGGCCACAGG GGTTCTGGTG
31561 CCTGTACTGG TGTGTTTGGG GGTGATGTTG GTGGTAGAAG TTGGGGTGAC TTCAGGATGG
31621 TGTGTTGAGG AAGTGTGGTA AGGTAGGGAT GTAGAAGTTT TGGCCGTGCT AAATGAGCTT
31681 GGGGATTGGC TGGTCCCACT GGTGGTCGGT GTCATTGGTG GGGCTGTGTG GGTGGACCCT
31741 GTGGCCTTGA TCGTGGTCGG TGGAGGAATG GTGCCTGTTG GCATTGAGTG GATGGAGGCA
31801 GAAGTGGCCA TCTGTGTGTG GGTAGGGATG ATGACCGTGT GAGTACTTGG AGTCACCAAG
31861 GAGGTGGAGA AAGATGGAAC GTGAGTGGGA AGTGTGGTGT GAGGGTGTGA TGGGGTTGGA
31921 TAGGTAGTGG TGGTCTGGAA GGATGTTGCA GTGACAGGAC CTGTGGAAGA GATGGGAGTG
31981 GTCCCTGTAG GTGGGGAGTG TGTGGTGAAG GGTGTGGGTA GCCTGCTGCT GGTGGCCGAC
32041 GTGGTGTGGG CCACAGGGGT TCTGGTGCCT GTACTGGTGT GGTTGGGGGT GATGCTGGTG
32101 GTAGAAGTTG AGGTGACTTC AGGATGGTGT GTGGAGGAAG TGTGTGAATG TAGGGATGTA
32161 GAGGTTTTGG CCGTGCTAAA TGAGCTTCGG GATTGGCTGG TCCCACTGGT GGTCACTGTC
32221 ATTGGTGGGG TTCCTGTACT GGTGGGGTTG GGGGTGATGT TGGTGGTAGA AGTTGAGGTG
32281 GCTTCAGCAT GGTGTGTGGA GGAAGTGTGT GAATGTAGGG ATGTAGAGGT TTTGGCTGTG
32341 TTTAATGAGC TCAGGGCTTG GCTGGTCCCG CTGGTGGTCA GCGTCATTGT TGGCGCTGTG
32401 TGGGTGGACC CTGTGGCCTT GAGCGTTGTC GGTGGAGGAA TCGTGCCTGT TGGCATTGAG
32461 TGGATGGAGG CAGAAGTGGC CATCTGTGCA TGGGTAGGGG TGATGACTGT GTGAGTACTT
32521 GGAGTCACCA AAGAGGTGGA GAAAGGTGGA ACGTGAGTGG GAAGTGTGGT CTGAGGGTGT
32581 GATGGGGTTG GATAGGTAGT GGTGGTCTGG AAGGATGTTG CAGTCATAGG ACCTGTGGAA
32641 GAGAAGGGAC TGCTCCCTGT AGGTGAGGAG TGTGTGGTGA AGGGTGTGGT TAGCCTGCTG
32701 CTGGTGGCTG AGGTGGTGTG GGCCACAGGG GTGCCGGTTC CTGGACTGGT GGGATTGGGG
32761 GTGATGGTGG TAGAAGTTGG GGTGACTTCA GGAAGGTGTG TGGAGGAAGT TTGTGAATGT

32821 AGGGATGTAG AGGTTTTGGC TGTGTTGAAT GAGCTCAGGG CTTGGCTCGT CCCGCTGGTG
32881 GTCGGCGTCA TTGTTGGCGC TGTGTGGGTG GACCCTGTGG CCTTGAGCGT TGTTGGTGGA
32941 GGAACGGTGC CTGTTGGCGT TGAGTGGATG GAGGCAGAAG TGGCCATCTG TGTGTGGGTA
33001 GTGATGATGA CTGTGTGAGT ACTTGGAGTC ACCAAGGAGG TGGAGAAAGG TGGAAGGTGA
33061 GTGGGAAGTG TGGTCTGAGG GTGTGATGGG GTTGGATAGG TAGTGGTGGT CTGAAAGGAT
33121 GTTGCAGTCA TAGGACCTGT GGAAGAGATG GGACTGCTCC CTGTAGGTGG GGAATGTGTG
33181 GTGAAGGGTA TGGGTAGCCT GCTGCTGGTG GCCGAGGTGG TGTGGGCCAC AGGGGTTCTG
33241 GTGCCTGTAC TGGTGTGGTT GGGGGTGATG GTGGTGGTAG AAGTTGGGGT GACTTCAGGA
33301 TGATGTGTTG AGGAAATGTG TGAATGTAGG GATGTAGAAG TTTTGGCCGT GCTAAATGAG
33361 CTTGGGGATT GGCTGGTCCC ACTGGTGGTT GCCGTCATTG GTGGGGCTGT GTGGGTGGAC
33421 CCTGTGGCCT TGATCGTGGT CGGTGGAGGA ATAGTGCCTG TTGGCATTGA GTGGATGGAG
33481 GCAGAAGTGG CCATCTGTGC GTGGGTAGGG GTGATGACTA TGTGAGTACT TGGAGTCACC
33541 AAGGAGGTGG AGAAAGATGG AACGTGAGTG GGAAGTGTGG TGTGAGGGTG TGATGGGGTT
33601 GGATCGGTAG TGGTGGTCTG GAAGGATGTT GCAGTGACAG GACCTGTGGA AGAGATGGGA
33661 GTGGTCCCTG TAGGTGGGGA GTGTGTGGTG AAGGGTGTGG GTAGCCTGCT GCTGGTGGCC
33721 GACGTGGTGT GGGCCACAGG GGTTCTGGTG CCTGTACTGG TGTGGTTGGG GGTGATGCTG
33781 GTGGTAGAAG TTGAGGTGAC TTCAGGATGG TGTGTGGAGG AAGTGTGTGA ATGTAGGGAT
33841 GTAGAGGTTT TGGCCGTGCT AAATGAGCTT CGGGATTGGC TGGTCCCACT GGTGGTCACT
33901 GTCATTGGTG GGGTTCCTGT ACTGGTGGGG TTGGGGGTGA TGTTGGTGGT AGAAGTTGAG
33961 GTGGCTTCAG CATGGTGTGT GGAGGAAGTG TGTGAATGTA GGGATGTAGA GGTTTTGGCT
34021 GTGTTTAATG AGCTCAGGGC TTGGCTGGTC CCGCTGGTGG TCGGCGTCAT TGTTGGCGCT
34081 GTGTGGGTGG ACCCTGTGGC CTTGAGCGTT GTCGGTGGAG GAATCGTGCC TGTTGGCATT
34141 GAGTGGATGG AGGCAGAAGT GGCCATCTGT GCATGGGTAG GGGTGATGAC TGTGTGAGTA
34201 CTTGGAGTCA CCAAAGAGGT GGAGAAAGGT GGAACGTGAG TGGGAAGTGT GGTCTGAGGG
34261 TGTGATGGGG TTGGATAGGT AGTGGTGGTC TGGAAGGATG TTGCAGTCAT AGGACCTGTG
34321 GAAGAGAAGG GACTGCTCCC TGTAGGTGAG GAGTGTGTGG TGAAGGGTGT GGTTAGCCTG
34381 CTGCTGGTGG CTGAGGTGGT GTGGGCCACA GGGGTGCCGG TTCCTGTACT GGTGGGATTG
34441 GGGGTGATGG CGGTAGAAGT TGGGGTGACT TCAGGAAGGT GTGTGGAGGA AGTTTGTGAA
34501 TGTAGGGATG TAGAGGTTTT GGCTGTGTTG AATGAGCTCA GGGCTTGGCT CGTCCCGCTG
34561 GTGGTCGGCG TCATTGTTGG CGCTGTGTGG GTGGACCCTG TGGCCTTGAG CGTTGTTGGT
34621 GGAGGAACGG TGCCTGTTGG CGTTGAGTGG ATGGAGGCAG AAGTGGCCAT CTGTGTGTGG
34681 GTAGTGATGA TGACTGTGTG AGTACTTGGA GTCACCAAGG AGGTGGAGAA AGGTGGAAGG
34741 TGAGTGGGAA GTGTGGTCTG AGGGTGTGAT GGGGTTGGAT AGGTAGTGGT GGTCTGAAAG
34801 GATGTTGCAG TCATAGGACC TGTGGAAGAG ATGGGACTGC TCCCTGTAGG TGGGGAATGT
34861 GTGGTGAAGG GTATGGGTAG CCTGCTGCTG GTGGCCGAGG TGGTGTGGGC CACAGGGGTT
34921 CTGGTGCCTG TACTGGTGTG GTTGGGGGTG ATGGTGGTGG TAGAAGTTGG GGTGACTTCA
34981 GGATAGTGTG TGGAGGAAGT GTGTGAATGT AAGGATGTAG AGGTTTTGCC TGTGCTAAAT
35041 GAGCTTGCGG ATGGGCTGGT CCCACTGGTG GTCACTGTGA TTGGTGGGGC TGTGTGGGTG
35101 GACCCTGTGA CCTTGAGCGT TGTTAGTGGA GGAATGGTGC CTGTTGGCGT TGAGTGGATG
35161 GAGGCAGAAG TGGACATCTG TGCATGGGTA GGGGTGATGA CCTTGTGAGT ACTTGGAGTC
35221 ACCAAGGAGG TGGAGAAAGG TGGAATGTGA GTGGGAAGTG TGGTCTGAGG GTGTGATGGG
35281 GTTGGATAGG TAGTGGTGGT CTGAAAGGAT GGTGCAGTCA TAGGACCTGT GGAAGAGATG
35341 GGACTGCTCC CTGTAGGTGG GGAATGTGTG GTGAAGGGTG TGGGTAGTCT GCTGCTGGTG
35401 GCCGAGGTGG TGTGGGCCAC AGGGGTTCTG GTGCCTGTAC TGGTGTGTTT GGGGGTGATG
35461 TTGGTGGTAG AAGTTGGGGT GACTTCAGGA TGGTGTGTGG AGGAAGTGTG GTAAGGTAGG
35521 GATGTAGAAG TTTTGGCCGT GCTAAATGAG CTTAGGGATT GGCTGGTCCC ACTGGTGGTC
35581 GGTGTCATTG GTGGGGCTGT GTGGGTGGAC CCTGTGGCCT TGATCGTGGT CGGTGGAGGA
35641 ATGGTGCCTG TTTGCATTGA GTGGATGGAG GCAGAAGTGG CCATCTGTGC GTGGGTAGGG
35701 GTGATGACTA TGTGAGTACT TGGAGTCACC AAGGAGGTGG AGAAAGATGG AACGTGAGTG
35761 GGAAGTGTGG TCTGAGGGTG TGATGGTGTT GGATAGGTAG TGGTGGCATG GAAGGATGTT
35821 GCAGTGACAG GACCTGTGGA AGAGATGGGA GTGGTCCCTG TAGGTGGGGA GTGTGTGGTG
35881 AAGGGTGTGG TTAGCCTGCT GCTGGTGGCT GAGGTGGTGT GGGCCACGGG TGTCCAGGTT
35941 CCTATACTGG TGGGGTTGAG GGTGATGTTG GTGATAGAAG TTGGGGTGAC TTCAGGATGG
36001 TGTGTGGAGG AAGTGTGTGA ATGTAGGGAT GTAGAGGTTT TGGCTGTGTT GAATGAGCTT
36061 GAGGCTTGGC TGGTCCCACT GGTGGTTGGC ATCATTGGTG GGGCTGTGTG GGTGGACCCT
36121 GTGGCCTTCA GCGTTGTCGG TGGAGGAATG GTGCCTGTTG GCATTGAGTG GATGGAGGCA
36181 GAAGTGGTCA TCTGTGCGTG GGTAGGGGTG ATGACTGTGT GAGTACTTGG AGTCACCAAG
36241 GAGGTGGAGA AAGATGGAAC GTGAGTGGGA AGTGTGGTCT GAGGGTGTGA TGGTGTTGGA
36301 TAGGTAGTGG TGGCATGGAA GGATGTTGCA GTGACAGGAC CTGTGGAAGA GATGGGACTG
36361 CTCCCTGTAG GTGGGGAGTG TGTGGTGAAG GGTGTGGTTA GCCTGCTGCT GGTGGCTGAG
36421 GTGGTGTGGG CCACAGGGGT GACGGTTTCT GTACTGGTGG GATTGGGGGT GATGGTGGTA

36481 GAAGTTGGGG TGACTTCAGG ATGGTGTGCA GAGGAAGTGT GTGAATGTAA GGATGTAGAG
36541 GTTTTGGCTG TGCTGAATGA GCTCGGGGCT TGGCTGGTCC CACTGGTGGT CGGCGTCATT
36601 GGTGGGGCTG TGTGGGTAGA CCCTGTGGCC TTGAGCGTTG TTGGTGGAGG AATGGTGCCT
36661 GTTGGCGTTG AGTGGATGGA GGCAGAAGTG GACATCTGTG CGTGGGTAGT GGTGATGACT
36721 GTGTGAGTAC TTGGAGTCAC CAAGGAGGTG GAGAAAGGTG GAATGTGAGT GGGAAGTGTG
36781 GTGTGAGGGT GTGATGGGGT TGGATAGGTA GTAGTGGTCT GGAAGGATGT TGCAGTCATA
36841 GGACCTGTGG AAGAGAAGGG ACTGCTCCCT GTAGGTAAGG AGTGTGTGGT GAAGGGTGTG
36901 GGTAGCCTGC TGCTGGTGGC TGACGTGGTG TCGGCCACAG GGGTGCCGGT TTCTGTACTG
36961 GTGGGTTTGG GGGTGATGGT GGTAGAAGTT GGGGTGACTT CAGGATGGTG TGTGGAGGAA
37021 GTGTGTGAAT GTAGGGATGT AGAGGTTTTG GCTGTGCTGA ATGAGCTCAG GGCTTGGCTG
37081 GTCCCACTGG TGGTCGGCGT CATTGGTGGG GCTGTGTGGG TGGACCCTGT GGCCTTGAGC
37141 GTTGTCAGTG GAGGAATGGT GCCTGTTGGC GTTGAGTGGA TGGAGGCAGA AGTGGCCATC
37201 TGTTGATGGG TTGGGGTGAT GACTGTGTGT GTACTTGGAG TCACCAAGGA GGTGGAGAAA
37261 GGTGGAATGT GAGTGGGAAG TGTGGTCTGA GACTGTGATG TGGTTGGATA GGTAGTGGTG
37321 GTCTGGAAGG ATGTTGCAGT GACAGGAGCT GTGGAAGAGA TGGGACTGCT CCCTGTCGGT
37381 GGGGAGTGTG TGGTGAAGGG TGTGGGTAGC CTGCTGCTGG TGGCCGAAGT GGTGTGGGCC
37441 ACAGGGGTCC TGGTGCCTGT ACTGGTGTGG TTGGGGGTGA TGTTGGTGGT AGAAGTTGGG
37501 GTGACTGCAG GATGGTGTGT GGAGGAAGTG TGTGAATGTA GGGATGTAGA AGTTTTGGCT
37561 GTGCTGAATG AGCTTGGGGC TTGCCTGGTC CCACTGGTGG TCGGCGTCAT TGGTGGGGCT
37621 GTGTGGGTGG ACCCTGTGGC CTTGAGCGTT GTCGGTGGAG GAATGGTGCC TGTTGGCGTT
37681 GAGTGGATGG AGGCAGAAGT GGACATCTGT GCATGGGTAT GGGTGATGAC TGTGTGAGTA
37741 TTTGGAGTCA CCAAGGAGGT GGAGGAAGGT GGAACGTGAG TGGGAAGTGT GGTGTGAGAG
37801 TGTGATGGGG TTGGATAGGT AGTGGTGGTC TGGAAGGATG TTGCAGTGAC AGGACCTGTG
37861 GAAGAGATGG GACTGCTTCC TGTAGGTGGG GAGTGTGTGG TGAAGGGTGT GGGTAGCTTG
37921 CTGCTGGTGG CCGAGGTGGT GTGGGCCACA GGGGTTCTTG TGCCTGTACT GGTGTGTTTG
37981 GGGGTGATGT TGGTGGTAGA AGTTGGGGTG ACTTCAGGAT GGTGTGTGGA GGAAGTGTGG
38041 TAAGGTAGGG ATGTAGAAGT TTTGGCCATG CTAAATGAGC TTGGGGATTG GCTGGTCCCA
38101 CTGGTGGTCG GCGTCATTGG TGGGGCTGTG TGGATGGACC CTGTGGCCTT GAGCGTTGTT
38161 GGTGGAGAAA TGGTGCCTGT TGGCGTTGAG TGGATGGACG CAGAAGTGGC CATCTGTGCG
38221 TGGGTAGGGG TGATGACTGT GTGAGTACTT GGAGTCACCA AGGAGGTGGA GAAAGATGTA
38281 ACGTGAGTGG GATGTGTGGT CTGAGGGTGT GATGGTGTTG GATAGGTAGT GGTGGCATGG
38341 AAGGATGTTG CAGTGACAGG ACCTGTGGAA GAGATGGGAG TGCTCCCTGT AGGTGGGGAG
38401 TGTGTGGTGA AGGGTGTGGT TAGCCTGCTG CTGGTGGCTG AGGTGGTGTG GGCCACAGGT
38461 GTCCCAGTTC CTGTACTGGT GGGGTTGGGG GTGATGTTGG TGGTAGAAGT TGAGGTGGCT
38521 TCAGGATGGT GTGTGGAGGA AGTGTGTGAA TGTAGGGATG TAGAGGTTTT GGCTGTGTTT
38581 AATGAGCTCA GGGCTTGGCT GGTCCCGCTG GTGGTCAGCG TCCTTGTTGG CGCTGTGTGG
38641 GTGGACCCTG TGGCCTTGAG CGTTGTCGGT GGAGGAATGG TGCCTGTTGG CGTTGAGTGG
38701 ATGGAGGCAG AAGTGGCCAT CTGTGTGCGG GGAGGGGTGA TGACTGTGTG AGTACTTGGA
38761 GTCACCAAGG AGGTGGAGAA AGGTGGAACG TAAGTGGGAA GAGTGGTCTG AGAGAGTGAT
38821 GGTGTTGGAT AGGTAGTGGT GGTCTGGAAG GATGTTGCAG TCATAGGACC TGTGGAAGAG
38881 ACAGGACTGC TCCCTGTAGA TGGGGAGTGT GTGGTGAATG GTGTAGTTAG CCTGCTGCTG
38941 GTGGCTGAGG TAGTGTGGGC CATGGGTGTG CTGCTTCCTA TACTGGTGGG GTTGGTGGTG
39001 ATTTTGGTGG TAGCAGTTGG GGTGACTTCA GGATGGTGTG TGGAGGAAGT GTGTGAATGT
39061 AAGGATGTAG AGGTTTTGGC TGTGCTGAAT GAGCTCGCGG CTTGGCTCGT CCCACTGGTG
39121 GTCGGCGTCA CTGGTGGGGC TGTGTGTGTG GACCCTGTGT TCATGAGCGT TGTCAGTGGA
39181 GGAATGGTGC CTGTTGGCGT TGAATGGATG GAGGCGGAAG TGGCCATCTG TGCGTGGGTA
39241 GGGGTGATGA CTGTGTGAGT ACTTGGAGTC ACCAAGGAGG TGGAGAAAGG TGGAACGTGA
39301 GTGGGAAGTG TGGTCTGAGG GTGTGATGGG GTTGGATATG TAGTGGTGGT CTGGAAGGAT
39361 GTTGCAGTCA TAGGACCTGT GGAAGAGATG GGACTGCTCC CTGTAGGTGG GGAGTGTGTG
39421 GTGAAGGGTG TGGTTAGCCT GCTGCTGGTG GCTGAGGTGG TGTTTGCCAC AGGCGTTCTG
39481 ATGCCTGTAT TGGTGGGGTT GGGGGTGATG GTGGTGGTAG AAGTTGGGGT GACTTCAGGA
39541 TGGAGTGTTG AAGAAGCATG TGAGTGTAGG GATGTGGAGG TTTTGGCTGT GCTGAAAGAG
39601 CTGTGCGCTT GGCTGGTCCC GCTGGTGGTC ACTGTCATTC GTGGTGCTGT GTGCGTGGAC
39661 CCTGTGCCTG TGGCCTTTAC CGTTGTAGGT GGAGGAATGG TGCCTGTTGG TGTTGAGTGG
39721 ATGGAGGCAG AAGTGGACAC TTGTGCGTGG GTAGGGGTGA TGACTGTGTG AGTACTTGGA
39781 GTCACCAAGG AGGTGGAGAA AGGTGGAATG TGAGTGGGAA GTGTGGTCTG AGGGTGTGAT
39841 GGGGTTGGAT GGGTAGTGGT GGTCTGGGAG GATGTTGCAG TCATAGGACC TGTGGAAGAG
39901 ACATGACTGC TCCCTGTAGG TGGGAAGTGT GTGGTGAAGG TTGTGGGTAG CCTGCTGCTG
39961 GTGGCCAAGG TGGTGTGGGC CACAGGGGTG CTGGTTCCTG CACTAGTGGA CTTGGGAGTA
40021 ATGTTGGTGG TAGAAGTTGG TGTGGTTTCA GGATGGTGTG TGGAGGAAGC ATGTGAGTGG
40081 AGAGATGTAG AAGTTTTGGC TGTGCTGAAT GAGTTGTGAG CTTTGCTTGT CTGAATAATG

40141 GTCCCCGTCA TTGGTGGGCC TGTGTGTGTC GACCCTGTGG GCATGCGCGT TGTCAGTGGA
40201 GGAACGGTGC CTGTTGGCGT TGAGTGGATC GAAGCAGAAG TGGACATTTG TGCGTGGGTA
40261 GGGGTGATGA CTGTGTGAGT ACTTGGAGTG ACTGATGAGG TGGAGAAAGG TGGAACATGA
40321 GTGGTAAGTG TGGTCTGAGG GTGTGATGGG GTTGGATAGG TCGTGGTGGT CTTGATGGAT
40381 GTTGCAGTCA TAGGACCTGT GGAAGAGATG GGACTGCTCC CTGTAGGTGG GGAGTGTGTG
40441 GTGAAGGGTG TGGGTGGCCT GCTGCTGGTG GCCAAGGTGG TGTGGGCCAC AGGGGTGCTG
40501 GTTCCTGCAC TAGTGGACTT GGGAGTAATG TTGGTGGTAG AAGTTGGTGT GGTTTCCGGA
40561 TGGTGTGTGG AGGAAGCATG TGAGAGGATG GATGTAGAGG TTTTGGCTAT GCTGAATGAG
40621 CTGTGGGCTT GGGTGGTCCG AATGGTTGTC CCCGTCATTG GTGAGGCTGT GTGTGTGGAC
40681 CCTGTGGCCG TGAGCGTTGT CAGTGGAGGA ATGGTACCTG TTGGCGTTGA GTGGATCGAG
40741 GCAGAAGTGG ACATCTGTGC ATGGGTAGGG GTGATGACTG TGTGAGTACT TGGAGTCACT
40801 GACGAGCTGG AGAAAGGTGG AACGTGAGTG GGAAGTGTGG TCTGAGGGTG TGATGTGGTT
40861 GGATAGGTAG TGGTGGTCTT GAAGGATGTT GGAGTCATAG GACCTGTGGG AGAGAGGGGA
40921 CTGCTCTCTG TAGGTGGGGA GTGTGTGGTG AAGGGTGGTG GTGGCCTGCT GCTGGTGGCT
40981 GAGGTGGTGT GGGCCACAGG GGTTCCGGTG CCTGTACTAG TGGGGTTGGG AGTAATGGTG
41041 GTGGTAGAAT TTGTGGTGAT TTCTGGATGG TGTGTGGAGG AAGCATGGGA GAGGAGGGAT
41101 GTAGAGGTTT TGGCTGTGCT GAAGGAGCTG TGGACTTGGC TGGTCCTACT GGTGGTCACT
41161 GTTATTAGTG GGGCTGTGTG TGTGGACCCT GTGGCCATGA GCGTTGTCAG TGGAGGAATG
41221 GTGCCTGTTG ACGTTGAGTG GTTGGAGGCA GAAGTGGACA TCTGTGGGTG GGTTGGGGTG
41281 ATGACTGTGT GAGTACTTGG AGTCACCGAT GAGGTGGAGA AAGGTGGAAC ATGAATGGGA
41341 AGTGTGATGT GAGCTTGTGA TGGGGTTGGA TAGGTAGTGG TGGTCTTGAG AGATGTTGCA
41401 GTCATACGAC CTGTGGAAGA GAGGGGACTG CTCCTGGTAG GTGAAGAGTG TGTTGTGATG
41461 GGTGTGGGTG GCCTGCTACT GGTGGCCGAG GTGGTGTGGG TCACAGGGGT GCTGGTGCCT
41521 CTACTGGTGG ACTTGGGAGT CACGTTGGTG GTAGAAGTTG GGGTGACTTC AGGATGGTGT
41581 GTGGAGGAAG CATGTGAGTG GAGGGATGTA GAGGTTTTGG CTGTGCTGAT TGAGCTGTGG
41641 GCTTGGCTGA TCCTACTGGT GGTTGCCGTC ATTAGTGGGG CTGTGTAGGT GGACCTTGTG
41701 GCCTTAACTG TTGTTGGTGG AGCAATCGTG CCTGTTGGCG TTGAGTGGAT ATAGGCAGAA
41761 GTGGACATCT GTGGGTGGGT AGGGGTGATG ACTGTGTGAG TAATTGGAGT CACCAAAGAG
41821 GTTGAGAAAG GTGGAACGTG AGTGAGAAGA GTGGTCTGAG GGAGTGATGG GGTTGGATAG
41881 GTAGTGGTGG TCTTGAAGGA TGTTGCCGTC ATGGGACCTG TGGAAGAGAA GGGACTGCTC
41941 CCTGTAGGTG GGGAGTGTGT GGTGAAGGGT GGTGGTGGCC TGCTGCTGGT GGCTGAGTTG
42001 GTGTGGGCCA CAGGGGTTCT GGTGCGTGTA CTAGTGGGGT TGGGAGTAAT CGTGGTAGTA
42061 GAAGTTGGGG TGACTTCAGG ATGGTGTGTG GAGGAAGTGT GTGAATGTAG CGAGGTAGGT
42121 GTTTTGTTTG TGCTGAATGA GCTGTGGGCT TGGCTGGTCC CACTGGTGGT CGGCGTTATT
42181 GGTGGGGCTG TGTGGGTGGA CCCTGTGGCC TTGAGCGTTG TTGGTGGAGG AATGGTACCT
42241 GTTGGCGCTG AGTGGTTGGA GGCAGATGTG GCCATCTGTG CGTGGGTAGG GGTGATGACT
42301 GTGTGAGTAC TTGGAGTCAC CAAGGAGGTG GAGAAAGGTG GAACGTGAGT GGGAAGTGTG
42361 GTCTCAGGGT GTGATGGGGT TGGATAGGTA GTGGTGGCAT GGAAAGATGT TGCAGTGACA
42421 GGACCTGTGG AAGGGACGGG ACTCCCCGCC GTAGGCGGGG AGTGTGTGGT GTGTGGGGTT
42481 TGGGGCGTTG TGTATTCAGT AGTCGTTCTT GTTTGAGTGG TCTCTGTGGC TGTGGGCCTC
42541 GTGGGTTGTC CTGGCTGTGG GGTGGTTGGG CCTGTGGTGC TTGCTGGGGT TGGACGTGGG
42601 CCTGTCGTCT GGGTGGCCGT TGTTCCTGGC AGTTCCTGAT TGGTCGATTT TGCTGTGGGA
42661 ATTGGTGAAG TTGTCATCGT TATTGTTTTT GTTTCTCTAC CCTGACCTCC GCTGGCCCGT
42721 CCTTTTGTCT ATGTGACCTT TTTCTTGCCT GTCTGTGCCT CTGTTCCTGT GACATGGCCC
42781 CTGCTGGGCA CTCCAGCCTG CCCCATTGTC TCCATCTCAC CTGGACTTGC TCCACATCCT
42841 GCCCTGAGCT GCGTGCTGAC TCCTTCAGTG CAGCTGCCGC TCTGTGACTG GAACCCAGGC
42901 CCTACTTTAT CCCCTTCTGG TTCCCTGTGG CCGTGGTGGT GGCCCCCACC CTCCTGCACC
42961 TCTGCTCCCC AGGCCTGCCT TTGTGAGTGC CAGGCGGCCT TCCTTGCCTC CAGCTCCAGC
43021 CTACACTTTT GGGCTGCCTT CTCGCTTGCC CTCTGGGAAA TACGGGGTCT TCTTTATGGC
43081 TGAATCACTG AATGTGAGCT GGTGGTGGGA CCGGGTGCCT TCGGCAGTGC TGGCATCCCA
43141 TGGCGCCATG ACTTACGCAG CGTGGGGCTT GTCCCTGATG TGGCTGGGGT TGGTAGTGTC
43201 ATTGTGGTCC GTGTTGTGGA CTGAGCTGTG GACGTCGTGG CTGGGCTGGC GGTCGACGCC
43261 GTGGCCCTGG TTGTGGCCTG GGTCACTGTG GGTTTTGTGG CTGTCGATCT CAGTGTGGCT
43321 GTGGGAGGCA GCCCTGATGT GGCTTGTGGG GTGACGGCCG TGGTTGGTCT AGGTGGTTCT
43381 GCAGAGGACA GCCGCCCGGA ACATCCCCTT GCTGTGGGCC TGCATTTCGA AGGCTTGTGT
43441 CCCAGCCCCC TGCCCTGCTT CTGGGATCCC TGGCCTGCTG TCCGGGACTC AGCCTCCTTG
43501 GAGGGGCTCT TCCTCTTGCC TTTGTTAGGT CCTCCCACCG TACTCCTGGC TGTGGTGGCC
43561 AGAGCTGGGG CAGTGACCAC ATGCTTATGG GGCTGCGGCT GCTCCTGCGC CCACCCTTGC
43621 TTAGCTGAAC GGACTGTCGT CCTGTCCCCT CCAGGGTCCC TGGGCAGCAG ATGGGGCCCT
43681 GCTCGGTGTG GGGCAGGGGC AGGCTGCCTG GCAGAGGCCC TGCAGGTCCC ACACGGTTTC
43741 TAGTGACAGC AGCCAGCAGC AAGGAATGCC CCATGCCATG ACCAGCTTGT CTTTAAAAGT

43801 TTTTCCGAAA AATCCCCAGT TTGGCTCCCA AGCATAGGAA GTTCTACGCT GGGAATCTGC
43861 TTAGTGGCAG ATGTGAGCCA GGAAGCAGGG CCATCCCTAA GCCCACCCCA GAGGTGTACC
43921 TAGGACCTCC TGCAGCTGCC CTCTCCATGG GCTCAGCTGG AGGCTCCTTA CCTCCCGAGG
43981 AGGCTGTGGG CTTGGAGGAT GTGAGCGTGG CTGGAAGGAG GGGTGTCTGG GTGGGGCTGG
44041 CAGGGGTGTG ATTAGAGCTG GGTGAGGGTC CGGTGGAGCT GAGAAGCCCG ATGGTGGTGG
44101 AGGTTCCCGT CATGGGCCAG ACTTGCGTGG GCCGTGAGCC TGGGTGGGCG GACATGCCAT
44161 CAGGGCTGCA GGGTACCGGC ATATCCTTGG GGAGCACCCT CCCCTCTGCC TGCTTGGCCC
44221 TGAAGCCGGG CAGCCCTGCA GGGGCCAATG ATGTGCAGTT GAGGGCTGGC CTGTGGCACT
44281 CTGAGTGCAG CCTGGCTCCT GGCTGGGCCT CCTGCATGGC GGGGGTCACC TGTTGAGGCC
44341 CCACTCTGTG CTCATCCGTG GGTCCAGCCA GGGCCATGGG GACCAGGCTG CTTCCTGGCT
44401 GTGGGCTGAG CTCCTCCCCA ACTCTGATTG CCAGGTTAGA CCTGAGAAGG GCACAGGTAC
44461 TGCCTGCCCC CTCCCTGCTT CCCACCCGAC AGATTTGTCC AGGGAACCGA GGGGAGCCCA
44521 GGAAAGGGCC TGCGTCACCT TGGCACCTAG TGTGCGTGCA ATTACCTGTG GTGGGCAGCT
44581 GCGGCGTGGT GGGTGGCTGC GGCGTGGTGG GCGGCACTGC AAGAAGATGG GGTCAGCTCC
44641 CTGTGGTTCT CTAAGCCTCC CCACCCCGTG GGGCCTCTGG GAGCTCCGAG GGCCTGAGTC
44701 AGAGATGCTC ACCAAGGCTG TGGTGGAGGG GACTGGAGGG GCTGGGAGCC CACCCTCCCC
44761 TCTCTCCCTT TCTCCTTCCC AGGATGATTC CCACTCTATA CCCCAGGCAC CCTGGCAGGC
44821 CTGGTGAGGG TAGGGGGAGC TGGGAGCTGG TGGAAGAGGG GATGGGAGGG CCCAGTGGGG
44881 TTCAGTGCTG TGTTTTTTTC TCTCTGCTGC CATGGGCTGG AGGCTGCCTG AGTCTCTGGA
44941 GACCCAAGGG GCCAGGGTCC TGGAGAGTGG AGTCCCAGAG CTCACGTAAG GGCTCACAGC
45001 CCCCGAGGGC TCTCTCCCTT GTTTGTGGGA TGTGGACGTG CGTTCTGCCT GCATGCAGGC
45061 AGGGGCAGGG TTCTCAGGGC AGCCGACTGG ACTTACTGCA GGGCACGCAC ACCCCCTCCT
45121 CGTGGTCGAA GTACTCATCC TGGGAGCAGT TGTAGCAGCC TAGGGTGGAG AACGGCCAGG
45181 GTCTGTGTGA CTGGTGGCCA GCCAGGCCCA CCTGCGTGTT TCCTGCCCTG GCGGCCTCCT
45241 TCCTCTCTGC TTTTTTTTTT TTTTTTTTTT GAGACAGAGT CTCGCTCTGT CGCCCAGGCT
45301 GGAGTGCAGT GGCACAATCT TCGCTCACTG CAACCTCTGC CTCCTGGATT CACATGATTC
45361 TCCTGCCCCA GCCTCCCAGG TAGCTGCGAT TGCAGGCATG TGCCATGACG CCCGGCTAAT
45421 TTTTGTATTT TTAGTAGTGA TGGGGCTTCG CCACGTTGGC CAGGCTGGTC TCAAACTCTT
45481 GACCTGAGGT GATCTTCCTG CCTCGGCCTC CCAGAGTGCT GGGATTACAG TTTCACCCGC
45541 GCACCTTGCT CTAAGCCCCT CCTTTCCTGC CATGCCTTCC TCAGGCCTTG GTCTCTCATA
45601 CCCTGCTTAT CACCCGGGGC TGGGGCTTCG GTGCCATCTT CCAGTCCTCA CCTCCCCCTG
45661 GACCTCAGAC ACTGGCCCCT CTTCTGTGCT CCCCGATGCA GGTGCCCTGC ATTGCCCTCC
45721 TGAGCCCCAA CACATCTGTC CTCTGTGTGT CCGGATGGCT CCAGACACCA CCCTCCTCCA
45781 CCACTTGGGC CAGGACCTGT TAATCCCAGG ACCCTCTGCA TTCAGACCTC TGCCTTGGGG
45841 CAGCCACAGG CCTCACAAAG ACCCCTCCCT CCCGGCCACA CCCCACCCCA CAAGTCTGTG
45901 TACCCCACAC CCCTCTGCAG CCCGGTGCCC CTCACTCGCT CCCTCTGCCC TCTGCAGCCC
45961 CGCGCCTCTC ACTCGCTCCC TCTGCCCTCT GCAGCCCCGC GCCCCTCACT CGCTCTGCCC
46021 TCTGCAGCCC ACGCCCCTCA CTCGCTCCCT CTGCCCTCTA CAGCCCCGCG CCCCTCACTC
46081 ACTCCCTCTG CCCTCTGCAG CCCTGTGCCC CTCACTCGCT TGCTCTGCCC TCTGCAGCCC
46141 CGCGCCCCAC TCGCTCCTTC TGCCCTCTAC AGCCCCGCGC CCCTCACTCA CTCCATCTGC
46201 CCTCTACAGC CCCGCACCCC TCCACCGCTC CCTCTGCCCT CTGCAGCCCG CGCCCCTCAC
46261 TCGCTGGCTC TGCCCGCTGC CCTGGCTTTC TTAAGGGTTC TGGGCACAAG GCTGCTCCTG
46321 GCTGCTCCTC TGGACACTTT CCTGGCTCCC CTCCTCACCT TTCCCATCCT CGCCCTCACA
46381 TCTGCCTCCC ACCAAGGGCC CCAACCACCC TGTGTGGCAC AGAAGCCTGT GGCTCTCCCA
46441 CCCCAGCCTG CCTTTCTCCT CTCCATGGTG GGCGCCTCGG CCGACACTGG GTCTGCCTCC
46501 TTAAGCACCT CTCCCTGGCT GGGAGGGAAG CTCCAGGTTG CAGGACTGTT TGTTTTGTGT
46561 AAGGCCAGGT CCCCTGACCT GGAGCCGGCC TGGAGGACAG AGGTGCTCAG GACCCATCTG
46621 CTGAGTGACT GTGTGTGTGA AAGAATGAAT GTGCAAATGA ATGCATGAAT GTGCGTGTGT
46681 GTATGAGCGA ATGTGCATGA ATGAATAAGC AAACATGAAT GAATGAATGA ATATGTGTGA
46741 ATGAATGTGC ATGGGTGAAT GTGCGTGAGT GAATGTTGAA TGAATGTGCG TGAATGTGCA
46801 TGAGTGAATG TGCGTGAGTG AACAAATGTG CGTAAATGAA TGCATGAGTG CGTGAATGTG
46861 CATGAATGTG GGTGAGTAAA TGTGTGAATG AGCATGAATG AATGTGAATG TGTTGAGTGA
46921 ATGTGCGTGA ATGAATGTGA ATGAGCGTGA ATATGTTGAA TGAGTGTGTG TGGGTGAATG
46981 AATGGATGAA TGGAGTGAAT GTGCATGAGT GTGCGTGAAT GTGCGTGAGT GAATGTGCGT
47041 GAATGAATGT GCATGAATCT GCGGGAATGT GACTGCGTGA ATGAGCATGA ATGAATGTGT
47101 GTGTAGTGAC TGCGTGAATG AATGTGTGAA TGAATGTGCA TGAGTGTGTG TGAGTGGATG
47161 AACAAATGTG CGTGAATGAA TGAATATGCG TGAATGAATG CATGAATCTG CATGAATGAA
47221 TGTGAATGTG CGTGAGTGTG TGTGAATGAA TGTGTGCAAA TTAATGAATG TGTGAATTAA
47281 TGAGCATGAA TGAATGTGTG AATGAATGAA TGCGTGTGAA TGAATGAATG TGCATGAATG
47341 AGTGAATGGG TCCCCCTGTG TATCTGCGTT GCCTCCCGGT CACCTGGCAC CTTCGATGTT
47401 GCTGCCTGGG ACGCTCTGTG GCTGGCTGGG GCAGAGGCAG GGCTGGTAGT GCCACGTGCA

47461 GTTGGCCTCC TGTGTGTACT GGTACTCGCC ATGGCCGTCC TGCGTGTGCG TGTTGTAGAA
47521 GCCGCAGTAG ATGGCTGGGA GGAAGGGAGC TGTCAGCTGG TGGGGTTCCT GGCCCTGGCC
47581 CTGGCCCTGA CCCGGTGGTT CCCCTGGGCA TGCATGGAAC CTGAGTGTGG GCGGGGAAGG
47641 TCTGGGCCCT CTTGGTGAAG CCTCCCCTGG GCAGCTGGGG GCTGCGGGAG GGCCAGGGTG
47701 GCCCCGCAGG GCACAGCCCG GCGCCTGTCC AGGGAGAGGG GCCTGTGGGC AAGGGCAGCC
47761 CTGCGCCGGC CCTTAGTGGC GCTGGGTGGC AGGGGCTGGA GCAACCTGTG GGAGGGGTGG
47821 TCACTCACGG CAGAAGGCCG GGGTCCTCCA GTCCACGCAC ACACCCTTGT CCAGACAGGC
47881 TTGGGCGTAG GCAGCCACGG CATCGCACAG ACACTCACAG TCCCCGCCAC TGTCACACCC
47941 ACATGCGTCG CGCACGCAGG CCTCGTAGTA GGGCAGGTGG TATACCTGCA GGGGTGTGTG
48001 CCAGTCAGTG TCTGGCTGCC GGGGGATGGC GGGGCATCAG GCTTTGCCAC CTGCAGGGCC
48061 CTCAGTGTGG TCAGGCCGGA GTGTGGCGGT AAGGGCGCTG GGACTGGGTG AGCGGCACCA
48121 CGTGGACCTC AGCCCTGACC GCTAGCCACG CTCCCGGAGC CGATGCTGCC ACGGAGGCCT
48181 GACCCGAGCT CACGCCTTGA GACCCGCCAT CGGGACCAAG ATGCCGCTGC CGCTAACCAC
48241 GGCCACTGCA GTCCCACCCA GGGTCTCTGC TCCCCCCACG TCACGCTCAC ATTCAGCGGG
48301 CCAGTGCCAT GCTGTTCCCC CGCGGGGTGC CCCCATCCTC TCAGTTCTTA CTGCGCCGCG
48361 ATGGCCGGAT CACGCCCTGG GCTGGGAAGT GCACGCCCTG CATCCTGGAG GTCTCCCTGA
48421 CCACCAGCTC CTGAGCAGGG GGCCCACCAG GCGATGCTGC CCCAGGGGAC TCGTGGCACT
48481 GTCTGGGGGT GCTATTGGCA TCTGGTGGGT GGAGGCCGGG GGGCTGCTTA ACAGCCACAG
48541 TGCACGAGTC GGCCCCACAT CAGGGCCCTG CACTCAGGCA GAGGGTCTGA AACTCTCTGC
48601 ACCCTGACCT GGCTGGTCTG GGATCCCACG GAGGGAGAAC CGTGCCTGGC TTCCCCGCCC
48661 CTTCCCCCGC CCCCACCGGA CATTTGTGGA GGGGCAGGCG CACAGCCCTC GTGCCCGGTG
48721 CCCACCTTGC TGTGGCAGGT GGCAAAGGTC TGGCTGTTGA TGACGCTGCA CTTGCGCTCG
48781 GCCCAGGAGC GCCGGAAGGC ATTGAGACTG CAGGGGTCTG TCACGAAGCT CACGTCCCCG
48841 CACAGCGGGC TCTCCTTCCA CGAGTTCACC AACTCCAGCT CGCTGGATGC CACGTACCTG
48901 CTGCGCGTCT CGAAGTCGTC CTTCATGTTC CCGTTGAAGT TGCCACACAA GCCGCAGAGG
48961 GGATCCTGCA GACGGTGGCA TCAGGCCGGG CCCAGGGGCC GTGCCATCTG TCTCCACCCC
49021 TGCATCAGGG AGGGCCTGGG AGGAGGCAGA GGGCGTGCGG TACCTGGGAG GCACGGGCGA
49081 TCCTGATGAG GATGGTCATG TGCCTGTTCC AGATGAGCGT CAGGTTGTAC CTCCCGGGGA
49141 TGCTGATGTC CACGACAAGG CTCAGCGCAC CCGGCGTCAC CCCGAGCTGC ACGTGGGGCT
49201 CCTCCCCGGT GACCGTGTAG TTTCTGTCCG CCAGCACCAC GGACAGGCCC TGTGGGGTGG
49261 GGTTGGCATA GGACTGCCTG TCTGTCTCCC CCGGCCCCTG GCACAGCCGT GCTGGACCGA
49321 GCTCTCAGAG ACAGAGCTGC CCAGGTCTGT TAAGGCCATG CACAGGAGCG CCTGCTGAGA
49381 GCCAGCTTGG GGCAGAAGGG CCTGGCATGC CTGGAACAGG GCTGAGGGCT GAAGCTCGGG
49441 CTTCCTGGAG AGTCCCTCCC CTCTGCCCGG GAGACATTCT GCAGTCCCTG AAACCTTGTG
49501 GGGCACAAGC ACCCGTGGTC CTGAGCGTCT GGCCAGGGAG GGGCAGCAGG CACCCCTCAA
49561 GGAGAGGCAG GCGGGGAGGG CTGCATCTCG GGGCAGGACC TGGAGGCTCC AGTGCGCGGG
49621 ATCCAGCTGT GTGGCAGGGC CCCCTACCGC CCGTCCTGCC CTGCCAGAGT CTGCCCGGCT
49681 GCTCACCCCC AGGAAGATCT TGATGGCCCG TGAGCATGTG ACCCCGGAGT TCCCACAGAT
49741 GACGTTCTCT GTCAGGATCT TGAAGGTGGG CTGTGAGTCG TTGACACCAC AGACGTCCTG
49801 CAGGGAGAGG GCGCTGAGGA GGAGCCCTGG AGGCCGTGCC TCTGGGTCCC CGGCCCCTGC
49861 GGCCTGGCAC CCGATGGTTA CCGTGGCCAG GATGTACTCG CAGTTGCCGT CGAATACGAA
49921 GCGCTGGCCG TCGAAGGTGA TGACGTGGCC CTCCCCGTAG AGGGTGCAGG TGGATGGGCA
49981 GTGGGTGCCC TGCTGACAGG CCCACCTCCC CCTTGAGCAG GAGCTGTGGA GACAGCAGGT
50041 GTGGGTGGTG GGCCTGCGGC CCTCCTGCCA TACTGGGTGT GGTCCCTGGA GAGGCCAGAC
50101 TGCACCTCTG GACGCCCCCG CCTCTGGGAC AGCCCCACCC CGGGCAGCCT CCGCCTGCCC
50161 CAGATGGCCC CAGGAGCCCA GGGCGTCTGA AGCGAGGCTT TGTCTCACCA GGTCCTGCAG
50221 TCAGTGTGGA GCTCAGCTCC TCCAGGGTAG GAGACCCCCG AGAACTCACA TGGGCACTCC
50281 TCGGGGGGCA CACACTGCCC GTCGGCATTC TCGTAGAGGC CCTCGGCGCA GACACAGCCA
50341 GGCTCACACT TGGTGGGCAC CTGGAGGGAG GCAGGTCAGC AGCTCCTGGG AGGGTGGCCT
50401 CAGCCAGCTG CTGCCCAGCC CTGGGCCCCT CTGAGACTGC CCGGCGTGTC TCCCAGGTCC
50461 TCTCCCTGAA TACAGCCCTG CTCTGTGGCC TTTGTGGGCA GCTGGGCTTA CTGCCTTAGC
50521 AAAGCTTCCC CCACCTCGTG GAAGGTCCTG GGACCCAGTG CACACGGCTG GGCTGCTGTA
50581 CCCACTCCCA CACATGGGCA GCCCACCCTC CCCGAGCTGG TGCCCACTGT CCTTAGGGAG
50641 CCCTGCAGAC GAGCCCCCGG GGCCTCCAGC TGCCTGGCCA CAGGGCCGCT TCCACCTCGT
50701 GGCCAGCCCC AGGCACCCGC TGCAGGGCAG AATGCGGCCA GGTCTCCCGG AGTTCTGTGG
50761 AAACCCCCTC ATGGTTCAGC TGGGGCCCGG GCATTGTCCC TGCTCCTCGC GCGCCCCCTT
50821 ACGCAGGCAA CACCGGTGGC CAGCATCTGG CATGTGGGGG CACAGGCTGC CCCAAACTTG
50881 TTCTCGGAGG ACTGGCTGCA GGACTTGAAG GTCTTAGGGG CCTGGCAGGA GGCTGCAGGA
50941 AAGAGGGGTG CGCGGTCAGG ACACTCAGAG GAAGCCGGGG CCCCTCACAG TCCCAGCCTG
51001 CGGCCAGCGC TGCTGTACGT ACCCAGGAAC ATCTGTGGCC GCTGCGGGCA ACTCAGCCGC
51061 CCGTTGATGC AGTGGCTGCA AGAGAGAGGC TGCGTGAGAC CCCGGGACCT GGCAGGGACC

51121 CCCCGCCTGG CCCCTGCCCG GTCCCTCACC AGGTGATGCC GTTGATGACA GTGGACTGCT
51181 CGGCCAGGAT GAACTTGTAA CCCTCCAGTA TGCACGGGCA CTGGGCCTTG CGCACACACT
51241 CGCCCTTTTG GTTCAGGTAG GTGCCATCGG GGCAGTTGCA ACCGTCCACG GGCACGGCGC
51301 TGTGGTGGCA CTCGGTGGCA CGGTCCGACA GCGACAGGCA GGTGCGCTCA CAGGCTTGGC
51361 TGTTGTAGCT GAAGGTGGTG TTACCCGTGC AGGGGATGGC TGTGGGGGAC CCGGGCATCA
51421 GACTCTCCGG GAGGGGGCGG CCGGGAGGGC AATCTCGGGT TCCCCTGCCT GCCGGGCACT
51481 GCAGAGCCTC AGAGCTTGGG GTCCCAGGAC ACCCCCTCGT GAGCCCAGCC TGCCGTGACC
51541 CCGCTTAAGC CCCGTCGGGC ACTCACTGCA GTTGTCCACA CTGCTTCTCC AGCCCCAGAG
51601 CAGGACGCCC CGCAAGGAGC AGGCGTGTAC GTAGTCGCCC AGGGCGGCAC AGATGTGGGG
51661 AAAGGTCTCC TCGTAGTTGC AGGCCTGGTA CACGCACCTC TGCGGGCAGA GAGCCAGCAT
51721 GGGCTGGTGG CAGGCACCCT GCCCTGGGGA CATGGGGGTC CCAAACCTAT GCCCTTGGGT
51781 GCCTGAGACC TTGTCAGCCA CCACAGCCTC CCCTGCAGCC CTCCCAAGAC GCCCTCGGGC
51841 CCTCACCTTG TAGAAGGGTG CAGGGTTCAC TGTGGCGTGG CACCTCTCGA ACACCGTGCC
51901 TGTCCTCAGC AGCATGGAGC AGTGGGTCTC TGCACACACC TCTGGGGATG ACAGGCCCGG
51961 GCGTGAGTCC CGGCCCCTCC CATTCCAGCT ACGGCTCCTC CCAGGGACCC CCCACCCTGG
52021 CAGCTGGGCC TGTGGTCCAG TCCAGGGGTC TGTCAGAACT GTGGGCGCTG GGGGGGCAGC
52081 CAGGGGAGTG GGGGGCCGGA CACTCACTGT TGAGCTGGCT CATGGAGCAG GGGTCAGTCT
52141 CACGCTCCAG AGCGGCCGGA CAGTTCCCCG CCCGCCAGGA GTCCACAAAC AGCGAGGCGG
52201 TGCCCTCGGC GATACCCATG CTAGTGGTGA AGTCATCCGT TGTGTCCCCG TTGAAGTTGC
52261 CGCAGAGCCC TGAGCCGGCG GGGCGTGAGC TCGACTTGAA CCCATCCCTC CTGCACCCAT
52321 TCCTGCCCAC ACGCCCTGGA GCTCCCGTCC TTCCTCTAAC AGCACCCTGG GTGAGAGGCT
52381 GCGTGGGCCA CACGTGCCTG TTACCCCTGG GGGCTCCCCG GACACAGAGG GTTGTGTGAA
52441 CCTCTCTTGG ACCTTTCTCC TCCCCTGGTC ATGGCCAGAC CCTGTAGGGA TGAGGCAACA
52501 GGGCCACCTG GAGAGGCAGG GACTCACCTC TGGTCTGACC TCTGAACTGG GGCCCAACAG
52561 TGACATAGGC CTGGAAGATG GGGCGCAGCT GGACCACGAG CTCCAGCCCG AAGCTGGTGG
52621 CCATCTGGAG GTGGGTGGAC GTCTGCCTGA AGACCGTGAT GTTGCCTGCA GGACGCAGTG
52681 CTCAGTGGGC CGTCTGGGCT CCCTCCCCAC CCACTGCAGC CCGCCCCGAA GGCACAACTC
52741 TGGGGGGCCA CAGACTGGGC CAGGACGTAC GAGTCTTGTA TGGCAGCCAC TTGGCTTCTC
52801 CGTTGTTGGT GACCACCTCG TCCTGAGAGA TCACAATTTT GTCCTGGAGA GAGGGTGGCC
52861 TGAGTCAGGG TGCAGGCACC AGGGAGCGGA GCCCTGCTGG CAGGGATGGG CGCAGGAAAG
52921 GCCTTACCTG CCTGGAGAGG TAGACCACAG CCACCAGGGA GGTCTCGGAG TGTGAGACGC
52981 CGGACTTGTC GTACACAGCC ATGAGGGCAC CGTCCTCGGG AAGCTGGGGG CTCTGCGAGG
53041 GGGCGGGGCT CAGACACGGG TGGGGTCACC GGGAGCGCCC CTCCCCACGG GCCACAGGGC
53101 TCGTCCTACC TGGAGGAGGA TGTAGGTGCA GGTGCCGTGG AAGCGGTAGG GCCTGGCGTC
53161 AAATGTGGTA ACAAAGGAGC CACCTTCCAG GGAGCAGTGT CCGGGGCACG GCCGCTCCGT
53221 GCACACCCAG CGGCCCAGGG TGCACCGGCT GTGGGTGGGC GTGGGGGTAG CGGCATGGTG
53281 GGCAGGGCCG CTGGAGCCAG ACAGCACACC CCTGCCTGCC CTAGGCCAGT GGAACCCCTG
53341 CCGGCCGGCC AGAGCCCCCT CCGGCGCCTC ACTCACCAGG TTTGGCAGGC AGCTATTGTG
53401 ACCTCCCCGG GGGCATACAT GGCGCCGTGG AGCACACAGG GGCACTGGGT GACGGGCACG
53461 CAGGTGTGGT TATTGGAGAG GTCATTCAGG ACCGTACCTG CAGGAGAGGG TCTTCTTGGG
53521 GCTTGGGTGG GACTGACTGC CTCCCACTCT CCCCTCCCTG GCTCCAGGGA GACGCCCCCT
53581 CCAGCCTGGC TCCAGGGAGA CGCCCCCTCC AGCCTGGCTC CAGGGAGACG CCCCTCCAGC
53641 CTGGCTCCAG GGAGACGCCC CCTCTAGCCT GGGCCATGAC CTCTCTCCAG CTGCACTTGC
53701 ATTCGCAACT CTATGTCAGG CCCTGGGCGT GCACAGCCCC TCGTGCCAAT GTGCCCGGCC
53761 CAGGTTCCTG GTGAGTGGTG GCCTCCAGCA GGCAGGGACG TGTAACTGTC CCTTGGGCCC
53821 GGCTCAAACC CTGCCTTCCC TCGGCAGCTC CTAGACCGCA GTGAGCAGCC CTCACCGCCC
53881 GTGGGTTTGC AGGGGTGGCC GTGGCTGGGG GACCTGCAGG CTGTAAGCGT CCAGGCGGGA
53941 AAAGCCTGTC CCAGGGCAAG AGGCGCCAGA GCTGGGACTC AGGCAGCAGA ATGTTGGGGT
54001 GACCTGGGTC GGGGTGGGTG GGGTCTGACG TCCCCTTGTC TCTGGGTTCT CTCTAGGGGT
54061 CCCGGGGAGA GAGAGTGCCT GCGACTGCCC TCACCTTCCG GGCAGAAGCA CCCGAAGGTG
54121 CAGGAGCTGG AGCAGCTGTG CTGCGGGTTG GAGCAGGTCT TCACGCAGGC CGAGCCGCAC
54181 TCCTGGTACA CCTGGTTGGC CGGGCACTGA CCCACGGCTG TGGGCACACG CGGCTCCGGT
54241 GAGAGGGTCC CACCCCCCCC ACCCCTCCCT GCCCCACCCC AGCTTGATGG AGGACTTAGC
54301 CCAGCCCTTC CTCCATCTAG AAGCAGCAGC GAGGATCTCC TGTCTCTCAG ACCAGGAGGG
54361 ATGCAGGCGT CACGTCAGGC AGTCCAGGGG CTGGGAGAGC TGGGTCTGGA GCCCTCGGCC
54421 TTCCTGCCCC TCCCTCTCCC TTCCCTGGAC TCACAGCACA GGCCGGGGCT CCGCCAGCGG
54481 CGGACCGGCT GGCCCACCAT GCTGCACTGG CGGGAGTACT CCGACAGGGT GGCACAACTG
54541 CTGTTCTGTG GGCCTGGCTG GGGGGCTGCG GCCACGTCCG CCTGGCAGCT TAGCACGAAG
54601 GGCTCCTTGG ACACGCTGCA CTCAGGGGCC ACCAGGGTCA GCAGCTGGGT GCAGATCCGG
54661 GCCTGGGGGG GCCACTCAGG GTCATGGGGG CAAAGGCCAC ACCCCATGCC ACCACGACTG
54721 GGGAGGTCGG GCAGGGCGTC TGTGATGCGG CTGCTTGTGG GGGCCCTTGG TGGTCTCGGC

54781 GACCCTGTCA GACCTGGGGT CAGCCCCACC TGGAGCCCCC TTGCTTACGT GCTGGGCCTG
54841 CCGGACGTGG GTGCTGGGGA TGTCCTGGAA GGTGCAGATC TCGCCGGGGT CGTCCAGCTT
54901 CTGGAGGGCA GCAAACTTGT GGGGTTCCAG GAACTTGCCT GGGGTGCAGA ATGGGGGTCA
54961 GCACCGTGGG GGCTGGGCCT CAGAGGCCCC CCTGCCCTGC CCCCCACCTA GAGGCCCCCC
55021 AGAGGCCCCC CAGCCCTGCC CCCACCTACC CTCCTCACTG ACAAACTCGT TGGTCACCTT
55081 CCCGTCAAAG TTCCCGCAGA GCCCGCACAT CTGACCCATG TACTTCCGCT CCACCAGAAC
55141 CTGCGGGAGA CGGCTCTGCT GGGGGCCCGG GGGCCAGGGG CCCCCTCATC TGCTGTGGAG
55201 GGCTCTCAGT TCCTGCTCCT GGACCCAGAG CCCCCACCAT CCCCCCACCT GCTCATCTGC
55261 CTCTTCTTAT GGGAGGCTAA TTTTCCAGTG GAGAAGCCGA GTCACCCACA AAACCCAGTC
55321 CTGGCTCATG TTGCTGGTCA GGGGTCAGCA CTGCTTGGCA CGAAGGGCCT GGCTGCATGG
55381 CAGCCTGAGG GCTGGCTGGG ACCCCCAAGG AGGGCAGCCC CTCCCAAGTC CCACCTGCCC
55441 CCCCGTGCTG CGGGTCTCCA GGCCCACCCT GGCCCTTCTC TCCTCACCAT GAGGTGGCTG
55501 TCAGGACCCC ACACGACTTC CAGCTCCAGC TCCAGCTGCT TGGCCACCAG CCGCACGCTC
55561 TGGCCGAAGG GTGTGATCTG GAGTCCATTG CTGGTATAGG GCAGGCTGAT GACCCTGTGG
55621 GGCAAGGGAA GTCGGTGGTC GATCCTCAGT CCTCCGGCCC CCGAGCCCCC GGGCCCCGGC
55681 CCACCTGACC TACCCGATGT CCTTGACTGA GATGATGGCT TCGCTCACAG TGACGACGGA
55741 GGCCCCCAGC TCCACGATGA TCCGCGAGAT GCTCCCGTCT GGGCCTCGCC GCAGCTGGAC
55801 ACTGAAGGTG GGGAAGGCGT CCTTGCAGGT GGCCGCGAAG ATGTAGTTGC ACGTCCCCGA
55861 GAAGTCGTAC ACGTGGTGGT CGAAGGTGGA GAAGTGACCA GCCCCCCACG TGGAGCACTG
55921 GCCTTTGTCC GGGGCTACAG AGAGAGCAGT GCTCACACAG CCCTGTGTCC CCACCATCCT
55981 GGCCAGGCAG GGCTGGGGCA GGCAGAGAGG TCACTCGTCT CCCTGGGCCA GGGTTTTTGA
56041 GTTGGGGCCA GGCTGTGGAA ACCCCAGACA TCCGATGAAC CTGAGTGCTA TGGTATATGC
56101 CTGGCCAGGA GTCAGCACCG CTGTGGGCAT GTGCACACAC GTGTGTGTGG GTACACGTAT
56161 GTGTGTTGTG TGTGTGCACG TGTGTGCACG TATGTGCGTG TCTCGGGTGT GTGCATACGT
56221 GTCAGGTGTG TCCATGTGTG CATTGTGGGT GTGTGTGTGT TGGAGGGCTG TGTAGGCGTG
56281 TGAGATATAC ACCTGCATGT GTGTTGGGTG TGTGTGATTG TGTTGCATGC ACATGTGTGT
56341 TTGTGTGAGC TGGGCATGTG TGTCGAGTGC ATGTGTGTGC ATTCGGGGTG TGTATGTGTG
56401 TGTTGGGTGC GTGTCTCAGG TGTGTAGGGT GTGTACATAG GGTGCATGTG TGTGTAGGGT
56461 GTGTGTGTGT TGGGTGCGTG CACATGTGTA AGTCGTGCGT GTTGTAAGTG TGCATGTTTG
56521 CATGTCTGGG ATGTGTGTGC ATGGGTGTTG GGTGTGTGTG CATGTGTTTG TCAGGTGTGT
56581 TTGTGTGTAG GTTGTGTGTG TTGGGTGTGT GTGCATGTGT GTTGGGTTGT GTGTCTGTGT
56641 AGAGTGTTGG GTGTGTGTGT GACGTGTGCT CATGCATGTG TCATGTACAT GTGTATGCGT
56701 GTGTCAGGTG TGTGTGCATG TGTATATTGG GTATGTGTGT GTGTTGGGTG TATGTGCATG
56761 TGTGTTCATG TTGGTGCATA TATGTGTGTT GGATGTGTGT GTCTTGGGGG TGACCTGGGC
56821 TCCGGGCCCC TCTCTCCTGC ACACCGGGCC TGGGTGGTCT GGGCGGCAGG ATCAGTGGCC
56881 GCTGTGTTCT TACCTGTCTG TGGAGAGTCC TTCAGCCTCT GGAGGCCTGG GCTGGTGTAG
56941 GAGGTGTTAG CCAGACCTGT GTGGACGGGA CCCGCAGTCG GTGTGGGGCT ACCCCGTCGT
57001 CCCTGAGGGC GCCGCTCACC TCTGCTCAGG GCTGCTCCGC CCGTTTCCCT GCACACACTC
57061 GGCGTGCGAG AAGTGTCCAT GGCCCGTGGG GCTCGAGGCC TCAACAGCAA GCCAAGCGCT
57121 TGGCCTCCCA TGCTGTCCTT CACGAGCCCC AGCTTCCTTC CCTGCTCTCG TTCACTCCCT
57181 CGTTTGTCCA CTCAGTCCCA GTTCAGCCGT CAGCCCCTCG GGGTTCGGCA GATGGCGGGC
57241 ACCCTGTGTG CAGGGTACTC ATCCCTGGGG CTTCTGGGTG GGGCTAGGAG GGGCTGGTGC
57301 GGGTGGCAGG GGCTTGGCGG CGGAGCCAAC AAAGTGGGCT GTGCCCGCCT CCCCAGCTTG
57361 GCCGCCTGGT TTCCCTGAGG GGTCTGCGCC AAGGCCCCAC AGCCGGTTCT CCCTGCTCTC
57421 GGTGGCTCCG GGGCTCTAAA CATGGCCGCT TTCCTGCACG TCAGCCTTGA GACAGCCTTG
57481 ATGTCAGGCC TGGCACTGAG GCCACTGTTG TCAGAGAAAC ATCCAGATAG CCCAGTCACG
57541 GTGACTCCAG GGCAGGGCAG CGGGGGACGT CCCACCCTGA CTCCCAGAGG CTGGGGTGGG
57601 GCCAACACCC CCCACGTCCC ACCCCCTGTA CCCAGAGGGA GACTTTTCCC ACCTCTGGGT
57661 AACTCCCCGG ACCCTGCCTC CGAGACTATG ACCCTTCCTG TGGCTCCCAC GAGCCCCCGA
57721 TGTCTGAGTG GTGGTGCGGC ACCTGAGCAG GACCCGTGAC CTCTCCAGGC CCTTCTTGGG
57781 GCCGGGACCC TCCTTGCCCC TGCCCATTGG TTAGCAGGGC ACTCACAGCA CCTCCTTCCA
57841 GATGTAGCTC AGAGATCCCA TTCCTGCCAC TCCTCACCTG TGCTCTCACC CCAGGCTCTG
57901 CCCACTTTCT GGGACTTACT TGATGCTGCC CCCATTACCG CTGGACACCT GTGTCTCCGG
57961 TGACGCAGGG GCAGGGGCCT GTCAGCTGGG AGATGGTGTT AACCGCGGTC CAGGGAGGAG
58021 CCAGGCTGCC TGCCCCTTGG CCGCCTGGGT GAGTCCTGTC TGGCACAGGC AGGAGGTTGG
58081 GTATTTGAGT GACTGGATTT AAGAAGAATG CCAACCTTGG CTATGTGGGA CACATATTTG
58141 GCTGTGGTCC CCTTTGGCCT TGAGATCGTT TTCTGATGGG TTGCACCTGG CTTATCCCCA
58201 GTGTAAGCTG AGTTTACTCC AGACTGGTGT GGGGGTGGCT CATGCTGGGC CCTGAGCCTG
58261 GCGAGCACCT ACCCCCTCAC TCATCCCCCT CCTGGGGGTC CCAGTAGGTG TCATGGTCCT
58321 CAGCCCCCCG CCATTCCTTC CAGGGATGAT CCACAGGGCT CAGAGCACCT GCTGCAGGAG
58381 TGCGCAGCAC ACCGAACCCT CAGCCATGGA ACTCAGTGGT TCCTGGGATG GGGCCCTCGC

58441 TGGCCTCTGG TGGGGGAGGG GTGGGAGAGC TCTGAGCGGG AGGAGCAGTT GGCTGCCTGC
58501 CCCTGGGCCA GCACCTCTGG AGGCTGCAGA GCTGTGACCG GTCTCCCCTG GGCAATGCCC
58561 CCTGCAACTC AGTTTCTCCA CCTTTCCGAT GGGGGGTGGC ACTTGGGGGC CGTCCCCGCT
58621 GTAGCACAGA CACCTCGGGC CTGTGTTTCG GTGCCCTCAG CCCTGCCAGG CTGGCTTCAC
58681 TGCCCCGGGT GGGTGCCTCA TTGCTGCACC CCCTGCGCCC CCCGCCGCCC TCGCCCTGCC
58741 CCACCCGCAG GAGGTTCCCC GAGTCCACTG GCTCCAAGTC GGCTGCCTCC AGCTCTGAAG
58801 TCCAGCGGGA GACTCCTGCG TTCCTGCCCA GACTGGCTGT GCTTCCCCTC GGCTGATCCC
58861 TTGGCCAGGC GCATGGGCAT CTGGGCGGGT TGGTCCGCCA CCCCCGGCCT CTCAGCCCCT
58921 GGACGAGCAC GAGGCGGCAG GTGCGCACTG TTCCTTCTGC ACGCTTGGCG GCCGCCCACC
58981 CCTGCCTCCC GGCCCACCGC GGCCCAGGCC TGCAGCCCGT CTGTCCGACC CCACACTCTG
59041 CTGGCTGAGG CCCCTCCCAT GTTGGCAGAG ATGGCGCTCA GAGATTATTT GCTGGGGTGA
59101 CGGGTGGACA CCCCCACTTG GCCCTCCACC CTCTGCACTG CCCACAGCCC AGACCTCCAG
59161 CCTCTCTGGG CCTCACCACA CCTCCCTCCC TGGCTCCAGG GTGGTGGCCC GAGGGACTAG
59221 GGGTCGCACT CTGCTGCAAG TTTAGCCCCA CTTCCTGGCA CTTGTCCCCA AGCTGTGCCA
59281 GGTGTGCAGG TAGGGGCTGG GGTCCCAAGC AGAGGGCACC GCTGGGAGGG CTCAGAGACC
59341 CCCACATCTC CCACGGTTGA AGGGTGGTGC CCGGCGTGGT AGGGAGCGGG CTCAGAGACC
59401 CCCATATCTC CTACAGTTGA AGGGTGGTGC CCGGCGTGGT GGGGAGCGAG CTCAGAGACC
59461 CCCACATCTC CCACGGTTGA AGGGTGGTGC CCGGCGTGGT GGGGAAGGGG CTTCAAAGGA
59521 GAGAAGGGGT GGGGTGGGCT TGGGTCGCCC TACAGTGGGA CACCCCTGGG GGGGTCCCAC
59581 TGTGCTCTGA GCCCGAGGCG CCCAGGTTGG CAGCAGCTCC GAGGGCGAGC TCTGTCACGC
59641 CAGCGTCCAC CCCAGGGGGC TCCCGGCTGT GGAGGAGGGT CGGGCCAGCT TGGAGAGGAA
59701 GGAGGAGCAC GGAACACAGA GGCCCTTGGG CTGGTGCCTT GGTCGGCAAG AGGGGTCTCC
59761 CCAGGTCTCA GGCTGCGGCC ACAGAGGGAC CCCTTGGGGC TGGCTCCTTC TGGGGGCCTG
59821 GGGAGGGACT GATGGTCACC TGCAGCAGCC GCAGGGCAGG CGGTTGGCAG AGGATCCAGG
59881 ATGTCAGTCC CTCCTGGATC CTGCCCGAGC TGGGTAGGGG AGCGCGGGGG AGAGGGCAGG
59941 CTGCCTGCGG CGGTTCTGAG CCCAGGATGG CCGTACACCT TCCCCCCTCT TCCCCCCACG
60001 GCGGCCACAC CAGGGGAGGC AGCGTCCCCC ACTTCTGTAG ACTAGGAAAC GAGGCCCAGG
60061 GCCCAGCAGA GACTGTGCCT CTGTCCCCCA CACTCCAGAG ACCTCTCAGG ACTCAGGACC
60121 CCACTCGGGG AGGGAGAGCG CCAGGGCTGG GCACCAGCAC ACCTGCCCAC TGTTCCCGCG
60181 GGAGTGCCGG ACCCCACTCC AGCTGAGCGT CCTCCCCCGC TGGCTGCTGT GCCAGCTCAC
60241 AGGAGGGCGT CCCTGCCCAG GCCCCATGGG GTCCCTGACC CCAATTGGCA GAGCCCAGCA
60301 CCGCGGCTGC TGGGGGGACC CGGGCCTGGC AGCGGGGGCT GGTCACGGAG CCGAGCTGGG
60361 GCCTCCCGTC CATCAGCGTC CATGTGGGCC AGCCGAGTTG TCGAGGCGAG AAGGCCCAGA
60421 GACCCCCGCA CACAGGGCCC CTCTGAGGGC ACCGCAGTGT CTGGCGCCCC TCGACCTCAC
60481 TCACCAGCGC TGAGCAGGGC TCCGCAGCAG GACAGCAGCA GCCACCGCTG GACCATGGTG
60541 CACAGTGGAG AGGAGCTCGC GCTGGGCCCG GCAGGCCTGC TGCTGCCATC CATGCGGCTC
60601 CAACGGCCGG TCCTGGGTGC CTTATATAGG CTGGCGGGCC CTCCCCCGCC GCACCTGCCT
60661 GCGCCCCGCG GTCCAGCCCC TTAATCACCA CTGCCGGCGG GCGCCGCGGT GGCCAAACAG
60721 GATCTGGGCC TGCTTTATCA GGACTCGGCT TTCTTTGGAA AATCCTGCAG GCAGCGGCCC
60781 CATTATCACC CATTCCCAGC GGGGGGCTGA CGCACGCACG CCCCCAAAGG TCCTGCAAAC
60841 ACCCCCTGCA TGGGGGCCAT CCTGGCCCGG GCCCTCCCCG CTGCTGGGTC AACGTGGCAC
60901 TGTCAGCCAC ACGCCTGGTG GCCGGGATGG ACCTGTTGGG GGAGGTCGGG GGCCCTGGGG
60961 CACCTGCTCT GCCCCTTCCG GGGAGCCCAG TGCTGACCCA GCTTGGGGGG AACCCTGCTG
61021 CACCTGAATC CCAGGACCCC CTGAGGGGCA CTGGGCCCAC CCCTCACCCT GAATCCCAGC
61081 ACCCCCTGAA GGGCCCTGGG CCCACCCCTG AGTTCCCTGC CCAACAGGAA AGATCACCCA
61141 GCGGGAGGGA CCTTGCCAGG GGGTGTTGGG GAGCAGGGGC TCCGCCGTCT GGGACAGGCA
61201 GCGGCCTTGG GGCTTAGACA GGCTGCCCTG GAGGCCTGAG GAGCTACCGT GTCTCTGCAG
61261 CCCGCCAGGT CCCAGGCTTG CTCCTGAAGA CCCCTCCACA GCTGTTTTCT TCCCCCTTCG
61321 AGCGCTGTTG GCAGCCATCT TGAGGACAGG AACTCGGGGG CTGCTCGTCT GCTGGGGCAT
61381 CTCTGCGGGG CTGTGGGGTC GCCGGTATGC TCCTGACCCC TTCAGGCAGA GGGGCAGGGA
61441 CTGGGATGAC CAGGGGGCCT TGCCTGTGGA GGCACGTGCC ACGTCTGAGG GGCCGGGGAC
61501 AGGGCAAGGT CTGGTGACAT CGGGGTGGTG CCGGTGGCTG GCGGGGGGGA GGGAGGTGGA
61561 GGTCTGAGTG GTCACCAGGA TTGCCCCAGC TTCGTTTGGC AGCCCAGGGA CCCCTCCCAA
61621 AGGGGATTTT GGTCCCCGAC TCCGACAGGA GCCCTCGCTG GCCTCGGGGC TGCTCCCACC
61681 ACACGTCAGC ATCCCGGCCT GAGCCAGGAG GGACTCCGAG GCCGCCTGGT CTCCCTGGGC
61741 CGGGCTCCCC ACATGGGGCT GCACCCCCAT CCCACAGGTG AGCTGAGCCT GCTGGCTGGG
61801 ACCCGGGCCT GCCACCTCTG TCCCACCTGT GCCCACACGC CCGGGAGCTG CCCCACCTGC
61861 CCCCGGAAGA GGCCCCAGGG CCACGCGTGC CATACACAGT AGGTGTCGCG TGTTTCACTT
61921 AAATCAGCTT CAGTTTTGTG GAACGTTGCT TGAAGTTACA ATAAGTTGCT TTTGTAGGAA
61981 ATTGCAGTAC GTGTGCTGCC CCCCACCAGG AGCCCCACAC CAGGCATTCT GGGTGTTCTA
62041 CTCTCATCTG TTCTTCTTTT TAATTTTTCG GTTTTGTAGG GACGAGGTCT CACTTTGTTG

62101 TCCAGGCTGG CCTTGAACTC CCGGACTCAA GAGATCCTCC TGCCTCAGCC TCCCAGAGTG
62161 CTGAGATGAC AGGCGTGAGC CACCGTGTCC AGCCTTGGCT GTCCCTTCTT AACACGCTCA
62221 GTGGTCCCTG TGGTTCAGGT GAGAAGCTGA GGCACAGGAG TGAGGCCCTT GCCCTGTCAT
62281 GCTGAGAGGT TGGATGGACG CAGGGCTCAC AGCTGGCCCT CGTTCTGAGC TGGGGCCTCT
62341 CAGCCCGGGC TCGAGGTGGG GACGATGAGG GGCAGAGATC TTAGGGCCTC CTTCCCTGGG
62401 TGGGTCACTG CACCAGGGCA GCTGGTGCCA AAAGGGACCT CATGGGCAGG TGTCATGGCT
62461 GGGAGCCCTG TGGCCATGTC AGCCCGGCGG TTGTGCTTGT CTGACCACCT GTCTGGGCTT
62521 GTGAGAACCG AGCTCAGGTT CTCAGGAGGG ATCCTATGAA GCTTTGAATG TAGGGGCAGG
62581 GGTGGGTTGG GGGTGAGAGC CAGTGGACAC TGGTTCCCCG GCCAGGCCTG TAGGGCTTCG
62641 GGTCCTGGCC ACTGAGGCTT CCATTTCCCA GCTGTAAAGT GGCAGAGACC CCTGACCTTG
62701 AAGGTTATGG CTGTGGCGTT CAGCATGTGA GTCCCCAGCG TCCTCTAGAA TTGGGGGATC
62761 CCTGCAACTT GCACAGGTGT GGGGCGGGTG GGGGCTGGAT ACCACCAGCC TGCAATGGGG
62821 CTGCCCACTG GGCCTGTGGT GAAGATGCCC GCTTGCAGGT GTGTGGTGAT TGTGTATGTG
62881 TGTGTGCGCA TATGCATATG TGTGCGTGCA CGTGTACCTG TGTGTGCGTG TGTATGTGTG
62941 TGCCTGTGTG CACGTGAGTG CCTGCCTGTG TGTGTGCATG TGTGCCTGTG TGTGCACGTG
63001 TGTGCATGTG TGTGCGTGTG TGTGCACGTG CCTGTGTGTG CCTATGTGGG TGCCTGTGCC
63061 TGTGTGCCTG TGAGTGCGTG TGTTTGCGTG TGTGTGCATG TGTGTGCACG TGTACCTGTG
63121 TGTGCCTGTG TGTGTGCATG TGTGTGTATA GGTTGAATTT CTGGTGAAAG CTGTGCATCC
63181 CAGAATTTAT TGTCCCCATG ATCTCAGGGC CACTCGAGTG TGTAGAAGGC CCTGGCCCCG
63241 AGCCCTGGTG GGGGCTCCAT CGTGGCACCA TGGGGCCTCC GGAGCCTGGG GGTCTCTCTC
63301 TCTCAATGCA CTCGCTTGGT GGCAAATGGC TGGGAGCGGT GTTTCTGTCC TGGCCGGGGA
63361 GCGCTTGAAT GCACTGAGCA GTGGGGGAGA TAAGCGTGTG AGGTGTTGTT CCTGGAGCAA
63421 CCTGGTGATA GGAGCCATGT CCTGTGGGCC TGGCTGGAGA ACAGGGCCTG AGGACTGGAC
63481 AAGTGGGTGA TTGTCCCCTC TCATGCCCAG CGCTGGCCCC GCCCTCTATG TCCGTCTAAG
63541 TGGCCTGGCC CTGAACAGCG GTCACTCCAG GCCTGGTGTG GCTGTGAAGC GGGAGTCAGG
63601 GTGGGCCCCG GGCCCCAGCT TCCCGGCTGA ACCGTTAGGG TGGGCGCTGG CTCCTAGACC
63661 CGCAGGGCTC CCTGGACAGG TGTGAGCAGA CCTTCACTTG GACTTGAGGC CTTCACCCCT
63721 GCAGGTGTCT CCAGAAGGCT CTGGAACTGA CTCGGGCCTG GCTCCCCTCA CAGGAGGCTG
63781 AGGCCCTGGG TCTGCTGGGA CTTGTGTTCA CAGAGGCGCT GGGATGGACC TGCTGGTGGC
63841 CCCCTGCCCC TCGGCCAGCC CCTTCCCTGC CCCTCGGCCA GCCCCTTCCC TGCCCCTTGG
63901 CCAGCCCCTT CCCTGCCCCT GCCCCGCTGG GCCCTGTGGT CAGGTGTGAC GTCCCCTGGA
63961 GGAGAACAGG TGGGGCTTCA CCCCCTGAGG CTTGGCAGCT GGTCCCCATC AGATCAAAGG
64021 ATTGAAAACT GTGGGTTTGG GGAGTGTTTG GGAAAGATTT CAACCCCGGG AGTGGGAATG
64081 CACCCGGGCT TGGATCGCTG CCATTCCCCA TCCGAGGCGC GGGCCCAGTG CTGGCCGGCA
64141 GAGCTCAATA TGGCCAGCCA GTGCCCAGGT GCATGCCCGA GGGGGCGCGT CACGGCTTCC
64201 TGTTGATCCG GATGCCCGAT GTCTTGGGAG GCACTGACCC TGTGCCAGGT CCTGGGGCCC
64261 ATTGCTTGGG GAGGCGGTGG GAACCTGGAG ACCCTGGGGA CAGAGTGGCT GGATGCAAGG
64321 TCCCCCAAGG GGCAGGGGGA TGGCAGGGAA GGGGCGTCAG GCCTGGGGGA GGTCACCTGC
64381 CCCACCCTCC CTCCAAGGCC CTTGTGTGGC TGGGTGGGGC TGGCCCTGGG TCCTCTCCCT
64441 CTCCCCCGTC CCTGAGCGTC ACTGGTGACC TCCCCATGGA CTTGTCCAGC TTGCCAGTAC
64501 CCTGCGGTTT TGCCTCCTGA GTATGTTGAA ACGTTCCTAG TCCAGCTCTT GCCTGCAGCC
64561 ACCCCCATCG AATCTGGCCT GCCGTGGCCC CCGCCCTGCA GCCCCGAGTG TGTGGTACCC
64621 CCTCATTCTC CCTAGCACCT CAGAGGAGGC CCTGCCTGGC TCTGACACTC CCTGACCTCT
64681 GGCCTCTATC CCCCTCCCTG GACAACTCCT GCTTCTCCAC CAGCCCTGCA GACCCCTCTG
64741 ACCCCAGCTG CTGGGGTCCA CCCTGCCGGA GGTAGAAACA GAGCCAGCTG CATGCCACAG
64801 GTGAGGGCCC TCCCCAAGGC CTGGGGAGGG GCAGCGTGAC CCCGGAAGGG CAGCTGCAAG
64861 GGCCGGGCAG TGCAGCTCAC CCGGGCGACC ACCAGCCCAC CCTCAATGAG ACCCCACAGC
64921 CCCTGTCCAG TGATGTTCAG AGGGCTAGGG CTCCCCGGGC ACCCACTGGT CTGCACTTTC
64981 AGACTTGACT GCTTGGTGCC TGGGCTGGGA TTTGGGGGCA CTGCCCTTCC CCCGTATCCC
65041 TGGGACCCCC TTTGGGAGCC GGACACTCAG GTGCGCACAG CCCCCACCCC CAGCCCAGCC
65101 CCAGCCCTGT GCTGTCCGCT GCCCACAGCC CACTGTGTGG CCTTGAGTGG ACTGAAGGTC
65161 TCGTCTGGCT GCGGGAGGGG CCCTGGTAGC TGCTGGGCCT GGAGGTGGCT GGGATGGTGG
65221 GCTGGCCCGC CTGTCCGGGG TGGGTAGAGG AGTAGCTAGC AGGAACCACT GCCTGAGGCT
65281 GGGCTCAGCC CAGGCTGGTA GCCTCATGTT CCAGAAAGGG CTGGGGAGCT CTCCACCTGC
65341 CCTCCTCCAG AGAGGGGCCC TTGTCCTTTT TGTCTACTTA TGCCCCGTGC AGGAAGCAGG
65401 TCAGGGTAGG CTGGGGCCCT CAGGAGCGGG CACGTTTCAG CCTGGGGTGG GGGCAGGATA
65461 CGGGCTGCCG GGGCCATTGA GGCAGCAGCA CAGGAGGGGT CCTGGCCAGG CCACTCCTCC
65521 CTCCAGGTGG GAGCTTAGCT GGGGCGGGAC CCCCACCTCC CCGGGTGAGC ATTGCCGGCC
65581 GGTAGCCTTG GGCAGGCACC CAGGAGAGGC CCCTGGCCTG AACTCCTCTC ACACCTGACT
65641 CTGGTACCTC TGAGCCCCAG GGTAGGTGGC TGAGTCATCT CCCCAGCTGG CACCCCAAAT
65701 ACCTTCTCCC CTTTTCCCAT CCCATGGGAG GTGGCTGTGT GCCACAGGTG AGGGTGCAGA

65761 GCCCCCAGCT GCTCCCTGCC CTGAGAGTTT CGCCCCCTGG CATGGGGCCC TGGGCAGACC
65821 CTCTCAGGAC AGAGCCTTCC TTGCAGGCCA GCCCTGCCAG GCCCTCACAG TGGGGGTAGC
65881 TGTGCCCCAC CAGCCCCGAG GGGAAGACCA GGATCCTGAG GGCTGGGGTT CACCTCCGAT
65941 GTCCTGGGGG AGATGTCCAT GCGTTGCCAA CTCCAGGCCT GAACCCAAGG CGCCCTCATC
66001 TGAGCACAGG GAGAGGCTGC CGGGACGGCT GGGTGTCTGG CTGACTGCCC CGCCCGGGCT
66061 CTGGAGCTGA TAACCAGCGG ATGCGGAGTG AAATGCAAAC AGATAAGGCT GTTGGGAACT
66121 AGGCCCCCTG GACTGCTCCG GGCGGGCAGC CGTGGGGCAC CACACTGCCC AGGGCCAGGG
66181 GTTATTGGGC CGCCTTTTGC CCTCAGTGAG CGCAGCTGGA TGGGGAGTTA GCAAGGGCTT
66241 GCTCTCCGAG GGTGGCTGGT GAGGGGGCTT TGAGGAGGGT GGGGGGACCC TGGGCTGCCG
66301 ACTCAGACTC TGGAGGCTGC TGGTCAAGTG GGGGTGGAGG TCAAGCTGGA GCTGAGGCTC
66361 ATGAGACCAC CTCTCTCCCA GGAATCCTCA TCCCTCTCAT GGGGGTGACA TTTAGGACTC
66421 CCTTTGGGGA GCCCGGCAGG GCCCCAAAAG CACAAGTCAA ATGGCCTCTG AGGGCAGCTG
66481 CAGCTCACAG CAAGGGGGCC CTGGGCAGCC CAGGGTGAGA TGAAGGGCTT CCAGGGCTCC
66541 AGGTGAAGAG CAGGACTGGG TGGGGGGTGG GGCCAGGTCA CCAGGAGGTG TCGGAGACCA
66601 GGCTGGAAGG TGACCTGGCC TAGAGTAAGC TGGGAGAGGT GGGTAAACTG AGGACCAGAG
66661 CTGGGGGTGG GGAAGGGACC CTGAGCCAGC CTGGGGGGCG GTGGTGCTGT GTGCTGGCGT
66721 GGAGTCTGGG CAGGTGCCCC GGCAGAGGTG CCTCTGCCAG CAATGGCGTT GGGGCCACAG
66781 GTCCTGCAGA GAATTTGAAG AATTTGTTTT TTTCCCCAGA AAAGCTCCAG GTATTGCAGC
66841 CAGACCTGAG TCTCTCTCAG GAATCACATC CAGGGCCCAT TCCAGGCAGT TGGACGTGCA
66901 GTGGGGGCAG CCACTGAGGC CCAGGCAAGA AGGGGTGACG GGGCAGGTCC TGGTCCAGAG
66961 GGTTGTGGGG GATGCTGGCA TGGCTGGCAT GGCTGGCATG GCCTGCAGAG AGTCGAGGAG
67021 CGCCCAGGCT CACCACAGCT GGTTCTTCGG GTGTTGTTCA GGGGAAACAC AGTGGAGGCC
67081 TGACGGGAAC AGGTGGGTTT CCCGGCCACT CACACCAGGT GCCGGCCCAA GGGAGCCTTG
67141 GGCCTGATCT GCTGCCCAGC GGCCGTGAGT GCTGTGTGGC CCGTGGGAGG CCCCGTGCAT
67201 ATGGCCTGCA GAGTCAGGTG TGGGGGGCCC AGAACACGTA GGCCTGGCAG AAGCCCAGGT
67261 GGCACAGACG TGGCCCTACA GCCGGGCTCG GGGCCCCGGG AAGCCAGAGG CTCAGGCCCT
67321 CCCAGCTGGC ATCAGGGCTG ATCGGGGCTG AGACCGGTCA TCGGAGCAGC CCATGTCCAC
67381 TGTCCCCTGT CTCAGCAGCT CCCCGGGGGT CCCCCAAACC CCAACCCTCC CCCAGATAGG
67441 AGCACAAGCA GGGTGGCCAA GGAGCACCCC CCAGCTTACC CACGGACCCT GGCTCAAGAC
67501 TGGTGGCCAA GGTGCACCCT CGCTGTACTA GGCAGTGGCC TCCAGTCCTG ATGCCACCGA
67561 CTCTTGGGGG CATGGCCAGT GCCCGAGGCG GATGCGTGCG CACACATGCT CCCAGGAGGT
67621 GTTTGTAACA ACCCCAAACT GGAAATGACC CAGATGTCCA TCAGAGCGCA GCCTGTTCAC
67681 CTGCTCTTCC AGCTTCAGCA ATTACAAACA AAGCAGTTAG AACACGTGTG TCCGTGTCTC
67741 AGAGGAGCAT GTGCTGCCAC TTCTCCCGGC GTGGACTGGC TGGATCACGT GGTTGGCGTC
67801 TGAATTTTTC AGAAACGGCC AAACTGTTTT CCGGCATGGC TGTGCCATTG TGCCTCCCAC
67861 CCGTGATGTG AGTTCCAGCC CCTCCTCACC CTCCTGTATG CTTGACGTCG TCCGTTTTTA
67921 AAATTTTAGC CATTCTAACA GGTGTGCAGT GCTATCTGAC TGTGGTTTTC ATTTGAATTT
67981 TCCTAACGAC TAATGGCGTT GAGCATCTTT CCATGCGTTT ATTTGCCCTC TGTGTATCTT
68041 CTTTGATGAA GTGTCTGTTC TAACGTTTTG TCCATCCATT TTAAAATTGG GTTGCTTCAG
68101 TTTTGAGAGT TCTTTATATA TTCTGCATTC GAGTCCTTCA CTAAAAATAC ATACTTGGCA
68161 AAGATTTTTC TCCCAGTCAG GGCTTGTCTT TTTATTCTCT TAATAGTGTC AGAAGATACT
68221 ATTAAGTGTC AAAACTTCTG TTCAAAAGAG CGGAAGTTCA TTCTTTCCCT CCCTCCCTCC
68281 TTTCTTGCTT TCTTCTCTGT TCCTTCCTTC CTTCCTTCCT TCCTTCCTTC CTTCCTTCCT
68341 TCCTTCCTTC CTTCCTTCCT TCCTTCTTTC CCTCCCTCCC TCCCTCCCTT CCTTCCCTCT
68401 CTCTCTCTCT CTCTCGATGG AGTCTCGTGC TCTGCCGCCC AGGCTGGAGT GCAATGGTGC
68461 AATCTCGGCT CACTGCAACC TCTGCCTTTC CAGTTCTGGC AATTCTCCTG CCTCAGCCTC
68521 CGGAGTAGTT GGGACTACAG GTGAGTGCCA CCCCGCCCGG CTAGTTTTTG TATTTTTGGT
68581 AGAGATGGGG TTTCACCATG ATGGTCAGGC TGGTTTCAAG CTCCTGACCT CAGGTGATCC
68641 ACCCACCTCG GCCTCCCAAA TTGCTGGGAT TACAGGCGTG CGCCACCTAG ACTGGCCGAA
68701 GTTCTTAATT TTGGCAAAGC CCAATGTATT AATTTTTACT TTACACATCA TGCTTTTAGT
68761 GTTGTGTCTA AGAAATCTTT GCCTAATCCA AGGTCATGAT TTTATCATTA TGAAAGGATC
68821 TTCTTTATTG CCAGTTCCTT GCTCTAAAGT CTACCTTTGT ATTAATATTG CCACTTGATC
68881 TTTCTTTCTG TGTTAGCACG GTGTGTTTTT TCCTCTTCCT TTACTTTCAA TAACTAATGT
68941 GTGTCTTTTA CTTACAGTGA GATTCTCTGG GCAGCTTATA GAGTGATCTT GCTTTTTATT
69001 CTAGTCTGAC AATCCACATC TTTTTATTGG GGGTGTTTAG ACTATTACAT TTAATGTAAT
69061 TAGTGATGTG GCCAGGTGAA AAGCTACCAT CTTGCTACTT GTTTTCTGTT CCTTCCATCT
69121 GTTCTTTGTC TTTCTTTTCT GCCTTCTTTT GGATTGACTA TTTTTCCACA GTTCAGTTTT
69181 ATCTCCTTGT TTGCTTATCT GCCATAACTT TTTATTATTT TAGTAGTTGT CTTAGAGTTT
69241 ATAGTATGTC TACAACCTAC TGTAGTCTCC TTTCCAAAGA TATTATCACA TTCCATATAT
69301 AGAAGAAGAA CCTTCCAGGC TGGGCACGGC GGCTTACGCC TGTAATTCCA ACACTATGGG
69361 AGGCCAAGGT GGGAGGATCA CCTGAGGTCA GGAGTTCGAG ACCAGCCTGG CCAACGTGGT

69421 GAAACCCCAT CTCTACTAAA AATACAAAAA TTAGCTGGGT GTGGTGGTGG GCACCGGTAA
69481 TCCCAGCTAC TCGGGAGGCC CAGGCAGGAG AATTGCTTGA ACCCGGGAGA CAGAGGCTGC
69541 AGTGAGACAA GATCGTGCCA CTGCATTCCA GCCTGGGTGA CAGAACAAGA CTCCATCTCA
69601 AAAAAAAAAA AAAAAAAGGT GCCTCACAGC TCCCTTCTCT TGGGTTGTGC AATTCTTGTC
69661 ACCCATTTTG TGTCCACACA CGATTCACAC CGCACTGCAT TGTTGGTATT TTTGTTTAAT
69721 TAATTATTTT TTAAAGAGGC TTAAATAGTA AAAAAACTTC TATATTTACC CATACAATTA
69781 CTATTTCAAA TGCATTATTC CATCGTGTAG ATCCATATTT CTTTTTTCTT TTCTTTTTTT
69841 TTTTGAGATG GAGTCTCGCT CTGTCACCCA GGCTGGAGTG CAGTGGCGTG ATCTTGGCTC
69901 CCTGCAACCT CCACCTCCCG GGTTCAAGTG ATTCTCCTGC CTCAGCCTCC CAAGTAGCTG
69961 TGACCTCAGG CACCCACCAC AACGCCTGGC TAATTTTTGT GTTTTTAGTA GAGATGGGGT
70021 TTTACCATGT TGGTCAGGCT GGTCTTGAAC TCCTGACCTC AAGTGATCTG CCTGCCTCTG
70081 CCTCCCAAAG TGCTGGGATG ACAGGCCTGA GCCACCGAGC CCGGCCGTGG AGCCACATTT
70141 CTATCTGATT TCCTTTTCCA CCCGCCTGAA GGGCTTTCTT TAACATTTCT TAATGGACAC
70201 TGCAATGGTT ATTTGTCTGA AAATATCTTC CTTTTGCCTT TGTTTTTGAA AGATGTTTTT
70261 CTAGGATGAC AGCTTCTTCT TCCGTTTGTA CTGTAAAGGT GCTGCTCTAG TTTTCTTACT
70321 GGCACAAGTT CCCCCGCCAA AAAAATCTGT TGTTATTTTA ATATGTTTCC TCTTTGTAAC
70381 ACGCCCCCAC CCCACAACTT AAGATTTTCT CTTTTACACT GATTCTGTAT GATTAGGATT
70441 TGGTGTCATT TCCTTCATGT TTCTTGTACT TTGTACTCAC TGAGCTTCTT GTATCTCTGG
70501 GTTTTAGTTT TCATTAATTT TTTTTTTTTT TTTTTGAAAC AGAGTCTTGC TCTGTTGCCC
70561 AGGCTGGAGT GCAATGGCGC AATCTCGGCT CGCCGCAACC TCAAACTCTG GGGCTCAAGC
70621 AGTCCTTCCG CCTCAGCCTC CCGAGTAGCT GGGATTACAG GCGCCTGCTA CCACACCCAG
70681 CTAATTTTTG TATTTTTAGT AGAGATGGGG TTTCACCATG TTGGCTGGGC TGGTTTCAAG
70741 CTCCTGACTT CAGGTGATCC ACCTGCTTTG GCCTCCCAAA TTGCTGGGAT TACAGGCGTG
70801 AGCCACCGCA CCCAGCCAAG TTTTCCTCAG ATTTGAACAA ATTATGGCCA TGATTTCTTC
70861 AATTTTTCCT GTCCTCCTCT CCCTTCGTGG GCTCCGTTTA CAGACGTGTC AGGCTGCGCG
70921 AAGCTTTGCC ACCTACTGCT CGCTAAGGAG CTGTTTATTT TTTGGATTCT TTTGTCTTTC
70981 TGTGTCTCTA TTATGTCTTC AAGTTCACCG CTGTCCTCTT CTGCATTGTC GCATCCGTTG
71041 TTAATCCTGT CCAGTGTGTT TCATCTCACA CATAGTCATT TTCATCTTTA GATGTTTAAT
71101 TTGGATCCTT TTTTATATGT TTCATGTTTT TTACTAAATA CGTTCAATTT TCAGCATAGC
71161 ATTTTGATGA CATGGAATAC TGTCGTAACG CCTGTTTTAA TGTCTTTGTC GGCTAATTCC
71221 AACATCTGTG TTGGTTTTGT GGTTTGGATT AGTTGAATTT TCTCCTGATT ATGGGTGGAA
71281 TTATCCCCTT TCTTTACCTG CCTGGTACTC TTTGAATGGA GGTTGGACTT CGTGAATTTT
71341 ACCTCGTGGG CTGCTGGATA CTTTTGTGTC CTGGGGCTTT GTTCTGGGAC ATAATTAAGG
71401 GATTGGAGAG AGTTTGGGCC TCCTGGGCCT CGTGTGGCTC GTAGGTGGGC TCAGAGCAGT
71461 GCTGAGTCCA GGGCAAACTA CGCCTCACCA TTGAGGCAAG ACCCTGAGGG GCACTCTGCC
71521 CACCGCACCG TGAACTTTGA GTGGCGAGGT TTCTCGGTGG TGCTGGCGGG AGCAGTGCTT
71581 TTCCCAGCCT CTGTGAGCTC CGCTGCTCTT CCTTCCAATC CTTCGGGTCG TTGCTCGCCA
71641 GCCTTAGTCT CCTCCCAGGC ACGTGCTGAG CGGGTTCCCT GCCAAACGCT CAAGACGGAC
71701 CTTCTGCAGG TCTCCAGGCT TTGCTCTGGG CAGCTCTCTC CCTGCCAGTG TCCTGTGCAC
71761 TCCAGCTGCC GTGGTCTCCC CGGCCTCTCA TCTGAGCTCT TCCTGAGTTC TCCTTCCTGG
71821 CCCCTTGTCC TGGAAACCCT CAGGCGGTGA CCTGGGCAGT TTAGGGCTGT TTCCTGTCTG
71881 CCATGGACTG AACGTTTGTG TCTTCCCCAC CCTCCGGTTC CTGTGTTGAA ATCCTGGTGC
71941 CCAAGGTGAC GATATTAGGA GGTGGGGACT TGGGGAGGTG GCAGAGCCCT CAAGAATGGG
72001 GTTAGTGTCC TTACAGGAGA GATCCCAGAG CCGGGCGCGG TGGCTCACGC CTGTAATCCC
72061 AGCACTTTGG GAGGCCGAGG CAGGTGGATC ACCTGAGTTC AGGAGTTCGA GATGAGCCTG
72121 GCCAGCATGG AGAAACCCTG TCTCTACTAA AAATATGAAA ATTAGCTGGG CATGGTGGTG
72181 GGCGCCTGTA ATCTCGGCTA CTCGGGAGGC TGAGGCGGAG AATCACTCGA ACCTGGGAAG
72241 TGAGCTTGCA GTGAACCGAG ATTGTGCTAC TGCACTCCAG CCTGGGTGAC AGAGTGAGAC
72301 CCTGTCTCGA AAAAAAAAAA AAAGAGACCT CAGAGAGCTC TCCCACCCCT TCTACCAGAG
72361 GCGAAGGGGA CCCAGTGGGA AGGTGCCGTC CGTGAACCAG GGAGTGGCCT CACCAGACAT
72421 CGAATCTGCC AGAGTGTGGA TCTTGAACGT CCCGTCCTCT GCAGCCGTGG AGATGCGTGT
72481 CTGTTGCTTT TATGCCGCTC AGGCCATGGC GTTTCCGGAT AGCAGCCAGG TTGGAGGGAC
72541 CCGCTGTCTC TCTGGGACCA CTCTCTTCGT CGCCTGATGT CCACCGTCTT GACAGATGTT
72601 GTTTCCCATA TTTCCTGGAG TTTTTTAGTT GCTAAATCCT GGCCTCTGCT ACTCTCTTTG
72661 CTAAAAATGC TAGTCCTTGA TGAAATGGAA TCTTGATACT TTAATACAGA GTGGAACATA
72721 GTCACTAAAT AAAACCTGGA AAAATGTAGA AGAGTAGCAG GAAGAGAACA CACCCCAGCC
72781 CCGCGGTCCA CCCACCCGCA GTCCCTATCG CCACCTCCTG GCTCTGGTTT CCCTGCTCGT
72841 GGGGCTGGGC AGCATTGCTG CAACTCTTGA TTCGCTTTCC TGGGGCCACC ATAACCAAGC
72901 AGCACAACCC GGGAGATGGC TCAGAGTCTC TCTGCGTCCT GGGAGCCTGA GATCCAGGAG
72961 TGGGTGGGTT GGAGAATGGT TCTCCCTGGG TCACTCTGGA GGACCTCTTA AGGTGGACTT
73021 CTTAAGGGAC CCCCGGCTCC CACCTTGCCA AAGAGCCTCC GAGCCACACC TGCCTCCCGC

73081 AGGCCTCCAG TTGGTGCCCA CATGCAGCCC CCGCCGTGGG GTCACCCTGG GGTAAGGGTC
73141 TCTCCTCCCC TGCTAACCTT CTGCTGGGTC TTGGCATCCC TGGGATGAAC CCTCCTGGCC
73201 TGGACCTTGC CGGCCCACAT CAACCCCCAG CCCTCTGCTC CTTGGCCTCA CCCACAGCCT
73261 GCAGAATGTA GGTGAGTTTC CTGATGATAA TATAATAATA GCAATGATGA TGATGATGAT
73321 GATGATGGCA ATAACAATGA TGACAGTCAA CACTGCCGCG TGCCGGGCTC TGGCCCTTTG
73381 CATGTCTGGG CCGGCTGTAC AAGGCACTGC TGGGGCTCCC GGGCTGGGAG CCAGGGACAG
73441 GCACCGTCAC AGCAGCCAAG GGGCCCCCCT ACCACCCCAG CACCTGCTGG CCCTGAACCA
73501 GCTGCTCCTT GAGAGCTTTG ACAGACTCTT CCGCTTGGCA GCATTTTAAT CTGCTGGTGC
73561 AAGAGCCTTG TCGCTTCCTC AAAGCCTGTG CCCATGGCCG TGGGCAGCTG CCTGTCCCTG
73621 TATAGGCAGA GCTGCGTCAT GCCCTGTGGC CCCAGGTCTG GCTCTGGGGT TCTGGCTGGG
73681 CGGGGCCTGG AACCTTCTGG GAGTCACCAT TGACTGGCTG CAGCCACCGG CTTCCCAGCA
73741 AGGATGTCCT CTTCTGTAGG AAAATGGGGA CGTCGGGAGC ATTCTCTGGA CAGCAGGAGA
73801 TGCATGTCGA CAGGCTGGCC CTGCCTCTTC CCACCCCAGA GTGGTGAGTG GGGATTCTGG
73861 GGACTTCCCT GGCCCCCATG GGCCTCGCGT GTCCTGGTGC CCATGTGTCT CCAGTCCCAG
73921 GGTCGGCCAC GGTGTGGACC CCTCCACTTT CCTCCAGGCC TGAGGTGGGG CCCGGGCGGC
73981 CTGTCAAAGA AGAGCCCAGC CCAGACCCTC CCCAGATCCC TGGGGGAAGG AGGCCACCAG
74041 GCACCCTGCC TTCCTGCTGC CTGTCAGGGT GAATCTCAGG GGACCCTGGT GCAGGAGGGG
74101 CTGGCTCTGA GCTGGACAGG CCTCTGGGGT TGCCCTGGTG GCCTTCCCTC TCTCTGGGTC
74161 AGACGTGCCC AGGCAGGGAG GGTCCAGGCC ATGACAGAGC CGGGCCAGCT GGGGACAGGG
74221 CATCCCCCAG TGCAATCCTT GTAGCGAACG GGTCCTAGAG TGCAGCTGGC GGGACACTCC
74281 CGCCATCACT GGATTTACTG CAGGTGGAGA CGGGTTCTGT ACCCACGTAG ATGAGGAAAC
74341 AGGCACAGAG AGGGGGCAGC CTGCCCAAGG TCACCGGGGA GGGAGTGGAG AGGCTGGATG
74401 CCCTTGGGCC CAGACTGCAG CCTCCCCTCC CCGATGTGCT GCGGCCCCAG TGTCCTGGGC
74461 CCCATGTGGC GCCTGAGCCC ACAGGTGTCA CCAAAGGTCT GAGTTGCGGG GACAAAGGCG
74521 GGGACAGGCC CGAGGTGCGG GGACAAGGGC GGGGATGGGC CTGAGGTGCA GGGACAGAGG
74581 CGGGGACAGG CAACCCCTGC GGTTCGAGGG GTAGGTGGCC ACACGTCAGT CCCTGGGAGG
74641 GCCAGGCAGG AGGTGGGCCC AGCGGGGCTG GGAAATGAGG GGCCAGTGCC CCCACGCTCA
74701 CTGCAGGGGA TGTGAGAGGG CGAGTGGTCG CTCAGCCACA CCCACACCCG TGTGTACAGG
74761 ACCCACGCCC GCCCGCCTTG CTGTCCCGCC CGTGCACGGC TCACACAGCT TCTCCCATTA
74821 TTCAGGCGCT GCCGGGCCCT GCAGGACTCA GATCGTCCCT GTGCCACTTG GCAGGGTCAC
74881 CCTCACGCCT GCCCGGTGGC CCCACCTGCC CCACCTGCCC AGCTTCTCTC CCTTCTCAGT
74941 GCTGTCTGTC CCAGAGCCTC CCATCAATGG GGCCAGAGGG CTCTGGCCCG GCTGTGGGCT
75001 GACTGTTCTC CCCAAGATGC CTGAGAGCCA CCCGGGCACC GGACTTGCCA ACATGCAGGC
75061 ACACGCAGGC ACACACCTGG GCCACACGGC CCTGAGCACA CGCCTTGCCC AGCCCTCCTC
75121 CGGGCTGTGG TGCGGGTGCC AGGTGCCACC TGCTGGCGGG CTACAGCATC ACGGCTCTCG
75181 CCCTCCGGAA CCTTCCATGG TGCCACACCA GCCTCTGGTC TGGCTGCAGA CGGCTTGGGC
75241 TGGGCAGGGA AGCTTCCACT GTCGGGGAAC CTGGGACCAC AGTCTTTGCT CCAAGCGCCC
75301 TGGGCGGGTA GAGGCCAGGT CCGGTATGAG GAGCCCTGGT TCCCTGAGGT AGACAGAGCC
75361 AGGCAGACGT TGAGGAGACA TGGGGGCTGT AGCCCTGGCC CCACTGCCCG ACTCTCTCTC
75421 TGCATTTCGG GAGCTGGCAA GATCCAAGGG GGTCCCCAAG GCCCTGAGGC TGCACTGAGC
75481 ACCCCTTTCC TGCTCATTTT CAGATGCAGC GGTGTCCTGG ACCCCTGGGG AGAGGTGACC
75541 CCCCCAGCAG GAAGCTGGGC CTGGTTTCTG TCCCTCTGCA GCCACAAGGC CTGGCTAGGA
75601 TGCTGGGAGC ACCACACCCT GGAGACTCAG CTCACCAAGG ACTCAGGGGC GGGGGCTCCC
75661 CTGGCACCTG GGAAGCTGGG CCCCCGGCCC CCTGGACTCC CACCCAGACA CCATCTCAAC
75721 CCAGGTCTGT CTGCTCTCCC CGTCACTGCC CTGGGTTCTC CACTCCTGGA CCCGACCCTG
75781 GTCCTGGGGC AGTGTCCCAG CTGCTGTGTC CCAGGCCCCC CATCCACTGC CCTTGGCCCA
75841 CCTTCTATGT GGCCCATTAG AGGGGTGTGG GGGCTGCAAC CTGCTTCTGC TGAACCCCTA
75901 GGCCTGCCAG GGGGCATCAG GATTCTGCAC AGAGAGGCAG GGGACCCGGC CAGACATGCA
75961 GGTACCCACG TACCTAATTC TGGGCCTTGG GGCAAGGACT GCGTATTTAT CCTGGCCCAG
76021 GCAAGTCTGG GGGTGTCTGA GGGGTGGTGA GACGTCTGGG CAGATTGTCC CTGCCCTGCT
76081 CTGCTCTGCC CCACCCTGGC CAGCCCTCCT GGCTTCTCAA GGTGCTCCTG GCCACGATGG
76141 CACAGTCCCC CAGGCCCAAA ATGTGCCCCC ATGCTGCCCG AATGCCAGTG CGGGGGCTGG
76201 TAGCAACCAA GGCCATGCCC TCCCCTCCCC CTCCTGGGGC ACCTGCCCCC ACGCTGTGGC
76261 CATGGCTCCC TGGCTCCACC AGCTTCTTGT GACCCCTGAG GCTCCCCAGC TCCTGGCCCC
76321 CCATGGAGAG TTCCCTCCCC ATAAGGGGGG GCCAGACACC CCCCTGTGCC CACTCCTTGG
76381 GAGACTGGCA TTAGATCCTG GCTGTGGGAC AGGCTGGGCC ACTCTGGGGG TGAGACCAGG
76441 CTTCTCCCAC CTGGGGGACC CATCCTGGCT CCGGACTTGG GCGGGTGACA GCCTGGGTTT
76501 CCTGTGTCCT CACCGCCCGC TTATCAGCAA GATGGGGATG TGGATGACAG GAGTGCTGGC
76561 CACGGAGGGC CTGGTGTGGG TGGCCGTCGG GACGAGGGTA GTGTGGGCGG CCACGGCAGT
76621 TGGTGCTACT GCTGGGAGGG GCCGAAGGTG AGGCCCTCCT GGGCTGTGGG TCCCTGTAAG
76681 CCACTGCCCC CGTGGGGTGT GCAGTCACCA AGCCGACCTG CTGCCGGGGT TCCGCCCCCT

76741 CCCTTTCCCT TCTGCTTCCC CGGCACCTGT GTCCCAAGCC TGAAGCCCTG TCCGTGTCAG
76801 AGGTGGCACC TCCCTGGGCA CGGGCACTTG GACGCTGGTG CCTCCCCTGC TGCGGACCCC
76861 TTTGCTGTGG TCAGGGCCTC CTCCCTGCAC TGGCCCCGCC TGTGACCGCA GGCCTGGCGG
76921 GGCCCTCAAT GGCCACAGAG GGCACAGGCC GGGGCCAGGA GGGCACCGTC TCCTGGGGCA
76981 CAGATATGCT GGAGTGAGCT ATGGAATTGT CCATCAGCAG CGGCAAGCTT TGCTGAGCGC
77041 CAGCTGTATG CAGAGCAAAG GCGGGCCAGC CGGGAAGGGC CAGTGAGGAC CTGGAGTCCC
77101 CAGCATCCCC AGGAGCCTGC ACCCCTACGC GGGGGACGCC TGGGCAGCTC CTCCCTCCTC
77161 CACTCCCCGC CCTCTCCTCC CCTCCTTCCT GCAGTTCCCA CAGCTGTTTG GGTGCCTCCC
77221 GTGGCGCCTG GTTGAGCTGC AGGGGGAGAT GTGTGTGGAC ACTGCAACCC TCCCGAAGCT
77281 GGGCCTGGAA GAAATGACTT CCCCAGAACC TTATCTGGGC CGGAGAGGGG CGTTGGCAGC
77341 GGGGGTCTTG CCGCCTTCCG GTCCTCTGCA TGCCAGGCAC CACCTGGGGC CGGGCCAGGG
77401 CAGGCTGCCT GGACACCATG GACCTGCCCA GCTGTAGGGG AGGTGTGTCC TGCAGCCCTA
77461 CCCGAGGCCC AGGCCTGCGT GGCTGAGAGG ACTGAGGACG GCTGACCCCC TTGGTGACTG
77521 CCTGGGCCCA GAGGGGAGTT GGGGGAGTGG TCAGCTGGGT GTGGCCAGCC CTGGGGGAAG
77581 GATCCAGGGA CTGTGTCCAC TTAGGGATAG GAGGCAGCTA GCAGAGCCCT CCCAGCTGAC
77641 CAGGGGAGGC CCTGTGGGCA CAGGAGGGGC CCCAGGTGTA GGTACAGGTG CAGGGCTGTG
77701 CGGCTCTGTG TCACCCAGGT GGAGCGTCTT GCCCCGTTTG ATGGCTGACA AAGTGCCCTT
77761 GAATGCGTCA GACCCAGCGT GGTCCCAGGG TCCTGACCCT AACATACCAC CCCAAATTAC
77821 CCTCACCCCA GCCTACCCCT GCCCTAAATT CACCCCAAGC CTCCACCCAA ACCCCTTAGT
77881 CCCAACACCT TAACCCTAGA CCCAACCCTA CACCCCAAGC CCTAATCCCT AACCGCTAGC
77941 CTCACCCTAA CCTTCCTACC GGATGCCACC TGGCAAACAT CCTCCTGGCC CCTATCTGCC
78001 CCTCCCCCGG GGATCCGGGA GGCAGTGGGG TCCCTGGGAG GCTGCTCCCT GCAGAACGCG
78061 ATGGACAGAT GCATGGGCAC CGGGCCCCAG CACCCTCTGG CTTCCTTCAT CCTCCCCAGG
78121 CACTTCCCCC GGGCCAGAGG GCAGCCTGGC AGCCCTGGAC TCCGGGAGGG CCGAGTCTGG
78181 GGAGCTGGAC GACGTCACAT TCCACTCTTG ATGATGCCGC CCCAGTCCTA CAATCTGGAC
78241 AGCAGGAGAA GCTGCCCATT TCCTCCATCT CCGGTAATGA GAAGCAAATG CTGATCCTGG
78301 CCGTCTCCTG GCTCTTGGGT CCCTAGGTTC CTGCTGCCCC TGCCAGTTCC TGGAAGATGC
78361 CAGCAGGAGG GAGGGCAGTG GAGCTGGACT TGCGCCCACA ACAGGATCTG GCCATAAACG
78421 CAGTCAGGGC GAGGCTGCGG GGTGGGTGGC TGAGCCTCCT GGTGGACTAT CTCCCCTCCC
78481 ACCCCCCGCC CTGGCCCGCA GCTGTTGGCT CTTCCTGGGA AGCCACAGCC TTTGTTTGTT
78541 CCTTGAAGGA GCTGGTGCGT TGCCTGCGAG CTCCTCTGCA GGCTTGGGGG TCGCACCTGC
78601 TGTCCACCCC CCTCGAAACC AGCTCCATCC AGCAGCAGGC TGCAAGCCTG GAGAGGCCAC
78661 CGGGGACCAG GCAGGGAAAC TGAGGTCCCA AAAAGGCAGG GAACAGGTGC AGGGGGCCTG
78721 TCTGACCTGC GCTGGGACTG GGCCAGGTGA GTCTCCTCCA GGGCCCAGCC CCATCTTGGA
78781 CAGAGGAAGT TGATAGTCAG GAAAGGCAGG AGTGGCCTCT TCTAACAATT TCAGCTCTGA
78841 AGGGGCCAAG GGTGGCGATG CTGACAGCAT TTCCTCCAGC TCCCACCTGC CTGCTCCGGG
78901 GAACTCCCTA CCCAGGCATT GATATTCACC TGGTGATACT AATAATTGTC TCAGGTGATA
78961 AATTGTCTCA GCTGCTCTCC CACATGGCCC ACAGGTCCCT GGAGGTTCCC CGGATCCTTT
79021 CAGGGAAGAC CATGGGCCCT AAACGATCTT CATAACAAAA CTGAGAAGTG GCCTGGCTTC
79081 TTCACTGCGT GCCTGTGTGC TGGGATCTAG CGTGGCCCGA CTGGGCAGCT CCTTTCCTTC
79141 CCTGACGCTG CTCCAGGGGA AAGAAGGACC AGGGTCAATC CGGGGTGCCC CAGGTGGATC
79201 AGCGAATGCC CCTGAAGCCA GGTGCCAGTT GCATCCCCGT CAGATGCCCC CACCCCAGTC
79261 TCCTGAGCCT CATTAGTCAC CCCTCATTTT CCTCTGGGGA CCCCAGGCCC CAGCTCCTGT
79321 CTGGTTAGAG GCTGAAGGGG TCTGAGCGAC ACCTACCCTA GGGGACCTCC GGAGGGGTCT
79381 TGGTGTCCTC TGACTTCTGT CCTCAGAAGG GAGCTCAGAA TGCAGCTGAG GCTTCCCCTG
79441 GGGCCCAGGG TGTCTGTGTC CGAGCCACAG CCAGAGTGTG CCCTGGAGAG TCGCTGGGAT
79501 GCACGGTGGG CTCTCGTCAT CCTGTCCCCA CTCCAGGCTC AGGCTGGAGC CGTGATGGCA
79561 CCGCTGCACT CCAACCTGGG CAACAGAGTG AGACCCTGTC TCTATAAAAT AAAAACAAAA
79621 ATAAAGAAGA GCATGTGGTT TCTGCAGTTT CCTGGCCAAA AATGCATAAC TCCAATCTCA
79681 ATCATAAGAA CACATCCAAC GTCCCCAACA AGGGGAGGTT CTACACACAC CTGCCCAGAG
79741 CCCCTCAAAA GTGTCAAGGT GTGAAAGACA TGGAGGCATG TAGAAGACGG GAGAGCCAGT
79801 GTGGCCTGTG GTATGTTCAT GACGGTCACA GGTCAGCGCC CACGGTCACT CAGCAGCCAT
79861 GCCAGGCAGG CCCCAGGGGC CCAAGAGGAA GGTGGGATGT GGCGGGATGG GGGCTCTGCA
79921 CGGCCCTGCA GCACAAGTGC CCTGCCGACT CCAGGGGCAG CAATCTGGTC CCCTCATGGT
79981 GCCATTCCCC AGGGACGGGT CCTCGCCTGC CTCGCCGGCC CTCCACAGAC AGGACAGGGA
80041 CAGGTCGTCA GCTGTCACGG AGGCCCTCGC AGTTTTCTGA ATGTGGACTT GCTGTTCCTG
80101 CCCATGGCTT CGTCCACGGA CTCGACAGCA CCCCACTGTC CATCACACCC CCACCGATGC
80161 TGCTTCCCAC AGCAGGACTC ATCCCATGCT CAGGCAGTGC TCCCAGGAGC AGCGGGTTAA
80221 TAGAGCGTGG GACGGCCCCT GAAGACCAGT TATGGCGCCG GCCAGGAGGC CCTCTGAAAG
80281 GACGGGCGCA ACCCCGCAGA ACAGACCAGG TGCTCGCCAT CCATGTCCGG TGCCGCTTCT
80341 CTGTGGCGAT GGTATCCAGG AATCACGGGT GGGAGTCACG GCCACAGTCC CTGTCGCACC

80401 AGCCGCCTGC CCACACAGCC ACCGTTCACT CTAGCTTGAA GGTCCCGCTT CCTCAGGGAG
80461 GAGCACTCCC TCCAGGGTCG AGGTGCTGGC CGTGTGGAGT TCGACGTCGG GACTGTCCCC
80521 AGGGGCCTCT CTTCCCACTG AATCGACAGA GAGAATGTGA GCTTGCTGTC CGGCGGGCGG
80581 GGCCGGGGAT CCTGTCCGTC GAGGGGACAC CGGATTGCTG CGGAGAGAGG ACTAGACCCA
80641 GAGTCCAGCC AAGACCAGAA CTGAGACCAG GCGACCAGCA GAGCCTGCAC AATAATAATG
80701 CTTCGTTTTG CAAATGTATT TTGTTCTTTT ATTGTATATA TTTATCATGT CCAATGTGAT
80761 GTTTTGAAAC GTGTTTACAT TGTGCTTAAA TCAAGCAGCA TAACACACAT GACTTCACAT
80821 ACTTCCCATT TTTTTGTGGT AAGAACACTC AAATTCTACT CGCAATGATT TTCTTTCTTT
80881 TTTTCTCTCT CTTAATGATT TTCAAGGATG CAATATGGTG TTATTAACTG TAGTCACCAT
80941 GCTGTGCAGT ACATCTCTTG AATTTATTCC TTTTGTCTAA CGGAACTTTT TTTTTCTTTT
81001 TGAGATGGAG TTTGCTCTGT CGCCAGGCTG GAGTGCAGTG GCACGATCTC GGCTTACTGT
81061 AACCTCTATC TCCCAGGTTC AAGCAACTCT CATGCCTCAG CCTCTGGAGT AGCTGGGATT
81121 ACAGGCACCC GCCACCACGC CCAGCTAATT TTTTTGTTTG TTTGTTTTTT GAACCGGAGT
81181 CTCACTCTGT TGCCAGGCTG GAGTGCCGTG GTGCCATCTT GGCTCACTGC AACCTCCGCC
81241 TCCTGGGTTC AAGCGATTCT CCTGCCTCAG CCTTCTGAGT AGCTGGGATT ACAGGCGCCT
81301 GCCACCATGC CTAGCTAATT TTTGTATTTT TAGTAGAGAT GGGGTTTCAC CATGTTGGCC
81361 AGGATGATCT TGATCTCCTG ACCTCATGAT CTGACCGCCT CAGCATCCCA AAGTGCTGGG
81421 ATTACAGGCA TGAGCCACCG CGCCTGGCCT AATTTTTGTA TTTTCAGTAG AGACGGGGGT
81481 CTCATCATGT TGGCCAGGCT GGTCTCGAAC TCCTAACCTC AGGTGATCTG CCTGCCTCTG
81541 CCTCCCAAAG TGCTGGGATT ACAGGCATGA GCCACTGTGC ACGGTCTGTA ACAGAAACTT
81601 TGTACTCTTG GACCAACCCC TCCCCAGTTC CCAGCCCCTA TGTCCCTGGG TCCTCTCTCT
81661 GCTTCTGTGA GTTCAACTTT TCTAGGCTCC ACTTATGTGT GAGATCATGC AGTGTCTGTC
81721 TTTCTGGGCC TGGCTTCCTT CATTCAGCAT GAAGTCCTGC AGTTCATCCA TGTCGGCACC
81781 AATGACACAA TTCCCTCTTC TGTGGAGGAA CAGTTCTCTG ACGTGGATGC ACCTTCCTTG
81841 GCTGCTGGAC GCTGAGGCTG GTTCCATGTC TGGCCGTAGT GGACAGCGCT GCGGCGAACA
81901 TGGGGTGCGG GTGTCTCTTC TGCGTACTGA TGTGGCCTCC GCGTGTCACG CCCGGGAGTG
81961 GGACAGCTGG ATTACGTGGT GGTTCTGTTT TCAGTTTTTT GGGAATCTCC ATGCTGTTTT
82021 CCATAACGGC CATCATCACA GACTTCTTAT CATGAAATCA TTTTAGACTC GTAGAATCCT
82081 GGTAAAAATA GTATAGAGAA TGGTGATCTG TCATCTCTGG GCAGGGCAAA GGGAGCTGGG
82141 GCGACCAGGC AGGAGGGAGG TTTGCTTTCA TGATCTGTTC TTTACCTATT CAAAAACAAT
82201 TAAAATAAAG GCCAAGGCAG ATGGATCACT TGAGGCCAGG AGTTTGAGAC CAGCCTGGCC
82261 AGCGTGGTGA AACCCCATGT CTACTAAAAA TACAAAAATT AGCCGGGTGT GGTGGGGCAC
82321 ACCTGCAATC CCAGCTACTC GCGAGGCTGA GGCAGGAGAA TTGCTTGAAC CTGGGAGGCG
82381 GAGGTTGCAG TGAGCCGAGA TCGCGCCACT GCACTCCATC CAGCCTGGGT GAAAGAGTGA
82441 GACTCTATTG CAAAAAAAAA AAAGAAAAGA AAAAGAAAAA GAAAAGAAAA ACCAGAAAAA
82501 ACACATAAAA TGCGCCATTG CACTCTAGCC TGTGTGACAG AGTGAGACTC CATTTCAAAA
82561 AAAAAAAAAG GCTGGGCACG GTGGCTCATG CCTGTAATTC CAGCACTTTG GGAGGCTGAG
82621 GCGGGTGGAT CATGAGGTCA GGAGATCGAG ACCATCCTGG CTAACACGGT GAAACCCTGT
82681 CTCTACTAAA AATACAAAAA CTTAGCCGGG CGTGGTGGCG GATGCCTGTA GTCCCAGCTA
82741 CTCAGGAGGC TGAGGCAGGA GAATGGTATG AACCCATGAG GCGGAGCTTG CAGTGAGCCG
82801 AGATTGCGCC ACTGCAGTCC AGCTTGGGTC GCAGAGACAA ACAAACAAAC AAACAAAAAA
82861 ACCCAGAAAA AAACCACAAA AAAACCCCCA TTAAAATAAT TTAAAATTTT AAAATAATGT
82921 TTAGTAAAAT AATGTTTAAT AAAAAATAAT GTTTAATGTT TAATGTGAGA AATGCTTATA
82981 TGGTAAAAAA TAAGGAAAAA GGATAATTTA AAAAAGAGTG AGAAGAAAAT AGAGTCAGAT
83041 ATTTATCTGA TCTTTAGAGA AGAGCACTTT TCTAAGCATA CAAACAAAGG AAGAAATAAT
83101 AAAGAAAAAA AGTGACAAAT GTGTTACCTC CCTAAAAACG CGTGCGTAAG AACAAGGGTT
83161 TGATCCTCAC TGACGGGGTC CCAGGTGCTG ACTCGAGTCC AGCAAGCCCC AGAGCCAGAG
83221 GCTGAGTGCA AACCACGGAG GTGACCTCCA TGGCAAGAGC GGTGTGGCGT GTCCGTCCCG
83281 CACAGACAGA AATGCGGTCC AGGGGTGTGG GAGGACCATG AAGGGGTCAC GCTGAACGTG
83341 CTGCAGGTGC CCACCAAGCA GACAGTCTGC CTTTCGAGGT GTGGTTCAGG GAGTTAGCAG
83401 GGCGCTAACC GCTGGCTGCT TCGTTGGGTG CAACATAGAC AGAAAAGGGG TCACATTAAA
83461 CTGGAAAGGC CAGACCTGGC TTGGTTTACT GTGGAGGAAG GGATGCAAAG GCCTAGGGAG
83521 ATTGGAAGGC TAGGGTAGGT TTGTCACTTA AGACCTCCTC ACCCACCAGA AGGTCCAGAA
83581 GACAGACCTT TCCCCACACT GGGAACTAGA TTAGTGAGTG GGGTAGAAAT AGGGACAGAT
83641 CCCTGGGCTG CTCTTCTCTG AGGGCCACAG ACCTCACAGT GGGAGCCACA GCCACTCAGC
83701 TGGAAAAGAG TCGCAATGGG AAGAATCGAC CCGGGACAGC CGAGCGGTGG CTTTCAGCCG
83761 TCAGAGGCGA GGGGGACCTG GGACTGCCAG GAGGTGGCTC TCAGCCGTCA GAGGCGAGGG
83821 GGACCTGGGA CTGCCAGGAG GTGGCTCTCA GCCGTCAGAG GCAAGGGGGA TGCGGGCGCC
83881 ATCATGAGTA GCAGAGGCAG AACGACAGTC AAGCTGGGCT GACCCCTTAG CCACAGCTCT
83941 CCTAGGAGGA AAACAGAAAG CCTGGGAAGA TGGTACTGGA TCTGCGGGAG GCCAAGGTCG
84001 GGGTGTCACC TGAGACCAGG AGTTCAAGAC CAGTCTGGGC AATATCGTGA GAACCCCATC

84061 TCTAAAAACT AAAAAATTAG CTGGGCTTGG TGGTGCACAC CTGTGGTCCC AGCTACTCAG
84121 GAGGCTGAGG CAGGAGGATG GCTTAAGCCC AGGTGTTCAA GGCTACAGTG AGCCAAGATT
84181 GCATCACTGC ACTCCTGCCT GGGCAACAGA GCGAGACCCT GCCTCAAAAT CAATCAATCA
84241 ATCAATAATC ATGTTCTCAG CAAATATTTA ACTTCCCCGA TATATTGTGG GGGAAAAGAG
84301 GCTGTAAAAC AATCTGTGGT AATAACCAGG AGAACTTAGG AGCACCCGTG GGTGCCCATG
84361 CGGGCAGAGA TTCACGAACA CTGTTCACCC CCATAACCAG CAAGGGTGCT GCTATCCAGA
84421 ACCCATCGCA GATGAGCAAC TGTGCCTGCC CAGGCCAGGC CACGGCACCC AGCAGCCGGG
84481 ACCCCTCCCC ACCTGAAACG ATACTGCCAC CTGGGGACCC CAGGCCGCTG ACATGGGGAC
84541 GCGCTAGAGC TTTCGAACAC GTCTCTGCAT CTGGACATGC CCTCGGTGCA CGTGTATTGC
84601 TTTAAGTTCA GAGACGTCAA ATGTCATTTA ATGAAAAGAC TCCGGCTACA TAATTGTCCC
84661 CAGGAATGCC CAGGACAATG TCAGGAATCT CCCAGACCTG CACAGAAGGT GGGAAAGATT
84721 TTGAAGGATT AAGAGGCTTC TCGGGGGAGG GGGAGGGGGA CATTTTTCTT CCACCCACGG
84781 ACAGTTTTGT GTTTTCTGAT TTCTGTAACG AGTGCCACAG CTCTGTATGG AAAACAGCAC
84841 TGCCTTTGGC GTCCCTCCCA ATTCCTGGCA CATGAGTCAA GGGCTCCCAT AGCGTCACCG
84901 TTGAGGGCAG GGCAGCCCCG GGCTCACAAA GTAAAGAAGG AAAGGGCCAG GCCTCACTGC
84961 AGGGTCACCC CGAGGCCATA AAATCCTGCA CCTTATGCCT TGGGCACCAT TTAATACAAT
85021 TAAACGGTGC AATAAAGGAA GGGAGTAATA AAGAAAGCAA ATGTCAGCCA GCGAGGACAT
85081 CCGGCACAGG CCACACCCCA GGTCCAGCCC CACTAGGGTG GAGTTTGAGG TCCCTGGGAA
85141 CCCCAGAGTC TATGCACTGC TGTGCCCAGC TAATTTTTTA ACATTTCTGC AGAGACAAGG
85201 TCTCCCTAGA TTGCCCAGGC TGGTCTCGAA CTCCTGGCCT CAAGCGGTTT TCCCAATGTG
85261 GCCTCTCCAA ATGCTGGGAT TATAGGTGTG AGCCAGTGGG CCCGGCCACG TGCTCTTATG
85321 TAGCTTATCA GAGTGTATGA CGCTGCCTGT CCCATTGCTG GGGATGTTAA CCTTGAACCC
85381 TTGGTTCAGG TGGCGTCTGC CAGGTCTCTG CACTGCAAAG TTACTATTTT GCGACTTTGT
85441 AATTAATAAA TATCTTAGGG AAGATACTTT GAATTCATGT AAATAACTTG TTTCTCTTCT
85501 AACCTCAGCC TATTGACTTT AGCATACCTC TGTAGACAGC TGTTAATCCT GTGGCCTCTG
85561 ATGGTGATTT TCTATTTACT TCATTTCTTC TGCATTAAGT AACTGGAATT CTATTAAAAT
85621 AAGCTGTTTC TTCTTATTTG TTTGTGCAGT TATTTACTTC ACTATGGGTT TGTAGATACT
85681 TATTTTATTA TTTGGGTTAT AATCCAACAT TATCTTTATT TTGTTTCTCA AATTGTTCCA
85741 GTGTTGGCCA TGGCAGCCCC TTCACAGTGG CTCCTGTGTG CTTTCACCCT ATCTGTTTTT
85801 GAGCCCTTCC TCACTTTCTG GCACCATAAA ATGCTCCAGG TTTATCTTGT AATTTCCTTC
85861 CCCAGAGACC CAGCTACTTC TCTAAGGAGT CCTGATTCTT TTAATTGGAA AATGGTATTT
85921 AGAAACCAAG ATCTGGACAT TGGGTGTGGT AGTTACTACT GGGGTGTCAT TTTTTTTTGG
85981 TAGACCTTCT CAGGGGAGAA TACTAGAAAG CATCTGTATA TACCCTAATC CATACACACA
86041 CACATTCACA CACACACACA CACACACACA CACGTGCGCA CACACATTTC TACCTATCTC
86101 TTCTATCTCT CTATCCGTCT ATCATCCATG AAACACATTG TTGTAAGCCC TTGGTTTTTG
86161 GGTGCTTTGT TATGCAGCAA TAGCTGACTG ATACATGCGG CTGGGATTGT GCACACTGTA
86221 TTTTCAGGCT CTTTGACCAG ATGTCTTCCT GGAAGGCACT GGTGGGAGGT GAGGAGAGGA
86281 GATTTCTGTT CCTTCACCAG CTTCTTTCAG GGCCCTCCAG CCATAGAGGG GGGTGGCTAC
86341 TCCAGCCCAC CACTTCTTTT GGCACTCCCA ATACTGGCTC TGCCACTCCA TCCTTTCTCT
86401 GTGCTCCTGG GTTCTGGCAG CCCAACCTCT TTCATTTGTT CCCCCAGCCC TGGGAGTGGA
86461 AGCTGCTTCC TGCTGTTCAC AACGTCAGGT GCCTCGGTGC CCGTCTTCTC TTCCAGCCTC
86521 CAGCACCTGT GTAACCAGTT CTCTGTATTA ACTTCCTTCT GTTTGAAATA ACGAGTGTGG
86581 CTTCCTTTTC CTGACTGACT GCACAGTGAT GGATGTCAAG TGGACTTTCA AGGTGAGTCT
86641 GAGGTACAGA AGCTGGAGAT GCACTAGCGG GTGGATGAGG AGACAGACCC TCAGGAGGGA
86701 GGGGAACGTG CGGTTTAGCC AAAGCCGCAT GTAGGGAAAA TCCACCACGT TCCACTCCTA
86761 CATGGGAAAA GAAGGAAAGC CTCAAATCAA TGGTCTCTCT ACCGTAAGAA ATTAGGAAAG
86821 AAAGAGAAGA GCAAATTAAG CCCAACGTGA GCAGGAAAAG CAAAATCATC AATAAAACAA
86881 CAAAATGAAA CACTAGAGAA AAGTCAACAA AACCAAAAGC TGGTTCTTTG AGAAGATCAA
86941 TTAGACGGAT AAACCCCTAG CCAGGCTGGA GAATAAAAGA GAAAACAGAC AAATTACCAA
87001 TGTTAGGAAC AAGACAGGTG ACGCCACTGT GGATTCTACA CATATTACAA AGATAATAGG
87061 GGGACATCAT GAACTACTTC AAGTCGAAAC ATTTGGCAGC CCGGATGAAA AGGACAAACT
87121 CCTTGGACAC AGGATGAATC GAAGATCACT TGGGCAGAAA TAACTTGAAT GGCCCTATAT
87181 GTTAAAGGAA TTGAATTGTA AATAAAACAC AACAAAGCCT TCCTGCAAAG AAAACCCCAG
87241 GCCCAGATGG TTTCACTGGT GAATTCTACC AAAGATTTAA GAAAGAAGTA TGTCAACTCT
87301 GCACAAACCC TTCCAACAAT AACTGAAAAG AGAGGAACAC CTTCAACTCT TCCTCTGAGG
87361 CCAGTATTAC CCCGATTTCA AAACCACATA AAAACATTAT AAGAAGACTA TGAACCTTTC
87421 CTTTGAAAGC AGTCTTTAGA TACAAGCTTT ATCTGACACA TTAACATGTT AGACTGCTTT
87481 CAAATGAAAG ACTGTGTATC AAATGAAAGA AGGGGCTTAT GAAGGTGTTG GAAATGAGAT
87541 CATCGGGAAA AGAGTGTGGG AAGGGGCTTT CCTATCCCTC TTATTTACAA CCCAACTTAA
87601 TAAACACACG GTATACTATT TCCTGCTATT CTTTGTCCTA TGTTTCCTGG TTATTTTGAT
87661 AATGTACTTT ACCACTGTGG CCAGTTCTTC TCCTGACTCT TTCACATAAA TAAAGTGTCC

87721 ATATTTCTGG AAAAAAAAAA AAGAAGGCTA TGAACCAACA TCCCTCATAA ACACAGATGT
87781 AAAAATTCTA AACAAATTAA AAAAATATTT TACTTTAAGT CCTGGGATGC ATGTGTAGAA
87841 CGTGCAGGTT TGTTACATAG GTAAACGTGT GTCATGGTGG TTTGCTGCAC CTGTCAACCC
87901 ATCACCTAGG TATTAAGCCC CACATGCATT ACCTATTTGT CCTGATGCTC TCCCTCCCCT
87961 CACCTCCCAC CCCCCACCAG GCCCCAGTGT GTGTTGTTCC CCTCCCTGTA TCCACGTGTT
88021 CTCATTCTAA ACAAGATTTT TTCAAATCGA ATCAAAAAAT TTATTAAAAC TATAACCACA
88081 GGGGATTTAT TCCAAGAATC CAGAGTTGGT CTAACATTCA AAATCAATGT AATTCATCAT
88141 GTGAACAGAC TAAAGATGAC CAATCATAGG ATCATCTCAG TCAATTCAGA AGAACGCACT
88201 TGACCAGATT CAACACCCAC TCCTGATAAG AACTCTCCTA AAAATAGAAT ATAACTTCCT
88261 CATCCTCATA ATGGCATTTA TAAAAAGCCT GCAGATGACA TCATACTTAA ACACTAATGC
88321 TTTCCCCTGA AATCAGGAAC AAGACCAAGA TACCTGCTCT CACCACTTCT AATGAACATG
88381 GTGCTGGAAA GCACAGCCAG GCAATCGGGA AGGTGAATAC ACTTAAAAGA TCTAGATTGG
88441 AAGGGGGAGT GAAACTGTGA CAGATGATGT GATCATCTGT GCATAATCGA GTATCTATAA
88501 AAGGCAGCGG CAGTTGTCCC TTGATATCCA CGGGGCATGG GTGCCAGCAC CCCTAGGACA
88561 CAAAATGTAT GCGTGCGCTA ATCCTCTACA TAAAATGGCA TAGTCTTTGC ATATAATCTA
88621 CATACATCTT CCCATGTACT TTATTTATTT ATTTATTTTT TTAAGACAGA ACCTCTCTCT
88681 ATCACCCAAG CTGGAGTGCA GTGGTGTGAT CTAGGCTCAC TGCAACCTCT GCCTCCTGGG
88741 TTCAAGCAAT TCTCCTGCCT CAGCCTCCCG AGTAGCTGGG ACTACAGGCA ACTGCCACCA
88801 CCCCTGGCTA ATTTTTGTAT TTTTAGTAGA GACAGGGTTT CACCATGTTG GCTAGACTGG
88861 TCTCGAACTC CTGACCTCAG GTGATCCACC CGCCTCGGCC TCCCAAAGTG CTGGGATTAC
88921 AGGCATGAGC CACCACGCCT GACCACATCC TCCCGTATAC TTTAAATCAT CTTTATATCA
88981 TGTTAAAATT TCTATTAGGG GCCAGGGTCA GTGGCTTACG CCTGTAATCC CAGCACTTTG
89041 GGAGGCCGAG GCAGGTGGAT CATTTGAGGT CGGGAGTTTG AGACCAGCCT GGCCAACATA
89101 GCAAACCCCT GTCTTTATTA AAAATACAAA AAAAATTTAG CCAGGCATGG TGGTGTGTGC
89161 CTGTAATCCC AGCTACTCTT GTGGCTGAGG CAGGGTGCGG TGGCTCATGC CTGTAATCCC
89221 AGCACTTTGG GAGGCTGAGG TGGGCAGATC ACCTGAGGTC AGGAGTTCAA GACCAGCCTG
89281 GTCAACACAG CAAAACCCTG TCTCTACTAA AAATACAAAA AGTAGCTGGG TGTGGTGGTG
89341 GGCGCCTGTA ATCCCAGCTA CTTGGGAGGC TGAGGCAGGA GAATCACTTG AACCTGGGAG
89401 GTGGAGGTTG CAGTGAGCCA AGATTGTACC ATTGCACTCC AGCCTGGGTG ATAAAGCAAG
89461 ACTCCATCTC AAAAAAAAAA AAAAATTAAA AGCAGGTCTT GAAAAGATAT TTGCACACTC
89521 ATGTTCACAG CAGTTGAAGC CACCCAAGTG CCCCTTGACA GATGAAGGGA TAAACAGAAT
89581 GTGGTCTGTC CTTACAGTGG AATATTATTC TGCCTTGAAA AGGAAGGAAG GAAATTCTGA
89641 CACAGGCTAC AATACATAGA TGACATGAGG CCGAGTGAAA TAAGCCAGAC ACAAAAAGAC
89701 CAACACGGTA TCATTCCACT TATTTGAGGT ATCTCAAGTT GTCAGATTCA TAGCAACAGA
89761 AAGCAGAATG GTGGCTACCT GAGGCTGGGG GAGAGCGGAG GGTGTGTTTA GTGGGAGCAG
89821 AGTTTCAGTT TAAGAAAAAT GAAGGTGTTC TGGAGCTGAA TGCTGGTGAT GGCTGCACAG
89881 CATGGTGACA TAGTCAATAC CACTGAGCCA CGCACTTAAG ACTGCGTAAG ATGAGAAATT
89941 CTGTTATGTG TATCTTTCCA TAATAAAAAG TCCCAAAAAA TCAATTGTAT CTCTCTATAT
90001 TAGCAATAAA TAATTGGAAA TGGGAACAAA CAATATCATT ATAACGGTAC AAAAATATGA
90061 AATACTTAGG GAAAAATCTG ACAAAGGATG TGAAAGACGA GTACGCTGAA AACTGTAAAC
90121 CATTGGGAGG GAAATTAGAG ACCTGCAGAA ACAGGTATGT ACACCTTGTC CATGGGCTGG
90181 AAGACTCCAT ATTGCTAGGA CGTAAGTTCT TTCTAAACTG ATAAATATAT TTAATGCAAT
90241 TTCAGTCAAA ATCCCTGCAG GCTTTTTTTT TTTTGGTAGA AACTGACAAA TTGAGACTAA
90301 ATTCAGAGGG AAATGCAGAG GACATAGAAG AGCCAACGCA GTCCTGAAAA AGGGGCAAAG
90361 TTGGAGGAAA CTGAAACATT GGCTGGGATT GAGAACTGTT ACAAAGCTAC AATAATCAAA
90421 ACAGTGCAGC ATTAGCATAA TGACAGACAA ATAGGTCAAT GAACAGAGTA AAGTCCAGAA
90481 ACAAATTCAT GTGTATATAA ACAATAATTA TTTTTTCTTT AAAAAAATTT TTTTTTGAGA
90541 CAGGGTCTGA CTCTGTCACC CAGGCTGGAG TGCAGTGGCA TGATCTCAGC TCACTGCAAC
90601 CTCTACACCC TGGGCTCAAG TGATCCTCCC ATCTCAGCCT CCCCAGTAGC CATATAGGCA
90661 CATGCCACCC CACCTGGCTA ATTGAATTTT CAACAAAGGA GCAGAAGTAC TGCAATGAAG
90721 AAAGAATAGT CTTGTCAACA AATGGGGCTA GAACAATTGG ATATCCATAC ATAATAAGAC
90781 AAACTTCAAG CCATACCTCA TACTACATAT AAAAAATTAA CTCAAAATGC ATCATGGAAA
90841 ACCCCAAACT ATAAAATTTC TAAAAGAAAA CATAGGGGAA AATATTTGTG TGATCTTGGG
90901 TTAGGCAAAG ATTTCCTACA TGTAATACCA AAGGCAAAAT TTATAAAAAA GTAAATTGAT
90961 CAATTGGATT TTATCAAAAT TTTGAAACTC CTACTTTAAA AAACACTATT GAGAGAATGA
91021 AAAGACAAGC CACAGACTGG GAGAAATATA TCTACAAAGG ATCTCTCCAA TAAAGGACTC
91081 GTATCTAGAA TACATAAATA ATGTTTAGAT CTTAAAAAGA AACCCACCCC AATAACCCAA
91141 TAAATAATGG GCAAAAGATT TGAGTAGGCG TTTCACTAAA GAAGACATAT GGGGCTTATA
91201 AGCCTGGGAG TAAACTGACT TTTTTTTTTG TAAGAAATTA GATATCCTAA GTTAAACAGT
91261 CACACAAGGA TGTGAGGAGA AAGTGCTTTG ACAGGAATTG CTATACTAGT CACAAGTACA
91321 TTACCAAAGA TTTCTCTATA ATGGATTTAA TATTTAAACA CAGCACCCAG TATTTGTTGA

91381 AAAAGCAAAA CTATATAATA GGGGTTTTTA AAATAATTTT TTTTTTGAGA CAGTCTCACT
91441 CTGTTGCCCA GGCTTGAGTG CAGCAGCACG ATCTTGGCTC ACTGCAACGT CTGCCTCCCG
91501 GGTTCCACTG ATTCTCCTGC CTCAGCCTCC CAAGTAGCTG GGATTACAGG TGCGCCACCA
91561 CGCCTGGCTA ATTTTTGTAT TTTTAGTAGG GATGGGGTTT CATCATGTTG GCCAGGTTCA
91621 TCTCAAACTC CTGACCTCAG GTGATCCGCC TGCCTTGGCC TCCCAAAGTG CTGGGATTAC
91681 AGGTATAAGC CACTGTGCCT GGTCTAAAAT AAAGTTTTTT AAAAAGTGAA AAAAAGAAGA
91741 CATATGGATG GCAGATAAGT GTATAAAAAA ATTCTTAACA TCATTAGTCA TTAGTGAAAT
91801 ACAATTTCAA GTAATAATGA GACAACATCA CACACTTACT GGAGCGGCTA AAATTAAAAA
91861 GACTGACCAT CCCAAGTGTT GACAAGGCTG TGGGGTAACG GATGTCTCAT ACACTGCCGG
91921 TGGGAATGTA GAAAGGTACA ACCACTTTGG AAAGCAGTTT AGCGATTTCT CAAAAAGTTG
91981 AACATAGACC TACCTGTCTG ATCCAGATAC TCCACTCCTA GGTATTTACC TGGCAGAAAA
92041 GAAAGTGTAT GTCCACACTA AGATTACACA AATGTTCGAA GCAGTTTTGT TCGTAGTGGC
92101 CTCAAATGGA CAATGAGCCC AGTGTCCATT AGCAGGGAAT GAATGAGCCC AGGAAGGAAT
92161 GAGCTATTTA CACAGCAATG CTGGATGCAA TCTCAACATA ATTATGCTTA GTGAAAGAGA
92221 CCGAAGAATG CATACTGTAT AGTTCCATTT ACACAGAATC CTAGAAAATG CAAACAAATC
92281 TGCAAAGTCC TAGAAAATGC AAACAAATGC CATGGAAAAG AAACCCTGGG TAGCTCCCGA
92341 GTGGGGAAGG GTGGGAGGGA GAGATTCCAA AGAGGTGTAA GGAGCCCTGT GTGGGTGATG
92401 GGTGGGGACA TATTCATCCT GATTGGGGTG GTGGTTTCCC AAGTTCAACC AATTTATGTC
92461 AAAACTGACC CAATCATACA CGTAAACATG TGCGGTTTAT TGTATGTCAA TTCTAACTCA
92521 GTTAAGCTGT TTTAGCCTGT GTCAACGCAC TTTCCTGACC AGTAAATCCT AAGTGTTTTC
92581 CTTGTTGCCA CATAAAATCC TAGTCATAAT TTATAGTGGA TGCATGTTAT TCTAGAGTTG
92641 TTTGGCTCTT AGAGATTTAA AATTAATCAG AGAAATATAT TAGAGCCCAA ACTATAGTGA
92701 CAGTAAAAAA TGACCAGTGC TTGCCAGGGA TTTGTGGGGA AGGATGGAAT AGCTGAGACA
92761 CAGGGCATGT GTTAGGGTGG TACAACCATT TTGCATGCTA CTATCCTGGT GGATATTTGA
92821 CACTCTACAT TTGCAAAACC CAGAAAACTT AATAGTGCAA AGAGTGAACC TTGATGTACA
92881 CAAACATAAA AATATTCAGG AGGTTGAGGG ATGCCACAGA TGAAATGCGG AATGTGGCAA
92941 AACAATCCAA CTGTATTTCA AATGCATGAA ATAAACTCAC AGAAGGGGGC GGGGGTGGAG
93001 GGATGTTGAA GGGCCCTGAC CTTTGGGCAC TTCGGCAAGG AGCAAGGGGG CACTGAGATT
93061 CCTGCCCTGG GGCTACGACA CCACCCAGGA ACCCCTGATC TTTCTGTTAC TTATGACTGG
93121 GGTTTCTCCA AAAAGCAACA GTCTCCTTTG CAAAAAGTGG CCACACCATT ATCAAGCGGT
93181 GGACAGTGTA AGTTTGGGGC TGGGAAGATT CTCTGGGACC ACATTGCAGC TCTGCCATTC
93241 TTGGGCTGGG TGAGCCGAAG GAGTGCTTTC ACCCTCTCTG AGTCTCAGTT TCCTTGTCTA
93301 TAGGATGGAG TGAGGTGAGT GCCTTCCTCC CAGGGTTGAG GGCTCCAGTG AGGTGGGCCT
93361 ATAGAGCAGT TGGCCTGTAC TTGCTCATCA TGAGCTCTTG TGAGGTGGAG CCCTGTGGAG
93421 CTGCCACAGA AGGTGAACTC CAGTACCAGG GAGATTTCAT TTGTCCATCC TTTTACCCAC
93481 CCACCCACCG ATTAATCCAT CATCCACCCA CCCACCCACC CATCCACCCA CCCACTCATC
93541 CATCTATCTG TCCATTTGTC CATCCATCCA TCCATCCATC CATCCATCCA TCCATCCACT
93601 CATCCACTCA TCCATCTCTC CATTCATCCA TCCACCTATC CAGTCACTCA CTCACATATC
93661 CATCTATCCA TCCATCCTTT CATCCATCCA TTCTTTGACC CATCCACACA TCCATCCCTC
93721 CATTCATCCA TGCCTCCATC CATCCACCTA TCCATTCACT AACCCATCTA TCCATCCATC
93781 CATCCATCCA TCTATCCATC CATCCTTCCA TTCACCCATT CATCCACTCA TCCATCCATC
93841 CATCCATCCA CTCACCCACC CATTCATCTA TCCATCCATC CACCCACCCA TTCATCCATC
93901 CCTCCCTCCC ATCCATCCAT CCCTGACCCA TTCATCCATC CCTTCCATCC ATCCATCCAT
93961 CCATCCATCC ATCCACTCAC CCATCCACCC ATCCATCCAT CCCATCCATC TACCCACCCA
94021 CTGACCCATT CATCCATCCT TCCCTCCCTC CCTCCCATCC ATCCATCCTT TCATTCACCC
94081 ATTCATCCAT CCATCCACCC ACCCACCCAT TCATCCATCC ATCTAACGAT CTGTCCCTCC
94141 CTCCCTCCAT TTATCCACTC ATTCACCCAG CCAACCACCC ATTCATCCAT CCGTCCACTC
94201 ATTTACCCAC TCATTTATTG ATCTATTCAT CCATCCATCC ATCCATCCAT CCATCCATCC
94261 ATCCATCCCA TCCATCTACC CACTGACCCA TTCATTTCCC CTCCCTGCTT CCCATCCGTC
94321 CATCTATCCA TCCATCCATC CATCCACCTG CTGACCCATT CATTCCCCCT CCCTTCCTCC
94381 CATCCATCCA TCCATCCATC CATCCACCCA CCCACCCACC CACCCACCCA CCCACCCACC
94441 CAGAAGCAAA GTTATTGAGT GCATCTGCTG TGAGGGTGGC CATGGGGGGC CCACATGGGC
94501 TGCTGCCCTT GCAGCATTCG GAGCAGGATG GCCAGCAAGG GTCACACAGT GTGGCCAGGG
94561 TGGTGAGAAC AGTGTTCAGT GTGAGGATGT GACACCTGGA CCAAGTCTTG AGGGGCAAGT
94621 GCCCCAGGGT GTCTGGACTC CTGAAGCACA GCTATCCACC TGATGGCCCT GGCCCTGGGC
94681 TGCCCTTTCC TCAATGGAGC CTGCCCAGTG CCCGCCTGCC CAGCAGAGGC CTGGACTGGT
94741 TGAGGCCTCT CTTGATCCCT CACCTGAGAA CAAACGCCTC TGTGTGCTCA CTTGCTCACC
94801 CTCCACACCT CACAGAGCAG TGGCACCAAC ATCCCGCCTT TCACCTCTGC AGTCCTTGGG
94861 AGGTGAAGTG CACACCTCCA GGTGTCTCCG AACCTTTGGA GCCCCAGAAA GTGGACATGC
94921 TTGGGGTGTC TGAGTCTGCC CTGGAGAGCT GGACGTGACT GGTGTTTTAT TTGCCCTCCC
94981 TTGAATCCTC AGATGCTCAC CTGCCAGGGT GTAGGTCTGG GCCTTGCCAC TGTGTCCCTG

95041 TGTGGGGCTG GGGTTGGAGC TGGGGTCCTC CTTGGTGTCC AGAGGGCTGA GAACCACGGC
95101 TTCTCTGGGT GTGCTCTCCA TCTGCCCACC AAGGCAGGGC CAGAGCTGTG GCGCTGGGGT
95161 TGGGTGCCAG CCCCCACCAC CCTGGTTAAG CCCAGTTGGC TGGGGGCTGG GCAGCCTGGT
95221 GCTGGCAGGG TTGGCTGTGG CAGGATGGCT CTGGCCTGCA GAATGGGTGT GGGCCAGCCT
95281 GGCCTCTTGT GTCCTGAGAT GCTGGCCTTG AGATGGAGAC ACAAGATGCT CGGTGACTCA
95341 GTTTCCCCTT CTGTAATGTG AGCTAATAAT CAGACCAACC ACATAGGGAC ATGGAAAAAT
95401 GGCCCCAGAT GACACATGTG ACTGACATGT GGGCAGCCTG CACTTGCTGG AGGCCGACAT
95461 TTCAGTAATC AGCAATGGGG GCCACGTTTG GACCAACACA GGATGTAGGG AGACAGGGGC
95521 TGCCACGTGG GGATACCTAG GGCTGCGGTG TGGGCGGGTG GAGTGCTCTC CAGGAAAGCA
95581 GGCTCTGTGA CTGGTCTCCC GCCCCCGTGG GAGAGGTATT GGCCACATGG GGAGTGCTTG
95641 CGGGGGTGGG TGGTGATACT CCAGGGAAGC AATCAGTGGG GCATGGGCCA TTACTTCAGG
95701 CAGCCTAGGG TGGGCCTCAG GCTGGAGACC CCCGGAGGCC ATCAGGAGGC AGCACGTCCT
95761 GGAGGCAGCA CGTCCTGCTC CTTGGAGGAC CAAGGTCTTT TCTTTCAAGG CCCTGCACTG
95821 ATTGTGTCAG ACCCACCTGC TTCGCCAGGG TCACATTTAA GAAGTCCCTT CACAGCAGCA
95881 TCCAGCCCGG CGCCATGCCA AAGACTGGAG CCATGGCCAG ACAGGCCGAC TCACAAAGCC
95941 ACCGTTCGAG CGACCGGGAC GTGTGCAGGG GACGCACCTG CTGTCTTTGG CTCCCCCAGC
96001 AGCATAGGGG TTCTCTCTTC TCGCCCCCTC CCCAGCCTCC TCTTCCCCTT CCCCTTCCTC
96061 CTCCTCCTTT CCCTCTCCCT CTTCCTCCGG TGGCTCTGAC CCCCACATGC CCCTCACCCG
96121 GGCTTCCTGG TGAGTCAGGT TTGTGGATAC ACAGTAGGGG GGCCGTCACT TCAGGTGCCA
96181 TGTGACGTCA GTGCTGCCTC CTCCCTGCAG TCACTACTGC AAATTCCATG CAGCCGTTGG
96241 CAACAGTGTC TTGTCACGGT AAACATGTTG GGCGTGGGCA GGGTGGGGAG AGGCTGGGGG
96301 GGCCAGGATG CTGTCTGTGT GGGAAGCTGT ACTCCAGGAT GCTGTCTGTG TGGGAGGCTG
96361 TACTCCAGGA TGCTGTCTGT GTGGGAGGCT GTACTCCATG CAGCCTCGTT CTGAGTCCCA
96421 GCCGGGGCCT GAGGCTGGGG CAGCCTGACA TGGGGCTGGG GATGTGGGCT CCTGGCTCAA
96481 CTGTGTCCGG CCCGCCGTTG GGTGGGTGGT CATTCCCACC ACTGCCATCA TGTTCTTGTC
96541 ACAGAAAGCA GAACCGACAA GCATTTTCCT CGGAGTGGTC AAAGCCCCCA CTCTCTCCCT
96601 CCACCAGGGT CCCTCGAACC CCATGGGAAA GCCACAGAGT GGCGATGGGG TCCCAGGGCA
96661 ATACGGCTGC CCACTCTCCC ACTCTGCACT TGCTGGAGGC CGACATTTCA GTAACCAGCA
96721 ATGGGGGCCA CATTTGGACC AACACAGGAC GTGGGGAGAG AGGGGATGCA GTGCTGTGGA
96781 ACTGCCTGGC CCCCATCGCC TCCACCACCA CAGTAGATGC TGCGAATCTG GCAGTGGGGA
96841 CCGGCTGAGG CTTGGAGGCA AAGAGGCTGG GGACACTCTC GCTTTGGGGG TGCCAGTGAC
96901 TCCATAGTCG CCCCGGCCTG ATGGAGCCCT CACTGTCCTC TTCCCCAGAA CAGCTCCCTT
96961 TCCCCGTTAG GAATGGGGTT CAGCTCTCTG CACCCTGCCC CATGCAGGGC TGCCTGTAAC
97021 CTCAGACAGG GCTCCTACCT GGGACCTTGC CATGGGCCAA GAGGGGTGTT GGGGTCAGAC
97081 CCCTGAGCCT GGGAACCACC ACTGCCCGTC CAGAGGGGAC ACAGGGTCAG ATCTAGGGGA
97141 CCTACCTCAG GCTTCAGGGC TGGAGGGGCT GTGCTGGGCA GCTTGTGCTC CGCTAGGATT
97201 CCTCCAATCC CCCCAGGGCA GGGGGAGCCT GGCCTGGAAA ATCTCTGTCC TGAGGCCCGC
97261 CTGGGGCATG TCAGGGTCAG GGGACTGCAG TTGGGGCCGT CCTTGGGTTT CCCCAGGGCT
97321 CAGTGCCAGT GTGCGGAGGC CCACACCAGG AAGTGAACAA TGATCTCCTC TGGCCTCGCC
97381 CAGCTGGCTC TGGTTTCCTA ATCCCCGGTC CTCCTGGCAG GGGCCACACA CTGAGCTTCC
97441 TCCACGTGCC CAGGTCCTGG CAGGGAGCGC AGACCCTGGG GCCTGGTGCT GGCGGGCACC
97501 GCAGGAGGGC GGGAGGGGCT TCGTCCCAGG CCCTGGGTCT GGGCAGCAGG TCAGCCAGGG
97561 AAACAGGCTT GGTGCTTTGG GCCCCGAGTC TCTATAACTG TTGGGGTGAG TCCCTCCCCA
97621 CTGCCATCAT GCTGCCGGCA TGTCCCTGGC ATGTTCAGGC CAAAGCCAGG AACTCAACTC
97681 AGGGCCCCTC TCTATTTTCA GGAGGAGAAA ATTGTAGAGA GAGGGGAGGG CCCCCAGACC
97741 TCAGTTTACC CACTGGCGAC ACAGGGGTGC CTGCCTGTGC CCTCCCGGGC CGGGGCAAGC
97801 AGTGGTGGGC CCAGTGGTCT CGTAGTCTGG GGTCGGTGTG AGTTCCGGTT CTCCAGGCTT
97861 TTTTCCAGAC AACTGCTGGG ATTGGTGGGC GAGACCAAGG CTCATCAAAG GCACAGCCTT
97921 GGGGGCAGGA TCCCCACCAT GAGTCAGAGG TAGTTCTGGG GAGCCTGGGC AGGCTGTCAC
97981 CTCCTCAGCT GTCAGGCCCG AGGTCCTCAT GTGGTCCCCA GGAGAAGGGG CAGACGGCCA
98041 CTTCCGGCCA CCAGCCAGCT CCCTGTGTGC CTGATTCCGT AACATGTCCC CTGGCTGGGC
98101 ATGTACTCCC CAAGTTCTAA TTACATGTAA CTGCAGAGAA GGGCTCAGCC TGGGAAAAGG
98161 ATGGGCATAG GGGGTGGTTG GGGGCTGGGG CCTCTGACAC AGCTCCATGA GCCCGGCCAA
98221 GAGTCCCACA CAAGTCAGTG GCCCCCCCGG ACCCTGAAGG ATCCCACATC CTCCCTGCCC
98281 TCGGGGAGGC CCCTTTCTGG GGTCAGGCCT GGAAGCTGCC CCAGAGCTTG GGCCCCAGGA
98341 ATGGGTTGGT CCTCCCAGCG TAACGTGAGC CTGATCAGGC CTGGGGACCT GCTCAGCGGG
98401 TGTCTGGGGG CCCATGGCGG GCTAAGGAGC CTGACCAGAC TTGCTTCTGG CAGGACACCC
98461 CTCCCCCGGC CACCCTGGGC TCGCCCCTCT AGTAGCTGCA TGTGTTCCCC GGGTGTGTGT
98521 TGGCATTCAG GCTACAGGGC TGCCTCATCC TGAAGAAGGC TGCGTTTACC CAGGGAGCCA
98581 TAAAGAGATG ACCTCCGATA ACCTGAATCA ATATTTCCCC ATTGGGGCTC GGGCCCCCGC
98641 AGCTGTCTTC TTGATCATCT GGCAGATGCC ACACCCACCC TTGGCCCTCC CCTGCCTTCC

```
 98701 TGCCCTCCTA CCCTCCTGCC AGGACATATA AGGACCAGAC CCCTGCCCCC GGGCGCAACC
 98761 CACACCGCCC CTGCCAGCCA CCATGGGGCT GCCACTAGCC CGCCTGGCGG CTGTGTGCCT
 98821 GGCCCTGTCT TTGGCAGGGG GCTCGGAGCT CCAGACAGGT GAGAGAGCAG ACACAGGGGT
 98881 CTGGGGCCTG GCAGAGTGTC CTGGGGGCAG GGCGAGGCGG GCGGGCAAGT CGCGTCTGGG
 98941 AGGAGGAGCT GGTCCCAGAG TGCAGCCTGC GCGGCTCTGC TGAGGCTCCT GGCCCGGGTT
 99001 GGTCCCTGGA AGCCCCCGGC CCTGCTGACT TTCAAGGAGC TGGAAGGTCG GGGCTCCCCT
 99061 GCTATTCCTT TGGGGTTGAC TGCCCGACGA CAGTGTGGGT CTTGGGGCCA GCACCAGGTG
 99121 GAAACAGCAG GTCAGGCCCC AGTGAACTGG GTCATTGTCC ATAGGGGAGG AAGGGGTGGC
 99181 CAGGATCCCA CCAGAAGGCC CCATTCTCAG GTGGCAGAGA CCCTTGAAGA GTTGGGGCAG
 99241 CACAGCCCTT GCTGGGGAGC GGGGTGCCCA GAATGCCCTC TCCTACATCC CGCTTGGCAC
 99301 CCGGCCGCAC TCCTCACCAG GCCGGGGGTA GAAGCCCTGA GACCCCTGTG GTGGGGTGAC
 99361 CAAGGCCCAG CAGAGGGCCC GAGGATAGGA AGGAACCTTT CCCGGCCAGG GGCCCTGTGC
 99421 TGGGCTCGAA GCTGCTTCCA GGTGCTTCTT CAGGGGCCTT CTCTCGAGGG TAGCTTGGGC
 99481 AGCCTTCCCC CTCCGGGGCC ACTCACCCCT CATTCCCCGC TGCTCCCTCA GAGGGCAGAA
 99541 CCCGAAACCA CGGCCACAAC GTCTGCAGCA CCTGGGGCAA CTTCCACTAC AAGACCTTCG
 99601 ACGGGGACGT CTTCCGCTTC CCCGGCCTCT GCGACTACAA CTTCGCCTCC GACTGCCGAG
 99661 GCTCCTACAA GGAATTTGCT GTGCACCTGA AGCGGGGTCC GGGCCAGGCT GAGGCCCCCG
 99721 CCGGGGTGGA GTCCATCCTG CTGACCATCA AGGATGACAC CATCTACCTC ACCCGCCACC
 99781 TGGCTGTGCT TAACGGGGCC GTGTGAGTGT GGTCGGTGGC ACCCCTCCCA CATCCTAGCA
 99841 ACGGGGGCTG ATGTTTCCCA AAGGGATATT CCTTGTAGCC CTAGAAGACC CCTTCCGCCC
 99901 CAGCACACAG CTCAGGAGAA CAGCCTTGAG GTTTGGGTTC AGGTCACTAA TTCATTCAAC
 99961 AAACACTGAT GAGCCCCCAC CATTCCCCCC ATAGGCAAGG GGTTTCAGTT ATCCCTTTGC
100021 CTGTGTGTCC CTGACAGCCC CTCCCCTCGG AGCCCACCAG GCTCCGGACA GACTTGGCAC
100081 CCCTGGAGGC TGCATGTCTC TGGTCCTGTG CATGGAGTGG CCGTGTGTGC CCTCCCCAGG
100141 CTAGAGTTAC AGAAGCCGGT GCAGGGGGCT GTGGGACCCC CTTCCCCATC CCCAGCTATT
100201 GCTCCCCTAT TGTCTCCAGA ACAATGAGGC CCTGTAAGTG CGTTCCCATC CAGCGCCTGC
100261 CCCTCTTCTG CCTGGGGATT TAGTTTCCTG CAAGGGGCCC CAGCATGGGC ATGGGCAGGC
100321 GGGTGGAGGC CCTCAGGCAT GGGCATGGGC AGGCGGGTGG GTAGAGGCCC TCAGGCGTGG
100381 GTGCAGGCAG GTGGGTAGAG GCCCTCAGGC ATGGGCATGG GCGGGCGGGT GGGTAGAGGC
100441 CATCAGGTGT GGGCGTGGGT GGGTGGGTAG AGGCCCTCAG GCATGGGCGC GGGCGGGTGG
100501 GTGGGTAGAG GCCCTCAGGC GTGAGTGCGG GCGGGTGGGT GGATAGAAGC CGTCAGGCAT
100561 GGGTGCAGGC GGGTGGGTAG AGGTCCTCAG GTGTGGGCAT GGGCAGGTGG GTGGGTAGAG
100621 GCCGTCAGGT GTGGGCGCGG GTGGGTGGGT AGAGGCCCTC AGGCATGGGT GCGGGCGGGT
100681 GGGTGGGTAG AGGCCCTCAG GCGTGGGCGC GGGTGGGTGG ATAGAGGCCG TCAGGCGTAG
100741 GTGCGGGCGG GTGGGTAGAG GTCCTCAGGT GTGGGCGCAG GTGGGTGGGT GGGTAGAGGC
100801 CCTCAGGCAT GGCACAGGTG GGTGGGTAGA GGCCCTCAGG CATGGGCGCA GGCGGGTGGG
100861 TGGGTAGGGG CCCTCAGGCA TGGGTGTTGG CAGGTGGGTG GGTAGAGGCT TTCAGGCATG
100921 GGCAGGCAGG TAGAGGCCCT TGAGGACCGA GGCACAGAGG CTGGGGTGAG TGCCTCTACC
100981 TGGACCAGCA AGGGGCACTG GCAGGAGGTG GGGTAGGGCC CCTGACAGTC TCAGGGGCAG
101041 CCTGGGGGGC TCTGGGGGGT TTGGGACCCC ATGGGGGGAT GTTCCACCAA GCAGGGGGCC
101101 TGGAAGGGGG CTGGGCAGCC TGGTCCTCCC TCCTCTCCCA ACCTGGTGCC CTCAGGGCCT
101161 CTGAGGGGGG ACCCTGCCCA GGACCGTGCC CCGAGGAGGG AGTGGAGAGG AGGGGCGTGC
101221 AGGCAGGAGG TGGCTCTGCC GGGGAAGCCC GGCCAGCGGA GATGGACAGG TGCTCTTTGG
101281 CCACTGCCTA TGTCCCTCCA CCCCAGAGGC CGGCCAAGTT GGTGACCCCA GGGCAGGAGC
101341 TGGGCCTGGC AGAGCCATCT CCACCACCCC AGGCGCCCAG CTTCAGTCCC CTCTGGGCGG
101401 CGGGGTCCCG GGAGGACAAG CTGGGGCGGG GGGGCCTGGG TGGTGGACCC AAGAGTGACC
101461 CCGATGTGCC TCCGCCAGGG TCAGCACCCC GCACTACAGC CCCGGGCTGC TCATTGAGAA
101521 GAGCGATGCC TACACCAAAG TCTACTCCCG CGCCGGCCTC ACCCTCATGT GGAACCGGGA
101581 GGATGCACTC ATGGTGCTCA GGGGTCCCCG GACTCGTGGG GCTGGTGGGG GCTCCGTCAG
101641 GCCTCTGGGC AGACCCCAAG GGAGGGCAGG GAGGGCAGTG CTCTGACCCC TCACCGAGAG
101701 GGCATGGGTG GGGCAGGGCC TCGGCAGCGC GGGGCGTCGG TGCTGGACTT GGGGGGCAGC
101761 AGCAGAAGCC GACCTGGCCC TGACCCCCCC AGGCCTCAGC CTTCCCCCAA ACGCACTCGG
101821 CTTCTCAGGG ACCTGCCCTG CCAGGCCGCT CCCTGGCTGC TGACCCCAGC CTTCCTGCCC
101881 CACCTTCCTC TGGCTCAAAC AAGCCACGAG TCTTGGGGGT TCCTGGCGGC TGTGGGCCGG
101941 GCGGGAGGCC AGCTCACCTG CTCCCTCCCG CAACAGCTGG AGCTGGACAC TAAGTTCCGG
102001 AACCACACCT GTGGCCTCTG CGGGGACTAC AACGGCCTGC AGAGCTATTC AGAATTCCTC
102061 TCTGACGGTG AGGCCCGGAG GGCTTGGAGG GGGCAGGGTA GGCTACGGGC CCCCAGGAGC
102121 CCTAGCTGAA GGGCCGTGCA TCCCCAGGCG TGCTCTTCAG TCCCCTGGAG TTTGGGAACA
102181 TGCAGAAGAT CAACCAGCCC GATGTGGTGT GTGAGGATCC CGAGGAGGAG GTGGCCCCCG
102241 CATCCTGCTC CGAGCACGTG AGTCCCCTCG GTCTGGGGTG GGGGTCCTGG CGGAGCTGGC
102301 CTCTGAATAG CATGCTCACC CTGCGTCTGT CCCCAGCGCG CCGAGTGTGA GAGGCTGCTG
```

102361 ACCGCCGAGG CCTTCGCGGA CTGTCAGGAC CTGGTGCCGC TGGAGCCGTA TCTGCGCGCC
102421 TGCCAGCAGG ACCGCTGCCG GTGCCCGGGC GGTGACACCT GCGTCTGCAG CACCGTGGCC
102481 GAGTTCTCCC GCCAGTGCTC CCACGCCGGC GGCCGGCCCG GGAACTGGAG GACCGCCACG
102541 CTCTGCCGTA AGCCCCGGCG CCTTGTGGGC AGGGGACCCC AGGGAGACCC CACGCTGGTG
102601 CTTTCCCCAA GCCCGGGTGG GAGCTGTGTC TGCGCCGGGC ACCTTGAGCT GGGGGGACAC
102661 TCACCGCACC GGGCACCTTG AGCTGGGGGA ACACTCACCG TGCCGGGCAC CGGGAGCTGG
102721 GGGGACACTC ACCGTGCCGG GCACCTTGAG CTGGGGGGAC ACTCACCGTG CTGGGCACTG
102781 GGAGCTGGGG GGACACTCAC TGAGGGCACC GGGAGCTGGG GGGACACTCA CCGTGACGGG
102841 CACCGGGAGC TGGGGGGACA CTCACCACGG GCACCGGGAG CTGGGGGGAC ACTCACCACG
102901 GGCACCGGGA GCTGGGGGGA CACTCACCGC ACCGGGCACC TTGAGCTGGG GGAACACTCA
102961 CCGTGCCGGG CACCGGGAGC TGGGGGGACA CTCACCGTGC CGGGCACCTT GAGCTGGGGG
103021 GACACTCACC GTGCCGGGCA CCGGGAGCTG GGGGGACACT CACTGAGGGC ACCGGGAGCT
103081 GGGGGGACAC TCACTGTGAC GGGCACCGGG AGCTGGGGGG ACACTCACCA CGGGCACCGG
103141 GAGCTGGGGG GACACTCACC ACGGGCACCG GGAGCTGGGG GGACACTCAC CACGGGCACC
103201 GGGAGCTGGG GGGACACTCA CCACGGGCAC CGGGAGCTGG GGGGACACTC ACTGAGGGCA
103261 CCGGGAGCTG GGGGGACACT CACCGCGCCG GGCACTGGGA GCTGGGGGGA CACTCACTGA
103321 GGGCACCGGG AGCTGGGGGG ACACTCACCG CGCCGGGCAC TGGGAGCTGG GGGGACACTC
103381 ACTGAGGGCA CCGGGAGCTG GGGGGACACT CACCACGCCG GGCACCGGGA GCTGGGGGGA
103441 CACTCACCGT GGGCTGAGAG CCCTTCTCGG TGCACTTCGG GGTGGAGCGG CTGCTGTGCC
103501 CCAGCCTCAC CCTCACTGCG TGGCCTCTGC GGTTCCAGCC AAGACCTGCC CCGGGAACCT
103561 GGTGTACCTG GAGAGCGGCT CGCCCTGCAT GGACACCTGC TCACACCTGG AGGTGAGCAG
103621 CCTGTGCGAG GAGCACCGCA TGGACGGCTG TTTCTGCCCA GAAGGTGCGT GTGGAGGATG
103681 GCCCCGCCCC GGCACTGCCC ACCAGATGAG AGGCAGCCCT GGCCTGGGGT TCTCGCCTGC
103741 GCTGAGGGGA CGGCTCCGCT GGGTGGTGGG GGCAGCGGCG GCACAGAAGT GCCTCTCCCT
103801 CCACCCGATA CCGGGGGAGA AGGGGCCTCG GTGTGAGGCC CTTCCCAAAG GGTGGCTTCA
103861 GGGAGGCCGG GAAGGGGGCT GCCTTCCTGG TTATCACCCT GGGGACAGAC CTCCTCCTGC
103921 CCGGCCCCTG GCCTGGTGCC TGAGGCCTTT GGGAGCAGCT CGATTGTCAG GGGCAGGAAG
103981 GTGGCCTGGA GGCTGGACCC CCATGGCCAG ACCCCAACCC AGGGACCAGG TGGGGACCGC
104041 AGGCGTCAGC ACAGGGGACC AGTGGTGCCT GCGGGTGGGA GGCCTGGCTG GCAGCCCCTC
104101 GGTGGGGATT CTGGCTCTTT CTGAGCCAGC CGGGGTGACA TCGCCTCCCT GGCTGTCCCA
104161 GGCACCGTAT ATGACGACAT CGGGGACAGT GGCTGCGTTC CTGTGAGCCA GTGCCACTGC
104221 AGGCTGCACG GACACCTGTA CACACCGGGC CAGGAGATCA CCAATGACTG CGAGCAGTGG
104281 TGAGTCCCGG GGCCAGGGCT GGGCACAGCA GAGGCTGGGG CGGCTGAGCC CTGACCCTGT
104341 GCCCCGCTGC CCAACAGTGT CTGTAACGCT GGCCGCTGGG TGTGCAAAGA CCTGCCCTGC
104401 CCCGGCACCT GTGCCCTGGA AGGCGGCTCC CACATCACCA CCTTCGATGG GAAGACGTAC
104461 ACCTTCCACG GGGACTGCTA CTATGTCCTG GCCAAGGTAG GCTGCCCAGG GTCTGGGGCA
104521 TGGGGCAGAG CTGGGGCTGG CATCCAGGCC CTTGGCTGTC CCGGGGTGGG TGGGCTGGCT
104581 GTCCCTGAAG CAGAGGGTGC CTGTGGGCTG TCCTGGGGCA GGTGACCATG CTTCTGCTCT
104641 CTGGCTGGAG AATAAGAAGC AGGCCTTCCT TTCTAAGCCA CTGCCGGGTC CTAGGGTGCA
104701 GGGTGCTGCC CGTCCCGGCC CTCAGCAGCT GCACTGCCTC TTGCCCCATC ACAGGGTGAC
104761 CACAACGATT CCTACGCTCT CCTGGGCGAG CTGGCCCCCT GTGGCTCCAC AGACAAGCAG
104821 ACCTGCCTGA AGACGGTGGT GCTGCTGGCT GACAAGAAGA AGAATGTGAG TGGTCCTGCC
104881 CCCTCCTTCT GGAGCCCCAG GTCCCCCGAG GGGGGCCCTT CTCAGCCCTG AGCAACCTCG
104941 GCCTTCCCTG CAGGTGGTGG TCTTCAAGTC CGATGGCAGT GTACTGCTCA ACGAGCTGCA
105001 GGTGAACCTG CCCCACGTGA CCGGTGAGTT GTGCCCCAGG GAGGGGCCCG GGCCCTTCGA
105061 GCTCCACTGG GCCTGCAGTG ATTCGGACAG TCCAGCCACC TCGGACCCAG GAGGCTGGGT
105121 GGGAAGGTTC CACGGGGGGA GGGTCCCTGC GGCACCCAGC AGGCTCCGTC CTGGGTCCTC
105181 TGCTGGAGGG GGTGGTGGGA GGGTGACACC CTCCCGCTGC TCACCTGGGC CAGGCAGGTC
105241 CCGGGAGCCC CGCCCCTCGC CATGCCCCTT ACTGTGTCCC TCATCGTGCC CCTGCCCACA
105301 GCGAGCTTCT CTGTCTTCCG CCCGTCTTCC TACCACATCA TGGTGAGCAT GGCCATTGGC
105361 GTCCGGCTGC AGGTGCAGCT GGCCCCAGTC ATGCAACTCT TTGTGACACT GGACCAGGCC
105421 TCCCAGGGGC AGGTGCAGGG TAAGTGGCCC CACCGGGGTT GCCCCAACAA AGGCCCACAG
105481 GGGGGCCTGC TAGCCCCAGA CTCTTCCCAA CCCTGTCCTG GCCCCTCAGG CCTCTGCGGG
105541 AACTTCAACG GCCTGGAAGG TGACGACTTC AAGACGGCCA GCGGGCTGGT GGAGGCCACG
105601 GGGGCCGGCT TTGCCAACAC CTGGAAGGCA CAGTCAAGCT GCCATGACAA GCTGGACTGG
105661 TTGGACGATC CCTGCTCCCT GAACATCGAG AGCGGTGAGG CTCGGCAACA CGGGCGCCCC
105721 CACCTAGCGT GCCTAGGGTA CCCGGCCCAT GGCCTGGAAG GGCAGACGGG GCTCCCAGCA
105781 GGAAGCATGG GTGGTGAGGG GCAGAAGTGA GGTGGCTCTC CTCCAGGGGC AGCCCGGCCC
105841 CTGCTGCTTC CTGCTGTGGC TAGTTTATGG CGGCCATGGT GGCAGCCTGC CAGGTGACCT
105901 GGAAGAGGGC CTGGGCTGGT CCCTACCTGC CCCGTCATGT CCAGGATGCT GGGCCCTTGG
105961 GGGTGAGAGA CGGGAGGTGG TGGGTGCCCT GCAGGGGTTT CTATCTAGCC AGGAGCTGCC

106021 TGGAAATTTG ACTCACGGGG AGGAAGGGGC CTGGGCATCG GTGCACAGAG GGAACCATAT
106081 CTGGGGCCTA GGCAGCCAGG CAGCAGGGCC CAGGGGATCT CACGGGGGTC CCGGGCCCCG
106141 CTGAAGTTCC GATCCCCCAC TCCCCAGCCA ACTACGCCGA GCACTGGTGC TCCCTCCTGA
106201 AGAAGACAGA GACCCCCTTT GGCAGGTGCC ACTCGGCTGT GGACCCTGCT GAGTATTACA
106261 AGGTGGGTGG GACCCACACC CCCAGGCCCC CATGCCATCG AGGTGGACTC AGGGCACCCC
106321 CAGCCCCCCA TGCCACCCGT GAGGTGGACT CAGAGCACCC GGTTGGGCCC ACTGGTTGCT
106381 GTGTGTGCGT GTGAGCTTGC ATCTGTGAGC GCCGGGCCAC ACTCTGCCTC CCTGCCTCAC
106441 TGCCCGTCCA CCTTGCTCTG TCGCCCAGAG GTGCAAATAT GACACGTGTA ACTGTCAGAA
106501 CAATGAGGAC TGCCTGTGCG CCGCCCTGTC CTCCTACGCG CGCGCCTGCA CCGCCAAGGG
106561 CGTCATGCTG TGGGGCTGGC GGGAGCATGT CTGCAGTGAG TGCCGTCCCC GTGGGCTGCA
106621 TCCTGGGGAT GGGGTCCGGG CTTTGAGCTC CTGGGACGGG GCTGGGGGCC CTGAGCACGG
106681 GTGGTCCAGG GAGAGGGGTC GGCCCCCTGC AGCCACGGAC CAGGCTCCAG CTTCGTCAGC
106741 CGGTGGTAGC AGGAAACCAG CAACTCCTAT AGCAAGGGGC GGCCACGTAG CAGGGGCAGA
106801 ACCTGGGGTG GGCCTGGAGC TGTGGCGGCC GAGTGTGGGA GTGGGTCCCA GAGTGTGCAC
106861 TCCCTGGCCC CCTGGCCACC CTGGGGATGG GAGCTGGGCG TCTGGCTCTT CCCGTCCCTC
106921 ACACCACCCC GTGGTCCTCT GCAGACAAGG ATGTGGGCTC CTGCCCCAAC TCGCAGGTCT
106981 TCCTGTACAA CCTGACCACC TGCCAGCAGA CCTGCCGCTC CCTCTCCGAG GCCGACAGCC
107041 ACTGTCTCGA GGGCTTTGCG CCTGTGGACG GCTGCGGCTG CCCTGACCAC ACCTTCCTGG
107101 ACGAGAAGGG CCGCTGCGTA CCCCTGGCCA AGTGCTCCTG TTACCACCGC GGTCTCTACC
107161 TGGAGGCGGG GGATGTGGTC GTCAGGCAGG AAGAACGATG GTGGGTACCT GCTCGGGGGT
107221 CAGGTGTGGC GTGGGGGCGG GGGAGCTCCT TCTGAACCTG CCCCAAGCGG AGACCTGGGA
107281 GTCTCTACCT GGGGAAGCTG AGACACCCAA GGCTGAGGGG TGCCTGGGGT GGGGGGCGCT
107341 GAGAGGCATC AGGCTCACAT CTGCGGGGAA GCTGCGGGCT GTCTGTGGCC GTCCTGCATG
107401 GGCCCCGCTC ATCCCTGGCC TTTTCCACAG TGTGTGCCGG GATGGGCGGC TGCACTGTAG
107461 GCAGATCCGG CTGATCGGCC AGAGTAAGTG GCACTGCCCC GGCCACCCCT CCCCAGCCAC
107521 CCCTCCCTGC CTGCCCTGGC CACCCTCCCC GGCCACCCCT CCCGGGCCTG CCTGAGACCC
107581 CCAGCTTCAG CTGGAGCTGA GGTGGCCCCT CCGTCCCACA GGCTGCACGG CCCCAAAGAT
107641 CCACATGGAC TGCAGCAACC TGACTGCACT GGCCACCTCG AAGCCCCGAG CCCTCAGCTG
107701 CCAGACGCTG GCCGCCGGCT ATGTGCGTGT TGGGGGCGCT GCTGTGGGCG GGCAGGGATT
107761 CCTGGCTGGC TGAGCCTGGC TCTTGTGCTG TGCCCCCGCT AGGGTCTGGG TGCCGAGTCC
107821 TGAGGACGCA GGCCCTGTTG ATGCTGTCCC TGGCCCTGGG AGGGAAGTGG CAGCCTGTGA
107881 GCCACCGGGG CACAGGGGCC AGTGTAGGGC CCTTGGCCGG CAGCCCTCAC CAGTCTCACT
107941 GCCCTGTGGC GGGCCCAAGG GGAGGGAAGC CTGAGCCCAG GCCAGGGGGA GTGGTGGGAG
108001 GTCTGGGACA TGACAGAGAC TGCACGGTCA GGCCTTTCCT GGTTGCACAT CCAATCCTGA
108061 CCCCAGGGAG GGCTGCAGCC TCACCTGTCC ACCCCTGAAC CCCACTCTCT GGCTGTCCCC
108121 AGTACCACAC AGAGTGTGTC AGTGGCTGTG TGTGCCCCGA CGGGCTGATG GATGACGGCC
108181 GGGGTGGCTG CGTGGTGGAG AAGGAATGCC CTTGCGTCCA TAACAACGAC CTGTATTCTT
108241 CCGGCGCCAA GATCAAGGTG GACTGCAATA CCTGGTAAGC TGGCCCGGCC TGTCCTGGCT
108301 GCCTCCCAGG CCCCACGTGC TCCGCAGGGG TGGCCACTGG AGAGCGGTCC AAGGGGCAAG
108361 TGCCTCTCCT GGGGGTTCCG CCTGGGTCTT GCGAGATCCT GTGGTGGCCC CTGTCCCACG
108421 GGCAGGGTGG TCTCTCATGT CAACTGCTGG TCTTGAAGCC ATGGGAGAAG GGACATTTGG
108481 AGCCACTTTT GGGGCCTGCA GGTGTCCTGT GTGGGAGGCA CAGGGAGCTG TCTGCACGGT
108541 GCCCAGGGTC TCCTCCAGCC ACCCATGAGC AGGTCCTGGG TCCCTTCAGG CTCCTCTCCT
108601 GTCCTCCTCA GCACCTGCAA GAGAGGACGC TGGGTGTGCA CCCAGGCTGT GTGCCATGGC
108661 ACCTGCTCCA TTTACGGGAG TGGCCACTAC ATCACCTTTG ATGGGAAGTA CTACGACTTT
108721 GACGGACACT GCTCCTACGT GGCTGTTCAG GTGTGGTCAC GGGCACTGCC TGGTCGGGCT
108781 GCTTATGGTC AGGGACCCTC TGCCTGCCCC AAGTGCAGTG CTTAGCTCCC CGAGAAACCC
108841 TGAGACTTGG GAAGGCCGGC CTTTCCTCAG CCCCAGACCC GCACCTGCAC CCGCAGGAGG
108901 ATTCGTTCTT CTAGCCAGGG CTGGGTAGGG GTGGTAAAAC CCCTCTGTAC TGCCCAGTTC
108961 TGTGGTTCTC CTCTGGGTCC TCCTCTGGGT TCTCCTGTGG GTCCTCCTCT GTGGTTCTCC
109021 TCTGGGTCCT CCTCTGGGTC CTCCCTCCTC TGGATCCTCC CTCCTCTGGA TCCTCCCTCC
109081 TCTGGGTCCT CCCTCCTCTG GGTCCTCCCT CCTCTGGGTC CTCCTCCAGG TCCTCCTCTG
109141 GGTCCTCCCT CCTCTGGGTC CTCCTCTGGG TCCTCCTCTG AGTCCTCCTC TGGGTCCTCC
109201 CTCCTCTGGG TCCTCCTCTG AGTCCTCCTC TGGGTCCTCC CTCCTCTGGG TCCTCCCTCC
109261 TCTGGGTCCT CCTCTAGGTC CTCCTCTGTG GTCCTCATTT GGGTCCTCCT CTGGGTCCTT
109321 CTCTGGGTGC ACAAGGTGGG TGCACCAGCC ATGGGGACTG AGGGCACCTG TTTGGGGAGC
109381 TGAGTAAAGG CCAGGGCTAG GCCGCTGCCC GCGCGGCTCT CCAGATCCAA ATCCCACAGC
109441 CCTTTGAGGC ACCGTGATCC CCAGGGACAG GGGACAGGCC TGCAGCAGGG TCAGGTCCTT
109501 GGATGGGCCA GGCCAGGGCC TGGTTTGTCT GCTCAGTGGC TGTGACCCTG CCAACTGGGG
109561 CGGGTGTGCC CCGGGACACC TGGGGTCCAG CTGTCCTGGC TGACCTTGCC CTCCTGGCCC
109621 CCAGGACTAC TGCGGCCAGA ACTCCTCACT GGGCTCATTC AGCATCATCA CCGAGAACGT

109681 CCCCTGTGGC ACTACGGGCG TCACCTGCTC CAAGGCCATC AAGATCTTCA TGGGGGTGAG
109741 TGCTGCTGGC CCTGGGGACG CGTGAGCCCT GCGGGACCCT CAGACCAGCC AGTGACTGGG
109801 CCTCTCCTCC GGGCAGAGGA CGGAGCTGAA GTTGGAAGAC AAGCACCGTG TGGTGATCCA
109861 GCGTGATGAG GGTCACCACG TGGCCTACAC CACGCGGGAG GTGGGCCAGT ACCTGGTGGT
109921 GGAGTCCAGC ACGGGCATCA TCGTCATCTG GGACAAGAGG ACCACCGTGT TCATCAAGCT
109981 GGCTCCCTCC TACAAGGTGG GCTGCCTCCC TGCCTGCCCT GCCCCCTCCT GGCCAGCCCC
110041 CCACCCCCTG CCCTGGTGTT TGCAGGACAA GCCCCTGTCC TCCCTCCAGC CCCTTTTTGG
110101 AGCCCCTGTG ATGCTTGTCT CTTGCAGGGC ACCGTGTGTG GCCTGTGTGG GAACTTTGAC
110161 CACCGCTCCA ACAACGACTT CACCACGCGG GACCACATGG TGGTGAGCAG CGAGCTGGAC
110221 TTCGGGAACA GCTGGAAGGA GGCCCCCACC TGCCCAGATG TGAGCACCAA CCCCGAGCCC
110281 TGCAGCCTGA ACCCGCACCG CCGCTCCTGG GCCGAGAAGC AGTGCAGCAT CCTCAAAAGC
110341 AGCGTGTTCA GCATCTGCCA CAGCAAGGTG GGCTGGCCGG GCCATGGTGG GGCAAGTAGG
110401 CAGAGGAGGG CTGTAGGTGG GCTGTGACTG TGGGCTGGGG CCATGGGCGG GGCCGACTAA
110461 GCAGAGCAGG GCTGTAGGTG GGCTATAGCT GTGGGCGGGG CCATGGGCGG GGCCGACTAA
110521 GCAGAGCAGG GCTGTAGGTG GACTATAGCT GTGGGCGGGG CATGGCGGGG CTAACTAGGC
110581 AGAGCAGGGC TGTAGGTGGG CTATAGCTGT GGGCGGGGCC ATGGGCGGGG CCGACTAAGC
110641 AGAGCAGGGC TGTAGGTGGA CTATAGCTGT GGGCGGGGCC ATGGGCGGGG CCGACTGTAG
110701 GCAGAGCAGG GCTATGGGCT GACTGTGGGC GTGGTGAGGG TGCCGTAGAG CATGCTAATG
110761 ACCAGGGCGT GGTCATAGCA GGGTAGGGTC TTGGGTGCTC CTGGGGCTGG GGGGCTTCTC
110821 CACATGCTCC CCACACCTTC AGGAGTCGCC CTGCTGCGTC ACGCACCACA CGGCGCTTGT
110881 CCTCCAGCTT TGGCTCTGGC CGCTGCCTCC TTTGGTCACA TGACCGTATA ATCGGCCTCC
110941 CCTCTGAGAC CCTGGGCTGG ACCCCCGGCC TCCCTCTGCC TCCCCAGGCT CAGATATTCA
111001 CCCGGAGGGA GAAAGGACAT GTGTCCCCCA TGCCCACACA TCCCCAGCTA CAGGCAGCTG
111061 GGGAGGACGG GTTCTAGGAT GGCCATGTTA CAGCTGAGGA TGCAGAGGGG TTGGGTGATG
111121 GGTCTGCACA GCCACGGCGG GACAGGTGTC TCTGGACCCT CTCCCCAAGG TTGGCCCTGC
111181 CGGGGCCCTG GCTGGCTGGT GCTGGGTAAT GTGCCCTGTC CCAGGAGCAG GGCCGGCCTC
111241 AGGGTCCTGA GCTCCAGGGC ACTGGGGAAG TCCTGGCTCC ATGAGGGCAG GACGGGCCCA
111301 GGACAGACCA GGGTGTTCTC CCCAGGTGGA CCCCAAGCCC TTCTACGAGG CCTGTGTGCA
111361 CGACTCGTGC TCCTGTGACA CGGGTGGGGA CTGTGAGTGC TTCTGCTCTG CCGTGGCCTC
111421 CTACGCCCAG GAGTGTACCA AAGAGGGGGC CTGCGTGTTC TGGAGGACGC CGGACCTGTG
111481 CCGTAAGAGC CTGCCCGAAC TGCACTCAGG GCCGGGACGG GGGCTGGGAG GTGCTGTATT
111541 GCGGGCCGGG GTGACACTCC TTGTCCATCC AGGTGATGGG TGTGCATCAC CCACCCTTTC
111601 CCCGACTTCT CCAGTGTCCT TCTTTGGGGC CCTGTGGGAC CCGGGTTGGC AGAGCAAGCT
111661 TGATGCGTCT GCGTCCCAGC CCCCGACCCC AGATTCGCCC TCACCCCGGC CCAGGCCTGA
111721 GCCCTCCTGC GTCTGACCCT GGCCCTGTCT CCCCCAAGCC ATATTCTGCG ACTACTACAA
111781 CCCTCCGCAT GAGTGTGAGT GGCACTATGA GCCATGTGGG AACCGGAGCT TCGAGACCTG
111841 CAGGACCATC AACGGCATCC ACTCCAACAT CTCCGTGTCC TACCTGGAGG GTGAGCAGGG
111901 TGGGGCGGGC TTCAGCGGGG GTGATGGCCG AGGGGCCTGG AGGCTGAGTG GGGCAGCCCT
111961 CGGGAGAGGC AACAGTCCAC TGGCCTGGAG GGTGAGCCAG GCGGCCCTCG GGGGAGGCTA
112021 CGGCCGACGG GCCTGGCACT GTGGGGCTGA AGGCTGATGT CTGGAGACCC ATGGGGACAC
112081 CCGGAGGGAG GCCTGACCCT CAGGGTACCC ACAGCCCAGG GCAGCCAGGC TCCCCTTGCT
112141 GCAGGATCAG GAGGGAAGCA GGCTATCGTG GAAACTGGGA GTGGCAGGGG TGGGAGGTGC
112201 TGAGGTTCGT GCAGAGCAGG GCGGGTTGGG GAGCATTTCA GGCACAGGTC AGGGGAGGCC
112261 CCTGCCGGGT GCTGGTGTCT GAGCTGAGAA CCAGTGACGT GAAGGAGGGA CTGGTGGGAA
112321 GTTTGGGAGG AGTATCCCGC CATGGGAGAG GAACATGGGT CTTGGGACTC AGGGCTGCTC
112381 GGGGGGCCCG ATGAGACTGG GCAGGGCTCC TCAGCAGGCA GCGTTCAGGG CTCAGTGGGG
112441 TGGGGAGATC CAGGCCCTGC CTTTCCAATC CCCGGCCTTC CCAGAGGGGC ATCCTGCAGA
112501 GAAGGGCCTG CCAGGGTAGG GACGGTGGGT GGGGTGTGGT GGACTGCGGT GGTCCCAACC
112561 CTATGCCCTG TGTCCACCAG GCTGCTACCC CCGGTGCCCC AAGGACAGGC CCATCTATGA
112621 GGAGGATCTG AAGAAGTGTG TCACTGCAGA CAAGTGTGGC TGCTATGTCG AGGACACCCA
112681 CTACCCACCT GGAGCATCGG TTCCCACCGA GGAGACCTGC AAGTCCTGGT ACCTAAGCCC
112741 ACGTGGCAGG GGGCCTGGGG GAGCTGCACA TATGGGCACA TGAGTACACA CACACGTGTG
112801 AGCACACAGT GTACACAGTA CACAGACACA CAACCGTTCC ACATGGGTGC ACATGCACAC
112861 AAACGCACAC AGCATACCAC GTGCACACAC ACGGTCACAT GCATGCATGG TGCACACATG
112921 CACACATGAA TGGATGCCAA CATGCAGGCA CACACAGTCA CACATGCACA CAGCGCACAC
112981 ATGGACACAT GCCTAGACGC AGATACCCAG GCATACACTC ACGGTTACAC ACTCACGCAC
113041 ATATGCATGG ATGCAGACAC GCAGGCACAC ACGGTCATAT AGTCATACAC CACATGCACA
113101 CATGCACAGA CAGACACCCA GGCACACACA GTTACACAGT CACACATGCA CACATGCATG
113161 GATGCAGACA CGCAGGCGCA CACACACATG CACAGTGCAC ACGTACACAT GCCTAGACAC
113221 AGATACCCAG GCACACACAG TCACACATGC ATGGACACAG AGTCACATGT GCACACATAC
113281 ACACGTGTGG ACAGACATAG GCACAGTCAC GTGCACACAT GCACTCACAC TCAGTCACAC

113341 ATGAACATGT GCTCACATGC ATGGACACTG ACACGCAAGG ACACACAGTC ACACATGCAC
113401 ACATGCATAG ACACAGACAC CCAGGCACAC ACAGTTACAC AGTCACACAT GCATGGATGC
113461 AGACACGCAG TCACACAGTC ACACATGCAC ACACTGCACA CATGTACACA TGCCTAGACA
113521 CAGATATGCA GGCACACACA CATAGTCAAA CATGCACACA TGCATGGACA CAAAGTCACA
113581 CGTGCACACA TGCACACATG CATGGACAGA CACAGGCACA CACAGTCACG TGCACAGATG
113641 CACTCACAGT CACACATGAA CACATGCTCA CATGCACAGA CACTGACACG CAGGCACACA
113701 CAGTCACACA TGTACACGTG CCTAGACACA GATACCCAGA CACACACAAT TACACAGTCG
113761 CACAGTCACA CATGCATGGA TGCAGACACA CAGGTACACA AGGTCACACA GTCATATAAT
113821 GCACACATGC ACACATGCAT AGATACAGAC ACCCAGGTAC ACACTCACGG TGACACAGTC
113881 ACACATGCAC ACATGCATGG AGGCAGACAC ACAAGCACAC ACAGTCACAC AGTCACACAT
113941 GCCACACACC ACCACCACGG TGACCCCAAC CCCAACACCC ACCGGCACAC AGACCCCAAC
114001 ATCGACACCC ATCACCACCA CCACCACGGT GACCCCAACC CCAACACCCA CCGGCACACA
114061 GACCCCAACC ACGACACCCA TCAGCACCAC CACCACGGTG ACCCCAACCC CAACACCCAC
114121 CGGCACACAG ACCCCAACAT CGACACCCAT CACCACCACC ACCACGGTGA CCCCAACCCC
114181 AACACCCACC GGCACACAGA CCCCAACCCC GACACCCATC ACCACCACCA CCACGGTGAC
114241 CCCAACCCCA ACACCCACCG GCACACAGAC CCCAACATCG ACACCCATCA CCACCACCAC
114301 CACGGTGACC CCAACCCCAA CACCCACCGG CACACAGACC CCAACCCCGA CACCCATCAC
114361 CACCACCACC ACGGTGACCC CAACCCCAAC ACCCACCGGC ACACAGACCC CAACCCCGAC
114421 ACCCATCACC ACCACCACCA CGGTGACCCC AACCCCAACA CCCACCGGCA CACAGACCCC
114481 AACATCGACA CCCATCACCA CCACCACTAC GGTGACCCCA ACCCCAACAC CCACCGGCAC
114541 ACAGACCCCA ACCACGACAC CCATCACCAC CACCACCACG GTGACCCCAA CCCCAACACC
114601 CACTGGCACA CAGAGTACAA CCCTGACACC CATCACCACC ACCACCACGG TGACACCAAC
114661 CCCAACACCC ACCGGCACAC AGACCCCAAC ATCGACACCC ATCACCACCA TCACTACGGT
114721 GACCCCAACC CCAACACCCA CCGGCACACA GACCCCAACC CCGACACCCA TCTCCACCAC
114781 CACTACAGTG ACCCCAACCC CAACACCCAC CGGCACACAG ACCCCAACCA TGACACCCAT
114841 CACCACCACC ACCACGGTGA CCCCAACCCC AACACCCACC GGCACACAGA CCCCAACAAC
114901 GACACCCATC AGCACCACCA CCACGGTGAC CCCAACCCCA ACACCCACCG GCACACAGAC
114961 CCCAACATCG ACACCCATCA CCACCACCAC TACGGTGACC CCAACCCCAA CACCCACCGG
115021 CACACAGACC CCAACCACGA CACCCATCAC CACCACCACC ACGGTGACCC CAACCCCAAC
115081 ACCCACCGGC ACACAGAGTA CAACCCTGAC ACCCATCACC ACCACCACCA CGGTGACACC
115141 AACCCCAACA CCCACCGGCA CACAGACCCC AACCCCGACA CCCATCTCCA CCACCACTAC
115201 GGTGACCCCA ACCCCAACAC CCACCGGCAC ACAGACCCCA ACCATGACAC CCATCACCAC
115261 CACCACCACG GTGACCCCAA CCCCAACACC CACCGGCACA CAGACCCCAA CAACGACACC
115321 CATCAGCACC ACCACCACGG TGACCCCAAC CCCAACACCC ACCGGCACAC AGACCCCAAG
115381 ATCGACACCC ATCACCACCA CCACTAAGGT GACCCCAACC CCAACACCCA CCGGCACACA
115441 GACCCCAACC CCGACACCCA TCACCACCAC CACCACGGTG ACCCCAACCC CAACACCCAC
115501 TGGCACACAG GCCCCAACCC CAGCAGCCAT CACCACCACC AGTACGGTGA CCCCAACCCC
115561 AACACCCACC GGCACACAGA CCCCAACCAC GACACCCATC ACCACCACCA CCACGGTGAC
115621 CCCAACCCCA ACACCCACCG GCACACAGAG TACAACCCTG ACACCCATCA CCACCACCAC
115681 CACGGTGACA CCAACCCCAA CACCCACCGG CACACAGACC CCAACATCGA CACCCATCAC
115741 CACCACCACT ACGGTGACCC CAACCCCAAC ACCCACCGGC ACACAGACCC CAACCCCGAC
115801 ACCCATCTCC ACCACCAGTA CGGTGACCCC AACCCCAACA CCCACCGGCA CACAGACCCC
115861 AACCATGACA CCCATCACCA CCACCACCAC GGTGACCCCA ACCCCAACAC CCACCGGCAC
115921 ACAGACCCCA ACAACGACAC CCATCAGCAC CACCACCACG GTGACCCCAA CCCCAACACC
115981 CACCGGCACA CAGAACCCAA CATCGACACC CATCACCACC ACCACTACGG TGACCCCAAC
116041 CCCAACACCC ACCGGCACAC AGACCCCAAC CATGACACCC ATCACCACCA CCACCACGGT
116101 GACCCCAACC CCAACACCCA CTGGCACACA GGCCCCAACC CCAACAGCCA TCACCACCAC
116161 CACTACGGTG ACCCCAACCC CAACACCCAC CGGCACACAG ACCCCAACCA CGACACCCAT
116221 CACCACCACC ACCACGGTGA CCCCAACCCC AATACCCACC GGCACACAGA GTACAACCCT
116281 GACACCCATC ACCACCACCA CCACGGTGAC ACCAACCCCA ACACCCACCG GCACACAGAC
116341 CCCAACCCCG ATACCCATCT CCACCACCAC TACGGTGACC CCAACCCCAA CACCCACCGG
116401 CACACAGACC CCAACCATGA CACCCATCAC CACCACCACC ACGGTGACCC CAACCCCAAC
116461 ACCCACCGGC ACACAGACCC CAACAACGAC ACCCATCAGC ACCACCACCA CGGTGACCCC
116521 AACCCCAACA CCCACCGGCA CACAGACCCC AACATCGACA CCCATCACCA CCACCACTAC
116581 GGTGACCCCA ACCCCAATAC CCACCGGCAC ACAGACCCCA ACCACGACAC CCATCACCAC
116641 CACCACCACG GTGACCCCAA CCCCAACACC CACTGGCACA CAGGCCCCAA CCCCAACAGC
116701 CATCACCACC ACCACTACGG TGACCCCAAC CCCAACACCC ACCGGCACAC AGACCCCAAC
116761 CACGACACCC ATCACCACCA CCACCACGGT GACCCCAACC CCAATACCCA CCGGCACACA
116821 GAGTACAACC CTGACACCCA TCACCACCAC CACCACGGTG ACACCAACCC CAACACCCAC
116881 CAGCACACAG ACCCCAACCC CGACACCCAT CTCCACCACC ACTACGGTGA CCCCAACCCC
116941 AACACCCACC GGCACACAGA CCCCAACCAT GACACCCATC ACCACCACCA CCACGGTGAC

117001 CCCAACCCCA ACACCCACCG GCACACAGAC CCCAACAACG ACACCCATCA GCACCACCAC
117061 CACGGTGACC CCAACCCCAA CACCCACCGG CACACAGACC CCAACATCGA CACCCATCAC
117121 CACCACCACT ACAGTGACCC CAACCCCAAC ATCCACCGGC ACACAGACCC CAACCACGAC
117181 ACCCATCACC ACCACCACCA CGGTGACCCC AACCCCAACA CCCACTGGCA CACAGGCCCC
117241 AACCCCAACA GCCATCACCA CCACCAGTAC GGTGACCCCA ACCCCAACAC CCACCGGCAC
117301 ACAGACCCCA ACCACGACAC CCATCACCAC CACCACTACG GTGACACCAA CCCCAACACC
117361 CACCGGCACA CAGTCCCCAA CCCCAACAGC CATCACCACC ACCACTACGG TGACCCCAAC
117421 CCCAACACCC ACCGGCACAC AGACCCCAAC ATCGACACCC ATCACCACCA CCACTACGGT
117481 GACCCCAACC CCAACACCCA CCGGCACACA GACCCCAACC CCGACACCCA TCTCCACCAC
117541 CACTACGGTG ACCCCAACCC CAACACCCAC CGGCACACAG ACCCCAACCA CGACACCCAT
117601 CACCACCACC ACCACGGTGA CCCCAACCCC GACACCCACC GGCACACAGA CCCCAACCAC
117661 GGTACTCATC ACCACCACCA CTACGATGAC CCCAACCCCA ACACCCACCA GCACAAAGAG
117721 TACAACCGTG ACACCCATCA CCACCACAAC TACGGTGACC GCAACCCCAA CACCCACCGG
117781 CACACAGACC CCAACCATGA TACCCATCAG CACCACCACT ACGGTGACCC CAACCCCAAC
117841 ACCCACCACT GGAAGCACGG GGCCCCCCAC CCACACAAGC ACAGCACCCA TTGCTGAGTT
117901 GACCACATCC AATCCTCCGC CTGAGTCCTC AACCCCTCAG ACCTCTCGGT CCACCTCTTC
117961 CCCTCTCACG GAGTCAACCA CCCTTCTGAG TACCCTACCA CCTGCCATTG AGATGACCAG
118021 CACGGCCCCA CCCTCCACAC CCACGGCACC CACGACCACG AGCGGAGGCC ACACACTGTC
118081 TCCACCGCCC AGCACCACCA CGTCCCCTCC AGGTAAGCAG AGCTGCTTGG TTCCTCTGGC
118141 CTGGGATGCT TCTTCCTCCC CTTGTGCCGG GCAGGACTGT CCCAGGAAGG CTCAAGGCAC
118201 GTTCTGGGCG CCTCTCTGCC CACGAAGCTT GGTCACTGTG TGGGCAGAAG CCACTGACAC
118261 TGGCCAGTGC TGGGCAGTGA AGCCAAAGGC CATTCCGCTT GCCCATAGGA CAGCCTTCTG
118321 AGGAGCTGCT GACACCGGCC AGTGCTGGGC AGTGGAGCCC TTGGCTATCC TGCTCGCCCA
118381 TAAGACGGCC TTCTTCAGGG GCCCACTGCT ATGTGATGCG GTGCTGTGGG AGCCCATCAA
118441 GGCTGGGGGG CAGAGAGAGG CTGCCAGTGA GGTGCCTGCG GGTCCACCTG CTTCTGGCTG
118501 CAGCCCCTCC TTGGGGCCTT TTCCTGGTGG ACGGCGTGCC ACAGCCAGTG CCTTCTGGAC
118561 GCCTCTTGCT GGCCATCGGC TTGGCCAGCA AGCTGTGTTG CTGCCAGAGC ACCAGGTCAC
118621 CTGCAGGCTC TCGTGACACT CGGCTGTGGT GATACTGGCC TTGCCGCTCC ACCCTGCCTG
118681 GTGACTCTGA GAGCCTGGGA GGTGGGCACG AGGCCCTGGT CCTCCAGTTC TGCCACCCGG
118741 TCGGCTGTCT GGCTCCCTTG CAGCTGGGGA GTGGCAGTTG GGACCCTGTG GCATCTGAGA
118801 TGTGCAATGT CTCAGCCCTC ACTGGTGTCT CCTGCTCTCA CAGGCACCCC CACTCGCGGT
118861 ACCACGACCG GGTCATCTTC AGCCCCCACC CCCAGCACTG TGCAGACGAC CACCACCAGT
118921 GCCTGGACCC CAACGCCGAC CCCACTCTCC ACACCCAGCA TCATCAGGAC CACAGGCCTG
118981 AGGCCCTACC CTTCCTCTGT GCTTATCTGC TGTGTCCTGA ACGACACCTA CTACGCACCA
119041 GGTACTCAGG CTGTTCACAT CCTGTGCTTG GGTGGCCGAG GCTGGCCCCG GCATGTACCA
119101 ATGGGTCAGG TGCCAGGGCT GAGATCGCAG TAGAAGCGTC TCAGGAGGCA GCAGCCGTCG
119161 AGGGTGGCTG TGTCCAGGGC ACGGCTTCCC TTGGGTGGCC TCTGTGGGGA CCTCCGCTGT
119221 GGGGACCTCC ACGGGGTCCA GCGGCTAGCC CTGCCTCCGG ATAGCCCTGC CTCTGGACGG
119281 TGTGATCGTG GGTCTGTCTC CCTTCGCAGG TGAGGAGGTG TACAACGGCA CATACGGAGA
119341 CACCTGTTAT TTCGTCAACT GCTCACTGAG CTGTACGTTG GAGTTCTATA ACTGGTCCTG
119401 CCCATCCACG CCCTCCCCAA CACCCACGCC CTCCAAGTCG ACGCCCACGC CTTCCAAGCC
119461 ATCGTCCACG CCCTCCAAGC CGACGCCCGG CACCAAGCCC CCCGAGTGCC CAGACTTTGA
119521 TCCTCCCAGA CAGGTCAGTG GGCTGCAGGC GGCTTTGTCC CCATGGCACT CTGCGCAGCA
119581 TGTCCGGGCA GCTGAGGCCC CAGGCACCAC TTCCTGCTGG TCGTCTGAGG GCCGAGGCCT
119641 CCAGCAACCC TTGGGTGCAG GGTCTGCCGA GCCCTCCACA TTTTCACCGT GCCCCGCTGT
119701 GCCTGGCGAG GTGGCTGGCT GCAGTGAGGT CCGTGGAAGC CACTTCGGCC TCCAGCCTCC
119761 CGGCTCAGCA CCCGCCCCTC CTGAGCGCAG ACCACCCCAT CCTGTGCCGG TCCCCCTGAC
119821 GTCCCTTGCC TCCCGTCCCC AGGAGAACGA GACTTGGTGG CTGTGCGACT GCTTCATGGC
119881 CACGTGCAAG TACAACAACA CGGTGGAGAT CGTGAAGGTG GAGTGTGAGC CGCCGCCCAT
119941 GCCCACCTGC TCCAACGGCC TCCAACCCGT GCGCGTCGAG GACCCCGACG GCTGCTGCTG
120001 GCACTGGGAG TGCGACTGTG AGTCCGGGGC CCCCAGGCCC TCCCCGCATC TCCTGCCCTC
120061 TCCGTGGGTG GGGGCTGCAG GGCCCGTCTC CCGGGGGCGG AAGGGCTGAG GCTCCTTGGG
120121 CACAGATCCC ACTGAGGTGT TCGCTGAGGC TGGGTGACTT CTGAGGGTCT TCTCACAGCC
120181 CTGCTTTTGC CTCATTGGGT GGGGAGGGCC TGGGCAGGTG GAGGGCTTGC CTGGTGGAGT
120241 TAGGGCTCCT CCCTGGAACA AGGGTGCTTC TGAGGCAAGA GGGGGCTGAG TTGAAGTTTG
120301 AACCCTGGTC CGTCCTGCAG AATGGGCCAC TGTGGGTGCG CCAGGGCAAG TGCAGCTCAG
120361 ACATCCCCGT GCCCACGCAC AGGAGTGGGG TTTTCAGGCC CCAGCTTCCT GCTGGCTCTT
120421 CCTGACTATG CCCCAGCCCA GCCCTTGCAC CCGACCCCGG CCGAGGGGCA CAGGTGGCAC
120481 GGCTCACTCC GGCTCCCTTG CAGGCTACTG CACGGGCTGG GGCGACCCGC ACTATGTCAC
120541 CTTCGACGGA CTCTACTACA GCTACCAGGG CAACTGCACC TACGTGCTGG TGGAGGAGAT
120601 CAGCCCCTCC GTGGACAACT TCGGAGTTTA CATCGACAAC TACCACTGCG ATCCCAACGA

120661 CAAGGTGTCC TGTCCCCGCA CCCTCATCGT GCGCCACGAG ACCCAGGAGG TGCTGATCAA
120721 GACCGTGCAT ATGATGCCCA TGCAGGTGCA GGTAGGCACA GCGTGGCCAC AGGAGGCTGG
120781 CATGGAGGCG GGTGCTGACA TGGGCCCCAA TGCACCCTGG TTCCCCAGGG GCCAGAGGAC
120841 TGGGCTGTGG GGGTGCCAAG GCATAGCCTC TCCTAGAGCT GGGCTAGAAG GTAGGATGGG
120901 GTGGGCGACT GGCTCCGGGA CATATCAGCT CTTCCTGCAG GCCCTCCAGG TGTGTCCTGG
120961 GCCCCTCGAG CCCTGGCACC ATGCCACGCT GGGCACAGTC TCTGCAGCAG AAGCTGCCTC
121021 CTGAGGACAG AGTCAGGGAC AGGGCTCTGC ACACCCTTGG CTGAGATGCC CCTACTTGCA
121081 GGGGAATCAT TGGTTCTGAG GCTCAGGAGG CCCCGGGAGC CTGCGCCGGG CTCCACAGTC
121141 CCCAGGTGCT CCCAGGAGAG CTCCTTCACT GGCTCACCCA TGGGACCAGG GTCTGGTTGG
121201 GAGCAGTGGA GTGGAAGCAA GAAAGGGGGC AGGAAAGCGG GGTAGGCAGG GCCCTCTCCC
121261 TACATGTGTA GGTCAGAGAG CAGGCGGGGT GGGGCAGCCC TGGAGCTCTC ACAAGGAGAG
121321 GACCGAGGCA GCTGCAGCTC CCATGGTGTG TCGGCCACAG GTGCAGGTGA ACAGGCAGGC
121381 GGTGGCACTG CCCTACAAGA AGTACGGGCT GGAGGTGTAC CAGTCTGGCA TCAACTACGT
121441 GGTGGACATC CCCGAGCTGG GTGTCCTCGT CTCCTACAAT GGCCTGTCCT TCTCCGTCAG
121501 GCTGCCCTAC CACCGGTTTG GCAACAACAC CAAGGGCCAG TGTGGTGAGT TCCGTGACCC
121561 CCATGGCCCC CGAGGCCCCC ACGGCTCCCA CCGTCCCCTG TGCCCCCATG TCCTGCCCCA
121621 GGGCGGGTGG CCAGGCCAGG CTGAGGCTGA GGCTGCGTGT AAACACCCAT GGGCCTGGCT
121681 GTGGGCCTCT TGCCCCGCTG CTCGGGGCTG CTGTGGCCAT CACCCGGGTT CAGTCTCTGT
121741 GAGGAGCCAA CAGGAGGGGG CCTGGCCTGG TCTCTGCCCT CGGCCCTGGC TGGCCGGTCC
121801 TGGGCATCTG GGCTGGAGAA GGGCAGGGCT TACCCTGTCT GCAACGTGGC CTCTCTCACT
121861 GATACAGGCA CCTGCACCAA CACCACCTCC GACGACTGCA TTCTGCCCAG CGGGGAGATC
121921 GTCTCCAACT GTGAGGCTGC GGCTGACCAG TGGCTGGTGA ACGACCCCTC CAAGCCACAC
121981 TGCCCCCACA GCAGCTCCAC GACCAAGCGC CCGGCCGTCA CTGTGCCCGG GGGCGGTAAA
122041 ACGACCCCAC ACAAGGACTG CACCCCATCT CCCCTCTGCC AGCTCATCAA GGACAGGTGA
122101 CCCCGCCCAG GCCTGCCTGT GGCCACGACA CCAATAAGCT GAGGGCCTCT GTGCCCCAGC
122161 CCCCAGCTCT TGCAAAGAGG AAGGAGGCAG CGCGTGGGGC CTGGCGCTGG GGCTGGGAAG
122221 GCACGGAGCC GCGGAACCAG GATCAGGCGC TAGGTCGCCG TGGGGTCCAG GACCCAGGCC
122281 CTTGGGTTCC ACGGGGCTGA GCTGCTACGT GCGGCCTGTG CCTTTGCTGA ACTCCAGTCT
122341 CTCCTGGCTC CCGGGAAGGT GCAGGGCTGG CCGAGTGTGA GGCCCGGAGT AAACCAGTCA
122401 ACCCAGGACA GAGCTCAGGG CTGATATTGG GAGGGCAGAT TTGGGCTTTG ACAGAGAGGG
122461 GGTGCTCCTA ACGCTGGCAG TCATGGGGGG TCAGCATCCT GTCCCTGGAA GTATAGGGGC
122521 CAGGTATAGG CTGGGTGTCC ATCTGCCAGG GTTGCTGGAG GGGGTCCTGA AGCTGATGAC
122581 CACATAGACG TGGTTTCTAT CTCTGGGAGC CGGGCTGCAG AGCCACCTTG CTCGGCCATC
122641 CCTTGGTCTG TCCCTGAGCT GTCCCCCTGG CTGGCCTGTC CCTTGACCCT CCATCAGCCA
122701 CAGGCGCCTC TCTGGCGGGT GCCGGACTCC AGGAGGACAG TCCGGGCAGA GACGCTGGGG
122761 TAGAGAGCAG GGGAGAGGCA GGTGCCACCT GAGTGTGACC TGTGCCTCTC CCTGCACAGC
122821 CTGTTTGCCC AGTGCCACGC ACTGGTGCCC CCGCAGCACT ACTACGATGC CTGCGTGTTC
122881 GACAGCTGCT TCATGCCGGG CTCGAGCCTG GAGTGCGCCA GTCTGCAGGC CTACGCAGCC
122941 CTCTGTGCCC AGCAGAACAT CTGCCTCGAC TGGCGGAACC ACACGCATGG GGCCTGCTGT
123001 AAGTGCCCAT CTGCCCCTGC CCTGGAGCTG GGGGCCTGCA GGCCAGACGT GGTCTCTAGG
123061 CTCTGCCAGG TGCTGTGCCC AGCCTGAAGC TAGACCTAGA TGGGCTGCGG CCAGGGATGC
123121 AGAGATGGCG GGTGTGAGAC CAGGGCTGGG GCCATGGGGT GGGGAAGGCC AGGCTGGAGG
123181 GGCTGAGGTG CTGGGGCTTC TGCCAGCATC GCTAAATGCA ACTGGGTGCC CACCACCCAG
123241 CTCGGGACAA CCTCGAGGGT GGAGGTTGAT GCCCAGGCAG CTGGTCACCC TCCTCCGTGT
123301 GTGGGGCACT GGGCAGCTGT CACTCAAGGG GGTCCAGGCT CCTCCGCCTG ACATGAGGCA
123361 GCCCTCTGAC CTCTGCCCAT GTCCCTCAGT GGTGGAGTGC CCATCTCACA GGGAGTACCA
123421 GGCCTGTGGC CCTGCAGAAG AGCCCACGTG CAAATCCAGG TATGTTGTTT GAGGGTCCAC
123481 CAGGACCGTG GGCTCGCCTT CTGCAGTGCG GAGGGTGGCA TCATCTGGGC ATAGCAGTCC
123541 CACCTGCCAG CTCCCCAGCC CCACCCCACC TGTCTGACAA TGCCCTCCCG CCCCCAGCTC
123601 CTCCCAGCAG AACAACACAG TCCTGGTGGA AGGCTGCTTC TGTCCTGAGG GCACCATGAA
123661 CTACGCTCCT GGCTTTGATG TCTGCGTGAA GACCTGCGGT ACGCCACCCA CTCACACTGT
123721 CCCCTCCTGC CTCCCTCCTG CCTCCTCCTG GGTGTCCACG GAGGCTGGGA CCAGGACGCT
123781 GACCACCCCC CACCTCTGAT CCCTGTTGCA CAAGGACTCT GCTAACACAA CTTGTCTCCT
123841 GGGTGTCCAT GGAGGCTGGG ACCAGGAGGC TGACCACCCC CACCCCTGCT CCCTGCTGCA
123901 CAAGGACTCT GCTAACACAA CTTGTTTCTT CCCTCTTCCT AGGCTGTGTG GGACCTGACA
123961 ATGTGCCCAG AGAGGTAGGC CCCACCGTGT TGCTGGGGGA TCCTTCCACA AATTCTGAAT
124021 TCTGGGGAGT GAGGGATGGA CATGAAAACC TGGAGCCTCA AAGATTGAGG AATGAGGTCA
124081 TCTAAGTCCT GGATGGCTGA GTTGGCATGG ACACCACCCA CTCACCCACC CATCCTTCCA
124141 CCCACCCACT CATCCACCTG TGCACCCATC TACCCACTCA CCTACCCCTC CATCCTTCCA
124201 CCTACCTAGT CATCACCCAC TCATCTATGC ACCCCCACCC ACCCACTCAT CCATCCATCC
124261 ATCCACCATC CACCTACCCA ACCATCCACC CATCCATCCA CCATCCATCT ACCATCCACC

124321 ATCCACCCAA CCATCCACCA TCCATCCATC CACCATCATC CATCTACCAT CCACCCACCC
124381 ACCTATCCAT CCATCCATCC ACCATCTGTC TACCATCCAC CCACCCACTC ATCCATCCAT
124441 CCATCCACCA TCTGTCTACC ATCCACCCAC CCACCTATCC ATCCACCCAT CCATCCATCC
124501 ATCCATCCAT CCATCCATCC ATCCATCCAC CCACCATCTG TCTACCATCC ACCCACCCAC
124561 CTATCCACCC ATCCACCCAC CCATCCATCC ACCCAACCAT CCACCATCCA TCCATCCATC
124621 CATCCATCCA CCATCCATCT ACCATCCACC CTCCCATCCA TCCACGCATC CACCCAACCA
124681 TCCATCCATC CATCCACCAT CCACCCACCA TCCACCCATT TATCCATCCA TTCTCCCTCC
124741 CTCCATTCAC CACCCATTGG TCATATGATA CTCTGTCTAG AAGCTCTGAC ATGACATCTT
124801 GGCCACCTCT GTGCTGCCCA TGCCTCCTAC CTGTGGTAGC AGCCATGTGG ATGATTCCTT
124861 AGCTAAATTC TGTACAAACC TGAGAGGCCT GAGTGGAGAA TTTGCCACGT GCCAAGCCCC
124921 TGCTTGTCGA TGCTGGTGAG CAGGTAATGG CTTTGTGATA TCAGTGAATG AGCAGCTACT
124981 GTCCTATCCC AGAACCTGCC TGGTGTGCTC AGAAGTGAGG AGGGACATGG TTTTCCCCCA
125041 GGATCCCTCA GCACTCTGCT CAGGGTGGCT GTTTCTCCCC GCTGACCACA GCTGCAGCTC
125101 CGGGGCTGTG GTGAGGTGGG GCCTGCCTGG TGCCACCTGT CCTCTCTACT CACCCTTCTT
125161 TCCCTGCAGT TTGGGGAGCA CTTCGAGTTC GACTGCAAGA ACTGTGTCTG CCTGGAGGGT
125221 GGAAGTGGCA TCATCTGCCA ACCCAAGAGG TGCAGCCAGA AGCCCGTTAC CCACTGCGTG
125281 GAAGACGGCA CCTACCTCGC CACGGAGGTC AACCCTGCCG ACACCTGCTG CAACATTACC
125341 GTCTGCAGTA AGGCCATCCC CTGGGGCCCA TGCCACCTCT CAGGGGTGCA CACATCCCTG
125401 TAGGCTGGGC TGCCTGCTGT CCCCTCCTTG GCAAGTGAGG AAACAGCTGG CTTGGGGGCC
125461 TCTGCTGTGC CCCTTGAGAG GGCTTGGGAG GGGGCCGCTG GGCCCAGTCC AGGCATCCCT
125521 GCTGCAGGGC CTGACCTGGG TGGGGAGGGG ACCCTTGGAG GTGCTGGAGG CCCGACCCTG
125581 TGCAGTGGCC CCGGGGGCTT TGCCTGGGAG GAGCCACCCT CACGGCCGCG TGCGCACCCT
125641 GTCTTCAGAG TGCAACACCA GCCTGTGCAG TGGCCCCGGG GGCTTGGCCT GGGAGGAGCC
125701 ACCCTCACGG CCGCGTGCAC ACCCTGTCTT CAGAGTGCAA CACCAGCCTG TGCAAAGAGA
125761 AGCCCTCCGT GTGCCCGCTG GGATTCGAAG TGAAGAGCAA GATGGTGCCT GGAAGGTGCT
125821 GTCCTTTCTA CTGGTGTGGT AAGCAGGGCT GGTGGGCAGG GCAGGGAGGA GGCTGCCGCC
125881 CGGGGTGGGG TGGCTGTAAG GGGGTTGGCT CCCTCCTGGG GGTCTCAGAT TCTGGGGACA
125941 CAGATGGCTG TACGCTTGGC TGATGCACCC ACCCCAGCCC TGAGCGCTCG CTCCATCCAC
126001 TGGGTGTGCA CCGGGAGTGG GGGTCTGGCC AGGTGGCCGC CCCGGGGCAG TCTCCAACGA
126061 ACGGCCTTCT CCGTTCTTTC TCCCAAGAGT CCAAGGGGGT GTGTGTTCAC GGGAATGCTG
126121 AGTACCAGGT GAGCCCTGGG CTGGGTGAGA GGGAGGAGGG GAGGAGGTCG GCTGCAGCGT
126181 GGGGGTCCTG GCAGGCTGTT GGGCTGGCTG GGATGCTGGA GAGGCCCCTG CCTCATGTCT
126241 CTCCCTGTGC CCGAAGCCCG GTTCTCCAGT TTATTCCTCC AAGTGCCAGG ACTGCGTGTG
126301 CACGGACAAG GTGGACAACA ACACCCTGCT CAACGTCATC GCCTGCACCC ACGTGCCCTG
126361 CAACACCTCC TGCAGCCCTG TAAGCGGCCA CCCTCCTCCT TCAGCCTGCC CTTTTCCCTC
126421 CTCCCAGACA AGCACCCGGG CCCATGTCTG CATCGTGACC CTTTCTTTCC TCCTTTCAAC
126481 GCCAACCTGT CCCTGTCCCC ACCTCTCCAT CCTGACACCT GCCCAGCCTG GGGCCTCCTC
126541 CAGGTGGGGG GGTCTCGGCA GCCCTGCAGG CTTTGTGTGG TGTGGGGTAC AGCCTGGGAG
126601 TTCAGTTGCA GTGGCGTGTC TATGTGCGCA GGGCTTCGAA CTCATGGAGG CCCCCGGGGA
126661 GTGCTGTAAG AAGTGTGAAC AGACGCACTG TATCATCAAA CGGCCCGACA ACCAGCACGT
126721 CATCCTGAAG GTAGGTGTGC ACTGCCGGCC CCGACGCGGC CGGGTTGCTT GAGCCCAGGG
126781 CAAGGCGCGG GCCACCCAGG ATCCCCCAGC TGAGTCCTCC CAGTCCTGGG CGCAGCTGTG
126841 ATGGGCGCCC TGGGGCTGCC ATGACAAATG AGCAGGCGTC TTCAGGGCAG AAAGGGATTC
126901 TCCTGGTTCT GCGGCCCAGA AATCCATAGA GCAAAGGGCC TCAGGGCTGT GCTCCCTCGG
126961 AGGCGCTAGG CAAGGACCTT TCCCAGCCTC TGGTCACTCT AGGTGCCCCT TGGCTGTGAC
127021 CACGAGGTTT CCTTCCCTGT GTCTGCCTCT CCTCTCCCTT TTAAGGATTT AGGCACCCCA
127081 AGCAGGATGA TCTCATCTTA GGATCCTTCA CTTAATGACA CCTTCAAAGA CCCCCTTTCC
127141 AAGGCAGGTC ACATTCATAG ATTCAGAGTT AGAACACAGA CAGACCTTTG AGGGTTGTGT
127201 GGGCTCCAGG CTGGTGCCTG ATGTGGGGCC CCGCCCATGT CACTTGTCCT GTGGCCCTGG
127261 GCCTCACCAG GAAGCCTCCC CGGCCAGGTG TCTCCAGGGT GTCTTCCTGG CCGGGCTGGG
127321 GCTGGGCCTG CTGCCCTCCC TCACCAGAGC TCCCTGCCCC ACAGCCCGGG GACTTCAAGA
127381 GCGACCCGAA GAACAACTGC ACATTCTTCA GCTGCGTGAA GATCCACAAC CAGCTCATCT
127441 CGTCCGTCTC CAACATCACC TGCCCCAACT TTGATGCCAG CATTTGCATC CCGGTGAGTT
127501 GGCCACCTGG GGCCTGGCTG TGTGTACTCT GCCGGGAGTG GGGGTGCCTG GTGTTCTGGG
127561 GGGCTGGGGC CCCAGTGCTG CGACAGTGAC CTCGGGCCTG GTCTGAGCTG CCGCAGGAGG
127621 CTTTGCCTGG GGCTTTCTGC AGCAGCTACC CCCGCCCACG GCATCGTGGG AAGGTGCTCT
127681 CATCCCCAGG AATGTCCGGG GGTCCCGGGC TCATTCTCCT TTCCCTCTAG GGCTCCATCA
127741 CATTCATGCC CAATGGATGC TGCAAGACCT GTGAGTACAG GGCACAGCCT GGGGGGTAGG
127801 CAGGGTGGGG GCACAAGGGC TGGTGCCCTC AGCCCCGCCT GGGGTGGCTG GAGGCTGGAC
127861 AACGGCCTCT GGGTGGGCAG TGAGGGCTGG GGGCTGAGGC CGAGCCTGGG GAGGGGACGC
127921 AGCGAGGGAG AGCCTCCTCG AAGATGTGGA GGCCCTGCCC TAAGCCGCTG CCCGCTCTCC

127981 CCAGGCACCC CTCGCAATGA GACCAGGGTG CCCTGCTCCA CCGTCCCCGT CACCACGGAG
128041 GTTTCGTACG CCGGCTGCAC CAAGACCGTC CTCATGAATC ATTGCTCCGG GTCCTGCGGG
128101 ACATTTGTCA TGTGAGTCCC AGGCTGGGAG TGTGCCTGGA GGGGGTGGTG GAGACCCCAG
128161 GGAGGCGAGA GGCCAGCGCT GGCCCCGGAA GGTCACCCCT CACTCCGCCC TCCCCCCAGG
128221 TACTCGGCCA AGGCCCAGGC CCTGGACCAC AGCTGCTCCT GCTGCAAAGA GGAGAAAACC
128281 AGCCAGCGTG AGGTGGTCCT GAGCTGCCCC AATGGCGGCT CGCTGACACA CACCTACACC
128341 CACATCGAGA GCTGCCAGTG CCAGGACACC GTCTGCGGGC TCCCCACCGG CACCTCCCGC
128401 CGGGCCCGGC GCTCCCCTAG GCATCTGGGG AGCGGGTGAG CGGGGTGGGC ACAGCCCCCT
128461 TCACTGCCCT CGACAGCTTT ACCTCCCCCG GACCCTCTGA GCCTCCTAAG CTCGGCTTCC
128521 TCTCTTCAGA TATTTATTGT CTGAGTCTTT GTTCAGTCCT TGCTTTCCAA TAATAAACTC
128581 AGGGGGACAT GCTGTACTGT GTGGTTTAGG TTGGTGCTGC AGGGGTGCGG CTTGGCCACT
128641 GTGCATGGCG GAGGCCACCA GGCTCTGCGT GCAGGACACG GGGGCACCAC ACACACGCCA
128701 CGCGGTTGGC AAATCCCTTG TACCAAATGC AGAGGGGACG TGGGGGCTGC CTGCCCTCCG
128761 CTCCCCATTC TCACCCTGGG CAAAACCCCC ACGGGGCTGT CGAAATGTGG TCAGATTCCC
128821 TTGTAGGAAT CCCCCGGCCC TGAATCTGTG AAAAGAGCCC TGGGTTCCTT CACAGCCATC
128881 TCACAGGCTT TCCACGGTTG GTGGGGCCTT TGTGTAGCCC CGAGGGTCAG GTGGCCCTGG
128941 GGAGGGTGTG TGTGTGTGTG TGTGTGTGTG TGTGTGTGTG TGTGTAGCCC CGAGGGTCAG
129001 GTGGTACTGG GGAGAGTGTG TGCGTGTGTG TGTGTGTGTA GCCCCGAGGG TCAGGTGACC
129061 CTGGGGAGGT GATGTGTGTG TGTGTGTGTG TGTGTAGCCC CAAGGGTCAG GTGGCCCTGG
129121 GGAGGGTGTC TGTGTGTGTG TGTAGCCCCG AGGGTCAGGT GGCCCTGGGC AGAGGGTGTG
129181 TGTGTGTGTG TGTGTGAGTG CATAGCCCCG GGGGTCGGGT GGCCCTGGGG AGGTGGTGTG
129241 TGTGTGTGTG CGTGTGTGCG TGCATGCACG TGTGAGAATT TGCTTTGCAC ACGTGTCTGC
129301 ATGTGCCATG TGTATGTGAT GTGTGTGCAC GTGTGTGCAT GCATGTGCGT GTGAGCATTT
129361 GCTTGTGTGC ACACGTGTCT GCATGTGCCA TGTATGTGAT GTGTGTGTGC ACGTGTGTGT
129421 GTGCATGAGC GTGTGAGCAT TTGCTTGTGT GCACACGTGT CTGCATGTGC CGTGTGTATG
129481 TGATGTGTGT GCACGTGTGT GTGTGCATGA GCGTGCTAGC ATTTGCTTGT GTGCACATGT
129541 GTCTGCATGT GCCATGTGTA TGTGAGATGC GCCTTGTCCT GGGTCTCTAC ACTGGGAAAA
129601 TGGAGAAGGT CTTAGTCCTC AGGTCACTGT CAGCTCCTGC TGCCTAGTGA CAAAAGGTGG
129661 CCAGGACCTG GTGGCCCCGT CAGGGCTTTG GGGAGCTGCC AGTCAGAGCC GCCAATACCT
129721 TCAAACCTGC AGAAGTTCTG GTTGGCTCTG CGGACCCAGC TGGACAAGGG GCACTGGCTG
129781 TGGACCCTGG GCACAGTGCC CACTCATGTT GGAGAGCTGA GCCCCTGACT TCTGGGTGGA
129841 GCCCATGGAG GCTGCTTGGC CCAGAGCAGG TGCCTGGTGT CTCCTGTCAA AGGCAGCAGG
129901 TGTGTGTGGG GAGGCCGAGG CCTCTAAGCA CAGTGATCCA ACCTCGAGCA TCCTCTGAGG
129961 TCAAAGGGGA GGACATTTGT CTCCAGACTA CAAGGCTGTG CCACACCCCT TACATGTGAC
130021 GAAACCCTCA GGAGGGCAGA CACCTGGGCT GGGCTCCACA CTTCCAAAGC CAGGGCACAA
130081 CGGGTCCTCA GGGTGTGACA GGTCCTCAGG GCATGACGGA TCCTCAGGTG TGATGGGTCC
130141 CCTGGGCGTG ACGGGTCCTC AGGGTGCAAC AGGTCCCACC TCCTGCTGTC CTGCCTCCCA
130201 AATAGTTGTA CAGGCATGTG GCACCATGAT TGGCTAATTT TTAAGAGCTT TTTGCTTTTT
130261 GTTCTTATCT TTCAAAACAA TCCTTTGTTT TACTAAAGTA ACAGGCACGT GATTTTTGCA
130321 TAGTTTTGTT TGTTTTGCAG AGACAGGGTC TCACTATGTT GCCCAGGCTG GTCTCAAACT
130381 CCTTCCTGGG TTCAAGTGAT CCTCCCACCT CAGCCACCCA AAGTACCAGG ATTACAAGCA
130441 TGAGCCACTG ATCTTGGCTC CTGGCAGGGG GCTGAGGCCA CTGCCCTCCC CTAGCTGCCC
130501 TCCCCTAGCT GCCCTCTCCC CCATCTGACC CCAGGGAGGG CCGCGGTTGA GCAGCAACCC
130561 TGGGGTCTGA AGCCAGTTGG GACATCCCAG CTCATCAGCG CCACCCTGGG TCCTGGAACC
130621 CGCTCAGGCC TGGGGTCTTT CAACTGCCCC ACTTGGCCCA GGACAGCCCC CCAAAGGACC
130681 TGCTCATCCC CTCTGGTGGC CCCAGGAGCC CACACTGTCT AGGCCTAGGG ACCTCCTGGG
130741 CAGTCAGTTG GCCTGGGCCT GCCATGGAGC CCCTGAGGCC ACCCTAGAGC CTCTGGCATA
130801 GCCACGTGGG GATTCTGGGT GCTTCAGAAC TGGCCCTTCC ATGAAGCTGT CAGCATGGAC
130861 CCCAGAGCCA TCCTTGGGTC ATTTCGAGGG TGGCCTGTTT CTGGCCCACC CCCAAAACCT
130921 ATCACATCTC TGCCTGGCTG TGACCCTTGG GCCCAGCAGT CCCTGCACAG GCCAGGAAAT
130981 GGGCAGGGCG GGGTGGGGGC ACTGCGGCCA GAGACCTGGT AAGGAAGAGG TGGTCAGGCT
131041 CCCTCCAGTT TCCTCATCTG TCTCCCAGCT TGCAGCTGCG AAGAGGGGCT TCCCCTGGTG
131101 ATGCAGCGTG GACACGGGTA CGGCTAGGCC CCTGCCTGCT CTTCCCTGCT GTGCCCTTTA
131161 AAGCAGAGGC TACCCGGGAA GCTCCAGGAG AGCATGAGCC CTGACCCCAG TCCTTCCCCA
131221 GACAGCCCTG CCCCTTCCCA GAGCAGGCCC CACCCCTTCC CTAGAGGAGG CCCCGCCCCT
131281 TCCCCAGAGC AGGCCCCACC CCTTCCCTAG AGGAGGCCCC GCCCCTTCCC CAGAGCAGGC
131341 CCCACCCCTT CCCTAGAGGA GGCCCCGCCC CTTCCCCAGA GCAGGCCCCA CCCCTTCCCT
131401 AGAGGAGGCC CCACCCCTTC CCCAGAGCAG GCCCCACCCC TTCCCTAGAG GAGGCCCCGC
131461 CCCTTCCCCA GAGCAGGCCC CACCCCTTCC CTAGAGGAGG CCCCGCCCCT TCCCCAGAGC
131521 AGGCCCCACC CCTTTCCTAG GACAGGCCCC GCCCCTTCTG CCCACTCAGG ATCTTCCTTT
131581 TGGGTTCTCT GTGTCCAGAA CTCCAGTGCA GGTGTTGAGG GTGGGGAGGG AGCTGCCCCT

131641 TCAGGTGGAG GCAGGGTTGG TGCCAACGGA GGGGCAGGGA GACGCAGGGG CTCCCCCCAA
131701 CCCCGTCCAG TCACGGTGCA GCCCCCGACT TTATCCCCAG CGCCCTCCTC TTCCTCGCAT
131761 AACTCATGCC CCCAGCTGGG TCCTCCTGGG TCTCCCTAGG GGTGACTCGG GCCAGGGGCT
131821 ACCTGTTTCC CCGGGCTCAC CACAGTGGGC TAAGCCTACA GCAGAGGAGA TAGGGAGCCC
131881 GCCAGCCAGG TGGGCAGCCG GCCACCCCCT CGGAGTAGCT GCACGGGTTG GGGTCAAGTT
131941 CTCGCATTCT CTGGAAGAAG CTGGCTGTTC TGTTCCCACG GCGGCCTCCC TGTTTCTGGG
132001 AGCAGACGGA AGGCCCCAGC GCGCACTCCT CCCTTCCGCC CAGGTGAGAC TTGCTGTTGT
132061 TCCGTGGGCT GAAACAGGCC ATGCGCCTGG CTGTCGGGTG CTCCGGGCGG CACCAGCAAA
132121 TGACCACAAA CCGGGGGCCG AAAGCCACAG GAAAGCTGTC TCCCACAGTC CCGGAGGCCG
132181 GAGTCTGAGG TGCAGGCGTG GCGGTGCCAC AAGGCCGCTC AGGCTCCGGG GAAGATGCTT
132241 TTGGCCCTTC CAGCTGCAGG TGGCTCTGGG CGTTCCTTGG CTTGCGGCTG CATCGCCCAT
132301 GCCCTGCCTC CGTCTCCACG GGGCTTCTCC TGGGCGTTTC TCCCTTTGCG TCTCTTCTAA
132361 GGACATTGGT CTTTGGATTT AGGGCCCACC TGATAATCCA GGCTGATCTC GTGAGGTCCT
132421 CAATCTAATC ACATCTGCAA AGACCCTTTT TCTTTTTCTG TTTTTGGAGA CAGATTCTCG
132481 CTCTGTCACC CAGGCTAGAG TGCAGTGGTG TGATATCGGC TCACTGCAAC CTCCGCCTCC
132541 CGGGTTCAAG CGGTTCTCCT GTCTCAGCCT CCTGAGTAGC TGGGATTACA GGCGCCCACC
132601 ACCACACCCA GCTGATTTTG TATTTTTAGT AGAGATGAGA TTTCACCATG TTGGCCAGGC
132661 TGGTCTCGAA CTCCTGACCC CAGGTGATCT GCCCGCCTCG GCCTCCCAAA ATGCTGGGAT
132721 TATAGGGGTG AGCCACTGCA CCTGGCCCCG AGTCTTGGTG GTTCACGGCA GCAGGGTTCT
132781 GTCTGGTTCA TGCTCCGTTT GTGGCTGCGT GGCTGGTGCC CTCCACTACA TCCGCTCACT
132841 CCAGGGCCTA GGCTGTGGGA GCAGCTATTG CCTCCGTGGT CACGGTGTGG GGACGGGAGA
132901 CCGAGGGTCT TGTCCATGTC CCAGTCCCCT GAGAGGTTCT GGCTCTGCTC AGACACCGAG
132961 TCCCTGGGGC TGCTGTACCA CCACCAGGCA GGAGAGGGTG CCAGAGACGT GTGGAGGATT
133021 TAAAAACGGA AGGATCTTAG GTGCAGACAG GTGTCTGATG GGAGTCAACA GCGTGTCTCA
133081 ACTCGCTATT ATAAAAAGAG CAAGCCCAGA CTAGAAGGAA GCCGTCTTAA CCTGTTTAGG
133141 AACATCTGCC CAGAAACTAC AGCACACATC GAACCCCTCC GCTTGCTGGG ACTTGAGAAT
133201 CACTCCCACA GAAGCCAGCG GACATGGGAG ATGTTGGCGA TGGCTGTGCC TTCCCTCTAT
133261 GGGGGTGCTG GAGGCTTCAC CAAGTCCGTG AGACAAGAGC AAGAAGAAAT GAAGACCAGC
133321 ATTGGGAAAC AAAACTGTCC TGATTTGTTT CATTTTAATT TATTTTTTGT AGAGACAGGG
133381 TTTTGCTATG TTGCCCAGAC TGGCCTTGAA CTCCTGGGCT CAAGTGATCT GCCTGCCTTG
133441 ACCTCCCAAA GTGCTGGGAT TACAGGTGTA AGCCATTGTA CCTGGCCTTT ATTTCATATA
133501 ATGTGATTAT CAAGATGTAA AAGCCAACTG GCTGGGCACG GTGGCTCACG CCTGTATTCC
133561 CAGCATTTTG GGAGGCTGAG GCAGGCAGAT CACCTGTGGT TGGGAGTTCA AGACCAGCCA
133621 GGCCAACATG GTGAAACCCC ATCTCTACTA AAAATACTAA AATTACCCGG GAGGTGGTGG
133681 TGGTGGGCAC CTGTAATCCC AGGTACTCGG GAGGCTGAGG CAGGAGAATG GCTTGAACCT
133741 GGGAGGCGGA GGTTACAGTG AGCCGAGATC ACACCATTGC ATTCCAGCCT GGGTGACAGA
133801 GCAAGACTCT GTCTCAAAAA CAAACAAAAA CAAACAAACA AACAAAGAAA GAAACATGAA
133861 GATGTAAAAA CCAAGAGAAT TTTTGACTCT ATTATTTGAA CTAATGAGAG GGTTCAGTAA
133921 TGTTGGAGAC AAGGTTGATG AGGGAAAAAA CAATAGCGTA TGTTTACACC AGTAGAAACC
133981 AAGCTGAAAT ATAATAGAAA AATACTTCAT TAACAGCAGC CTCAAAACCT ACGAAGATAC
134041 CTATGTAGAA AACGTCAGAT GGAAGGTGTC CAGGCCACTA TAACATTGCA AAGGGCATAA
134101 AAGAAGACTT GACTGATGGA GACGTATGCC ACGTTCATGA ATGAAAAGTG TAAGTTTTGA
134161 AAAATGAGAT TAATTTTAAA AAGTGAAAAA CGAGGTCGAT TCTCCGCAAA CAGCCTTTCT
134221 CAATCCCAGC TGAGTTTTTC GTGGGATTTG GCCGGCTGGT CCAAAGGTCC ACGTGGAAGG
134281 CCAGAGAGCC AAGCAGAGCT AAGTGAGTTT CAGAGAAGAA TCATGTGGGA CGCCTGCCTC
134341 TAAGACAGCG TGGCATTGGT GCCGGGATAG GCAAGTGGAT GCCTGGAACA GCCCATGTGC
134401 TTACGATGAT GCTTTGAGGT GGGTGGGTGC TCCACATCTA CAAGGAAGGA GGGTTGTTCT
134461 CTGAGGGTGC TGGGCCGGGC TGTTCCCTGA GTGTGCTGGG CTGGGCTGTC CCCTGAGTGT
134521 GCTGGGCTGG GCTGTCCCCT GAGTGTGCTG GGCTGTCCCC TGAGTGTGCT GGGCCGGGGC
134581 TTCTCTACAT GGGGAAAATT AGGTGCCTGT GTCACGCTCT AAACAAAAAT AAAACACCAG
134641 GTAGATTAAA CAACTCAGAT GTGAAAAGCT AAACCTGAAA ACATTTAGAA GGAAAACAGG
134701 CATAATATTG ATGACCTCAG GGTAGGAAAG GGTTTCTTAA GTCACTAAAG TCACAAGCTA
134761 TCAAAGTAAA GAGAGACGCA TTTGACCACA TTACATTTTA AAAACTGTGT TTGAAAAAAG
134821 ACAACATAAC TAAGTAAAAA TACAAGCCAG GGAGTGGAAG GAGGTCATCT CTGTGCATCC
134881 GGCGCTGAGG CCGGAGTCCA GGAGATCTAG CGAACGTCAT GGTCAGACAA GCAGCCCAGG
134941 GTTGAGGCGG GGGTCACACA GGAGGAGACG CGGACAGGCG GGGGTCACAC AGGAGGAAAC
135001 ACGGACAACA CAGGGAGTAG CCAGCAGGGA GGCTCGGCCA CTGGGACCCC AGGAAGGGCG
135061 TGATAATCCT TGAGACCAGC TGAAGGTTCT CACACCTCCA GAGAGAACAT GCTGGGGCGT
135121 TTAGGGGAGA GCGGTTGGTC AGGGCAGCCA AGCTGAGGAC ACAGTCCTGC TTCTGGGCAC
135181 CCAGGGAGCT GCCCACCCGG GAGGATGGAG ACTGGAAATG GCCTGCATGC TGGTCCACCA
135241 GGAACGGGCT GCTCCTCTGG GCATATTCAT CCTCTGGATG GGGCCCAGCA CTAAACCGGA

135301 ACTGCCTGTC TTGACAAGGA GGCATCGCAA ACCCAGCATC GAGCAGAAGG AATGAATCGC
135361 GGGTCTCTAA TCCTGCACCC TGATGCTGTC CGTTTCTGTG AATGTGAAAC AGGACCCTAA
135421 TGCTGGACAC TTACAAAAGT AGTAAAATTA TAAAATATTT TGAGAGGCAC TGGATTCAGG
135481 GTCACGGTTG CCTCTGGAGA GGGTTGGAGA GCAGGGAGGG GCACCCACTT CTGCCCGTGA
135541 TGTTTATTCC TTGATGGAAA ACACCTGAGC TAGAGTGGCA GGTCTGATAT TAGTTACAGC
135601 TGACGGTGAG GAGGTAGTGG TCATGTCCAT TCCATTACTC TGTTCTTCAT AGGGTTTGAA
135661 ATCATCCATA ACAAAAAAGT CAGAGGGGCC GGGCACGGTG GCTCATGCCT GTAATCCCAG
135721 CACTTTGGGA GGCCGAAGCG GGTGGATCAC CTGAGGCCAG GGGTTCGAGA CCAGCCTGGC
135781 CAACATGGCA AAACCCCATC TCTACTAAAA ATATAAAAAT TAATTAGCTG GGCATGGTGG
135841 CACATGCCTG TAATCCCAGC TGCTCGGGAG GCTGAGGCAG AAGGATCACT TGAACCCAGG
135901 AGGCGGAGGC TGCAGTGAGC TGAGATTGCG CCACTGCTCT CCAGCTTTCA TGACAGACTT
135961 TGTCTCCAAA AAAAAAAAAA AGAAAAAAGA AAGATTTAGC ACTATTCATT CATTTGAGGA
136021 AAAAAGTGGC CGGCGCTGTG GCTCACGCCT GTGATCCCAG CACTTTGGGA GGCTGAGGTG
136081 GGTAGATCAT GAGGTCAGAC CATGAGGTGG GATCAGAAGA CCAATACCCC AGGGTTGGCT
136141 CCCTAAGACC AGCCTAAACT GGCAGCCCAG GGCTGAGGGC ACGGCCAGAG CTGGGGCCCT
136201 CGCCCACCCA CCACCCACCT GCATGGGGAG GGGCCTTGGG GTGACTGAGC GTGAGAGGGG
136261 CTGTGCGGAG GTGGCTGTGA TGGTCTCTGG TCCCGCCTCT GGGGCAGGTG GTGCTGACAG
136321 GGCAGGATTT GGGCAGCAGG AGTCAGGGGC TCCCAGGGAA GCCTGGAGAG GCTGAGCATG
136381 GGGTTGGACC TCAACAGGCA CTTTTGGGAA ATCTTGTTGG GGGCTGGGGC TGGTGGAGAA
136441 GAGTTGGGGT CCTCGTGGGA GGGCAAGGCA GGCCTGCTGG GGCTGGGTAG GGCTGGGGAG
136501 GGATGAGAAG GAAGAAGTGT TTCCTTTTGT TTTTTTAAAT TATACTTTAA GTTTTAGGGT
136561 ACATGTGCAC AATGTGCAGG TTTGTTACCT ATGTATAATG TGCCATGTTG GTGTGCTGCA
136621 CCCATTAACT CGTCATTTGT ATTAGGTATT TCTCCTAATG CTATCCCTCC CCCCTCCCCC
136681 ACCCCACAAC AGGCCCTGGT GTGTGATGTT CCCCTTCCTG TGTCCATGTG TTCTCATTGT
136741 TCAGTTCCCA CCTATGAGTG AGAACATGTG GTGTTTGGTT TTTTCGTCCT TGCGATAGTT
136801 TGCTGAGAAT GATGGTTTCC AGCTTCATCC ATGTCCCTAC AAAGGACATG AACTCATCAT
136861 TTTTTTATGG CTGCATAGTA TTCCATGGTG TATATGTGCC ACGTTTTCTT AATCCAGTCT
136921 ATCATTGTTG GACATTTGGG TTGGTTCCAA GAAGAAGTGT TTCTAGAAGG ATCTTCCGGA
136981 GGCTGAGAGA AGGAGGGAGT GGGGAGGCTG GTGGGAACTG GGAGGGCAAG GGAAGGTGAG
137041 GCGTGGGGGC CAGAGACCCT GGAAGGTCTC CCCAAAGTGG ACAGAGTATC TGGAGACCCA
137101 GAAGGTGGAG AATACGGTGG GGCCCGGTGG GCTTCAGAGG GCCTGGGAGC TGGGTAGTAG
137161 GGGCCCCCCT CGGGCACTCG AGAAAGACCC CTCCATGCTG AGCTCTAGCC ATTTATTCCA
137221 GTCCAGAGGT ACGGGTCTTA GAGCACCCGA GGCCACTCCT GTCCCCAGGT CACTGTGGCT
137281 CCTGACCAGC CCTCAGGAGC CGAAGGGCGG GACCCCTCCC CGTACCCCAT GTTGGGTGCT
137341 GACCAGCCAC TGTGTGCGTC ATTAGGTAGG GCTGAATTAA AACCCCATAA ATCTCATAAA
137401 TAAATAAGGT GGCTGGGCCA GCCCTGTGTG CAAACACTCA GCATCGGTGT GGCTGCACCC
137461 GTTGCTGGGG GAGCCCGGGA CCCCCCTCCC CTCCTCGCCC TTGTCCCTGG TCCCTTGCTC
137521 TCCTCCCTCC TCCGGAATTC TAGCTCTTTC TCTCCAAACC AGGCGGTGAC ACCTCTGCGC
137581 TCCCAGTGTC CTTTTGAGGC TGAAGGGGGC GGCGTCCCAC AGGGCAGGGG CCTGGCCTTG
137641 TCTGGGTCTG GGGGCTCAGG ACTCTGGGGG CTGTTGCATC TCTGCTTGGA CCCAGAGTGG
137701 GGTCTGTGCC ACCCTGGATG GCGGCTCACA GAGGGCAGGG CTGTGGGGGA CAGAACTTGG
137761 GCCGTGGAGA GGCCTCAAGA GGAGGTGAGC TTGGGGCATC TCCTGGGCCC ATTACAGAGC
137821 ATGAGGTTGT GTGCTGGGGG CGGCCGGGGC TGAGACTGCA CCGTGGGGCA GCCAGGGCTG
137881 AGTCCATCAC AGAGCATGAG GTTGGGCTGT GGGGGAGGCC GGGGGCGGCC CGGGCTGAGA
137941 CTATGCGGTG GAGTGGGCTG AGGCTGCACC GTGCTCAGGG CTGGTTTAAG CTCTGGGTCC
138001 CTGTCTTGGA TTCCGTCATC TTTGGAGGGG CCTCCTTTCC AGTTTGCAGG GGGCCCCATG
138061 GGTTCTGCTC TGCAGGGGCT GTGGGAATGG TAGGTGCCCG CCCTGAGCCT GGCTCGGCTG
138121 TGATTGAATT TGTTGTGTGG CAGCCGTGTG GGTTACATGA AGGGAGAGGC TGAGGACCCC
138181 AACTCGAGGA AATTGCGATC TACGGCATCT ATGCCAGATT TCAAGTCAGC GTGTGAAGAG
138241 GGTCCGGGGT GTCCGAGGGA CCAACTGCAA ACCCTCCCCA CAGGGACCCT TTGGTGCCCA
138301 GATGGGACCC TGGGCCTGTT TCCACCGGCC TGGGGCTGCA GCCTGGGCAT GGCCAGGGGC
138361 AGAGCATCAG GAAGCCCTGG GAGATAGAGG GCTTGCCCAG GATGGGGTGC AGTGGTGGAT
138421 CACAGCCCCC TGAAGCCTCC ACCTGCTGGG CTCAAGTCAC CCTCCCATCT CAGCCTCCCA
138481 AATCTCCAGG ATTACAGGCA TGAGCCACTG CACCTGGCCA TGCTGCATTT TTCAATAGAG
138541 CAGAAATGTT TTTCTCAGCA GCAAACCACA TTTCCAGTGC AACGGGCTCC TGCAATCTCG
138601 GGGCCACTGC CAGCCCCGAG GCACTGACCC TCTGCCCTGC GTGTGCTGAG GAGAACGTGT
138661 GTCCCTCGCC TGACCCTGCA GAGCCCACAT CCTGCAGGAC CCCAGGTCCA GAGGACCCAC
138721 GCTCTGCCAC TCCCACCCCT GTCCCGGCCC CTCAGAGCCC CCCCATCACT GTGGTGCTTG
138781 CTGCTCTTGG AAAAGTGAGG AGGGTGCAGA CGGCAGGGGC CATGTTCTCC TGGGAGGCCC
138841 CTCTTGGCTT CTGCTGGTCT GGCTGGAGGC AGGGGCTGTT TGGACCCGGA CCCCACCGCT
138901 GACACCGCAA TTTGACCCTG ACCCTTTCTT GACCCTTCTA TGGCTCCTGG CATTCTTGGA

138961 GGGGCCCAGG CAGTGCCCGG ATTCCTCACA GACCAGGCTG ACCAGAGCCT TCTGCTAGGC
139021 ACACCCGCAA ACCTTTAACC TCGCTGTAGA GCCCTGGACC CTCCTGGCCT CTCGCTGCCT
139081 CACCCTCACT CCTGCTCCTG CTCAGGCGTC TGGGGACAGA GCATGTCCTT GAGGGTGTGG
139141 AGGCCAGCAA GGGCTGCACT GCCCTCCCCA TGGGCTCTCG ACATGGGAGG GGCCCGGGAG
139201 ACTTGGAAGG GGGTGGCCTG CAGGGATGGG CAGCCCTGGG GCGGTGGGAG GGTGGCGTGT
139261 GGCTGGCCAC CTGCCAGGAG TGCTGATTGC GGCGGGTGGT AAACTGCGGG TGCCCCTTTG
139321 CGGTGCCCGG ATACCTGGCG GGGAGCCCTG GGGGCTGCCC ATGAACCAGT GCCCACTTTC
139381 CTGCCCCTGG CACGGGCTCG GCATGGGACC CAGTGCCCAG TGCCCACTGC CCATTGCACG
139441 CCCCCCTCCT GCCCCAACGG CCGGCAGCAC TAGTGGCCTG CTTGGCCTCA CCTCGCTGTG
139501 CAGACGGGGG GTGCCTCAGG GCTGCCCAAA AATCCTGAGT CTGTGGCTCA GTGGAATCTC
139561 CAAACCACAG AGGCCAAAGA GGGCGGGGCC CCAGGAGGAT GAGCCATGTT GTCAGGAGGG
139621 GCCAGTGCAC AGAGGATGGC AGCCCCCTGG TCCCTGTCAC GGAGTCCGCG CCCCTGCCTG
139681 TGCTGTCACT GCCTGCTTGA GTCCCGGAGA GCCATTCACA TCCGTGCCTT GCACCCGTGT
139741 CCTCCACACC CAGGCCGGGT CCTGGTGCAC CCCGGGGTCC CGGAGAGCTG TTCACATCCG
139801 TGCCTTGCAC CCGTGTCCTC CACTCCCAGG CCAGGTCCTC GTGTACCCCA GGGTTGGAGG
139861 GACAAGGAAG AAGGCGTTCA CACTGTGACC TCCCCTGGGG CTGCCAGCAC CGGAGGGTCT
139921 TAGGCCTGGC TTGAGGACGG CCCCATCCCC ACCCCGTCCT CCTGGGCCTT GCCAGCATCA
139981 CTGGGTGGTC GTGGTGCCCC ACTCCTGTGT GGGCCCAGAA GTGACCTACG AGTCCCACTC
140041 AGCGCGGCAC CCTGGCAGCC TGGCATGGAA GGTTGTGGGT CAGGAGTGTC CCCTGCTGCC
140101 CTGGGAGCCC CCACCCGCCC AGGCTGGTGG CGGCTGCTCA GGGGTGACCT GCAGGCAGGA
140161 CAAGAGCCGA GGCTGCAGGG CAGCCTGTGT GTTGTCTTTG AGAGTCGGTT AGAGCCGCCC
140221 CAGCTGCCCC AGCCTCCTCT ACCTCCCTCT TGGCCCAGGC AGCAGCCTCG GCTGCAGAGA
140281 CGTCTGGTCA ACCCGAGGAG GGCCTGGGAT GTGGCCTTGG TTGAGTCAAT GTTTCCCTCT
140341 CTCCCCCGAG GGGCTGGCCT CTCCTCCCTG GGGCGTGTTC CCATGCTCCA GTCCAGATGA
140401 GCCGTGTCTG TTTTCCCAGG GAGACGAGAG AATATCTTTT CCTTTTCTTT GAATAAATGT
140461 GCCCATTGCT GGCACCTGTG CCAAGCGGTT CCCAGGCCCG AGAACCCTGC CAGGCTGTGG
140521 TCCTGACCGC CCCAGGGAAC CTGGAAGTGC CTGCACCTGG AGCTGGGAGA GATCCTGGAT
140581 TAGCCCCCGA GGGCAGGCAC GCAATGCCGG GGTCAGCCAG GGGAAGCAGC TGTCCACAGC
140641 TCCACAGGCT GCATGCCAAC CACGGCTGCA TGGCTGGGAC CTTCTCACCC GCCAGGGCCT
140701 CTCCACCAAG CAGCGCCTGC CTGGCCACCC CTGGACAGCC CCTGGGTTGG TCAGCGGGCT
140761 CCAGGCAGGC CAGCAGGGTC TGCCCACACC ACCCAGTGAG CGCTCTTGGA GGGGATGGGG
140821 GGCCTGGCAC ACAGTCAGGC TCAATGAACA TTTGGAGAAA GAAGGAATGA AGGGTCCCCC
140881 CCCACAGGCA GCTGGATGGG GGTCCGCTGT GGCCCAGCAC CATGTGCCCA GCCCTCTCCC
140941 CCTGGCCACC GTGAGGCCAC CTCTTCTGTG GTGGATGGCG GCCTGGGCTG TGAGCCCTGC
141001 GTCAGCCGTG GACCCACCCT CCCTCTCTTT CCTTCCTCTC TCTCTATCTC TCTCCATCAT
141061 TTCTCTCCCT CCCTCTTTCC TTCTCTTGCT TTCTTTCTCT TTTCCCTTCT ATCTCTTTTT
141121 TCTTTTTCTC TCCCTTGTAC CCTCCCTCTT CCCTCCTTCC TCCTGATTCC CTCCTTCCTC
141181 CCTCTTCCCT CTCTCTCTCC CTCCTTCTTC CTTCCCCCTC CCCCTTCCTT CCTCCCTCCG
141241 TCTTTCCTTC CTTCCCTCCT TTTTCTCTCT CCCTCCCTCT GCCTCCTCTG TTTCTCTCAC
141301 TCCCTCTGCC TCCTCTCTCT CTCTCTCTTC CTCCTCTCTC TGTCTCTCTC TCCCTCTGCC
141361 TCTTGTCTCT GTCTCTTTCC CTCCTTCTGC CTCCTCTCTC TGTCTCTCTC CCTCCCTCTG
141421 CCTCCTCTCT CTGTCTCTCC TTCCATTAGT ACATCATATG GGATGCAATG CTGGCTGAAG
141481 GCATGTGGGA CAATTTTTTA GACTGTCCTT TTTCTCAATC CCTCCATAAC ATTCAGGCCA
141541 CAGAGGCTTT GGTCCTGTCC TTGCTGTGTC CAGCATCACC TGCAGTAAAA TCCAGCCTCA
141601 GTCCCCCTTT AGGACATGCG GGGACAGAGC TCCGTGGAAC CCCAGCATGA CATGCTGTTC
141661 ACCTCTTTAA CGCTGCACCT TTTCATACTG TTAAAATGGC AATCAGATTT CCAATGTATG
141721 AACTTTGAGG AATATATTCA CACCATAACA TGCCTCTCTG TCCTTTGGGC CTTCTCAGAC
141781 CTGTTCTGTC CACCTAGTGC AGGTGCTGTC AGCTTCAGCC TCCTCCTCAG AGACCAGACA
141841 TGTCCTACAA TCTGGGGTCC CTTGTCTGGC CAGAGGGAGG GACGTCAGGA TGGGACAGGT
141901 ACTCCAAGAG TTCTGACTTA AACAGCCCAG GAGGTGGTTC CTTGGGGTGT GGAAAGAGAG
141961 CAGGGGTGGT TCAGGAGATA GCCCGTGGCT ATTGTTCATG TGGGGTCTGA ATTCAGGCTG
142021 CTCACATCTG GTTCTGCCCA AGACTTAGCT TCTCAACATC TGCACCAGGA ATAGAGGACC
142081 CACCTGCCCC AGGGAGAGAT GACTGACCTG CCCCAGGGCC AGAGGATGCA CCTGCCTGAG
142141 GACAGAGGGG TGTGAGTCCT GCCCTCTGCT CATAGGCAAC CCTGGAGCAT GGGCTCAAGC
142201 CTTCTGAGAC TTGGTGGGGG CATGGGATTT ACTCCCAGGC AGGGCTCACC TGGACCAGGG
142261 CCCTCCACTG CCCCTGGTGC TGAACTCAAC CCCACCCCTG AATACTGCCG TGGGGTTGTC
142321 TATGAACATC TCCCCCAGCT CTCAGTGAGC TCCCTGAGGG CAAGACCTGT TCCAGCCCCA
142381 TGAAGGCCCC ATACATGTTG TGGAATGAAT AAATATGAGT GAATGAGTAA GTGAATATGA
142441 ATATCAATAA TACAGTTGAA CATGTATGAA TGAATGAAGA GAAATATGTG AATGTAGATG
142501 GATGGGAATG AATGGATGGG AATGAGTGGA TGAGTGAGTG AACAGGAATG AGTGGATGGG
142561 AATGAGTGGA TGGGACTGAA TGGATGGGGA TAAGTGAATA AGGATGAGGG GATGTGAATG

142621 TAGATGGATG GGAATGAGCA CATGAATGAG TGAACAGGAA TGCATGGATG GGAATGAGTG
142681 GATGGGAATG TAGATGGATG GGAATGTAGA TGGATGGGAA CGAGTGGATG GGAATGAGTT
142741 GATGGGAATG AGTGGATGGG AACGAGTGGA TGGAAACGAG TGAATGGGAA TGTAGATGGA
142801 TGGGAATGAG TGGATGGGAA CGAGTGAATG GGAATGTAGA TGGATGGGAA CGAGTGGATG
142861 GGAACAAGTG AATGGGAATG TAGATGGATG GGAATGAGTG AATGGGAATG AGTGGATGGA
142921 AATGTAGATG GATGGGAATG AGTGGATGGG AATATAGATG GATAGGAATG AGTGGATGGG
142981 AATGTAGATG GATGGGAATG AGTGGATGGG AATATAGATG GATGGGAATG AGCGGATGGG
143041 AATGTAGATG GATAGGAATG TAGACGGATG GGAATGAGTG GATGGGAATG AGTGGATGGG
143101 AATGTAGATG GTTGGGAATG AGTGGATGGG AATGTAGATG GATGGGAATG AGTGGATGGG
143161 AATGAGTGAT GGGAATGTAG ATGGTTGGAA TGGATGGGAA TGATGGATGG TGTGATGGAT
143221 GGGAATGTAT GGAATGAGTG GATGGGAATG AGTGGATGGG AATGAGTGGG TGGGAATGTA
143281 GATGGATGGG AATGAGTGGG TGGAAATGTA GATGGATGGG AATGAGTGGA TGGAATGAGT
143341 GGATGGTGAT GGATGGGAAT GAGTGGATGG GAATGCGGAT GGGAATGTAG GTGGATGGGA
143401 ATGAGTGGAT GGGAATGTGT GGATGGGAAT GTAGATGGAT GGGATGGATG TGGATGGGAA
143461 TGTAGATGGG AATGAGTGGA TGGGAATGAG TGGATGGATG GATGAATAGA TGGGAATGAG
143521 TGGATGGGAA TGAGTGGATG GATGGATGAA ATGAATAGTG GGAATGAGTG GATGGGAATG
143581 TAGATGATTG GGAATGAATA GATGGGAATG AGTGATGGGA ATGTAGATGG ATGGAATGAG
143641 TGATGGGGAT GGGGATGTAG ATGATGGATG GGAATGAGCG GATGGGAATG TAGATGGATG
143701 GGAATGAGAT GGATGGGAAT GAGTGGATGG GAATGAGTGG ATGGGAATGA GCGGATGCAT
143761 GAATGAATGA AGATGACTCA ATGAATGAAC AAACAAACAA CAGTCTCCCA CAGTGGCCTG
143821 TGGTGTGAGC CCTGCTGCCA GCCATGGAAA CTGAGGCAGA GAGGCTCCTG GCTCAGCCCT
143881 GCCCAGCCTG TCCAGCCCCT GTGATGTTGG ACCCTCCGGT GCAGACCCAC CAGTCATCTG
143941 CAGTGACGGG TGGCAGTGGA CGTGGGCTGA GTCGCGGCGG ACTGAGTCCT GGTGGCCACT
144001 GCTTGATTTC ATTGCTGGGC AGTGCTCCCG TGCAGGGTGT ATTTGTCCAC TCCCCCTTGG
144061 GGTGCAGGGG CTGTGCCTTC GGTGCCTGTG GCTTCAGTGC CTGTGCCCGG GAGAGGGCTC
144121 ATTCCCCATG TTGGCTCCTG TGGGCCCCTG AGCCCTAGTA TTCAGTTCAT TGGCTGCTGT
144181 GTGTCTACCG GGGCCGCCAT AATGAATGCC CACAACAGGT GGCTTGAAAA CATGGGAAAT
144241 GTATCCTCTC ACCGTTCAGG AGGGCAGAAG TCCCAGTTCT GGGAGGCAGA AGTGTTGATT
144301 CCCACTGGAG GCTTGGAGTA CCTCTGACAC ACATACCCGG CCTTTCCCTA TGGGGACACA
144361 CATGGATGGT GGACGACACC ACCCAGATCG TCCAAGAAGA TTTCATCTGA GATCCACATT
144421 ATACCTTCTG CCAGCAAGGC AGCCCTCCCA GGTCCCAGGG TTCAGGGTTT GGGCTTATCT
144481 TTTGGGGGCC ACCCTAATCC TCCTATAGTC CCAGAGGCCT GATGGGACTC ACAGCTCAGC
144541 CTAAATGGTC CCCTGGGACA GATGGACGGG CAGTCCAGCA GCCAGGCAGG ATGGGAGGGA
144601 GGTCCACCCG TGCTGGAGAG CTCGGACCCC TGGGCCAGCC AGGGCCTGTG CGAGGCCCAT
144661 GCACAGACAG GAGCTGCTTG GGAAGTCGTG GTCCTAACCC CTGATGCTGA CACGCAACGT
144721 CTCCAGATGC CCTGGTGGTG GGGAGACATC AGTCCCTCCC AGCTGGTCAG CCTGCGGACT
144781 ACTGAGCTGG ACACCGTGAG GAGCCATCTG ACTCGACGGA GGAAGCCCTC ATGGGGGCGT
144841 GGGATGCTCT GGGGCCTCTG CACTGCCCCC ACCCCCCCGC ACCCTGAAAA CGACGTCTGT
144901 GTGTGGTGCA GCTAGCGTGG ACCATGTCCC ACGGAGGGCC CACCCCTACC AGGTCCGTCT
144961 TCTGGCATGG CCATGGCTGG GCCTTCCGCC TCACCCGCTC TGGGAGGGAG CCCTGGCCTG
145021 CGCTGGAACA CTCGGCCTCC TCCGTGAGGG GGTTTGTGCT CGGACAACAT GAAGGCAGAC
145081 GGATGCCTAG CACAGCTGGC TTGGTAAACA GAGAATGTGC CGTTTACTCC CACATCCGCA
145141 GAGTAAATCC CCACGGGCAG TGCGCCGGGT GCTGGGGACC GGCAGTCTCC ACTTTGGGGA
145201 GAGGCAGAGC AACAACGCCT GACCCCAGGA GTTTGGAGCT GCACCCTGGT CTGTGTGCGT
145261 AAGGCTCCTT GGCTGCACCC CACGCTGGGT GAGGGCACTG GGCTGGAGAT GGGGCAGGTG
145321 GGCGACTGTC CCGGTGGCCC TCCAGCGCGG TCCCCAAACC GGGCCTTTCC CACCACCCGG
145381 GGAGGACGCT GCTTATGGCC AGGCATCTTG GGCAGCCCCA GTGCACAAAG TCAGGGCTCC
145441 TGGTGGCCCG GGCAGGCTCT GTGCATGGTG GGACCACCTT AGCCTTGCCT CTTGCAAAGC
145501 AGCGCCCAGC CCCCACCCTG CCCTCTTGTT GGCTTTTCTG GGAGGCCTGT GGGGGGCCTT
145561 TCCAGTGCCC TGGTGCCGCC CCTGAGCTCT GGGTCACTGC CCAGAGGCGT CTGAAGCAGA
145621 CACCTGCCCC TGCCTCCCTG AGCCCACGCT ACCCGCTCTG TATCCTATCT AGAACTTTCA
145681 CGAGCCGACA TTGTCCTGTG TGCCAGCTCA CTGTCCCCAC GGAACAGCCA TGCCACGAGG
145741 GTGGGGTCCC TTTTGTTTGG GGGGCAGAGC TCAAGCTTTT GGATCTGCTT CAAGCCCAGA
145801 GCCACCCGTC AGCTGAAATA CTGAAAGGAG GAAGGGGTGA GAGGAGGAAG GACTACGGGG
145861 TGGACAGGGC TCTCAGCAGG CACCCCTGCC CCACTTCACA GCAGCCCTGC GGCCGGGGAC
145921 GGACTTTGGC AGGGCTGGTG GCAGCTGGGT CAATCTGGGC TTTTGGGAGG CTCTGGTTCT
145981 GTGGACCCTC TAGGGCTTGT CCCCCCGCCT GTGACCTGAG GTCTCCTGAG CTCTGCATCT
146041 CAGTCTGCTC TCGAGTTAAA TGGGGACAGT ATCACATGCT ACAGAAGGCC TGTGGCAAAA
146101 CGCTGTCTGT AGAGACCCCA GCACAGCTGC AGCTCAGAAA CAGCCACCGT TGTGAAGAAG
146161 ATGCGTAAGA GTCAGTTGAT CATGACTACT AGCCTTTCCT TGAAGCCTGT GTGTACAGGT
146221 AAAACTGCTG TGTAGCACAC ACACACACAC ACACACACAC ACACACACAC ACACACCCAC

146281 GAAAAGCACG CAGCCAACTG AGCCCAGGTC TGACGCTGTC TGTCCTGACT CTAGGACACA
146341 CCCGGGTTCC TGACATTCAC AGTGAACCTG TGAGCTCTGG AGCCAACGAG AGACGTTATT
146401 GGCCAACGCA CTGCCCTGGG GCCGGGTCGC TGGGGGGACC CAGGTAGGAG GCAGGGCTGG
146461 GGGAGCAGAA AGGCAGGGAT GGGGATGGAC GTGGCTGATG CTGTGACCTC AGCCAGGACA
146521 GTCCCCGAGT GGAGGAGGAG GCTGGGCCCC CCTAAGGCCG CTCTTCCCCA GCCTCCTGCC
146581 CCCCGGCCCA GGTCTTATCA CCCTCACCCA GCCCCGGCCT CCCCTCTCGA CCCCCAATCT
146641 GTTCCCACCT CCGTTGGAGG AGGCTCCTGA GGTGGGTGCC CCTCTAGCCT GCCTGCCCCT
146701 GTCCAGGCCC TGAGGGCTCC AGAGGGGGGC CGAGGCCTGG GGGTCCCGAC CTCCATGTGC
146761 CTCCCGTGTC CCCCGCTGCA GCCCCACACG CACCCCACTG TCCAGCCACA CAGGCCGTCT
146821 GAGGCTGGGG GCCCAGAGGC AGACTGCCCC AGATATGTGG GCAGGGGTCA GCGGGGCTCT
146881 CAGGACACCA GCTGGGGGTG AGGGGAGCAT GGAGGGAGCG TGGGACAGGC TGAGCTGCCG
146941 AGGGAGCCAC CCTGGAGCTC TGGAGCCGGG AGGCCTTGGG AGACATCCCA GGCTGGGTTC
147001 TGTCCCCCGA TGTGGCAGGC GTTGCACGCA GGCTCGCTGT GGCCTTGGGC GAGGTGGCTT
147061 TCTGGGGAGG GCAGTTCCCA GGGAAGGAGG GCCGAGCCCT CGGCAGCCAA CACTCGGGTG
147121 GCTTGGGACG AAGGCCTCGG TCCTGGTGGG GCCTGGGCGA CGCCCCCAGT GCCCACCACG
147181 CAGGCCTATG GTTGGTCACG GAGGTGGCCT TCTCCTCCTA CCCACTGTCT CTACCTCAGC
147241 TTCCAGCAGG TGCCCAGTGC TCACGCCGGC CTCGAGCCTG GACTGGGGGC TGCACCTGCC
147301 CCAACACTCT CAATGTGTGG CTGGGGAGTC TGAGGCCCAG AGGCACCCAG CCCATCCCCC
147361 CACAGAGCAG GCCTCAGACC TGCTGCCCTG CTGTCTGGCC GAGAGCACCA CCCGGGGTGG
147421 AGCGGCCGGT GAGCTGAGCC CGGGTCATAA GCCAGGCAGG GGAGCAGGCT TCCTGGGATC
147481 CGCCGACCCC GTCCAGCTGC TGCAGGGCCC CATGCCTGCC ACAGGATGCC CCTGAGCCTG
147541 GGGCATGGCT GTTGCCCTGC AGGGGAGGCC ATCTACTGGT GCAGCTGTCC TGCAGCTGGA
147601 CATGGCTTTG AGATGACCTC TGCAGGGTGG GGTGCAGGGA AAGGCCATCG AAACCTGAGA
147661 CCCAACACAG GGGAAGGTGG GGAAGGCTGC GGGGCCCAGC TGCACAGCGG GCTGGGACGC
147721 AGAGCCAGCC TCCCACCCTG GGCCTGTCCC CTGATCTCCG CCCAGTGTGG ACTCCTTTTG
147781 GGCACAGCTG AGACCAACCC AGCAGATAAG CGGATTAGCA TGGTGCATGC CCCCAGGGTG
147841 CACAACTGGC TTTATCAGGG CCCGGATGTT GTCTCAGGTG GAGCTGGGCA GGCTTAGGTG
147901 AGGTGGGTGT TCCCAGGTCA GATACGCTCT GCCCAGCTCT CACCCCTCAG CCGGCGCAGG
147961 GCCAGGGGCC TGGATCAGGG GTTCGCTCCA GCTGCCTGCT ATCTGGCTGC CCGTGGCGGG
148021 CTCGGGGCAA GGGTGTGCAG GAGAGCCGGG TCTTGGTGGC TTTGTGCTCC TACCCTGGGT
148081 TTCAAAGGCG CTGCCTCCCT TGTTCCTTGT TCATTCACAA AATCCGTGAG CGCCCCAGCA
148141 TGTGGGACAC AGATGAAGGG GCCGGGGGAG GGTTGCCACT GGGCCAGGGC ACCGGGAGTG
148201 TCCCCACGTG CCATTCGGCC GAGTCCTTGG CAGGGGGAAG CCCTGCTCAG AAACGGAGAA
148261 ACTGAGTCTT GGGACATTTG CTGTCTCGCT CACTGTCAGA GCCCCCTCCA TCCCAAGACA
148321 GGCCTGGGCA CCTGACAACC GTGCAGGACC GTGACATAAA ATATGCCAGG AAATCGGGTG
148381 GAGGAAACTC AGACGTGAGG GAATCAGGCT GGCAGAGCCG GGTGGGGAGA GGGTGCTGGC
148441 ACAGGCAGGG ACGCTCTAGG TGCTGGTTTT AGGGGCAGGG CTGCCTGGGA AGCCACCTGA
148501 GATGAGAGGG AGAGAGGCCA GGGGGACTTG AAGAAGCCCA GGGTGCTGGG AGAACCTGGG
148561 GGCCAAGCAG AGGAGGTGCC TGGGGGCCAG GCCAGGTGGG CACAGACCCA CAGCTACCCG
148621 GGCCCTCCTC ATCTGCCCCA CCCCAGAGCC CACCTGCATC CGCCACAGCT GGCCTGGCTT
148681 TCTGCCCGAC ACCTGCCAGG TCAGGGGGCC CCTGCTGGAA GCCACACCCC ACTCTGCCCA
148741 CCTCCCACCT CCTCACTCCC CCTGCCTGAG CTCCTGCCAG CCCTCACGGC GCCCTCCTAA
148801 CCTCCACTCA TCAGAGCCCT GCCCTGTGCC CCAGACTCGG CTGGAGGCAC CCCCAGGAAG
148861 CCCCTGTGGG AAGCAAGTCC TGGTGTCGGT CCTCACCCCA CCCCTCAGGG AGCCACAGGC
148921 CCAGCTCCTG GGTGTGTCCC TCTGGGTGGG GGCCTGGGGT CACAGGCTGG CCTGGAGCCA
148981 TGGAGACAGG GGAGCTGCCT GGCTGTGGGG GAAAGGCAAA CACCCTTTTG TCTGTGGCCG
149041 CCTCTTCTGC TCGGGGAACT CTGGGGACAA AGCCGGTGGG CCAAGGAGCC CCCGCTGTCT
149101 AGACAGCCCT GCCCCACGAG GCAGGCAGGG GAGGGGGCGC CCTGAGCAAA CAAGCAGGTA
149161 CCAGGCGTCC TGAGACTGCG GGGCCAGGCC AGGGTGGGCG AGCGACGAGG CCCGCGGGAG
149221 TGGGAGGGGG CGCGGGATGC TGTCCTTAGG GACCCTGGGC CCCAGCCGGG GTTTCTCCCT
149281 GGTCTCTCTC CTTCCTTTCT GCTGGCCACC ACCTCCAGGA AGCCAGCTGG ACCTGACCCT
149341 GCCTCCTGAG GGCTCTAGGA ACCCACCTCC CAGGTCCAGC CATGCACCAG CCTGTCCTCG
149401 TGACCCTCAC TGGCCTCAGG GATCCCCCAG TAAGGGGGCA GCGGTGGGGA CTCACCTGTG
149461 GGGCTTGAGT GAGCCATGCC CAAGACAAGA GGCAGAGCCA GGAGGTGCAG GTGACGCTAT
149521 GGTCCCCAGC TGCGTGGCGG CTCTCTCCAG GTGTGTGGGC CTCCTGGTCC CTTCGTGGTG
149581 TCTGGTGTCA CCATGCACAG CTCAGACCCC TGCTGCAGTC CCCAGGGATC CACAGCCGCA
149641 CCTGCCCTTT CCTCTTGCTT CTCACTGCGC CCCAGCCCCC TGGCGGCTGC TCACCAGCCT
149701 TCATGTCCTC ACTGACGCCT GCCCTGCACC GGGACCACCC TGTGTTCCCC AGCTGTGCCC
149761 AGACCTGATG GAAATTCTCA GCAGCCCAGG CCAGACCCAC CTTCCCGCTC TCCTGCCAGC
149821 TCTGAACGTT GGGCCCCTGG CACTGTTATT CACATCTTAC TCCATCAGTT CCCAGCCCAG
149881 CCCCAGCCCC TGCCCCTTCA GATGCAAACC CTGTGAGGGC CAGAGCCTCC CTGGTGTCCT

149941 CAC

Figure S3. DNA sequence of *HinfI MUC2* PTS-TR2 fragment.

```
   1 GATTGGAGAC GTCTGTGGAC CAGGCTGGGC AGCTAACATC TCTTGCAGAG CCACCATGTA
  61 TCCTGATGTT CCCATTGGAC AGCTTGGACA AACAGTGGTG TGTGATGTCT CTGTGGGGCT
 121 GATATGCAAA AATGAAGACC AAAAGCCAGG TGGGGTCATC CCTATGGCCT TCTGCCTCAA
 181 CTACGAGATC AACGTTCAGT GCTGTGAGTG TGTCACCCAA CCCACCACCA TGACAACCAC
 241 CACCACAGAG AACCCAACTC CGACACCAAT CACCACCACC ACTACGGTGA CCCCAACCCC
 301 AACACCCACC AGCACACAGA GTACAACACC AACACCCATC ACCACCACCA ATACGGTAAC
 361 CCCAACCCCA ACCCCCACTG GCACACAGAC CCCAACCCCG ACACCCATCA CCACCACCAC
 421 CACTATGGTG ACCCCAACCC CAACAATCAC CAGCACACAG ACCCCAACCC CGACACCCAT
 481 CACCACCACT ACGGTGACCC CAACCCCAAC ACCCACCAGC ACACAGAGAA CAACACCGAC
 541 ATCCATCACC ACCACCACCA CGGTGACCCC AACCCCAACA CCCACCGGCA CACAGACCCC
 601 AACCACGACA CCCATCACCA CCACCACCAC GGTGACCCCA ACCCCAACAC CCACCGGCAC
 661 ACAGACCCCA ACAACGACAC CCATCAGCAC CACCACCACG GTGACCCCAA CCCCAACACC
 721 CACTGGAACA CAGACCCTAA CCCCAACACC CATCACCACC ACCACTACGG TGACCCCAAC
 781 CCCTACACCC ACCGGCACAC AGACCCCAAC ATCGACACCC ATCACCACCA CCACTACGGT
 841 GACCCCAACA CCAACACCCA CTGGCACACA GACCCCAACC CTGACACCCA TCACCACCAC
 901 CACTACGGTG ACCCCAACCC CAACACCCAC CGGCACACAG ACCCCAACCA CGACACCCAT
 961 CACCACCACC ACTACGGTGA CCCCAACCCC AACACCCACC GGCACAAAGA GTACAACCCC
1021 GACATCCATC ACCACCACCA CTATGGTGAC CCCAACCCCA CCACCCACTG GCACACAGAC
1081 CCCAACCACG ACACCCATCA CCACCACCAC TACGGTGACC CCAACCCCAA CACCCACCGG
1141 CACACAGACC CCAACCCCGA CACCCATCAC CACCACCACC ACGGTGACCC CAACCCCAAC
1201 ACCCACCGGC ACACAGACCC CAACATCGAC ACCCATCACC ACCAACACTA CGGTGACCCC
1261 AACCCCAACA CCAACCGGCA CACCGAGTAC AACCCTGACA CCCATCACCA CCACCACTAC
1321 GGTGACCCCA ACCCCAACAC CCACCGGCAC ACAGACCCCA ACATCGACAC CCATCAGCAC
1381 CACCACTATG GTGACCCCAA CCCCAACACC CACCGGCACA CAGACCCCAA CCCCTACACC
1441 CATCTCCACC ACCACTACGG TGACCCCAAC CCCAACACCC ACCGGCACAC AGACCCCAAC
1501 CCCGACACCC ATCACCACCA CCACCACGGT GACCCCAACC CCAACACCCA CCGGCACACA
1561 GACCCCAACA TCGACACCCA TCACCACCAC CACTACGGTG ACCCCAACCC CAACACCCAC
1621 CGGCACACAG ACCCCAACCA CGACACCCAT CACCACCAAC ACCACGGTGA CCCCAACCCC
1681 GACACCCACC GGCACACAGA CCCCAACCAC GGTACTCATC ACCACCACCA CTACGATGAC
1741 ACCAACCCCA ACACCCACCA GCACAAAGAG TACAACCGTG ACACCCATCA CCACCACCAC
1801 TACTGTGACC CCAACCCCAA CACCCACCGG CACACAGAGT ACAACCCTGA CACCCATCAC
1861 CACCACCACT ACGGTGACCC CAACCCCAAC ACCCACCGGC ATACAGACCC CAACAACGAC
1921 ACCCATCAGC ACCACCACCA CCGTGACCCC AACCCCAACA CCCACCGGCA CACAGACCCC
1981 AACATCGACA CCCATCACCA CCACCACTAC GGTGACCCCA ACCCCTACAC CCACTGGCAC
2041 ACAGACCCCA ACATCGACAC CCATCAGCAC CACCACTACG GTGACCCCAA CAGCAACACC
2101 CACCGGCACA CAGACCCCAA CCCTGACACC CATCACCACC ACCACTACGG TGACCCCAAC
2161 CCCAACACCC ACCGGCACAA AGAGTACAAC CCCGACATCC ATCACCACCA CCACTACGGT
2221 GACCCCAACC CCAACACCCA CTGGCACACA GACCCCAACC ACGACACCCA TCACCACCAC
2281 CACCACGGTG ACCCCAACCC CAACACCCAC CGGCACACAG ACCCCAACCC CGACACCCAT
2341 CACCACCACC ACCACGGTGA CCCCAACCCC AACACCCACC AGCACACAGA CCCCAACATC
2401 GACACCCATC ACCACCACCA CTACGGTGAC CCCAACCCCA ACACCCACTG GCACACAGAC
2461 CCCAACCACG ACACCCATTA CCACGACCAC CACGGTGACC CCAACCCCAA CACCCACCGG
2521 CACACAGGCC CCAACCCCAA CAGCCATCAC CACCACCACT ACGGGGACCC CAACCCCAAC
2581 ACCCACCGGC ACACAGACCC CAACCACGAC ACCCATCACC ACCACCACTA CGGTGACACC
2641 AACCCCAACA CCCACCGGCA CACAGTCCCC AACCCCAACA GCCATCACCA CCACCACTAC
2701 GGTGACCCCA ACCCCAACAC CCACCGGCAC ACAGACCCCA ACCACGACAC CCATCACCAC
2761 CACCACCACG GTGACCCCAA CCCCGACACC CACCGGCACA CAGAGTACAA CCCTGACACC
2821 CATCACCACC ACCACCACGG TGACACCAAC CCCAACACCC ACTGGCACAC AGACCCCAAC
2881 ATCGACACCC ATCACCACCA CCATTACGGT GACCCCAACC CCAACACCCA CCGGCACACA
2941 GACCCCAACC CCGACACCCA TCTCCACCAC CACTACGGTG ACCCCAACCC CAACACCCAC
3001 CGGCACACAG ACCCCAACAT CGACACCCAT CACCACCACC ACCACGGTGA CCCCAACCCC
3061 AACACCCACC GGCACACAGA CCCCAACAAC GACACCCATC AGCACCACCA CCACGGTGAC
3121 CCCAACCCCA ACACCCACCG GCACACAGAC CCCAACATCG ACACCCATCA CCACCACCAC
3181 CACGGTGACC CCAACCCCAA CACCCACCGG CACACAGACC CCAACCACGA CACCCATCAG
3241 CACCACCACC ACGGTGACCC CAACCCCAAC ACCCACCGGC ACACAGACCC CAACATCGAC
3301 ACCCATCACC ACCACCACCA CGGTGACCCC AACCCCAACA CCCACCGGCA CACAGACCCC
3361 AACCCCGACA CCCATCACCA CCACCACCAC GGTGACCCCA ACCCCAACAC CCACCGGCAC
3421 ACAGACCCCA ACATCGACAC CCATCACCAC CACCACCACG GTGACCCCAA CCCCAACACC
3481 CACCGGCACA CAGACCCCAA CCCCGACACC CATCACCACC ACCACCACGG TGACCCCAAC
```

3541 CCCAACACCC ACCGGCACAC AGACCCCAAC CCCGACACCC ATCACCACCA CCACCACGGT
3601 GACCCCAACC CCAACACCCA CCGGCACACA GACCCCAACA TCGACACCCA TCACCACCAC
3661 CACTACGGTG ACCCCAACCC CAACACCCAC CGGCACACAG ACCCCAACCA CGACACCCAT
3721 CACCACCACC ACCACGGTGA CCCCAACCCC AACACCCACT GGCACACAGA GTACAACCCT
3781 GACACCCATC ACCACCACCA CCACGGTGAC ACCAACCCCA ACACCCACCG GCACACAGAC
3841 CCCAACATCG ACACCCATCA CCACCATCAC TACGGTGACC CCAACCCCAA CACCCACCGG
3901 CACACAGACC CCAACCCCGA CACCCATCTC CACCACCACT ACAGTGACCC CAACCCCAAC
3961 ACCCACCGGC ACACAGACCC CAACCATGAC ACCCATCACC ACCACCACCA CGGTGACCCC
4021 AACCCCAACA CCCACCGGCA CACAGACCCC AACAACGACA CCCATCAGCA CCACCACCAC
4081 GGTGACCCCA ACCCCAACAC CCACCGGCAC ACAGACCCCA ACATCGACAC CCATCACCAC
4141 CACCACTACG GTGACCCCAA CCCCAACACC CACCGGCACA CAGACCCCAA CCACGACACC
4201 CATCACCACC ACCACCACGG TGACCCCAAC CCCAACACCC ACCGGCACAC AGAGTACAAC
4261 CCTGACACCC ATCACCACCA CCACCACGGT GACACCAACC CCAACACCCA CCGGCACACA
4321 GACCCCAACC CCGACACCCA TCTCCACCAC CACTACGGTG ACCCCAACCC CAACACCCAC
4381 CGGCACACAG ACCCCAACCA TGACACCCAT CACCACCACC ACCACGGTGA CCCCAACCCC
4441 AACACCCACC GGCACACAGA CCCCAACAAC GACACCCATC AGCACCACCA CCACGGTGAC
4501 CCCAACCCCA ACACCCACCG GCACACAGAC CCCAAGATCG ACACCCATCA CCACCACCAC
4561 TAAGGTGACC CCAACCCCAA CACCCACCGG CACACAGACC CCAACCCCGA CACCCATCAC
4621 CACCACCACC ACGGTGACCC CAACCCCAAC ACCCACTGGC ACACAGGCCC CAACCCCAGC
4681 AGCCATCACC ACCACCAGTA CGGTGACCCC AACCCCAACA CCCACCGGCA CACAGACCCC
4741 AACCACGACA CCCATCACCA CCACCACCAC GGTGACCCCA ACCCCAACAC CCACCGGCAC
4801 ACAGAGTACA ACCCTGACAC CCATCACCAC CACCACCACG GTGACACCAA CCCCAACACC
4861 CACCGGCACA CAGACCCCAA CATCGACACC CATCACCACC ACCACTACGG TGACCCCAAC
4921 CCCAACACCC ACCGGCACAC AGACCCCAAC CCCGACACCC ATCTCCACCA CCAGTACGGT
4981 GACCCCAACC CCAACACCCA CCGGCACACA GACCCCAACC ATGACACCCA TCACCACCAC
5041 CACCACGGTG ACCCCAACCC CAACACCCAC CGGCACACAG ACCCCAACAA CGACACCCAT
5101 CAGCACCACC ACCACGGTGA CCCCAACCCC AACACCCACC GGCACACAGA ACCCAACATC
5161 GACACCCATC ACCACCACCA CTACGGTGAC CCCAACCCCA ACACCCACCG GCACACAGAC
5221 CCCAACCATG ACACCCATCA CCACCACCAC CACGGTGACC CCAACCCCAA CACCCACTGG
5281 CACACAGGCC CCAACCCCAA CAGCCATCAC CACCACCACT ACGGTGACCC CAACCCCAAC
5341 ACCCACCGGC ACACAGACCC CAACCACGAC ACCCATCACC ACCACCACCA CGGTGACCCC
5401 AACCCCAATA CCCACCGGCA CACAGAGTAC AACCCTGACA CCCATCACCA CCACCACCAC
5461 GGTGACACCA ACCCCAACAC CCACCGGCAC ACAGACCCCA ACCCCGATAC CCATCTCCAC
5521 CACCACTACG GTGACCCCAA CCCCAACACC CACCGGCACA CAGACCCCAA CCATGACACC
5581 CATCACCACC ACCACCACGG TGACCCCAAC CCCAACACCC ACCGGCACAC AGACCCCAAC
5641 AACGACACCC ATCAGCACCA CCACCACGGT GACCCCAACC CCAACACCCA CCGGCACACA
5701 GACCCCAACA TCGACACCCA TCACCACCAC CACTACGGTG ACCCCAACCC CAATACCCAC
5761 CGGCACACAG ACCCCAACCA CGACACCCAT CACCACCACC ACCACGGTGA CCCCAACCCC
5821 AACACCCACT GGCACACAGG CCCCAACCCC AACAGCCATC ACCACCACCA CTACGGTGAC
5881 CCCAACCCCA ACACCCACCG GCACACAGAC CCCAACCACG ACACCCATCA CCACCACCAC
5941 CACGGTGACC CCAACCCCAA TACCCACCGG CACACAGAGT ACAACCCTGA CACCCATCAC
6001 CACCACCACC ACGGTGACAC CAACCCCAAC ACCCACCAGC ACACAGACCC CAACCCCGAC
6061 ACCCATCTCC ACCACCACTA CGGTGACCCC AACCCCAACA CCCACCGGCA CACAGACCCC
6121 AACCATGACA CCCATCACCA CCACCACCAC GGTGACCCCA ACCCCAACAC CCACCGGCAC
6181 ACAGACCCCA ACAACGACAC CCATCAGCAC CACCACCACG GTGACCCCAA CCCCAACACC
6241 CACCGGCACA CAGACCCCAA CATCGACACC CATCACCACC ACCACTACAG TGACCCCAAC
6301 CCCAACATCC ACCGGCACAC AGACCCCAAC CACGACACCC ATCACCACCA CCACCACGGT
6361 GACCCCAACC CCAACACCCA CTGGCACACA GGCCCCAACC CCAACAGCCA TCACCACCAC
6421 CAGTACGGTG ACCCCAACCC CAACACCCAC CGGCACACAG ACCCCAACCA CGACACCCAT
6481 CACCACCACC ACTACGGTGA CACCAACCCC AACACCCACC GGCACACAGT CCCCAACCCC
6541 AACAGCCATC ACCACCACCA CTACGGTGAC CCCAACCCCA ACACCCACCG GCACACAGAC
6601 CCCAACATCG ACACCCATCA CCACCACCAC TACGGTGACC CCAACCCCAA CACCCACCGG
6661 CACACAGACC CCAACCCCGA CACCCATCTC CACCACCACT ACGGTGACCC CAACCCCAAC
6721 ACCCACCGGC ACACAGACCC CAACCACGAC ACCCATCACC ACCACCACCA CGGTGACCCC
6781 AACCCCGACA CCCACCGGCA CACAGACCCC AACCACGGTA CTCATCACCA CCACCACTAC
6841 GATGACCCCA ACCCCAACAC CCACCAGCAC AAAGAGTACA ACCGTGACAC CCATCACCAC
6901 CACAACTACG GTGACCGCAA CCCCAACACC CACCGGCACA CAGACCCCAA CCATGATACC
6961 CATCAGCACC ACCACTACGG TGACCCCAAC CCCAACACCC ACCACTGGAA GCACGGGGCC
7021 CCCCACCCAC ACAA

Figure S4. Genomic sequence for BAC RP13-870H17 (Vector sequence removed, 8804bp)

1 gatcctccag ttgatcctac cacagatggt gcagaagggg ctctcgggca gatgggggtg
61 gcccccggag gacggtgggc ttcgccagag acggaacagt cctaatggag ggagggcagg
121 ggcccaaagc cagaggcagc ttctcttacc agcccagacc ctgctggggg cagccctgcc
181 caactgcaga tcccaggggc ccacagggtg cagatgtggg tcgggtgcct actaagggac
241 tggggagagg caaaagcagt gctcagggac cagcctctgt gccttgacaa gaaccggttt
301 atttggagtc tctgaccggg tgacgccctc tcccaaggac aggctgctgg ggatgggtgt
361 ggtgctggtg ggggccttct gcctggctgg gagcgggggc ggctgccctg ccctcgctgc
421 caccctctgc cctccctgac tctggggatc tcctcttctc tgtgctggtg gctgcctggg
481 cctcttgccc ccatctcggc acaggtttcc gtgccctcct cgcccctgat gggtctggtc
541 gagcgcctgc tctgtggctt catctgcagg gttctgggcc cagcagggct ggggccaagt
601 tcaggggctg ggaggtgttg gacggtgggc aggggtggtc gggagtgccc tgtgtgcctg
661 ggaggtccgt gaccactgtc cgcctcagtc cctctctgct ggctcagggt ctgcaggagt
721 gtggtagtct gagcccctgc ttggcagccc ctctccaacc ggtgccccag ggagccacgg
781 cccagggcac ttgggggcca ggcctggtga ccaggaaagc agctgctggc acaagcggga
841 agggggctgg tgggtgtttt cctgtctgtc atctgcagtc cttcagcccc aggagagcag
901 gcagcgaccc tgctagtctc cacaggccac agagctgcac acgcagtggc tgaacacctg
961 cagggtgagt acgagccgcc ggccaggcgt gctgggatcg gggcagggca gctccagctg
1021 ctgctcatag gagtggaggg ggcggcagca gctgcagcgg gcatccacct gctgggtgat
1081 gatgttgaag ctgccgagaa gtcaagacag agcagggtca tgactgctgc aggggcataa
1141 ggcccctccc tccccagggc agctgctccg cagaggcctg gacctccccg ctgagctccc
1201 ggctcacagg ggccagggcg gtgttgggca gatggagcgc agagtggctc acctgatggg
1261 gcagcaggcg cctgggtggg gtgcccgaac ctctctgggg tggggttgtg agcaccttgg
1321 tggacgtggg gcaggcctcc cacctgtgga ctcacctggc agcggaaatg caggcgccct
1381 cacagcgggt taccgtcacg ttcgccatgc accccttgaa cgtgatctcc tcctgctgct
1441 cccgcacact gcagacccct ggtagccgag tggacggtca gcagcgccca gggtgggcat
1501 ggagcgagga gggagggaaa ccctggctag agaccggggt ccccacctgt ctctggactc
1561 tcctgcccaa ggtgtggtct ccccttgtgg agccccacag agctcagacc tcagccatac
1621 acaaaggcaa aggccagccg catcctagtt tgtttttcc cctcaaagtc gctctgctgg
1681 agtccataag gtagagaatt ttctcagcgg actcagcaga tactgagccc tgggcctgtg
1741 tgagccaccc ttgggcccca gcttccctga tgaccaaccc caggcctcaa cactgacctg
1801 tgggcatggg cctctctggt tggccagacc caggacctgc agggggccg aggacaccgt
1861 gccagtgacc ccagagcggg acatatggtg ttgcaggagc tccgtgtgcc ccagctgggg
1921 aggagggaag tggagcaggc cccgtggtct gggctcctgg aaggggcggg gtgggggccc
1981 gcggggaagg cccagagaca gccagcctca gcgagaggcc tgggtacgtg gcaggcagca
2041 cagagcaggg tggaccgatg caggcagtgg cagcgttggc attagaaacc gccactggag
2101 agcgtgggtt taaaagtgga aaaggagtcc tggggtaaag gctggactct gctgcccgtg
2161 tgccctcccc tggctgctcc cagcttcgtt tcctggctgc agagaagaca ggtcacctgg
2221 agcctacctg cccgctgcag agggctttct ccgggagggt caggactctg ggtgcttcca
2281 taggccctgc cgtccccagc tgccctcccc tcccagctgg ttcctgctcc ctcgcctctg
2341 cttcttgggg tcacctccct ggttacacct tgcacccaag tcccggtctc agggagggac
2401 ccgaaccaag attccctcag cttgcagcaa cgagctgcca cccacgtgag agaccacgaa
2461 ggcaactgtg gcgttgtcgc ctggtggagg tcgatgcatg cgagggaccc agtccctggg
2521 tggttgctgt gagaggaaca cccaggaggg aagggaggga catccctgga acgcgaggtg
2581 aggtgaggtg gacatggata aggcctgagg tgtgctctgt ccccaggcat cccatggcct
2641 ggggagcagt ggactcgctc acaggcatgg gccgggaaga gccgtggccc agagaattgg
2701 catgagcacg aaggagcagg tgccctggtt gccaggtcca gctccacctt caacaggtcc
2761 tttggggcga ggcaggggat gtgccagact tttgtgtctc tctctgcact tggaggaagg
2821 gctgtgctgc ttgaaccctg gggttgggcc agccctgaca tcctctgagc aaccaaaggg
2881 gcagccaggg aaggcttttg gcaggtaaca ctctgagggg aggccaggag tgcagcagtg
2941 accgggcatg agtgagccag tgcagggaaa ggtcaaggtt agctggggaa caaggcccaa
3001 cggggaggcc aggcacttgc ttggccagga cagcgttggc atagagggtg cccaggagag
3061 gagagtggaa acgcctggca ggtgtgtagg gaaggcaggg aacaggcgtg tggtcctggg
3121 atcacaggac catgaggggt aagctgaggc aggggctgcc tgggagtcat gagcacagag
3181 gtgaggccag gagaagggcc acagagatgc cacttaccgg gtgaggtggg cgtaggtgtc
3241 ccgagagaag ataccggggc agaagcaggg gtgcttccat gggcagtgag ggagctggtc
3301 aggaaccgtg tggtaggcga caaggtggga ccagggtgcc tggtggtaag gttggtgact

3361 ggagaggtgg ggatacccgt caccccсgag gtgagtgaca caaagcctga tgtgggaact
3421 cgggtggtga gagaagtgga ccgcgaggtg gtggactgag aggagaaggc aggggcggtg
3481 tgggtgctgg ccgtggtcct gggcgtggac ggaaatgcaa tggtgctgga ggctaggtgg
3541 ctggatgggg tgggagacac ggtaacagtg gatatgggga gtagagcaga gagggtgaaa
3601 ggagaggaga tagtgtgggg gagagtggcc ctaatggtag tagaggcagc tggagaagaa
3661 ggaaaaagag gagatgcaga cactgatgca gtcgtgggat gagtggacaa tgaggagtgt
3721 gaccccgagc tcagggttgt ggagtgcacg gggcggaca cgaaagagga agatgtgcca
3781 acagaaggcg atgaagtctg ggagaggag tgggaggagg gcacataaga agaaacagta
3841 gaggggggcag aaggactggg agaaaatgag gaggacagct gattagttgt ggaaacagga
3901 gtggttgcag aactcaagtg gggagttgt gtggtgatag gtgatgacgg tggccttgag
3961 ctagagttct gaggcagcca agacgaggag gatatgaagg aagaagaggc tgtagctgtg
4021 ctgaatgagc tgtgggtttg gctggtccca ctggtggtca ctgtcattgg tggggctgtg
4081 tgggtggacc ctgtggcctt gagcgttgtt ggtggaggaa cggtgcctgt tggcgttgag
4141 tggatggagg cagaagtggc catctgtgtg tgggtagtga tgatgactgt gtgagtactt
4201 ggagtcacca aggaggtgga gaaaggtgga acgtgagtgg gaagtgtggt ctgagggtgt
4261 gatggggttg gataggtagt ggtggtctgg aaggatgttg cagtcatagg acctgtggaa
4321 gagaagggac tgctccctgt aggtggggag tgtgtggtga agggtgtggg tagcctgctg
4381 ctgctggccg aggtggtgtg ggccacaggg gttctggtgc ctgtactggt gtgtttgggg
4441 gtgatgttgg tggtagaagt tggggtgact tcaggatggt gtgttgagga agtgtggtaa
4501 ggtagggatg tagaagtttt ggccgtgcta aatgagcttg ggattggct ggtcccactg
4561 gtggtcggtg tcattggtgg ggctgtgtgg gtggaccctg tggccttgat cgtggtcggt
4621 ggaggaatgg tgcctgttgg cattgagtgg atggaggcag aagtggccat ctgtgtgtgg
4681 gtagggatga tgaccgtgtg agtacttgga gtcaccaagg aggtggagaa agatggaacg
4741 tgagtgggaa gtgtggtgtg agggtgtgat ggggttggat aggtagtggt ggtctggaag
4801 gatgttgcag tgacaggacc tgtggaagag atgggagtgg tccctgtagg tggggagtgt
4861 gtggtgaagg gtgtgggtag cctgctgctg gtggccgacg tggtgtgggc cacaggggtt
4921 ctggtgcctg tactggtgtg gttgggggtg atgctggtgg tagaagttga ggtgacttca
4981 ggatggtgtg tggaggaagt gtgtgaatgt agggatgtag aggttttggc cgtgctaaat
5041 gagcttcggg attggctggt cccactggtg gtcactgtca ttggtggggt tcctgtactg
5101 gtggggttgg gggtgatgtt ggtggtagaa gttgaggtgg cttcagcatg gtgtgtggag
5161 gaagtgtgtg aatgtaggga tgtagaggtt ttggctgtgt ttaatgagct cagggcttgg
5221 ctggtcccgc tggtggtcag cgtcattgtt ggcgctgtgt gggtggaccc tgtggccttg
5281 agcgttgtcg gtggaggaat cgtgcctgtt ggcattgagt ggatggaggc agaagtggcc
5341 atctgtgcat gggtaggggt gatgactgtg tgagtacttg gagtcaccaa agaggtggag
5401 aaaggtggaa cgtgagtggg aagtgtggtc tgagggtgtg atggggttgg ataggtagtg
5461 gtggtctgga aggatgttgc agtcatagga cctgtggaag agaagggact gctccctgta
5521 ggtgaggagt gtgtggtgaa gggtgtggtt agcctgctgc tggtggctga ggtggtgtgg
5581 gccacagggg tgccggttcc tggactggtg ggattggggg tgatggtggt agaagttggg
5641 gtgacttcag gaaggtgtgt ggaggaagtt tgtgaatgta gggatgtaga ggttttggct
5701 gtgttgaatg agctcagggc ttggctcgtc ccgctggtgg tcggcgtcat tgttggcgct
5761 gtgtgggtgg accctgtggc cttgagcgtt gttggtggag gaacggtgcc tgttggcgtt
5821 gagtggatgg aggcagaagt ggccatctgt gtgtgggtag tgatgatgac tgtgtgagta
5881 cttggagtca ccaaggaggt ggagaaaggt ggaaggtgag tgggaagtgt ggtctgaggg
5941 tgtgatgggg ttggataggt agtggtggtc tgaaaggatg ttgcagtcat aggacctgtg
6001 gaagagatgg gactgctccc tgtaggtggg gaatgtgtgg tgaagggtat gggtagcctg
6061 ctgctggtgg ccgaggtggt gtgggccaca ggggttctgg tgcctgtact ggtgtggttg
6121 ggggtgatgg tggtggtaga agttggggtg acttcaggat gatgtgttga ggaaatgtgt
6181 gaatgtaggg atgtagaagt tttggccgtg ctaaatgagc ttggggattg gctggtccca
6241 ctggtggttg ccgtcattgg tggggctgtg tgggtggacc ctgtggcctt gatcgtggtc
6301 ggtggaggaa tagtgcctgt tggcattgag tggatggagg cagaagtggc catctgtgcg
6361 tgggtagggg tgatgactat gtgagtactt ggagtcacca aggaggtgga gaaagatgga
6421 acgtgagtgg gaagtgtggt gtgagggtgt gatggggttg gatcggtagt ggtggtctgg
6481 aaggatgttg cagtgacagg acctgtggaa gagatgggag tggtccctgt aggtggggag
6541 tgtgtggtga agggtgtggg tagcctgctg ctggtggccg acgtggtgtg ggccacaggg
6601 gttctggtgc ctgtactggt gtggttgggg gtgatgctgg tggtagaagt tgaggtgact
6661 tcaggatggt gtgtggagga agtgtgtgaa tgtagggatg tagaggtttt ggccgtgcta
6721 aatgagcttc gggattggct ggtcccactg gtggtcactg tcattggtgg ggttcctgta
6781 ctggtggggt tgggggtgat gttggtggta gaagttgagg tggcttcagc atggtgtgtg

6841 gaggaagtgt gtgaatgtag ggatgtagag gttttggctg tgtttaatga gctcagggct
6901 tggctggtcc cgctggtggt cggcgtcatt gttggcgctg tgtgggtgga ccctgtggcc
6961 ttgagcgttg tcggtggagg aatcgtgcct gttggcattg agtggatgga ggcagaagtg
7021 gccatctgtg catgggtagg ggtgatgact gtgtgagtac ttggagtcac caaagaggtg
7081 gagaaaggtg gaacgtgagt gggaagtgtg gtctgagggt gtgatggggt tggataggta
7141 gtggtggtct ggaaggatgt tgcagtcata ggacctgtgg aagagaaggg actgctccct
7201 gtaggtgagg agtgtgtggt gaagggtgtg gttagcctgc tgctggtggc tgaggtggtg
7261 tgggccacag gggtgccggt tcctgtactg gtgggattgg gggtgatggc ggtagaagtt
7321 ggggtgactt caggaaggtg tgtggaggaa gtttgtgaat gtagggatgt agaggttttg
7381 gctgtgttga atgagctcag ggcttggctc gtcccgctgg tggtcggcgt cattgttggc
7441 gctgtgtggg tggaccctgt ggccttgagc gttgttggtg gaggaacggt gcctgttggc
7501 gttgagtgga tggaggcaga agtggccatc tgtgtgtggg tagtgatgat gactgtgtga
7561 gtacttggag tcaccaagga ggtggagaaa ggtggaaggt gagtgggaag tgtggtctga
7621 gggtgtgatg gggttggata ggtagtggtg gtctgaaagg atgttgcagt cataggacct
7681 gtggaagaga tgggactgct ccctgtaggt ggggaatgtg tggtgaaggg tatgggtagc
7741 ctgctgctgg tggccgaggt ggtgtgggcc acaggggttc tggtgcctgt actggtgtgg
7801 ttgggggtga tggtggtggt agaagttggg gtgacttcag gatagtgtgt ggaggaagtg
7861 tgtgaatgta aggatgtaga ggttttgcct gtgctaaatg agcttgcgga tgggctggtc
7921 ccactggtgg tcactgtgat tggtggggct gtgtgggtgg accctgtgac cttgagcgtt
7981 gttagtggag gaatggtgcc tgttggcgtt gagtggatgg aggcagaagt ggacatctgt
8041 gcatgggtag gggtgatgac cttgtgagta cttggagtca ccaaggaggt ggagaaaggt
8101 ggaatgtgag tgggaagtgt ggtctgaggg tgtgatgggg ttggataggt agtggtggtc
8161 tgaaaggatg gtgcagtcat aggacctgtg gaagagatgg gactgctccc tgtaggtggg
8221 gaatgtgtgg tgaagggtgt gggtagtctg ctgctggtgg ccgaggtggt gtgggccaca
8281 ggggttctgg tgcctgtact ggtgtgtttg gggtgatgt tggtggtaga agttggggtg
8341 acttcaggat ggtgtgtgga ggaagtgtgg taaggtaggg atgtagaagt tttggccgtg
8401 ctaaatgagc ttagggattg gctggtccca ctggtggtcg gtgtcattgg tggggctgtg
8461 tgggtggacc ctgtggcctt gatcgtggtc ggtggaggaa tggtgcctgt ttgcattgag
8521 tggatggagg cagaagtggc catctgtgcg tgggtagggg tgatgactat gtgagtactt
8581 ggagtcacca aggaggtgga gaaagatgga acgtgagtgg gaagtgtggt ctgagggtgt
8641 gatggtgttg gataggtagt ggtggcatgg aaggatgttg cagtgacagg acctgtggaa
8701 gagatgggag tggtccctgt aggtggggag tgtgtggtga agggtgtggt tagcctgctg
8761 ctggtggctg aggtggtgtg ggccacgggt gtccaggttc ctatactggt ggggttgagg
8821 gtgatgttgg tgatagaagt tggggtgact tcaggatggt gtgtggagga agtgtgtgaa
8881 tgtagggatg tagaggtttt ggctgtgttg aatgagcttg aggcttggct ggtcccactg
8941 gtggttggca tcattggtgg ggctgtgtgg gtggaccctg tggccttcag cgttgtcggt
9001 ggaggaatgg tgcctgttgg cattgagtgg atggaggcag aagtggtcat ctgtgcgtgg
9061 gtaggggtga tgactgtgtg agtacttgga gtcaccaagg aggtggagaa agatggaacg
9121 tgagtgggaa gtgtggtctg agggtgtgat ggtgttggat aggtagtggt ggcatggaag
9181 gatgttgcag tgacaggacc tgtggaagag atgggactgc tccctgtagg tggggagtgt
9241 gtggtgaagg gtgtggttag cctgctgctg gtggctgagg tggtgtgggc cacaggggtg
9301 acggtttctg tactggtggg attgggggtg atggtggtag aagttggggt gacttcagga
9361 tggtgtgcag aggaagtgtg tgaatgtaag gatgtagagg ttttggctgt gctgaatgag
9421 ctcggggctt ggctggtccc actggtggtc ggcgtcattg gtggggctgt gtgggtagac
9481 cctgtggcct tgagcgttgt tggtggagga atggtgcctg ttggcgttga gtggatggag
9541 gcagaagtgg acatctgtgc gtgggtagtg gtgatgactg tgtgagtact tggagtcacc
9601 aaggaggtgg agaaaggtgg aatgtgagtg ggaagtgtgg tgtgagggtg tgatggggtt
9661 ggataggtag tagtggtctg gaaggatgtt gcagtcatag gacctgtgga agagaaggga
9721 ctgctccctg taggtaagga gtgtgtggtg aagggtgtgg gtagcctgct gctggtggct
9781 gacgtggtgt cggccacagg ggtgccggtt tctgtactgg tgggtttggg ggtgatggtg
9841 gtagaagttg gggtgacttc aggatggtgt gtggaggaag tgtgtgaatg tagggatgta
9901 gaggttttgg ctgtgctgaa tgagctcagg gcttggctgg tcccactggt ggtcggcgtc
9961 attggtgggg ctgtgtgggt ggaccctgtg gccttgagcg ttgtcagtgg aggaatggtg
10021 cctgttggcg ttgagtggat ggaggcagaa gtggccatct gttgatgggt tggggtgatg
10081 actgtgtgtg tacttggagt caccaaggag gtggagaaag gtggaatgtg agtgggaagt
10141 gtggtctgag actgtgatgt ggttggatag gtagtggtgg tctggaagga tgttgcagtg
10201 acaggagctg tggaagagat gggactgctc cctgtcggtg gggagtgtgt ggtgaagggt
10261 gtgggtagcc tgctgctggt ggccgaagtg gtgtgggcca caggggtcct ggtgcctgta

```
10321 ctggtgtggt tgggggtgat gttggtggta gaagttgggg tgactgcagg atggtgtgtg
10381 gaggaagtgt gtgaatgtag ggatgtagaa gttttggctg tgctgaatga gcttggggct
10441 tgcctggtcc cactggtggt cggcgtcatt ggtggggctg tgtgggtgga ccctgtggcc
10501 ttgagcgttg tcggtggagg aatggtgcct gttggcgttg agtggatgga ggcagaagtg
10561 gacatctgtg catgggtatg ggtgatgact gtgtgagtat ttggagtcac caaggaggtg
10621 gaggaaggtg gaacgtgagt gggaagtgtg gtgtgagagt gtgatggggt tggataggta
10681 gtggtggtct ggaaggatgt tgcagtgaca ggacctgtgg aagagatggg actgcttcct
10741 gtaggtgggg agtgtgtggt gaagggtgtg ggtagcttgc tgctggtggc cgaggtggtg
10801 tgggccacag gggttcttgt gcctgtactg gtgtgtttgg gggtgatgtt ggtggtagaa
10861 gttggggtga cttcaggatg gtgtgtggag gaagtgtggt aaggtaggga tgtagaagtt
10921 ttggccatgc taaatgagct tggggattgg ctggtcccac tggtggtcgg cgtcattggt
10981 ggggctgtgt ggatggaccc tgtggccttg agcgttgttg gtggagaaat ggtgcctgtt
11041 ggcgttgagt ggatggacgc agaagtggcc atctgtgcgt gggtaggggt gatgactgtg
11101 tgagtacttg gagtcaccaa ggaggtggag aaagatgtaa cgtgagtggg atgtgtggtc
11161 tgagggtgtg atggtgttgg ataggtagtg gtggcatgga aggatgttgc agtgacagga
11221 cctgtggaag agatgggagt gctccctgta ggtggggagt gtgtggtgaa gggtgtggtt
11281 agcctgctgc tggtggctga ggtggtgtgg gccacaggtg tcccagttcc tgtactggtg
11341 gggttggggg tgatgttggt ggtagaagtt gaggtggctt caggatggtg tgtggaggaa
11401 gtgtgtgaat gtagggatgt agaggttttg gctgtgttta atgagctcag ggcttggctg
11461 gtcccgctgg tggtcagcgt ccttgttggc gctgtgtggg tggaccctgt ggccttgagc
11521 gttgtcggtg gaggaatggt gcctgttggc gttgagtgga tggaggcaga agtggccatc
11581 tgtgtgcggg gaggggtgat gactgtgtga gtacttggag tcaccaagga ggtggagaaa
11641 ggtggaacgt aagtgggaag agtggtctga gagagtgatg gtgttggata ggtagtggtg
11701 gtctggaagg atgttgcagt cataggacct gtggaagaga caggactgct ccctgtagat
11761 ggggagtgtg tggtgaatgg tgtagttagc ctgctgctgg tggctgaggt agtgtgggcc
11821 atgggtgtgc tgcttcctat actggtgggg ttggtggtga ttttggtggt agcagttggg
11881 gtgacttcag gatggtgtgt ggaggaagtg tgtgaatgta aggatgtaga ggttttggct
11941 gtgctgaatg agctcgcggc ttggctcgtc ccactggtgg tcggcgtcac tggtggggct
12001 gtgtgtgtgg accctgtgtt catgagcgtt gtcagtggag gaatggtgcc tgttggcgtt
12061 gaatggatgg aggcggaagt ggccatctgt gcgtgggtag gggtgatgac tgtgtgagta
12121 cttggagtca ccaaggaggt ggagaaaggt ggaacgtgag tgggaagtgt ggtctgaggg
12181 tgtgatgggg ttggatatgt agtggtggtc tggaaggatg ttgcagtcat aggacctgtg
12241 gaagagatgg gactgctccc tgtaggtggg gagtgtgtgg tgaagggtgt ggttagcctg
12301 ctgctggtgg ctgaggtggt gtttgccaca ggcgttctga tgcctgtatt ggtggggttg
12361 ggggtgatgg tggtggtaga agttggggtg acttcaggat ggagtgttga agaagcatgt
12421 gagtgtaggg atgtggaggt tttggctgtg ctgaaagagc tgtgcgcttg gctggtcccg
12481 ctggtggtca ctgtcattcg tggtgctgtg tgcgtggacc ctgtgcctgt ggcctttacc
12541 gttgtaggtg gaggaatggt gcctgttggt gttgagtgga tggaggcaga agtggacact
12601 tgtgcgtggg taggggtgat gactgtgtga gtacttggag tcaccaagga ggtggagaaa
12661 ggtggaatgt gagtgggaag tgtggtctga gggtgtgatg ggggttggatg ggtagtggtg
12721 gtctgggagg atgttgcagt cataggacct gtggaagaga catgactgct ccctgtaggt
12781 gggaagtgtg tggtgaaggt tgtgggtagc ctgctgctgg tggccaaggt ggtgtgggcc
12841 acaggggtgc tggttcctgc actagtggac ttgggagtaa tgttggtggt agaagttggt
12901 gtggtttcag gatggtgtgt ggaggaagca tgtgagtgga gagatgtaga agttttggct
12961 gtgctgaatg agttgtgagc tttgcttgtc tgaataatgg tccccgtcat tggtgggcct
13021 gtgtgtgtcg accctgtggg catgcgcgtt gtcagtggag gaacggtgcc tgttggcgtt
13081 gagtggatcg aagcagaagt ggacatttgt gcgtgggtag gggtgatgac tgtgtgagta
13141 cttggagtga ctgatgaggt ggagaaaggt ggaacatgag tggtaagtgt ggtctgaggg
13201 tgtgatgggg ttggataggt cgtggtggtc ttgatggatg ttgcagtcat aggacctgtg
13261 gaagagatgg gactgctccc tgtaggtggg gagtgtgtgg tgaagggtgt gggtggcctg
13321 ctgctggtgg ccaaggtggt gtgggccaca ggggtgctgg ttcctgcact agtggacttg
13381 ggagtaatgt tggtggtaga agttggtgtg gtttccggat ggtgtgtgga ggaagcatgt
13441 gagaggatgg atgtagaggt tttggctatg ctgaatgagc tgtgggcttg ggtggtccga
13501 atggttgtcc ccgtcattgg tgaggctgtg tgtgtggacc ctgtggccgt gagcgttgtc
13561 agtggaggaa tggtacctgt tggcgttgag tggatcgagg cagaagtgga catctgtgca
13621 tgggtagggg tgatgactgt gtgagtactt ggagtcactg acgagctgga gaaaggtgga
13681 acgtgagtgg gaagtgtggt ctgagggtgt gatgtggttg gataggtagt ggtggtcttg
13741 aaggatgttg gagtcatagg acctgtggga gagaggggac tgctctctgt aggtggggag
```

13801 tgtgtggtga agggtggtgg tggcctgctg ctggtggctg aggtggtgtg ggccacaggg
13861 gttccggtgc ctgtactagt ggggttggga gtaatggtgg tggtagaatt tgtggtgatt
13921 tctggatggt gtgtggagga agcatgggag aggagggatg tagaggtttt ggctgtgctg
13981 aaggagctgt ggacttggct ggtcctactg gtggtcactg ttattagtgg ggctgtgtgt
14041 gtggaccctg tggccatgag cgttgtcagt ggaggaatgg tgcctgttga cgttgagtgg
14101 ttggaggcag aagtggacat ctgtgggtgg gttggggtga tgactgtgtg agtacttgga
14161 gtcaccgatg aggtggagaa aggtggaaca tgaatgggaa gtgtgatgtg agcttgtgat
14221 ggggttggat aggtagtggt ggtcttgaga gatgttgcag tcatacgacc tgtggaagag
14281 aggggactgc tcctggtagg tgaagagtgt gttgtgatgg gtgtgggtgg cctgctactg
14341 gtggccgagg tggtgtgggt cacaggggtg ctggtgcctc tactggtgga cttgggagtc
14401 acgttggtgg tagaagttgg ggtgacttca ggatggtgtg tggaggaagc atgtgagtgg
14461 agggatgtag aggttttggc tgtgctgatt gagctgtggg cttggctgat cctactggtg
14521 gttgccgtca ttagtggggc tgtgtaggtg gaccttgtgg ccttaactgt tgttggtgga
14581 gcaatcgtgc ctgttggcgt tgagtggata taggcagaag tggacatctg tgggtgggta
14641 ggggtgatga ctgtgtgagt aattggagtc accaaagagg ttgagaaagg tggaacgtga
14701 gtgagaagag tggtctgagg gagtgatggg gttggatagg tagtggtggt cttgaaggat
14761 gttgccgtca tgggacctgt ggaagagaag ggactgctcc ctgtaggtgg ggagtgtgtg
14821 gtgaagggtg gtggtggcct gctgctggtg gctgagttgg tgtgggccac aggggttctg
14881 gtgcgtgtac tagtggggtt gggagtaatc gtggtagtag aagttggggt gacttcagga
14941 tggtgtgtgg aggaagtgtg tgaatgtagc gaggtaggtg ttttgtttgt gctgaatgag
15001 ctgtgggctt ggctggtccc actggtggtc ggcgttattg gtggggctgt gtgggtggac
15061 cctgtggcct tgagcgttgt tggtggagga atggtacctg ttggcgctga gtggttggag
15121 gcagatgtgg ccatctgtgc gtgggtaggg gtgatgactg tgtgagtact tggagtcacc
15181 aaggaggtgg agaaaggtgg aacgtgagtg ggaagtgtgg tctcagggtg tgatggggtt
15241 ggataggtag tggtggcatg gaaagatgtt gcagtgacag gacctgtgga agggacggga
15301 ctccccgccg taggcgggga gtgtgtggtg tgtggggttt ggggcgttgt gtattcagta
15361 gtcgttcttg tttgagtggt ctctgtggct gtgggcctcg tgggttgtcc tggctgtggg
15421 gtggttgggc ctgtggtgct tgctggggtt ggacgtgggc ctgtcgtctg ggtggccgtt
15481 gttcctggca gttcctgatt ggtcgatttt gctgtgggaa ttggtgaagt tgtcatcgtt
15541 attgtttttg tttctctacc ctgacctccg ctggcccgtc cttttgtcta tgtgaccttt
15601 ttcttgcctg tctgtgcctc tgttcctgtg acatggcccc tgctgggcac tccagcctgc
15661 cccattgtct ccatctcacc tggacttgct ccacatcctg ccctgagctg cgtgctgact
15721 ccttcagtgc agctgccgct ctgtgactgg aacccaggcc ctactttatc cccttctggt
15781 tccctgtggc cgtggtggtg gcccccaccc tcctgcacct ctgctcccca ggcctgcctt
15841 tgtgagtgcc aggcggcctt ccttgcctcc agctccagcc tacacttttg ggctgccttc
15901 tcgcttgccc tctgggaaat acggggtctt ctttatggct gaatcactga atgtgagctg
15961 gtggtgggac cgggtgcctt cggcagtgct ggcatcccat ggcgccatga cttacgcagc
16021 gtggggcttg tccctgatgt ggctggggtt ggtagtgtca ttgtggtccg tgttgtggac
16081 tgagctgtgg acgtcgtggc tgggctggcg gtcgacgccg tggccctggt tgtggcctgg
16141 gtcactgtgg gttttgtggc tgtcgatctc agtgtggctg tgggaggcag ccctgatgtg
16201 gcttgtgggg tgacggccgt ggttggtcta ggtggttctg cagaggacag ccgcccggaa
16261 catccccttg ctgtgggcct gcatttcgaa ggcttgtgtc ccagccccct gccctgcttc
16321 tgggatccct ggcctgctgt ccgggactca gcctccttgg aggggctctt cctcttgcct
16381 ttgttaggtc ctcccaccgt actcctggct gtggtggcca gagctggggc agtgaccaca
16441 tgcttatggg gctgcggctg ctcctgcgcc cacccttgct tagctgaacg gactgtcgtc
16501 ctgtcccctc cagggtccct gggcagcaga tggggccctg ctcggtgtgg ggcaggggca
16561 ggctgcctgg cagaggccct gcaggtccca cacggtttct agtgacagca gccagcagca
16621 aggaatgccc catgccatga ccagcttgtc tttaaaagtt tttccgaaaa atccccagtt
16681 tggctcccaa gcataggaag ttctacgctg ggaatctgct tagtggcaga tgtgagccag
16741 gaagcagggc catccctaag cccaccccag aggtgtacct aggacctcct gcagctgccc
16801 tctccatggg ctcagctgga ggctccttac ctcccgagga ggctgtgggc ttggaggatg
16861 tgagcgtggc tggaaggagg ggtgtctggg tggggctggc aggggtgtga ttagagctgg
16921 gtgagggtcc ggtggagctg agaagcccga tggtggtgga ggttcccgtc atgggccaga
16981 cttgcgtggg ccgtgagcct gggtgggcgg acatgccatc agggctgcag ggtaccggca
17041 tatccttggg gagcaccctc ccctctgcct gcttggccct gaagccgggc agccctgcag
17101 gggccaatga tgtgcagttg agggctggcc tgtggcactc tgagtgcagc ctggctcctg
17161 gctgggcctc ctgcatggcg ggggtcacct gttgaggccc cactctgtgc tcatccgtgg
17221 gtccagccag ggccatgggg accaggctgc ttcctggctg tgggctgagc tcctccccaa

17281 ctctgattgc caggttagac ctgagaaggg cacaggtact gcctgccccc tccctgcttc
17341 ccacccgaca gatttgtcca gggaaccgag gggagcccag gaaagggcct gcgtcacctt
17401 ggcacctagt gtgcgtgcaa ttacctgtgg tgggcagctg cggcgtggtg ggtggctgcg
17461 gcgtggtggg cggcactgca agaagatggg gtcagctccc tgtggttctc taagcctccc
17521 caccccgtgg ggcctctggg agctccgagg gcctgagtca gagatgctca ccaaggctgt
17581 ggtggagggg actggagggg ctgggagccc accctcccct ctctcccttt ctccttccca
17641 ggatgattcc cactctatac cccaggcacc ctggcaggcc tggtgagggt agggggagct
17701 gggagctggt ggaagagggg atgggagggc ccagtggggt tcagtgctgt gtttttttct
17761 ctctgctgcc atgggctgga ggctgcctga gtctctggag acccaagggg ccagggtcct
17821 ggagagtgga gtcccagagc tcacgtaagg gctcacagcc cccgagggct ctctcccttg
17881 tttgtgggat gtggacgtgc gttctgcctg catgcaggca ggggcagggt tctcagggca
17941 gccgactgga cttactgcag ggcacgcaca ccccctcctc gtggtcgaag tactcatcct
18001 gggagcagtt gtagcagcct agggtggaga acggccaggg tctgtgtgac tggtggccag
18061 ccaggcccac ctgcgtgttt cctgccctgg cggcctcctt cctctctgct tttttttttt
18121 tttttttttg agacagagtc tcgctctgtc gcccaggctg gagtgcagtg gcacaatctt
18181 cgctcactgc aacctctgcc tcctggattc acatgattct cctgccccag cctcccaggt
18241 agctgcgatt gcaggcatgt gccatgacgc ccggctaatt tttgtatttt tagtagtgat
18301 ggggcttcgc cacgttggcc aggctggtct caaactcttg acctgaggtg atcttcctgc
18361 ctcggcctcc cagagtgctg ggattacagt ttcacccgcg caccttgctc taagcccctc
18421 ctttcctgcc atgccttcct caggccttgg tctctcatac cctgcttatc acccggggct
18481 ggggcttcgg tgccatcttc cagtcctcac ctccccctgg acctcagaca ctggcccctc
18541 ttctgtgctc cccgatgcag gtgccctgca ttgccctcct gagccccaac acatctgtcc
18601 tctgtgtgtc cggatggctc cagacaccac cctcctccac cacttgggcc aggacctgtt
18661 aatcccagga ccctctgcat tcagacctct gccttggggc agccacaggc ctcacaaaga
18721 cccctccctc ccggccacac cccaccccac aagtctgtgt accccacacc cctctgcagc
18781 ccggtgcccc tcactcgctc cctctgccct ctgcagcccc gcgcctctca ctcgctccct
18841 ctgccctctg cagccccgcg cccctcactc gctctgccct ctgcagccca cgcccctcac
18901 tcgctccctc tgccctctac agccccgcgc ccctcactca ctccctctgc cctctgcagc
18961 cctgtgcccc tcactcgctt gctctgccct ctgcagcccc gcgccccact cgctccttct
19021 gccctctaca gccccgcgcc cctcactcac tccatctgcc ctctacagcc ccgcacccct
19081 ccaccgctcc ctctgccctc tgcagcccgc gcccctcact cgctggctct gcccgctgcc
19141 ctggctttct taagggttct gggcacaagg ctgctcctgg ctgctcctct ggacactttc
19201 ctggctcccc tcctcacctt tcccatcctc gccctcacat ctgcctccca ccaagggccc
19261 caaccaccct gtgtggcaca gaagcctgtg gctctcccac cccagcctgc ctttctcctc
19321 tccatggtgg gcgcctcggc cgacactggg tctgcctcct taagcacctc tccctggctg
19381 ggagggaagc tccaggttgc aggactgttt gttttgtgta aggccaggtc ccctgacctg
19441 gagccggcct ggaggacaga ggtgctcagg acccatctgc tgagtgactg tgtgtgtgaa
19501 agaatgaatg tgcaaatgaa tgcatgaatg tgcgtgtgtg tatgagcgaa tgtgcatgaa
19561 tgaataagca aacatgaatg aatgaatgaa tatgtgtgaa tgaatgtgca tgggtgaatg
19621 tgcgtgagtg aatgttgaat gaatgtgcgt gaatgtgcat gagtgaatgt gcgtgagtga
19681 acaaatgtgc gtaaatgaat gcatgagtgc gtgaatgtgc atgaatgtgg gtgagtaaat
19741 gtgtgaatga gcatgaatga atgtgaatgt gttgagtgaa tgtgcgtgaa tgaatgtgaa
19801 tgagcgtgaa tatgttgaat gagtgtgtgt gggtgaatga atggatgaat ggagtgaatg
19861 tgcatgagtg tgcgtgaatg tgcgtgagtg aatgtgcgtg aatgaatgtg catgaatctg
19921 cgggaatgtg actgcgtgaa tgagcatgaa tgaatgtgtg tgtagtgact gcgtgaatga
19981 atgtgtgaat gaatgtgcat gagtgtgtgt gagtggatga acaaatgtgc gtgaatgaat
20041 gaatatgcgt gaatgaatgc atgaatctgc atgaatgaat gtgaatgtgc gtgagtgtgt
20101 gtgaatgaat gtgtgcaaat taatgaatgt gtgaattaat gagcatgaat gaatgtgtga
20161 atgaatgaat gcgtgtgaat gaatgaatgt gcatgaatga gtgaatgggt ccccctgtgt
20221 atctgcgttg cctcccggtc acctggcacc ttcgatgttg ctgcctggga cgctctgtgg
20281 ctggctgggg cagaggcagg gctggtagtg ccacgtgcag ttggcctcct gtgtgtactg
20341 gtactcgcca tggccgtcct gcgtgtgcgt gttgtagaag ccgcagtaga tggctgggag
20401 gaagggagct gtcagctggt ggggttcctg gccctggccc tggccctgac ccggtggttc
20461 ccctgggcat gcatggaacc tgagtgtggg cggggaaggt ctgggccctc ttggtgaagc
20521 ctcccctggg cagctggggg ctgcgggagg gccagggtgg ccccgcaggg cacagcccgg
20581 cgcctgtcca gggagagggg cctgtgggca agggcagccc tgcgccggcc cttagtggcg
20641 ctgggtggca ggggctggag caacctgtgg gaggggtggt cactcacggc agaaggccgg
20701 ggtcctccag tccacgcaca caccсttgtc cagacaggct tgggcgtagg cagccacggc

20761 atcgcacaga cactcacagt ccccgccact gtcacaccca catgcgtcgc gcacgcaggc
20821 ctcgtagtag ggcaggtggt atacctgcag gggtgtgtgc cagtcagtgt ctggctgccg
20881 ggggatggcg gggcatcagg ctttgccacc tgcagggccc tcagtgtggt caggccggag
20941 tgtggcggta agggcgctgg gactgggtga gcggcaccac gtggacctca gccctgaccg
21001 ctagccacgc tcccggagcc gatgctgcca cggaggcctg acccgagctc acgccttgag
21061 acccgccatc gggaccaaga tgccgctgcc gctaaccacg gccactgcag tcccacccag
21121 ggtctctgct ccccccacgt cacgctcaca ttcagcgggc cagtgccatg ctgttccccc
21181 gcggggtgcc cccatcctct cagttcttac tgcgccgcga tggccggatc acgccctggg
21241 ctgggaagtg cacgccctgc atcctggagg tctccctgac caccagctcc tgagcagggg
21301 gcccaccagg cgatgctgcc ccaggggact cgtggcactg tctgggggtg ctattggcat
21361 ctggtgggtg gaggccgggg ggctgcttaa cagccacagt gcacgagtcg gccccacatc
21421 agggccctgc actcaggcag agggtctgaa actctctgca ccctgacctg gctggtctgg
21481 gatcccacgg agggagaacc gtgcctggct tccccgcccc ttcccccgcc cccaccggac
21541 atttgtggag gggcaggcgc acagccctcg tgcccggtgc ccaccttgct gtggcaggtg
21601 gcaaaggtct ggctgttgat gacgctgcac ttgcgctcgg cccaggagcg ccggaaggca
21661 ttgagactgc aggggtctgt cacgaagctc acgtccccgc acagcgggct ctccttccac
21721 gagttcacca actccagctc gctggatgcc acgtacctgc tgcgcgtctc gaagtcgtcc
21781 ttcatgttcc cgttgaagtt gccacacaag ccgcagaggg gatcctgcag acggtggcat
21841 caggccgggc ccaggggccg tgccatctgt ctccacccct gcatcaggga gggcctggga
21901 ggaggcagag ggcgtgcggt acctgggagg cacgggcgat cctgatgagg atggtcatgt
21961 gcctgttcca gatgagcgtc aggttgtacc tcccggggat gctgatgtcc acgacaaggc
22021 tcagcgcacc cggcgtcacc ccgagctgca cgtggggctc ctccccggtg accgtgtagt
22081 ttctgtccgc cagcaccacg gacaggccct gtggggtggg gttggcatag gactgcctgt
22141 ctgtctcccc cggcccctgg cacagccgtg ctggaccgag ctctcagaga cagagctgcc
22201 caggtctgtt aaggccatgc acaggagcgc ctgctgagag ccagcttggg gcagaagggc
22261 ctggcatgcc tggaacaggg ctgagggctg aagctcgggc ttcctggaga gtccctcccc
22321 tctgcccggg agacattctg cagtccctga aaccttgtgg ggcacaagca cccgtggtcc
22381 tgagcgtctg gccagggagg ggcagcaggc accoctcaag gagaggcagg cggggagggc
22441 tgcatctcgg ggcaggacct ggaggctcca gtgcgcggga tccagctgtg tggcagggcc
22501 ccctaccgcc cgtcctgccc tgccagagtc tgcccggctg ctcaccccca ggaagatctt
22561 gatggcccgt gagcatgtga ccccggagtt cccacagatg acgttctctg tcaggatctt
22621 gaaggtgggc tgtgagtcgt tgacaccaca gacgtcctgc agggagaggg cgctgaggag
22681 gagccctgga ggccgtgcct ctgggtcccc ggcccctgcg gcctggcacc cgatggttac
22741 cgtggccagg atgtactcgc agttgccgtc gaatacgaag cgctggccgt cgaaggtgat
22801 gacgtggccc tccccgtaga gggtgcaggt ggatgggcag tgggtgccct gctgacaggc
22861 ccacctcccc cttgagcagg agctgtggag acagcaggtg tgggtggtgg gcctgcggcc
22921 ctcctgccat actgggtgtg gtccctggag aggccagact gcacctctgg acgcccccgc
22981 ctctgggaca gccccacccc gggcagcctc cgcctgcccc agatggcccc aggagcccag
23041 ggcgtctgaa gcgaggcttt gtctcaccag gtcctgcagt cagtgtggag ctcagctcct
23101 ccagggtagg agacccccga gaactcacat gggcactcct cgggggggcac acactgcccg
23161 tcggcattct cgtagaggcc ctcggcgcag acacagccag gctcacactt ggtgggcacc
23221 tggagggagg caggtcagca gctcctggga gggtggcctc agccagctgc tgcccagccc
23281 tgggcccctc tgagactgcc cggcgtgtct cccaggtcct ctccctgaat acagccctgc
23341 tctgtggcct ttgtgggcag ctgggcttac tgccttagca aagcttcccc cacctcgtgg
23401 aaggtcctgg gacccagtgc acacggctgg gctgctgtac ccactcccac acatgggcag
23461 cccaccctcc ccgagctggt gcccactgtc cttagggagc cctgcagacg agcccccggg
23521 gcctccagct gcctggccac agggccgctt ccacctcgtg gccagcccca ggcacccgct
23581 gcagggcaga atgcggccag gtctcccgga gttctgtgga aaccccctca tggttcagct
23641 ggggcccggg cattgtccct gctcctcgcg cgccccctta cgcaggcaac accggtggcc
23701 agcatctggc atgtgggggc acaggctgcc ccaaacttgt tctcggagga ctggctgcag
23761 gacttgaagg tcttaggggc ctggcaggag gctgcaggaa agaggggtgc gcggtcagga
23821 cactcagagg aagccggggc ccctcacagt cccagcctgc ggccagcgct gctgtacgta
23881 cccaggaaca tctgtggccg ctgcgggcaa ctcagccgcc cgttgatgca gtggctgcaa
23941 gagagaggct gcgtgagacc ccgggacctg gcagggaccc cccgcctggc ccctgcccgg
24001 tccctcacca ggtgatgccg ttgatgacag tggactgctc ggccaggatg aacttgtaac
24061 cctccagtat gcacgggcac tgggccttgc gcacacactc gcccttttgg ttcaggtagg
24121 tgccatcggg gcagttgcaa ccgtccacgg gcacggcgct gtggtggcac tcggtggcac
24181 ggtccgacag cgacaggcag gtgcgctcac aggcttggct gttgtagctg aaggtggtgt

24241 tacccgtgca ggggatggct gtgggggacc cgggcatcag actctccggg agggggcggc
24301 cgggagggca atctcgggtt cccctgcctg ccgggcactg cagagcctca gagcttgggg
24361 tcccaggaca ccccctcgtg agcccagcct gccgtgaccc cgcttaagcc ccgtcgggca
24421 ctcactgcag ttgtccacac tgcttctcca gccccagagc aggacgcccc gcaaggagca
24481 ggcgtgtacg tagtcgccca gggcggcaca gatgtgggga aaggtctcct cgtagttgca
24541 ggcctggtac acgcacctct gcgggcagag agccagcatg ggctggtggc aggcaccctg
24601 ccctggggac atgggggtcc caaacctatg cccttgggtg cctgagacct tgtcagccac
24661 cacagcctcc cctgcagccc tcccaagacg ccctcgggcc ctcaccttgt agaagggtgc
24721 agggttcact gtggcgtggc acctctcgaa caccgtgcct gtcctcagca gcatggagca
24781 gtgggtctct gcacacacct ctggggatga caggcccggg cgtgagtccc ggcccctccc
24841 attccagcta cggctcctcc cagggacccc ccaccctggc agctgggcct gtggtccagt
24901 ccaggggtct gtcagaactg tgggcgctgg gggggcagcc aggggagtgg ggggccggac
24961 actcactgtt gagctggctc atggagcagg ggtcagtctc acgctccaga gcggccggac
25021 agttccccgc ccgccaggag tccacaaaca gcgaggcggt gccctcggcg atacccatgc
25081 tagtggtgaa gtcatccgtt gtgtccccgt tgaagttgcc gcagagccct gagccggcgg
25141 ggcgtgagct cgacttgaac ccatccctcc tgcacccatt cctgcccaca cgccctggag
25201 ctcccgtcct tcctctaaca gcaccctggg tgagaggctg cgtgggccac acgtgcctgt
25261 taccccctggg ggctccccgg acacagaggg ttgtgtgaac ctctcttgga cctttctcct
25321 cccctggtca tggccagacc ctgtagggat gaggcaacag ggccacctgg agaggcaggg
25381 actcacctct ggtctgacct ctgaactggg gcccaacagt gacataggcc tggaagatgg
25441 ggcgcagctg gaccacgagc tccagcccga agctggtggc catctggagg tgggtggacg
25501 tctgcctgaa gaccgtgatg ttgcctgcag gacgcagtgc tcagtgggcc gtctgggctc
25561 cctccccacc cactgcagcc cgccccgaag gcacaactct gggggccac agactgggcc
25621 aggacgtacg agtcttgtat ggcagccact tggcttctcc gttgttggtg accacctcgt
25681 cctgagagat cacaatttg tcctggagag agggtggcct gagtcagggt gcaggcacca
25741 gggagcggag ccctgctggc agggatgggc gcaggaaagg ccttacctgc ctggagaggt
25801 agaccacagc caccagggag gtctcggagt gtgagacgcc ggacttgtcg tacacagcca
25861 tgagggcacc gtcctcggga agctgggggc tctgcgaggg ggcggggctc agacacgggt
25921 ggggtcaccg ggagcgcccc tccccacggg ccacagggct cgtcctacct ggaggaggat
25981 gtaggtgcag gtgccgtgga agcggtaggg cctggcgtca aatgtggtaa caaaggagcc
26041 accttccagg gagcagtgtc cggggcacgg ccgctccgtg cacacccagc ggcccagggt
26101 gcaccggctg tgggtgggcg tgggggtagc ggcatggtgg gcagggccgc tggagccaga
26161 cagcacaccc ctgcctgccc taggccagtg gaacccctgc cggccggcca gagccccctc
26221 cggcgcctca ctcaccaggt ttggcaggca gctattgtga cctccccggg ggcatacatg
26281 gcgccgtgga gcacacaggg gcactgggtg acgggcacgc aggtgtggtt attggagagg
26341 tcattcagga ccgtacctgc aggagagggt cttcttgggg cttgggtggg actgactgcc
26401 tcccactctc ccctccctgg ctccagggag acgccccctc cagcctggct ccagggagac
26461 gccccctcca gcctggctcc agggagacgc ccctccagcc tggctccagg gagacgcccc
26521 ctctagcctg ggccatgacc tctctccagc tgcacttgca ttcgcaactc tatgtcaggc
26581 cctgggcgtg cacagcccct cgtgccaatg tgcccggccc aggttcctgg tgagtggtgg
26641 cctccagcag gcagggacgt gtaactgtcc cttgggcccg gctcaaaccc tgccttccct
26701 cggcagctcc tagaccgcag tgagcagccc tcaccgcccg tgggtttgca ggggtggccg
26761 tggctggggg acctgcaggc tgtaagcgtc caggcgggaa aagcctgtcc cagggcaaga
26821 ggcgccagag ctgggactca ggcagcagaa tgttggggtg acctgggtcg ggtgggtgg
26881 ggtctgacgt cccttgtct ctgggttctc tctaggggtc ccggggagag agagtgcctg
26941 cgactgccct caccttccgg gcagaagcac ccgaaggtgc aggagctgga gcagctgtgc
27001 tgcgggttgg agcaggtctt cacgcaggcc gagccgcact cctggtacac ctggttggcc
27061 gggcactgac ccacggctgt gggcacacgc ggctccggtg agagggtccc acccccccca
27121 cccctccctg ccccacccca gcttgatgga ggacttagcc cagcccttcc tccatctaga
27181 agcagcagcg aggatctcct gtctctcaga ccaggaggga tgcaggcgtc acgtcaggca
27241 gtccaggggc tgggagagct gggtctggag ccctcggcct tcctgcccct ccctctccct
27301 tccctggact cacagcacag gccggggctc cgccagcggc ggaccggctg gcccaccatg
27361 ctgcactggc gggagtactc cgacagggtg gcacaactgc tgttctgtgg gcctggctgg
27421 ggggctgcgg ccacgtccgc ctggcagctt agcacgaagg gctccttgga cacgctgcac
27481 tcaggggcca ccagggtcag cagctgggtg cagatccggg cctgggggg ccactcaggg
27541 tcatgggggc aaaggccaca ccccatgcca ccacgactgg ggaggtcggg cagggcgtct
27601 gtgatgcggc tgcttgtggg ggcccttggt ggtctcggcg accctgtcag acctggggtc
27661 agccccacct ggagccccct tgcttacgtg ctgggcctgc cggacgtggg tgctggggat

27721 gtcctggaag gtgcagatct cgccggggtc gtccagcttc tggagggcag caaacttgtg
27781 gggttccagg aacttgcctg gggtgcagaa tgggggtcag caccgtgggg gctgggcctc
27841 agaggccccc ctgccctgcc ccccacctag aggcccccca gaggcccccc agccctgccc
27901 ccacctaccc tcctcactga caaactcgtt ggtcaccttc ccgtcaaagt tcccgcagag
27961 cccgcacatc tgacccatgt acttccgctc caccagaacc tgcgggagac ggctctgctg
28021 ggggcccggg ggccaggggc cccctcatct gctgtggagg gctctcagtt cctgctcctg
28081 gacccagagc ccccaccatc cccccacctg ctcatctgcc tcttcttatg ggaggctaat
28141 tttccagtgg agaagccgag tcacccacaa aacccagtcc tggctcatgt tgctggtcag
28201 gggtcagcac tgcttggcac gaagggcctg gctgcatggc agcctgaggg ctggctggga
28261 cccccaagga gggcagcccc tcccaagtcc cacctgcccc cccgtgctgc gggtctccag
28321 gcccaccctg gcccttctct cctcaccatg aggtggctgt caggacccca cacgacttcc
28381 agctccagct ccagctgctt ggccaccagc cgcacgctct ggccgaaggg tgtgatctgg
28441 agtccattgc tggtataggg caggctgatg accctgtggg gcaagggaag tcggtggtcg
28501 atcctcagtc ctccggcccc cgagccccccg ggccccggcc cacctgacct acccgatgtc
28561 cttgactgag atgatggctt cgctcacagt gacgacggag gcccccagct ccacgatgat
28621 ccgcgagatg ctcccgtctg ggcctcgccg cagctggaca ctgaaggtgg ggaaggcgtc
28681 cttgcaggtg gccgcgaaga tgtagttgca cgtccccgag aagtcgtaca cgtggtggtc
28741 gaaggtggag aagtgaccag ccccccacgt ggagcactgg cctttgtccg gggctacaga
28801 gagagcagtg ctcacacagc cctgtgtccc caccatcctg gccaggcagg gctggggcag
28861 gcagagaggt cactcgtctc cctgggccag ggtttttgag ttggggccag gctgtggaaa
28921 ccccagacat ccgatgaacc tgagtgctat ggtatatgcc tggccaggag tcagcaccgc
28981 tgtgggcatg tgcacacacg tgtgtgtggg tacacgtatg tgtgttgtgt gtgtgcacgt
29041 gtgtgcacgt atgtgcgtgt ctcgggtgtg tgcatacgtg tcaggtgtgt ccatgtgtgc
29101 attgtgggtg tgtgtgtgtt ggagggctgt gtaggcgtgt gagatataca cctgcatgtg
29161 tgttgggtgt gtgtgattgt gttgcatgca catgtgtgtt tgtgtgagct gggcatgtgt
29221 gtcgagtgca tgtgtgtgca ttcggggtgt gtatgtgtgt gttgggtgcg tgtctcaggt
29281 gtgtagggtg tgtacatagg gtgcatgtgt gtgtagggtg tgtgtgtgtt gggtgcgtgc
29341 acatgtgtaa gtcgtgcgtg ttgtaagtgt gcatgtttgc atgtctggga tgtgtgtgca
29401 tgggtgttgg gtgtgtgtgc atgtgtttgt caggtgtgtt tgtgtgtagg ttgtgtgtgt
29461 tgggtgtgtg tgcatgtgtg ttgggttgtg tgtctgtgta gagtgttggg tgtgtgtgtg
29521 acgtgtgctc atgcatgtgt catgtacatg tgtatgcgtg tgtcaggtgt gtgtgcatgt
29581 gtatattggg tatgtgtgtg tgttgggtgt atgtgcatgt gtgttcatgt tggtgcatat
29641 atgtgtgttg gatgtgtgtg tcttgggggt gacctgggct ccgggcccct ctctcctgca
29701 caccgggcct gggtggtctg ggcggcagga tcagtggccg ctgtgttctt acctgtctgt
29761 ggagagtcct tcagcctctg gaggcctggg ctggtgtagg aggtgttagc cagacctgtg
29821 tggacgggac ccgcagtcgg tgtggggcta ccccgtcgtc cctgagggcg ccgctcacct
29881 ctgctcaggg ctgctccgcc cgtttccctg cacacactcg gcgtgcgaga agtgtccatg
29941 gcccgtgggg ctcgaggcct caacagcaag ccaagcgctt ggcctcccat gctgtccttc
30001 acgagcccca gcttccttcc ctgctctcgt tcactccctc gtttgtccac tcagtcccag
30061 ttcagccgtc agcccctcgg ggttcggcag atggcgggca ccctgtgtgc agggtactca
30121 tccctggggc ttctgggtgg ggctaggagg ggctggtgcg ggtggcaggg gcttggcggc
30181 ggagccaaca aagtgggctg tgcccgcctc cccagcttgg ccgcctggtt tccctgaggg
30241 gtctgcgcca aggccccaca gccggttctc cctgctctcg gtggctccgg ggctctaaac
30301 atggccgctt tcctgcacgt cagccttgag acagccttga tgtcaggcct ggcactgagg
30361 ccactgttgt cagagaaaca tccagatagc ccagtcacgg tgactccagg gcagggcagc
30421 gggggacgtc ccaccctgac tcccagaggc tggggtgggg ccaacacccc ccacgtccca
30481 ccccctgtac ccagagggag acttttccca cctctgggta actccccgga ccctgcctcc
30541 gagactatga cccttcctgt ggctcccacg agcccccgat gtctgagtgg tggtgcggca
30601 cctgagcagg acccgtgacc tctccaggcc cttcttgggg ccgggaccct ccttgcccct
30661 gcccattggt tagcagggca ctcacagcac ctccttccag atgtagctca gagatcccat
30721 tcctgccact cctcacctgt gctctcaccc caggctctgc ccactttctg ggacttactt
30781 gatgctgccc ccattaccgc tggacacctg tgtctccggt gacgcagggg caggggcctg
30841 tcagctggga gatggtgtta accgcggtcc agggaggagc caggctgcct gccccttggc
30901 cgcctgggtg agtcctgtct ggcacaggca ggaggttggg tatttgagtg actggattta
30961 agaagaatgc caaccttggc tatgtgggac acatatttgg ctgtggtccc ctttggcctt
31021 gagatcgttt tctgatgggt tgcacctggc ttatccccag tgtaagctga gtttactcca
31081 gactggtgtg gggtggctc atgctgggcc ctgagcctgg cgagcaccta cccccctcact
31141 catccccctc ctgggggtcc cagtaggtgt catggtcctc agccccccgc cattccttcc

31201 agggatgatc cacagggctc agagcacctg ctgcaggagt gcgcagcaca ccgaaccctc
31261 agccatggaa ctcagtggtt cctgggatgg ggccctcgct ggcctctggt gggggagggg
31321 tgggagagct ctgagcggga ggagcagttg gctgcctgcc cctgggccag cacctctgga
31381 ggctgcagag ctgtgaccgg tctcccctgg gcaatgcccc ctgcaactca gtttctccac
31441 ctttccgatg gggggtggca cttgggggcc gtccccgctg tagcacagac acctcgggcc
31501 tgtgtttcgg tgccctcagc cctgccaggc tggcttcact gccccgggtg ggtgcctcat
31561 tgctgcaccc cctgcgcccc ccgccgccct cgccctgccc cacccgcagg aggttccccg
31621 agtccactgg ctccaagtcg gctgcctcca gctctgaagt ccagcgggag actcctgcgt
31681 tcctgcccag actggctgtg cttcccctcg gctgatccct tggccaggcg catgggcatc
31741 tgggcgggtt ggtccgccac ccccggcctc tcagcccctg gacgagcacg aggcggcagg
31801 tgcgcactgt tccttctgca cgcttggcgg ccgcccaccc ctgcctcccg gcccaccgcg
31861 gcccaggcct gcagcccgtc tgtccgaccc cacactctgc tggctgaggc ccctcccatg
31921 ttggcagaga tggcgctcag agattatttg ctggggtgac gggtggacac ccccacttgg
31981 ccctccaccc tctgcactgc ccacagccca gacctccagc ctctctgggc ctcaccacac
32041 ctccctccct ggctccaggg tggtggcccg agggactagg ggtcgcactc tgctgcaagt
32101 ttagccccac ttcctggcac ttgtccccaa gctgtgccag gtgtgcaggt aggggctggg
32161 gtcccaagca gagggcaccg ctgggagggc tcagagaccc ccacatctcc cacggttgaa
32221 gggtggtgcc cggcgtggta gggagcgggc tcagagaccc ccatatctcc tacagttgaa
32281 gggtggtgcc cggcgtggtg gggagcgagc tcagagaccc ccacatctcc cacggttgaa
32341 gggtggtgcc cggcgtggtg gggaaggggc ttcaaaggag agaaggggtg gggtgggctt
32401 gggtcgccct acagtgggac acccctgggg gggtcccact gtgctctgag cccgaggcgc
32461 ccaggttggc agcagctccg agggcgagct ctgtcacgcc agcgtccacc ccagggggct
32521 cccggctgtg gaggagggtc gggccagctt ggagaggaag gaggagcacg gaacacagag
32581 gcccttgggc tggtgccttg gtcggcaaga ggggtctccc caggtctcag gctgcggcca
32641 cagagggacc ccttggggct ggctccttct gggggcctgg ggagggactg atggtcacct
32701 gcagcagccg cagggcaggc ggttggcaga ggatccagga tgtcagtccc tcctggatcc
32761 tgcccgagct gggtagggga gcgcggggga gagggcaggc tgcctgcggc ggttctgagc
32821 ccaggatggc cgtacacctt ccccctctt cccccacgg cggccacacc aggggaggca
32881 gcgtccccca cttctgtaga ctaggaaacg aggcccaggg cccagcagag actgtgcctc
32941 tgtcccccac actccagaga cctctcagga ctcaggaccc cactcgggga gggagagcgc
33001 cagggctggg caccagcaca cctgcccact gttcccgcgg gagtgccgga ccccactcca
33061 gctgagcgtc ctccccgct ggctgctgtg ccagctcaca ggagggcgtc cctgcccagg
33121 ccccatgggg tccctgaccc caattggcag agcccagcac cgcggctgct gggggaccc
33181 gggcctggca gcgggggctg gtcacggagc cgagctgggg cctcccgtcc atcagcgtcc
33241 atgtgggcca gccgagttgt cgaggcgaga aggcccagag acccccgcac acagggcccc
33301 tctgagggca ccgcagtgtc tggcgcccct cgacctcact caccagcgct gagcagggct
33361 ccgcagcagg acagcagcag ccaccgctgg accatggtgc acagtggaga ggagctcgcg
33421 ctgggcccgg caggcctgct gctgccatcc atgcggctcc aacggccggt cctgggtgcc
33481 ttatataggc tggcgggccc tcccccgccg cacctgcctg cgccccgcgg tccagcccct
33541 taatcaccac tgccggcggg cgccgcggtg gccaaacagg atctgggcct gctttatcag
33601 gactcggctt tctttggaaa atcctgcagg cagcggcccc attatcaccc attcccagcg
33661 gggggctgac gcacgcacgc ccccaaaggt cctgcaaaca ccccctgcat gggggccatc
33721 ctggcccggg ccctccccgc tgctgggtca acgtggcact gtcagccaca cgcctggtgg
33781 ccgggatgga cctgttgggg gaggtcgggg gccctggggc acctgctctg ccccttccgg
33841 ggagcccagt gctgacccag cttgggggga accctgctgc acctgaatcc caggaccccc
33901 tgaggggcac tgggcccacc cctcaccctg aatcccagca ccccctgaag ggccctgggc
33961 ccacccctga gttccctgcc caacaggaaa gatcacccag cgggagggac cttgccaggg
34021 ggtgttgggg agcaggggct ccgccgtctg ggacaggcag cggccttggg gcttagacag
34081 gctgccctgg aggcctgagg agctaccgtg tctctgcagc ccgccaggtc ccaggcttgc
34141 tcctgaagac ccctccacag ctgttttctt ccccctccga gcgctgttgg cagccatctt
34201 gaggacagga actcgggggc tgctcgtctg ctggggcatc tctgcggggc tgtggggtcg
34261 ccggtatgct cctgacccct tcaggcagag gggcagggac tgggatgacc agggggcctt
34321 gcctgtggag gcacgtgcca cgtctgaggg gccggggaca gggcaaggtc tggtgacatc
34381 ggggtggtgc cggtggctgg cggggggggag ggaggtggag gtctgagtgg tcaccaggat
34441 tgccccagct tcgtttggca gcccagggac ccctcccaaa ggggatttg gtccccgact
34501 ccgacaggag ccctcgctgg cctcggggct gctcccacca cacgtcagca tcccggcctg
34561 agccaggagg gactccgagg ccgcctggtc tccctgggcc gggctcccca catggggctg
34621 cacccccatc ccacaggtga gctgagcctg ctggctggga cccgggcctg ccacctctgt

34681 cccacctgtg cccacacgcc cgggagctgc cccacctgcc cccggaagag gccccagggc
34741 cacgcgtgcc atacacagta ggtgtcgcgt gtttcactta aatcagcttc agttttgtgg
34801 aacgttgctt gaagttacaa taagttgctt ttgtaggaaa ttgcagtacg tgtgctgccc
34861 cccaccagga gccccacacc aggcattctg ggtgttctac tctcatctgt tcttcttttt
34921 aatttttcgg ttttgtaggg acgaggtctc actttgttgt ccaggctggc cttgaactcc
34981 cggactcaag agatcctcct gcctcagcct cccagagtgc tgagatgaca ggcgtgagcc
35041 accgtgtcca gccttggctg tcccttctta acacgctcag tggtccctgt ggttcaggtg
35101 agaagctgag gcacaggagt gaggcccttg ccctgtcatg ctgagaggtt ggatggacgc
35161 agggctcaca gctggccctc gttctgagct ggggcctctc agcccgggct cgaggtgggg
35221 acgatgaggg gcagagatct tagggcctcc ttccctgggt gggtcactgc accagggcag
35281 ctggtgccaa aagggacctc atgggcaggt gtcatggctg ggagccctgt ggccatgtca
35341 gcccggcggt tgtgcttgtc tgaccacctg tctgggcttg tgagaaccga gctcaggttc
35401 tcaggaggga tcctatgaag ctttgaatgt aggggcaggg gtgggttggg ggtgagagcc
35461 agtggacact ggttccccgg ccaggcctgt agggcttcgg gtcctggcca ctgaggcttc
35521 catttcccag ctgtaaagtg gcagagaccc ctgaccttga aggttatggc tgtggcgttc
35581 agcatgtgag tccccagcgt cctctagaat tgggggatcc ctgcaacttg cacaggtgtg
35641 gggcgggtgg gggctggata ccaccagcct gcaatggggc tgcccactgg gcctgtggtg
35701 aagatgcccg cttgcaggtg tgtggtgatt gtgtatgtgt gtgtgcgcat atgcatatgt
35761 gtgcgtgcac gtgtacctgt gtgtgcgtgt gtatgtgtgt gcctgtgtgc acgtgagtgc
35821 ctgcctgtgt gtgtgcatgt gtgcctgtgt gtgcacgtgt gtgcatgtgt gtgcgtgtgt
35881 gtgcacgtgc ctgtgtgtgc ctatgtgggt gcctgtgcct gtgtgcctgt gagtgcgtgt
35941 gtttgcgtgt gtgtgcatgt gtgtgcacgt gtacctgtgt gtgcctgtgt gtgtgcatgt
36001 gtgtgtatag gttgaatttc tggtgaaagc tgtgcatccc agaatttatt gtccccatga
36061 tctcagggcc actcgagtgt gtagaaggcc ctggccccga gccctggtgg gggctccatc
36121 gtggcaccat ggggcctccg gagcctgggg gtctctctct ctcaatgcac tcgcttggtg
36181 gcaaatggct gggagcggtg tttctgtcct ggccggggag cgcttgaatg cactgagcag
36241 tgggggagat aagcgtgtga ggtgttgttc ctggagcaac ctggtgatag gagccatgtc
36301 ctgtgggcct ggctggagaa cagggcctga ggactggaca agtgggtgat tgtcccctct
36361 catgcccagc gctggccccg ccctctatgt ccgtctaagt ggcctggccc tgaacagcgg
36421 tcactccagg cctggtgtgg ctgtgaagcg ggagtcaggg tgggccccgg gccccagctt
36481 cccggctgaa ccgttagggt gggcgctggc tcctagaccc gcagggctcc ctggacaggt
36541 gtgagcagac cttcacttgg acttgaggcc ttcacccctg caggtgtctc cagaaggctc
36601 tggaactgac tcgggcctgg ctcccctcac aggaggctga ggccctgggt ctgctgggac
36661 ttgtgttcac agaggcgctg ggatggacct gctggtggcc ccctgcccct cggccagccc
36721 cttccctgcc cctcggccag ccccttccct gccccttggc cagccccttc cctgcccctg
36781 ccccgctggg ccctgtggtc aggtgtgacg tcccctggag gagaacaggt ggggcttcac
36841 cccctgaggc ttggcagctg gtccccatca gatcaaagga ttgaaaactg tgggtttggg
36901 gagtgtttgg gaaagatttc aaccccggga gtgggaatgc acccgggctt ggatcgctgc
36961 cattccccat ccgaggcgcg ggcccagtgc tggccggcag agctcaatat ggccagccag
37021 tgcccaggtg catgcccgag gggcgcgtc acggcttcct gttgatccgg atgcccgatg
37081 tcttgggagg cactgaccct gtgccaggtc ctggggccca ttgcttgggg aggcggtggg
37141 aacctggaga ccctggggac agagtggctg gatgcaaggt cccccaaggg gcagggggat
37201 ggcagggaag gggcgtcagg cctgggggag gtcacctgcc ccaccctccc tccaaggccc
37261 ttgtgtggct gggtggggct ggccctgggt cctctccctc tcccccgtcc ctgagcgtca
37321 ctggtgacct ccccatggac ttgtccagct tgccagtacc ctgcggtttt gcctcctgag
37381 tatgttgaaa cgttcctagt ccagctcttg cctgcagcca cccccatcga atctggcctg
37441 ccgtggcccc cgccctgcag ccccgagtgt gtggtacccc ctcattctcc ctagcacctc
37501 agaggaggcc ctgcctggct ctgacactcc ctgacctctg gcctctatcc ccctccctgg
37561 acaactcctg cttctccacc agccctgcag acccctctga ccccagctgc tggggtccac
37621 cctgccggag gtagaaacag agccagctgc atgccacagg tgagggccct ccccaaggcc
37681 tggggagggg cagcgtgacc ccggaagggc agctgcaagg gccgggcagt gcagctcacc
37741 cgggcgacca ccagcccacc ctcaatgaga ccccacagcc cctgtccagt gatgttcaga
37801 gggctagggc tccccgggca cccactggtc tgcactttca gacttgactg cttggtgcct
37861 gggctgggat ttgggggcac tgcccttccc ccgtatccct gggacccccct ttgggagccg
37921 gacactcagg tgcgcacagc ccccaccccc agcccagccc cagccctgtg ctgtccgctg
37981 cccacagccc actgtgtggc cttgagtgga ctgaaggtct cgtctggctg cgggaggggc
38041 cctggtagct gctgggcctg gaggtggctg ggatggtggg ctggcccgcc tgtccggggt
38101 gggtagagga gtagctagca ggaaccactg cctgaggctg ggctcagccc aggctggtag

38161 cctcatgttc cagaaagggc tggggagctc tccacctgcc ctcctccaga gaggggccct
38221 tgtccttttt gtctacttat gccccgtgca ggaagcaggt cagggtaggc tggggccctc
38281 aggagcgggc acgtttcagc ctggggtggg ggcaggatac gggctgccgg ggccattgag
38341 gcagcagcac aggaggggtc ctggccaggc cactcctccc tccaggtggg agcttagctg
38401 gggcgggacc cccacctccc cgggtgagca ttgccggccg gtagccttgg gcaggcaccc
38461 aggagaggcc cctggcctga actcctctca cacctgactc tggtacctct gagccccagg
38521 gtaggtggct gagtcatctc cccagctggc accccaaata ccttctcccc ttttcccatc
38581 ccatgggagg tggctgtgtg ccacaggtga gggtgcagag cccccagctg ctccctgccc
38641 tgagagtttc gccccctggc atggggccct gggcagaccc tctcaggaca gagccttcct
38701 tgcaggccag ccctgccagg ccctcacagt gggggtagct gtgccccacc agccccgagg
38761 ggaagaccag gatcctgagg gctggggttc acctccgatg tcctgggggа gatgtccatg
38821 cgttgccaac tccaggcctg aacccaaggc gccctcatct gagcacaggg agaggctgcc
38881 gggacggctg ggtgtctggc tgactgcccc gcccgggctc tggagctgat aaccagcgga
38941 tgcggagtga aatgcaaaca gataaggctg ttgggaacta ggccccctgg actgctccgg
39001 gcgggcagcc gtggggcacc acactgccca gggccagggg ttattgggcc gccttttgcc
39061 ctcagtgagc gcagctggat ggggagttag caagggcttg ctctccgagg gtggctggtg
39121 agggggcttt gaggagggtg gggggaccct gggctgccga ctcagactct ggaggctgct
39181 ggtcaagtgg gggtggaggt caagctggag ctgaggctca tgagaccacc tctctcccag
39241 gaatcctcat ccctctcatg gggtgacat ttaggactcc ctttggggag cccggcaggg
39301 ccccaaaagc acaagtcaaa tggcctctga gggcagctgc agctcacagc aagggggccc
39361 tgggcagccc agggtgagat gaagggcttc cagggctcca ggtgaagagc aggactgggt
39421 ggggggtggg gccaggtcac caggaggtgt cggagaccag gctggaaggt gacctggcct
39481 agagtaagct gggagaggtg ggtaaactga ggaccagagc tgggggtggg gaagggaccc
39541 tgagccagcc tgggggcgg tggtgctgtg tgctggcgtg gagtctgggc aggtgccccg
39601 gcagaggtgc ctctgccagc aatggcgttg gggccacagg tcctgcagag aatttgaaga
39661 atttgttttt ttccccagaa aagctccagg tattgcagcc agacctgagt ctctctcagg
39721 aatcacatcc agggcccatt ccaggcagtt ggacgtgcag tgggggcagc cactgaggcc
39781 caggcaagaa ggggtgacgg ggcaggtcct ggtccagagg gttgtggggg atgctggcat
39841 ggctggcatg gctggcatgg cctgcagaga gtcgaggagc gcccaggctc accacagctg
39901 gttcttcggg tgttgttcag gggaaacaca gtggaggcct gacgggaaca ggtgggtttc
39961 ccggccactc acaccaggtg ccggcccaag ggagccttgg gcctgatctg ctgcccagcg
40021 gccgtgagtg ctgtgtggcc cgtgggaggc cccgtgcata tggcctgcag agtcaggtgt
40081 gggggcccca gaacacgtag gcctggcaga agcccaggtg gcacagacgt ggccctacag
40141 ccgggctcgg ggccccggga agccagaggc tcaggccctc ccagctggca tcagggctga
40201 tcggggctga gaccggtcat cggagcagcc catgtccact gtcccctgtc tcagcagctc
40261 cccgggggtc ccccaaaccc caaccctccc ccagatagga gcacaagcag ggtggccaag
40321 gagcaccccc cagcttaccc acggaccctg gctcaagact ggtggccaag gtgcaccctc
40381 gctgtactag gcagtggcct ccagtcctga tgccaccgac tcttgggggc atggccagtg
40441 cccgaggcgg atgcgtgcgc acacatgctc ccaggaggtg tttgtaacaa ccccaaactg
40501 gaaatgaccc agatgtccat cagagcgcag cctgttcacc tgctcttcca gcttcagcaa
40561 ttacaaacaa agcagttaga acacgtgtgt ccgtgtctca gaggagcatg tgctgccact
40621 tctcccggcg tggactggct ggatcacgtg gttggcgtct gaatttttca gaaacggcca
40681 aactgttttc cggcatggct gtgccattgt gcctcccacc cgtgatgtga gttccagccc
40741 ctcctcaccc tcctgtatgc ttgacgtcgt ccgtttttaa aattttagcc attctaacag
40801 gtgtgcagtg ctatctgact gtggttttca tttgaatttt cctaacgact aatggcgttg
40861 agcatctttc catgcgttta tttgccctct gtgtatcttc tttgatgaag tgtctgttct
40921 aacgttttgt ccatccattt taaaattggg ttgcttcagt tttgagagtt ctttatatat
40981 tctgcattcg agtccttcac taaaaataca tacttggcaa agatttttct cccagtcagg
41041 gcttgtcttt ttattctctt aatagtgtca gaagatacta ttaagtgtca aaacttctgt
41101 tcaaaagagc ggaagttcat tctttccctc cctccctcct ttcttgcttt cttctctgtt
41161 ccttccttcc ttccttcctt ccttccttcc ttccttcctt ccttccttcc ttccttcctt
41221 ccttctttcc ctccctccct ccctcccttc cttccctctc tctctctctc tctcgatgga
41281 gtctcgtgct ctgccgccca ggctggagtg caatggtgca atctcggctc actgcaacct
41341 ctgcctttcc agttctggca attctcctgc ctcagcctcc ggagtagttg ggactacagg
41401 tgagtgccac cccgcccggc tagtttttgt atttttggta gagatggggt ttcaccatga
41461 tggtcaggct ggtttcaagc tcctgacctc aggtgatcca cccacctcgg cctcccaaat
41521 tgctgggatt acaggcgtgc gccacctaga ctggccgaag ttcttaattt tggcaaagcc
41581 caatgtatta atttttactt tacacatcat gcttttagtg ttgtgtctaa gaaatctttg

41641 cctaatccaa ggtcatgatt ttatcattat gaaaggatct tctttattgc cagttccttg
41701 ctctaaagtc tacctttgta ttaatattgc cacttgatct ttctttctgt gttagcacgg
41761 tgtgtttttt cctcttcctt tactttcaat aactaatgtg tgtcttttac ttacagtgag
41821 attctctggg cagcttatag agtgatcttg cttttattc tagtctgaca atccacatct
41881 ttttattggg ggtgtttaga ctattacatt taatgtaatt agtgatgtgg ccaggtgaaa
41941 agctaccatc ttgctacttg ttttctgttc cttccatctg ttctttgtct ttctttttctg
42001 ccttcttttg gattgactat ttttccacag ttcagtttta tctccttgtt tgcttatctg
42061 ccataacttt ttattatttt agtagttgtc ttagagttta tagtatgtct acaacctact
42121 gtagtctcct ttccaaagat attatcacat tccatatata gaagaagaac cttccaggct
42181 gggcacggcg gcttacgcct gtaattccaa cactatggga ggccaaggtg ggaggatcac
42241 ctgaggtcag gagttcgaga ccagcctggc caacgtggtg aaaccccatc tctactaaaa
42301 atacaaaaat tagctgggtg tggtggtggg caccggtaat cccagctact cgggaggccc
42361 aggcaggaga attgcttgaa cccgggagac agaggctgca gtgagacaag atcgtgccac
42421 tgcattccag cctgggtgac agaacaagac tccatctcaa aaaaaaaaaa aaaaaaggtg
42481 cctcacagct cccttctctt gggttgtgca attcttgtca cccattttgt gtccacacac
42541 gattcacacc gcactgcatt gttggtattt ttgtttaatt aattattttt taaagaggct
42601 taaatagtaa aaaaacttct atatttaccc atacaattac tatttcaaat gcattattcc
42661 atcgtgtaga tccatatttc ttttttcttt tctttttttt tttgagatgg agtctcgctc
42721 tgtcacccag gctggagtgc agtggcgtga tcttggctcc ctgcaacctc cacctcccgg
42781 gttcaagtga ttctcctgcc tcagcctccc aagtagctgt gacctcaggc acccaccaca
42841 acgcctggct aatttttgtg tttttagtag agatggggtt ttaccatgtt ggtcaggctg
42901 gtcttgaact cctgacctca agtgatctgc ctgcctctgc ctcccaaagt gctgggatga
42961 caggcctgag ccaccgagcc cggccgtgga gccacatttc tatctgattt ccttttccac
43021 ccgcctgaag ggctttcttt aacatttctt aatggacact gcaatggtta tttgtctgaa
43081 aatatcttcc ttttgccttt gtttttgaaa gatgtttttc taggatgaca gcttcttctt
43141 ccgtttgtac tgtaaaggtg ctgctctagt tttcttactg gcacaagttc ccccgccaaa
43201 aaaatctgtt gttattttaa tatgtttcct ctttgtaaca cgcccccacc ccacaactta
43261 agatttctc ttttacactg attctgtatg attaggattt ggtgtcattt ccttcatgtt
43321 tcttgtactt tgtactcact gagcttcttg tatctctggg ttttagtttt cattaatttt
43381 tttttttttt ttttgaaaca gagtcttgct ctgttgccca ggctggagtg caatggcgca
43441 atctcggctc gccgcaacct caaactctgg ggctcaagca gtccttccgc ctcagcctcc
43501 cgagtagctg ggattacagg cgcctgctac cacacccagc taatttttgt atttttagta
43561 gagatggggt ttcaccatgt tggctgggct ggtttcaagc tcctgacttc aggtgatcca
43621 cctgctttgg cctcccaaat tgctgggatt acaggcgtga gccaccgcac ccagccaagt
43681 tttcctcaga tttgaacaaa ttatggccat gatttcttca atttttcctg tcctcctctc
43741 ccttcgtggg ctccgtttac agacgtgtca ggctgcgcga agctttgcca cctactgctc
43801 gctaaggagc tgtttatttt ttggattctt ttgtctttct gtgtctctat tatgtcttca
43861 agttcaccgc tgtcctcttc tgcattgtcg catccgttgt taatcctgtc cagtgtgttt
43921 catctcacac atagtcattt tcatctttag atgtttaatt tggatccttt tttatatgtt
43981 tcatgttttt tactaaatac gttcaatttt cagcatagca ttttgatgac atggaatact
44041 gtcgtaacgc ctgttttaat gtctttgtcg gctaattcca acatctgtgt tggttttgtg
44101 gtttggatta gttgaatttt ctcctgatta tgggtggaat tatccccttt ctttacctgc
44161 ctggtactct ttgaatggag gttggacttc gtgaatttta cctcgtgggc tgctggatac
44221 ttttgtgtcc tggggctttg ttctgggaca taattaaggg attggagaga gtttgggcct
44281 cctgggcctc gtgtggctcg taggtgggct cagagcagtg ctgagtccag ggcaaactac
44341 gcctcaccat tgaggcaaga ccctgagggg cactctgccc accgcaccgt gaactttgag
44401 tggcgaggtt tctcggtggt gctggcggga gcagtgcttt tcccagcctc tgtgagctcc
44461 gctgctcttc cttccaatcc ttcgggtcgt tgctcgccag ccttagtctc ctcccaggca
44521 cgtgctgagc gggttccctg ccaaacgctc aagacggacc ttctgcaggt ctccaggctt
44581 tgctctgggc agctctctcc ctgccagtgt cctgtgcact ccagctgccg tggtctcccc
44641 ggcctctcat ctgagctctt cctgagttct ccttcctggc cccttgtcct ggaaaccctc
44701 aggcggtgac ctgggcagtt tagggctgtt tcctgtctgc catggactga acgtttgtgt
44761 cttccccacc ctccggttcc tgtgttgaaa tcctggtgcc caaggtgacg atattaggag
44821 gtggggactt ggggaggtgg cagagccctc aagaatgggg ttagtgtcct tacaggagag
44881 atcccagagc cgggcgcggt ggctcacgcc tgtaatccca gcactttggg aggccgaggc
44941 aggtggatca cctgagttca ggagttcgag atgagcctgg ccagcatgga gaaaccctgt
45001 ctctactaaa aatatgaaaa ttagctgggc atggtggtgg gcgcctgtaa tctcggctac
45061 tcgggaggct gaggcggaga atcactcgaa cctgggaagt gagcttgcag tgaaccgaga

45121 ttgtgctact gcactccagc ctgggtgaca gagtgagacc ctgtctcgaa aaaaaaaaaa
45181 aagagacctc agagagctct cccacccctt ctaccagagg cgaaggggac ccagtgggaa
45241 ggtgccgtcc gtgaaccagg gagtggcctc accagacatc gaatctgcca gagtgtggat
45301 cttgaacgtc ccgtcctctg cagccgtgga gatgcgtgtc tgttgctttt atgccgctca
45361 ggccatggcg tttccggata gcagccaggt tggagggacc cgctgtctct ctgggaccac
45421 tctcttcgtc gcctgatgtc caccgtcttg acagatgttg tttcccatat ttcctggagt
45481 tttttagttg ctaaatcctg gcctctgcta ctctctttgc taaaaatgct agtccttgat
45541 gaaatggaat cttgatactt taatacagag tggaacatag tcactaaata aaacctggaa
45601 aaatgtagaa gagtagcagg aagagaacac accccagccc cgcggtccac ccacccgcag
45661 tccctatcgc cacctcctgg ctctggtttc cctgctcgtg ggctgggca gcattgctgc
45721 aactcttgat tcgctttcct ggggccacca taaccaagca gcacaacccg ggagatggct
45781 cagagtctct ctgcgtcctg ggagcctgag atccaggagt gggtgggttg gagaatggtt
45841 ctccctgggt cactctggag gacctcttaa ggtggacttc ttaagggacc cccggctccc
45901 accttgccaa agagcctccg agccacacct gcctcccgca ggcctccagt tggtgcccac
45961 atgcagcccc cgccgtgggg tcaccctggg gtaagggtct ctcctcccct gctaaccttc
46021 tgctgggtct tggcatccct gggatgaacc ctcctggcct ggaccttgcc ggcccacatc
46081 aacccccagc cctctgctcc ttggcctcac ccacagcctg cagaatgtag gtgagtttcc
46141 tgatgataat ataataatag caatgatgat gatgatgatg atgatggcaa taacaatgat
46201 gacagtcaac actgccgcgt gccgggctct ggccctttgc atgtctgggc cggctgtaca
46261 aggcactgct ggggctcccg ggctgggagc cagggacagg caccgtcaca gcagccaagg
46321 ggccccccta ccaccccagc acctgctggc cctgaaccag ctgctccttg agagctttga
46381 cagactcttc cgcttggcag cattttaatc tgctggtgca agagccttgt cgcttcctca
46441 aagcctgtgc ccatggccgt gggcagctgc ctgtccctgt ataggcagag ctgcgtcatg
46501 ccctgtggcc ccaggtctgg ctctggggtt ctggctgggc ggggcctgga accttctggg
46561 agtcaccatt gactggctgc agccaccggc ttcccagcaa ggatgtcctc ttctgtagga
46621 aaatggggac gtcgggagca ttctctggac agcaggagat gcatgtcgac aggctggccc
46681 tgcctcttcc caccccagag tggtgagtgg ggattctggg gacttccctg gcccccatgg
46741 gcctcgcgtg tcctggtgcc catgtgtctc cagtcccagg gtcggccacg gtgtggaccc
46801 ctccactttc ctccaggcct gaggtggggc ccgggcggcc tgtcaaagaa gagcccagcc
46861 cagaccctcc ccagatccct gggggaagga ggccaccagg caccctgcct tcctgctgcc
46921 tgtcagggtg aatctcaggg gaccctggtg caggaggggc tggctctgag ctggacaggc
46981 ctctggggtt gccctggtgg ccttccctct ctctgggtca gacgtgccca ggcagggagg
47041 gtccaggcca tgacagagcc gggccagctg gggacagggc atcccccagt gcaatccttg
47101 tagcgaacgg gtcctagagt gcagctggcg ggacactccc gccatcactg gatttactgc
47161 aggtggagac gggttctgta cccacgtaga tgaggaaaca ggcacagaga gggggcagcc
47221 tgcccaaggt caccggggag ggagtggaga ggctggatgc ccttgggccc agactgcagc
47281 ctcccctccc cgatgtgctg cggccccagt gtcctgggcc ccatgtggcg cctgagccca
47341 caggtgtcac caaaggtctg agttgcgggg acaaaggcgg ggacaggccc gaggtgcggg
47401 gacaagggcg gggatgggcc tgaggtgcag ggacagaggc ggggacaggc aacccctgcg
47461 gttcgagggg taggtggcca cacgtcagtc cctgggaggg ccaggcagga ggtgggccca
47521 gcggggctgg gaaatgaggg gccagtgccc ccacgctcac tgcaggggat gtgagagggc
47581 gagtggtcgc tcagccacac ccacacccgt gtgtacagga cccacgcccg cccgccttgc
47641 tgtcccgccc gtgcacggct cacacagctt ctcccattat tcaggcgctg ccgggccctg
47701 caggactcag atcgtccctg tgccacttgg cagggtcacc ctcacgcctg cccggtggcc
47761 ccacctgccc cacctgccca gcttctctcc cttctcagtg ctgtctgtcc cagagcctcc
47821 catcaatggg gccagagggc tctggcccgg ctgtgggctg actgttctcc ccaagatgcc
47881 tgagagccac ccgggcaccg gacttgccaa catgcaggca cacgcaggca cacacctggg
47941 ccacacggcc ctgagcacac gccttgccca gccctcctcc gggctgtggt gcgggtgcca
48001 ggtgccacct gctggcgggc tacagcatca cggctctcgc cctccggaac cttccatggt
48061 gccacaccag cctctggtct ggctgcagac ggcttgggct gggcagggaa gcttccactg
48121 tcggggaacc tgggaccaca gtctttgctc caagcgccct gggcgggtag aggccaggtc
48181 cggtatgagg agccctggtt ccctgaggta gacagagcca ggcagacgtt gaggagacat
48241 gggggctgta gccctggccc cactgcccga ctctctctct gcatttcggg agctggcaag
48301 atccaagggg gtccccaagg ccctgaggct gcactgagca cccctttcct gctcattttc
48361 agatgcagcg gtgtcctgga cccctgggga gaggtgaccc ccccagcagg aagctgggcc
48421 tggtttctgt ccctctgcag ccacaaggcc tggctaggat gctgggagca ccacaccctg
48481 gagactcagc tcaccaagga ctcaggggcg ggggctcccc tggcacctgg gaagctgggc
48541 ccccggcccc ctggactccc acccagacac catctcaacc caggtctgtc tgctctcccc

48601 gtcactgccc tgggttctcc actcctggac ccgaccctgg tcctggggca gtgtcccagc
48661 tgctgtgtcc caggcccccc atccactgcc cttggcccac cttctatgtg gcccattaga
48721 ggggtgtggg ggctgcaacc tgcttctgct gaacccctag gcctgccagg gggcatcagg
48781 attctgcaca gagaggcagg ggacccggcc agacatgcag gtacccacgt acctaattct
48841 gggccttggg gcaaggactg cgtatttatc ctggcccagg caagtctggg ggtgtctgag
48901 gggtggtgag acgtctgggc agattgtccc tgccctgctc tgctctgccc caccctggcc
48961 agccctcctg gcttctcaag gtgctcctgg ccacgatggc acagtccccc aggcccaaaa
49021 tgtgccccca tgctgcccga atgccagtgc gggggctggt agcaaccaag gccatgccct
49081 cccctccccc tcctggggca cctgccccca cgctgtggcc atggctccct ggctccacca
49141 gcttcttgtg accccctgagg ctccccagct cctggccccc catggagagt tccctcccca
49201 taaggggggg ccagacaccc ccctgtgccc actccttggg agactggcat tagatcctgg
49261 ctgtgggaca ggctgggcca ctctgggggt gagaccaggc ttctcccacc tgggggaccc
49321 atcctggctc cggacttggg cgggtgacag cctgggtttc ctgtgtcctc accgcccgct
49381 tatcagcaag atggggatgt ggatgacagg agtgctggcc acggagggcc tggtgtgggt
49441 ggccgtcggg acgagggtag tgtgggcggc cacggcagtt ggtgctactg ctgggagggg
49501 ccgaaggtga ggccctcctg ggctgtgggt ccctgtaagc cactgccccc gtggggtgtg
49561 cagtcaccaa gccgacctgc tgccggggtt ccgccccctc cctttccctt ctgcttcccc
49621 ggcacctgtg tcccaagcct gaagccctgt ccgtgtcaga ggtggcacct ccctgggcac
49681 gggcacttgg acgctggtgc ctcccctgct gcggacccct ttgctgtggt cagggcctcc
49741 tccctgcact ggccccgcct gtgaccgcag gcctggcggg gccctcaatg gccacagagg
49801 gcacaggccg gggccaggag ggcaccgtct cctggggcac agatatgctg gagtgagcta
49861 tggaattgtc catcagcagc ggcaagcttt gctgagcgcc agctgtatgc agagcaaagg
49921 cgggccagcc gggaagggcc agtgaggacc tggagtcccc agcatcccca ggagcctgca
49981 cccctacgcg ggggacgcct gggcagctcc tccctcctcc actccccgcc ctctcctccc
50041 ctccttcctg cagttcccac agctgtttgg gtgcctcccg tggcgcctgg ttgagctgca
50101 gggggagatg tgtgtggaca ctgcaaccct cccgaagctg ggcctggaag aaatgacttc
50161 cccagaacct tatctgggcc ggagaggggc gttggcagcg ggggtcttgc cgccttccgg
50221 tcctctgcat gccaggcacc acctggggcc gggccagggc aggctgcctg gacaccatgg
50281 acctgcccag ctgtagggga ggtgtgtcct gcagccctac ccgaggccca ggcctgcgtg
50341 gctgagagga ctgaggacgg ctgacccccct tggtgactgc ctgggcccag aggggagttg
50401 ggggagtggt cagctgggtg tggccagccc tgggggaagg atccagggac tgtgtccact
50461 tagggatagg aggcagctag cagagccctc ccagctgacc aggggaggcc ctgtgggcac
50521 aggaggggcc ccaggtgtag gtacaggtgc agggctgtgc ggctctgtgt cacccaggtg
50581 gagcgtcttg ccccgtttga tggctgacaa agtgcccttg aatgcgtcag acccagcgtg
50641 gtcccagggt cctgacccta acataccacc ccaaattacc ctcaccccag cctacccctg
50701 ccctaaattc accccaagcc tccacccaaa ccccttagtc ccaacacctt aaccctagac
50761 ccaaccctac accccaagcc ctaatcccta accgctagcc tcaccctaac cttcctaccg
50821 gatgccacct ggcaaacatc ctcctggccc ctatctgccc ctcccccggg gatccgggag
50881 gcagtggggt ccctgggagg ctgctccctg cagaacgcga tggacagatg catgggcacc
50941 gggccccagc accctctggc ttccttcatc ctccccaggc acttccccccg ggccagaggg
51001 cagcctggca gccctggact ccgggagggc cgagtctggg gagctggacg acgtcacatt
51061 ccactcttga tgatgccgcc ccagtcctac aatctggaca gcaggagaag ctgcccattt
51121 cctccatctc cggtaatgag aagcaaatgc tgatcctggc cgtctcctgg ctcttgggtc
51181 cctaggttcc tgctgcccct gccagttcct ggaagatgcc agcaggaggg agggcagtgg
51241 agctggactt gcgcccacaa caggatctgg ccataaacgc agtcagggcg aggctgcggg
51301 gtgggtggct gagcctcctg gtggactatc tcccctccca ccccccgccc tggcccgcag
51361 ctgttggctc ttcctgggaa gccacagcct ttgtttgttc cttgaaggag ctggtgcgtt
51421 gcctgcgagc tcctctgcag gcttgggggt cgcacctgct gtccaccccc ctcgaaacca
51481 gctccatcca gcagcaggct gcaagcctgg agaggccacc ggggaccagg cagggaaact
51541 gaggtcccaa aaaggcaggg aacaggtgca gggggcctgt ctgacctgcg ctgggactgg
51601 gccaggtgag tctcctccag ggcccagccc catcttggac agaggaagtt gatagtcagg
51661 aaaggcagga gtggcctctt ctaacaattt cagctctgaa ggggccaagg gtggcgatgc
51721 tgacagcatt tcctccagct cccacctgcc tgctccgggg aactccctac ccaggcattg
51781 atattcacct ggtgatacta ataattgtct caggtgataa attgtctcag ctgctctccc
51841 acatggccca caggtccctg gaggttcccc ggatcctttc agggaagacc atgggcccta
51901 aacgatcttc ataacaaaac tgagaagtgg cctggcttct tcactgcgtg cctgtgtgct
51961 gggatctagc gtggcccgac tgggcagctc ctttccttcc ctgacgctgc tccaggggaa
52021 agaaggacca gggtcaatcc ggggtgcccc aggtggatca gcgaatgccc ctgaagccag

52081 gtgccagttg catccccgtc agatgccccc accccagtct cctgagcctc attagtcacc
52141 cctcattttc ctctggggac cccaggcccc agctcctgtc tggttagagg ctgaaggggt
52201 ctgagcgaca cctaccctag gggacctccg gaggggtctt ggtgtcctct gacttctgtc
52261 ctcagaaggg agctcagaat gcagctgagg cttcccctgg ggcccagggt gtctgtgtcc
52321 gagccacagc cagagtgtgc cctggagagt cgctgggatg cacggtgggc tctcgtcatc
52381 ctgtccccac tccaggctca ggctggagcc gtgatggcac cgctgcactc caacctgggc
52441 aacagagtga gaccctgtct ctataaaata aaaacaaaaa taaagaagag catgtggttt
52501 ctgcagtttc ctggccaaaa atgcataact ccaatctcaa tcataagaac acatccaacg
52561 tccccaacaa ggggaggttc tacacacacc tgcccagagc ccctcaaaag tgtcaaggtg
52621 tgaaagacat ggaggcatgt agaagacggg agagccagtg tggcctgtgg tatgttcatg
52681 acggtcacag gtcagcgccc acggtcactc agcagccatg ccaggcaggc cccaggggcc
52741 caagaggaag gtgggatgtg gcgggatggg ggctctgcac ggccctgcag cacaagtgcc
52801 ctgccgactc caggggcagc aatctggtcc cctcatggtg ccattcccca gggacgggtc
52861 ctcgcctgcc tcgccggccc tccacagaca ggacagggac aggtcgtcag ctgtcacgga
52921 ggccctcgca gttttctgaa tgtggacttg ctgttcctgc ccatggcttc gtccacggac
52981 tcgacagcac cccactgtcc atcacacccc caccgatgct gcttcccaca gcaggactca
53041 tcccatgctc aggcagtgct cccaggagca gcgggttaat agagcgtggg acggcccctg
53101 aagaccagtt atggcgccgg ccaggaggcc ctctgaaagg acgggcgcaa ccccgcagaa
53161 cagaccaggt gctcgccatc catgtccggt gccgcttctc tgtggcgatg gtatccagga
53221 atcacgggtg ggagtcacgg ccacagtccc tgtcgcacca gccgcctgcc cacacagcca
53281 ccgttcactc tagcttgaag gtcccgcttc ctcagggagg agcactccct ccagggtcga
53341 ggtgctggcc gtgtggagtt cgacgtcggg actgtcccca ggggcctctc ttcccactga
53401 atcgacagag agaatgtgag cttgctgtcc ggcgggcggg gccggggatc ctgtccgtcg
53461 aggggacacc ggattgctgc ggagagagga ctagacccag agtccagcca agaccagaac
53521 tgagaccagg cgaccagcag agcctgcaca ataataatgc ttcgttttgc aaatgtattt
53581 tgttctttta ttgtatatat ttatcatgtc caatgtgatg ttttgaaacg tgtttacatt
53641 gtgcttaaat caagcagcat aacacacatg acttcacata cttcccattt ttttgtggta
53701 agaacactca aattctactc gcaatgattt tctttctttt tttctctctc ttaatgattt
53761 tcaaggatgc aatatggtgt tattaactgt agtcaccatg ctgtgcagta catctcttga
53821 atttattcct tttgtctaac ggaacttttt ttttcttttt gagatggagt ttgctctgtc
53881 gccaggctgg agtgcagtgg cacgatctcg gcttactgta acctctatct cccaggttca
53941 agcaactctc atgcctcagc ctctggagta gctgggatta caggcacccg ccaccacgcc
54001 cagctaattt ttttgtttgt ttgttttttg aaccggagtc tcactctgtt gccaggctgg
54061 agtgccgtgg tgccatcttg gctcactgca acctccgcct cctgggttca agcgattctc
54121 ctgcctcagc cttctgagta gctgggatta caggcgcctg ccaccatgcc tagctaattt
54181 ttgtattttt agtagagatg gggtttcacc atgttggcca ggatgatctt gatctcctga
54241 cctcatgatc tgaccgcctc agcatcccaa agtgctggga ttacaggcat gagccaccgc
54301 gcctggccta atttttgtat tttcagtaga gacgggggtc tcatcatgtt ggccaggctg
54361 gtctcgaact cctaacctca ggtgatctgc ctgcctctgc ctcccaaagt gctgggatta
54421 caggcatgag ccactgtgca cggtctgtaa cagaaacttt gtactcttgg accaacccct
54481 ccccagttcc cagcccctat gtccctgggt cctctctctg cttctgtgag ttcaactttt
54541 ctaggctcca cttatgtgtg agatcatgca gtgtctgtct ttctgggcct ggcttccttc
54601 attcagcatg aagtcctgca gttcatccat gtcggcacca atgacacaat tccctcttct
54661 gtggaggaac agttctctga cgtggatgca ccttccttgg ctgctggacg ctgaggctgg
54721 ttccatgtct ggccgtagtg gacagcgctg cggcgaacat ggggtgcggg tgtctcttct
54781 gcgtactgat gtggcctccg cgtgtcacgc ccgggagtgg gacagctgga ttacgtggtg
54841 gttctgtttt cagtttttttg ggaatctcca tgctgttttc cataacggcc atcatcacag
54901 acttcttatc atgaaatcat tttagactcg tagaatcctg gtaaaaatag tatagagaat
54961 ggtgatctgt catctctggg cagggcaaag ggagctgggg cgaccaggca ggagggaggt
55021 ttgctttcat gatctgttct ttacctattc aaaaacaatt aaaataaagg ccaaggcaga
55081 tggatcactt gaggccagga gtttgagacc agcctggcca gcgtggtgaa accccatgtc
55141 tactaaaaat acaaaaatta gccgggtgtg gtggggcaca cctgcaatcc cagctactcg
55201 cgaggctgag gcaggagaat tgcttgaacc tgggaggcgg aggttgcagt gagccgagat
55261 cgcgccactg cactccatcc agcctgggtg aaagagtgag actctattgc aaaaaaaaaa
55321 aagaaaagaa aaagaaaaag aaaagaaaaa ccagaaaaaa cacataaaat gcgccattgc
55381 actctagcct gtgtgacaga gtgagactcc atttcaaaaa aaaaaaaagg ctgggcacgg
55441 tggctcatgc ctgtaattcc agcactttgg gaggctgagg cgggtggatc atgaggtcag
55501 gagatcgaga ccatcctggc taacacggtg aaaccctgtc tctactaaaa atacaaaaac

55561 ttagccgggc gtggtggcgg atgcctgtag tcccagctac tcaggaggct gaggcaggag
55621 aatggtatga acccatgagg cggagcttgc agtgagccga gattgcgcca ctgcagtcca
55681 gcttgggtcg cagagacaaa caaacaaaca aacaaaaaaa cccagaaaaa aaccacaaaa
55741 aaaccccccat taaaataatt taaaatttta aaataatgtt tagtaaaata atgtttaata
55801 aaaaataatg tttaatgttt aatgtgagaa atgcttatat ggtaaaaaat aaggaaaaag
55861 gataatttaa aaaagagtga gaagaaaata gagtcagata tttatctgat ctttagagaa
55921 gagcactttt ctaagcatac aaacaaagga agaaataata aagaaaaaaa gtgacaaatg
55981 tgttacctcc ctaaaaacgc gtgcgtaaga acaagggttt gatcctcact gacggggtcc
56041 caggtgctga ctcgagtcca gcaagcccca gagccagagg ctgagtgcaa accacggagg
56101 tgacctccat ggcaagagcg gtgtggcgtg tccgtcccgc acagacagaa atgcggtcca
56161 ggggtgtggg aggaccatga aggggtcacg ctgaacgtgc tgcaggtgcc caccaagcag
56221 acagtctgcc tttcgaggtg tggttcaggg agttagcagg gcgctaaccg ctggctgctt
56281 cgttgggtgc aacatagaca gaaaaggggt cacattaaac tggaaaggcc agacctggct
56341 tggtttactg tggaggaagg gatgcaaagg cctagggaga ttggaaggct agggtaggtt
56401 tgtcacttaa gacctcctca cccaccagaa ggtccagaag acagaccttt ccccacactg
56461 ggaactagat tagtgagtgg ggtagaaata gggacagatc cctgggctgc tcttctctga
56521 gggccacaga cctcacagtg ggagccacag ccactcagct ggaaaagagt cgcaatggga
56581 agaatcgacc cgggacagcc gagcggtggc tttcagccgt cagaggcgag ggggacctgg
56641 gactgccagg aggtggctct cagccgtcag aggcgagggg gacctgggac tgccaggagg
56701 tggctctcag ccgtcagagg caagggggat gcgggcgcca tcatgagtag cagaggcaga
56761 acgacagtca agctgggctg accccttagc cacagctctc ctaggaggaa aacagaaagc
56821 ctgggaagat ggtactggat ctgcgggagg ccaaggtcgg ggtgtcacct gagaccagga
56881 gttcaagacc agtctgggca atatcgtgag aaccccatct ctaaaaacta aaaaattagc
56941 tgggcttggt ggtgcacacc tgtggtccca gctactcagg aggctgaggc aggaggatgg
57001 cttaagccca ggtgttcaag gctacagtga gccaagattg catcactgca ctcctgcctg
57061 ggcaacagag cgagaccctg cctcaaaatc aatcaatcaa tcaataatca tgttctcagc
57121 aaatatttaa cttccccgat atattgtggg ggaaaagagg ctgtaaaaca atctgtggta
57181 ataaccagga gaacttagga gcacccgtgg gtgcccatgc gggcagagat tcacgaacac
57241 tgttcacccc cataaccagc aagggtgctg ctatccagaa cccatcgcag atgagcaact
57301 gtgcctgccc aggccaggcc acggcaccca gcagccggga cccctcccca cctgaaacga
57361 tactgccacc tggggacccc aggccgctga catggggacg cgctagagct ttcgaacacg
57421 tctctgcatc tggacatgcc ctcggtgcac gtgtattgct ttaagttcag agacgtcaaa
57481 tgtcatttaa tgaaaagact ccggctacat aattgtcccc aggaatgccc aggacaatgt
57541 caggaatctc ccagacctgc acagaaggtg ggaaagattt tgaaggatta agaggcttct
57601 cgggggaggg ggagggggac atttttcttc cacccacgga cagttttgtg ttttctgatt
57661 tctgtaacga gtgccacagc tctgtatgga aaacagcact gcctttggcg tccctcccaa
57721 ttcctggcac atgagtcaag ggctcccata gcgtcaccgt tgagggcagg gcagccccgg
57781 gctcacaaag taaagaagga aagggccagg cctcactgca gggtcacccc gaggccataa
57841 aatcctgcac cttatgcctt gggcaccatt taatacaatt aaacggtgca ataaaggaag
57901 ggagtaataa agaaagcaaa tgtcagccag cgaggacatc cggcacaggc cacaccccag
57961 gtccagcccc actagggtgg agtttgaggt ccctgggaac cccagagtct atgcactgct
58021 gtgcccagct aattttttaa catttctgca gagacaaggt ctccctagat tgcccaggct
58081 ggtctcgaac tcctggcctc aagcggtttt cccaatgtgg cctctccaaa tgctgggatt
58141 ataggtgtga gccagtgggc ccggccacgt gctcttatgt agcttatcag agtgtatgac
58201 gctgcctgtc ccattgctgg ggatgttaac cttgaaccct tggttcaggt ggcgtctgcc
58261 aggtctctgc actgcaaagt tactattttg cgactttgta attaataaat atcttaggga
58321 agatactttg aattcatgta aataacttgt ttctcttcta acctcagcct attgacttta
58381 gcatacctct gtagacagct gttaatcctg tggcctctga tggtgatttt ctatttactt
58441 catttcttct gcattaagta actggaattc tattaaaata agctgtttct tcttatttgt
58501 ttgtgcagtt atttacttca ctatgggttt gtagatactt attttattat ttgggttata
58561 atccaacatt atctttattt tgtttctcaa attgttccag tgttggccat ggcagcccct
58621 tcacagtggc tcctgtgtgc tttcacccta tctgtttttg agcccttcct cactttctgg
58681 caccataaaa tgctccaggt ttatcttgta atttccttcc ccagagaccc agctacttct
58741 ctaaggagtc ctgattcttt taattggaaa atggtattta gaaaccaaga tctggacatt
58801 gggtgtggta gttactactg gggtgtcatt tttttttggt agaccttctc aggggagaat
58861 actagaaagc atctgtatat accctaatcc atacacacac acattcacac acacacacac
58921 acacacacac acgtgcgcac acacatttct acctatctct tctatctctc tatccgtcta
58981 tcatccatga aacacattgt tgtaagccct tggtttttgg gtgctttgtt atgcagcaat

59041 agctgactga tacatgcggc tgggattgtg cacactgtat tttcaggctc tttgaccaga
59101 tgtcttcctg gaaggcactg gtgggaggtg aggagaggag atttctgttc cttcaccagc
59161 ttctttcagg gccctccagc catagagggg ggtggctact ccagcccacc acttcttttg
59221 gcactcccaa tactggctct gccactccat cctttctctg tgctcctggg ttctggcagc
59281 ccaacctctt tcatttgttc ccccagccct gggagtggaa gctgcttcct gctgttcaca
59341 acgtcaggtg cctcggtgcc cgtcttctct tccagcctcc agcacctgtg taaccagttc
59401 tctgtattaa cttccttctg tttgaaataa cgagtgtggc ttccttttcc tgactgactg
59461 cacagtgatg gatgtcaagt ggactttcaa ggtgagtctg aggtacagaa gctggagatg
59521 cactagcggg tggatgagga gacagaccct caggagggag gggaacgtgc ggtttagcca
59581 aagccgcatg tagggaaaat ccaccacgtt ccactcctac atgggaaaag aaggaaagcc
59641 tcaaatcaat ggtctctcta ccgtaagaaa ttaggaaaga aagagaagag caaattaagc
59701 ccaacgtgag caggaaaagc aaaatcatca ataaaacaac aaaatgaaac actagagaaa
59761 agtcaacaaa accaaaagct ggttctttga gaagatcaat tagacggata aacccctagc
59821 caggctggag aataaaagag aaaacagaca aattaccaat gttaggaaca agacaggtga
59881 cgccactgtg gattctacac atattacaaa gataataggg ggacatcatg aactacttca
59941 agtcgaaaca tttggcagcc cggatgaaaa ggacaaactc cttggacaca ggatgaatcg
60001 aagatcactt gggcagaaat aacttgaatg gccctatatg ttaaaggaat tgaattgtaa
60061 ataaaacaca acaaagcctt cctgcaaaga aaaccccagg cccagatggt ttcactggtg
60121 aattctacca aagatttaag aaagaagtat gtcaactctg cacaaaccct tccaacaata
60181 actgaaaaga gaggaacacc ttcaactctt cctctgaggc cagtattacc ccgatttcaa
60241 aaccacataa aaacattata agaagactat gaacctttcc tttgaaagca gtctttagat
60301 acaagcttta tctgacacat taacatgtta gactgctttc aaatgaaaga ctgtgtatca
60361 aatgaaagaa ggggcttatg aaggtgttgg aaatgagatc atcgggaaaa gagtgtggga
60421 aggggctttc ctatccctct tatttacaac ccaacttaat aaacacacgg tatactattt
60481 cctgctattc tttgtcctat gtttcctggt tattttgata atgtacttta ccactgtggc
60541 cagttcttct cctgactctt tcacataaat aaagtgtcca tatttctgga aaaaaaaaaa
60601 agaaggctat gaaccaacat ccctcataaa cacagatgta aaaattctaa acaaattaaa
60661 aaaatatttt actttaagtc ctgggatgca tgtgtagaac gtgcaggttt gttacatagg
60721 taaacgtgtg tcatggtggt ttgctgcacc tgtcaaccca tcacctaggt attaagcccc
60781 acatgcatta cctatttgtc ctgatgctct ccctcccctc acctcccacc ccccaccagg
60841 ccccagtgtg tgttgttccc ctccctgtat ccacgtgttc tcattctaaa caagattttt
60901 tcaaatcgaa tcaaaaaatt tattaaaact ataaccacag gggatttatt ccaagaatcc
60961 agagttggtc taacattcaa aatcaatgta attcatcatg tgaacagact aaagatgacc
61021 aatcatagga tcatctcagt caattcagaa gaacgcactt gaccagattc aacacccact
61081 cctgataaga actctcctaa aaatagaata taacttcctc atcctcataa tggcatttat
61141 aaaaagcctg cagatgacat catacttaaa cactaatgct ttcccctgaa atcaggaaca
61201 agaccaagat acctgctctc accacttcta atgaacatgg tgctggaaag cacagccagg
61261 caatcgggaa ggtgaataca cttaaaagat ctagattgga agggggagtg aaactgtgac
61321 agatgatgtg atcatctgtg cataatcgag tatctataaa aggcagcggc agttgtccct
61381 tgatatccac ggggcatggg tgccagcacc cctaggacac aaaatgtatg cgtgcgctaa
61441 tcctctacat aaaatggcat agtctttgca tataatctac atacatcttc ccatgtactt
61501 tatttattta tttatttttt taagacagaa cctctctcta tcacccaagc tggagtgcag
61561 tggtgtgatc taggctcact gcaacctctg cctcctgggt tcaagcaatt ctcctgcctc
61621 agcctcccga gtagctggga ctacaggcaa ctgccaccac ccctggctaa tttttgtatt
61681 tttagtagag acagggtttc accatgttgg ctagactggt ctcgaactcc tgacctcagg
61741 tgatccaccc gcctcggcct cccaaagtgc tgggattaca ggcatgagcc accacgcctg
61801 accacatcct cccgtatact ttaaatcatc tttatatcat gttaaaattt ctattagggg
61861 ccagggtcag tggcttacgc ctgtaatccc agcactttgg gaggccgagg caggtggatc
61921 atttgaggtc gggagtttga gaccagcctg gccaacatag caaacccctg tctttattaa
61981 aaatacaaaa aaaatttagc caggcatggt ggtgtgtgcc tgtaatccca gctactcttg
62041 tggctgaggc agggtgcggt ggctcatgcc tgtaatccca gcactttggg aggctgaggt
62101 gggcagatca cctgaggtca ggagttcaag accagcctgg tcaacacagc aaaaccctgt
62161 ctctactaaa aatacaaaaa gtagctgggt gtggtggtgg gcgcctgtaa tcccagctac
62221 ttgggaggct gaggcaggag aatcacttga acctgggagg tggaggttgc agtgagccaa
62281 gattgtacca ttgcactcca gcctgggtga taaagcaaga ctccatctca aaaaaaaaaa
62341 aaaattaaaa gcaggtcttg aaaagatatt tgcacactca tgttcacagc agttgaagcc
62401 acccaagtgc cccttgacag atgaagggat aaacagaatg tggtctgtcc ttacagtgga
62461 atattattct gccttgaaaa ggaaggaagg aaattctgac acaggctaca atacatagat

62521 gacatgaggc cgagtgaaat aagccagaca caaaaagacc aacacggtat cattccactt
62581 atttgaggta tctcaagttg tcagattcat agcaacagaa agcagaatgg tggctacctg
62641 aggctggggg agagcggagg gtgtgtttag tgggagcaga gtttcagttt aagaaaaatg
62701 aaggtgttct ggagctgaat gctggtgatg gctgcacagc atggtgacat agtcaatacc
62761 actgagccac gcacttaaga ctgcgtaaga tgagaaattc tgttatgtgt atctttccat
62821 aataaaaagt cccaaaaaat caattgtatc tctctatatt agcaataaat aattggaaat
62881 gggaacaaac aatatcatta taacggtaca aaaatatgaa atacttaggg aaaaatctga
62941 caaaggatgt gaaagacgag tacgctgaaa actgtaaacc attgggaggg aaattagaga
63001 cctgcagaaa caggtatgta caccttgtcc atgggctgga agactccata ttgctaggac
63061 gtaagttctt tctaaactga taaatatatt taatgcaatt tcagtcaaaa tccctgcagg
63121 cttttttttt tttggtagaa actgacaaat tgagactaaa ttcagaggga aatgcagagg
63181 acatagaaga gccaacgcag tcctgaaaaa ggggcaaagt tggaggaaac tgaaacattg
63241 gctgggattg agaactgtta caaagctaca ataatcaaaa cagtgcagca ttagcataat
63301 gacagacaaa taggtcaatg aacagagtaa agtccagaaa caaattcatg tgtatataaa
63361 caataattat tttttcttta aaaaaatttt tttttgagac agggtctgac tctgtcaccc
63421 aggctggagt gcagtggcat gatctcagct cactgcaacc tctacaccct gggctcaagt
63481 gatcctccca tctcagcctc cccagtagcc atataggcac atgccacccc acctggctaa
63541 ttgaattttc aacaaaggag cagaagtact gcaatgaaga aagaatagtc ttgtcaacaa
63601 atggggctag aacaattgga tatccataca taataagaca aacttcaagc catacctcat
63661 actacatata aaaaattaac tcaaaatgca tcatggaaaa ccccaaacta taaaatttct
63721 aaaagaaaac ataggggaaa atatttgtgt gatcttgggt taggcaaaga tttcctacat
63781 gtaataccaa aggcaaaatt tataaaaaag taaattgatc aattggattt tatcaaaatt
63841 ttgaaactcc tactttaaaa aacactattg agagaatgaa aagacaagcc acagactggg
63901 agaaatatat ctacaaagga tctctccaat aaaggactcg tatctagaat acataaataa
63961 tgtttagatc ttaaaaagaa acccacccca ataacccaat aaataatggg caaaagattt
64021 gagtaggcgt ttcactaaag aagacatatg ggcttataa gcctgggagt aaactgactt
64081 tttttttgt aagaaattag atatcctaag ttaaacagtc acacaaggat gtgaggagaa
64141 agtgctttga caggaattgc tatactagtc acaagtacat taccaaagat ttctctataa
64201 tggatttaat atttaaacac agcacccagt atttgttgaa aaagcaaaac tatataatag
64261 gggtttttaa aataattttt tttttgagac agtctcactc tgttgcccag gcttgagtgc
64321 agcagcacga tcttggctca ctgcaacgtc tgcctcccgg gttccactga ttctcctgcc
64381 tcagcctccc aagtagctgg gattacaggt gcgccaccac gcctggctaa tttttgtatt
64441 tttagtaggg atggggtttc atcatgttgg ccaggttcat ctcaaactcc tgacctcagg
64501 tgatccgcct gccttggcct cccaaagtgc tgggattaca ggtataagcc actgtgcctg
64561 gtctaaaata aagtttttta aaaagtgaaa aaaagaagac atatggatgg cagataagtg
64621 tataaaaaaa ttcttaacat cattagtcat tagtgaaata caatttcaag taataatgag
64681 acaacatcac acacttactg gagcggctaa aattaaaaag actgaccatc ccaagtgttg
64741 acaaggctgt ggggtaacgg atgtctcata cactgccggt gggaatgtag aaaggtacaa
64801 ccactttgga aagcagttta gcgatttctc aaaaagttga acatagacct acctgtctga
64861 tccagatact ccactcctag gtatttacct ggcagaaaag aaagtgtatg tccacactaa
64921 gattacacaa atgttcgaag cagttttgtt cgtagtggcc tcaaatggac aatgagccca
64981 gtgtccatta gcagggaatg aatgagccca ggaaggaatg agctatttac acagcaatgc
65041 tggatgcaat ctcaacataa ttatgcttag tgaaagagac cgaagaatgc atactgtata
65101 gttccattta cacagaatcc tagaaaatgc aaacaaatct gcaaagtcct agaaaatgca
65161 aacaaatgcc atggaaaaga aaccctgggt agctcccgag tggggaaggg tgggagggag
65221 agattccaaa gaggtgtaag gagccctgtg tgggtgatgg gtggggacat attcatcctg
65281 attggggtgg tggtttccca agttcaacca atttatgtca aaactgaccc aatcatacac
65341 gtaaacatgt gcggtttatt gtatgtcaat tctaactcag ttaagctgtt ttagcctgtg
65401 tcaacgcact ttcctgacca gtaaatccta agtgttttcc ttgttgccac ataaaatcct
65461 agtcataatt tatagtggat gcatgttatt ctagagttgt ttggctctta gagatttaaa
65521 attaatcaga gaaatatatt agagcccaaa ctatagtgac agtaaaaaat gaccagtgct
65581 tgccagggat ttgtggggaa ggatggaata gctgagacac agggcatgtg ttagggtggt
65641 acaaccattt tgcatgctac tatcctggtg gatatttgac actctacatt tgcaaaaccc
65701 agaaaactta atagtgcaaa gagtgaacct tgatgtacac aaacataaaa atattcagga
65761 ggttgaggga tgccacagat gaaatgcgga atgtggcaaa acaatccaac tgtatttcaa
65821 atgcatgaaa taaactcaca gaagggggcg ggggtggagg gatgttgaag ggccctgacc
65881 tttgggcact tcggcaagga gcaaggggc actgagattc ctgccctggg gctacgacac
65941 cacccaggaa cccctgatct ttctgttact tatgactggg gtttctccaa aaagcaacag

66001 tctcctttgc aaaaagtggc cacaccatta tcaagcggtg gacagtgtaa gtttggggct
66061 gggaagattc tctgggacca cattgcagct ctgccattct tgggctgggt gagccgaagg
66121 agtgctttca ccctctctga gtctcagttt ccttgtctat aggatggagt gaggtgagtg
66181 ccttcctccc agggttgagg gctccagtga ggtgggccta tagagcagtt ggcctgtact
66241 tgctcatcat gagctcttgt gaggtggagc cctgtggagc tgccacagaa ggtgaactcc
66301 agtaccaggg agatttcatt tgtccatcct tttacccacc cacccaccga ttaatccatc
66361 atccacccac ccacccaccc atccacccac ccactcatcc atctatctgt ccatttgtcc
66421 atccatccat ccatccatcc atccatccat ccatccactc atccactcat ccatctctcc
66481 attcatccat ccacctatcc agtcactcac tcacatatcc atctatccat ccatcctttc
66541 atccatccat tctttgaccc atccacacat ccatccctcc attcatccat gcctccatcc
66601 atccacctat ccattcacta acccatctat ccatccatcc atccatccat ctatccatcc
66661 atccttccat tcacccattc atccactcat ccatccatcc atccatccac tcacccaccc
66721 attcatctat ccatccatcc acccacccat tcatccatcc ctccctccca tccatccatc
66781 cctgacccat tcatccatcc cttccatcca tccatccatc catccatcca tccactcacc
66841 catccaccca tccatccatc ccatccatct acccacccac tgacccattc atccatcctt
66901 ccctccctcc ctcccatcca tccatccttt cattcaccca ttcatccatc catccaccca
66961 cccacccatt catccatcca tctaacgatc tgtccctccc tccctccatt tatccactca
67021 ttcacccagc caaccaccca ttcatccatc cgtccactca tttacccact catttattga
67081 tctattcatc catccatcca tccatccatc catccatcca tccatcccat ccatctaccc
67141 actgacccat tcatttcccc tccctgcttc ccatccgtcc atctatccat ccatccatcc
67201 atccacctgc tgacccattc attccccctc ccttcctccc atccatccat ccatccatcc
67261 atccacccac ccacccaccc acccacccac ccacccaccc agaagcaaag ttattgagtg
67321 catctgctgt gagggtggcc atggggggcc cacatgggct gctgcccttg cagcattcgg
67381 agcaggatgg ccagcaaggg tcacacagtg tggccagggt ggtgagaaca gtgttcagtg
67441 tgaggatgtg acacctggac caagtcttga ggggcaagtg ccccagggtg tctggactcc
67501 tgaagcacag ctatccacct gatggccctg gccctgggct gccctttcct caatggagcc
67561 tgcccagtgc ccgcctgccc agcagaggcc tggactggtt gaggcctctc ttgatccctc
67621 acctgagaac aaacgcctct gtgtgctcac ttgctcaccc tccacacctc acagagcagt
67681 ggcaccaaca tcccgccttt cacctctgca gtccttggga ggtgaagtgc acacctccag
67741 gtgtctccga acctttggag ccccagaaag tggacatgct tggggtgtct gagtctgccc
67801 tggagagctg gacgtgactg gtgttttatt tgccctccct tgaatcctca gatgctcacc
67861 tgccagggtg taggtctggg ccttgccact gtgtccctgt gtggggctgg ggttggagct
67921 ggggtcctcc ttggtgtcca gagggctgag aaccacggct tctctgggtg tgctctccat
67981 ctgcccacca aggcagggcc agagctgtgg cgctggggtt gggtgccagc ccccaccacc
68041 ctggttaagc ccagttggct gggggctggg cagcctggtg ctggcagggt tggctgtggc
68101 aggatggctc tggcctgcag aatgggtgtg ggccagcctg gcctcttgtg tcctgagatg
68161 ctggccttga gatggagaca caagatgctc ggtgactcag tttccccttc tgtaatgtga
68221 gctaataatc agaccaacca catagggaca tggaaaaatg gccccagatg acacatgtga
68281 ctgacatgtg ggcagcctgc acttgctgga ggccgacatt tcagtaatca gcaatggggg
68341 ccacgtttgg accaacacag gatgtaggga gacaggggct gccacgtggg gatacctagg
68401 gctgcggtgt gggcgggtgg agtgctctcc aggaaagcag gctctgtgac tggtctcccg
68461 cccccgtggg agaggtattg gccacatggg gagtgcttgc gggggtgggt ggtgatactc
68521 cagggaagca atcagtgggg catgggccat tacttcaggc agcctagggt gggcctcagg
68581 ctggagaccc ccggaggcca tcaggaggca gcacgtcctg gaggcagcac gtcctgctcc
68641 ttggaggacc aaggtctttt ctttcaaggc cctgcactga ttgtgtcaga cccacctgct
68701 tcgccagggt cacatttaag aagtcccttc acagcagcat ccagcccggc gccatgccaa
68761 agactggagc catggccaga caggccgact cacaaagcca ccgttcgagc gaccgggacg
68821 tgtgcagggg acgcacctgc tgtctttggc tcccccagca gcataggggt tctctcttct
68881 cgccccctcc ccagcctcct cttccccttc cccttcctcc tcctcctttc cctctccctc
68941 ttcctccggt ggctctgacc cccacatgcc cctcacccgg gcttcctggt gagtcaggtt
69001 tgtggataca cagtaggggg gccgtcactt caggtgccat gtgacgtcag tgctgcctcc
69061 tccctgcagt cactactgca aattccatgc agccgttggc aacagtgtct tgtcacggta
69121 aacatgttgg gcgtgggcag ggtggggaga ggctgggggg gccaggatgc tgtctgtgtg
69181 ggaagctgta ctccaggatg ctgtctgtgt gggaggctgt actccaggat gctgtctgtg
69241 tgggaggctg tactccatgc agcctcgttc tgagtcccag ccggggcctg aggctggggc
69301 agcctgacat ggggctgggg atgtgggctc ctggctcaac tgtgtccggc ccgccgttgg
69361 gtgggtggtc attcccacca ctgccatcat gttcttgtca cagaaagcag aaccgacaag
69421 cattttcctc ggagtggtca aagcccccac tctctccctc caccagggtc cctcgaaccc

69481 catgggaaag ccacagagtg gcgatggggt cccagggcaa tacggctgcc cactctccca
69541 ctctgcactt gctggaggcc gacatttcag taaccagcaa tgggggccac atttggacca
69601 acacaggacg tggggagaga ggggatgcag tgctgtggaa ctgcctggcc cccatcgcct
69661 ccaccaccac agtagatgct gcgaatctgg cagtggggac cggctgaggc ttggaggcaa
69721 agaggctggg gacactctcg ctttgggggt gccagtgact ccatagtcgc cccggcctga
69781 tggagccctc actgtcctct tccccagaac agctcccttt ccccgttagg aatggggttc
69841 agctctctgc accctgcccc atgcagggct gcctgtaacc tcagacaggg ctcctacctg
69901 ggaccttgcc atgggccaag aggggtgttg gggtcagacc cctgagcctg ggaaccacca
69961 ctgcccgtcc agaggggaca cagggtcaga tctaggggac ctacctcagg cttcagggct
70021 ggaggggctg tgctgggcag cttgtgctcc gctaggattc ctccaatccc cccagggcag
70081 ggggagcctg gcctggaaaa tctctgtcct gaggcccgcc tggggcatgt cagggtcagg
70141 ggactgcagt tggggccgtc cttgggtttc cccagggctc agtgccagtg tgcggaggcc
70201 cacaccagga agtgaacaat gatctcctct ggcctcgccc agctggctct ggtttcctaa
70261 tccccggtcc tcctggcagg ggccacacac tgagcttcct ccacgtgccc aggtcctggc
70321 agggagcgca gaccctgggg cctggtgctg gcgggcaccg caggagggcg ggaggggctt
70381 cgtcccaggc cctgggtctg ggcagcaggt cagccaggga aacaggcttg gtgctttggg
70441 ccccgagtct ctataactgt tggggtgagt ccctccccac tgccatcatg ctgccggcat
70501 gtccctggca tgttcaggcc aaagccagga actcaactca gggcccctct ctattttcag
70561 gaggagaaaa ttgtagagag aggggagggc ccccagacct cagtttaccc actggcgaca
70621 caggggtgcc tgcctgtgcc ctcccgggcc ggggcaagca gtggtgggcc cagtggtctc
70681 gtagtctggg gtcggtgtga gttccggttc tccaggcttt tttccagaca actgctggga
70741 ttggtgggcg agaccaaggc tcatcaaagg cacagccttg gggcaggat ccccaccatg
70801 agtcagaggt agttctgggg agcctgggca ggctgtcacc tcctcagctg tcaggcccga
70861 ggtcctcatg tggtccccag gagaaggggc agacggccac ttccggccac cagccagctc
70921 cctgtgtgcc tgattccgta acatgtcccc tggctgggca tgtactcccc aagttctaat
70981 tacatgtaac tgcagagaag ggctcagcct gggaaaagga tgggcatagg gggtggttgg
71041 gggctggggc ctctgacaca gctccatgag cccggccaag agtcccacac aagtcagtgg
71101 cccccccgga ccctgaagga tcccacatcc tccctgccct cggggaggcc cctttctggg
71161 gtcaggcctg gaagctgccc cagagcttgg gccccaggaa tgggttggtc ctcccagcgt
71221 aacgtgagcc tgatcaggcc tggggacctg ctcagcgggt gtctgggggc ccatggcggg
71281 ctaaggagcc tgaccagact tgcttctggc aggacacccc tcccccggcc accctgggct
71341 cgcccctcta gtagctgcat gtgttccccg ggtgtgtgtt ggcattcagg ctacagggct
71401 gcctcatcct gaagaaggct gcgtttaccc agggagccat aaagagatga cctccgataa
71461 cctgaatcaa tatttcccca ttggggctcg ggcccccgca gctgtcttct tgatcatctg
71521 gcagatgcca cacccaccct tggccctccc ctgccttcct gccctcctac cctcctgcca
71581 ggacatataa ggaccagacc cctgccccg ggcgcaaccc acaccgcccc tgccagccac
71641 catggggctg ccactagccc gcctggcggc tgtgtgcctg gccctgtctt tggcaggggg
71701 ctcggagctc cagacaggtg agagagcaga cacaggggtc tggggcctgg cagagtgtcc
71761 tgggggcagg gcgaggcggg cgggcaagtc gcgtctggga ggaggagctg gtcccagagt
71821 gcagcctgcg cggctctgct gaggctcctg gcccgggttg gtccctggaa gcccccggcc
71881 ctgctgactt tcaaggagct ggaaggtcgg ggctcccctg ctattccttt ggggttgact
71941 gcccgacgac agtgtgggtc ttggggccag caccaggtgg aaacagcagg tcaggcccca
72001 gtgaactggg tcattgtcca taggggagga aggggtggcc aggatcccac cagaaggccc
72061 cattctcagg tggcagagac ccttgaagag ttggggcagc acagcccttg ctggggagcg
72121 gggtgcccag aatgccctct cctacatccc gcttggcacc cggccgcact cctcaccagg
72181 ccgggggtag aagccctgag acccctgtgg tggggtgacc aaggcccagc agagggcccg
72241 aggataggaa ggaacctttc ccggccaggg gccctgtgct gggctcgaag ctgcttccag
72301 gtgcttcttc aggggccttc tctcgagggt agcttgggca gccttccccc tccggggcca
72361 ctcacccctc attccccgct gctccctcag agggcagaac ccgaaaccac ggccacaacg
72421 tctgcagcac ctggggcaac ttccactaca agaccttcga cggggacgtc ttccgcttcc
72481 ccggcctctg cgactacaac ttcgcctccg actgccgagg ctcctacaag gaatttgctg
72541 tgcacctgaa gcggggtccg ggccaggctg aggcccccgc cggggtggag tccatcctgc
72601 tgaccatcaa ggatgacacc atctacctca cccgccacct ggctgtgctt aacggggccg
72661 tgtgagtgtg gtcggtggca cccctcccac atcctagcaa cgggggctga tgtttcccaa
72721 agggatattc cttgtagccc tagaagaccc cttccgcccc agcacacagc tcaggagaac
72781 agccttgagg tttgggttca ggtcactaat tcattcaaca aacactgatg agcccccacc
72841 attcccccca taggcaaggg gtttcagtta tccctttgcc tgtgtgtccc tgacagcccc
72901 tcccctcgga gcccaccagg ctccggacag acttggcacc cctggaggct gcatgtctct

72961 ggtcctgtgc atggagtggc cgtgtgtgcc ctccccaggc tagagttaca gaagccggtg
73021 caggggggctg tgggaccccc ttccccatcc ccagctattg ctcccctatt gtctccagaa
73081 caatgaggcc ctgtaagtgc gttcccatcc agcgcctgcc cctcttctgc ctggggattt
73141 agtttcctgc aaggggcccc agcatgggca tgggcaggcg ggtggaggcc ctcaggcatg
73201 ggcatgggca ggcgggtggg tagaggccct caggcgtggg tgcaggcagg tgggtagagg
73261 ccctcaggca tgggcatggg cgggcgggtg ggtagaggcc atcaggtgtg ggcgtgggtg
73321 ggtgggtaga ggccctcagg catgggcgcg ggcgggtggg tgggtagagg ccctcaggcg
73381 tgagtgcggg cgggtgggtg gatagaagcc gtcaggcatg ggtgcaggcg ggtgggtaga
73441 ggtcctcagg tgtgggcatg ggcaggtggg tgggtagagg ccgtcaggtg tgggcgcggg
73501 tgggtgggta gaggccctca ggcatgggtg cgggcgggtg ggtgggtaga ggccctcagg
73561 cgtgggcgcg ggtgggtgga tagaggccgt caggcgtagg tgcgggcggg tgggtagagg
73621 tcctcaggtg tgggcgcagg tgggtgggtg ggtagaggcc ctcaggcatg gcacaggtgg
73681 gtgggtagag gccctcaggc atgggcgcag gcgggtgggt gggtaggggc cctcaggcat
73741 gggtgttggc aggtgggtgg gtagaggctt tcaggcatgg gcaggcaggt agaggccctt
73801 gaggaccgag gcacagaggc tggggtgagt gcctctacct ggaccagcaa ggggcactgg
73861 caggaggtgg ggtagggccc ctgacagtct caggggcagc ctgggggggct ctgggggggtt
73921 tgggacccca tggggggatg ttccaccaag cagggggcct ggaagggggc tgggcagcct
73981 ggtcctccct cctctcccaa cctggtgccc tcagggcctc tgagggggga ccctgcccag
74041 gaccgtgccc cgaggaggga gtggagagga ggggcgtgca ggcaggaggt ggctctgccg
74101 gggaagcccg gccagcggag atggacaggt gctctttggc cactgcctat gtccctccac
74161 cccagaggcc ggccaagttg gtgaccccag ggcaggagct gggcctggca gagccatctc
74221 caccacccca ggcgcccagc ttcagtcccc tctgggcggc ggggtcccgg gaggacaagc
74281 tggggcgggg gggcctgggt ggtggaccca agagtgaccc cgatgtgcct ccgccagggt
74341 cagcaccccg cactacagcc ccgggctgct cattgagaag agcgatgcct acaccaaagt
74401 ctactcccgc gccggcctca ccctcatgtg gaaccgggag gatgcactca tggtgctcag
74461 gggtccccgg actcgtgggg ctggtggggg ctccgtcagg cctctgggca gaccccaagg
74521 gagggcaggg agggcagtgc tctgacccct caccgagagg gcatgggtgg ggcagggcct
74581 cggcagcgcg gggcgtcggt gctggacttg gggggcagca gcagaagccg acctggccct
74641 gaccccccca ggcctcagcc ttcccccaaa cgcactcggc ttctcaggga cctgccctgc
74701 caggccgctc cctggctgct gaccccagcc ttcctgcccc accttcctct ggctcaaaca
74761 agccacgagt cttgggggtt cctggcggct gtgggccggg cgggaggcca gctcacctgc
74821 tccctcccgc aacagctgga gctggacact aagttccgga accacacctg tggcctctgc
74881 ggggactaca acggcctgca gagctattca gaattcctct ctgacggtga ggcccggagg
74941 gcttggaggg ggcagggtag gctacgggcc cccaggagcc ctagctgaag ggccgtgcat
75001 ccccaggcgt gctcttcagt cccctggagt ttgggaacat gcagaagatc aaccagcccg
75061 atgtggtgtg tgaggatccc gaggaggagg tggcccccgc atcctgctcc gagcacgtga
75121 gtcccctcgg tctggggtgg gggtcctggc ggagctggcc tctgaatagc atgctcaccc
75181 tgcgtctgtc cccagcgcgc cgagtgtgag aggctgctga ccgccgaggc cttcgcggac
75241 tgtcaggacc tggtgccgct ggagccgtat ctgcgcgcct gccagcagga ccgctgccgg
75301 tgcccgggcg gtgacacctg cgtctgcagc accgtggccg agttctcccg ccagtgctcc
75361 cacgccggcg gccggcccgg gaactggagg accgccacgc tctgccgtaa gccccggcgc
75421 cttgtgggca ggggacccca gggagacccc acgctggtgc tttccccaag cccgggtggg
75481 agctgtgtct gcgccgggca ccttgagctg gggggacact caccgcaccg ggcaccttga
75541 gctgggggaa cactcaccgt gccgggcacc gggagctggg gggacactca ccgtgccggg
75601 caccttgagc tgggggggaca ctcaccgtgc tgggcactgg gagctggggg gacactcact
75661 gagggcaccg ggagctgggg ggacactcac cgtgacgggc accgggagct gggggggacac
75721 tcaccacggg caccgggagc tgggggggaca ctcaccacgg gcaccgggag ctgggggggac
75781 actcaccgca ccgggcacct tgagctgggg gaacactcac cgtgccgggc accgggagct
75841 gggggggacac tcaccgtgcc gggcaccttg agctgggggg acactcaccg tgccgggcac
75901 cgggagctgg ggggacactc actgagggca ccgggagctg gggggacact cactgtgacg
75961 ggcaccggga gctgggggga cactcaccac gggcaccggg agctgggggg acactcacca
76021 cgggcaccgg gagctggggg gacactcacc acgggcaccg ggagctgggg ggacactcac
76081 cacgggcacc gggagctggg gggacactca ctgagggcac cgggagctgg ggggacactc
76141 accgcgccgg gcactgggag ctgggggggac actcactgag ggcaccggga gctgggggga
76201 cactcaccgc gccgggcact gggagctggg gggacactca ctgagggcac cgggagctgg
76261 ggggacactc accacgccgg gcaccgggag ctgggggggac actcaccgtg ggctgagagc
76321 ccttctcggt gcacttcggg gtggagcggc tgctgtgccc cagcctcacc ctcactgcgt
76381 ggcctctgcg gttccagcca agacctgccc cgggaacctg gtgtacctgg agagcggctc

76441 gccctgcatg gacacctgct cacacctgga ggtgagcagc ctgtgcgagg agcaccgcat
76501 ggacggctgt ttctgcccag aaggtgcgtg tggaggatgg ccccgccccg gcactgccca
76561 ccagatgaga ggcagccctg gcctggggtt ctcgcctgcg ctgaggggac ggctccgctg
76621 ggtggtgggg gcagcggcgg cacagaagtg cctctccctc cacccgatac cgggggagaa
76681 ggggcctcgg tgtgaggccc ttcccaaagg gtggcttcag ggaggccggg aagggggctg
76741 ccttcctggt tatcaccctg gggacagacc tcctcctgcc cggcccctgg cctggtgcct
76801 gaggcctttg ggagcagctc gattgtcagg ggcaggaagg tggcctggag gctggacccc
76861 catggccaga ccccaaccca gggaccaggt ggggaccgca ggcgtcagca caggggacca
76921 gtggtgcctg cgggtgggag gcctggctgg cagcccctcg gtggggattc tggctctttc
76981 tgagccagcc ggggtgacat cgcctccctg gctgtcccag gcaccgtata tgacgacatc
77041 ggggacagtg gctgcgttcc tgtgagccag tgccactgca ggctgcacgg acacctgtac
77101 acaccgggcc aggagatcac caatgactgc gagcagtggt gagtcccggg gccagggctg
77161 ggcacagcag aggctggggc ggctgagccc tgaccctgtg ccccgctgcc caacagtgtc
77221 tgtaacgctg gccgctgggt gtgcaaagac ctgccctgcc ccggcacctg tgccctggaa
77281 ggcggctccc acatcaccac cttcgatggg aagacgtaca ccttccacgg ggactgctac
77341 tatgtcctgg ccaaggtagg ctgcccaggg tctggggcat ggggcagagc tggggctggc
77401 atccaggccc ttggctgtcc cggggtgggt gggctggctg tccctgaagc agagggtgcc
77461 tgtgggctgt cctggggcag gtgaccatgc ttctgctctc tggctggaga ataagaagca
77521 ggccttcctt tctaagccac tgccgggtcc tagggtgcag ggtgctgccc gtcccggccc
77581 tcagcagctg cactgcctct tgccccatca cagggtgacc acaacgattc ctacgctctc
77641 ctgggcgagc tggcccccctg tggctccaca gacaagcaga cctgcctgaa gacggtggtg
77701 ctgctggctg acaagaagaa gaatgtgagt ggtcctgccc cctccttctg gagccccagg
77761 tcccccgagg ggggcccttc tcagccctga gcaacctcgg ccttccctgc aggtggtggt
77821 cttcaagtcc gatggcagtg tactgctcaa cgagctgcag gtgaacctgc cccacgtgac
77881 cggtgagttg tgccccaggg aggggcccgg gcccttcgag ctccactggg cctgcagtga
77941 ttcggacagt ccagccacct cggacccagg aggctgggtg ggaaggttcc acggggggag
78001 ggtccctgcg gcacccagca ggctccgtcc tgggtcctct gctggagggg gtggtgggag
78061 ggtgacaccc tcccgctgct cacctgggcc aggcaggtcc cgggagcccc gcccctcgcc
78121 atgcccctta ctgtgtccct catcgtgccc ctgcccacag cgagcttctc tgtcttccgc
78181 ccgtcttcct accacatcat ggtgagcatg gccattggcg tccggctgca ggtgcagctg
78241 gccccagtca tgcaactctt tgtgacactg gaccaggcct cccaggggca ggtgcagggt
78301 aagtggcccc accggggttg ccccaacaaa ggcccacagg ggggcctgct agccccagac
78361 tcttcccaac cctgtcctgg cccctcaggc ctctgcggga acttcaacgg cctggaaggt
78421 gacgacttca agacggccag cgggctggtg gaggccacgg gggccggctt tgccaacacc
78481 tggaaggcac agtcaagctg ccatgacaag ctggactggt tggacgatcc ctgctccctg
78541 aacatcgaga gcggtgaggc tcggcaacac gggcgccccc acctagcgtg cctagggtac
78601 ccggcccatg gcctggaagg gcagacgggg ctcccagcag gaagcatggg tggtgagggg
78661 cagaagtgag gtggctctcc tccaggggca gcccggcccc tgctgcttcc tgctgtggct
78721 agtttatggc ggccatggtg gcagcctgcc aggtgacctg gaagagggcc tgggctggtc
78781 cctacctgcc ccgtcatgtc caggatgctg ggcccttggg ggtgagagac gggaggtggt
78841 gggtgccctg caggggtttc tatctagcca ggagctgcct ggaaatttga ctcacgggga
78901 ggaaggggcc tgggcatcgg tgcacagagg gaaccatatc tggggcctag gcagccaggc
78961 agcagggccc aggggatctc acgggggtcc cgggccccgc tgaagttccg atcccccact
79021 ccccagccaa ctacgccgag cactggtgct ccctcctgaa gaagacagag acccccttg
79081 gcaggtgcca ctcggctgtg gaccctgctg agtattacaa ggtgggtggg acccacaccc
79141 ccaggccccc atgccatcga ggtggactca gggcaccccc agccccccat gccacccgtg
79201 aggtggactc agagcacccg gttgggccca ctggttgctg tgtgtgcgtg tgagcttgca
79261 tctgtgagcg ccgggccaca ctctgcctcc ctgcctcact gcccgtccac cttgctctgt
79321 cgcccagagg tgcaaatatg acacgtgtaa ctgtcagaac aatgaggact gcctgtgcgc
79381 cgccctgtcc tcctacgcgc gcgcctgcac cgccaagggc gtcatgctgt ggggctggcg
79441 ggagcatgtc tgcagtgagt gccgtccccg tgggctgcat cctggggatg gggtccgggc
79501 tttgagctcc tgggacgggg ctgggggccc tgagcacggg tggtccaggg agaggggtcg
79561 gccccctgca gccacggacc aggctccagc ttcgtcagcc ggtggtagca ggaaaccagc
79621 aactcctata gcaaggggcg gccacgtagc aggggcagaa cctggggtgg gcctggagct
79681 gtggcggccg agtgtgggag tgggtcccag agtgtgcact ccctggcccc ctggccaccc
79741 tggggatggg agctgggcgt ctggctcttc ccgtccctca caccaccccg tggtcctctg
79801 cagacaagga tgtgggctcc tgccccaact cgcaggtctt cctgtacaac ctgaccacct
79861 gccagcagac ctgccgctcc ctctccgagg ccgacagcca ctgtctcgag ggctttgcgc

79921 ctgtggacgg ctgcggctgc cctgaccaca ccttcctgga cgagaagggc cgctgcgtac
79981 ccctggccaa gtgctcctgt taccaccgcg gtctctacct ggaggcgggg gatgtggtcg
80041 tcaggcagga agaacgatgg tgggtacctg ctcgggggtc aggtgtggcg tgggggcggg
80101 ggagctcctt ctgaacctgc cccaagcgga gacctgggag tctctacctg gggaagctga
80161 gacacccaag gctgaggggt gcctggggtg gggggcgctg agaggcatca ggctcacatc
80221 tgcggggaag ctgcgggctg tctgtggccg tcctgcatgg gccccgctca tccctggcct
80281 tttccacagt gtgtgccggg atgggcggct gcactgtagg cagatccggc tgatcggcca
80341 gagtaagtgg cactgccccg gccacccctc cccagccacc cctccctgcc tgccctggcc
80401 accctccccg gccacccctc ccgggcctgc ctgagacccc cagcttcagc tggagctgag
80461 gtggcccctc cgtcccacag gctgcacggc cccaaagatc cacatggact gcagcaacct
80521 gactgcactg gccacctcga agccccgagc cctcagctgc cagacgctgg ccgccggcta
80581 tgtgcgtgtt gggggcgctg ctgtgggcgg gcagggattc ctggctggct gagcctggct
80641 cttgtgctgt gcccccgcta gggtctgggt gccgagtcct gaggacgcag gccctgttga
80701 tgctgtccct ggccctggga gggaagtggc agcctgtgag ccaccggggc acaggggcca
80761 gtgtagggcc cttggccggc agccctcacc agtctcactg ccctgtggcg ggcccaaggg
80821 gagggaagcc tgagcccagg ccagggggag tggtgggagg tctgggacat gacagagact
80881 gcacggtcag gcctttcctg gttgcacatc caatcctgac cccagggagg gctgcagcct
80941 cacctgtcca cccctgaacc ccactctctg gctgtcccca gtaccacaca gagtgtgtca
81001 gtggctgtgt gtgccccgac gggctgatgg atgacggccg gggtggctgc gtggtggaga
81061 aggaatgccc ttgcgtccat aacaacgacc tgtattcttc cggcgccaag atcaaggtgg
81121 actgcaatac ctggtaagct ggcccggcct gtcctggctg cctcccaggc cccacgtgct
81181 ccgcaggggt ggccactgga gagcggtcca aggggcaagt gcctctcctg ggggttccgc
81241 ctgggtcttg cgagatcctg tggtggcccc tgtcccacgg gcagggtggt ctctcatgtc
81301 aactgctggt cttgaagcca tgggagaagg gacatttgga gccacttttg gggcctgcag
81361 gtgtcctgtg tgggaggcac agggagctgt ctgcacggtg cccagggtct cctccagcca
81421 cccatgagca ggtcctgggt cccttcaggc tcctctcctg tcctcctcag cacctgcaag
81481 agaggacgct gggtgtgcac ccaggctgtg tgccatggca cctgctccat ttacgggagt
81541 ggccactaca tcacctttga tgggaagtac tacgactttg acggacactg ctcctacgtg
81601 gctgttcagg tgtggtcacg ggcactgcct ggtcgggctg cttatggtca gggaccctct
81661 gcctgcccca agtgcagtgc ttagctcccc gagaaaccct gagacttggg aaggccggcc
81721 tttcctcagc cccagacccg cacctgcacc cgcaggagga ttcgttcttc tagccagggc
81781 tgggtagggg tggtaaaacc cctctgtact gcccagttct gtggttctcc tctgggtcct
81841 cctctgggtt ctcctgtggg tcctcctctg tggttctcct ctgggtcctc ctctgggtcc
81901 tccctcctct ggatcctccc tcctctggat cctccctcct ctgggtcctc cctcctctgg
81961 gtcctccctc ctctgggtcc tcctccaggt cctcctctgg gtcctccctc ctctgggtcc
82021 tcctctgggt cctcctctga gtcctcctct gggtcctccc tcctctgggt cctcctctga
82081 gtcctcctct gggtcctccc tcctctgggt cctccctcct ctgggtcctc ctctaggtcc
82141 tcctctgtgg tcctcatttg ggtcctcctc tgggtccttc tctgggtgca caaggtgggt
82201 gcaccagcca tggggactga gggcacctgt ttggggagct gagtaaaggc cagggctagg
82261 ccgctgcccg cgcggctctc cagatccaaa tcccacagcc ctttgaggca ccgtgatccc
82321 cagggacagg ggacaggcct gcagcagggt caggtccttg gatgggccag gccagggcct
82381 ggtttgtctg ctcagtggct gtgaccctgc caactggggc gggtgtgccc cgggacacct
82441 ggggtccagc tgtcctggct gaccttgccc tcctggcccc caggactact gcggccagaa
82501 ctcctcactg ggctcattca gcatcatcac cgagaacgtc ccctgtggca ctacgggcgt
82561 cacctgctcc aaggccatca agatcttcat gggggtgagt gctgctggcc ctggggacgc
82621 gtgagccctg cgggaccctc agaccagcca gtgactgggc ctctcctccg ggcagaggac
82681 ggagctgaag ttggaagaca agcaccgtgt ggtgatccag cgtgatgagg gtcaccacgt
82741 ggcctacacc acgcgggagg tgggccagta cctggtggtg gagtccagca cgggcatcat
82801 cgtcatctgg gacaagagga ccaccgtgtt catcaagctg gctccctcct acaaggtggg
82861 ctgcctccct gcctgccctg ccccctcctg gccagccccc caccccctgc cctggtgttt
82921 gcaggacaag cccctgtcct ccctccagcc cctttttgga gcccctgtga tgcttgtctc
82981 ttgcagggca ccgtgtgtgg cctgtgtggg aactttgacc accgctccaa caacgacttc
83041 accacgcggg accacatggt ggtgagcagc gagctggact tcgggaacag ctggaaggag
83101 gcccccacct gcccagatgt gagcaccaac cccgagccct gcagcctgaa cccgcaccgc
83161 cgctcctggg ccgagaagca gtgcagcatc ctcaaaagca gcgtgttcag catctgccac
83221 agcaaggtgg gctggccggg ccatggtggg gcaagtaggc agaggagggc tgtaggtggg
83281 ctgtgactgt gggctggggc catgggcggg gccgactaag cagagcaggg ctgtaggtgg
83341 gctatagctg tgggcggggc catgggcggg gccgactaag cagagcaggg ctgtaggtgg

83401 actatagctg tgggcggggc atggcggggc taactaggca gagcagggct gtaggtgggc
83461 tatagctgtg ggcggggcca tgggcggggc cgactaagca gagcagggct gtaggtggac
83521 tatagctgtg ggcggggcca tgggcggggc cgactgtagg cagagcaggg ctatgggctg
83581 actgtgggcg tggtgagggt gccgtagagc atgctaatga ccagggcgtg gtcatagcag
83641 ggtagggtct tgggtgctcc tggggctggg gggcttctcc acatgctccc cacaccttca
83701 ggagtcgccc tgctgcgtca cgcaccacac ggcgcttgtc ctccagcttt ggctctggcc
83761 gctgcctcct ttggtcacat gaccgtataa tcggcctccc ctctgagacc ctgggctgga
83821 cccccggcct ccctctgcct ccccaggctc agatattcac ccggagggag aaaggacatg
83881 tgtcccccat gcccacacat ccccagctac aggcagctgg ggaggacggg ttctaggatg
83941 gccatgttac agctgaggat gcagaggggt tgggtgatgg gtctgcacag ccacggcggg
84001 acaggtgtct ctggaccctc tccccaaggt tggccctgcc ggggccctgg ctggctggtg
84061 ctgggtaatg tgccctgtcc caggagcagg gccggcctca gggtcctgag ctccagggca
84121 ctggggaagt cctggctcca tgagggcagg acgggcccag gacagaccag ggtgttctcc
84181 ccaggtggac cccaagccct tctacgaggc ctgtgtgcac gactcgtgct cctgtgacac
84241 gggtggggac tgtgagtgct tctgctctgc cgtggcctcc tacgcccagg agtgtaccaa
84301 agagggggcc tgcgtgttct ggaggacgcc ggacctgtgc cgtaagagcc tgcccgaact
84361 gcactcaggg ccgggacggg ggctgggagg tgctgtattg cgggccgggg tgacactcct
84421 tgtccatcca ggtgatgggt gtgcatcacc cacccttcc ccgacttctc cagtgtcctt
84481 ctttggggcc ctgtgggacc cgggttggca gagcaagctt gatgcgtctg cgtcccagcc
84541 cccgacccca gattcgccct caccccggcc caggcctgag ccctcctgcg tctgaccctg
84601 gccctgtctc ccccaagcca tattctgcga ctactacaac cctccgcatg agtgtgagtg
84661 gcactatgag ccatgtggga accggagctt cgagacctgc aggaccatca acggcatcca
84721 ctccaacatc tccgtgtcct acctggaggg tgagcagggt ggggcgggct tcagcggggg
84781 tgatggccga ggggcctgga ggctgagtgg ggcagccctc gggagaggca acagtccact
84841 ggcctggagg gtgagccagg cggccctcgg gggaggctac ggccgacggg cctggcactg
84901 tggggctgaa ggctgatgtc tggagaccca tggggacacc cggagggagg cctgaccctc
84961 agggtaccca cagcccaggg cagccaggct ccccttgctg caggatcagg agggaagcag
85021 gctatcgtgg aaactgggag tggcaggggt gggaggtgct gaggttcgtg cagagcaggg
85081 cgggttgggg agcatttcag gcacaggtca ggggaggccc ctgccgggtg ctggtgtctg
85141 agctgagaac cagtgacgtg aaggagggac tggtgggaag tttgggagga gtatcccgcc
85201 atgggagagg aacatgggtc ttgggactca gggctgctcg gggggcccga tgagactggg
85261 cagggctcct cagcaggcag cgttcagggc tcagtggggt ggggagatcc aggccctgcc
85321 tttccaatcc ccggccttcc cagaggggca tcctgcagag aagggcctgc cagggtaggg
85381 acggtgggtg gggtgtggtg gactgcggtg gtcccaaccc tatgccctgt gtccaccagg
85441 ctgctacccc cggtgcccca aggacaggcc catctatgag gaggatctga agaagtgtgt
85501 cactgcagac aagtgtggct gctatgtcga ggacacccac tacccacctg gagcatcggt
85561 tcccaccgag gagacctgca agtcctggta cctaagccca cgtggcaggg ggcctggggg
85621 agctgcacat atgggcacat gagtacacac acacgtgtga gcacacagtg tacacagtac
85681 acagacacac aaccgttcca catgggtgca catgcacaca aacgcacaca gcataccacg
85741 tgcacacaca cggtcacatg catgcatggt gcacacatgc acacatgaat ggatgccaac
85801 atgcaggcac acacagtcac acatgcacac agcgcacaca tggacacatg cctagacgca
85861 gatacccagg catacactca cggttacaca ctcacgcaca tatgcatgga tgcagacacg
85921 caggcacaca cggtcatata gtcatacacc acatgcacac atgcacagac agacacccag
85981 gcacacacag ttacacagtc acacatgcac acatgcatgg atgcagacac gcaggcgcac
86041 acacacatgc acagtgcaca cgtacacatg cctagacaca gatacccagg cacacacagt
86101 cacacatgca tggacacaga gtcacatgtg cacacataca cacgtgtgga cagacatagg
86161 cacagtcacg tgcacacatg cactcacact cagtcacaca tgaacatgtg ctcacatgca
86221 tggacactga cacgcaagga cacacagtca cacatgcaca catgcataga cacagacacc
86281 caggcacaca cagttacaca gtcacacatg catggatgca gacacgcagt cacacagtca
86341 cacatgcaca cactgcacac atgtacacat gcctagacac agatatgcag gcacacacac
86401 atagtcaaac atgcacacat gcatggacac aaagtcacac gtgcacacat gcacacatgc
86461 atggacagac acaggcacac acagtcacgt gcacagatgc actcacagtc acacatgaac
86521 acatgctcac atgcacagac actgacacgc aggcacacac agtcacacat gtacacgtgc
86581 ctagacacag atacccagac acacacaatt acacagtcgc acagtcacac atgcatggat
86641 gcagacacac aggtacacaa ggtcacacag tcatataatg cacacatgca cacatgcata
86701 gatacagaca cccaggtaca cactcacggt gacacagtca cacatgcaca catgcatgga
86761 ggcagacaca caagcacaca cagtcacaca gtcacacatg cacacaggag ccaggctaca
86821 gaggtaccag tccctcactg cggcgggggg tcttctgttc tcatcccatc ctctgggtct

86881 ggctttttcc ttcctctcct cgcccctgct ctgttcccac agttacaacc cagtgggggg
86941 ctcttccgga gctggctttg gggcagtgcc tgggggcttt gggctcggta ctagccacat
87001 ggggaagctg gggtctgag cagcgtgggc gcgttgtcag tggagtggga cttgtagcca
87061 tgtgcttgct ttgcagcgtg tgtaccaact cctcccaagt cgtctgcagg ccggaggaag
87121 gtaagctgcc ctctgctgcc agccctgcgg tggccgggcc catcctgggg aagcctgtgg
87181 ggccttggat cggtgggggg tgctggtctc ctcctgggct ctgcccottt ggtccccccc
87241 cagctcagac ccacctccga tgtgtatcag ccctgggggg ctgctgtgac ccattttgtt
87301 tcttctgggg tgtcggtgtc ctgtggggaa tttccgtcac cctctcccgt gatccagctt
87361 ctgcgttctg atgagattcc ctttattcaa agagaggggc tctgggacgg gtgcagtctc
87421 actggagcat ttcttagctg cttgtggggg ctcgggcaca cctggccttc ttcctatctt
87481 gctcctgatg aggtgattct tggcctcacc ctcaccccca ggaaagattc ttaaccagac
87541 ccaggatggc gccttctgct actgggagat ctgtggcccc aacgggacgg tggagaagca
87601 cttcaacatc tgttccatta cgacacgccc gtccaccctg accaccttca ccaccatcac
87661 cctccccacc acccccacca ccttcaccac taccaccacc accaccaccc cgacctccag
87721 cacaggtaag gccccctggt tccctccatg cttcctcggg ctctcacctt cccctgcatc
87781 cagcatccag cacagagggc tctttcgggg gcaggccccg gcctggtgca gccaggctgt
87841 gacccctgca caccagctgc agagtgaggt gacagtggca ttcctctgca ctgaggtgtg
87901 agggggcctg ccctggctcc cctggcctgg tgcattgaga tagtagcatc ctgaccacat
87961 ccccaagccc agaccacagt ggaggatcac ctggggagat ttctgaaaac cagcaggaaa
88021 ctatccctaa gggttagaga aattttctta tgttcccctg cgtttgttct ggttgaaatc
88081 ctagctacca ctgaacaagc caccaggggt atgatagcca cagaaaaaag aaactttttt
88141 taaaaaaggc aagattttaa aagatcttga actatataat gatatcctct tttcttcctg
88201 ctttattgca gttttatcaa caactccgag taagtgacgg tgatgatatt catgatgaca
88261 agcagggtgg gaggagcgaa gtcttataaa atcacctgca ggatgcttcc ttcagggccc
88321 agatgtgagg ctggcggggc tggactcctc tgcttatgga ccaaagatgg atgtattttg
88381 gccacttcat tcatggtttg ctgaggccag gggctaaagt gagacctgat tggctgtcgg
88441 tgacaatatt gctggttaag agtggagaca aagccccttc cgtcacactt ccttactgga
88501 atgggaagct ctcttgttat tgattctttg aaaaaaaagt attgaaaata gctgaggaaa
88561 gggtccatca cacccaggtg tggccctggg tggccccgtc tctttgggct caggttttca
88621 gttgcaaaat gaggatggaa gtggtgtcca gccctgagct ctctggccct gcactctggt
88681 tttttggcaa tgacagggaa aagagagatt gcagctgggg gatggtcatg gaggtccctg
88741 ggtcctctga atcctggtgg cttcctggag gtgcctctcc ccaggtgtga gagacaagaa
88801 cttggttttg cttccctaga gctgtgctgc ctctggtctg actggatcaa tgaggaccac
88861 cccagcagtg gcagcgacga cggtgaccga gaaacatttg atggggtctg cggggcccct
88921 gaggacatcg agtgcaggtc ggtcaaggat ccccacctca gcttggagca gctaggccag
88981 aaggtgcagt gtgatgtctc tgttgggttc atttgcaaga atgaagacca gtttggaaat
89041 ggaccatttg gactgtgtta cgactacaag atacgtgtca attgttgctg gcccatggat
89101 aagtgtatca ccactcccag ccctccaact accactccca gccctccacc aaccagcacg
89161 accacccttc caccaaccac cacccccagc cctccaacca ccaccacaac cacccctcca
89221 ccaaccacca cccccagccc tccaataacc accacgacca cccctccacc aaccaccact
89281 cccagccctc caataagcac cacaaccacc cctccaccaa ccaccactcc cagccctcca
89341 accaccactc ccagccctcc aaccaccact cccagccctc caacaaccac cacaaccacc
89401 cctccaccaa ccaccactcc cagccctcca acgactacgc ccatcactcc accagccagc
89461 actaccaccc ttccaccaac caccactccc agccctccaa caaccaccac aaccacccct
89521 ccaccaacca ccactcccag tcctccaacg actacgccca tcactccacc aaccagcact
89581 actacccttc caccaaccac cactcccagc cctccaccaa ccaccacaac cacccctcca
89641 ccaaccacca ctcccagccc tccaacaacc accactccca gtcctccaac aatcaccaca
89701 accacccctc caccaaccac cactcccagc cctccaacaa cgaccacaac cacccctcca
89761 ccaaccacca ctcccagccc tccaacgact acacccatca ctccaccaac cagcactacc
89821 acccttccac caaccaccac tcccagccct ccaccaacca ccacaaccac ccctccacca
89881 accaccactc ccagccctcc aacaaccacc actcccagcc ctccaataac caccacaacc
89941 acccctccac caaccaccac tcccagctct ccaataacca ccactcccag ccctccaaca
90001 accaccatga ccaccccttc accaaccacc acccccagct ctccaataac caccacaacc
90061 acccсttcct caactaccac tcccagccct ccaccaacca ccatgaccac cccttcacca
90121 accaccactc ccagccctcc aacaaccacc acgaccaccc ttccaccaac caccacttcc
90181 agccctctaa caactactcc tctacctcca tcaataactc ctcctacatt ttcaccattc
90241 tcaacgacaa cccctactac cccatgcgtg cctctctgca attggactgg ctggctggat
90301 tctggaaaac ccaactttca caaaccaggt ggagacacag aattgattgg agacgtctgt

NG-7351

90361 ggaccaggct gggcagctaa catctcttgc agagccacca tgtatcctga tgttcccatt
90421 ggacagcttg gacaaacagt ggtgtgtgat gtctctgtgg ggctgatatg caaaaatgaa
90481 gaccaaaagc caggtggggt catccctatg gccttctgcc tcaactacga gatcaacgtt
90541 cagtgctgtg agtgtgtcac ccaacccacc accatgacaa ccaccaccac agagaaccca
90601 actccgacac caatcaccac caccactacg gtgaccccaa ccccaacacc caccagcaca
90661 cagagtacaa caccaacacc catcaccacc accaatacgg taaccccaac cccaacccccc
90721 actggcacac agaccccaac cccgacaccc atcaccacca ccaccactat ggtgacccca
90781 accccaacaa tcaccagcac acagacccca accccgacac ccatcaccac cactacggtg
90841 accccaaccc caacacccac cagcacacag agaacaacac cgacatccat caccaccacc
90901 accacggtga ccccaacccc aacacccacc ggcacacaga ccccaaccac gacacccatc
90961 accaccacca ccacggtgac cccaacccca acacccaccg gcacacagac cccaacaacg
91021 acacccatca gcaccaccac cacggtgacc ccaaccccaa cacccactgg aacacagacc
91081 ctaaccccaa cacccatcac caccaccact acggtgaccc caacccctac acccaccggc
91141 acacagaccc caacatcgac acccatcacc accaccacta cggtgacccc aacaccaaca
91201 cccactggca cacagacccc aaccctgaca cccatcacca ccaccactac ggtgacccca
91261 accccaacac ccaccggcac acagacccca accacgacac ccatcaccac caccactacg
91321 gtgaccccaa ccccaacacc caccggcaca aagagtacaa ccccgacatc catcaccacc
91381 accactatgg tgaccccaac cccaccaccc actggcacac agaccccaac cacgacaccc
91441 atcaccacca ccactacggt gaccccaacc ccaacaccca ccggcacaca gaccccaacc
91501 ccgacaccca tcaccaccac caccacggtg accccaaccc caacacccac cggcacacag
91561 accccaacat cgacacccat caccaccaac actacggtga ccccaacccc aacaccaacc
91621 ggcacaccga gtacaaccct gacacccatc accaccacca ctacggtgac cccaacccca
91681 acacccaccg gcacacagac cccaacatcg acacccatca gcaccaccac tatggtgacc
91741 ccaaccccaa cacccaccgg cacacagacc ccaacccta cacccatctc caccaccact
91801 acggtgaccc caaccccaac acccaccggc acacagaccc caaccccgac acccatcacc
91861 accaccacca cggtgacccc aaccccaaca cccaccggca cacagacccc aacatcgaca
91921 cccatcacca ccaccactac ggtgacccca accccaacac ccaccggcac acagacccca
91981 accacgacac ccatcaccac caacaccacg gtgaccccaa ccccgacacc caccggcaca
92041 cagaccccaa ccacggtact catcaccacc accactacga tgacaccaac cccaacaccc
92101 accagcacaa agagtacaac cgtgacaccc atcaccacca ccactactgt gaccccaacc
92161 ccaacaccca ccggcacaca gagtacaacc ctgacaccca tcaccaccac cactacggtg
92221 accccaaccc caacacccac cggcatacag accccaacaa cgacacccat cagcaccacc
92281 accaccgtga ccccaacccc aacacccacc ggcacacaga ccccaacatc gacacccatc
92341 accaccacca ctacggtgac cccaacccct acacccactg gcacacagac cccaacatcg
92401 acacccatca gcaccaccac tacggtgacc ccaacagcaa cacccaccgg cacacagacc
92461 ccaaccctga cacccatcac caccaccact acggtgaccc caaccccaac acccaccggc
92521 acaaagagta caaccccgac atccatcacc accaccacta cggtgacccc aaccccaaca
92581 cccactggca cacagacccc aaccacgaca cccatcacca ccaccaccac ggtgacccca
92641 accccaacac ccaccggcac acagacccca accccgacac ccatcaccac caccaccacg
92701 gtgaccccaa ccccaacacc caccagcaca cagaccccaa catcgacacc catcaccacc
92761 accactacgg tgaccccaac cccaacaccc actggcacac agaccccaac cacgacaccc
92821 attaccacga ccaccacggt gaccccaacc ccaacaccca ccggcacaca ggccccaacc
92881 ccaacagcca tcaccaccac cactacgggg accccaaccc caacacccac cggcacacag
92941 accccaacca cgacacccat caccaccacc actacggtga caccaacccc aacacccacc
93001 ggcacacagt ccccaacccc aacagccatc accaccacca ctacggtgac cccaacccca
93061 acacccaccg gcacacagac cccaaccacg acacccatca ccaccaccac cacggtgacc
93121 ccaaccccga cacccaccgg cacacagagt acaaccctga cacccatcac caccaccacc
93181 acggtgacac caaccccaac acccactggc acacagaccc caacatcgac acccatcacc
93241 accaccatta cggtgacccc aaccccaaca cccaccggca cacagacccc aaccccgaca
93301 cccatctcca ccaccactac ggtgacccca accccaacac ccaccggcac acagacccca
93361 acatcgacac ccatcaccac caccaccacg gtgaccccaa ccccaacacc caccggcaca
93421 cagaccccaa caacgacacc catcagcacc accaccacgg tgaccccaac cccaacaccc
93481 accggcacac agaccccaac atcgacaccc atcaccacca ccaccacggt gaccccaacc
93541 ccaacaccca ccggcacaca gaccccaacc acgacaccca tcagcaccac caccacggtg
93601 accccaaccc caacacccac cggcacacag accccaacat cgacacccat caccaccacc
93661 accacggtga ccccaacccc aacacccacc ggcacacaga ccccaacccc gacacccatc
93721 accaccacca ccacggtgac cccaacccca acacccaccg gcacacagac cccaacatcg
93781 acacccatca ccaccaccac cacggtgacc ccaaccccaa cacccaccgg cacacagacc

93841 ccaaccccga cacccatcac caccaccacc acggtgaccc caaccccaac acccaccggc
93901 acacagaccc caaccccgac acccatcacc accaccacca cggtgacccc aaccccaaca
93961 cccaccggca cacagacccc aacatcgaca cccatcacca ccaccactac ggtgacccca
94021 accccaacac ccaccggcac acagacccca accacgacac ccatcaccac caccaccacg
94081 gtgaccccaa ccccaacacc cactggcaca cagagtacaa ccctgacacc catcaccacc
94141 accaccacgg tgacaccaac cccaacaccc accggcacac agaccccaac atcgacaccc
94201 atcaccacca tcactacggt gaccccaacc ccaacaccca ccggcacaca gaccccaacc
94261 ccgacaccca tctccaccac cactacagtg accccaaccc caacacccac cggcacacag
94321 accccaacca tgacacccat caccaccacc accacggtga ccccaacccc aacacccacc
94381 ggcacacaga ccccaacaac gacacccatc agcaccacca ccacggtgac cccaacccca
94441 acacccaccg gcacacagac cccaacatcg acacccatca ccaccaccac tacggtgacc
94501 ccaaccccaa cacccaccgg cacacagacc ccaaccacga cacccatcac caccaccacc
94561 acggtgaccc caaccccaac acccaccggc acacagagta caaccctgac acccatcacc
94621 accaccacca cggtgacacc aaccccaaca cccaccggca cacagacccc aaccccgaca
94681 cccatctcca ccaccactac ggtgacccca accccaacac ccaccggcac acagacccca
94741 accatgacac ccatcaccac caccaccacg gtgaccccaa ccccaacacc caccggcaca
94801 cagaccccaa caacgacacc catcagcacc accaccacgg tgaccccaac cccaacaccc
94861 accggcacac agaccccaag atcgacaccc atcaccacca ccactaaggt gaccccaacc
94921 ccaacaccca ccggcacaca gaccccaacc ccgacaccca tcaccaccac caccacggtg
94981 accccaaccc caacacccac tggcacacag gccccaaccc cagcagccat caccaccacc
95041 agtacggtga ccccaacccc aacacccacc ggcacacaga ccccaaccac gacacccatc
95101 accaccacca ccacggtgac cccaacccca acacccaccg gcacacagag tacaaccctg
95161 acacccatca ccaccaccac cacggtgaca ccaaccccaa cacccaccgg cacacagacc
95221 ccaacatcga cacccatcac caccaccact acggtgaccc caaccccaac acccaccggc
95281 acacagaccc caaccccgac acccatctcc accaccagta cggtgacccc aaccccaaca
95341 cccaccggca cacagacccc aaccatgaca cccatcacca ccaccaccac ggtgacccca
95401 accccaacac ccaccggcac acagacccca acaacgacac ccatcagcac caccaccacg
95461 gtgaccccaa ccccaacacc caccggcaca cagaacccaa catcgacacc catcaccacc
95521 accactacgg tgaccccaac cccaacaccc accggcacac agaccccaac catgacaccc
95581 atcaccacca ccaccacggt gaccccaacc ccaacaccca ctggcacaca ggccccaacc
95641 ccaacagcca tcaccaccac cactacggtg accccaaccc caacacccac cggcacacag
95701 accccaacca cgacacccat caccaccacc accacggtga ccccaacccc aatacccacc
95761 ggcacacaga gtacaaccct gacacccatc accaccacca ccacggtgac accaacccca
95821 acacccaccg gcacacagac cccaaccccg atacccatct ccaccaccac tacggtgacc
95881 ccaaccccaa cacccaccgg cacacagacc ccaaccatga cacccatcac caccaccacc
95941 acggtgaccc caaccccaac acccaccggc acacagaccc caacaacgac acccatcagc
96001 accaccacca cggtgacccc aaccccaaca cccaccggca cacagacccc aacatcgaca
96061 cccatcacca ccaccactac ggtgacccca accccaatac ccaccggcac acagacccca
96121 accacgacac ccatcaccac caccaccacg gtgaccccaa ccccaacacc cactggcaca
96181 caggccccaa ccccaacagc catcaccacc accactacgg tgaccccaac cccaacaccc
96241 accggcacac agaccccaac cacgacaccc atcaccacca ccaccacggt gaccccaacc
96301 ccaataccca ccggcacaca gagtacaacc ctgacaccca tcaccaccac caccacggtg
96361 acaccaaccc caacacccac cagcacacag accccaaccc cgacacccat ctccaccacc
96421 actacggtga ccccaacccc aacacccacc ggcacacaga ccccaaccat gacacccatc
96481 accaccacca ccacggtgac cccaacccca acacccaccg gcacacagac cccaacaacg
96541 acacccatca gcaccaccac cacggtgacc ccaaccccaa cacccaccgg cacacagacc
96601 ccaacatcga cacccatcac caccaccact acagtgaccc caaccccaac atccaccggc
96661 acacagaccc caaccacgac acccatcacc accaccacca cggtgacccc aaccccaaca
96721 cccactggca cacaggcccc aaccccaaca gccatcacca ccaccagtac ggtgacccca
96781 accccaacac ccaccggcac acagacccca accacgacac ccatcaccac caccactacg
96841 gtgacaccaa ccccaacacc caccggcaca cagtccccaa ccccaacagc catcaccacc
96901 accactacgg tgaccccaac cccaacaccc accggcacac agaccccaac atcgacaccc
96961 atcaccacca ccactacggt gaccccaacc ccaacaccca ccggcacaca gaccccaacc
97021 ccgacaccca tctccaccac cactacggtg accccaaccc caacacccac cggcacacag
97081 accccaacca cgacacccat caccaccacc accacggtga ccccaacccc gacacccacc
97141 ggcacacaga ccccaaccac ggtactcatc accaccacca ctacgatgac cccaacccca
97201 acacccacca gcacaaagag tacaaccgtg acacccatca ccaccacaac tacggtgacc
97261 gcaaccccaa cacccaccgg cacacagacc ccaaccatga tacccatcag caccaccact

97321 acggtgaccc caaccccaac acccaccact ggaagcacgg ggccccccac ccacacaagc
97381 acagcaccca ttgctgagtt gaccacatcc aatcctccgc ctgagtcctc aacccctcag
97441 acctctcggt ccacctcttc ccctctcacg gagtcaacca cccttctgag taccctacca
97501 cctgccattg agatgaccag cacggcccca ccctccacac ccacggcacc cacgaccacg
97561 agcggaggcc acacactgtc tccaccgccc agcaccacca cgtcccctcc aggtaagcag
97621 agctgcttgg ttcctctggc ctgggatgct tcttcctccc cttgtgccgg gcaggactgt
97681 cccaggaagg ctcaaggcac gttctgggcg cctctctgcc cacgaagctt ggtcactgtg
97741 tgggcagaag ccactgacac tggccagtgc tgggcagtga agccaaaggc cattccgctt
97801 gcccatagga cagccttctg aggagctgct gacaccggcc agtgctgggc agtggagccc
97861 ttggctatcc tgctcgccca taagacggcc ttcttcaggg gcccactgct atgtgatgcg
97921 gtgctgtggg agcccatcaa ggctgggggg cagagagagg ctgccagtga ggtgcctgcg
97981 ggtccacctg cttctggctg cagcccctcc ttggggcctt ttcctggtgg acggcgtgcc
98041 acagccagtg ccttctggac gcctcttgct ggccatcggc ttggccagca agctgtgttg
98101 ctgccagagc accaggtcac ctgcaggctc tcgtgacact cggctgtggt gatactggcc
98161 ttgccgctcc accctgcctg gtgactctga gagcctggga ggtgggcacg aggccctggt
98221 cctccagttc tgccacccgg tcggctgtct ggctcccttg cagctgggga gtggcagttg
98281 ggaccctgtg gcatctgaga tgtgcaatgt ctcagccctc actggtgtct cctgctctca
98341 caggcacccc cactcgcggt accacgaccg ggtcatcttc agcccccacc cccagcactg
98401 tgcagacgac caccaccagt gcctggaccc caacgccgac cccactctcc acacccagca
98461 tcatcaggac cacaggcctg aggccctacc cttcctctgt gcttatctgc tgtgtcctga
98521 acgacaccta ctacgcacca ggtactcagg ctgttcacat cctgtgcttg ggtggccgag
98581 gctggccccg gcatgtacca atgggtcagg tgccagggct gagatcgcag tagaagcgtc
98641 tcaggaggca gcagccgtcg agggtggctg tgtccagggc acggcttccc ttgggtggcc
98701 tctgtgggga cctccgctgt ggggacctcc acggggtcca gcggctagcc ctgcctccgg
98761 atagccctgc ctctggacgg tgtgatcgtg ggtctgtctc ccttcgcagg tgaggaggtg
98821 tacaacggca catacggaga cacctgttat ttcgtcaact gctcactgag ctgtacgttg
98881 gagttctata actggtcctg cccatccacg ccctccccaa cacccacgcc ctccaagtcg
98941 acgcccacgc cttccaagcc atcgtccacg ccctccaagc cgacgcccgg caccaagccc
99001 cccgagtgcc cagactttga tcctcccaga caggtcagtg ggctgcaggc ggctttgtcc
99061 ccatggcact ctgcgcagca tgtccgggca gctgaggccc caggcaccac ttcctgctgg
99121 tcgtctgagg gccgaggcct ccagcaaccc ttgggtgcag ggtctgccga gccctccaca
99181 ttttcaccgt gccccgctgt gcctggcgag gtggctggct gcagtgaggt ccgtggaagc
99241 cacttcggcc tccagcctcc cggctcagca cccgcccctc ctgagcgcag accaccccat
99301 cctgtgccgg tccccctgac gtcccttgcc tcccgtcccc aggagaacga gacttggtgg
99361 ctgtgcgact gcttcatggc cacgtgcaag tacaacaaca cggtggagat cgtgaaggtg
99421 gagtgtgagc cgccgcccat gcccacctgc tccaacggcc tccaacccgt gcgcgtcgag
99481 gaccccgacg gctgctgctg gcactgggag tgcgactgtg agtccggggc ccccaggccc
99541 tccccgcatc tcctgccctc tccgtgggtg ggggctgcag ggcccgtctc ccgggggcgg
99601 aagggctgag gctccttggg cacagatccc actgaggtgt tcgctgaggc tgggtgactt
99661 ctgagggtct tctcacagcc ctgcttttgc ctcattgggt ggggagggcc tgggcaggtg
99721 gagggcttgc ctggtggagt tagggctcct ccctggaaca agggtgcttc tgaggcaaga
99781 gggggctgag ttgaagtttg aaccctggtc cgtcctgcag aatgggccac tgtgggtgcg
99841 ccagggcaag tgcagctcag acatccccgt gcccacgcac aggagtgggg ttttcaggcc
99901 ccagcttcct gctggctctt cctgactatg ccccagccca gcccttgcac ccgaccccgg
99961 ccgaggggca caggtggcac ggctcactcc ggctcccttg caggctactg cacgggctgg
100021 ggcgacccgc actatgtcac cttcgacgga ctctactaca gctaccaggg caactgcacc
100081 tacgtgctgg tggaggagat cagcccctcc gtggacaact tcggagttta catcgacaac
100141 taccactgcg atcccaacga caaggtgtcc tgtccccgca ccctcatcgt gcgccacgag
100201 acccaggagg tgctgatcaa gaccgtgcat atgatgccca tgcaggtgca ggtaggcaca
100261 gcgtggccac aggaggctgg catggaggcg ggtgctgaca tgggccccaa tgcaccctgg
100321 ttccccaggg gccagaggac tgggctgtgg gggtgccaag gcatagcctc tcctagagct
100381 gggctagaag gtaggatggg gtgggcgact ggctccggga catatcagct cttcctgcag
100441 gccctccagg tgtgtcctgg gcccctcgag ccctggcacc atgccacgct gggcacagtc
100501 tctgcagcag aagctgcctc ctgaggacag agtcagggac agggctctgc acacccttgg
100561 ctgagatgcc cctacttgca ggggaatcat tggttctgag gctcaggagg ccccgggagc
100621 ctgcgccggg ctccacagtc cccaggtgct cccaggagag ctccttcact ggctcaccca
100681 tgggaccagg gtctggttgg gagcagtgga gtggaagcaa gaaaggggc aggaaagcgg
100741 ggtaggcagg gccctctccc tacatgtgta ggtcagagag caggcggggt ggggcagccc

100801 tggagctctc acaaggagag gaccgaggca gctgcagctc ccatggtgtg tcggccacag
100861 gtgcaggtga acaggcaggc ggtggcactg ccctacaaga agtacgggct ggaggtgtac
100921 cagtctggca tcaactacgt ggtggacatc cccgagctgg gtgtcctcgt ctcctacaat
100981 ggcctgtcct tctccgtcag gctgccctac caccggtttg gcaacaacac caagggccag
101041 tgtggtgagt tccgtgaccc ccatggcccc cgaggccccc acggctccca ccgtcccctg
101101 tgcccccatg tcctgcccca gggcgggtgg ccaggccagg ctgaggctga ggctgcgtgt
101161 aaacacccat gggcctggct gtgggcctct tgccccgctg ctcggggctg ctgtggccat
101221 cacccgggtt cagtctctgt gaggagccaa caggaggggg cctggcctgg tctctgccct
101281 cggccctggc tggccggtcc tgggcatctg ggctggagaa gggcagggct taccctgtct
101341 gcaacgtggc ctctctcact gatacaggca cctgcaccaa caccacctcc gacgactgca
101401 ttctgcccag cggggagatc gtctccaact gtgaggctgc ggctgaccag tggctggtga
101461 acgacccctc caagccacac tgcccccaca gcagctccac gaccaagcgc ccggccgtca
101521 ctgtgcccgg gggcggtaaa acgaccccac acaaggactg caccccatct cccctctgcc
101581 agctcatcaa ggacaggtga ccccgcccag gcctgcctgt ggccacgaca ccaataagct
101641 gagggcctct gtgccccagc ccccagctct tgcaaagagg aaggaggcag cgcgtggggc
101701 ctggcgctgg ggctgggaag gcacggagcc gcggaaccag gatcaggcgc taggtcgccg
101761 tggggtccag gacccaggcc cttgggttcc acggggctga gctgctacgt gcggcctgtg
101821 cctttgctga actccagtct ctcctggctc ccgggaaggt gcagggctgg ccgagtgtga
101881 ggcccggagt aaaccagtca acccaggaca gagctcaggg ctgatattgg gagggcagat
101941 ttgggctttg acagagaggg ggtgctccta acgctggcag tcatgggggg tcagcatcct
102001 gtccctggaa gtataggggc caggtatagg ctgggtgtcc atctgccagg gttgctggag
102061 ggggtcctga agctgatgac cacatagacg tggtttctat ctctgggagc cggctgcag
102121 agccaccttg ctcggccatc ccttggtctg tccctgagct gtccccctgg ctggcctgtc
102181 ccttgaccct ccatcagcca caggcgcctc tctggcgggt gccggactcc aggaggacag
102241 tccgggcaga gacgctgggg tagagagcag gggagaggca ggtgccacct gagtgtgacc
102301 tgtgcctctc cctgcacagc ctgtttgccc agtgccacgc actggtgccc ccgcagcact
102361 actacgatgc ctgcgtgttc gacagctgct tcatgccggg ctcgagcctg gagtgcgcca
102421 gtctgcaggc ctacgcagcc ctctgtgccc agcagaacat ctgcctcgac tggcggaacc
102481 acacgcatgg ggcctgctgt aagtgcccat ctgcccctgc cctggagctg ggggcctgca
102541 ggccagacgt ggtctctagg ctctgccagg tgctgtgccc agcctgaagc tagacctaga
102601 tgggctgcgg ccagggatgc agagatggcg ggtgtgagac cagggctggg gccatggggt
102661 ggggaaggcc aggctggagg ggctgaggtg ctggggcttc tgccagcatc gctaaatgca
102721 actgggtgcc caccacccag ctcgggacaa cctcgagggt ggaggttgat gcccaggcag
102781 ctggtcaccc tcctccgtgt gtggggcact gggcagctgt cactcaaggg ggtccaggct
102841 cctccgcctg acatgaggca gccctctgac ctctgcccat gtccctcagt ggtggagtgc
102901 ccatctcaca gggagtacca ggcctgtggc cctgcagaag agcccacgtg caaatccagg
102961 tatgttgttt gagggtccac caggaccgtg ggctcgcctt ctgcagtgcg gagggtggca
103021 tcatctgggc atagcagtcc cacctgccag ctccccagcc ccaccccacc tgtctgacaa
103081 tgccctcccg cccccagctc ctcccagcag aacaacacag tcctggtgga aggctgcttc
103141 tgtcctgagg gcaccatgaa ctacgctcct ggctttgatg tctgcgtgaa gacctgcggt
103201 acgccaccca ctcacactgt cccctcctgc ctccctcctg cctcctcctg ggtgtccacg
103261 gaggctggga ccaggacgct gaccaccccc cacctctgat ccctgttgca caaggactct
103321 gctaacacaa cttgtctcct gggtgtccat ggaggctggg accaggaggc tgaccacccc
103381 caccccctgct ccctgctgca caaggactct gctaacacaa cttgtttctt ccctcttcct
103441 aggctgtgtg ggacctgaca atgtgcccag agaggtaggc cccaccgtgt tgctggggga
103501 tccttccaca aattctgaat tctggggagt gagggatgga catgaaaacc tggagcctca
103561 aagattgagg aatgaggtca tctaagtcct ggatggctga gttggcatgg acaccaccca
103621 ctcacccacc catccttcca cccacccact catccacctg tgcacccatc tacccactca
103681 cctacccctc catccttcca cctacctagt catcacccac tcatctatgc acccccaccc
103741 acccactcat ccatccatcc atccaccatc cacctaccca accatccacc catccatcca
103801 ccatccatct accatccacc atccacccaa ccatccacca tccatccatc caccatcatc
103861 catctaccat ccacccaccc acctatccat ccatccatcc accatctgtc taccatccac
103921 ccacccactc atccatccat ccatccacca tctgtctacc atccacccac ccacctatcc
103981 atccacccat ccatccatcc atccatccat ccatccatcc atccatccac ccaccatctg
104041 tctaccatcc acccacccac ctatccaccc atccacccac ccatccatcc acccaaccat
104101 ccaccatcca tccatccatc catccatcca ccatccatct accatccacc ctcccatcca
104161 tccacgcatc cacccaacca tccatccatc catccaccat ccacccacca tccacccatt
104221 tatccatcca ttctccctcc ctccattcac cacccattgg tcatatgata ctctgtctag

104281 aagctctgac atgacatctt ggccacctct gtgctgccca tgcctcctac ctgtggtagc
104341 agccatgtgg atgattcctt agctaaattc tgtacaaacc tgagaggcct gagtggagaa
104401 tttgccacgt gccaagcccc tgcttgtcga tgctggtgag caggtaatgg ctttgtgata
104461 tcagtgaatg agcagctact gtcctatccc agaacctgcc tggtgtgctc agaagtgagg
104521 agggacatgg ttttccccca ggatccctca gcactctgct cagggtggct gtttctcccc
104581 gctgaccaca gctgcagctc cggggctgtg gtgaggtggg gcctgcctgg tgccacctgt
104641 cctctctact caccccttctt tccctgcagt ttggggagca cttcgagttc gactgcaaga
104701 actgtgtctg cctggagggt ggaagtggca tcatctgcca acccaagagg tgcagccaga
104761 agcccgttac ccactgcgtg gaagacggca cctacctcgc cacggaggtc aaccctgccg
104821 acacctgctg caacattacc gtctgcagta aggccatccc ctggggccca tgccacctct
104881 caggggtgca cacatccctg taggctgggc tgcctgctgt cccctccttg gcaagtgagg
104941 aaacagctgg cttgggggcc tctgctgtgc cccttgagag ggcttgggag ggggccgctg
105001 ggcccagtcc aggcatccct gctgcagggc ctgacctggg tggggagggg acccttggag
105061 gtgctggagg cccgaccctg tgcagtggcc ccgggggctt tgcctgggag gagccacccct
105121 cacggccgcg tgcgcaccct gtcttcagag tgcaacacca gcctgtgcag tggccccggg
105181 ggcttggcct gggaggagcc accctcacgg ccgcgtgcac accctgtctt cagagtgcaa
105241 caccagcctg tgcaaagaga agccctccgt gtgcccgctg ggattcgaag tgaagagcaa
105301 gatggtgcct ggaaggtgct gtcctttcta ctggtgtggt aagcagggct ggtgggcagg
105361 gcagggagga ggctgccgcc cggggtgggg tggctgtaag gggttggct ccctcctggg
105421 ggtctcagat tctggggaca cagatggctg tacgcttggc tgatgcaccc accccagccc
105481 tgagcgctcg ctccatccac tgggtgtgca ccgggagtgg gggtctggcc aggtggccgc
105541 cccggggcag tctccaacga acggccttct ccgttctttc tcccaagagt ccaagggggt
105601 gtgtgttcac gggaatgctg agtaccaggt gagccctggg ctgggtgaga gggaggaggg
105661 gaggaggtcg gctgcagcgt gggggtcctg gcaggctgtt gggctggctg ggatgctgga
105721 gaggcccctg cctcatgtct ctccctgtgc ccgaagcccg gttctccagt ttattcctcc
105781 aagtgccagg actgcgtgtg cacggacaag gtggacaaca acaccctgct caacgtcatc
105841 gcctgcaccc acgtgccctg caacacctcc tgcagccctg taagcggcca ccctcctcct
105901 tcagcctgcc cttttccctc ctcccagaca agcacccggg cccatgtctg catcgtgacc
105961 ctttctttcc tcctttcaac gccaacctgt ccctgtcccc acctctccat cctgacacct
106021 gcccagcctg gggcctcctc caggtggggg ggtctcggca gccctgcagg ctttgtgtgg
106081 tgtggggtac agcctgggag ttcagttgca gtggcgtgtc tatgtgcgca gggcttcgaa
106141 ctcatggagg cccccgggga gtgctgtaag aagtgtgaac agacgcactg tatcatcaaa
106201 cggcccgaca accagcacgt catcctgaag gtaggtgtgc actgccggcc ccgacgcggc
106261 cgggttgctt gagcccaggg caaggcgcgg gccacccagg atcccccagc tgagtcctcc
106321 cagtcctggg cgcagctgtg atgggcgccc tggggctgcc atgacaaatg agcaggcgtc
106381 ttcagggcag aaagggattc tcctggttct gcggcccaga aatccataga gcaaagggcc
106441 tcagggctgt gctccctcgg aggcgctagg caaggacctt tcccagcctc tggtcactct
106501 aggtgcccct tggctgtgac cacgaggttt ccttccctgt gtctgcctct cctctccctt
106561 ttaaggattt aggcacccca agcaggatga tctcatctta ggatccttca cttaatgaca
106621 ccttcaaaga ccccctttcc aaggcaggtc acattcatag attcagagtt agaacacaga
106681 cagacctttg agggttgtgt gggctccagg ctggtgcctg atgtggggcc ccgcccatgt
106741 cacttgtcct gtggccctgg gcctcaccag gaagcctccc cggccaggtg tctccagggt
106801 gtcttcctgg ccgggctggg gctgggcctg ctgccctccc tcaccagagc tccctgcccc
106861 acagcccggg gacttcaaga gcgacccgaa gaacaactgc acattcttca gctgcgtgaa
106921 gatccacaac cagctcatct cgtccgtctc caacatcacc tgccccaact ttgatgccag
106981 catttgcatc ccggtgagtt ggccacctgg ggcctggctg tgtgtactct gccgggagtg
107041 ggggtgcctg gtgttctggg gggctggggc cccagtgctg cgacagtgac ctcgggcctg
107101 gtctgagctg ccgcaggagg ctttgcctgg ggctttctgc agcagctacc cccgcccacg
107161 gcatcgtggg aaggtgctct catccccagg aatgtccggg ggtcccgggc tcattctcct
107221 ttccctctag ggctccatca cattcatgcc caatggatgc tgcaagacct gtgagtacag
107281 ggcacagcct gggggtagg cagggtgggg gcacaagggc tggtgccctc agccccgcct
107341 ggggtggctg gaggctggac aacggcctct gggtgggcag tgagggctgg gggctgaggc
107401 cgagcctggg gaggggacgc agcgagggag agcctcctcg aagatgtgga ggccctgccc
107461 taagccgctg cccgctctcc ccaggcaccc ctcgcaatga gaccagggtg ccctgctcca
107521 ccgtccccgt caccacggag gtttcgtacg ccggctgcac caagaccgtc ctcatgaatc
107581 attgctccgg gtcctgcggg acatttgtca tgtgagtccc aggctgggag tgtgcctgga
107641 gggggtggtg gagaccccag ggaggcgaga ggccagcgct ggccccggaa ggtcacccct
107701 cactccgccc tccccccagg tactcggcca aggcccaggc cctggaccac agctgctcct

107761 gctgcaaaga ggagaaaacc agccagcgtg aggtggtcct gagctgcccc aatggcggct
107821 cgctgacaca cacctacacc cacatcgaga gctgccagtg ccaggacacc gtctgcgggc
107881 tccccaccgg cacctcccgc cgggcccggc gctcccctag gcatctgggg agcgggtgag
107941 cggggtgggc acagcccccт tcactgccct cgacagcttt acctccccсg gaccctctga
108001 gcctcctaag ctcggcttcc tctcttcaga tatttattgt ctgagtcttt gttcagtcct
108061 tgctttccaa taataaactc agggggacat gctgtactgt gtggtttagg ttggtgctgc
108121 aggggtgcgg cttggccact gtgcatggcg gaggccacca ggctctgcgt gcaggacacg
108181 ggggcaccac acacacgcca cgcggttggc aaatcccttg taccaaatgc agaggggacg
108241 tgggggctgc ctgccctccg ctccccattc tcaccctggg caaaaccccc acggggctgt
108301 cgaaatgtgg tcagattccc ttgtaggaat cccccggccc tgaatctgtg aaaagagccc
108361 tgggttcctt cacagccatc tcacaggctt tccacggttg gtggggcctt tgtgtagccc
108421 cgagggtcag gtggccctgg ggagggtgtg tgtgtgtgtg tgtgtgtgtg tgtgtgtgtg
108481 tgtgtagccc cgagggtcag gtggtactgg ggagagtgtg tgcgtgtgtg tgtgtgtgta
108541 gccccgaggg tcaggtgacc ctggggaggt gatgtgtgtg tgtgtgtgtg tgtgtagccc
108601 caagggtcag gtggccctgg ggagggtgtc tgtgtgtgtg tgtagccccg agggtcaggt
108661 ggccctgggc agagggtgtg tgtgtgtgtg tgtgtgagtg catagccccg gggtcgggt
108721 ggccctgggg aggtggtgtg tgtgtgtgtg cgtgtgtgcg tgcatgcacg tgtgagaatt
108781 tgctttgcac acgtgtctgc atgtgccatg tgtatgtgat gtgtgtgcac gtgtgtgcat
108841 gcatgtgcgt gtgagcattt gcttgtgtgc acacgtgtct gcatgtgcca tgtatgtgat
108901 gtgtgtgtgc acgtgtgtgt gtgcatgagc gtgtgagcat ttgcttgtgt gcacacgtgt
108961 ctgcatgtgc cgtgtgtatg tgatgtgtgt gcacgtgtgt gtgtgcatga gcgtgctagc
109021 atttgcttgt gtgcacatgt gtctgcatgt gccatgtgta tgtgagatgc gccttgtcct
109081 gggtctctac actgggaaaa tggagaaggt cttagtcctc aggtcactgt cagctcctgc
109141 tgcctagtga caaaaggtgg ccaggacctg gtggccccgt cagggctttg gggagctgcc
109201 agtcagagcc gccaataccт tcaaacctgc agaagttctg gttggctctg cggacccagc
109261 tggacaaggg gcactggctg tggaccctgg gcacagtgcc cactcatgtt ggagagctga
109321 gcccctgact tctgggtgga gcccatggag gctgcttggc ccagagcagg tgcctggtgt
109381 ctcctgtcaa aggcagcagg tgtgtgtggg gaggccgagg cctctaagca cagtgatcca
109441 acctcgagca tcctctgagg tcaaagggga ggacatttgt ctccagacta caaggctgtg
109501 ccacacccct tacatgtgac gaaaccctca ggagggcaga cacctgggct gggctccaca
109561 cttccaaagc cagggcacaa cgggtcctca gggtgtgaca ggtcctcagg gcatgacgga
109621 tcctcaggtg tgatgggtcc cctgggcgtg acgggtcctc agggtgcaac aggtcccacc
109681 tcctgctgtc ctgcctccca aatagttgta caggcatgtg gcaccatgat tggctaattt
109741 ttaagagctt tttgcttttt gttcttatct ttcaaaacaa tcctttgttt tactaaagta
109801 acaggcacgt gatttttgca tagttttgtt tgttttgcag agacagggtc tcactatgtt
109861 gcccaggctg gtctcaaact ccttcctggg ttcaagtgat cctcccacct cagccaccca
109921 aagtaccagg attacaagca tgagccactg atcttggctc ctggcagggg gctgaggcca
109981 ctgccctccc ctagctgccc tcccctagct gccctctccc ccatctgacc ccagggaggg
110041 ccgcggttga gcagcaaccc tggggtctga agccagttgg gacatcccag ctcatcagcg
110101 ccaccctggg tcctggaacc cgctcaggcc tggggtcttt caactgcccc acttggccca
110161 ggacagcccc ccaaaggacc tgctcatccc ctctggtggc cccaggagcc cacactgtct
110221 aggcctaggg acctcctggg cagtcagttg gcctgggcct gccatggagc ccctgaggcc
110281 accctagagc ctctggcata gccacgtggg gattctgggt gcttcagaac tggcccttcc
110341 atgaagctgt cagcatggac cccagagcca tccttgggtc atttcgaggg tggcctgttt
110401 ctggcccacc cccaaaacct atcacatctc tgcctggctg tgacccttgg gcccagcagt
110461 ccctgcacag gccaggaaat gggcagggcg gggtgggggc actgcggcca gagacctggt
110521 aaggaagagg tggtcaggct ccctccagtt tcctcatctg tctcccagct tgcagctgcg
110581 aagaggggct tcccctggtg atgcagcgtg gacacgggta cggctaggcc cctgcctgct
110641 cttccctgct gtgcccttta aagcagaggc tacccgggaa gctccaggag agcatgagcc
110701 ctgaccccag tccttcccca gacagccctg ccccttccca gagcaggccc cacccсttcc
110761 ctagaggagg ccccgcccct tccccagagc aggccccacc ccttccctag aggaggcccc
110821 gccccttccc cagagcaggc cccacccctt ccctagagga ggccccgccc cttccccaga
110881 gcaggcccca ccccttccct agaggaggcc ccaccccttc cccagagcag gccccacccc
110941 ttccctagag gaggccccgc cccttcccca gagcaggccc caccccttcc ctagaggagg
111001 ccccgcccct tccccagagc aggccccacc cctttcctag gacaggcccc gccccttctg
111061 cccactcagg atcttccttt tgggttctct gtgtccagaa ctccagtgca ggtgttgagg
111121 gtggggaggg agctgcccct tcaggtggag gcagggttgg tgccaacgga ggggcaggga
111181 gacgcagggg ctccccccaa ccccgtccag tcacggtgca gcccccgact ttatccccag

111241 cgccctcctc ttcctcgcat aactcatgcc cccagctggg tcctcctggg tctccctagg
111301 ggtgactcgg gccaggggct acctgtttcc ccgggctcac cacagtgggc taagcctaca
111361 gcagaggaga tagggagccc gccagccagg tgggcagccg gccacccccct cggagtagct
111421 gcacgggttg gggtcaagtt ctcgcattct ctggaagaag ctggctgttc tgttcccacg
111481 gcggcctccc tgtttctggg agcagacgga aggccccagc gcgcactcct cccttccgcc
111541 caggtgagac ttgctgttgt tccgtgggct gaaacaggcc atgcgcctgg ctgtcgggtg
111601 ctccgggcgg caccagcaaa tgaccacaaa ccgggggccg aaagccacag gaaagctgtc
111661 tcccacagtc ccggaggccg gagtctgagg tgcaggcgtg gcggtgccac aaggccgctc
111721 aggctccggg gaagatgctt ttggcccttc cagctgcagg tggctctggg cgttccttgg
111781 cttgcggctg catcgcccat gccctgcctc cgtctccacg gggcttctcc tgggcgtttc
111841 tccctttgcg tctcttctaa ggacattggt ctttggattt agggcccacc tgataatcca
111901 ggctgatctc gtgaggtcct caatctaatc acatctgcaa agaccctttt tctttttctg
111961 tttttggaga cagattctcg ctctgtcacc caggctagag tgcagtggtg tgatatcggc
112021 tcactgcaac ctccgcctcc cgggttcaag cggttctcct gtctcagcct cctgagtagc
112081 tgggattaca ggcgcccacc accacaccca gctgattttg tatttttagt agagatgaga
112141 tttcaccatg ttggccaggc tggtctcgaa ctcctgaccc caggtgatct gcccgcctcg
112201 gcctcccaaa atgctgggat tatagggggtg agccactgca cctggccccg agtcttggtg
112261 gttcacggca gcagggttct gtctggttca tgctccgttt gtggctgcgt ggctggtgcc
112321 ctccactaca tccgctcact ccagggccta ggctgtggga gcagctattg cctccgtggt
112381 cacggtgtgg ggacgggaga ccgagggtct tgtccatgtc ccagtcccct gagaggttct
112441 ggctctgctc agacaccgag tccctggggc tgctgtacca ccaccaggca ggagagggtg
112501 ccagagacgt gtggaggatt taaaaacgga aggatcttag gtgcagacag gtgtctgatg
112561 ggagtcaaca gcgtgtctca actcgctatt ataaaaagag caagcccaga ctagaaggaa
112621 gccgtcttaa cctgtttagg aacatctgcc cagaaactac agcacacatc gaacccctcc
112681 gcttgctggg acttgagaat cactcccaca gaagccagcg gacatgggag atgttggcga
112741 tggctgtgcc ttccctctat gggggtgctg gaggcttcac caagtccgtg agacaagagc
112801 aagaagaaat gaagaccagc attgggaaac aaaactgtcc tgatttgttt cattttaatt
112861 tattttttgt agagacaggg ttttgctatg ttgcccagac tggccttgaa ctcctgggct
112921 caagtgatct gcctgccttg acctcccaaa gtgctgggat tacaggtgta agccattgta
112981 cctggccttt atttcatata atgtgattat caagatgtaa aagccaactg gctgggcacg
113041 gtggctcacg cctgtattcc cagcattttg ggaggctgag gcaggcagat cacctgtggt
113101 tgggagttca agaccagcca ggccaacatg gtgaaacccc atctctacta aaaatactaa
113161 aattacccgg gaggtggtgg tggtgggcac ctgtaatccc aggtactcgg gaggctgagg
113221 caggagaatg gcttgaacct gggaggcgga ggttacagtg agccgagatc acaccattgc
113281 attccagcct gggtgacaga gcaagactct gtctcaaaaa caaacaaaaa caaacaaaca
113341 aacaaagaaa gaaacatgaa gatgtaaaaa ccaagagaat ttttgactct attatttgaa
113401 ctaatgagag ggttcagtaa tgttggagac aaggttgatg agggaaaaaa caatagcgta
113461 tgtttacacc agtagaaacc aagctgaaat ataatagaaa aatacttcat taacagcagc
113521 ctcaaaacct acgaagatac ctatgtagaa aacgtcagat ggaaggtgtc caggccacta
113581 taacattgca aagggcataa aagaagactt gactgatgga gacgtatgcc acgttcatga
113641 atgaaaagtg taagttttga aaaatgagat taattttaaa aagtgaaaaa cgaggtcgat
113701 tctccgcaaa cagcctttct caatcccagc tgagtttttc gtgggatttg gccggctggt
113761 ccaaaggtcc acgtggaagg ccagagagcc aagcagagct aagtgagttt cagagaagaa
113821 tcatgtggga cgcctgcctc taagacagcg tggcattggt gccgggatag gcaagtggat
113881 gcctggaaca gcccatgtgc ttacgatgat gctttgaggt gggtgggtgc tccacatcta
113941 caaggaagga gggttgttct ctgagggtgc tgggccgggc tgttccctga gtgtgctggg
114001 ctgggctgtc ccctgagtgt gctgggctgg gctgtcccct gagtgtgctg ggctgtcccc
114061 tgagtgtgct gggccggggc ttctctacat ggggaaaatt aggtgcctgt gtcacgctct
114121 aaacaaaaat aaaacaccag gtagattaaa caactcagat gtgaaaagct aaacctgaaa
114181 acatttagaa ggaaaacagg cataatattg atgacctcag ggtaggaaag ggtttcttaa
114241 gtcactaaag tcacaagcta tcaaagtaaa gagagacgca tttgaccaca ttacatttta
114301 aaaactgtgt ttgaaaaaag acaacataac taagtaaaaa tacaagccag ggagtggaag
114361 gaggtcatct ctgtgcatcc ggcgctgagg ccggagtcca ggagatctag cgaacgtcat
114421 ggtcagacaa gcagcccagg gttgaggcgg gggtcacaca ggaggagacg cggacaggcg
114481 ggggtcacac aggaggaaac acggacaaca cagggagtag ccagcaggga ggctcggcca
114541 ctgggacccc aggaagggcg tgataatcct tgagaccagc tgaaggttct cacacctcca
114601 gagagaacat gctggggcgt ttaggggaga gcggttggtc agggcagcca agctgaggac
114661 acagtcctgc ttctgggcac ccagggagct gcccacccgg gaggatggag actggaaatg

114721 gcctgcatgc tggtccacca ggaacgggct gctcctctgg gcatattcat cctctggatg
114781 gggcccagca ctaaaccgga actgcctgtc ttgacaagga ggcatcgcaa acccagcatc
114841 gagcagaagg aatgaatcgc gggtctctaa tcctgcaccc tgatgctgtc cgtttctgtg
114901 aatgtgaaac aggaccctaa tgctggacac ttacaaaagt agtaaaatta taaaatattt
114961 tgagaggcac tggattcagg gtcacggttg cctctggaga gggttggaga gcagggaggg
115021 gcacccactt ctgcccgtga tgtttattcc ttgatggaaa acacctgagc tagagtggca
115081 ggtctgatat tagttacagc tgacggtgag gaggtagtgg tcatgtccat tccattactc
115141 tgttcttcat agggtttgaa atcatccata acaaaaaagt cagaggggcc gggcacggtg
115201 gctcatgcct gtaatcccag cactttggga ggccgaagcg ggtggatcac ctgaggccag
115261 gggttcgaga ccagcctggc caacatggca aaaccccatc tctactaaaa atataaaaat
115321 taattagctg ggcatggtgg cacatgcctg taatcccagc tgctcgggag gctgaggcag
115381 aaggatcact tgaacccagg aggcggaggc tgcagtgagc tgagattgcg ccactgctct
115441 ccagctttca tgacagactt tgtctccaaa aaaaaaaaaa agaaaaaaga aagatttagc
115501 actattcatt catttgagga aaaaagtggc cggcgctgtg gctcacgcct gtgatcccag
115561 cactttggga ggctgaggtg ggtagatcat gaggtcagac catgaggtgg gatcagaaga
115621 ccaatacccc agggttggct ccctaagacc agcctaaact ggcagcccag ggctgagggc
115681 acggccagag ctggggccct cgcccaccca ccacccacct gcatggggag gggccttggg
115741 gtgactgagc gtgagagggg ctgtgcggag gtggctgtga tggtctctgg tcccgcctct
115801 ggggcaggtg gtgctgacag ggcaggattt gggcagcagg agtcaggggc tcccagggaa
115861 gcctggagag gctgagcatg gggttggacc tcaacaggca cttttgggaa atcttgttgg
115921 gggctggggc tggtggagaa gagttggggt cctcgtggga gggcaaggca ggcctgctgg
115981 ggctgggtag ggctggggag ggatgagaag gaagaagtgt ttccttttgt ttttttaaat
116041 tatactttaa gttttagggt acatgtgcac aatgtgcagg tttgttacct atgtataatg
116101 tgccatgttg gtgtgctgca cccattaact cgtcatttgt attaggtatt tctcctaatg
116161 ctatccctcc cccctccccc accccacaac aggccctggt gtgtgatgtt ccccttcctg
116221 tgtccatgtg ttctcattgt tcagttccca cctatgagtg agaacatgtg gtgtttggtt
116281 ttttcgtcct tgcgatagtt tgctgagaat gatggtttcc agcttcatcc atgtccctac
116341 aaaggacatg aactcatcat ttttttatgg ctgcatagta ttccatggtg tatatgtgcc
116401 acgttttctt aatccagtct atcattgttg gacatttggg ttggttccaa gaagaagtgt
116461 ttctagaagg atcttccgga ggctgagaga aggagggagt ggggaggctg gtgggaactg
116521 ggagggcaag ggaaggtgag gcgtgggggc cagagaccct ggaaggtctc cccaaagtgg
116581 acagagtatc tggagaccca gaaggtggag aatacggtgg ggcccggtgg gcttcagagg
116641 gcctgggagc tgggtagtag gggcccccct cgggcactcg agaaagaccc ctccatgctg
116701 agctctagcc atttattcca gtccagaggt acgggtctta gagcacccga ggccactcct
116761 gtccccaggt cactgtggct cctgaccagc cctcaggagc cgaagggcgg gacccctccc
116821 cgtaccccat gttgggtgct gaccagccac tgtgtgcgtc attaggtagg gctgaattaa
116881 aaccccataa atctcataaa taaataaggt ggctgggcca gccctgtgtg caaacactca
116941 gcatcggtgt ggctgcaccc gttgctgggg gagcccggga ccccccctccc ctcctcgccc
117001 ttgtccctgg tcccttgctc tcctccctcc tccggaattc tagctctttc tctccaaacc
117061 aggcggtgac acctctgcgc tcccagtgtc cttttgaggc tgaaggggc ggcgtcccac
117121 agggcagggg cctggccttg tctgggtctg ggggctcagg actctggggg ctgttgcatc
117181 tctgcttgga cccagagtgg ggtctgtgcc accctggatg gcggctcaca gagggcaggg
117241 ctgtggggga cagaacttgg gccgtggaga ggcctcaaga ggaggtgagc ttggggcatc
117301 tcctgggccc attacagagc atgaggttgt gtgctggggg cggccggggc tgagactgca
117361 ccgtggggca gccagggctg agtccatcac agagcatgag gttgggctgt gggggaggcc
117421 gggggcggcc cgggctgaga ctatgcggtg gagtgggctg aggctgcacc gtgctcaggg
117481 ctggtttaag ctctgggtcc ctgtcttgga ttccgtcatc tttggagggg cctcctttcc
117541 agtttgcagg gggccccatg ggttctgctc tgcaggggct gtgggaatgg taggtgcccg
117601 ccctgagcct ggctcggctg tgattgaatt tgttgtgtgg cagccgtgtg ggttacatga
117661 agggagaggc tgaggacccc aactcgagga aattgcgatc tacggcatct atgccagatt
117721 tcaagtcagc gtgtgaagag ggtccggggt gtccgaggga ccaactgcaa accctcccca
117781 cagggaccct ttggtgccca gatgggaccc tgggcctgtt tccaccggcc tggggctgca
117841 gcctgggcat ggccaggggc agagcatcag gaagccctgg gagatagagg gcttgcccag
117901 gatggggtgc agtggtggat cacagccccc tgaagcctcc acctgctggg ctcaagtcac
117961 cctcccatct cagcctccca aatctccagg attacaggca tgagccactg cacctggcca
118021 tgctgcattt ttcaatagag cagaaatgtt tttctcagca gcaaaccaca tttccagtgc
118081 aacgggctcc tgcaatctcg ggccactgc cagccccgag gcactgaccc tctgccctgc
118141 gtgtgctgag gagaacgtgt gtccctcgcc tgaccctgca gagcccacat cctgcaggac

118201 cccaggtcca gaggacccac gctctgccac tcccacccct gtcccggccc ctcagagccc
118261 ccccatcact gtggtgcttg ctgctcttgg aaaagtgagg agggtgcaga cggcaggggc
118321 catgttctcc tgggaggccc ctcttggctt ctgctggtct ggctggaggc aggggctgtt
118381 tggacccgga ccccaccgct gacaccgcaa tttgaccctg accctttctt gacccttcta
118441 tggctcctgg cattcttgga ggggcccagg cagtgcccgg attcctcaca gaccaggctg
118501 accagagcct tctgctaggc acacccgcaa acctttaacc tcgctgtaga gccctggacc
118561 ctcctggcct ctcgctgcct caccctcact cctgctcctg ctcaggcgtc tggggacaga
118621 gcatgtcctt gagggtgtgg aggccagcaa gggctgcact gccctcccca tgggctctcg
118681 acatgggagg ggcccgggag acttggaagg gggtggcctg cagggatggg cagccctggg
118741 gcggtgggag ggtggcgtgt ggctggccac ctgccaggag tgctgattgc ggcgggtggt
118801 aaactgcggg tgcccctttg cggtgcccgg atacctggcg gggagccctg gggctgccc
118861 atgaaccagt gcccactttc ctgcccctgg cacgggctcg gcatgggacc cagtgcccag
118921 tgcccactgc ccattgcacg ccccctcct gccccaacgg ccggcagcac tagtggcctg
118981 cttggcctca cctcgctgtg cagacggggg gtgcctcagg gctgcccaaa aatcctgagt
119041 ctgtggctca gtggaatctc caaaccacag aggccaaaga gggcggggcc ccaggaggat
119101 gagccatgtt gtcaggaggg gccagtgcac agaggatggc agccccctgg tccctgtcac
119161 ggagtccgcg cccctgcctg tgctgtcact gcctgcttga gtcccggaga gccattcaca
119221 tccgtgcctt gcacccgtgt cctccacacc caggccgggt cctggtgcac cccggggtcc
119281 cggagagctg ttcacatccg tgccttgcac ccgtgtcctc cactcccagg ccaggtcctc
119341 gtgtacccca gggttggagg gacaaggaag aaggcgttca cactgtgacc tcccctgggg
119401 ctgccagcac cggagggtct taggcctggc ttgaggacgg ccccatcccc accccgtcct
119461 cctgggcctt gccagcatca ctgggtggtc gtggtgcccc actcctgtgt gggcccagaa
119521 gtgacctacg agtcccactc agcgcggcac cctggcagcc tggcatggaa ggttgtgggt
119581 caggagtgtc ccctgctgcc ctgggagccc ccacccgccc aggctggtgg cggctgctca
119641 ggggtgacct gcaggcagga caagagccga ggctgcaggg cagcctgtgt gttgtctttg
119701 agagtcggtt agagccgccc cagctgcccc agcctcctct acctccctct tggcccaggc
119761 agcagcctcg gctgcagaga cgtctggtca acccgaggag ggcctgggat gtggccttgg
119821 ttgagtcaat gtttccctct ctcccccgag gggctggcct ctcctccctg gggcgtgttc
119881 ccatgctcca gtccagatga gccgtgtctg ttttcccagg gagacgagag aatatctttt
119941 ccttttcttt gaataaatgt gcccattgct ggcacctgtg ccaagcggtt cccaggcccg
120001 agaaccctgc caggctgtgg tcctgaccgc cccagggaac ctggaagtgc ctgcacctgg
120061 agctgggaga gatcctggat tagcccccga gggcaggcac gcaatgccgg ggtcagccag
120121 gggaagcagc tgtccacagc tccacaggct gcatgccaac cacggctgca tggctgggac
120181 cttctcaccc gccagggcct ctccaccaag cagcgcctgc ctggccaccc ctggacagcc
120241 cctgggttgg tcagcgggct ccaggcaggc cagcagggtc tgcccacacc acccagtgag
120301 cgctcttgga ggggatgggg ggcctggcac acagtcaggc tcaatgaaca tttggagaaa
120361 gaaggaatga agggtccccc cccacaggca gctggatggg ggtccgctgt ggcccagcac
120421 catgtgccca gccctctccc cctggccacc gtgaggccac ctcttctgtg gtggatggcg
120481 gcctgggctg tgagccctgc gtcagccgtg gacccaccct ccctctcttt ccttcctctc
120541 tctctatctc tctccatcat ttctctccct ccctctttcc ttctcttgct ttctttctct
120601 tttcccttct atctcttttt tctttttctc tcccttgtac cctccctctt ccctccttcc
120661 tcctgattcc ctccttcctc cctcttccct ctctctctcc ctccttcttc cttcccctc
120721 ccccttcctt cctccctccg tctttccttc cttccctcct ttttctctct ccctccctct
120781 gcctcctctg tttctctcac tccctctgcc tcctctctct ctctctcttc ctcctctctc
120841 tgtctctctc tccctctgcc tcttgtctct gtctctttcc ctccttctgc ctcctctctc
120901 tgtctctctc cctccctctg cctcctctct ctgtctctcc ttccattagt acatcatatg
120961 ggatgcaatg ctggctgaag gcatgtggga caattttta gactgtcctt tttctcaatc
121021 cctccataac attcaggcca cagaggcttt ggtcctgtcc ttgctgtgtc cagcatcacc
121081 tgcagtaaaa tccagcctca gtcccccttt aggacatgcg gggacagagc tccgtggaac
121141 cccagcatga catgctgttc acctctttaa cgctgcacct tttcatactg ttaaaatggc
121201 aatcagattt ccaatgtatg aactttgagg aatatattca caccataaca tgcctctctg
121261 tcctttgggc cttctcagac ctgttctgtc cacctagtgc aggtgctgtc agcttcagcc
121321 tcctcctcag agaccagaca tgtcctacaa tctggggtcc cttgtctggc cagagggagg
121381 gacgtcagga tgggacaggt actccaagag ttctgactta aacagcccag gaggtggttc
121441 cttggggtgt ggaaagagag caggggtggt tcaggagata gcccgtggct attgttcatg
121501 tggggtctga attcaggctg ctcacatctg gttctgccca agacttagct tctcaacatc
121561 tgcaccagga atagaggacc cacctgcccc agggagagat gactgacctg ccccagggcc
121621 agaggatgca cctgcctgag gacagagggg tgtgagtcct gccctctgct cataggcaac

121681 cctggagcat gggctcaagc cttctgagac ttggtggggg catgggattt actcccaggc
121741 agggctcacc tggaccaggg ccctccactg cccctggtgc tgaactcaac cccacccctg
121801 aatactgccg tggggttgtc tatgaacatc tcccccagct ctcagtgagc tccctgaggg
121861 caagacctgt tccagcccca tgaaggcccc atacatgttg tggaatgaat aaatatgagt
121921 gaatgagtaa gtgaatatga atatcaataa tacagttgaa catgtatgaa tgaatgaaga
121981 gaaatatgtg aatgtagatg gatgggaatg aatggatggg aatgagtgga tgagtgagtg
122041 aacaggaatg agtggatggg aatgagtgga tgggactgaa tggatgggga taagtgaata
122101 aggatgaggg gatgtgaatg tagatggatg ggaatgagca catgaatgag tgaacaggaa
122161 tgcatggatg ggaatgagtg gatgggaatg tagatggatg ggaatgtaga tggatgggaa
122221 cgagtggatg ggaatgagtt gatgggaatg agtggatggg aacgagtgga tggaaacgag
122281 tgaatgggaa tgtagatgga tgggaatgag tggatgggaa cgagtgaatg ggaatgtaga
122341 tggatgggaa cgagtggatg ggaacaagtg aatgggaatg tagatggatg ggaatgagtg
122401 aatgggaatg agtggatgga aatgtagatg gatgggaatg agtggatggg aatatagatg
122461 gataggaatg agtggatggg aatgtagatg gatgggaatg agtggatggg aatatagatg
122521 gatgggaatg agcggatggg aatgtagatg gataggaatg tagacggatg ggaatgagtg
122581 gatgggaatg agtggatggg aatgtagatg gttgggaatg agtggatggg aatgtagatg
122641 gatgggaatg agtggatggg aatgagtgat gggaatgtag atggttggaa tggatgggaa
122701 tgatggatgg tgtgatggat gggaatgtat ggaatgagtg gatgggaatg agtggatggg
122761 aatgagtggg tgggaatgta gatggatggg aatgagtggg tggaaatgta gatggatggg
122821 aatgagtgga tggaatgagt ggatggtgat ggatgggaat gagtggatgg gaatgcggat
122881 gggaatgtag gtggatggga atgagtggat gggaatgtgt ggatgggaat gtagatggat
122941 gggatggatg tggatgggaa tgtagatggg aatgagtgga tgggaatgag tggatggatg
123001 gatgaataga tgggaatgag tggatgggaa tgagtggatg gatggatgaa atgaatagtg
123061 ggaatgagtg gatgggaatg tagatgattg ggaatgaata gatgggaatg agtgatggga
123121 atgtagatgg atggaatgag tgatggggat ggggatgtag atgatggatg ggaatgagcg
123181 gatgggaatg tagatggatg ggaatgagat ggatgggaat gagtggatgg gaatgagtgg
123241 atgggaatga gcggatgcat gaatgaatga agatgactca atgaatgaac aaacaaacaa
123301 cagtctccca cagtggcctg tggtgtgagc cctgctgcca gccatggaaa ctgaggcaga
123361 gaggctcctg gctcagccct gcccagcctg tccagcccct gtgatgttgg accctccggt
123421 gcagacccac cagtcatctg cagtgacggg tggcagtgga cgtgggctga gtcgcggcgg
123481 actgagtcct ggtggccact gcttgatttc attgctgggc agtgctcccg tgcagggtgt
123541 atttgtccac tccccccttgg ggtgcagggg ctgtgccttc ggtgcctgtg gcttcagtgc
123601 ctgtgcccgg gagagggctc attccccatg ttggctcctg tgggcccctg agccctagta
123661 ttcagttcat tggctgctgt gtgtctaccg gggccgccat aatgaatgcc cacaacaggt
123721 ggcttgaaaa catgggaaat gtatcctctc accgttcagg agggcagaag tcccagttct
123781 gggaggcaga agtgttgatt cccactggag gcttggagta cctctgacac acatacccgg
123841 cctttcccta tggggacaca catggatggt ggacgacacc acccagatcg tccaagaaga
123901 tttcatctga gatccacatt ataccttctg ccagcaaggc agccctccca ggtcccaggg
123961 ttcagggttt gggcttatct tttgggggcc accctaatcc tcctatagtc ccagaggcct
124021 gatgggactc acagctcagc ctaaatggtc ccctgggaca gatggacggg cagtccagca
124081 gccaggcagg atgggaggga ggtccacccg tgctggagag ctcggacccc tgggccagcc
124141 agggcctgtg cgaggcccat gcacagacag gagctgcttg ggaagtcgtg gtcctaaccc
124201 ctgatgctga cacgcaacgt ctccagatgc cctggtggtg gggagacatc agtccctccc
124261 agctggtcag cctgcggact actgagctgg acaccgtgag gagccatctg actcgacgga
124321 ggaagccctc atgggggcgt gggatgctct ggggcctctg cactgccccc accccccgc
124381 accctgaaaa cgacgtctgt gtgtggtgca gctagcgtgg accatgtccc acggagggcc
124441 cacccctacc aggtccgtct tctggcatgg ccatggctgg gccttccgcc tcacccgctc
124501 tgggagggag ccctggcctg cgctggaaca ctcggcctcc tccgtgaggg ggtttgtgct
124561 cggacaacat gaaggcagac ggatgcctag cacagctggc ttggtaaaca gagaatgtgc
124621 cgtttactcc cacatccgca gagtaaatcc ccacgggcag tgcgccgggt gctggggacc
124681 ggcagtctcc actttgggga gaggcagagc aacaacgcct gaccccagga gtttggagct
124741 gcaccctggt ctgtgtgcgt aaggctcctt ggctgcaccc cacgctgggt gagggcactg
124801 ggctggagat ggggcaggtg ggcgactgtc ccggtggccc tccagcgcgg tccccaaacc
124861 gggcctttcc caccacccgg ggaggacgct gcttatggcc aggcatcttg ggcagcccca
124921 gtgcacaaag tcagggctcc tggtggcccg ggcaggctct gtgcatggtg ggaccacctt
124981 agccttgcct cttgcaaagc agcgcccagc ccccaccctg ccctcttgtt ggcttttctg
125041 ggaggcctgt ggggggcctt tccagtgccc tggtgccgcc cctgagctct gggtcactgc
125101 ccagaggcgt ctgaagcaga cacctgcccc tgcctccctg agcccacgct acccgctctg

125161 tatcctatct agaactttca cgagccgaca ttgtcctgtg tgccagctca ctgtccccac
125221 ggaacagcca tgccacgagg gtggggtccc ttttgtttgg ggggcagagc tcaagctttt
125281 ggatctgctt caagcccaga gccacccgtc agctgaaata ctgaaaggag gaaggggtga
125341 gaggaggaag gactacgggg tggacagggc tctcagcagg cacccctgcc ccacttcaca
125401 gcagccctgc ggccggggac ggactttggc agggctggtg gcagctgggt caatctgggc
125461 ttttgggagg ctctggttct gtggaccctc tagggcttgt ccccccgcct gtgacctgag
125521 gtctcctgag ctctgcatct cagtctgctc tcgagttaaa tggggacagt atcacatgct
125581 acagaaggcc tgtggcaaaa cgctgtctgt agagacccca gcacagctgc agctcagaaa
125641 cagccaccgt tgtgaagaag atgcgtaaga gtcagttgat catgactact agcctttcct
125701 tgaagcctgt gtgtacaggt aaaactgctg tgtagcacac acacacacac acacacacac
125761 acacacacac acacacccac gaaaagcacg cagccaactg agcccaggtc tgacgctgtc
125821 tgtcctgact ctaggacaca cccgggttcc tgacattcac agtgaacctg tgagctctgg
125881 agccaacgag agacgttatt ggccaacgca ctgccctggg gccgggtcgc tggggggacc
125941 caggtaggag gcagggctgg gggagcagaa aggcagggat ggggatggac gtggctgatg
126001 ctgtgacctc agccaggaca gtccccgagt ggaggaggag gctgggcccc cctaaggccg
126061 ctcttcccca gcctcctgcc ccccggccca ggtcttatca ccctcaccca gccccggcct
126121 cccctctcga cccccaatct gttcccacct ccgttggagg aggctcctga ggtgggtgcc
126181 cctctagcct gcctgcccct gtccaggccc tgagggctcc agaggggggc cgaggcctgg
126241 gggtcccgac ctccatgtgc ctcccgtgtc cccgctgca gccccacacg caccccactg
126301 tccagccaca caggccgtct gaggctgggg gcccagaggc agactgcccc agatatgtgg
126361 gcaggggtca gcggggctct caggacacca gctgggggtg aggggagcat ggagggagcg
126421 tgggacaggc tgagctgccg agggagccac cctggagctc tggagccggg aggccttggg
126481 agacatccca ggctgggttc tgtcccccga tgtggcaggc gttgcacgca ggctcgctgt
126541 ggccttgggc gaggtggctt tctggggagg gcagttccca gggaaggagg gccgagccct
126601 cggcagccaa cactcgggtg gcttgggacg aaggcctcgg tcctggtggg gcctgggcga
126661 cgccccccagt gcccaccacg caggcctatg gttggtcacg gaggtggcct tctcctccta
126721 cccactgtct ctacctcagc ttccagcagg tgcccagtgc tcacgccggc ctcgagcctg
126781 gactgggggc tgcacctgcc ccaacactct caatgtgtgg ctggggagtc tgaggcccag
126841 aggcacccag cccatccccc cacagagcag gcctcagacc tgctgccctg ctgtctggcc
126901 gagagcacca cccggggtgg agcggccggt gagctgagcc cgggtcataa gccaggcagg
126961 ggagcaggct tcctgggatc cgccgacccc gtccagctgc tgcagggccc catgcctgcc
127021 acaggatgcc cctgagcctg gggcatggct gttgccctgc aggggaggcc atctactggt
127081 gcagctgtcc tgcagctgga catggctttg agatgacctc tgcagggtgg ggtgcaggga
127141 aaggccatcg aaacctgaga cccaacacag gggaaggtgg ggaaggctgc ggggcccagc
127201 tgcacagcgg gctgggacgc agagccagcc tcccaccctg ggcctgtccc ctgatctccg
127261 cccagtgtgg actccttttg ggcacagctg agaccaaccc agcagataag cggattagca
127321 tggtgcatgc ccccagggtg cacaactggc tttatcaggg cccggatgtt gtctcaggtg
127381 gagctgggca ggcttaggtg aggtgggtgt tcccaggtca gatacgctct gcccagctct
127441 caccccctcag ccggcgcagg gccaggggcc tggatcaggg gttcgctcca gctgcctgct
127501 atctggctgc ccgtggcggg ctcggggcaa gggtgtgcag gagagccggg tcttggtggc
127561 tttgtgctcc taccctgggt ttcaaaggcg ctgcctccct tgttccttgt tcattcacaa
127621 aatccgtgag cgccccagca tgtgggacac agatgaaggg gccgggggag ggttgccact
127681 gggccagggc accgggagtg tccccacgtg ccattcggcc gagtccttgg caggggggaag
127741 ccctgctcag aaacggagaa actgagtctt gggacatttg ctgtctcgct cactgtcaga
127801 gccccctcca tcccaagaca ggcctgggca cctgacaacc gtgcaggacc gtgacataaa
127861 atatgccagg aaatcgggtg gaggaaactc agacgtgagg gaatcaggct ggcagagccg
127921 ggtggggaga gggtgctggc acaggcaggg acgctctagg tgctggtttt aggggcaggg
127981 ctgcctggga agccacctga gatgagaggg agagaggcca gggggacttg aagaagccca
128041 gggtgctggg agaacctggg ggccaagcag aggaggtgcc tgggggccag gccaggtggg
128101 cacagaccca cagctacccg ggccctcctc atctgcccca ccccagagcc cacctgcatc
128161 cgccacagct ggcctggctt tctgcccgac acctgccagg tcaggggggcc cctgctggaa
128221 gccacacccc actctgccca cctcccacct cctcactccc cctgcctgag ctcctgccag
128281 ccctcacggc gccctcctaa cctccactca tcagagccct gccctgtgcc ccagactcgg
128341 ctggaggcac ccccaggaag cccctgtggg aagcaagtcc tggtgtcggt cctcacccca
128401 cccctcaggg agccacaggc ccagctcctg ggtgtgtccc tctgggtggg ggcctggggt
128461 cacaggctgg cctggagcca tggagacagg ggagctgcct ggctgtgggg gaaaggcaaa
128521 caccctttttg tctgtggccg cctcttctgc tcggggaact ctggggacaa agccggtggg
128581 ccaaggagcc cccgctgtct agacagccct gccccacgag gcaggcaggg gagggggcgc

128641 cctgagcaaa caagcaggta ccaggcgtcc tgagactgcg gggccaggcc agggtgggcg
128701 agcgacgagg cccgcgggag tgggaggggg cgcgggatgc tgtccttagg gaccctgggc
128761 cccagccggg gtttctccct ggtctctctc cttcctttct gctggccacc acctccagga
128821 agccagctgg acctgaccct gcctcctgag ggctctagga acccacctcc caggtccagc
128881 catgcaccag cctgtcctcg tgaccctcac tggcctcagg gatcccccag taagggggca
128941 gcggtgggga ctcacctgtg gggcttgagt gagccatgcc caagacaaga ggcagagcca
129001 ggaggtgcag gtgacgctat ggtccccagc tgcgtggcgg ctctctccag gtgtgtgggc
129061 ctcctggtcc cttcgtggtg tctggtgtca ccatgcacag ctcagacccc tgctgcagtc
129121 cccagggatc cacagccgca cctgcccttt cctcttgctt ctcactgcgc cccagccccc
129181 tggcggctgc tcaccagcct tcatgtcctc actgacgcct gccctgcacc gggaccaccc
129241 tgtgttcccc agctgtgccc agacctgatg gaaattctca gcagcccagg ccagacccac
129301 cttcccgctc tcctgccagc tctgaacgtt gggcccctgg cactgttatt cacatcttac
129361 tccatcagtt cccagcccag ccccagcccc tgccccttca gatgcaaacc ctgtgagggc
129421 cagagcctcc ctggtgtcct caccttccca cccatacact cagctctcca tttattcagg
129481 attaattcag ctgtttgggg aataaagatg tgaaaaagtt accatcttta acaatcccct
129541 tgactgactt cagaatagtg ttccctttca tcccctctgt ccacctggga atgcttctga
129601 tgctgcctgc attatcagga ggggaagagg cccagccagt ctgcagtgaa catgccgtga
129661 aagcctggac tcaacatttg ctatttgaag ttacagacag acttttaagt acctgactgg
129721 atataccatg tgaacatgcc gtgaaagcct ggactcaaca tttgctattt gaagttacag
129781 acagactttt aagtacctga ctgtatatac catccaacct gggacccggg gatgggagag
129841 cccacgcccc tgactggaga gttccagagg cctggaaaga acacccacag tagaaaaccc
129901 gcggaagagg ggcctagcaa aagattcatt aggtattatt attattattt tgagatggag
129961 tctcgctctg ttgcccaggc tggagtgcag tggcttgatc tcagctcact gcaagctcta
130021 cctcccgagt tcacgccatt cgcctgcctc agcctcctga gtagctggga ctacaggcgc
130081 ctgccaccac acctggctat tttttgtatt ttcttataga gataggattt tgctatgttg
130141 ccgaggctgg tctcaaactc ctggccttaa gggatctgcc caccttggcc tcccaaagtg
130201 ctgggattac aggcatgagc caccatgcct ggccttcatt agatattatt attattattt
130261 ttttaaaaag tttctcctga aaggcagaga gaacacatag ttttatttaa aaaatacatt
130321 agaaacaagt atatttagaa aatatatgct tttattattt atgaatttaa aaaaacgact
130381 caatgaaaca aaaagtgaaa gacgaataaa aaaggtaaca gactgagagg agagaatcag
130441 cctgaggcag agatgagggg gaactagata atgtaaagaa accacgatgg gagagttaat
130501 atctgtactc aaaacagtaa aacggcaagt aacaaaaaac accatgtaaa attgagtaag
130561 taatgtggaa gacaattaaa gttctttgag aataggaggg aaagaacaga gagattaaac
130621 tggtgtgaaa ggctccttca gtgtttgggg actagctcat atcagaataa catgcttgcc
130681 cttcataatc ataaactcaa gatgaaatag aaatgacacc tgttttgaag actggtgagt
130741 ggctgggcat ggtggctcat tcatgtaatc ccagcacttt gggaggctga ggcaggtgga
130801 tcacctgagg tcaggagttc gagaccagcc tggccaacat ggtgaaaccc catctgtact
130861 aaaaatacaa aaattagctg ggtgtggtgg cgcacacctg tagtcccagc tactagggag
130921 gctgaggcac cagagtcaca tgaacccagg aggcagaggt tgcagtgagc tgagatcgca
130981 ccactgcact ccaatctgga caacagagtg agcctcagtc acacatacat acacacacac
131041 acacacacac acacaaccca aaacaacaaa aaaccactgg tgagaaatca gaggcaggca
131101 gcatcagagg ggatgcaagc ctggaaaagg gaaataagtg aggtacgatc cccattcagc
131161 tgactttcca ctggagggag cttccctgtt tacaggaatg tgagggagag ggtgccagct
131221 gactacagct cctgagctgg gctgagaaga ctgaggtcca agcttaaggt ctccagaggc
131281 tggaaattag gaaggatgtt acaggaagaa tagatccaca aatttttgac tgagttaaaa
131341 gtcacatata ggatgagacc ttaaacatct gagtgaaagg aaacagctgg aaggctgaaa
131401 gataggggca tggggtcaca gagtttggag ttcgagttgg cccagctaga cctcctggag
131461 aaacaccttg cttcccatta cgactctgta agaactgtat gttaggagca aggaccatct
131521 gctaggacta aatgctgtgc cccaggaaca agggctaaac tgaaagagac ccagataata
131581 aagcctgaga agaatcctcc tcaggatcaa gttgatccac caaaaattta accacctgct
131641 aaacaaaact tagcattctc cttcggaaga tcacagaatc taaagtctcc acagtgtatc
131701 atacacaatg cacagtgtac aataaaaatt accacagagg acaatatagc ccacaatcaa
131761 aggaacaaac caaccatcag aagcatatcc acagaggccc atatgttgaa attaggagat
131821 aagtatttta aattaaccat tataaaaatg tgacataaca tacattggaa aatgaatata
131881 atggaaaaga ggtggggaat gtcaggagag agatggaaac tctaaaacag aaccaaatga
131941 aaatcttgaa actgaaaaca aacaacacaa ccaaatatct gaaatgaaat agtcattgaa
132001 ttggtttaac agaagctggg aaactacaga ggaaaagatc aagaaacttg aagagaggtt
132061 gatagaaaca atccacactg aagaacagac aaaacagttt tttgaaaaag tctcagtgac

132121 tggtgggatg atattaagtt gtctgaggaa ggagaagaaa gagaaaaggg ggtaaaaaat
132181 attaaagaaa aaatagccaa atgttttaaa atttgactaa aaacatcaac tctgaattcc
132241 aagaagcaaa tcttcagtgc aataaatatg aagaaaacta cacttacgca tgtaatagtc
132301 aaactactga aaagaaaagg taaaagaaaa tctttaaagc atccaggaag aaaaagacat
132361 agcacacaga gggaataaat gataaaatgg tagctataaa tgcagtcata tcaaaattta
132421 aattaatgta agtggactat acactccaat taaaagacag acttttagac tgaatcaatg
132481 agcaagaccc aaagatatgc tgcttacaag agacatgttg tgactataga cacagataag
132541 gttaaaagtg aaaggatgga aaaatgtatg tcatgcaagc agtaatgata ataaagctag
132601 agtggttaca tcaatatcag acaaagtaga tgtcaagaca aggaatatta ccgagataaa
132661 aggacatttt ataattatag aaatgacaat tcattgagaa agcacaatat tactgtgtat
132721 acatctaata acaaagcttc aaagtataca aagcaaaaat agacaaaact taagggtgaa
132781 acaggcacag attttaacat cattttcttg atcattgata aagcaaataa acactcagga
132841 aggctgttag aagttctgaa accactatca accaacttga gctaattgac atttatggaa
132901 ccctagtcaa tgactgtaga atacaccatt cttcaaaaga ccacatggat tgctcaccga
132961 aatggaacag tgctgggcta taaaacaagt ctcaataaac ttctaaggat tgaaataatc
133021 aacatatgtt ctctcactaa aatacaaatt agttaaaagc agtaatgata agatattaag
133081 aaaaacctca aatatttaaa aatcaagaag cacaatgcat tccaaacaac ctatgggtca
133141 aaaaagtcac ataagaatta caaccaactt ctcatagaaa caaaggtggc cagaagatgc
133201 cagaaagaca tctttatagt gttgaaagaa aaaaaaaaaa gcctgtcagt caacactaaa
133261 atttaggtct acaggaagga atgaggagca ctagaaataa caaatatgag ggtaagtaca
133321 agtaaaatgt tcttttttaa aaactttctt taagggactg tttaaagcaa aaaattaatt
133381 gtggaagttt ataacacatg gagtaattaa aaacatcaca ccaaatagca ccaacaatgg
133441 agaaaaaatg aaattagact gttcaaaggt tcatatatat acatatatat acacatatat
133501 atacatatat acatatatac acatatatac atatatacac atatacacat atatacatat
133561 atacatatat acacatatat acatatatac atatatacac atatatatac atatatacat
133621 atatatatac acatatatat atacatatat atatacatat ataatttttt ttttttttgag
133681 acagtctcac ttactctgtc acccaggctg gagtgcagtg gtgcgatctt ggctcactgc
133741 aacctccacc tcctgggttc cagcaattct cctgcctcag cctcccaagt agctgggagt
133801 acagatctgt gccaccacgc ctggctaatt tttatatttt tagtagagat ggggtttcac
133861 catgttggcc aggctggtct cgaactcctg aactcaagtg atccacatgc cttggcctcc
133921 caaagtgctg agattacagg tgtgagacac tgcacccggc caggttctta cattttacgt
133981 gatgtgatat aatattaatt caagatagac tgataaggat gcatactata actttcagag
134041 aaactactaa aaatataatg atatctagat aaaaagcaaa tagaagtatt aaaactgaat
134101 attgaaaata cttaactgac catactaagt gttggcaaga atgtggcaca gccggagctc
134161 tcagacagtg cagacaagac ataatgtggt acaatcactt tggaaggctg cttgaaagtt
134221 tcttatgaaa ttaaacacat gcttagcata ggactcagca attctactcc tagacattta
134281 ctcaaaagaa atgaaaacgt gtgcttacaa aaagactcgc acaaaaatgt tcctggcagc
134341 attattcgta actgtaaaag actgggaaag gctcaggtat tcctcaagag gtgtatggaa
134401 taaatacatt gtggtatagc catatgatgg aatactatcc agcaataaca agaaacagtg
134461 aaaacacacc acaacatggg taaatctcaa aaacatgatg tgtgaaaaag gccagacaca
134521 gaagagttaa tattttatga ctccatttgt ctgaagttcc ataatagacc aaactaatgt
134581 acagtgacaa gactcagacc catagttgcc tgggtcagga ataagagtgg ggccaggcat
134641 ggtggctcag gcctgtaatc ccagcacttt gggaggccaa ggtgggcaga tcacctgagg
134701 tcaggagttt gagaccagcc tggccaacat ggcaaaaccc catctctact aaaaatacac
134761 aaaaaagtag ccaggtgtgg tcgtgggcac ctctagtccc agctactcag gaggctgagc
134821 caggagaatc acttgaaccc aagaggtgga ggttgcagtg agccaagatg atgccactgc
134881 actccagcct gagcgacaga gcgagactcc atcttaaaaa acaaaacaaa caaaaaagag
134941 tagggttgac tggaaagggg catgaaactc tttgggtgat gggaacattt tctggcttga
135001 tcgcggtcat ggttccatgg gtgtatacgt ttgccaacac tcattgaata ccatcatccc
135061 ttggcatctg ttggggattg gttccaggac ctcctggaat acctagacag tatttgcata
135121 aaaactgtgc acaccgtcct gtgtacagtt ggcccttgaa caacatgggg gccaagagtg
135181 ccagcccctc atgcaggtga aaatccaagt ataacttttg atttccccca aacttaacta
135241 ctaatagcct cctgttggct ggaaacctta ctaacaacat aaacagttgg ttaacacata
135301 ttttgtctgt tatatgtatc atagactgta ttcttataat aaagtaagct agaggaaaga
135361 aaatgttatt atgaaaatca taaggaagag aaaatatatt gactatgtat aaagtggaag
135421 tgggtcatcc caaacgtctt catcctggtt gtcttcttgt tgagtaggct gaggaggagg
135481 agaaggagga ggaggggttg gtttcaaagg tggcagaggt ggaagagatg gaggaggtgg
135541 aaggggagtc gggagaggca ggcacacttg gtgcaacttt cgtggaaaaa actccatgca

135601 taagaggaca cacacagtta caacccctgt cattcaaggg ttgactgcac tgtaagtcat
135661 cgctggatta ctcataacac caaagacaac acccacacac cccttcattc ccgtggattc
135721 agcgtggagc tcggctgtgt gacctcaacg tgtgacctca gtggcagatt caggttttgc
135781 ttttttggaa cattgtgact ttttttttcc tgaatatttt tgatcagccg ttggttgagt
135841 caacagatgc agaagccaca gacatgcatg gctggctgta ttaacttaaa atgaattcat
135901 cttaagccag gcatggctta tgcctgtaat cccagcactt cgggataata agtgctggga
135961 ttacagcact tattataagc caggctcatg cctgtaatcc cagcacttcg ggaggctgag
136021 atgggtggat cacttgaggt cacgagttca agaccagcct ggtgaccatg gcgaaacccc
136081 gtttccaata aaaatacaaa aattagccag gcgtggtggc gcgtgcctgt agtcccagct
136141 actcaggagg ctgaggcagg agaatcactt gaacctggga ggcagaggtt gcagtgagct
136201 gagattatgc cactgcattc cagcctcatg acagagcgag actccatctc aaaaaaaaaa
136261 aaaaaaagag ttgattttat tatgggtaaa ttatatctca acaaatttta tttcaaaaga
136321 aaacaagaat atcgattcac tgaaaggaaa gcaggcaaag aagatcgaag gaacaaagaa
136381 tgcatgggtt acacataaag caaagtgcaa gacggtgggc tgaacttgcc ctacctgtaa
136441 aacagcaata ttgaagcgat gataaaataa aacatgtctg agtcaaagca agacttcatt
136501 tattcggcta gttgagagtc tctcacgtgt cattcatcaa ggtgcacaaa accagacaag
136561 atccttcttg tcagtgggat aagagtttag tgggatcata atatttcagt tgtataatct
136621 catcaatcaa tgggtaactg cagaagggaa cacatttctg tggggaaatt caatacgggg
136681 agctgagcgg ggtgtaggca ggggagatgg ccttgcacgg ggtggctggg ggaggcgggg
136741 gtggctgggg gagggagggg gtagctgggg gaggcggggg tggtgggagg aggcggggtg
136801 ctcagacaga agcagctgca gggaagccct ggggtggggg aacaggccct ggaaaactgc
136861 taaggcaatc aattagaaag aatatttccc atgcattttt cctgcaaaca gaaaggactg
136921 gaagacatac atcagcccat caagatacaa tctcagcaaa aaaccacaaa agcggcagac
136981 aaaaccaaac ctcaagaaag gcaaagttgt acattcagaa ggtctggaat gactgggttt
137041 aggggtcatc ccggccattt gctgcaatgg ctgccacaga ttgcaaagag cacagtcaat
137101 tcttggagga ggacaagggg aggtgtcatg tttacaatcc agagtgcaca gcccagaact
137161 gagacctgga aaggaggggc cagcaggagg aagggaaggg gtagtgacgt ctcttccaca
137221 cgcacaagca aatcagggtc actcatagaa acacagatcc cagggttaaa aggatgacct
137281 ctatagtcat gaaaacagag tgaagacctg caagaggaaa cggcaaaggg agcacaaacc
137341 cggctgcaca agaacagacc ggagaaaggt caggactctc aggccaggga ctgaggagcc
137401 agcgataagc catggcagta aaggctgact gaatgcccct atgaaaagga ggctggagat
137461 ggggctgaaa agaaaagagc acgcccacag cctcactgca cgttgtttga aagagacaca
137521 gctaaaacga atgccccagc aaagttaaaa tcgaaaggca acatggccgg gcacagtggc
137581 tcacgcctgt aattccagca ctttgggagg ccaaggtggg tttgaggtca ggagtttgcg
137641 accagcccgg ccaacatggt gaaaccccgt ctctactaaa aatacaagaa ttagccgggc
137701 gtggtggcgc gtgcctataa tcccagctac tcgggcagga gaattgcttg aacctgggag
137761 gtggaggttg cagtgagctg agattgcagt gagctgagtg ccactgcact ccagcctggg
137821 tgacaagagc acaactccat caacaacaac aacaacaaaa agcaacagga tatgagacaa
137881 aagccagcag aggcagagct gtggccgtag tattaataaa tcgaaaggcg gggttaagat
137941 atgaacaccg aacacgacaa agaggatcag tttgagttta tagaagaagc agaagaaaca
138001 atggtgattt attttgattt ttactcatta aaaaacatag tgctgctgct cccagatgat
138061 ggcatagctc ttcttttccc tgtcttcctc ctaagtacaa agaagaatcc tgaatattag
138121 acatgaaaga aatgtaggaa ggctccggaa ggtggagagg agagggcaga ccagtcgggg
138181 acctcgggcc ccaggaaaga cccattgcaa gttccctggg ttttgctttt gcctcatgga
138241 tcccaggcct ggagctggag aagctggcag tgtggacact tcaacacatg tacaccaaaa
138301 aaaggcccca ccagtccccg cctcctggag ccagcgtgat caggaaagta gctgcccggc
138361 aaagcagaag gcttttaggt gataactgcc ctgctccagc tgaacaccac ggaaaacctg
138421 cactccactg gagcaaagtc tgggcagggc tgggcagggc tgggcaccca ccctggtggc
138481 tggaatgagg cgccccaggt ggccccaggg ctggtgtcca agtgggcaca gcaggaggct
138541 ggggtcatcc cagtgggtgg ttctgaaccg ctttctctgt gaccagtgat ggggatggtg
138601 gagaccacac ggggttaggg agcttggatt tccaccccat ccctggtgtt gaaacactcc
138661 tcctcttccc catgaggagt gtcagaggag gctgagccga gagtcatgac tttgaccact
138721 gctcagtgta acagggcccc ctccatcatg cccgtggagg gaatgagagc agcaacgagg
138781 cgccatgccc ctcccagcca gaaggcatcg gtgagggctc caccctgccc ggcaggcatg
138841 gacaaaaccc ctggggtgtt aagagagggc cggtggggaa tgagcacttc cacaccacaa
138901 gctgcccctt cccttcatta atctgtgcca gaggaagtca acgagaaagg tttaaataag
138961 atccagagtc tcgtagcaat ctccaaaatg cccaggtttc aataacaaat tgctcatcat
139021 cccaagaccc aggaagatct cagactgaat tcaaaaggac aatcagcaga ggctgaccct

139081 gagacaaaga ggtgttagaa tgatctggaa agggtttttg agaagcccat aaagtgcttc
139141 atttgtgaac aaattgaaac caatttaaaa atagaaagtc tcaacagata atagaaaatc
139201 ccagcacaga aacagaacac acaaagacga actgaatgga aatggtagaa ctgaaaatta
139261 caataattga agagataaaa aacaaaaaca agacttcaat gaatgggctc aacacagagt
139321 agagggagag tccagtgaga ggaagaaaca gtgagcaggg aggtacaata atagacgtta
139381 tcagacccat cagcgaagag aaaacagact gaacaggatg agagaaacac acagagcctc
139441 aggaaccata gggctagaac aagagaccca gcattccagg tgttggggtc atgggaggag
139501 agaaagagga tggggatgga aaagagctca aagacctaag gttgaaaact ccccaaactt
139561 gccaaaacac ccacagattc aagaagctcg atgaacccct aacaggataa acccaagaaa
139621 atccacagca aaacatctcg tagtcaaact tttgaaaact ataaaaacaa aaaatttgg
139681 aagtagcagg agagaaacaa cattttacat ataagggaaa gacagtttga gtggcatttg
139741 attcctcatc agaaacccca aaggccaaaa agagcacaac atttttcaaa tgctgacaga
139801 aaggaactgt cagcccagaa tgctatattc agtgaaagta tccccgaaga aggaagagga
139861 agttaaggca ttttcagatg aaggaaaagt aagagaaatt tccaccagaa gatgtaccct
139921 gaaagcttca aaatagttga accaaatctg ataaaactga aaggagaaat gcacccattt
139981 ctaattatag ctagagacat caacacccct gtctaaacaa ttaatagaac aactaggcag
140041 aaaatcaaca aagatgtgga aaaacttaac accatcaacc aacaggacca acatttgtag
140101 agctccaccc aaccacagaa cacacattct tttcaaatgt gcacggaaca cattccagga
140161 gaggccgtgt cctgggcctg aacacaaact ttagcacatt tcacatactg acatcacact
140221 ccagcatgtt ctctgacaac aatgacatca aattagaaat gaacaacaga aagttaacag
140281 gaaaatctcc aaacattcag aaactagaga acagacctcc aaataatctg tgggccaatg
140341 aggaagtctc aagggaagtt ttaaaaaaca acaacattga actcaatgaa aacgaaaatg
140401 caacatatca aaaattgagg gacatagcca ggcatggaga catgcaactg gggtcccagc
140461 ttctcaggag gttgaggtgg gagcatcact agaggctagg agtttgaatc tagcctgggc
140521 aacatagcaa gaccctatct ctgaaaaata aaaataaaaa aaattagaga gatacagtca
140581 aagcagtgct gagggaaatt tgtagcagta acagaacaca ctagaaaaaa ggatgagtca
140641 agtcagtaat ctatgccacc cactgaagaa atgagaaaaa gaagcacagg ttgcacacag
140701 agcaaactga aggaaggaaa gagtaaagac ctcagtgaaa ccgaaagcag ggagacagtg
140761 gcagaaagaa aaataccaat aacacggaca aggctctagc attacagcaa gaaacaagag
140821 caaagacaca aatgaccaag gacaggacta aaacaggagc aatcactaca gaccctgcag
140881 atatcatgag ggtgacaagg ggctgtggca cacaactctg cacacagaac tttggcacct
140941 tagatgaaat atgccagttc ctcaaaaagc acaagtgatc acaactcagc taacacaaat
141001 agataactgg gcagcccaat aaatggttga gaacattgaa tttataattt taaactccaa
141061 aagaagaaat tccgggctca gatgagttca gcggtgaatt ctgtaaaaac tttaaagaag
141121 aattaacacc aatcatacat aattcttcca gaatgtagaa gaggagggcg tctgtatgac
141181 ccagatactg aaatcataca ggcaggagaa aaacaaagca aatccaaacc agaactatgg
141241 accaacaccc ctcatgaaga tggatacaga ctctaacaaa ataacagcaa agagaattca
141301 gtgctatatg gagcattatc catcttaacc aagagggatc tattccagaa atgcaaggtg
141361 gccaggtcaa tatttaaaaa attaatgcaa ccagccatat taacaggcca aagaagaaaa
141421 attgcacaag cctatcaatc agagcagcaa aaacatttga aaagactcaa cattcattct
141481 tataaattct caaaaaataa gaatacaggg aatttcctca atgagataga gaacagctac
141541 aaaaaaagga aaccaaaaag caaaaaccac tcctgctaac attgtaccta atggtgaagg
141601 ctgagttctt cctgtctgac actgggagca cggtgagggt gtcccctctc atcgctctta
141661 ttcaacgtac tgctgggagt tctgccagtg caataaagca agaaaaggaa ataaaaggca
141721 attcgtttgg aagaaataaa gccgtcccta tttttaagtg acatgattgt ctatatagaa
141781 aattccaaag atactgaaat ttaaaaaaat ccaagactaa tgtctgtgca gaaaggtcaa
141841 aggatacagg ataaacacac aacaatgaat agtatttcta tttagtagca atgaccatga
141901 cttcatcgac tttaaaaata taccaccatt ctcaatcacg caaataactt gaaacatgta
141961 ggcgtaaatc taaccaaacg tggaggactt gtgtgcttaa aactacaaaa tgccgatgaa
142021 agaaatcttt cttttttttc atttagaatc catttttatt cccacaaaca gttctgaaaa
142081 atattagaat tggcaaatgg tttaccatga atggaaaaaa aaaacccatt gattttcctt
142141 ttttcttttt ttttgcttaa ataattttat tatttttatt ttatttttcc ataagttact
142201 gggttgtatt tgggtatatg agtaagttct ttagtggtga tttgtgagat tttggtgcac
142261 ccattacctg agcagtatac actgcaccat attttttgtc ttttatccct caccccctcc
142321 cactcttccc cccaagtccc caaagtccat tatatcattc ttatgccttt gcatcctcat
142381 agcttagctc ccgcatatca gtgagaacat atgatgtttg gttttccatt cctgagtgac
142441 ttcacttaga ataatagtct ccaatctcat ccaggtcact gcaaaagctg ttaattcatt
142501 ccattttatg actgagtagt attccatcag atctatatct atatgtatat ctatacctat

142561 atctatatct atataccatc acagagcagg taaacctgta ggggtgcaaa acagatatgt
142621 ggtggctgga gactgggaga ggggacgggg ttggctgtac agggcacggg ggacctcgtg
142681 gggtgaccga gcgactgtct ctgagtgtgg tgatggttac ctgactgtgt gttcatcaca
142741 gctcccagaa ctgtaaccct aacaggagtg gatcttactg tatgcaaatt atatcttaat
142801 aaaaaatgaa agatatatgt aaagttgatg tcggaaaaag tgcagagaaa ttttaaaaag
142861 ttcagttata gtatcttact ccatgacaga tagagtaggc aagaggtgga gagaagattt
142921 gaattatgta attaataaga tttatttaca catttatttc aaactttgtt ctctaataca
142981 gataatactc cttcatctta ggcatccata gaatagttac aaaaattaat ctttttttaca
143041 tgtatttttt attttcaagg tataatttgc atacagtaaa attcactctt gttagtgtgt
143101 aattctgtcc aatttgttgt ctacggaatt ggctgcagtt ttctctgggg cctaatgtga
143161 gattaatttt tgtggctatg gtctctaaca aaatacaact gaaaattgat aacaaacatg
143221 tgaataagac aaaagaggct gggcacagtg gttcacgcct gtaatcccag cacttcggga
143281 ggccgaggcg ggcggatcac gaggtcagga gatcgagacc atcctggcta acacggtgaa
143341 accctgtctc tactaaaaat acaaaaaatt aggtgggcgc ggtggtgggc gcctgtagtc
143401 ccagctactc gggaggctga ggaaggagaa tggcatgaac tcgggaggca gaggttgcag
143461 tgagccgaga tcgggccact gcactccagc ctgggcgaca gagcgagact ccgtctcaaa
143521 aaaaaaaaaa aaaaaaaaga caaacagcct aagcggctgg gggtggacac ggcagggctg
143581 tctctggcct ctttcctgtc tctggcgtct ctcctctccc ctcccctgga ccctccttat
143641 tctgcaaaac gggccatggg cactttctgg gaaacgtcac tttgctcaaa ccgtgtttgc
143701 agatccagga ccccttaaga gatgttactt tgcataaagt gtttgccact taggttttcc
143761 ctaagggatg ttatgtgttt aagctgccat taaggtgcag agtggtaaac ctgttacagt
143821 cacgtgtggt ccgcaattgc caccaagacc atcctggctg tgtggagccg ggctgctgcc
143881 gtggcaaagg atcacagccc tgtggctgag cacagcacgc tcacgccgtg cggtccgggg
143941 gtgccaagtc tccagggagc tcgcaggact gcgttccttc cagaggctcc aggggagggt
144001 tcacgtcctc gcctcttccc gctctggagg ccgcgtctct tccccggcgg accgtcctct
144061 acctgcagag ccatcgggag cctctgctct ccccgactgc ctccctctcc tcccgctgtc
144121 tctgtctgcc tgacctcttt ctctgaccct cttgcttctt tcacaagagc ccttgtggtt
144181 acttcgggcc catccagata atccaggaca acctccccac ccaaggccca tcactctgtc
144241 caccaggtcc cttcccccat ggctccaggg tttagaatgc gggcatctct ggggccatca
144301 ttcaggctaa tgctgtcagt atattaaaaa ctgcatttct atatgctagc aacaaactag
144361 tagaaaagag catcaaaaaa ctgctactgc ctagaaataa atttaacaaa atgtccccat
144421 gacctctaca ctaaaaactg caaagaattg cagaaattca aaaggaccaa aatatgtgga
144481 gatgtattaa tatagttgaa gactcaatat tgttaaaatg tcattttcct ccaaactgat
144541 cggtaaattc aatcccaata aaaaatccag tttttttttgg gtggacattg acaagcgcct
144601 tctaaaattt atacggccat ggaaaagacc tgggatagcc atgacgatct tgacgaagca
144661 gaaaaaaaca tttccaagac ttagaatgaa gctcttagtc gagacggagt gacattgata
144721 taaacttaga cattcagatt aatgagacga actgagagca cagaaatagg ctgtcacctg
144781 taccgggcaa acacaccgag gaaaaccaag agtgccagtg tgtgtgtgtg tgtgtgtgtg
144841 tgtgtgtgtt cacagggtta tgtgtctgta tgtgtatgta tttgtaggct tgtgtatgtg
144901 tttctgtgtt tgtgtgtttg cagggttcta tatgtgtttg catgtgtgtg tatgcaggtt
144961 gtgagtatgt gcttgcgtgt ttgtgtgttt gaatggttgc atgtgtttgc atgtgcgtgt
145021 gtttgcaggg ttctgtgtgt gcacatctgc ttgtttgcat gtgtgtttgc agggttgtgt
145081 ctctgtttgc atgtgtgtgt gtttgtgtat gtatttgcag ggttgtgtgt gtgtctgttt
145141 gcatgtgtgt gtttgcagag ttatgtatgt gtgtttccat gtttgtgtgt ttgcaaggtt
145201 ctgtgtgtgt ttgcatgtat gtgtatgcag gtgtgtgtgg gcttgtgtgt ttgcatggtt
145261 gtgtgcgtgt ttgtgtgtgt gcttgcatgt tgtgtgtgct ttgtgtgtgt gcttgcatgg
145321 tgtgtgtgct tgatgtgtgt gtgcgcttgt gtgtgtgtgt ttgcatggta cgtgtgtgtg
145381 cttgtgtgtg tgtttgcatg gtatgtgtgt gtgcttgcat agtgtgtgtg tgcttgcatt
145441 gtgtgtgtgt gctttgtgtg tgtgcttaca tggtgtgtgt gtgctttgtg tgtgtgcttg
145501 catggtgtgt gtgtgtgttt gcatggtgtg tatgtgcgtg tgtgtgtgtt tgcatggcat
145561 gtgtgtgtgc ttgtgtgtgt gtgcttgcat ggtgtgtgtg tgcctgtgtg tgtgtgcttg
145621 cgtggtgtgt gtgtgtttgc atggcgtgtg tgtgtgcttg tgtgtgtgtg cttgcatggc
145681 gtgtgtgtgt ttgcatggtg tgtgtgtgct tgcatggcgt gtgtgcttgt gtgtgtgtgc
145741 ttgcatggtg tgtgtgtgtg tttgcatggt gtgtgtgcgc ttgtgtgtgt gtgtttgcat
145801 ggtgtgtgtg tgcttgcatg gtgtgtgtgt gcttgtgtgt gtgtgtttgc atggtgtgtg
145861 tgcttgtgtg tgtgtgtttg catggtgtgt gtgtgtttgt gtgtgtgtgt ttgcatggag
145921 tgtgtgtgtg tttgcatggt gtgtgtgtgt ttgcatggtg tgtgtgcgct tgtgtgtgtt
145981 gtttgcatgg agtgtgtgtg tgtttgcatg gtgtgtgtgt gtggactgct actgctgcca

146041 tgtcgctgca gttcaatggg tttgtgcgtt gtgatcacac ggtcctgccc acagagctga
146101 atgggctatt ggggtttcct tgggtgagtc ccaggctgtg tgtggggtga gtgtggggcc
146161 tttcttgcac cgtttagcct ggcacgcgtt gccctctctc tctctcccag gaccacattc
146221 aggtgccagg gcctttaatc ctgtgtccct tgtcctactt actccctctc cccagactgt
146281 gggacaggca cctggggcgc actgtggggg ctctgagatg caggtgggtg ggccaggcct
146341 gccgagggga ggtgaggcga gcagagccct ttgagggtga tggagcttgg ctggccccat
146401 ggcccgaacc cacctggcct aggggcgtgg ctcagagcag gtgcacacag ggcttattca
146461 cctgtgcatt cttcaaacag tccaggtgtg agtgtgaggc tgtgaacaca ccgggggtgt
146521 gtctgccagg tggggccctg gacggggtgg cccccagcac ctctgggttt ccagccccca
146581 aaagtgggtg tctctgggcc cacctgctcc tggagctggg ctcgcactga ccctgggaag
146641 acagcctccc ctgggcattt tggggacgct gggcccagcc catgcctcgt ggctcagctt
146701 ccctcagcca atactttcga gacatgtatt tcgagacatc cctggagaca tggccagtat
146761 atttgtattt tatatgaacc cggaaggctg gacctgaaga ggccccctgg gtgagggggc
146821 caggctgagc tcgagttccc cagggcctcc agcaagaaag gcacccaatg cctccttaca
146881 ggaggagcag gcgcatgtcc acagaagatc tgaaaagacc ccatcaaaat attcgtgggt
146941 gtcttctcaa gacgaggggg gcaattttcg ctttacattt tgttccaatt ttttattgtt
147001 gtaaaaaaca cataacataa agttgacgtt ttttgccact gctgggtgca cagtgcagtg
147061 ctgttaggac attcacactg ccgtgcggcc cgcagagcca ccatctccag aacatcttcg
147121 tcttgcagaa ctgaagctct gtcttgttaa acaccaggtc cccactcccc gtgcccagcc
147181 cctgcccagc cctggcggcc accatctgct ttctgtgtgt gtggatttgg cggccccagc
147241 cgctggtgta gtggaaccac gtggcgtttg cccttccgtg gatggcttgt ctctccaagc
147301 caatgtcctt ggatccgcct acactgccgc ctatgccagg gcctccttcc ttttgggggc
147361 tgcttggtgc tccccggcac aatgggcctc accttgtttt cccccagatt cgtggacaga
147421 caccaggctg cttccacctc ttggctgtgg tcaacaagac tgctgaccct gctttcaatt
147481 ctttgtgatt ttgcactgag aaaaatacct tttaaatgca cggtcagaat ttggtggacg
147541 acaccccacc ctgcgtatgc atttggtgga cgccccaccc tgcgcatgca tttggtggac
147601 gccccaccct gcgtatgcat ttggtggatg acgccccacc ctgtgttgat gcccctctgt
147661 cctcccaggg gtcagaggtg ctaacctgtg ccccaatgcc ctggttggtc tgggcccctg
147721 gaaaccacag cgagggcagc acgctctggc tggtcctctc cccgaggccg acccggatgg

Figure S5. Complete MUC2 genomic sequence for Rp13-870H17. Genomic sequence for MUC2 showing locations for Exons and Introns. Detailed information about the missing region in NG-6867 (Underlined), HinfI restriction sites (marked in bold pink) used in NG-7351 and the Sanger sequencing Contig (Marked with start and stop (Blue).

```
   1 CAACCCACAC CGCCCCTGCC AGCCACCATG GGGCTGCCAC TAGCCCGCCT GGCGGCTGTG   Exon 1
  61 TGCCTGGCCC TGTCTTTGGC AGGGGGCTCG GAGCTCCAGA CAGgtgagag agcagacaca
 121 ggggtctggg gcctggcaga gtgtcctggg ggcagggcga ggcgggcggg caagtcgcgt
 181 ctgggaggag gagctggtcc cagagtgcag cctgcgcggc tctgctgagg ctcctggccc
 241 gggttggtcc ctggaagccc ccggccctgc tgactttcaa ggagctggaa ggtcggggct
 301 cccctgctat tcctttgggg ttgactgccc gacgacagtg tgggtcttgg ggccagcacc
 361 aggtggaaac agcaggtcag gccccagtga actgggtcat tgtccatagg ggaggaaggg
 421 gtggccagga tcccaccaga aggccccatt ctcaggtggc agagacccttgaagagttgg
 481 ggcagcacag cccttgctgg ggagcggggt gcccagaatg ccctctccta catcccgctt
 541 ggcacccggc cgcactcctc accaggccgg gggtagaagc cctgagaccc ctgtggtggg
 601 gtgaccaagg cccagcagag ggcccgagga taggaaggaa cctttcccgg ccaggggccc
 661 tgtgctgggc tcgaagctgc ttccaggtgc ttcttcaggg gccttctctc gagggtagct
 721 tgggcagcct tccccctccg gggccactca cccctcattc cccgctgctc cctcagAGGG
 781 CAGAACCCGA AACCACGGCC ACAACGTCTG CAGCACCTGG GGCAACTTCC ACTACAAGAC
 841 CTTCGACGGG GACGTCTTCC GCTTCCCCGG CCTCTGCGAC TACAACTTCG CCTCCGACTG   Exon 2
 901 CCGAGGCTCC TACAAGGAAT TTGCTGTGCA CCTGAAGCGG GGTCCGGGCC AGGCTGAGGC
 961 CCCCGCCGGG GTGGAGTCCA TCCTGCTGAC CATCAAGGAT GACACCATCT ACCTCACCCG
1021 CCACCTGGCT GTGCTTAACG GGCCGTgtg agtgtggtcg gtggcacccc tcccacatcc
1081 tagcaacggg ggctgatgtt tcccaaaggg atattccttg tagccctaga agacccсttc
1141 cgccccagca cacagctcag gagaacagcc ttgaggtttg ggttcaggtc actaattcat
1201 tcaacaaaca ctgatgagcc cccaccattc cccccatagg caaggggttt cagttatccc
1261 tttgcctgtg tgtccctgac agcccctccc ctcggagccc accaggctcc ggacagactt
1321 ggcacccctg gaggctgcat gtctctggtc ctgtgcatgg agtggccgtg tgtgccctcc
1381 ccaggctaga gttacagaag ccggtgcagg gggctgtggg accccсttcc ccatccccag
1441 ctattgctcc cctattgtct ccagaacaat gaggccctgt aagtgcgttc ccatccagcg
1501 cctgcccctc ttctgcctgg ggatttagtt tcctgcaagg ggccccagca tgggcatggg
1561 caggcgggtg gaggccctca ggcatgggca tgggcaggcg ggtgggtaga ggccctcagg
1621 cgtgggtgca ggcaggtggg tagaggccct caggcatggg catgggcggg cgggtgggta
1681 gaggccatca ggtgtgggcg tgggtgggtg ggtagaggcc ctcaggcatg ggcgcgggcg
1741 ggtgggtggg tagaggccct caggcgtgag tgcgggcggg tgggtggata gaagccgtca
1801 ggcatgggtg caggcgggtg ggtagaggtc ctcaggtgtg ggcatgggca ggtgggtggg
1861 tagaggccgt caggtgtggg cgcgggtggg tgggtagagg ccctcaggca tgggtgcggg
1921 cgggtgggtg ggtagaggcc ctcaggcgtg ggcgcgggtg ggtggataga ggccgtcagg
1981 cgtaggtgcg ggcgggtggg tagaggtcct caggtgtggg cgcaggtggg tgggtgggta
2041 gaggccctca ggcatggcac aggtgggtgg gtagaggccc tcaggcatgg gcgcaggcgg
2101 gtgggtgggt aggggccctc aggcatgggt gttggcaggt gggtgggtag aggctttcag
2161 gcatgggcag gcaggtagag gcccttgagg accgaggcac agaggctggg gtgagtgcct
2221 ctacctggac cagcaagggg cactggcagg aggtggggta gggcccctga cagtctcagg
2281 ggcagcctgg ggggctctgg ggggtttggg accccatggg gggatgttcc accaagcagg
2341 gggcctggaa gggggctggg cagcctggtc ctccctcctc tcccaacctg gtgccctcag
2401 ggcctctgag gggggaccct gcccaggacc gtgccccgag gagggagtgg agaggagggg
2461 cgtgcaggca ggaggtggct ctgccgggga agcccggcca gcggagatgg acaggtgctc
2521 tttggccact gcctatgtcc ctccacccca gaggccggcc aagttggtga ccccagggca
2581 ggagctgggc ctggcagagc catctccacc accccaggcg cccagcttca gtcccctctg
2641 ggcggcgggg tcccgggagg acaagctggg gcggggggc ctgggtggtg gacccaagag
2701 tgaccccgat gtgcctccgc cagGGTCAGC ACCCCGCACT ACAGCCCCGG GCTGCTCATT   Exon 3
2761 GAGAAGAGCG ATGCCTACAC CAAAGTCTAC TCCCGCGCCG GCCTCACCCT CATGTGGAAC
2821 CGGGAGGATG CACTCATGgt gctcaggggt ccccggactc gtggggctgg tgggggctcc
2881 gtcaggcctc tgggcagacc ccaagggagg gcagggaggg cagtgctctg acccctcacc
2941 gagagggcat gggtggggca gggcctcggc agcgcggggc gtcggtgctg gacttggggg
3001 gcagcagcag aagccgacct ggccctgacc cccccaggcc tcagccttcc cccaaacgca
3061 ctcggcttct cagggacctg ccctgccagg ccgctccctg gctgctgacc ccagccttcc
3121 tgccccacct tcctctggct caaacaagcc acgagtcttg gggggttcctg gcggctgtgg
3181 gccgggcggg aggccagctc acctgctccc tcccgcaaca gCTGGAGCTG GACACTAAGT   Exon 4
3241 TCCGGAACCA CACCTGTGGC CTCTGCGGGG ACTACAACGG CCTGCAGAGC TATTCAGAAT
3301 TCCTCTCTGA CGgtgaggcc cggagggctt ggagggggca gggtaggcta cgggcccccca
3361 ggagccctag ctgaagggcc gtgcatcccc agGCGTGCTC TTCAGTCCCC TGGAGTTTGG   Exon 5
3421 GAACATGCAG AAGATCAACC AGCCCGATGT GGTGTGTGAG GATCCCGAGG AGGAGGTGGC
3481 CCCCGCATCC TGCTCCGAGC ACgtgagtcc cctcggtctg ggtgggggt cctggcggag
3541 ctggcctctg aatagcatgc tcaccctgcg tctgtcccca gCGCGCCGAG TGTGAGAGGC
3601 TGCTGACCGC CGAGGCCTTC GCGGACTGTC AGGACCTGGT GCCGCTGGAG CCGTATCTGC   Exon 6
3661 GCGCCTGCCA GCAGGACCGC TGCCGGTGCC CGGGCGGTGA CACCTGCGTC TGCAGCACCG
```

3721 TGGCCGAGTT CTCCCGCCAG TGCTCCCACG CCGGCGGCCG GCCCGGGAAC TGGAGGACCG
3781 CCACGCTCTG CCgtaagccc cggcgccttg tgggcagggg accccaggga gaccccacgc
3841 tggtgctttc cccaagcccg ggtgggagct gtgtctgcgc cgggcacctt gagctggggg
3901 gacactcacc gcaccgggca ccttgagctg ggggaacact caccgtgccg ggcaccggga
3961 gctgggggga cactcaccgt gccgggcacc ttgagctggg gggacactca ccgtgctggg
4021 cactgggagc tgggggggaca ctcactgagg gcaccgggag ctggggggac actcaccgtg
4081 acgggcaccg ggagctgggg ggacactcac cacgggcacc gggagctggg gggacactca
4141 ccacgggcac cgggagctgg ggggacactc accgcaccgg gcaccttgag ctgggggaac
4201 actcaccgtg ccgggcaccg ggagctgggg ggacactcac cgtgccgggc accttgagct
4261 ggggggacac tcaccgtgcc gggcaccggg agctgggggg acactcactg agggcaccgg
4321 gagctggggg gacactcact gtgacgggca ccgggagctg gggggacact caccacgggc
4381 accgggagct ggggggacac tcaccacggg caccgggagc tgggggggaca ctcaccacgg
4441 gcaccgggag ctgggggggac actcaccacg ggcaccggga gctgggggga cactcactga
4501 gggcaccggg agctgggggg acactcaccg cgccgggcac tgggagctgg ggggacactc
4561 actgagggca ccgggagctg gggggacact caccgcgccg ggcactggga gctgggggga
4621 cactcactga gggcaccggg agctgggggg acactcacca cgccgggcac cgggagctgg
4681 ggggacactc accgtgggct gagagccctt ctcggtgcac ttcggggtgg agcggctgct
4741 gtgccccagc ctcaccctca ctgcgtggcc tctgcggttc cagCCAAGAC CTGCCCCGGG Exon 7
4801 AACCTGGTGT ACCTGGAGAG CGGCTCGCCC TGCATGGACA CCTGCTCACA CCTGGAGGTG
4861 AGCAGCCTGT GCGAGGAGCA CCGCATGGAC GGCTGTTTCT GCCCAGAAGg tgcgtgtgga
4921 ggatggcccc gccccggcac tgcccaccag atgagaggca gccctggcct ggggttctcg
4981 cctgcgctga ggggacggct ccgctgggtg gtgggggcag cggcggcaca gaagtgcctc
5041 tccctccacc cgataccggg ggagaagggg cctcggtgtg aggcccttcc caaagggtgg
5101 cttcagggag gccgggaagg gggctgcctt cctggttatc accctgggga cagacctcct
5161 cctgcccggc ccctggcctg gtgcctgagg cctttgggag cagctcgatt gtcaggggca
5221 ggaaggtggc ctggaggctg gaccccccatg gccagacccc aacccaggga ccaggtgggg
5281 accgcaggcg tcagcacagg ggaccagtgg tgcctgcggg tgggaggcct ggctggcagc
5341 ccctcggtgg ggattctggc tctttctgag ccagccgggg tgacatcgcc tccctggctg
5401 tcccagGCAC CGTATATGAC GACATCGGGG ACAGTGGCTG CGTTCCTGTG AGCCAGTGCC Exon 8
5461 ACTGCAGGCT GCACGGACAC CTGTACACAC CGGGCCAGGA GATCACCAAT GACTGCGAGC
5521 AGTGgtgagt cccgggggcca gggctgggca cagcagaggc tggggcggct gagccctgac
5581 cctgtgcccc gctgcccaac agTGTCTGTA ACGCTGGCCG CTGGGTGTGC AAAGACCTGC Exon 9
5641 CCTGCCCCGG CACCTGTGCC CTGGAAGGCG GCTCCCACAT CACCACCTTC GATGGGAAGA
5701 CGTACACCTT CCACGGGGAC TGCTACTATG TCCTGGCCAA Ggtaggctgc ccagggtctg
5761 gggcatgggg cagagctggg gctggcatcc aggcccttgg ctgtcccggg gtgggtgggc
5821 tggctgtccc tgaagcagag ggtgcctgtg ggctgtcctg gggcaggtga ccatgcttct
5881 gctctctggc tggagaataa gaagcaggcc ttcctttcta agccactgcc gggtcctagg
5941 gtgcagggtg ctgcccgtcc cggccctcag cagctgcact gcctcttgcc ccatcacagG Exon 10
6001 GTGACCACAA CGATTCCTAC GCTCTCCTGG GCGAGCTGGC CCCCTGTGGC TCCACAGACA
6061 AGCAGACCTG CCTGAAGACG GTGGTGCTGC TGGCTGACAA GAAGAAGAAT gtgagtggtc
6121 ctgccccctc cttctggagc cccaggtccc ccgagggggg cccttctcag ccctgagcaa
6181 cctcggcctt ccctgcagGT GGTGGTCTTC AAGTCCGATG GCAGTGTACT GCTCAACGAG Exon 11
6241 CTGCAGGTGA ACCTGCCCCA CGTGACCGgt gagttgtgcc ccagggaggg gcccgggccc
6301 ttcgagctcc actgggcctg cagtgattcg gacagtccag ccacctcgga cccaggaggc
6361 tgggtgggaa ggttccacgg ggggagggtc cctgcggcac ccagcaggct ccgtcctggg
6421 tcctctgctg gagggggtgg tgggagggtg acaccctccc gctgctcacc tgggccaggc
6481 aggtcccggg agccccgccc ctcgccatgc cccttactgt gtccctcatc gtgcccctgc
6541 ccacagCGAG CTTCTCTGTC TTCCGCCCGT CTTCCTACCA CATCATGGTG AGCATGGCCA Exon 12
6601 TTGGCGTCCG GCTGCAGGTG CAGCTGGCCC CAGTCATGCA ACTCTTTGTG ACACTGGACC
6661 AGGCCTCCCA GGGGCAGGTG CAGGgtaagt ggccccaccg gggttgcccc aacaaaggcc
6721 cacagggggg cctgctagcc ccagactctt cccaaccctg tcctggcccc tcagGCCTCT
6781 GCGGGAACTT CAACGGCCTG GAAGGTGACG ACTTCAAGAC GGCCAGCGGG CTGGTGGAGG Exon 13
6841 CCACGGGGGC CGGCTTTGCC AACACCTGGA AGGCACAGTC AAGCTGCCAT GACAAGCTGG
6901 ACTGGTTGGA CGATCCCTGC TCCCTGAACA TCGAGAGCGg tgaggctcgg caacacgggc
6961 gcccccacct agcgtgccta gggtacccgg cccatggcct ggaagggcag acggggctcc
7021 cagcaggaag catgggtggt gaggggcaga agtgaggtgg ctctcctcca ggggcagccc
7081 ggccccctgct gcttcctgct gtggctagtt tatggcggcc atggtggcag cctgccaggt
7141 gacctggaag agggcctggg ctggtcccta cctgccccgt catgtccagg atgctgggcc
7201 cttgggggtg agagacggga ggtggtgggt gccctgcagg ggtttctatc tagccaggag
7261 ctgcctggaa atttgactca cggggaggaa ggggcctggg catcggtgca cagagggaac
7321 catatctggg gcctaggcag ccaggcagca gggcccaggg gatctcacgg gggtcccggg
7381 ccccgctgaa gttccgatcc cccactcccc agCCAACTAC GCCGAGCACT GGTGCTCCCT Exon 14
7441 CCTGAAGAAG ACAGAGACCC CCTTTGGCAG GTGCCACTCG GCTGTGGACC CTGCTGAGTA
7501 TTACAAGgtg ggtgggaccc acaccccccag gcccccatgc catcgaggtg gactcagggc

7561 acccccagcc ccccatgcca cccgtgaggt ggactcagag cacccggttg ggcccactgg
7621 ttgctgtgtg tgcgtgtgag cttgcatctg tgagcgccgg gccacactct gcctccctgc
7681 ctcactgccc gtccaccttg ctctgtcgcc cagAGGTGCA AATATGACAC GTGTAACTGT
7741 CAGAACAATG AGGACTGCCT GTGCGCCGCC CTGTCCTCCT ACGCGCGCGC CTGCACCGCC Exon 15
7801 AAGGGCGTCA TGCTGTGGGG CTGGCGGGAG CATGTCTGCA gtgagtgccg tccccgtggg
7861 ctgcatcctg ggatggggt ccgggctttg agctcctggg acggggctgg gggccctgag
7921 cacgggtggt ccagggagag gggtcggccc cctgcagcca cggaccaggc tccagcttcg
7981 tcagccggtg gtagcaggaa accagcaact cctatagcaa ggggcggcca cgtagcaggg
8041 gcagaacctg gggtgggcct ggagctgtgg cggccgagtg tgggagtggg tcccagagtg
8101 tgcactccct ggccccctgg ccaccctggg gatgggagct gggcgtctgg ctcttcccgt
8161 ccctcacacc accccgtggt cctctgcagA CAAGGATGTG GGCTCCTGCC CCAACTCGCA
8221 GGTCTTCCTG TACAACCTGA CCACCTGCCA GCAGACCTGC CGCTCCCTCT CCGAGGCCGA Exon 16
8281 CAGCCACTGT CTCGAGGGCT TTGCGCCTGT GGACGGCTGC GGCTGCCCTG ACCACACCTT
8341 CCTGGACGAG AAGGGCCGCT GCGTACCCCT GGCCAAGTGC TCCTGTTACC ACCGCGGTCT
8401 CTACCTGGAG GCGGGGGATG TGGTCGTCAG GCAGGAAGAA CGATGgtggg tacctgctcg
8461 ggggtcaggt gtggcgtggg ggcgggggag ctccttctga acctgcccca agcggagacc
8521 tgggagtctc tacctgggga agctgagaca cccaaggctg aggggtgcct ggggtggggg
8581 gcgctgagag gcatcaggct cacatctgcg gggaagctgc gggctgtctg tggccgtcct
8641 gcatgggccc cgctcatccc tggcctttc cacagTGTGT GCCGGGATGG GCGGCTGCAC Exon 17
8701 TGTAGGCAGA TCCGGCTGAT CGGCCAGAgt aagtggcact gccccggcca cccctcccca
8761 gccacccctc cctgcctgcc ctggccaccc tccccggcca cccctcccgg gcctgcctga
8821 gacccccagc ttcagctgga gctgaggtgg cccctccgtc ccacagGCTG CACGGCCCCA
8881 AAGATCCACA TGGACTGCAG CAACCTGACT GCACTGGCCA CCTCGAAGCC CCGAGCCCTC Exon 18
8941 AGCTGCCAGA CGCTGGCCGC CGGCTATgtg cgtgttgggg gcgctgctgt gggcgggcag
9001 ggattcctgg ctggctgagc ctggctcttg tgctgtgccc ccgctagggt ctgggtgccg
9061 agtcctgagg acgcaggccc tgttgatgct gtccctggcc ctgggaggga agtggcagcc
9121 tgtgagccac cggggcacag gggccagtgt agggcccttg gccggcagcc ctcaccagtc
9181 tcactgccct gtggcgggcc caaggggagg gaagcctgag cccaggccag ggggagtggt
9241 gggaggtctg ggacatgaca gagactgcac ggtcaggcct ttcctggttg cacatccaat
9301 cctgacccca gggagggctg cagcctcacc tgtccacccc tgaaccccac tctctggctg
9361 tccccagTAC CACACAGAGT GTGTCAGTGG CTGTGTGTGC CCCGACGGGC TGATGGATGA Exon 19
9421 CGGCCGGGGT GGCTGCGTGG TGGAGAAGGA ATGCCCTTGC GTCCATAACA ACGACCTGTA
9481 TTCTTCCGGC GCCAAGATCA AGGTGGACTG CAATACCTGg taagctggcc cggcctgtcc
9541 tggctgcctc ccaggcccca cgtgctccgc aggggtggcc actggagagc ggtccaaggg
9601 gcaagtgcct ctcctggggg ttccgcctgg gtcttgcgag atcctgtggt ggcccctgtc
9661 ccacgggcag ggtggtctct catgtcaact gctggtcttg aagccatggg agaagggaca
9721 tttggagcca cttttggggc ctgcaggtgt cctgtgtggg aggcacaggg agctgtctgc
9781 acggtgccca gggtctcctc cagccaccca tgagcaggtc ctgggtccct tcaggctcct
9841 ctcctgtcct cctcagCACC TGCAAGAGAG GACGCTGGGT GTGCACCCAG GCTGTGTGCC Exon 20
9901 ATGGCACCTG CTCCATTTAC GGGAGTGGCC ACTACATCAC CTTTGATGGG AAGTACTACG
9961 ACTTTGACGG ACACTGCTCC TACGTGGCTG TTCAGgtgtg gtcacgggca ctgcctggtc
10021 gggctgctta tggtcaggga ccctctgcct gccccaagtg cagtgcttag ctcccccgaga
10081 aaccctgaga cttgggaagg ccggcctttc ctcagcccca gacccgcacc tgcacccgca
10141 ggaggattcg ttcttctagc cagggctggg taggggtggt aaaacccctc tgtactgccc
10201 agttctgtgg ttctcctctg ggtcctcctc tgggttctcc tgtgggtcct cctctgtggt
10261 tctcctctgg gtcctcctct gggtcctccc tcctctggat cctccctcct ctggatcctc
10321 cctcctctgg gtcctccctc ctctgggtcc tccctcctct gggtcctcct ccaggtcctc
10381 ctctgggtcc tccctcctct gggtcctcct ctgggtcctc ctctgagtcc tcctctgggt
10441 cctccctcct ctgggtcctc ctctgagtcc tcctctgggt cctccctcct ctgggtcctc
10501 cctcctctgg gtcctcctct aggtcctcct ctgtggtcct catttgggtc ctcctctggg
10561 tccttctctg ggtgcacaag gtgggtgcac cagccatggg gactgagggc acctgtttgg
10621 ggagctgagt aaaggccagg gctaggccgc tgcccgcgcg gctctccaga tccaaatccc
10681 acagcccttt gaggcaccgt gatccccagg gacaggggac aggcctgcag cagggtcagg
10741 tccttggatg ggccaggcca gggcctggtt tgtctgctca gtggctgtga ccctgccaac
10801 tggggcgggt gtgccccggg acacctgggg tccagctgtc ctggctgacc ttgccctcct
10861 ggcccccagG ACTACTGCGG CCAGAACTCC TCACTGGGCT CATTCAGCAT CATCACCGAG Exon 21
10921 AACGTCCCCT GTGGCACTAC GGGCGTCACC TGCTCCAAGG CCATCAAGAT CTTCATGGGG
10981 gtgagtgctg ctggccctgg ggacgcgtga gccctgcggg accctcagac cagccagtga
11041 ctgggcctct cctccgggca gAGGACGGAG CTGAAGTTGG AAGACAAGCA CCGTGTGGTG
11101 ATCCAGCGTG ATGAGGGTCA CCACGTGGCC TACACCACGC GGGAGGTGGG CCAGTACCTG Exon 22
11161 GTGGTGGAGT CCAGCACGGG CATCATCGTC ATCTGGGACA AGAGGACCAC CGTGTTCATC
11221 AAGCTGGCTC CCTCCTACAA Ggtgggctgc ctccctgcct gccctgcccc ctcctggcca
11281 gccccccacc ccctgccctg gtgtttgcag gacaagcccc tgtcctccct ccagcccctt
11341 tttggagccc ctgtgatgct tgtctcttgc agGGCACCGT GTGTGGCCTG TGTGGGAACT Exon 23

```
11401 TTGACCACCG CTCCAACAAC GACTTCACCA CGCGGGACCA CATGGTGGTG AGCAGCGAGC
11461 TGGACTTCGG GAACAGCTGG AAGGAGGCCC CCACCTGCCC AGATGTGAGC ACCAACCCCG
11521 AGCCCTGCAG CCTGAACCCG CACCGCCGCT CCTGGGCCGA GAAGCAGTGC AGCATCCTCA   Exon 23
11581 AAAGCAGCGT GTTCAGCATC TGCCACAGCA AGgtgggctg gccgggccat ggtggggcaa
11641 gtaggcagag gagggctgta ggtgggctgt gactgtgggc tggggccatg ggcggggccg
11701 actaagcaga gcagggctgt aggtgggcta tagctgtggg cggggccatg ggcggggccg
11761 actaagcaga gcagggctgt aggtggacta tagctgtggg cggggcatgg cggggctaac
11821 taggcagagc agggctgtag gtgggctata gctgtgggcg gggccatggg cggggccgac
11881 taagcagagc agggctgtag gtggactata gctgtgggcg gggccatggg cggggccgac
11941 tgtaggcaga gcagggctat gggctgactg tgggcgtggt gagggtgccg tagagcatgc
12001 taatgaccag ggcgtggtca tagcagggta gggtcttggg tgctcctggg gctgggggc
12061 ttctccacat gctccccaca ccttcaggag tcgccctgct gcgtcacgca ccacacggcg
12121 cttgtcctcc agctttggct ctggccgctg cctcctttgg tcacatgacc gtataatcgg
12181 cctcccctct gagaccctgg gctggacccc cggcctccct ctgcctcccc aggctcagat
12241 attcacccgg agggagaaag gacatgtgtc ccccatgccc acacatcccc agctacaggc
12301 agctggggag gacgggttct aggatggcca tgttacagct gaggatgcag aggggttggg
12361 tgatgggtct gcacagccac ggcgggacag gtgtctctgg accctctccc caaggttggc
12421 cctgccgggg ccctggctgg ctggtgctgg gtaatgtgcc ctgtcccagg agcagggccg
12481 gcctcagggt cctgagctcc agggcactgg ggaagtcctg gctccatgag ggcaggacgg
12541 gcccaggaca gaccagggtg ttctccccag GTGGACCCCA AGCCCTTCTA CGAGGCCTGT
12601 GTGCACGACT CGTGCTCCTG TGACACGGGT GGGGACTGTG AGTGCTTCTG CTCTGCCGTG   Exon 24
12661 GCCTCCTACG CCCAGGAGTG TACCAAAGAG GGGGCCTGCG TGTTCTGGAG GACGCCGGAC
12721 CTGTGCCGta agagcctgcc cgaactgcac tcagggccgg gacgggggct gggaggtgct
12781 gtattgcggg ccggggtgac actccttgtc catccaggtg atgggtgtgc atcacccacc
12841 ctttccccga cttctccagt gtccttcttt ggggccctgt gggacccggg ttggcagagc
12901 aagcttgatg cgtctgcgtc ccagcccccg accccagatt cgccctcacc ccggcccagg
12961 cctgagccct cctgcgtctg accctggccc tgtctccccc aagCCATATT CTGCGACTAC
13021 TACAACCCTC CGCATGAGTG TGAGTGGCAC TATGAGCCAT GTGGGAACCG GAGCTTCGAG   Exon 25
13081 ACCTGCAGGA CCATCAACGG CATCCACTCC AACATCTCCG TGTCCTACCT GGAGGgtgag
13141 cagggtgggg cgggcttcag cgggggtgat ggccgagggg cctggaggct gagtggggca
13201 gccctcggga gaggcaacag tccactggcc tggagggtga gccaggcggc cctcgggggа
13261 ggctacggcc gacgggcctg gcactgtggg gctgaaggct gatgtctgga gacccatggg
13321 gacacccgga gggaggcctg accctcaggg tacccacagc ccagggcagc caggctcccc
13381 ttgctgcagg atcaggaggg aagcaggcta tcgtggaaac tgggagtggc aggggtggga
13441 ggtgctgagg ttcgtgcaga gcagggcggg ttggggagca tttcaggcac aggtcagggg
13501 aggcccctgc cgggtgctgg tgtctgagct gagaaccagt gacgtgaagg agggactggt
13561 gggaagtttg ggaggagtat cccgccatgg gagaggaaca tgggtcttgg gactcagggc
13621 tgctcggggg gcccgatgag actgggcagg gctcctcagc aggcagcgtt cagggctcag
13681 tggggtgggg agatccaggc cctgcctttc caatccccgg ccttcccaga ggggcatcct
13741 gcagagaagg gcctgccagg gtagggacgg tgggtggggt gtggtggact gcggtggtcc
13801 caaccctatg ccctgtgtcc accagGCTGC TACCCCCGGT GCCCCAAGGA CAGGCCCATC   Exon 26
13861 TATGAGGAGG ATCTGAAGAA GTGTGTCACT GCAGACAAGT GTGGCTGCTA TGTCGAGGAC
13921 ACCCACTACC CACCTGGAGC ATCGGTTCCC ACCGAGGAGA CCTGCAAGTC CTGgtaccta
13981 agcccacgtg gcagggggcc tgggggagct gcacatatgg gcacatgagt acacacacac
14041 gtgtgagcac acagtgtaca cagtacacag acacacaacc gttccacatg ggtgcacatg
14101 cacacaaacg cacacagcat accacgtgca cacacacggt cacatgcatg catggtgcac
14161 acatgcacac atgaatggat gccaacatgc aggcacacac agtcacacat gcacacagcg
14221 cacacatgga cacatgccta gacgcagata cccaggcata cactcacggt tacacactca
14281 cgcacatatg catggatgca gacacgcagg cacacacggt catatagtca tacaccacat
14341 gcacacatgc acagacagac acccaggcac acacagttac acagtcacac atgcacacat
14401 gcatggatgc agacacgcag gcgcacacac acatgcacag tgcacacgta cacatgccta
14461 gacacagata cccaggcaca cacagtcaca catgcatgga cacagagtca catgtgcaca
14521 catacacacg tgtggacaga cataggcaca gtcacgtgca cacatgcact cacactcagt
14581 cacacatgaa catgtgctca catgcatgga cactgacacg caaggacaca cagtcacaca
14641 tgcacacatg catagacaca gacacccagg cacacacagt tacacagtca cacatgcatg
14701 gatgcagaca cgcagtcaca cagtcacaca tgcacacact gcacacatgt acacatgcct
14761 agacacagat atgcaggcac acacacatag tcaaacatgc acacatgcat ggacacaaag
14821 tcacacgtgc acacatgcac acatgcatgg acagacacag gcacacacag tcacgtgcac
14881 agatgcactc acagtcacac atgaacacat gctcacatgc acagacactg acacgcaggc
14941 acacacagtc acacatgtac acgtgcctag acacagatac ccagacacac acaattacac
15001 agtcgcacag tcacacatgc atggatgcag acacacaggt acacaaggtc acacagtcat
15061 ataatgcaca catgcacaca tgcatagata cagacaccca ggtacacact cacggtgaca   Sanger seq.
15121 cagtcacaca tgcacacatg catggaggca gacacacaag cacacacagt cacacagtca   Contig Start
15181 cacatgcaca caggagccag gctacagagg taccagtccc tcactgcggc gggggtctt
```

15241 ctgttctcat cccatcctct gggtctggct ttttccttcc tctcctcgcc cctgctctgt
15301 tcccacagtt acaacccagt ggggggctct tccggagctg gctttggggc agtgcctggg
15361 ggctttgggc tcggtactag ccacatgggg aagctggggg tctgagcagc gtgggcgcgt
15421 tgtcagtgga gtgggacttg tagccatgtg cttgctttgc agCGTGTGTA CCAACTCCTC Exon 27
15481 CCAAGTCGTC TGCAGGCCGG AGGAAGgtaa gctgccctct gctgccagcc ctgcggtggc
15541 cgggcccatc ctggggaagc ctgtggggcc ttggatcggt gggggtgct ggtctcctcc
15601 tgggctctgc ccctttggtc cccccccagc tcagacccac ctccgatgtg tatcagccct
15661 ggggggctgc tgtgacccat tttgtttctt ctggggtgtc ggtgtcctgt ggggaatttc
15721 cgtcacccctc tcccgtgatc cagcttctgc gttctgatga gattcccttt attcaaagag
15781 aggggctctg ggacgggtgc agtctcactg gagcatttct tagctgcttg tgggggctcg
15841 ggcacacctg gccttcttcc tatcttgctc ctgatgaggt gattcttggc ctcaccctca
15901 cccccagGAA AGATTCTTAA CCAGACCCAG GATGGCGCCT TCTGCTACTG GGAGATCTGT
15961 GGCCCCAACG GGACGGTGGA GAAGCACTTC AACATCTGTT CCATTACGAC ACGCCCGTCC Exon 28
16021 ACCCTGACCA CCTTCACCAC CATCACCCTC CCCACCACCC CCACCACCTT CACCACTACC
16081 ACCACCACCA CCACCCCGAC CTCCAGCACA Ggtaaggccc cctggttccc tccatgcttc
16141 ctcgggctct caccttcccc tgcatccagc atccagcaca gagggctctt tcgggggcag
16201 gccccggcct ggtgcagcca ggctgtgacc cctgcacacc agctgcagag tgaggtgaca
16261 gtggcattcc tctgcactga ggtgtgaggg ggcctgccct ggctcccctg gcctggtgca
16321 ttgagatagt agcatcctga ccacatcccc aagcccagac cacagtggag gatcacctgg
16381 ggagatttct gaaaaccagc aggaaactat ccctaagggt tagagaaatt ttcttatgtt
16441 cccctgcgtt tgttctggtt gaaatcctag ctaccactga acaagccacc aggggtatga
16501 tagccacaga aaaaagaaac tttttttaaa aaaggcaaga ttttaaaaga tcttgaacta
16561 tataatgata tcctcttttc ttcctgcttt attgcagTTT TATCAACAAC TCCGAgtaag Exon 29
16621 tgacggtgat gatattcatg atgacaagca gggtgggagg agcgaagtct tataaaatca
16681 cctgcaggat gcttccttca gggcccagat gtgaggctgg cggggctgga ctcctctgct
16741 tatggaccaa agatggatgt attttggcca cttcattcat ggtttgctga ggccaggggc
16801 taaagtgaga cctgattggc tgtcggtgac aatattgctg gttaagagtg gagacaaagc
16861 cccttccgtc acacttcctt actggaatgg gaagctctct tgttattgat tctttgaaaa
16921 aaaagtattg aaaatagctg aggaaagggt ccatcacacc caggtgtggc cctgggtggc
16981 cccgtctctt tgggctcagg ttttcagttg caaaatgagg atggaagtgg tgtccagccc
17041 tgagctctct ggccctgcac tctggttttt tggcaatgac agggaaaaga gagattgcag
17101 ctgggggatg gtcatggagg tccctgggtc ctctgaatcc tggtggcttc ctggaggtgc
17161 ctctccccag gtgtgagaga caagaacttg gttttgcttc cctagAGCTG TGCTGCCTCT Exon 30
17221 GGTCTGACTG GATCAATGAG GACCACCCCA GCAGTGGCAG CGACGACGGT GACCGAGAAA
17281 CATTTGATGG GGTCTGCGGG GCCCCTGAGG ACATCGAGTG CAGGTCGGTC AAGGATCCCC
17341 ACCTCAGCTT GGAGCAGCTA GGCCAGAAGG TGCAGTGTGA TGTCTCTGTT GGGTTCATTT
17401 GCAAGAATGA AGACCAGTTT GGAAATGGAC CATTTGGACT GTGTTACGAC TACAAGATAC CysD coding
17461 GTGTCAATTG TTGCTGGCCC ATGGATAAGT GTATCACCAC TCCCAGCCCT CCAACTACCA region
17521 CTCCCAGCCC TCCACCAACC AGCACGACCA CCCTTCCACC AACCACCACC CCCAGCCCTC
17581 CAACCACCAC CACAACCACC CCTCCACCAA CCACCACCCC CAGCCCTCCA ATAACCACCA
17641 CGACCACCCC TCCACCAACC ACCACTCCCA GCCCTCCAAT AAGCACCACA ACCACCCCTC
17701 CACCAACCAC CACTCCCAGC CCTCCAACCA CCACTCCCAG CCCTCCAACC ACCACTCCCA
17761 GCCCTCCAAC AACCACCACA ACCACCCCTC CACCAACCAC CACTCCCAGC CCTCCAACGA
17821 CTACGCCCAT CACTCCACCA GCCAGCACTA CCACCCTTCC ACCAACCACC ACTCCCAGCC
17881 CTCCAACAAC CACCACAACC ACCCCTCCAC CAACCACCAC TCCCAGTCCT CCAACGACTA TR1
17941 CGCCCATCAC TCCACCAACC AGCACTACTA CCCTTCCACC AACCACCACT CCCAGCCCTC
18001 CACCAACCAC CACAACCACC CCTCCACCAA CCACCACTCC CAGCCCTCCA ACAACCACCA
18061 CTCCCAGTCC TCCAACAATC ACCACAACCA CCCCTCCACC AACCACCACT CCCAGCCCTC
18121 CAACAACGAC CACAACCACC CCTCCACCAA CCACCACTCC CAGCCCTCCA ACGACTACAC
18181 CCATCACTCC ACCAACCAGC ACTACCACCC TTCCACCAAC CACCACTCCC AGCCCTCCAC
18241 CAACCACCAC AACCACCCCT CCACCAACCA CCACTCCCAG CCCTCCAACA ACCACCACTC
18301 CCAGCCCTCC AATAACCACC ACAACCACCC CTCCACCAAC CACCACTCCC AGCTCTCCAA
18361 TAACCACCAC TCCCAGCCCT CCAACAACCA CCATGACCAC CCCTTCACCA ACCACCACCC
18421 CCAGCTCTCC AATAACCACC ACAACCACCC CTTCCTCAAC TACCACTCCC AGCCCTCCAC
18481 CAACCACCAT GACCACCCCT TCACCAACCA CCACTCCCAG CCCTCCAACA ACCACCACGA
18541 CCACCCTTCC ACCAACCACC ACTTCCAGCC CTCTAACAAC TACTCCTCTA CCTCCATCAA
18601 TAACTCCTCC TACATTTTCA CCATTCTCAA CGACAACCCC TACTACCCCA TGCGTGCCTC
18661 TCTGCAATTG GACTGGCTGG CTG*GATTC*TG GAAAACCCAA CTTTCACAAA CCAGGTGGAG *HifI* site
18721 ACACAGAATT GATTGGAGAC GTCTGTGGAC CAGGCTGGGC AGCTAACATC TCTTGCAGAG CysD coding
18781 CCACCATGTA TCCTGATGTT CCCATTGGAC AGCTTGGACA AACAGTGGTG TGTGATGTCT region
18841 CTGTGGGGCT GATATGCAAA AATGAAGACC AAAAGCCAGG TGGGGTCATC CCTATGGCCT
18901 TCTGCCTCAA CTACGAGATC AACGTTCAGT GCTGTGAGTG TGTCACCCAA CCCACCACCA Sanger seq.
18961 TGACAACCAC CACCACAGAG AACCCAACTC CGACACCAAT CACCACCACC ACTACGGTGA Contig Stop
19021 CCCCAACCCC AACACCCACC AGCACACAGA GTACAACACC AACACCCATC ACCACCACCA

19081 ATACGGTAAC CCCAACCCCA ACCCCCACTG GCACACAGAC CCCAACCCCG ACACCCATCA
19141 CCACCACCAC CACTATGGTG ACCCCAACCC CAACAATCAC CAGCACACAG ACCCCAACCC
19201 CGACACCCAT CACCACCACT ACGGTGACCC CAACCCCAAC ACCCACCAGC ACACAGAGAA Exon 30
19261 CAACACCGAC ATCCATCACC ACCACCACCA CGGTGACCCC AACCCCAACA CCCACCGGCA
19321 CACAGACCCC AACCACGACA CCCATCACCA CCACCACCAC GGTGACCCCA ACCCCAACAC
19381 CCACCGGCAC ACAGACCCCA ACAACGACAC CCATCAGCAC CACCACCACG GTGACCCCAA
19441 CCCCAACACC CACTGGAACA CAGACCCTAA CCCCAACACC CATCACCACC ACCACTACGG
19501 TGACCCCAAC CCCTACACCC ACCGGCACAC AGACCCCAAC ATCGACACCC ATCACCACCA TR2
19561 CCACTACGGT GACCCCAACA CCAACACCCA CTGGCACACA GACCCCAACC CTGACACCCA
19621 TCACCACCAC CACTACGGTG ACCCCAACCC CAACACCCAC CGGCACACAG ACCCCAACCA
19681 CGACACCCAT CACCACCACC ACTACGGTGA CCCCAACCCC AACACCCACC GGCACAAAGA
19741 GTACAACCCC GACATCCATC ACCACCACCA CTATGGTGAC CCCAACCCCA CCACCCACTG
19801 GCACACAGAC CCCAACCACG ACACCCATCA CCACCACCAC TACGGTGACC CCAACCCCAA
19861 CACCCACCGG CACACAGACC CCAACCCCGA CACCCATCAC CACCACCACC ACGGTGACCC
19921 CAACCCCAAC ACCCACCGGC ACACAGACCC CAACATCGAC ACCCATCACC ACCAACACTA
19981 CGGTGACCCC AACCCCAACA CCAACCGGCA CACCGAGTAC AACCCTGACA CCCATCACCA
20041 CCACCACTAC GGTGACCCCA ACCCCAACAC CCACCGGCAC ACAGACCCCA ACATCGACAC
20101 CCATCAGCAC CACCACTATG GTGACCCCAA CCCCAACACC CACCGGCACA CAGACCCCAA
20161 CCCCTACACC CATCTCCACC ACCACTACGG TGACCCCAAC CCCAACACCC ACCGGCACAC
20221 AGACCCCAAC CCCGACACCC ATCACCACCA CCACCACGGT GACCCCAACC CCAACACCCA
20281 CCGGCACACA GACCCCAACA TCGACACCCA TCACCACCAC CACTACGGTG ACCCCAACCC
20341 CAACACCCAC CGGCACACAG ACCCCAACCA CGACACCCAT CACCACCAAC ACCACGGTGA
20401 CCCCAACCCC GACACCCACC GGCACACAGA CCCCAACCAC GGTACTCATC ACCACCACCA
20461 CTACGATGAC ACCAACCCCA ACACCCACCA GCACAAAGAG TACAACCGTG ACACCCATCA
20521 CCACCACCAC TACTGTGACC CCAACCCCAA CACCCACCGG CACACAGAGT ACAACCCTGA
20581 CACCCATCAC CACCACCACT ACGGTGACCC CAACCCCAAC ACCCACCGGC ATACAGACCC
20641 CAACAACGAC ACCCATCAGC ACCACCACCA CCGTGACCCC AACCCCAACA CCCACCGGCA
20701 CACAGACCCC AACATCGACA CCCATCACCA CCACCACTAC GGTGACCCCA ACCCCTACAC
20761 CCACTGGCAC ACAGACCCCA ACATCGACAC CCATCAGCAC CACCACTACG GTGACCCCAA
20821 CAGCAACACC CACCGGCACA CAGACCCCAA CCCTGACACC CATCACCACC ACCACTACGG
20881 TGACCCCAAC CCCAACACCC ACCGGCACAA AGAGTACAAC CCCGACATCC ATCACCACCA
20941 CCACTACGGT GACCCCAACC CCAACACCCA CTGGCACACA GACCCCAACC ACGACACCCA
21001 TCACCACCAC CACCACGGTG ACCCCAACCC CAACACCCAC CGGCACACAG ACCCCAACCC
21061 CGACACCCAT CACCACCACC ACCACGGTGA CCCCAACCCC AACACCCACC AGCACACAGA
21121 CCCCAACATC GACACCCATC ACCACCACCA CTACGGTGAC CCCAACCCCA ACACCCACTG
21181 GCACACAGAC CCCAACCACG ACACCCATTA CCACGACCAC CACGGTGACC CCAACCCCAA
21241 CACCCACCGG CACACAGGCC CCAACCCCAA CAGCCATCAC CACCACCACT ACGGGGACCC
21301 CAACCCCAAC ACCCACCGGC ACACAGACCC CAACCACGAC ACCCATCACC ACCACCACTA
21361 CGGTGACACC AACCCCAACA CCCACCGGCA CACAGTCCCC AACCCCAACA GCCATCACCA
21421 CCACCACTAC GGTGACCCCA ACCCCAACAC CCACCGGCAC ACAGACCCCA ACCACGACAC
21481 CCATCACCAC CACCACCACG GTGACCCCAA CCCCGACACC CACCGGCACA CAGAGTACAA
21541 CCCTGACACC CATCACCACC ACCACCACGG TGACACCAAC CCCAACACCC ACTGGCACAC
21601 AGACCCCAAC ATCGACACCC ATCACCACCA CCATTACGGT GACCCCAACC CCAACACCCA
21661 CCGGCACACA GACCCCAACC CCGACACCCA TCTCCACCAC CACTACGGTG ACCCCAACCC
21721 CAACACCCAC CGGCACACAG ACCCCAACAT CGACACCCAT CACCACCACC ACCACGGTGA
21781 CCCCAACCCC AACACCCACC GGCACACAGA CCCCAACAAC GACACCCATC AGCACCACCA
21841 CCACGGTGAC CCCAACCCCA ACACCCACCG GCACACAGAC CCCAACATCG ACACCCATCA
21901 CCACCACCAC CACGGTGACC CCAACCCCAA CACCCACCGG CACACAGACC CCAACCACGA
21961 CACCCATCAG CACCACCACC ACGGTGACCC CAACCCCAAC ACCCACCGGC ACACAGACCC
22021 CAACATCGAC ACCCATCACC ACCACCACCA CGGTGACCCC AACCCCAACA CCCACCGGCA
22081 CACAGACCCC AACCCCGACA CCCATCACCA CCACCACCAC GGTGACCCCA ACCCCAACAC
22141 CCACCGGCAC ACAGACCCCA ACATCGACAC CCATCACCAC CACCACCACG GTGACCCCAA
22201 CCCCAACACC CACCGGCACA CAGACCCCAA CCCCGACACC CATCACCACC ACCACCACGG
22261 TGACCCCAAC CCCAACACCC ACCGGCACAC AGACCCCAAC CCCGACACCC ATCACCACCA
22321 CCACCACGGT GACCCCAACC CCAACACCCA CCGGCACACA GACCCCAACA TCGACACCCA
22381 TCACCACCAC CACTACGGTG ACCCCAACCC CAACACCCAC CGGCACACAG ACCCCAACCA
22441 CGACACCCAT CACCACCACC ACCACGGTGA CCCCAACCCC AACACCCACT GGCACACAGA
22501 GTACAACCCT GACACCCATC ACCACCACCA CCACGGTGAC ACCAACCCCA ACACCCACCG
22561 GCACACAGAC CCCAACATCG ACACCCATCA CCACCATCAC TACGGTGACC CCAACCCCAA
22621 CACCCACCGG CACACAGACC CCAACCCCGA CACCCATCTC CACCACCACT ACAGTGACCC
22681 CAACCCCAAC ACCCACCGGC ACACAGACCC CAACCATGAC ACCCATCACC ACCACCACCA
22741 CGGTGACCCC AACCCCAACA CCCACCGGCA CACAGACCCC AACAACGACA CCCATCAGCA
22801 CCACCACCAC GGTGACCCCA ACCCCAACAC CCACCGGCAC ACAGACCCCA ACATCGACAC
22861 CCATCACCAC CACCACTACG GTGACCCCAA CCCCAACACC CACCGGCACA CAGACCCCAA

```
22921 CCACGACACC CATCACCACC ACCACCACGG TGACCCCAAC CCCAACACCC ACCGGCACAC
22981 AGAGTACAAC CCTGACACCC ATCACCACCA CCACCACGGT GACACCAACC CCAACACCCA
23041 CCGGCACACA GACCCCAACC CCGACACCCA TCTCCACCAC CACTACGGTG ACCCCAACCC
23101 CAACACCCAC CGGCACACAG ACCCCAACCA TGACACCCAT CACCACCACC ACCACGGTGA   Exon 30
23161 CCCCAACCCC AACACCCACC GGCACACAGA CCCCAACAAC GACACCCATC AGCACCACCA
23221 CCACGGTGAC CCCAACCCCA ACACCCACCG GCACACAGAC CCCAAGATCG ACACCCATCA
23281 CCACCACCAC TAAGGTGACC CCAACCCCAA CACCCACCGG CACACAGACC CCAACCCCGA
23341 CACCCATCAC CACCACCACC ACGGTGACCC CAACCCCAAC ACCCACTGGC ACACAGGCCC   TR2
23401 CAACCCCAGC AGCCATCACC ACCACCAGTA CGGTGACCCC AACCCCAACA CCCACCGGCA
23461 CACAGACCCC AACCACGACA CCCATCACCA CCACCACCAC GGTGACCCCA ACCCCAACAC
23521 CCACCGGCAC ACAGAGTACA ACCCTGACAC CCATCACCAC CACCACCACG GTGACACCAA
23581 CCCCAACACC CACCGGCACA CAGACCCCAA CATCGACACC CATCACCACC ACCACTACGG
23641 TGACCCCAAC CCCAACACCC ACCGGCACAC AGACCCCAAC CCCGACACCC ATCTCCACCA
23701 CCAGTACGGT GACCCCAACC CCAACACCCA CCGGCACACA GACCCCAACC ATGACACCCA
23761 TCACCACCAC CACCACGGTG ACCCCAACCC CAACACCCAC CGGCACACAG ACCCCAACAA
23821 CGACACCCAT CAGCACCACC ACCACGGTGA CCCCAACCCC AACACCCACC GGCACACAGA
23881 ACCCAACATC GACACCCATC ACCACCACCA CTACGGTGAC CCCAACCCCA ACACCCACCG
23941 GCACACAGAC CCCAACCATG ACACCCATCA CCACCACCAC CACGGTGACC CCAACCCCAA
24001 CACCCACTGG CACACAGGCC CCAACCCCAA CAGCCATCAC CACCACCACT ACGGTGACCC
24061 CAACCCCAAC ACCCACCGGC ACACAGACCC CAACCACGAC ACCCATCACC ACCACCACCA
24121 CGGTGACCCC AACCCCAATA CCCACCGGCA CACAGAGTAC AACCCTGACA CCCATCACCA
24181 CCACCACCAC GGTGACACCA ACCCCAACAC CCACCGGCAC ACAGACCCCA ACCCCGATAC
24241 CCATCTCCAC CACCACTACG GTGACCCCAA CCCCAACACC CACCGGCACA CAGACCCCAA
24301 CCATGACACC CATCACCACC ACCACCACGG TGACCCCAAC CCCAACACCC ACCGGCACAC
24361 AGACCCCAAC AACGACACCC ATCAGCACCA CCACCACGGT GACCCCAACC CCAACACCCA
24421 CCGGCACACA GACCCCAACA TCGACACCCA TCACCACCAC CACTACGGTG ACCCCAACCC
24481 CAATACCCAC CGGCACACAG ACCCCAACCA CGACACCCAT CACCACCACC ACCACGGTGA
24541 CCCCAACCCC AACACCCACT GGCACACAGG CCCCAACCCC AACAGCCATC ACCACCACCA
24601 CTACGGTGAC CCCAACCCCA ACACCCACCG GCACACAGAC CCCAACCACG ACACCCATCA
24661 CCACCACCAC CACGGTGACC CCAACCCCAA TACCCACCGG CACACAGAGT ACAACCCTGA
24721 CACCCATCAC CACCACCACC ACGGTGACAC CAACCCCAAC ACCCACCAGC ACACAGACCC
24781 CAACCCCGAC ACCCATCTCC ACCACCACTA CGGTGACCCC AACCCCAACA CCCACCGGCA
24841 CACAGACCCC AACCATGACA CCCATCACCA CCACCACCAC GGTGACCCCA ACCCCAACAC
24901 CCACCGGCAC ACAGACCCCA ACAACGACAC CCATCAGCAC CACCACCACG GTGACCCCAA
24961 CCCCAACACC CACCGGCACA CAGACCCCAA CATCGACACC CATCACCACC ACCACTACAG
25021 TGACCCCAAC CCCAACATCC ACCGGCACAC AGACCCCAAC CACGACACCC ATCACCACCA
25081 CCACCACGGT GACCCCAACC CCAACACCCA CTGGCACACA GGCCCCAACC CCAACAGCCA
25141 TCACCACCAC CAGTACGGTG ACCCCAACCC CAACACCCAC CGGCACACAG ACCCCAACCA
25201 CGACACCCAT CACCACCACC ACTACGGTGA CACCAACCCC AACACCCACC GGCACACAGT
25261 CCCCAACCCC AACAGCCATC ACCACCACCA CTACGGTGAC CCCAACCCCA ACACCCACCG
25321 GCACACAGAC CCCAACATCG ACACCCATCA CCACCACCAC TACGGTGACC CCAACCCCAA
25381 CACCCACCGG CACACAGACC CCAACCCCGA CACCCATCTC CACCACCACT ACGGTGACCC
25441 CAACCCCAAC ACCCACCGGC ACACAGACCC CAACCACGAC ACCCATCACC ACCACCACCA
25501 CGGTGACCCC AACCCCGACA CCCACCGGCA CACAGACCCC AACCACGGTA CTCATCACCA
25561 CCACCACTAC GATGACCCCA ACCCCAACAC CCACCAGCAC AAAGAGTACA ACCGTGACAC
25621 CCATCACCAC CACAACTACG GTGACCGCAA CCCCAACACC CACCGGCACA CAGACCCCAA
25681 CCATGATACC CATCAGCACC ACCACTACGG TGACCCCAAC CCCAACACCC ACCACTGGAA
25741 GCACGGGGCC CCCCACCCAC ACAAGCACAG CACCCATTGC TGAGTTGACC ACATCCAATC
25801 CTCCGCCTGA GTCCTCAACC CCTCAGACCT CTCGGTCCAC CTCTTCCCCT CTCACGGAGT   HinfI site
25861 CAACCACCCT TCTGAGTACC CTACCACCTG CCATTGAGAT GACCAGCACG GCCCCACCCT
25921 CCACACCCAC GGCACCCACG ACCACGAGCG GAGGCCACAC ACTGTCTCCA CCGCCCAGCA
25981 CCACCACGTC CCCTCCAGgt aagcagagct gcttggttcc tctggcctgg gatgcttctt
26041 cctccccttg tgccgggcag gactgtccca ggaaggctca aggcacgttc tgggcgcctc
26101 tctgcccacg aagcttggtc actgtgtggg cagaagccac tgacactggc cagtgctggg
26161 cagtgaagcc aaaggccatt ccgcttgccc ataggacagc cttctgagga gctgctgaca
26221 ccggccagtg ctgggcagtg gagcccttgg ctatcctgct cgcccataag acggccttct
26281 tcaggggccc actgctatgt gatgcggtgc tgtgggagcc catcaaggct gggggggcaga
26341 gagaggctgc cagtgaggtg cctgcgggtc cacctgcttc tggctgcagc ccctccttgg
26401 ggccttttcc tggtggacgg cgtgccacag ccagtgcctt ctggacgcct cttgctggcc
26461 atcggcttgg ccagcaagct gtgttgctgc cagagcacca ggtcacctgc aggctctcgt
26521 gacactcggc tgtggtgata ctggccttgc cgctccaccc tgcctggtga ctctgagagc
26581 ctgggaggtg ggcacgaggc cctggtcctc cagttctgcc acccggtcgg ctgtctggct
26641 cccttgcagc tggggagtgg cagttgggac cctgtggcat ctgagatgtg caatgtctca   Exon 31
26701 gccctcactg gtgtctcctg ctctcacagG CACCCCCACT CGCGGTACCA CGACCGGGTC
```

26761 ATCTTCAGCC CCCACCCCCA GCACTGTGCA GACGACCACC ACCAGTGCCT GGACCCCAAC
26821 GCCGACCCCA CTCTCCACAC CCAGCATCAT CAGGACCACA GGCCTGAGGC CCTACCCTTC
26881 CTCTGTGCTT ATCTGCTGTG TCCTGAACGA CACCTACTAC GCACCAGgta ctcaggctgt Exon 31
26941 tcacatcctg tgcttgggtg gccgaggctg gccccggcat gtaccaatgg gtcaggtgcc
27001 agggctgaga tcgcagtaga agcgtctcag gaggcagcag ccgtcgaggg tggctgtgtc
27061 cagggcacgg cttcccttgg gtggcctctg tggggacctc cgctgtgggg acctccacgg
27121 ggtccagcgg ctagccctgc ctccggatag ccctgcctct ggacggtgtg atcgtgggtc
27181 tgtctccctt cgcagGTGAG GAGGTGTACA ACGGCACATA CGGAGACACC TGTTATTTCG
27241 TCAACTGCTC ACTGAGCTGT ACGTTGGAGT TCTATAACTG GTCCTGCCCA TCCACGCCCT Exon 32
27301 CCCCAACACC CACGCCCTCC AAGTCGACGC CCACGCCTTC CAAGCCATCG TCCACGCCCT
27361 CCAAGCCGAC GCCCGGCACC AAGCCCCCCG AGTGCCCAGA CTTTGATCCT CCCAGACAGg
27421 tcagtgggct gcaggcggct ttgtccccat ggcactctgc gcagcatgtc cgggcagctg
27481 aggccccagg caccacttcc tgctggtcgt ctgagggccg aggcctccag caacccttgg
27541 gtgcagggtc tgccgagccc tccacatttt caccgtgccc cgctgtgcct ggcgaggtgg
27601 ctggctgcag tgaggtccgt ggaagccact tcggcctcca gcctcccggc tcagcacccg
27661 cccctcctga gcgcagacca ccccatcctg tgccggtccc cctgacgtcc cttgcctccc
27721 gtccccagGA GAACGAGACT TGGTGGCTGT GCGACTGCTT CATGGCCACG TGCAAGTACA
27781 ACAACACGGT GGAGATCGTG AAGGTGGAGT GTGAGCCGCC GCCCATGCCC ACCTGCTCCA Exon 33
27841 ACGGCCTCCA ACCCGTGCGC GTCGAGGACC CCGACGGCTG CTGCTGGCAC TGGGAGTGCG
27901 ACTgtgagtc cggggccccc aggccctccc cgcatctcct gccctctccg tgggtggggg
27961 ctgcagggcc cgtctcccgg gggcggaagg gctgaggctc cttgggcaca gatcccactg
28021 aggtgttcgc tgaggctggg tgacttctga gggtcttctc acagccctgc ttttgcctca
28081 ttgggtgggg agggcctggg caggtggagg gcttgcctgg tggagttagg gctcctccct
28141 ggaacaaggg tgcttctgag gcaagagggg gctgagttga agtttgaacc ctggtccgtc
28201 ctgcagaatg ggccactgtg ggtgcgccag ggcaagtgca gctcagacat ccccgtgccc
28261 acgcacagga gtggggtttt caggccccag cttcctgctg gctcttcctg actatgcccc
28321 agcccagccc ttgcacccga ccccggccga ggggcacagg tggcacggct cactccggct
28381 cccttgcagG CTACTGCACG GGCTGGGGCG ACCCGCACTA TGTCACCTTC GACGGACTCT
28441 ACTACAGCTA CCAGGGCAAC TGCACCTACG TGCTGGTGGA GGAGATCAGC CCCTCCGTGG Exon 34
28501 ACAACTTCGG AGTTTACATC GACAACTACC ACTGCGATCC CAACGACAAG GTGTCCTGTC
28561 CCCGCACCCT CATCGTGCGC CACGAGACCC AGGAGGTGCT GATCAAGACC GTGCATATGA
28621 TGCCCATGCA GGTGCAGGta ggcacagcgt ggccacagga ggctggcatg gaggcgggtg
28681 ctgacatggg ccccaatgca ccctggttcc ccaggggcca gaggactggg ctgtgggggt
28741 gccaaggcat agcctctcct agagctgggc tagaaggtag gatggggtgg gcgactggct
28801 ccgggacata tcagctcttc ctgcaggccc tccaggtgtg tcctgggccc ctcgagccct
28861 ggcaccatgc cacgctgggc acagtctctg cagcagaagc tgcctcctga ggacagagtc
28921 agggacaggg ctctgcacac ccttggctga gatgccccta cttgcagggg aatcattggt
28981 tctgaggctc aggaggcccc gggagcctgc gccgggctcc acagtcccca ggtgctccca
29041 ggagagctcc ttcactggct cacccatggg accagggtct ggttgggagc agtggagtgg
29101 aagcaagaaa gggggcagga aagcggggta ggcagggccc tctccctaca tgtgtaggtc
29161 agagagcagg cggggtgggg cagccctgga gctctcacaa ggagaggacc gaggcagctg
29221 cagctcccat ggtgtgtcgg ccacagGTGC AGGTGAACAG GCAGGCGGTG GCACTGCCCT
29281 ACAAGAAGTA CGGGCTGGAG GTGTACCAGT CTGGCATCAA CTACGTGGTG GACATCCCCG Exon 35
29341 AGCTGGGTGT CCTCGTCTCC TACAATGGCC TGTCCTTCTC CGTCAGGCTG CCCTACCACC
29401 GGTTTGGCAA CAACACCAAG GGCCAGTGTG gtgagttccg tgacccccat ggcccccgag
29461 gcccccacgg ctcccaccgt cccctgtgcc cccatgtcct gccccagggc gggtggccag
29521 gccaggctga ggctgaggct gcgtgtaaac acccatgggc ctggctgtgg gcctcttgcc
29581 ccgctgctcg gggctgctgt ggccatcacc cgggttcagt ctctgtgagg agccaacagg
29641 agggggcctg gcctggtctc tgccctcggc cctggctggc cggtcctggg catctgggct
29701 ggagaagggc agggcttacc ctgtctgcaa cgtggcctct ctcactgata cagGCACCTG
29761 CACCAACACC ACCTCCGACG ACTGCATTCT GCCCAGCGGG GAGATCGTCT CCAACTGTGA
29821 GGCTGCGGCT GACCAGTGGC TGGTGAACGA CCCCTCCAAG CCACACTGCC CCCACAGCAG Exon 36
29881 CTCCACGACC AAGCGCCCGG CCGTCACTGT GCCCGGGGGC GGTAAAACGA CCCCACACAA
29941 GGACTGCACC CCATCTCCCC TCTGCCAGCT CATCAAGGAC AGgtgacccc gcccaggcct
30001 gcctgtggcc acgacaccaa taagctgagg gcctctgtgc cccagccccc agctcttgca
30061 aagaggaagg aggcagcgcg tggggcctgg cgctggggct gggaaggcac ggagccgcgg
30121 aaccaggatc aggcgctagg tcgccgtggg gtccaggacc caggcccttg ggttccacgg
30181 ggctgagctg ctacgtgcgg cctgtgcctt tgctgaactc cagtctctcc tggctcccgg
30241 gaaggtgcag ggctggccga gtgtgaggcc cggagtaaac cagtcaaccc aggacagagc
30301 tcagggctga tattgggagg gcagatttgg gctttgacag agagggggtg ctcctaacgc
30361 tggcagtcat ggggggtcag catcctgtcc ctggaagtat aggggccagg tataggctgg
30421 gtgtccatct gccagggttg ctggaggggg tcctgaagct gatgaccaca tagacgtggt
30481 ttctatctct gggagccggg ctgcagagcc accttgctcg gccatccctt ggtctgtccc
30541 tgagctgtcc ccctggctgg cctgtccctt gaccctccat cagccacagg cgcctctctg

30601 gcgggtgccg gactccagga ggacagtccg ggcagagacg ctggggtaga gagcagggga
30661 gaggcaggtg ccacctgagt gtgacctgtg cctctccctg cacagCCTGT TTGCCCAGTG
30721 CCACGCACTG GTGCCCCCGC AGCACTACTA CGATGCCTGC GTGTTCGACA GCTGCTTCAT Exon 37
30781 GCCGGGCTCG AGCCTGGAGT GCGCCAGTCT GCAGGCCTAC GCAGCCCTCT GTGCCCAGCA
30841 GAACATCTGC CTCGACTGGC GGAACCACAC GCATGGGGCC TGCTgtaagt gcccatctgc
30901 ccctgccctg gagctggggg cctgcaggcc agacgtggtc tctaggctct gccaggtgct
30961 gtgcccagcc tgaagctaga cctagatggg ctgcggccag ggatgcagag atggcgggtg
31021 tgagaccagg gctggggcca tggggtgggg aaggccaggc tggaggggct gaggtgctgg
31081 ggcttctgcc agcatcgcta aatgcaactg ggtgcccacc acccagctcg ggacaacctc
31141 gagggtggag gttgatgccc aggcagctgg tcaccctcct ccgtgtgtgg ggcactgggc
31201 agctgtcact caaggggtc caggctcctc cgcctgacat gaggcagccc tctgacctct
31261 gcccatgtcc ctcagTGGTG GAGTGCCCAT CTCACAGGGA GTACCAGGCC TGTGGCCCTG Exon 38
31321 CAGAAGAGCC CACGTGCAAA TCCAGgtatg ttgtttgagg gtccaccagg accgtgggct
31381 cgccttctgc agtgcggagg gtggcatcat ctgggcatag cagtcccacc tgccagctcc
31441 ccagccccac cccacctgtc tgacaatgcc ctcccgcccc cagCTCCTCC CAGCAGAACA Exon 39
31501 ACACAGTCCT GGTGGAAGGC TGCTTCTGTC CTGAGGGCAC CATGAACTAC GCTCCTGGCT
31561 TTGATGTCTG CGTGAAGACC TGCGgtacgc cacccactca cactgtcccc tcctgcctcc
31621 ctcctgcctc ctcctgggtg tccacggagg ctgggaccag gacgctgacc accccccacc
31681 tctgatccct gttgcacaag gactctgcta acacaacttg tctcctgggt gtccatggag
31741 gctgggacca ggaggctgac cacccccacc cctgctccct gctgcacaag gactctgcta
31801 acacaacttg tttcttccct cttcctagGC TGTGTGGGAC CTGACAATGT GCCCAGAGAG Exon 40
31861 gtaggcccca ccgtgttgct gggggatcct tccacaaatt ctgaattctg ggagtgagg
31921 gatggacatg aaaacctgga gcctcaaaga ttgaggaatg aggtcatcta agtcctggat
31981 ggctgagttg gcatggacac cacccactca cccacccatc cttccaccca cccactcatc
32041 cacctgtgca cccatctacc cactcaccta cccctccatc cttccaccta cctagtcatc
32101 acccactcat ctatgcaccc ccacccaccc actcatccat ccatccatcc accatccacc
32161 tacccaacca tccacccatc catccaccat ccatctacca tccaccatcc acccaaccat
32221 ccaccatcca tccatccacc atcatccatc taccatccac ccacccacct atccatccat
32281 ccatccacca tctgtctacc atccacccac ccactcatcc atccatccat ccaccatctg
32341 tctaccatcc acccacccac ctatccatcc acccatccat ccatccatcc atccatccat
32401 ccatccatcc atccacccac catctgtcta ccatccaccc acccacctat ccacccatcc
32461 acccacccat ccatccaccc aaccatccac catccatcca tccatccatc catccaccat
32521 ccatctacca tccaccctcc catccatcca cgcatccacc caaccatcca tccatccatc
32581 caccatccac ccaccatcca cccatttatc catccattct ccctccctcc attcaccacc
32641 cattggtcat atgatactct gtctagaagc tctgacatga catcttggcc acctctgtgc
32701 tgcccatgcc tcctacctgt ggtagcagcc atgtggatga ttccttagct aaattctgta
32761 caaacctgag aggcctgagt ggagaatttg ccacgtgcca agcccctgct tgtcgatgct
32821 ggtgagcagg taatggcttt gtgatatcag tgaatgagca gctactgtcc tatcccagaa
32881 cctgcctggt gtgctcagaa gtgaggaggg acatggtttt cccccaggat ccctcagcac
32941 tctgctcagg gtggctgttt ctccccgctg accacagctg cagctccggg gctgtggtga
33001 ggtggggcct gcctggtgcc acctgtcctc tctactcacc cttctttccc tgcagTTTGG
33061 GGAGCACTTC GAGTTCGACT GCAAGAACTG TGTCTGCCTG GAGGGTGGAA GTGGCATCAT Exon 41
33121 CTGCCAACCC AAGAGGTGCA GCCAGAAGCC CGTTACCCAC TGCGTGGAAG ACGGCACCTA
33181 CCTCGCCACG GAGGTCAACC CTGCCGACAC CTGCTGCAAC ATTACCGTCT GCAgtaaggc
33241 catcccctgg ggcccatgcc acctctcagg ggtgcacaca tccctgtagg ctgggctgcc
33301 tgctgtcccc tccttggcaa gtgaggaaac agctggcttg gggcctctg ctgtgcccct
33361 tgagagggct tgggaggggg ccgctgggcc cagtccaggc atccctgctg cagggcctga
33421 cctgggtggg gaggggaccc ttggaggtgc tggaggcccg accctgtgca gtggccccgg
33481 gggctttgcc tgggaggagc caccctcacg gccgcgtgcg caccctgtct tcagagtgca
33541 acaccagcct gtgcagtggc cccgggggct tggcctggga ggagccaccc tcacggccgc
33601 gtgcacaccc tgtcttcagA GTGCAACACC AGCCTGTGCA AAGAGAAGCC CTCCGTGTGC Exon 42
33661 CCGCTGGGAT TCGAAGTGAA GAGCAAGATG GTGCCTGGAA GGTGCTGTCC TTTCTACTGG
33721 TGTGgtaagc agggctggtg ggcagggcag ggaggaggct gccgcccggg gtggggtggc
33781 tgtaaggggg ttggctccct cctgggggtc tcagattctg gggacacaga tggctgtacg
33841 cttggctgat gcacccaccc cagccctgag cgctcgctcc atccactggg tgtgcaccgg
33901 gagtgggggt ctggccaggt ggccgccccg gggcagtctc caacgaacgg ccttctccgt
33961 tctttctccc aagAGTCCAA GGGGGTGTGT GTTCACGGGA ATGCTGAGTA CCAGgtgagc Exon 43
34021 cctgggctgg gtgagaggga ggaggggagg aggtcggctg cagcgtgggg gtcctggcag
34081 gctgttgggc tggctgggat gctggagagg cccctgcctc atgtctctcc ctgtgcccga
34141 agCCCGGTTC TCCAGTTTAT TCCTCCAAGT GCCAGGACTG CGTGTGCACG GACAAGGTGG Exon 44
34201 ACAACAACAC CCTGCTCAAC GTCATCGCCT GCACCCACGT GCCCTGCAAC ACCTCCTGCA
34261 GCCCTgtaag cggccaccct cctccttcag cctgcccttt tccctcctcc cagacaagca
34321 cccgggccca tgtctgcatc gtgacccttt ctttcctcct ttcaacgcca acctgtccct
34381 gtccccacct ctccatcctg acacctgccc agcctggggc ctcctccagg tgggggggtc

```
34441 tcggcagccc tgcaggcttt gtgtggtgtg gggtacagcc tgggagttca gttgcagtgg
34501 cgtgtctatg tgcgcagGGC TTCGAACTCA TGGAGGCCCC CGGGGAGTGC TGTAAGAAGT   Exon 45
34561 GTGAACAGAC GCACTGTATC ATCAAACGGC CCGACAACCA GCACGTCATC CTGAAGgtag
34621 gtgtgcactg ccggccccga cgcggccggg ttgcttgagc ccagggcaag gcgcgggcca
34681 cccaggatcc cccagctgag tcctcccagt cctgggcgca gctgtgatgg gcgccctggg
34741 gctgccatga caaatgagca ggcgtcttca gggcagaaag ggattctcct ggttctgcgg
34801 cccagaaatc catagagcaa agggcctcag ggctgtgctc cctcggaggc gctaggcaag
34861 gacctttccc agcctctggt cactctaggt gccccttggc tgtgaccacg aggtttcctt
34921 ccctgtgtct gcctctcctc tcccttttaa ggatttaggc accccaagca ggatgatctc
34981 atcttaggat ccttcactta atgacacctt caaagacccc ctttccaagg caggtcacat
35041 tcatagattc agagttagaa cacagacaga cctttgaggg ttgtgtgggc tccaggctgg
35101 tgcctgatgt ggggccccgc ccatgtcact tgtcctgtgg ccctggccct caccaggaag
35161 cctccccggc caggtgtctc cagggtgtct tcctggccgg gctggggctg ggcctgctgc
35221 cctccctcac cagagctccc tgccccacag CCCGGGGACT TCAAGAGCGA CCCGAAGAAC   Exon 46
35281 AACTGCACAT TCTTCAGCTG CGTGAAGATC CACAACCAGC TCATCTCGTC CGTCTCCAAC
35341 ATCACCTGCC CCAACTTTGA TGCCAGCATT TGCATCCCGg tgagttggcc acctggggcc
35401 tggctgtgtg tactctgccg ggagtggggg tgcctggtgt tctgggggc tggggcccca
35461 gtgctgcgac agtgacctcg ggcctggtct gagctgccgc aggaggcttt gcctggggct
35521 ttctgcagca gctaccccg cccacggcat cgtgggaagg tgctctcatc cccaggaatg
35581 tccgggggtc ccgggctcat tctcctttcc ctctagGGCT CCATCACATT CATGCCCAAT   Exon 47
35641 GGATGCTGCA AGACCTgtga gtacagggca cagcctgggg ggtaggcagg gtgggggcac
35701 aagggctggt gccctcagcc ccgcctgggg tggctggagg ctggacaacg gcctctgggt
35761 gggcagtgag ggctgggggc tgaggccgag cctggggagg ggacgcagcg agggagagcc
35821 tcctcgaaga tgtggaggcc ctgccctaag ccgctgcccg ctctccccag GCACCCCTCG
35881 CAATGAGACC AGGGTGCCCT GCTCCACCGT CCCCGTCACC ACGGAGGTTT CGTACGCCGG   Exon 48
35941 CTGCACCAAG ACCGTCCTCA TGAATCATTG CTCCGGGTCC TGCGGGACAT TTGTCATgtg
36001 agtcccaggc tgggagtgtg cctggagggg gtggtggaga ccccagggag gcgagaggcc
36061 agcgctggcc ccggaaggtc acccctcact ccgccctccc cccagGTACT CGGCCAAGGC   Exon 49
36121 CCAGGCCCTG GACCACAGCT GCTCCTGCTG CAAAGAGGAG AAAACCAGCC AGCGTGAGGT
36181 GGTCCTGAGC TGCCCCAATG GCGGCTCGCT GACACACACC TACACCCACA TCGAGAGCTG
36241 CCAGTGCCAG GACACCGTCT GCGGGCTCCC CACCGGCACC TCCCGCCGGG CCCGGCGCTC
36301 CCCTAGGCAT CTGGGGAGCG GGTGAGCGGG GTGGGCACAG CCCCCTTCAC TGCCCTCGAC
36361 AGCTTTACCT CCCCCGGACC CTCTGAGCCT CCTAAGCTCG GCTTCCTCTC TTCAGATATT   3´UTR
36421 TATTGTCTGA GTCTTTGTTC AGTCCTTGCT TTCCAATAAT AAACTCAGGG GGACATGC
```

Figure S6. MUC6 genomic sequence (Reverse Complement).

```
   1 GGCAGCAGCA GGCCTGCCGG GCCCAGCGCG AGCTCCTCTC CACTGTGCAC CATGGTCCAG    Exon 1
  61 CGGTGGCTGC TGCTGTCCTG CTGCGGAGCC CTGCTCAGCG CTGgtgagtg aggtcgaggg
 121 gcgccagaca ctgcggtgcc ctcagagggg ccctgtgtgc gggggtctct gggccttctc
 181 gcctcgacaa ctcggctggc ccacatggac gctgatggac gggaggcccc agctcggctc
 241 cgtgaccagc cccgctgcc aggcccgggt ccccccagca gccgcggtgc tgggctctgc
 301 caattggggt cagggacccc atggggcctg ggcagggacg ccctcctgtg agctggcaca
 361 gcagccagcg ggggaggacg ctcagctgga gtggggtccg gcactcccgc gggaacagtg
 421 ggcaggtgtg ctggtgccca gccctggcgc tctccctccc cgagtggggt cctgagtcct
 481 gagaggtctc tggagtgtgg gggacagagg cacagtctct gctgggccct gggcctcgtt
 541 tcctagtcta cagaagtggg ggacgctgcc tcccctggtg tggccgccgt gggggggaaga
 601 ggggggaagg tgtacggcca tcctgggctc agaaccgccg caggcagcct gccctctccc
 661 ccgcgctccc ctacccagct cgggcaggat ccaggaggga ctgacatcct ggatcctctg
 721 ccaaccgcct gccctgcggc tgctgcaggt gaccatcagt ccctccccag gcccccagaa
 781 ggagccagcc ccaaggggtc cctctgtggc cgcagcctga gacctgggga gacccctctt
 841 gccgaccaag gcaccagccc aagggcctct gtgttccgtg ctcctccttc ctctccaagc
 901 tggcccgacc ctcctccaca gccgggagcc ccctggggtg gacgctggcg tgacagagct
 961 cgccctcgga gctgctgcca acctgggcgc ctcgggctca gagcacagtg ggaccccccc
1021 aggggtgtcc cactgtaggg cgacccaagc ccaccccacc ccttctctcc tttgaagccc
1081 cttccccacc acgccgggca ccacccttca accgtgggag atgtgggggt ctctgagctc
1141 gctccccacc acgccgggca ccacccttca actgtaggag atatgggggt ctctgagccc
1201 gctccctacc acgccgggca ccacccttca accgtgggag atgtgggggt ctctgagccc
1261 tcccagcggt gccctctgct tgggacccca gcccctacct gcacacctgg cacagcttgg
1321 ggacaagtgc caggaagtgg ggctaaactt gcagcagagt gcgaccccta gtccctcggg
1381 ccaccaccct ggagccaggg agggaggtgt ggtgaggccc agagaggctg gaggtctggg
1441 ctgtgggcag tgcagagggt ggagggccaa gtgggggtgt ccacccgtca ccccagcaaa
1501 taatctctga gcgccatctc tgccaacatg ggaggggcct cagccagcag agtgtggggt
1561 cggacagacg ggctgcaggc ctgggccgcg gtgggccggg aggcaggggt gggcggccgc
1621 caagcgtgca gaaggaacag tgcgcacctg ccgcctcgtg ctcgtccagg ggctgagagg
1681 ccgggggtgg cggaccaacc cgcccagatg cccatgcgcc tggccaaggg atcagccgag
1741 gggaagcaca gccagtctgg gcaggaacgc aggagtctcc cgctggactt cagagctgga
1801 ggcagccgac ttggagccag tggactcggg gaacctcctg cgggtggggc agggcgaggg
1861 cggcgggggg cgcagggggt gcagcaatga ggcacccacc cggggcagtg aagccagcct
1921 ggcagggctg agggcaccga aacacaggcc cgaggtgtct gtgctacagc ggggacggcc
1981 cccaagtgcc accccccatc ggaaaggtgg agaaactgag ttgcaggggg cattgcccag
2041 gggagaccgg tcacagctct gcagcctcca gaggtgctgg cccaggggca ggcagccaac
2101 tgctcctccc gctcagagct ctcccacccc tcccccacca gaggccagcg agggccccat
2161 cccaggaacc actgagttcc atggctgagg gttcggtgtg ctgcgcactc ctgcagcagg
2221 tgctctgagc cctgtggatc atccctggaa ggaatggcgg ggggctgagg accatgacac
2281 ctactgggac ccccaggagg gggatgagtg agggggtagg tgctcgccag gctcagggcc
2341 cagcatgagc cacccccaca ccagtctgga gtaaactcag cttacactgg ggataagcca
2401 ggtgcaaccc atcagaaaac gatctcaagg ccaaagggga ccacagccaa atatgtgtcc
2461 cacatagcca aggttggcat tcttcttaaa tccagtcact caaataccca acctcctgcc
2521 tgtgccagac aggactcacc caggcggcca aggggcaggc agcctggctc ctccctggac
2581 cgcggttaac accatctccc agctgacagg cccctgcccc tgcgtcaccg gagacacagg
2641 tgtccagcgg taatgggggc agcatcaagt aagtcccaga aagtgggcag agcctggggt
2701 gagagcacag gtgaggagtg gcaggaatgg gatctctgag ctacatctgg aaggaggtgc
2761 tgtgagtgcc ctgctaacca atgggcaggg gcaaggaggg tcccggcccc aagaagggcc
2821 tggagaggtc acgggtcctg ctcaggtgcc gcaccaccac tcagacatcg ggggctcgtg
2881 ggagccacag gaagggtcat agtctcggag gcagggtccg gggagttacc cagaggtggg
2941 aaaagtctcc ctctgggtac agggggtggg acgtgggggg tgttggcccc accccagcct
3001 ctgggagtca gggtgggacg tcccccgctg ccctgccctg gagtcaccgt gactgggcta
3061 tctggatgtt tctctgacaa cagtggcctc agtgccaggc ctgacatcaa ggctgtctca
3121 aggctgacgt gcaggaaagc ggccatgttt agagccccgg agccaccgag agcagggaga
3181 accggctgtg gggccttggc gcagacccct cagggaaacc aggcggccaa gctggggagg
3241 cgggcacagc ccactttgtt ggctccgccg ccaagcccct gccacccgca ccagcccctc
3301 ctagccccac ccagaagccc cagggatgag taccctgcac acagggtgcc cgccatctgc
3361 cgaaccccga ggggctgacg gctgaactgg gactgagtgg acaaacgagg gagtgaacga
3421 gagcagggaa ggaagctggg gctcgtgaag gacagcatgg gaggccaagc gcttggcttg
3481 ctgttgaggc ctcgagcccc acgggccatg gacacttctc gcacgccgag tgtgtgcagg
```

3541 gaaacgggcg gagcagccct gagcagaggt gagcggcgcc ctcagggacg acggggtagc Exon 2
3601 cccacaccga ctgcgggtcc cgtccacaca gGTCTGGCTA ACACCTCCTA CACCAGCCCA
3661 GGCCTCCAGA GGCTGAAGGA CTCTCCACAG ACAGgtaaga acacagcggc cactgatcct
3721 gccgcccaga ccacccaggc ccggtgtgca ggagagaggg gcccggagcc caggtcaccc
3781 ccaagacaca cacatccaac acacatatat gcaccaacat gaacacacat gcacatacac
3841 ccaacacaca cacataccca atatacacat gcacacacac ctgacacacg catacacatg
3901 tacatgacac atgcatgagc acacgtcaca cacacaccca acactctaca cagacacaca
3961 acccaacaca catgcacaca cacccaacac acacaaccta cacacaaaca cacctgacaa
4021 acacatgcac acacacccaa cacccatgca cacacatccc agacatgcaa acatgcacac
4081 ttacaacacg cacgacttac acatgtgcac gcacccaaca cacacacacc ctacacacac
4141 atgcacccta tgtacacacc ctacacacct gagacacgca cccaacacac acatacacac
4201 cccgaatgca cacacatgca ctcgacacac atgcccagct cacacaaaca cacatgtgca
4261 tgcaacacaa tcacacacac ccaacacaca tgcaggtgta tatctcacac gcctacacag
4321 ccctccaaca cacacacacc cacaatgcac acatggacac acctgacacg tatgcacaca
4381 cccgagacac gcacatacgt gcacacacgt gcacacacac aacacacata cgtgtaccca
4441 cacacacgtg tgtgcacatg cccacagcgg tgctgactcc tggccaggca tataccatag
4501 cactcaggtt catcggatgt ctggggtttc cacagcctgg ccccaactca aaaaccctgg
4561 cccagggaga cgagtgacct ctctgcctgc cccagccctg cctggccagg atggtgggga
4621 cacagggctg tgtgagcact gctctctctg tagCCCCGGA CAAAGGCCAG TGCTCCACGT Exon 3
4681 GGGGGGCTGG TCACTTCTCC ACCTTCGACC ACCACGTGTA CGACTTCTCG GGGACGTGCA
4741 ACTACATCTT CGCGGCCACC TGCAAGGACG CCTTCCCCAC CTTCAGTGTC CAGCTGCGGC
4801 GAGGCCCAGA CGGGAGCATC TCGCGGATCA TCGTGGAGCT GGGGGCCTCC GTCGTCACTG
4861 TGAGCGAAGC CATCATCTCA GTCAAGGACA TCGGgtaggt caggtgggcc ggggcccggg
4921 ggctcggggg ccggaggact gaggatcgac caccgacttc ccttgcccca cagGGTCATC
4981 AGCCTGCCCT ATACCAGCAA TGGACTCCAG ATCACACCCT TCGGCCAGAG CGTGCGGCTG Exon 4
5041 GTGGCCAAGC AGCTGGAGCT GGAGCTGGAA GTCGTGTGGG GTCCTGACAG CCACCTCATG
5101 gtgaggagag aagggccagg gtgggcctgg agacccgcag cacgggggggg caggtgggac
5161 ttgggagggg ctgccctcct tgggggtccc agccagccct caggctgcca tgcagccagg
5221 cccttcgtgc caagcagtgc tgacccctga ccagcaacat gagccaggac tgggttttgt
5281 gggtgactcg gcttctccac tggaaaatta gcctcccata agaagaggca gatgagcagg
5341 tggggggatg gtgggggctc tgggtccagg agcaggaact gagagccctc cacagcagat
5401 gagggggccc ctggcccccg ggcccccagc agagccgtct cccgcagGTT CTGGTGGAGC Exon 5
5461 GGAAGTACAT GGGTCAGATG TGCGGGCTCT GCGGGAACTT TGACGGGAAG GTGACCAACG
5521 AGTTTGTCAG TGAGGAGGgt aggtgggggc agggctgggg ggcctctggg gggcctctag
5581 gtgggggggca gggcaggggg gcctctgagg cccagccccc acggtgctga cccccattct
5641 gcaccccagG CAAGTTCCTG GAACCCCACA AGTTTGCTGC CCTCCAGAAG CTGGACGACC Exon 6
5701 CCGGCGAGAT CTGCACCTTC CAGGACATCC CCAGCACCCA CGTCCGGCAG GCCCAGCACg
5761 taagcaaggg ggctccaggt ggggctgacc ccaggtctga cagggtcgcc gagaccacca
5821 agggcccccca caagcagccg catcacagac gccctgcccg acctccccag tcgtggtggc
5881 atggggtgtg gcctttgccc ccatgaccct gagtggcccc cccagGCCCG GATCTGCACC Exon 7
5941 CAGCTGCTGA CCCTGGTGGC CCCTGAGTGC AGCGTGTCCA AGGAGCCCTT CGTGCTAAGC
6001 TGCCAGGCGG ACGTGGCCGC AGCCCCCCAG CCAGGCCCAC AGAACAGCAG TTGTGCCACC
6061 CTGTCGGAGT ACTCCCGCCA GTGCAGCATG GTGGGCCAGC CGGTCCGCCG CTGGCGGAGC
6121 CCCGGCCTGT GCTgtgagtc cagggaaggg agagggaggg gcaggaaggc cgagggctcc
6181 agacccagct ctcccagccc ctggactgcc tgacgtgacg cctgcatccc tcctggtctg
6241 agagacagga gatcctcgct gctgcttcta gatggaggaa gggctgggct aagtcctcca
6301 tcaagctggg gtggggcagg gaggggtggg gggggtggga ccctctcacc ggagccgcgt
6361 gtgcccacag CCGTGGGTCA GTGCCCGGCC AACCAGGTGT ACCAGGAGTG CGGCTCGGCC Exon 8
6421 TGCGTGAAGA CCTGCTCCAA CCCGCAGCAC AGCTGCTCCA GCTCCTGCAC CTTCGGGTGC
6481 TTCTGCCCGG AAGgtgaggg cagtcgcagg cactctctct ccccgggacc cctagagaga
6541 acccagagac aaggggacgt cagaccccac ccaccccgac ccaggtcacc ccaacattct
6601 gctgcctgag tcccagctct ggcgcctctt gccctgggac aggcttttcc cgcctggacg
6661 cttacagcct gcaggtcccc cagccacggc cacccctgca aacccacggg cggtgagggc
6721 tgctcactgc ggtctaggag ctgccgaggg aaggcagggt ttgagccggg cccaagggac
6781 agttacacgt ccctgcctgc tggaggccac cactcaccag gaacctgggc cgggcacatt
6841 ggcacgaggg gctgtgcacg cccagggcct gacatagagt tgcgaatgca agtgcagctg
6901 gagagaggtc atggcccagg ctagaggggg cgtctccctg gagccaggct ggaggggcgt
6961 ctccctggag ccaggctgga gggggcgtct ccctggagcc aggctggagg gggcgtctcc
7021 ctggagccag ggaggggaga gtgggaggca gtcagtccca cccaagcccc aagaagaccc
7081 tctcctgcag GTACGGTCCT GAATGACCTC TCCAATAACC ACACCTGCGT GCCCGTCACC Exon 9
7141 CAGTGCCCCT GTGTGCTCCA CGGCGCCATG TATGCCCCCG GGGAGGTCAC AATAGCTGCC

7201 TGCCAAACCT Ggtgagtgag gcgccggagg gggctctggc cggccggcag gggttccact
7261 ggcctagggc aggcaggggt gtgctgtctg gctccagcgg ccctgcccac catgccgcta
7321 cccccacgcc cacccacagC CGGTGCACCC TGGGCCGCTG GGTGTGCACG GAGCGGCCGT Exon 10
7381 GCCCCGGACA CTGCTCCCTG GAAGGTGGCT CCTTTGTTAC CACATTTGAC GCCAGGCCCT
7441 ACCGCTTCCA CGGCACCTGC ACCTACATCC TCCTCCAGGt aggacgagcc ctgtggcccg
7501 tggggagggg cgctcccggt gaccccaccc gtgtctgagc cccgccccct cgcagAGCCC Exon 11
7561 CCAGCTTCCC GAGGACGGTG CCCTCATGGC TGTGTACGAC AAGTCCGGCG TCTCACACTC
7621 CGAGACCTCC CTGGTGGCTG TGGTCTACCT CTCCAGGCAG gtaaggcctt tcctgcgccc
7681 atccctgcca gcagggctcc gctccctggt gcctgcaccc tgactcaggc caccctctct
7741 ccagGACAAA ATTGTGATCT CTCAGGACGA GGTGGTCACC AACAACGGAG AAGCCAAGTG Exon 12
7801 GCTGCCATAC AAGACTCgta cgtcctggcc cagtctgtgg ccccccagag ttgtgccttc
7861 ggggcgggct gcagtgggtg gggagggagc ccagacggcc cactgagcac tgcgtcctgc
7921 agGCAACATC ACGGTCTTCA GGCAGACGTC CACCCACCTC CAGATGGCCA CCAGCTTCGG Exon 13
7981 GCTGGAGCTC GTGGTCCAGC TGCGCCCCAT CTTCCAGGCC TATGTCACTG TTGGGCCCCA
8041 GTTCAGAGGT CAGACCAGAG gtgagtccct gcctctccag gtggccctgt tgcctcatcc
8101 ctacagggtc tggccatgac caggggagga gaaaggtcca agagaggttc acacaaccct
8161 ctgtgtccgg ggagccccca ggggtaacag gcacgtgtgg cccacgcagc ctctcaccca
8221 gggtgctgtt agaggaagga cgggagctcc agggcgtgtg ggcaggaatg ggtgcaggag
8281 ggatgggttc aagtcgagct cacgccccgc cggctcagGG CTCTGCGGCA ACTTCAACGG Exon 14
8341 GGACACAACG GATGACTTCA CCACTAGCAT GGGTATCGCC GAGGGCACCG CCTCGCTGTT
8401 TGTGGACTCC TGGCGGGCGG GGAACTGTCC GGCCGCTCTG GAGCGTGAGA CTGACCCCTG
8461 CTCCATGAGC CAGCTCAACA gtgagtgtcc ggccccccac tcccctggct gccccccccag
8521 cgcccacagt tctgacagac ccctggactg gaccacaggc ccagctgcca gggtgggggg
8581 tccctgggag gagccgtagc tggaatggga ggggccggga ctcacgcccg ggcctgtcat
8641 ccccagAGGT GTGTGCAGAG ACCCACTGCT CCATGCTGCT GAGGACAGGC ACGGTGTTCG Exon 15
8701 AGAGGTGCCA CGCCACAGTG AACCCTGCAC CCTTCTACAA Ggtgagggcc cgagggcgtc
8761 ttgggagggc tgcaggggag gctgtggtgg ctgacaaggt ctcaggcacc caagggcata
8821 ggtttgggac ccccatgtcc ccagggcagg gtgcctgcca ccagcccatg ctggctctct
8881 gcccgcagAG GTGCGTGTAC CAGGCCTGCA ACTACGAGGA GACCTTTCCC CACATCTGTG Exon 16
8941 CCGCCCTGGG CGACTACGTA CACGCCTGCT CCTTGCGGGG CGTCCTGCTC TGGGGCTGGA
9001 GAAGCAGTGT GGACAACTGC Agtgagtgcc cgacggggct taagcggggt cacggcaggc
9061 tgggctcacg agggggtgtc ctgggacccc aagctctgag gctctgcagt gcccggcagg
9121 caggggaacc cgagattgcc ctcccggccg ccccctcccg gagagtctga tgcccgggtc
9181 ccccacagCC ATCCCCTGCA CGGGTAACAC CACCTTCAGC TACAACAGCC AAGCCTGTGA Exon 17
9241 GCGCACCTGC CTGTCGCTGT CGGACCGTGC CACCGAGTGC CACCACAGCG CCGTGCCCGT
9301 GGACGGTTGC AACTGCCCCG ATGGCACCTA CCTGAACCAA AAGGGCGAGT GTGTGCGCAA
9361 GGCCCAGTGC CCGTGCATAC TGGAGGGTTA CAAGTTCATC CTGGCCGAGC AGTCCACTGT
9421 CATCAACGGC ATCACCTGGt gagggaccgg gcaggggcca ggcggggggt ccctgccagg
9481 tcccggggtc tcacgcagcc tctctcttgc agCCACTGCA TCAACGGGCG GCTGAGTTGC Exon 18
9541 CCGCAGCGGC CACAGATGTT CCTGGgtacg tacagcagcg ctggccgcag gctgggactg
9601 tgaggggccc cggcttcctc tgagtgtcct gaccgcgcac ccctctttcc tgcagCCTCC
9661 TGCCAGGCCC CTAAGACCTT CAAGTCCTGC AGCCAGTCCT CCGAGAACAA GTTTGGGGCA Exon 19
9721 GCCTGTGCCC CCACATGCCA GATGCTGGCC ACCGGTGTTG CCTGCgtaag ggggcgcgcg
9781 aggagcaggg acaatgcccg ggccccagct gaaccatgag ggggtttcca cagaactccg
9841 ggagacctgg ccgcattctg ccctgcagcg ggtgcctggg gctggccacg aggtggaagc
9901 ggccctgtgg ccaggcagct ggaggccccg ggggctcgtc tgcagggctc cctaaggaca
9961 gtgggcacca gctcggggag ggtgggctgc ccatgtgtgg gagtgggtac agcagcccag
10021 ccgtgtgcac tgggtcccag gaccttccac gaggtggggg aagctttgct aaggcagtaa
10081 gcccagctgc ccacaaaggc cacagagcag ggctgtattc agggagagga cctgggagac
10141 acgccgggca gtctcagagg ggcccagggc tgggcagcag ctggctgagg ccaccctccc
10201 aggagctgct gacctgcctc cctccagGTG CCCACCAAGT GTGAGCCTGG CTGTGTCTGC Exon 20
10261 GCCGAGGGCC TCTACGAGAA TGCCGACGGG CAGTGTGTGC CCCCCGAGGA GTGCCCATGT
10321 GAGTTCTCGG GGGTCTCCTA CCCTGGAGGA GCTGAGCTCC ACACTGACTG CAGGACCTGg
10381 tgagacaaag cctcgcttca gacgccctgg gctcctgggg ccatctgggg caggcggagg
10441 ctgcccgggg tggggctgtc ccagaggcgg gggcgtccag aggtgcagtc tggcctctcc
10501 agggaccaca cccagtatgg caggagggcc gcaggcccac cacccacacc tgctgtctcc
10561 acagCTCCTG CTCAAGGGGG AGGTGGGCCT GTCAGCAGGG CACCCACTGC CCATCCACCT Exon 21
10621 GCACCCTCTA CGGGGAGGGC CACGTCATCA CCTTCGACGG CCAGCGCTTC GTATTCGACG
10681 GCAACTGCGA GTACATCCTG GCCACGGtaa ccatcgggtg ccaggccgca ggggccgggg
10741 acccagaggc acggcctcca gggctcctcc tcagcgccct ctccctgcag GACGTCTGTG Exon 22
10801 GTGTCAACGA CTCACAGCCC ACCTTCAAGA TCCTGACAGA GAACGTCATC TGTGGGAACT

```
10861 CCGGGGTCAC ATGCTCACGG GCCATCAAGA TCTTCCTGGG Ggtgagcagc cgggcagact    Exon 22
10921 ctggcagggc aggacgggcg gtaggggggcc ctgccacaca gctggatccc gcgcactgga
10981 gcctccaggt cctgccccga gatgcagccc tccccgcctg cctctccttg aggggtgcct
11041 gctgcccctc cctggccaga cgctcaggac cacgggtgct tgtgccccac aaggtttcag
11101 ggactgcaga atgtctcccg ggcagagggg agggactctc caggaagccc gagcttcagc
11161 cctcagccct gttccaggca tgccaggccc ttctgcccca agctggctct cagcaggcgc
11221 tcctgtgcat ggccttaaca gacctgggca gctctgtctc tgagagctcg gtccagcacg
11281 gctgtgccag gggccgggggg agacagacag gcagtcctat gccaacccca ccccacagGG
11341 CCTGTCCGTG GTGCTGGCGG ACAGAAACTA CACGGTCACC GGGGAGGAGC CCCACGTGCA    Exon 23
11401 GCTCGGGGTG ACGCCGGGTG CGCTGAGCCT TGTCGTGGAC ATCAGCATCC CCGGGAGGTA
11461 CAACCTGACG CTCATCTGGA ACAGGCACAT GACCATCCTC ATCAGGATCG CCCGTGCCTC
11521 CCAGgtaccg cacgccctct gcctcctccc aggccctccc tgatgcaggg gtggagacag
11581 atggcacggc ccctgggccc ggcctgatgc caccgtctgc agGATCCCCT CTGCGGCTTG    Exon 24
11641 TGTGGCAACT TCAACGGGAA CATGAAGGAC GACTTCGAGA CGCGCAGCAG GTACGTGGCA
11701 TCCAGCGAGC TGGAGTTGGT GAACTCGTGG AAGGAGAGCC CGCTGTGCGG GGACGTGAGC
11761 TTCGTGACAG ACCCCTGCAG TCTCAATGCC TTCCGGCGCT CCTGGGCCGA GCGCAAGTGC
11821 AGCGTCATCA ACAGCCAGAC CTTTGCCACC TGCCACAGCA AGgtgggcac cgggcacgag
11881 ggctgtgcgc ctgcccctcc acaaatgtcc ggtgggggcg ggggaagggg cggggaagcc
11941 aggcacggtt ctccctccgt gggatcccag accagccagg tcagggtgca gagagtttca
12001 gaccctctgc ctgagtgcag ggccctgatg tggggccgac tcgtgcactg tggctgttaa
12061 gcagcccccc ggcctccacc caccagatgc caatagcacc cccagacagt gccacgagtc
12121 ccctggggca gcatcgcctg gtgggccccc tgctcaggag ctggtggtca gggagacctc
12181 caggatgcag ggcgtgcact tcccagccca gggcgtgatc cggccatcgc ggcgcagtaa
12241 gaactgagag gatgggggca ccccgcgggg gaacagcatg gcactggccc gctgaatgtg
12301 agcgtgacgt ggggggagca gagaccctgg gtgggactgc agtggccgtg gttagcggca
12361 gcggcatctt ggtcccgatg gcgggtctca aggcgtgagc tcgggtcagg cctccgtggc
12421 agcatcggct ccgggagcgt ggctagcggt cagggctgag gtccacgtgg tgccgctcac
12481 ccagtcccag cgcccttacc gccacactcc ggcctgacca cactgagggc cctgcaggtg
12541 gcaaagcctg atgccccgcc atcccccggc agccagacac tgactggcac acacccctgc
12601 agGTATACCA CCTGCCCTAC TACGAGGCCT GCGTGCGCGA CGCATGTGGG TGTGACAGTG    Exon 25
12661 GCGGGGACTG TGAGTGTCTG TGCGATGCCG TGGCTGCCTA CGCCCAAGCC TGTCTGGACA
12721 AGGGTGTGTG CGTGGACTGG AGGACCCCGG CCTTCTGCCg tgagtgacca cccctcccac
12781 aggttgctcc agcccctgcc acccagcgcc actaagggcc ggcgcagggc tgcccttgcc
12841 cacaggcccc tctccctgga caggcgccgg gctgtgccct gcggggccac cctggccctc
12901 ccgcagcccc cagctgccca ggggaggctt caccaagagg gcccagacct tccccgccca
12961 cactcaggtt ccatgcatgc ccaggggaac caccgggtca gggccagggc cagggccagg
13021 aaccccacca gctgacagct cccttcctcc cagCCATCTA CTGCGGCTTC TACAACACGC    Exon 26
13081 ACACGCAGGA CGGCCATGGC GAGTACCAGT ACACACAGGA GGCCAACTGC ACGTGGCACT
13141 ACCAGCCCTG CCTCTGCCCC AGCCAGCCAC AGAGCGTCCC AGGCAGCAAC ATCGAAGgtg
13201 ccaggtgacc gggaggcaac gcagatacac agggggaccc attcactcat tcatgcacat
13261 tcattcattc acacgcattc attcattcac acattcattc atgctcatta attcacacat
13321 tcattaattt gcacacattc attcacacac actcacgcac attcacattc attcatgcag
13381 attcatgcat tcattcacgc atattcattc attcacgcac atttgttcat ccactcacac
13441 acactcatgc acattcattc acacattcat tcacgcagtc actacacaca cattcattca
13501 tgctcattca cgcagtcaca ttcccgcaga ttcatgcaca ttcattcacg cacattcact
13561 cacgcacatt cacgcacact catgcacatt cactccattc atccattcat tcacccacac
13621 acactcattc aacatattca cgctcattca cattcattca cgcacattca ctcaacacat
13681 tcacattcat tcatgctcat tcacacattt actcacccac attcatgcac attcacgcac
13741 tcatgcattc atttacgcac atttgttcac tcacgcacat tcactcatgc acattcacgc
13801 acattcattc aacattcact cacgcacatt cacccatgca cattcattca cacatattca
13861 ttcattcatt catgtttgct tattcattca tgcacattcg ctcatacaca cacgcacatt
13921 catgcattca tttgcacatt cattctttca cacacacagt cactcagcag atgggtcctg
13981 agcacctctg tcctccaggc cggctccagg tcaggggacc tggccttaca caaaacaaac
14041 agtcctgcaa cctggagctt ccctcccagc cagggagagg tgcttaagga ggcagaccca
14101 gtgtcggccg aggcgcccac catggagagg agaaaggcag gctggggtgg gagagccaca
14161 ggcttctgtg ccacacaggg tggttggggc ccttggtggg aggcagatgt gagggcgagg
14221 atgggaaagg tgaggagggg agccaggaaa gtgtccagag gagcagccag gagcagcctt
14281 gtgcccagaa cccttaagaa agccagggca gcgggcagag ccagcgagtg aggggcgcgg
14341 gctgcagagg gcagagggag cggtggaggg gtgcggggct gtagagggca gatggagtga
14401 gtgaggggcg cggggctgta gagggcagaa ggagcgagtg gggcgcgggg ctgcagaggg
14461 cagagcaagc gagtgagggg cacagggctg cagagggcag agggagtgag tgaggggcgc
```

```
14521 ggggctgtag agggcagagg gagcgagtga ggggcgtggg ctgcagaggg cagagcgagt
14581 gaggggcgcg gggctgcaga gggcagaggg agcgagtgag aggcgcgggg ctgcagaggg
14641 cagagggagc gagtgagggg caccgggctg cagaggggtg tggggtacac agacttgtgg
14701 ggtggggtgt ggccgggagg gaggggtctt tgtgaggcct gtggctgccc caaggcagag
14761 gtctgaatgc agagggtcct gggattaaca ggtcctggcc caagtggtgg aggagggtgg
14821 tgtctggagc catccggaca cacagaggac agatgtgttg gggctcagga gggcaatgca
14881 gggcacctgc atcggggagc acagaagagg ggccagtgtc tgaggtccag gggaggtga
14941 ggactggaag atggcaccga agccccagcc ccgggtgata agcagggtat gagagaccaa
15001 ggcctgagga aggcatggca ggaaaggagg ggcttagagc aaggtgcgcg ggtgaaactg
15061 taatcccagc actctgggag gccgaggcag gaagatcacc tcaggtcaag agtttgagac
15121 cagcctggcc aacgtggcga agccccatca ctactaaaaa tacaaaaatt agccgggcgt
15181 catggcacat gcctgcaatc gcagctacct gggaggctgg ggcaggagaa tcatgtgaat
15241 ccaggaggca gaggttgcag tgagcgaaga ttgtgccact gcactccagc ctgggcgaca
15301 gagcgagact ctgtctcaaa aaaaaaaaaa aaaaaaagca gagaggaagg aggccgccag
15361 ggcaggaaac acgcaggtgg gcctggctgg ccaccagtca cacagaccct ggccgttctc
15421 caccctagGC TGCTACAACT GCTCCCAGGA TGAGTACTTC GACCACGAGG AGGGGGTGTG   Exon 27
15481 CGTGCCCTGC Agtaagtcca gtcggctgcc ctgagaaccc tgcccctgcc tgcatgcagg
15541 cagaacgcac gtccacatcc cacaaacaag ggagagagcc ctcgggggct gtgagccctt
15601 acgtgagctc tgggactcca ctctccagga ccctggcccc ttgggtctcc agagactcag
15661 gcagcctcca gcccatggca gcagagagaa aaaaacacag cactgaaccc cactgggccc
15721 tcccatcccc tcttccacca gctcccagct ccccctaccc tcaccaggcc tgccagggtg
15781 cctggggtat agagtgggaa tcatcctggg aaggagaaag ggagagaggg gagggtgggc
15841 tcccagcccc tccagtcccc tccaccacag ccttggtgag catctctgac tcaggccctc
15901 ggagctccca gaggccccac ggggtgggga ggcttagaga accacaggga gctgacccca
15961 tcttcttgca gTGCCGCCCA CCACGCCGCA GCCACCCACC ACGCCGCAGC TGCCCACCAC   Exon 28
16021 AGgtaattgc acgcacacta ggtgccaagg tgacgcaggc cctttcctgg gctcccctcg
16081 gttccctgga caaatctgtc gggtgggaag cagggagggg gcaggcagta cctgtgccct
16141 tctcaggtct aacctggcaa tcagagttgg ggaggagctc agcccacagc caggaagcag
16201 cctggtcccc atggccctgg ctggacccac ggatgagcac agagtggggc ctcaacaggt
16261 gacccccgcc atgcaggagg cccagccagg agccaggctg cactcagagt gccacaggcc
16321 agccctcaac tgcacatcat tggcccctgc agggctgccc ggcttcaggg ccaagcaggc
16381 agaggggagg gtgctcccca aggatatgcc ggtaccctgc agccctgatg gcatgtccgc
16441 ccacccagGC TCACGGCCCA CGCAAGTCTG GCCCATGACG GGAACCTCCA CCACCATCGG   Exon 29
16501 GCTTCTCAGC TCCACCGGAC CCTCACCCAG CTCTAATCAC ACCCCTGCCA GCCCCACCCA
16561 GACACCCCTC CTTCCAGCCA CGCTCACATC CTCCAAGCCC ACAGCCTCCT CGGGAGgtaa
16621 ggagcctcca gctgagccca tggagagggc agctgcagga ggtcctaggt acacctctgg
16681 ggtgggctta gggatggccc tgcttcctgg ctcacatctg ccactaagca gattcccagc
16741 gtagaacttc ctatgcttgg gagccaaact ggggattttt cggaaaaact tttaaagaca
16801 agctggtcat ggcatggggc attccttgct gctggctgct gtcactagaa accgtgtggg
16861 acctgcaggg cctctgccag gcagcctgcc cctgccccac accgagcagg gccccatctg
16921 ctgcccaggg accctggagg ggacaggacg acagtccgtt cagctaagca agggtgggcg
16981 caggagcagc cgcagcccca taagcatgtg gtcactgccc cagctctggc caccacagcc
17041 aggagtacgg tgggaggacc taacaaaggc aagaggaaga gcccctccaa ggaggctgag
17101 tcccggacag caggccaggg atcccagaag cagggcaggg ggctgggaca caagccttcg
17161 aaatgcaggc ccacagcaag gggatgttcc gggcggctgt cctctgcagA ACCACCTAGA   Exon 30
17221 CCAACCACGG CCGTCACCCC ACAAGCCACA TCAGGGCTGC CTCCCACAGC CACACTGAGA
17281 TCGACAGCCA CAAAACCCAC AGTGACCCAG GCCACAACCA GGGCCACGGC GTCGACCGCC
17341 AGCCCAGCCA CGACGTCCAC AGCTCAGTCC ACAACACGGA CCACAATGAC ACTACCAACC
17401 CCAGCCACAT CAGGGACAAG CCCCACGCTG Cgtaagtcat ggcgccatgg gatgccagca
17461 ctgccgaagg cacccggtcc caccaccagc tcacattcag tgattcagcc ataaagaaga
17521 ccccgtattt cccagagggc aagcgagaag gcagcccaaa agtgtaggct ggagctggag
17581 gcaaggaagg ccgcctggca ctcacaaagg caggcctggg gagcagaggt gcaggagggt
17641 gggggccacc accacggcca cagggaacca gaaggggata aagtagggcc tgggttccag
17701 tcacagagcg gcagctgcac tgaaggagtc agcacgcagc tcagggcagg atgtggagca
17761 agtccaggtg agatggagac aatggggcag gctggagtgc ccagcagggg ccatgtcaca
17821 ggaacagagg cacagacagg caagaaaaag gtcacataga caaaaggacg ggccagcgga
17881 ggtcagggta gagaaacaaa aacaataacg atgacaactt caccaattcc cacagCAAAA
17941 TCGACCAATC AGGAACTGCC AGGAACAACG GCCACCCAGA CGACAGGCCC ACGTCCAACC   Exon 31
18001 CCAGCAAGCA CCACAGGCCC AACCACCCCA CAGCCAGGAC AACCCACGAG GCCCACAGCC
18061 ACAGAGACCA CTCAAACAAG AACGACTACT GAATACACAA CGCCCCAAAC CCCACACACC
18121 ACACACTCCC CGCCTACGGC GGGGAGTCCC GTCCCTTCCA CAGGTCCTGT CACTGCAACA
```

18181 TCTTTCCATG CCACCACTAC CTATCCAACC CCATCACACC CTGAGACCAC ACTTCCCACT
18241 CACGTTCCAC CTTTCTCCAC CTCCTTGGTG ACTCCAAGTA CTCACACAGT CATCACCCCT Exon 31
18301 ACCCACGCAC AGATGGCCAC ATCTGCCTCC AACCACTCAG CGCCAACAGG TACCATTCCT
18361 CCACCAACAA CGCTCAAGGC CACAGGGTCC ACCCACACAG CCCCACCAAT AACGCCGACC
18421 ACCAGTGGGA CCAGCCAAGC CCACAGCTCA TTCAGCACAA ACAAAACACC TACCTCGCTA
18481 CATTCACACA CTTCCTCCAC ACACCATCCT GAAGTCACCC CAACTTCTAC TACCACGATT
18541 ACTCCCAACC CCACTAGTAC ACGCACCAGA ACCCCTGTGG CCCACACCAA CTCAGCCACC
18601 AGCAGCAGGC CACCACCACC CTTCACCACA CACTCCCCAC CTACAGGGAG CAGTCCCTTC
18661 TCTTCCACAG GTCCCATGAC GGCAACATCC TTCAAGACCA CCACTACCTA TCCAACCCCA
18721 TCACTCCCTC AGACCACTCT TCTCACTCAC GTTCCACCTT TCTCAACCTC TTTGGTGACT
18781 CCAATTACTC ACACAGTCAT CACCCCTACC CACCCACAGA TGTCCACTTC TGCCTATATC
18841 CACTCAACGC CAACAGGCAC GATTGCTCCA CCAACAACAG TTAAGGCCAC AAGGTCCACC
18901 TACACAGCCC CACTAATGAC GGCAACCACC AGTAGGATCA GCCAAGCCCA CAGCTCAATC
18961 AGCACAGCCA AAACCTCTAC ATCCCTCCAC TCACATGCTT CCTCCACACA CCATCCTGAA
19021 GTCACCCCAA CTTCTACCAC CAACGTGACT CCCAAGTCCA CCAGTAGAGG CACCAGCACC
19081 CCTGTGACCC ACACCACCTC GGCCACCAGT AGCAGGCCAC CCACACCCAT CACAACACAC
19141 TCTTCACCTA CCAGGAGCAG TCCCCTCTCT TCCACAGGTC GTATGACTGC AACATCTCTC
19201 AAGACCACCA CTACCTATCC AACCCCATCA CAAGCTCACA TCACACTTCC CATTCATGTT
19261 CCACCTTTCT CCACCTCATC GGTGACTCCA AGTACTCACA CAGTCATCAC CCCAACCCAC
19321 CCACAGATGT CCACTTCTGC CTCCAACCAC TCAACGTCAA CAGGCACCAT TCCTCCACTG
19381 ACAACGCTCA TGGCCACAGG GTCCACACAC ACAGCCCCAC TAATAACAGT GACCACCAGT
19441 AGGACCAGCC AAGTCCACAG CTCCTTCAGC ACAGCCAAAA CCTCTACATC CCTCCTCTCC
19501 CATGCTTCCT CCACACACCA TCCAGAAATC ACCACAAATT CTACCACCAC CATTACTCCC
19561 AACCCCACTA GTACAGGCAC CGGAACCCCT GTGGCCCACA CCACCTCAGC CACCAGCAGC
19621 AGGCCACCAC CACCCTTCAC CACACACTCC CCACCTACAG AGAGCAGTCC CCTCTCTCCC
19681 ACAGGTCCTA TGACTCCAAC ATCCTTCAAG ACCACCACTA CCTATCCAAC CACATCACAC
19741 CCTCAGACCA CACTTCCCAC TCACGTTCCA CCTTTCTCCA GCTCGTCAGT GACTCCAAGT
19801 ACTCACACAG TCATCACCCC TACCCATGCA CAGATGTCCA CTTCTGCCTC GATCCACTCA
19861 ACGCCAACAG GTACCATTCC TCCACTGACA ACGCTCACGG CCACAGGGTC CACACACACA
19921 GCCTCACCAA TGACGGGGAC AACCATTCGG ACCACCCAAG CCCACAGCTC ATTCAGCATA
19981 GCCAAAACCT CTACATCCAT CCTCTCACAT GCTTCCTCCA CACACCATCC GGAAACCACA
20041 CCAACTTCTA CCACCAACAT TACTCCCAAG TCCACTAGTG CAGGAACCAG CACCCCTGTG
20101 GCCCACACCA CCTTGGCCAC CAGCAGCAGG CCACCCACAC CCTTCACCAC ACACTCCCCA
20161 CCTACAGGGA GCAGTCCCAT CTCTTCCACA GGTCCTATGA CTGCAACATC CATCAAGACC
20221 ACCACGACCT ATCCAACCCC ATCACACCCT CAGACCACAC TTACCACTCA TGTTCCACCT
20281 TTCTCCACCT CATCAGTCAC TCCAAGTACT CACACAGTCA TCACCCCTAC CCACGCACAA
20341 ATGTCCACTT CTGCTTCGAT CCACTCAACG CCAACAGGCA CCGTTCCTCC ACTGACAACG
20401 CGCATGCCCA CAGGGTCGAC ACACACAGGC CCACCAATGA CGGGGACCAT TATTCAGACA
20461 AGCAAAGCTC ACAACTCATT CAGCACAGCC AAAACTTCTA CATCTCTCCA CTCACATGCT
20521 TCCTCCACAC ACCATCCTGA AACCACACCA ACTTCTACCA CCAACATTAC TCCCAAGTCC
20581 ACTAGTGCAG GAACCAGCAC CCCTGTGGCC CACACCACCT TGGCCACCAG CAGCAGGCTA
20641 CCCACAACCT TCACCACACA CTTCCCACCT ACAGGGAGCA GTCATGTCTC TTCCACAGGT
20701 CCTATGACTG CAACATCCTC CCAGACCACC ACTACCCATC CAACCCCATC ACACCCTCAG
20761 ACCACACTTC CCACTCACAT TCCACCTTTC TCCACCTCCT TGGTGACTCC AAGTACTCAC
20821 ACAGTCATCA CCCCTACCCA CGCACAAGTG TCCACTTCTG CCTCCATCCA CTCAACACCA
20881 ACAGGCACCA TTCCTCCACC TACAACGGTA AAGGCCACAG GCACAGGGTC CACGCACACA
20941 GCACCACGAA TGACAGTGAC CACCAGCGGG ACCAGCCAAG CGCACAGCTC TTTCAGCACA
21001 GCCAAAACCT CCACATCCCT ACACTCACAT GCTTCTTCAA CACTCCATCC TGAAGTCACC
21061 CCAACTTCTA CCACCACCAT CACCCCCAAC CCCACCAATA CAGGCATCAG AACGCCTGTG
21121 GCAAACACCA CCTCAGCCAC CAGCAGCAGG CTAACCACAC CCTTCACCAC ACACTCCCCA
21181 CCTACAGGGA GCAGTCCCAT CTCTTCCACA GGTCCTATGA CTGCAACATC CTTCCAGACC
21241 ACCACTACAT ATCCAACCCC ATCACACCCT CAGACCACAC TTCCCACTCA CGTTCCACCT
21301 TTCTCCACCT CCTTGGTGAC TCCAAGTACT CACACAGTCA TCACCCCTAC CCACGCACAG
21361 ATGGCCACTT CCGCCTCCAT CCATTCAACG CCAACAGGCA CCATTCCTCC ACTGACAACG
21421 CTCATGAACA CAGGGTCCAC ACACACAGCC CCACCAGTGA CGCCGACCAC CAGTGGGACG
21481 AGCCAAGCCG CGAGCTCATT CAGCACAGCC AAAACCTCTA CATCCTTACA TTCACACACT
21541 TCCTCCACAC ACCATCCTGA AGTCACCCCA ACTGCTACCA CCAAAATCAC CACCAACCCC
21601 ACCAGTATAG GAAGCAGCAC ACCCATGGCC CACACTACCT CAGCCACCAG CAGCAGGCTA
21661 ACTACACCAT TCACCACACA CTCCCCATCT ACAGGGAGCA GTCCTGTCTC TTCCACAGGT
21721 CCTATGACTG CAACATCCTT CCAGACCACC ACTACCTATC CAACACCATC ACTCTCTCAG
21781 ACCACTCTTC CCACTTACGT TCCACCTTTC TCCACCTCCT TGGTGACTCC AAGTACTCAC

21841 ACAGTCATCA CCCCTCCCCG CACACAGATG GCCACTTCTG CCTCCATCCA CTCAACGCCA Exon 31
21901 ACAGGCACCA TTCCTCCACC GACAACGCTC AAGGCCACAG GGTCCACCCA CACAGCGCCA
21961 ACAAGGACGC TGACCACCAG CGGGACCAGC CAAGCCCTGA GCTCATTAAA CACAGCCAAA
22021 ACCTCTACAT CCCTACATTC ACACACTTCC TCCACACACC ATCCTGAAGC CACCTCAACT
22081 TCTACCACCA ACATCACCCC CAACCCCACC AGTACAGGAA CTGGGACACC TGTGGCCCAC
22141 ACCACCTCAG CCACCAGCAG CAGGCTAACC ACACCCTTCA CCACACACTC CCCACCTACA
22201 GGGAGCACTC CCATCTCTTC CACAGGTCCT GTCACTGCAA CATCCTTCCA TGCCACCACT
22261 ACCTATCCAA CACCATCACA CCCTCAGACC ACACATCCCA CTCACGTTAC ATCTTTCTCC
22321 ACCTCCTTGG TGACTCCAAG TACTCACACA GTCATCACCC CTACCCACGC ACAGATGGCC
22381 ACTTCTGCGT CCATCCACTC AACGCCAACA GGCACCATTT CTCCACCAAC AACGCTCAAG
22441 GCCACAGGGT CCATCCACAC AGCCCCACCA ATGACGCCGA CCACCAGTGG GACCAGCCAA
22501 TCCCCAAGCT CATTTAGCAT GGCCAAAACT TCTACATCCC TACCTTACCA CACTTCCTCC
22561 ACACACCATC CTGAAGTCAC CCCAACTTCT ACCACCAACA TCACCCCCAA ACACACCAGT
22621 ACAGGCACAA GAACCCCTGT GGCCCACACC ACCTCGGCCA CCAGCAGCAA GCTACCCACA
22681 CCCTTCACCA CACACTCCCC ACCTACAGGA AGCAGTCCCA TCTCTTCCAC AGGTCCTGTC
22741 ACTGCAACAT CCTTCCAGAC CACCACTACC TATCCAACCC CATCACACTC TCACACCACA
22801 CTTCCCACTC ACGTTCCACC TTCCTCCACC TCCTTGGTGA CTCCAAATAC TCACACAGTC
22861 ATCACCCATA CCCATGCACA GATGTCCACT TCTGCCTCCA TCCACTCAAC GCCAACAGGC
22921 ACCATTCCTC CACCGACAAC GCTCAAGGCC ACAGGGTCCA CCCACACAGC CCCACCAATG
22981 ACGCCGACCA CCAGTGGGAC CAGGCAAGCC CCAAGCTCAT TCAGCACAGC CAAAACTTCT
23041 ACATCCCTAC ATTCACACAC TTCCTCCACA CACCATCCTG CAGTCACCCC AACTTCTACC
23101 ACCAACATCA CCCCCAACCA CACCAGTACA GGCACCAGGA CCCCTGTGGC CCACACCACT
23161 TCGGCCACCA GCAGCAGGCT ACCCACACCC TTCACCACAC ACTCCCCACC GACAGGGAGC
23221 AGTCCCATCT CTTCCACAGC TCCTGTCACT GCAACATCCT TCCAGACCAC CACTACCTAT
23281 CCAACCACAT CACAGTCTCA GACCACACTT CCCACTCACA TTCCACCTTT CTCCACCTCC
23341 TTGGTGACTC CAAGTACACA CACAGTCATC ACCCCAACCC ATCAACAGAT GGCCACTTCT
23401 GCCTCCATCC ACTCAACGCC AACAGGCACC ATTCCTCCAC TGACAACGCT CAAGGCCACA
23461 GGGTCCACCC ACACAGCCCC ACCAATGACG CCGACCACCA GTGGGACCAG CCAAGCCCTG
23521 AGCTCATTCA GCACAGCCAA AACCTCTACA TCCCTACATT CACACACTTC CTCCACACAC
23581 CATCCTGAAG TCACCCCAAC TTCTACCACC ATCACCCCCA AACCCACCAG TACAGAAACC
23641 GGCACCCCTG TGGCCGACAC CACGTCAGCC ACCAGCAGCA GGCTACCCAC ACCCTTCACC
23701 ACACACTCCT TACCTACAGG GAGCAGTCCC TTCTCTTCCA CAGGTCCTAT GACTGCAACA
23761 TCCTTCCAGA CCACTACTAC CTATCCAACC CCATCACACC CTCACACCAC ACTTCCCACT
23821 CACATTCCAC CTTTCTCCAC CTCCTTGGTG ACTCCAAGTA CTCACACAGT CATCACCACT
23881 ACCCACGCAC AGATGTCCAC TTCTGCCTCC ATCCACTCAA CGCCAACAGG CACCATTCCT
23941 CCACCAACAA CGCTCAAGGC CACAGGGTCT ACCCACACAG CCCCACCAAT GACGCCGACC
24001 ACCAGTGGGA CCAGCCAAGC CCCGAGCTCA TTCAGCACAG CCAAAACCTC TACATCCTTA
24061 CATTCACACA CTTCCTCTGC ACACCATCCT GAAGTCACCC CAACTTCTAC CACCATCACC
24121 CCCAATCCCA CCAGTACAGA AACCGTCACC CCTGTGGCCC ACACCACCTC AGCCACCAGC
24181 AGCAGGCTAA CCACACCCTT CACCACACAC TCCCCACCTA CAGGGAGCAG TCCCATCTCT
24241 TCCACAGGTC CTGTCACTGC AACATCCTTC CATGCCACCA CTACCTATCC AACACCATCA
24301 CACCCTCAGA CCACACTTCC CACTCACGTT CCATCTTTCT CCACCTCCTT GGTGACTCCA
24361 AGTACTCACA CAGTCATCAC CCCTACCCAC GCACAGATGA CCACTTCTGC CTCCATCCAC
24421 TCAATGCCAA CAGGCACCAT TCCTCCACCG ACAACGCTGA AGGCCACAGG GTCCACCCAC
24481 ACAGCCCCAC CAATGATGCC AACCACCAGT GGGACCAGCC AAGCCTCAAG CTCATTCAAC
24541 ACAGCCAAAA CCTCTACATC CCTACATTCA CACACTTCCT CCACACACCA TCCTGAAGTC
24601 ACCCCAACTT CTATCACCAA CATCACCCTC AACCCCACCA GTATAGGAAC CTGGACACCC
24661 GTGGCCCACA CCACCTCAGC CACCAGCAGC AGGCTAACCA CACCCTTCAC CACACACTCC
24721 CCACCTACAG GGACCACTCC CATCTCTTCC ACAGGTCCTG TCACTGCAAC ATCCTTCCAT
24781 GCCACCACTA CCTATCCAAC ACCATCACAC CCTCAGACCA CACTTCCCAC TCACGTTCCA
24841 TCTTTCTCCA CCTCCTTGGT GACTCCAAGT ACTCACATAG TCATCACCCC TACCCACGCA
24901 CAGATGGCCA CTTCTGCCTC CATCCACTCA ATGCAAACAG GCACCATTCC TCCACCGACC
24961 ACGATCAAGG CCACAGGGTC CACCCACACA GCCCCACCAA TGACACCGAC CACCAGTGGG
25021 ACCAGCCAAT CCCTAAGCTC ATTTAGCACG GCCAAAACTT CTACATCCCT ACCTTACCAC
25081 ACTTCCTCCA CACACCATCC TGAAGTCACC CCAACTTCTA CCACCAACAT CACCCCCAAA
25141 CACACCAGTA CAGGCACCAG AACCCCTGTG GCCCACACCA CCTCGGCCAC CAGCAGCAGA
25201 CTACCCACAC CCTTCACCAC ACATTCCCCA CCTACAGGGA GCAGTCCCAT CTCTTCCACA
25261 GGTCCTATGA CTGCACCATC CTTTCAGACC ACCACTACCT ATCCAACCCC ATCACACCCT
25321 CAGACCACAC TTCCCACTCA CATTCCACCT TTCTCCACCT CCTTGGTGAC TCCAAGTACT
25381 CACAAGGTCA TCACCCCTAC CCATGCACAG ATGTCCACTT CTGCCTCCAT CCACTCAACG
25441 CCAACAGGCA CCATTCCTCC ACTAACAACG CTCAAGGTCA CAGGGTCCAC CCACACAGCC

25501 CCACCAATCA CAGTGACCAC CAGTGGGACC AGCCCATCCG CAAGCTCATT TAGCACAGGC
25561 AAAACCTCTA CATCCTTACA TTCACACACT TCCTCCACAC ACTATCCTGA AGTCACCCCA Exon 31
25621 ACTTCTACCA CCACCATCAC CCCCAACCAC ACCAGTACAG GCACCAGAAC CCCTGTGGCC
25681 CACACCACCT CGGCCACCAG CAGCAGGCTA CCCATACCCT TCACCACACA TTCCCCACCT
25741 ACAGGGAGCA GTCCCATCTC TTCCACAGGT CCTATGACTG CAACATCCTT TCAGACCACC
25801 ACTACCTATC CAACCCCATC ACACCCTCAG ACCACACTTC CCACTCACCT TCCACCTTTC
25861 TCCACCTCCT TGGTGACTCC AAGTACTCAC ACAGTCATCA TCACTACCCA CACACAGATG
25921 GCCACTTCTG CCTCCATCCA CTCAACGCCA ACAGGCACCG TTCCTCCACC AACAACGCTC
25981 AAGGCCACAG GGTCCACCCA CACAGCGCCA ACAATGACGC CGACCACCAG CGGGACGAGC
26041 CAAGCCCTGA GCTCATTCAA CACAGCCAAA ACCTCTACAT CCCTACATTC ACAAACTTCC
26101 TCCACACACC TTCCTGAAGT CACCCCAACT TCTACCGCCA TCACCCCCAA TCCCACCAGT
26161 ACAGGAACCG GCACCCCTGT GGCCCACACC ACCTCAGCCA CCAGCAGCAG GCTAACCACA
26221 CCCTTCACCA CACACTCCTC ACCTACAGGG AGCAGTCCCT TCTCTTCCAC AGGTCCTATG
26281 ACTGCAACAT CCTTCCAGAC CACCACTACC TATCCAACCC CATCACACCC TCAGACCACA
26341 CTTCCCACTC ACGTTCCACC TTTCTCCACC TCTTTGGTGA CTCCAAGTAC TCACACAGTC
26401 ATCACCCCTA CCCATGCACA GATGGCCACT TCTGCCTCCA TCCACTCAAT GCCAACAGGC
26461 ACGATTCCTC CACCGACAAC GCTCAAGGCC ACAGGGTCCA CCCACACAGC GCCAACAATG
26521 ACGCCGACCA CCAGCGGGAC CAGCCAAGCC CTGAGCTCAT TAAACACAGC CAAAACCTCT
26581 ACATCCCTAC ATTCACACAC TTCCTCCACA CACCATGCTG AAGCCACCTC AACTTCTACC
26641 ACCAACATCA CCCCCAACCC CACCAGTACA GGAACCCCAC CAATGACAGT GACCACCAGT
26701 GGGACCAGCC AATCCCGAAG CTCATTTAGC ACGGCCAAAA CCTCTACATC CCTACATTCA
26761 CACACTTCCT CCACACACCA TCCTGAAGTC ACCTCAACTT CTACCACCAG CATCACCCCC
26821 AACCACACCA GTACAGGCAC CAGAACCCCT GTGGCCCACA CCACGTCGGC CACCAGCAGC
26881 AGGCTACCCA CACCCTTCAC CACACACTCC CCACCTACAG GGACCACTCC CATCTCTTCC
26941 ACAGGTCCTG TCACTGCAAC ATCCTTCCAG ACCACCACTA CCGATCCAAC CCCATCACAC
27001 CCTCACACCA CACTTCCCAC TCACGTTCCA TCTTTCTCCA CCTCCTTGGT GACTCCAAGT
27061 ACTCACATAG TCATCACCCC TACCCACGCA CAGATGGCCA CTTCTGCCTC CATCCACTCA
27121 ATGCCAACAG GCACTATTCC TCCACCGACC ACGATCAAGG CCACAGGGTC CACCCACACA
27181 GCCCCACCAA TGACGGCAAC CACCAGTGGG ACCAGCCAAT CCCCAAGCTC ATTTAGCACG
27241 GCCAAAACTT CTACATCCCT ACATTCACAC ATTTCCTCAA CACATCATCC TGAAGTCACC
27301 CCAACTTCTA CCACCACCAT CACCCCCAAC CACACCAGTA CAGGCACCAG AACCCCTGTG
27361 GCCCACACCA CCTCGGCCAC CAGCAGCAGG CTACCCATAC CCTTCACCAC ACATTCCCCA
27421 CCTACAGGGA GCAGTCCCAT CTCTTCCACA GGTCCTATGA CTGCAACATC CTTTCAGACC
27481 ACCACTACCT ATCCAACCCC ATCACACCCT CAGACCACAC TTCCCACTCA CCTTCCACCT
27541 TTCTCCACCT CCTTGGTGAC TCCAAGTACT CACACAGTCA TCATCACTAC CCACACACAG
27601 ATGGCCACTT CTGCCTCCAT CCACTCAACG CCAACAGGCA CCGTTCCTCC ACCAACAACG
27661 CTCAAGGCCA CAGGGTCCAC CCACACAGCG CCAACAATGA CGCCGACCAC CAGCGGGACG
27721 AGCCAAGCCC TGAGCTCATT CAACACAGCC AAAACCTCTA CATCCCTACA TTCACAAACT
27781 TCCTCCACAC ACCTTCCTGA AGTCACCCCA ACTTCTACCA CCATCACCCC CAATCCCACC
27841 AGTCCAGGAA CCGGCACCCC TGTGGCCCAC ACCACCTCAG CCACCAGCAG CAGGCTAACC
27901 ACACCCTTCA CCACACACTC CTCACCTACA GGGAGCAGTC CCTTCTCTTC CACAGGTCCT
27961 ATGACTGCAA CATCCTTCAA GACCACCACT ACCTATCCAA CCCCATCACA CCCTCAGACC
28021 ACACTTCCCA CTCACGTTCC ACCTTTCTCC ACCTCTTTGG TGACTCCAAG TACTCACACA
28081 GTCATCACCC CTACCCATGC ACAGATGGCC ACTTCTGCCT CCATCCACTC AATGCCAACA
28141 GGCACGATTC CTCCACCGAC AACGCTCAAG GCCACAGGGT CCACCCACAC AGCGCCAACA
28201 ATGACGCTGA CCACCAGCGG GACCAGCCAA GCCCTGAGCT CATTAAACAC AGCCAAAACC
28261 TCTACATCCC TACATTCACA CACTTCCTCC ACACACCATG CTGAAGCCAC CTCAACTTCT
28321 ACCACCAACA TCACCCCCAA CCCCACCAGT ACAGGAACCC CACCAATGAC AGTGACCACC
28381 AGTGGGACCA GCCAATCCCG AAGCTCATTT AGCACGGCCA AAACCTCTAC ATCCCTACAT
28441 TCACACACTT CCTCCACACA CCATCCTGAA GTCACCTCAA CTTCTACCAC CAGCATCACC
28501 CCCAACCACA CCAGTACAGG CACCAGAACC CCTGTGGCCC ACACCACGTC GGCCACCAGC
28561 AGCAGGCTAC CCACACCCTT CACCACACAC TCCCCACCTA CAGGGACCAC TCCCATCTCT
28621 TCCACAGGTC CTGTCACTGC AACATCCTTC CAGACCACCA CTACCTATCC AACCCCATCA
28681 CACCCTCACA CCACACTTCC CACTCACGTT CCATCTTTCT CCACCTCCTT GGTGACTCCA
28741 AGTACTCACA CGGTCATCAT CCCTACCCAC ACACAGATGG CCACTTCTGC CTCCATCCAC
28801 TCAATGCCAA CAGGCACCAT TCCTCCACCG ACCACGATCA AGGCCACAGG GTCCACCCAC
28861 ACAGCCCCAC CAATGACACC GACCACCAGT GGGACCAGCC AATCCCCAAG CTCATTTAGC
28921 ACGGCCAAAA CTTCTACATC CCTACCTTAC CACACTTCCT CAACACACCA TCCTGAAGTC
28981 ACCCCAACTT CTACCACCAA CATCACCCCC AAACACACCA GTACAGGCAC CAGAACCCCT
29041 GTGGCCCACA CCACCTCGGC CAGCAGCAGC AGGCTACCCA CACCCTTCAC CACACACTCC
29101 CCACCTACAG GGAGCAGTCC CTTCTCTTCC ACAGGTCCTA TGACTGCAAC ATCCTTCCAG

```
29161 ACCACCACTA CCTATCCAAC CCCATCACAC CCTCAGACCA CACTTCCCAC TCACGTTCCA      Exon 31
29221 CCTTTCTCCA CCTCCTTGGT GACTCCAAGT ACTCACACAG TCATCATCAC TACCCACACA
29281 CAGATGGCCA CTTCTGCCTC CATCCACTCA ACGCCAACAG GCACCGTTCC TCCACCAACA
29341 ACGCTCAAGG CCACAGGGTC CACCCACACA GCCCCACCAA TGACAGTGAC CACCAGTGGG
29401 ACCAGCCAAA CCCACAGCTC ATTCAGCACA GCTACAGCCT CTTCTTCCTT CATATCCTCC
29461 TCGTCTTGGC TGCCTCAGAA CTCTAGCTCA AGGCCACCGT CATCACCTAT CACCACACAA
29521 CTCCCCCACT TGAGTTCTGC AACCACTCCT GTTTCCACAA CTAATCAGCT GTCCTCCTCA
29581 TTTTCTCCCA GTCCTTCTGC CCCCTCTACT GTTTCTTCTT ATGTGCCCTC CTCCCACTCC
29641 TCTCCCCAGA CTTCATCGCC TTCTGTTGGC ACATCTTCCT CTTTCGTGTC CGCCCCCGTG
29701 CACTCCACAA CCCTGAGCTC GGGGTCACAC TCCTCATTGT CCACTCATCC CACGACTGCA
29761 TCAGTGTCTG CATCTCCTCT TTTTCCTTCT TCTCCAGCTG CCTCTACTAC CATTAGGGCC
29821 ACTCTCCCCC ACACTATCTC CTCTCCTTTC ACCCTCTCTG CTCTACTCCC CATATCCACT
29881 GTTACCGTGT CTCCCACCCC ATCCAGCCAC CTAGCCTCCA GCACCATTGC ATTTCCGTCC
29941 ACGCCCAGGA CCACGGCCAG CACCCACACC GCCCCTGCCT TCTCCTCTCA GTCCACCACC
30001 TCGCGGTCCA CTTCTCTCAC CACCCGAGTT CCCACATCAG GCTTTGTGTC ACTCACCTCG
30061 GGGGTGACGG GTATCCCCAC CTCTCCAGTC ACCAACCTTA CCACCAGGCA CCCTGGTCCC
30121 ACCTTGTCGC CTACCACACG GTTCCTGACC AGCTCCCTCA CTGCCCATGG AAGCACCCCT
30181 GCTTCTGCCC CGGTATCTTC TCTCGGGACA CCTACGCCCA CCTCACCCGg taagtggcat
30241 ctctgtggcc cttctcctgg cctcacctct gtgctcatga ctcccaggca gcccctgcct
30301 cagcttaccc ctcatggtcc tgtgatccca ggaccacacg cctgttccct gccttcccta
30361 cacacctgcc aggcgtttcc actctcctct cctgggcacc ctctatgcca acgctgtcct
30421 ggccaagcaa gtgcctggcc tccccgttgg gccttgttcc ccagctaacc ttgacctttc
30481 cctgcactgg ctcactcatg cccggtcact gctgcactcc tggcctcccc tcagagtgtt
30541 acctgacaaa agccttccct ggctgcccct ttggttgctc agaggatgtc agggctggcc
30601 caaccccagg gttcaagcag cacagccctt cctccaagtg cagagagaga cacaaaagtc
30661 tggcacatcc cctgcctcgc cccaaaggac ctgttgaagg tggagctgga cctggcaacc
30721 agggcacctg ctccttcgtg ctcatgccaa ttctctgggc cacggctctt cccggcccat
30781 gcctgtgagc gagtccactg ctcccccggc catgggatgc ctggggacag agcacacctc
30841 aggccttatc catgtccacc tcacctcacc tcgcgttcca gggatgtccc tcccttccct
30901 cctgggtgtt cctctcacag caaccaccca gggactgggt ccctcgcatg catcgacctc
30961 caccaggcga caacgccaca gttgccttcg tggtctctca cgtgggtggc agctcgttgc
31021 tgcaagctga gggaatcttg gttcgggtcc ctccctgaga ccgggacttg ggtgcaaggt
31081 gtaaccaggg aggtgacccc aagaagcaga ggcgagggag caggaaccag ctgggagggg
31141 agggcagctg gggacggcag ggcctatgga agcacccaga gtcctgaccc tcccggagaa
31201 agccctctgc agcgggcagg taggctccag gtgacctgtc ttctctgcag ccaggaaacg
31261 aagctgggag cagccagggg agggcacacg ggcagcagag tccagccttt accccaggac
31321 tccttttcca cttttaaacc cacgctctcc agtggcggtt tctaatgcca acgctgccac
31381 tgcctgcatc ggtccaccct gctctgtgct gcctgccacg tacccaggcc tctcgctgag
31441 gctggctgtc tctgggcctt ccccgcgggc ccccaccccg ccccttccag gagcccagac
31501 cacggggcct gctccacttc cctcctcccc agctggggca cacggagctc ctgcaacacc
31561 atatgtcccg ctctggggtc actggcacgg tgtcctcggc ccccctgcag gtcctgggtc
31621 tggccaacca gagaggccca tgcccacagg tcagtgttga ggcctggggt tggtcatcag
31681 ggaagctggg gcccaagggt ggctcacaca ggcccagggc tcagtatctg ctgagtccgc
31741 tgagaaaatt ctctacctta tggactccag cagagcgact ttgaggggaa aaaacaaact
31801 aggatgcggc tggcctttgc ctttgtgtat ggctgaggtc tgagctctgt ggggctccac
31861 aaggggagac cacaccttgg gcaggagagt ccagagacag gtggggaccc cggtctctag
31921 ccagggtttc cctccctcct cgctccatgc ccaccctggg cgctgctgac cgtccactcg
31981 gctaccagGG GTCTGCAGTG TGCGGGAGCA GCAGGAGGAG ATCACGTTCA AGGGGTGCAT      Exon 32
32041 GGCGAACGTG ACGGTAACCC GCTGTGAGGG CGCCTGCATT TCCGCTGCCA Ggtgagtcca
32101 caggtgggag gcctgcccca cgtccaccaa ggtgctcaca accccacccc agagaggttc
32161 gggcacccca cccaggcgcc tgctgcccca tcaggtgagc cactctgcgc tccatctgcc
32221 caacaccgcc ctggcccctg tgagccggga gctcagcggg gaggtccagg cctctgcgga
32281 gcagctgccc tggggaggga ggggccttat gcccctgcag cagtcatgac cctgctctgt
32341 cttgacttct cggcagCTTC AACATCATCA CCCAGCAGGT GGATGCCCGC TGCAGCTGCT      Exon 33
32401 GCCGCCCCCT CCACTCCTAT GAGCAGCAGC TGGAGCTGCC CTGCCCCGAT CCCAGCACGC
32461 CTGGCCGGCG GCTCGTACTC ACCCTGCAGG TGTTCAGCCA CTGCGTGTGC AGCTCTGTGG
32521 CCTGTGGAGA CTAGCAGGGT CGCTGCCTGC TCTCCTGGGG CTGAAGGACT GCAGATGACA
32581 GACAGGAAAA CACCCACCAG CCCCCTTCCC GCTTGTGCCA GCAGCTGCTT TCCTGGTCAC
32641 CAGGCCTGGC CCCCAAGTGC CCTGGGCCGT GGCTCCCTGG GGCACCGGTT GGAGAGGGGC
32701 TGCCAAGCAG GGGCTCAGAC TACCACACTC CTGCAGACCC TGAGCCAGCA GAGAGGGACT
32761 GAGGCGGACA GTGGTCACGG ACCTCCCAGG CACACAGGGC ACTCCCGACC ACCCCTGCCC
```

32821 ACCGTCCAAC ACCTCCCAGC CCCTGAACTT GGCCCCAGCC CTGCTGGGCC CAGAACCCTG
32881 CAGATGAAGC CACAGAGCAG GCGCTCGACC AGACCCATCA GGGGCGAGGA GGGCACGGAA
32941 ACCTGTGCCG AGATGGGGGC AAGAGGCCCA GGCAGCCACC AGCACAGAGA AGAGGAGATC
33001 CCCAGAGTCA GGGAGGGCAG AGGGTGGCAG CGAGGGCAGG GCAGCCGCCC CCGCTCCCAG
33061 CCAGGCAGAA GGCCCCCACC AGCACCACAC CCATCCCCAG CAGCCTGTCC TTGGGAGAGG
33121 GCGTCACCCG GTCAGAGACT CCAAATAAAC CGGTTCTTGT CAAGGCA

Figure S7. Complete MUC2 mRNA sequence.

```
   1 CAACCCACAC CGCCCCTGCC AGCCACCATG GGGCTGCCAC TAGCCCGCCT GGCGGCTGTG
  61 TGCCTGGCCC TGTCTTTGGC AGGGGGCTCG GAGCTCCAGA CAGAGGGCAG AACCCGAAAC
 121 CACGGCCACA ACGTCTGCAG CACCTGGGGC AACTTCCACT ACAAGACCTT CGACGGGGAC
 181 GTCTTCCGCT TCCCCGGCCT CTGCGACTAC AACTTCGCCT CCGACTGCCG AGGCTCCTAC
 241 AAGGAATTTG CTGTGCACCT GAAGCGGGGT CCGGGCCAGG CTGAGGCCCC CGCCGGGGTG
 301 GAGTCCATCC TGCTGACCAT CAAGGATGAC ACCATCTACC TCACCCGCCA CCTGGCTGTG
 361 CTTAACGGGG CCGTGGTCAG CACCCCGCAC TACAGCCCCG GGCTGCTCAT TGAGAAGAGC
 421 GATGCCTACA CCAAAGTCTA CTCCCGCGCC GGCCTCACCC TCATGTGGAA CCGGGAGGAT
 481 GCACTCATGC TGGAGCTGGA CACTAAGTTC CGGAACCACA CCTGTGGCCT CTGCGGGGAC
 541 TACAACGGCC TGCAGAGCTA TTCAGAATTC CTCTCTGACG GCGTGCTCTT CAGTCCCCTG
 601 GAGTTTGGGA ACATGCAGAA GATCAACCAG CCCGATGTGG TGTGTGAGGA TCCCGAGGAG
 661 GAGGTGGCCC CCGCATCCTG CTCCGAGCAC CGCGCCGAGT GTGAGAGGCT GCTGACCGCC
 721 GAGGCCTTCG CGGACTGTCA GGACCTGGTG CCGCTGGAGC CGTATCTGCG CGCCTGCCAG
 781 CAGGACCGCT GCCGGTGCCC GGGCGGTGAC ACCTGCGTCT GCAGCACCGT GGCCGAGTTC
 841 TCCCGCCAGT GCTCCCACGC CGGCGGCCGG CCCGGGAACT GGAGGACCGC CACGCTCTGC
 901 CCCAAGACCT GCCCCGGGAA CCTGGTGTAC CTGGAGAGCG GCTCGCCCTG CATGGACACC
 961 TGCTCACACC TGGAGGTGAG CAGCCTGTGC GAGGAGCACC GCATGGACGG CTGTTTCTGC
1021 CCAGAAGGCA CCGTATATGA CGACATCGGG GACAGTGGCT GCGTTCCTGT GAGCCAGTGC
1081 CACTGCAGGC TGCACGGACA CCTGTACACA CCGGGCCAGG AGATCACCAA TGACTGCGAG
1141 CAGTGTGTCT GTAACGCTGG CCGCTGGGTG TGCAAAGACC TGCCCTGCCC CGGCACCTGT
1201 GCCCTGGAAG GCGGCTCCCA CATCACCACC TTCGATGGGA AGACGTACAC CTTCCACGGG
1261 GACTGCTACT ATGTCCTGGC CAAGGGTGAC CACAACGATT CCTACGCTCT CCTGGGCGAG
1321 CTGGCCCCCT GTGGCTCCAC AGACAAGCAG ACCTGCCTGA AGACGGTGGT GCTGCTGGCT
1381 GACAAGAAGA AGAATGTGGT GGTCTTCAAG TCCGATGGCA GTGTACTGCT CAACGAGCTG
1441 CAGGTGAACC TGCCCCACGT GACCGCGAGC TTCTCTGTCT TCCGCCCGTC TTCCTACCAC
1501 ATCATGGTGA GCATGGCCAT TGGCGTCCGG CTGCAGGTGC AGCTGGCCCC AGTCATGCAA
1561 CTCTTTGTGA CACTGGACCA GGCCTCCCAG GGGCAGGTGC AGGGCCTCTG CGGGAACTTC
1621 AACGGCCTGG AAGGTGACGA CTTCAAGACG GCCAGCGGGC TGGTGGAGGC CACGGGGGCC
1681 GGCTTTGCCA ACACCTGGAA GGCACAGTCA AGCTGCCATG ACAAGCTGGA CTGGTTGGAC
1741 GATCCCTGCT CCCTGAACAT CGAGAGCGCC AACTACGCCG AGCACTGGTG CTCCCTCCTG
1801 AAGAAGACAG AGACCCCCTT TGGCAGGTGC CACTCGGCTG TGGACCCTGC TGAGTATTAC
1861 AAGAGGTGCA AATATGACAC GTGTAACTGT CAGAACAATG AGGACTGCCT GTGCGCCGCC
1921 CTGTCCTCCT ACGCGCGCGC CTGCACCGCC AAGGGCGTCA TGCTGTGGGG CTGGCGGGAG
1981 CATGTCTGCA ACAAGGATGT GGGCTCCTGC CCCAACTCGC AGGTCTTCCT GTACAACCTG
2041 ACCACCTGCC AGCAGACCTG CCGCTCCCTC TCCGAGGCCG ACAGCCACTG TCTCGAGGGC
2101 TTTGCGCCTG TGGACGGCTG CGGCTGCCCT GACCACACCT TCCTGGACGA GAAGGGCCGC
2161 TGCGTACCCC TGGCCAAGTG CTCCTGTTAC CACCGCGGTC TCTACCTGGA GGCGGGGGAT
2221 GTGGTCGTCA GGCAGGAAGA ACGATGTGTG TGCCGGGATG GGCGGCTGCA CTGTAGGCAG
2281 ATCCGGCTGA TCGGCCAGAG CTGCACGGCC CCAAAGATCC ACATGGACTG CAGCAACCTG
2341 ACTGCACTGG CCACCTCGAA GCCCCGAGCC CTCAGCTGCC AGACGCTGGC CGCCGGCTAT
2401 TACCACACAG AGTGTGTCAG TGGCTGTGTG TGCCCCGACG GGCTGATGGA TGACGGCCGG
2461 GGTGGCTGCG TGGTGGAGAA GGAATGCCCT TGCGTCCATA ACAACGACCT GTATTCTTCC
2521 GGCGCCAAGA TCAAGGTGGA CTGCAATACC TGCACCTGCA AGAGAGGACG CTGGGTGTGC
2581 ACCCAGGCTG TGTGCCATGG CACCTGCTCC ATTTACGGGA GTGGCCACTA CATCACCTTT
2641 GATGGGAAGT ACTACGACTT TGACGGACAC TGCTCCTACG TGGCTGTTCA GGACTACTGC
2701 GGCCAGAACT CCTCACTGGG CTCATTCAGC ATCATCACCG AGAACGTCCC CTGTGGCACT
2761 ACGGGCGTCA CCTGCTCCAA GGCCATCAAG ATCTTCATGG GGAGGACGGA GCTGAAGTTG
2821 GAAGACAAGC ACCGTGTGGT GATCCAGCGT GATGAGGGTC ACCACGTGGC CTACACCACG
2881 CGGGAGGTGG GCCAGTACCT GGTGGTGGAG TCCAGCACGG GCATCATCGT CATCTGGGAC
2941 AAGAGGACCA CCGTGTTCAT CAAGCTGGCT CCCTCCTACA AGGGCACCGT GTGTGGCCTG
3001 TGTGGGAACT TTGACCACCG CTCCAACAAC GACTTCACCA CGCGGGACCA CATGGTGGTG
3061 AGCAGCGAGC TGGACTTCGG GAACAGCTGG AAGGAGGCCC CCACCTGCCC AGATGTGAGC
3121 ACCAACCCCG AGCCCTGCAG CCTGAACCCG CACCGCCGCT CCTGGGCCGA GAAGCAGTGC
3181 AGCATCCTCA AAAGCAGCGT GTTCAGCATC TGCCACAGCA AGGTGGACCC CAAGCCCTTC
3241 TACGAGGCCT GTGTGCACGA CTCGTGCTCC TGTGACACGG GTGGGGACTG TGAGTGCTTC
3301 TGCTCTGCCG TGGCCTCCTA CGCCCAGGAG TGTACCAAAG AGGGGGCCTG CGTGTTCTGG
3361 AGGACGCCGG ACCTGTGCCC CATATTCTGC GACTACTACA ACCCTCCGCA TGAGTGTGAG
3421 TGGCACTATG AGCCATGTGG GAACCGGAGC TTCGAGACCT GCAGGACCAT CAACGGCATC
3481 CACTCCAACA TCTCCGTGTC CTACCTGGAG GGCTGCTACC CCCGGTGCCC CAAGGACAGG
3541 CCCATCTATG AGGAGGATCT GAAGAAGTGT GTCACTGCAG ACAAGTGTGG CTGCTATGTC
3601 GAGGACACCC ACTACCCACC TGGAGCATCG GTTCCCACCG AGGAGACCTG CAAGTCCTGC
3661 GTGTGTACCA ACTCCTCCCA AGTCGTCTGC AGGCCGGAGG AAGGAAAGAT TCTTAACCAG
3721 ACCCAGGATG GCGCCTTCTG CTACTGGGAG ATCTGTGGCC CCAACGGGAC GGTGGAGAAG
3781 CACTTCAACA TCTGTTCCAT TACGACACGC CCGTCCACCC TGACCACCTT CACCACCATC
3841 ACCCTCCCCA CCACCCCCAC CACCTTCACC ACTACCACCA CCACCACCAC CCCGACCTCC
```

3901 AGCACAGTTT TATCAACAAC TCCGAAGCTG TGCTGCCTCT GGTCTGACTG GATCAATGAG
3961 GACCACCCCA GCAGTGGCAG CGACGACGGT GACCGAGAAA CATTTGATGG GGTCTGCGGG
4021 GCCCCTGAGG ACATCGAGTG CAGGTCGGTC AAGGATCCCC ACCTCAGCTT GGAGCAGCTA
4081 GGCCAGAAGG TGCAGTGTGA TGTCTCTGTT GGGTTCATTT GCAAGAATGA AGACCAGTTT
4141 GGAAATGGAC CATTTGGACT GTGTTACGAC TACAAGATAC GTGTCAATTG TTGCTGGCCC
4201 ATGGATAAGT GTATCACCAC TCCCAGCCCT CCAACTACCA CTCCCAGCCC TCCACCAACC
4261 AGCACGACCA CCCTTCCACC AACCACCACC CCCAGCCCTC CAACCACCAC CACAACCACC
4321 CCTCCACCAA CCACCACCCC CAGCCCTCCA ATAACCACCA CGACCACCCC TCCACCAACC
4381 ACCACTCCCA GCCCTCCAAT AAGCACCACA ACCACCCCTC CACCAACCAC CACTCCCAGC
4441 CCTCCAACCA CCACTCCCAG CCCTCCAACC ACCACTCCCA GCCCTCCAAC AACCACCACA
4501 ACCACCCCTC CACCAACCAC CACTCCCAGC CCTCCAACGA CTACGCCCAT CACTCCACCA
4561 GCCAGCACTA CCACCCTTCC ACCAACCACC ACTCCCAGCC CTCCAACAAC CACCACAACC
4621 ACCCCTCCAC CAACCACCAC TCCCAGTCCT CCAACGACTA CGCCCATCAC TCCACCAACC
4681 AGCACTACTA CCCTTCCACC AACCACCACT CCCAGCCCTC CACCAACCAC CACAACCACC
4741 CCTCCACCAA CCACCACTCC CAGCCCTCCA ACAACCACCA CTCCCAGTCC TCCAACAATC
4801 ACCACAACCA CCCCTCCACC AACCACCACT CCCAGCCCTC CAACAACGAC CACAACCACC
4861 CCTCCACCAA CCACCACTCC CAGCCCTCCA ACGACTACAC CCATCACTCC ACCAACCAGC
4921 ACTACCACCC TTCCACCAAC CACCACTCCC AGCCCTCCAC CAACCACCAC AACCACCCCT
4981 CCACCAACCA CCACTCCCAG CCCTCCAACA ACCACCACTC CCAGCCCTCC AATAACCACC
5041 ACAACCACCC CTCCACCAAC CACCACTCCC AGCTCTCCAA TAACCACCAC TCCCAGCCCT
5101 CCAACAACCA CCATGACCAC CCCTTCACCA ACCACCACCC CCAGCTCTCC AATAACCACC
5161 ACAACCACCC CTTCCTCAAC TACCACTCCC AGCCCTCCAC CAACCACCAT GACCACCCCT
5221 TCACCAACCA CCACTCCCAG CCCTCCAACA ACCACCACGA CCACCCTTCC ACCAACCACC
5281 ACTTCCAGCC CTCTAACAAC TACTCCTCTA CCTCCATCAA TAACTCCTCC TACATTTTCA
5341 CCATTCTCAA CGACAACCCC TACTACCCCA TGCGTGCCTC TCTGCAATTG GACTGGCTGG
5401 CTGGATTCTG GAAAACCCAA CTTTCACAAA CCAGGTGGAG ACACAGAATT GATTGGAGAC
5461 GTCTGTGGAC CAGGCTGGGC AGCTAACATC TCTTGCAGAG CCACCATGTA TCCTGATGTT
5521 CCCATTGGAC AGCTTGGACA AACAGTGGTG TGTGATGTCT CTGTGGGGCT GATATGCAAA
5581 AATGAAGACC AAAAGCCAGG TGGGGTCATC CCTATGGCCT TCTGCCTCAA CTACGAGATC
5641 AACGTTCAGT GCTGTGAGTG TGTCACCCAA CCCACCACCA TGACAACCAC CACCACAGAG
5701 AACCCAACTC CGACACCAAT CACCACCACC ACTACGGTGA CCCCAACCCC AACACCCACC
5761 AGCACACAGA GTACAACACC AACACCCATC ACCACCACCA ATACGGTAAC CCCAACCCCA
5821 ACCCCCACTG GCACACAGAC CCCAACCCCG ACACCCATCA CCACCACCAC CACTATGGTG
5881 ACCCCAACCC CAACAATCAC CAGCACACAG ACCCCAACCC CGACACCCAT CACCACCACT
5941 ACGGTGACCC CAACCCCAAC ACCCACCAGC ACACAGAGAA CAACACCGAC ATCCATCACC
6001 ACCACCACCA CGGTGACCCC AACCCCAACA CCCACCGGCA CACAGACCCC AACCACGACA
6061 CCCATCACCA CCACCACCAC GGTGACCCCA ACCCCAACAC CCACCGGCAC ACAGACCCCA
6121 ACAACGACAC CCATCAGCAC CACCACCACG GTGACCCCAA CCCCAACACC CACTGGAACA
6181 CAGACCCTAA CCCCAACACC CATCACCACC ACCACTACGG TGACCCCAAC CCCTACACCC
6241 ACCGGCACAC AGACCCCAAC ATCGACACCC ATCACCACCA CCACTACGGT GACCCCAACA
6301 CCAACACCCA CTGGCACACA GACCCCAACC CTGACACCCA TCACCACCAC CACTACGGTG
6361 ACCCCAACCC CAACACCCAC CGGCACACAG ACCCCAACCA CGACACCCAT CACCACCACC
6421 ACTACGGTGA CCCCAACCCC AACACCCACC GGCACAAAGA GTACAACCCC GACATCCATC
6481 ACCACCACCA CTATGGTGAC CCCAACCCCA CCACCCACTG GCACACAGAC CCCAACCACG
6541 ACACCCATCA CCACCACCAC TACGGTGACC CCAACCCCAA CACCCACCGG CACACAGACC
6601 CCAACCCCGA CACCCATCAC CACCACCACC ACGGTGACCC CAACCCCAAC ACCCACCGGC
6661 ACACAGACCC CAACATCGAC ACCCATCACC ACCAACACTA CGGTGACCCC AACCCCAACA
6721 CCAACCGGCA CACCGAGTAC AACCCTGACA CCCATCACCA CCACCACTAC GGTGACCCCA
6781 ACCCCAACAC CCACCGGCAC ACAGACCCCA ACATCGACAC CCATCAGCAC CACCACTATG
6841 GTGACCCCAA CCCCAACACC CACCGGCACA CAGACCCCAA CCCCTACACC CATCTCCACC
6901 ACCACTACGG TGACCCCAAC CCCAACACCC ACCGGCACAC AGACCCCAAC CCCGACACCC
6961 ATCACCACCA CCACCACGGT GACCCCAACC CCAACACCCA CCGGCACACA GACCCCAACA
7021 TCGACACCCA TCACCACCAC CACTACGGTG ACCCCAACCC CAACACCCAC CGGCACACAG
7081 ACCCCAACCA CGACACCCAT CACCACCAAC ACCACGGTGA CCCCAACCCC GACACCCACC
7141 GGCACACAGA CCCCAACCAC GGTACTCATC ACCACCACCA CTACGATGAC ACCAACCCCA
7201 ACACCCACCA GCACAAAGAG TACAACCGTG ACACCCATCA CCACCACCAC TACTGTGACC
7261 CCAACCCCAA CACCCACCGG CACACAGAGT ACAACCCTGA CACCCATCAC CACCACCACT
7321 ACGGTGACCC CAACCCCAAC ACCCACCGGC ATACAGACCC CAACAACGAC ACCCATCAGC
7381 ACCACCACCA CCGTGACCCC AACCCCAACA CCCACCGGCA CACAGACCCC AACATCGACA
7441 CCCATCACCA CCACCACTAC GGTGACCCCA ACCCCTACAC CCACTGGCAC ACAGACCCCA
7501 ACATCGACAC CCATCAGCAC CACCACTACG GTGACCCCAA CAGCAACACC CACCGGCACA
7561 CAGACCCCAA CCCTGACACC CATCACCACC ACCACTACGG TGACCCCAAC CCCAACACCC
7621 ACCGGCACAA AGAGTACAAC CCCGACATCC ATCACCACCA CCACTACGGT GACCCCAACC
7681 CCAACACCCA CTGGCACACA GACCCCAACC ACGACACCCA TCACCACCAC CACCACGGTG
7741 ACCCCAACCC CAACACCCAC CGGCACACAG ACCCCAACCC CGACACCCAT CACCACCACC
7801 ACCACGGTGA CCCCAACCCC AACACCCACC AGCACACAGA CCCCAACATC GACACCCATC
7861 ACCACCACCA CTACGGTGAC CCCAACCCCA ACACCCACTG GCACACAGAC CCCAACCACG

7921 ACACCCATTA CCACGACCAC CACGGTGACC CCAACCCCAA CACCCACCGG CACACAGGCC
7981 CCAACCCCAA CAGCCATCAC CACCACCACT ACGGGGACCC CAACCCCAAC ACCCACCGGC
8041 ACACAGACCC CAACCACGAC ACCCATCACC ACCACCACTA CGGTGACACC AACCCCAACA
8101 CCCACCGGCA CACAGTCCCC AACCCCAACA GCCATCACCA CCACCACTAC GGTGACCCCA
8161 ACCCCAACAC CCACCGGCAC ACAGACCCCA ACCACGACAC CCATCACCAC CACCACCACG
8221 GTGACCCCAA CCCCGACACC CACCGGCACA CAGAGTACAA CCCTGACACC CATCACCACC
8281 ACCACCACGG TGACACCAAC CCCAACACCC ACTGGCACAC AGACCCCAAC ATCGACACCC
8341 ATCACCACCA CCATTACGGT GACCCCAACC CCAACACCCA CCGGCACACA GACCCCAACC
8401 CCGACACCCA TCTCCACCAC CACTACGGTG ACCCCAACCC CAACACCCAC CGGCACACAG
8461 ACCCCAACAT CGACACCCAT CACCACCACC ACCACGGTGA CCCCAACCCC AACACCCACC
8521 GGCACACAGA CCCCAACAAC GACACCCATC AGCACCACCA CCACGGTGAC CCCAACCCCA
8581 ACACCCACCG GCACACAGAC CCCAACATCG ACACCCATCA CCACCACCAC CACGGTGACC
8641 CCAACCCCAA CACCCACCGG CACACAGACC CCAACCACGA CACCCATCAG CACCACCACC
8701 ACGGTGACCC CAACCCCAAC ACCCACCGGC ACACAGACCC CAACATCGAC ACCCATCACC
8761 ACCACCACCA CGGTGACCCC AACCCCAACA CCCACCGGCA CACAGACCCC AACCCCGACA
8821 CCCATCACCA CCACCACCAC GGTGACCCCA ACCCCAACAC CCACCGGCAC ACAGACCCCA
8881 ACATCGACAC CCATCACCAC CACCACCACG GTGACCCCAA CCCCAACACC CACCGGCACA
8941 CAGACCCCAA CCCCGACACC CATCACCACC ACCACCACGG TGACCCCAAC CCCAACACCC
9001 ACCGGCACAC AGACCCCAAC CCCGACACCC ATCACCACCA CCACCACGGT GACCCCAACC
9061 CCAACACCCA CCGGCACACA GACCCCAACA TCGACACCCA TCACCACCAC CACTACGGTG
9121 ACCCCAACCC CAACACCCAC CGGCACACAG ACCCCAACCA CGACACCCAT CACCACCACC
9181 ACCACGGTGA CCCCAACCCC AACACCCACT GGCACACAGA GTACAACCCT GACACCCATC
9241 ACCACCACCA CCACGGTGAC ACCAACCCCA ACACCCACCG GCACACAGAC CCCAACATCG
9301 ACACCCATCA CCACCATCAC TACGGTGACC CCAACCCCAA CACCCACCGG CACACAGACC
9361 CCAACCCCGA CACCCATCTC CACCACCACT ACAGTGACCC CAACCCCAAC ACCCACCGGC
9421 ACACAGACCC CAACCATGAC ACCCATCACC ACCACCACCA CGGTGACCCC AACCCCAACA
9481 CCCACCGGCA CACAGACCCC AACAACGACA CCCATCAGCA CCACCACCAC GGTGACCCCA
9541 ACCCCAACAC CCACCGGCAC ACAGACCCCA ACATCGACAC CCATCACCAC CACCACTACG
9601 GTGACCCCAA CCCCAACACC CACCGGCACA CAGACCCCAA CCACGACACC CATCACCACC
9661 ACCACCACGG TGACCCCAAC CCCAACACCC ACCGGCACAC AGAGTACAAC CCTGACACCC
9721 ATCACCACCA CCACCACGGT GACACCAACC CCAACACCCA CCGGCACACA GACCCCAACC
9781 CCGACACCCA TCTCCACCAC CACTACGGTG ACCCCAACCC CAACACCCAC CGGCACACAG
9841 ACCCCAACCA TGACACCCAT CACCACCACC ACCACGGTGA CCCCAACCCC AACACCCACC
9901 GGCACACAGA CCCCAACAAC GACACCCATC AGCACCACCA CCACGGTGAC CCCAACCCCA
9961 ACACCCACCG GCACACAGAC CCCAAGATCG ACACCCATCA CCACCACCAC TAAGGTGACC
10021 CCAACCCCAA CACCCACCGG CACACAGACC CCAACCCCGA CACCCATCAC CACCACCACC
10081 ACGGTGACCC CAACCCCAAC ACCCACTGGC ACACAGGCCC CAACCCCAGC AGCCATCACC
10141 ACCACCAGTA CGGTGACCCC AACCCCAACA CCCACCGGCA CACAGACCCC AACCACGACA
10201 CCCATCACCA CCACCACCAC GGTGACCCCA ACCCCAACAC CCACCGGCAC ACAGAGTACA
10261 ACCCTGACAC CCATCACCAC CACCACCACG GTGACACCAA CCCCAACACC CACCGGCACA
10321 CAGACCCCAA CATCGACACC CATCACCACC ACCACTACGG TGACCCCAAC CCCAACACCC
10381 ACCGGCACAC AGACCCCAAC CCCGACACCC ATCTCCACCA CCAGTACGGT GACCCCAACC
10441 CCAACACCCA CCGGCACACA GACCCCAACC ATGACACCCA TCACCACCAC CACCACGGTG
10501 ACCCCAACCC CAACACCCAC CGGCACACAG ACCCCAACAA CGACACCCAT CAGCACCACC
10561 ACCACGGTGA CCCCAACCCC AACACCCACC GGCACACAGA ACCCAACATC GACACCCATC
10621 ACCACCACCA CTACGGTGAC CCCAACCCCA ACACCCACCG GCACACAGAC CCCAACCATG
10681 ACACCCATCA CCACCACCAC CACGGTGACC CCAACCCCAA CACCCACTGG CACACAGGCC
10741 CCAACCCCAA CAGCCATCAC CACCACCACT ACGGTGACCC CAACCCCAAC ACCCACCGGC
10801 ACACAGACCC CAACCACGAC ACCCATCACC ACCACCACCA CGGTGACCCC AACCCCAATA
10861 CCCACCGGCA CACAGAGTAC AACCCTGACA CCCATCACCA CCACCACCAC GGTGACACCA
10921 ACCCCAACAC CCACCGGCAC ACAGACCCCA ACCCCGATAC CCATCTCCAC CACCACTACG
10981 GTGACCCCAA CCCCAACACC CACCGGCACA CAGACCCCAA CCATGACACC CATCACCACC
11041 ACCACCACGG TGACCCCAAC CCCAACACCC ACCGGCACAC AGACCCCAAC AACGACACCC
11101 ATCAGCACCA CCACCACGGT GACCCCAACC CCAACACCCA CCGGCACACA GACCCCAACA
11161 TCGACACCCA TCACCACCAC CACTACGGTG ACCCCAACCC CAATACCCAC CGGCACACAG
11221 ACCCCAACCA CGACACCCAT CACCACCACC ACCACGGTGA CCCCAACCCC AACACCCACT
11281 GGCACACAGG CCCCAACCCC AACAGCCATC ACCACCACCA CTACGGTGAC CCCAACCCCA
11341 ACACCCACCG GCACACAGAC CCCAACCACG ACACCCATCA CCACCACCAC CACGGTGACC
11401 CCAACCCCAA TACCCACCGG CACACAGAGT ACAACCCTGA CACCCATCAC CACCACCACC
11461 ACGGTGACAC CAACCCCAAC ACCCACCAGC ACACAGACCC CAACCCCGAC ACCCATCTCC
11521 ACCACCACTA CGGTGACCCC AACCCCAACA CCCACCGGCA CACAGACCCC AACCATGACA
11581 CCCATCACCA CCACCACCAC GGTGACCCCA ACCCCAACAC CCACCGGCAC ACAGACCCCA
11641 ACAACGACAC CCATCAGCAC CACCACCACG GTGACCCCAA CCCCAACACC CACCGGCACA
11701 CAGACCCCAA CATCGACACC CATCACCACC ACCACTACAG TGACCCCAAC CCCAACATCC
11761 ACCGGCACAC AGACCCCAAC CACGACACCC ATCACCACCA CCACCACGGT GACCCCAACC
11821 CCAACACCCA CTGGCACACA GGCCCCAACC CCAACAGCCA TCACCACCAC CAGTACGGTG
11881 ACCCCAACCC CAACACCCAC CGGCACACAG ACCCCAACCA CGACACCCAT CACCACCACC

```
11941 ACTACGGTGA CACCAACCCC AACACCCACC GGCACACAGT CCCCAACCCC AACAGCCATC
12001 ACCACCACCA CTACGGTGAC CCCAACCCCA ACACCCACCG GCACACAGAC CCCAACATCG
12061 ACACCCATCA CCACCACCAC TACGGTGACC CCAACCCCAA CACCCACCGG CACACAGACC
12121 CCAACCCCGA CACCCATCTC CACCACCACT ACGGTGACCC CAACCCCAAC ACCCACCGGC
12181 ACACAGACCC CAACCACGAC ACCCATCACC ACCACCACCA CGGTGACCCC AACCCCGACA
12241 CCCACCGGCA CACAGACCCC AACCACGGTA CTCATCACCA CCACCACTAC GATGACCCCA
12301 ACCCCAACAC CCACCAGCAC AAAGAGTACA ACCGTGACAC CCATCACCAC CACAACTACG
12361 GTGACCGCAA CCCCAACACC CACCGGCACA CAGACCCCAA CCATGATACC CATCAGCACC
12421 ACCACTACGG TGACCCCAAC CCCAACACCC ACCACTGGAA GCACGGGGCC CCCCACCCAC
12481 ACAAGCACAG CACCCATTGC TGAGTTGACC ACATCCAATC CTCCGCCTGA GTCCTCAACC
12541 CCTCAGACCT CTCGGTCCAC CTCTTCCCCT CTCACGGAGT CAACCACCCT TCTGAGTACC
12601 CTACCACCTG CCATTGAGAT GACCAGCACG GCCCCACCCT CCACACCCAC GGCACCCACG
12661 ACCACGAGCG GAGGCCACAC ACTGTCTCCA CCGCCCAGCA CCACCACGTC CCCTCCAGGC
12721 ACCCCCACTC GCGGTACCAC GACCGGGTCA TCTTCAGCCC CCACCCCCAG CACTGTGCAG
12781 ACGACCACCA CCAGTGCCTG GACCCCAACG CCGACCCCAC TCTCCACACC CAGCATCATC
12841 AGGACCACAG GCCTGAGGCC CTACCCTTCC TCTGTGCTTA TCTGCTGTGT CCTGAACGAC
12901 ACCTACTACG CACCAGGTGA GGAGGTGTAC AACGGCACAT ACGGAGACAC CTGTTATTTC
12961 GTCAACTGCT CACTGAGCTG TACGTTGGAG TTCTATAACT GGTCCTGCCC ATCCACGCCC
13021 TCCCCAACAC CCACGCCCTC CAAGTCGACG CCCACGCCTT CCAAGCCATC GTCCACGCCC
13081 TCCAAGCCGA CGCCCGGCAC CAAGCCCCCC GAGTGCCCAG ACTTTGATCC TCCCAGACAG
13141 GAGAACGAGA CTTGGTGGCT GTGCGACTGC TTCATGGCCA CGTGCAAGTA CAACAACACG
13201 GTGGAGATCG TGAAGGTGGA GTGTGAGCCG CCGCCCATGC CCACCTGCTC CAACGGCCTC
13261 CAACCCGTGC GCGTCGAGGA CCCCGACGGC TGCTGCTGGC ACTGGGAGTG CGACTGCTAC
13321 TGCACGGGCT GGGGCGACCC GCACTATGTC ACCTTCGACG GACTCTACTA CAGCTACCAG
13381 GGCAACTGCA CCTACGTGCT GGTGGAGGAG ATCAGCCCCT CCGTGGACAA CTTCGGAGTT
13441 TACATCGACA ACTACCACTG CGATCCCAAC GACAAGGTGT CCTGTCCCCG CACCCTCATC
13501 GTGCGCCACG AGACCCAGGA GGTGCTGATC AAGACCGTGC ATATGATGCC CATGCAGGTG
13561 CAGGTGCAGG TGAACAGGCA GGCGGTGGCA CTGCCCTACA AGAAGTACGG GCTGGAGGTG
13621 TACCAGTCTG GCATCAACTA CGTGGTGGAC ATCCCCGAGC TGGGTGTCCT CGTCTCCTAC
13681 AATGGCCTGT CCTTCTCCGT CAGGCTGCCC TACCACCGGT TTGGCAACAA CACCAAGGGC
13741 CAGTGTGGCA CCTGCACCAA CACCACCTCC GACGACTGCA TTCTGCCCAG CGGGGAGATC
13801 GTCTCCAACT GTGAGGCTGC GGCTGACCAG TGGCTGGTGA ACGACCCCTC CAAGCCACAC
13861 TGCCCCCACA GCAGCTCCAC GACCAAGCGC CCGGCCGTCA CTGTGCCCGG GGGCGGTAAA
13921 ACGACCCCAC ACAAGGACTG CACCCCATCT CCCCTCTGCC AGCTCATCAA GGACAGCCTG
13981 TTTGCCCAGT GCCACGCACT GGTGCCCCCG CAGCACTACT ACGATGCCTG CGTGTTCGAC
14041 AGCTGCTTCA TGCCGGGCTC GAGCCTGGAG TGCGCCAGTC TGCAGGCCTA CGCAGCCCTC
14101 TGTGCCCAGC AGAACATCTG CCTCGACTGG CGGAACCACA CGCATGGGGC CTGCTTGGTG
14161 GAGTGCCCAT CTCACAGGGA GTACCAGGCC TGTGGCCCTG CAGAAGAGCC CACGTGCAAA
14221 TCCAGCTCCT CCCAGCAGAA CAACACAGTC CTGGTGGAAG GCTGCTTCTG TCCTGAGGGC
14281 ACCATGAACT ACGCTCCTGG CTTTGATGTC TGCGTGAAGA CCTGCGGCTG TGTGGGACCT
14341 GACAATGTGC CCAGAGAGTT TGGGGAGCAC TTCGAGTTCG ACTGCAAGAA CTGTGTCTGC
14401 CTGGAGGGTG GAAGTGGCAT CATCTGCCAA CCCAAGAGGT GCAGCCAGAA GCCCGTTACC
14461 CACTGCGTGG AAGACGGCAC CTACCTCGCC ACGGAGGTCA ACCCTGCCGA CACCTGCTGC
14521 AACATTACCG TCTGCAAGTG CAACACCAGC CTGTGCAAAG AGAAGCCCTC CGTGTGCCCG
14581 CTGGGATTCG AAGTGAAGAG CAAGATGGTG CCTGGAAGGT GCTGTCCTTT CTACTGGTGT
14641 GAGTCCAAGG GGGTGTGTGT TCACGGGAAT GCTGAGTACC AGCCCGGTTC TCCAGTTTAT
14701 TCCTCCAAGT GCCAGGACTG CGTGTGCACG GACAAGGTGG ACAACAACAC CCTGCTCAAC
14761 GTCATCGCCT GCACCCACGT GCCCTGCAAC ACCTCCTGCA GCCCTGGCTT CGAACTCATG
14821 GAGGCCCCCG GGGAGTGCTG TAAGAAGTGT GAACAGACGC ACTGTATCAT CAAACGGCCC
14881 GACAACCAGC ACGTCATCCT GAAGCCCGGG GACTTCAAGA GCGACCCGAA GAACAACTGC
14941 ACATTCTTCA GCTGCGTGAA GATCCACAAC CAGCTCATCT CGTCCGTCTC CAACATCACC
15001 TGCCCCAACT TTGATGCCAG CATTTGCATC CCGGGCTCCA TCACATTCAT GCCCAATGGA
15061 TGCTGCAAGA CCTGCACCCC TCGCAATGAG ACCAGGGTGC CCTGCTCCAC CGTCCCCGTC
15121 ACCACGGAGG TTTCGTACGC CGGCTGCACC AAGACCGTCC TCATGAATCA TTGCTCCGGG
15181 TCCTGCGGGA CATTTGTCAT GTACTCGGCC AAGGCCCAGG CCCTGGACCA CAGCTGCTCC
15241 TGCTGCAAAG AGGAGAAAAC CAGCCAGCGT GAGGTGGTCC TGAGCTGCCC CAATGGCGGC
15301 TCGCTGACAC ACACCTACAC CCACATCGAG AGCTGCCAGT GCCAGGACAC CGTCTGCGGG
15361 CTCCCCACCG GCACCTCCCG CCGGGCCCGG CGCTCCCCTA GGCATCTGGG GAGCGGGTGA
15421 GCGGGGTGGG CACAGCCCCC TTCACTGCCC TCGACAGCTT TACCTCCCCC GGACCCTCTG
15481 AGCCTCCTAA GCTCGGCTTC CTCTCTTCAG ATATTTATTG TCTGAGTCTT TGTTCAGTCC
15541 TTGCTTTCCA ATAATAAACT CAGGGGGACA TGC
```

Figure S8. Complete MUC6 mRNA sequence (reverse complement).

```
   1 ATGGTCCAGC GGTGGCTGCT GCTGTCCTGC TGCGGAGCCC TGCTCAGCGC TGGTCTGGCT
  61 AACACCTCCT ACACCAGCCC AGGCCTCCAG AGGCTGAAGG ACTCTCCACA GACAGCCCCG
 121 GACAAAGGCC AGTGCTCCAC GTGGGGGGCT GGTCACTTCT CCACCTTCGA CCACCACGTG
 181 TACGACTTCT CGGGGACGTG CAACTACATC TTCGCGGCCA CCTGCAAGGA CGCCTTCCCC
 241 ACCTTCAGTG TCCAGCTGCG GCGAGGCCCA GACGGGAGCA TCTCGCGGAT CATCGTGGAG
 301 CTGGGGGCCT CCGTCGTCAC TGTGAGCGAA GCCATCATCT CAGTCAAGGA CATCGGGGTC
 361 ATCAGCCTGC CCTATACCAG CAATGGACTC CAGATCACAC CCTTCGGCCA GAGCGTGCGG
 421 CTGGTGGCCA AGCAGCTGGA GCTGGAGCTG GAAGTCGTGT GGGGTCCTGA CAGCCACCTC
 481 ATGGTTCTGG TGGAGCGGAA GTACATGGGT CAGATGTGCG GGCTCTGCGG GAACTTTGAC
 541 GGGAAGGTGA CCAACGAGTT TGTCAGTGAG GAGGGCAAGT TCCTGGAACC CCACAAGTTT
 601 GCTGCCCTCC AGAAGCTGGA CGACCCCGGC GAGATCTGCA CCTTCCAGGA CATCCCCAGC
 661 ACCCACGTCC GGCAGGCCCA GCACGCCCGG ATCTGCACCC AGCTGCTGAC CCTGGTGGCC
 721 CCTGAGTGCA GCGTGTCCAA GGAGCCCTTC GTGCTAAGCT GCCAGGCGGA CGTGGCCGCA
 781 GCCCCCCAGC CAGGCCCACA GAACAGCAGT TGTGCCACCC TGTCGGAGTA CTCCCGCCAG
 841 TGCAGCATGG TGGGCCAGCC GGTCCGCCGC TGGCGGAGCC CCGGCCTGTG CTCCGTGGGT
 901 CAGTGCCCGG CCAACCAGGT GTACCAGGAG TGCGGCTCGG CCTGCGTGAA GACCTGCTCC
 961 AACCCGCAGC ACAGCTGCTC CAGCTCCTGC ACCTTCGGGT GCTTCTGCCC GGAAGGTACG
1021 GTCCTGAATG ACCTCTCCAA TAACCACACC TGCGTGCCCG TCACCCAGTG CCCCTGTGTG
1081 CTCCACGGCG CCATGTATGC CCCCGGGGAG GTCACAATAG CTGCCTGCCA AACCTGCCGG
1141 TGCACCCTGG GCCGCTGGGT GTGCACGGAG CGGCCGTGCC CCGGACACTG CTCCCTGGAA
1201 GGTGGCTCCT TTGTTACCAC ATTTGACGCC AGGCCCTACC GCTTCCACGG CACCTGCACC
1261 TACATCCTCC TCCAGAGCCC CCAGCTTCCC GAGGACGGTG CCCTCATGGC TGTGTACGAC
1321 AAGTCCGGCG TCTCACACTC CGAGACCTCC CTGGTGGCTG TGGTCTACCT CTCCAGGCAG
1381 GACAAAATTG TGATCTCTCA GGACGAGGTG GTCACCAACA ACGGAGAAGC CAAGTGGCTG
1441 CCATACAAGA CTCGCAACAT CACGGTCTTC AGGCAGACGT CCACCCACCT CCAGATGGCC
1501 ACCAGCTTCG GGCTGGAGCT CGTGGTCCAG CTGCGCCCCA TCTTCCAGGC CTATGTCACT
1561 GTTGGGCCCC AGTTCAGAGG TCAGACCAGA GGGCTCTGCG GCAACTTCAA CGGGGACACA
1621 ACGGATGACT TCACCACTAG CATGGGTATC GCCGAGGGCA CCGCCTCGCT GTTTGTGGAC
1681 TCCTGGCGGG CGGGGAACTG TCCGGCCGCT CTGGAGCGTG AGACTGACCC CTGCTCCATG
1741 AGCCAGCTCA ACAAGGTGTG TGCAGAGACC CACTGCTCCA TGCTGCTGAG GACAGGCACG
1801 GTGTTCGAGA GGTGCCACGC CACAGTGAAC CCTGCACCCT TCTACAAGAG GTGCGTGTAC
1861 CAGGCCTGCA ACTACGAGGA GACCTTTCCC CACATCTGTG CCGCCCTGGG CGACTACGTA
1921 CACGCCTGCT CCTTGCGGGG CGTCCTGCTC TGGGGCTGGA GAAGCAGTGT GGACAACTGC
1981 ACCATCCCCT GCACGGGTAA CACCACCTTC AGCTACAACA GCCAAGCCTG TGAGCGCACC
2041 TGCCTGTCGC TGTCGGACCG TGCCACCGAG TGCCACCACA GCGCCGTGCC CGTGGACGGT
2101 TGCAACTGCC CCGATGGCAC CTACCTGAAC CAAAAGGGCG AGTGTGTGCG CAAGGCCCAG
2161 TGCCCGTGCA TACTGGAGGG TTACAAGTTC ATCCTGGCCG AGCAGTCCAC TGTCATCAAC
2221 GGCATCACCT GCCACTGCAT CAACGGGCGG CTGAGTTGCC CGCAGCGGCC ACAGATGTTC
2281 CTGGCCTCCT GCCAGGCCCC TAAGACCTTC AAGTCCTGCA GCCAGTCCTC CGAGAACAAG
2341 TTTGGGGCAG CCTGTGCCCC CACATGCCAG ATGCTGGCCA CCGGTGTTGC CTGCGTGCCC
2401 ACCAAGTGTG AGCCTGGCTG TGTCTGCGCC GAGGGCCTCT ACGAGAATGC CGACGGGCAG
2461 TGTGTGCCCC CCGAGGAGTG CCCATGTGAG TTCTCGGGGG TCTCCTACCC TGGAGGAGCT
2521 GAGCTCCACA CTGACTGCAG GACCTGCTCC TGCTCAAGGG GGAGGTGGGC CTGTCAGCAG
2581 GGCACCCACT GCCCATCCAC CTGCACCCTC TACGGGGAGG GCCACGTCAT CACCTTCGAC
2641 GGCCAGCGCT TCGTATTCGA CGGCAACTGC GAGTACATCC TGGCCACGGA CGTCTGTGGT
2701 GTCAACGACT CACAGCCCAC CTTCAAGATC CTGACAGAGA ACGTCATCTG TGGGAACTCC
2761 GGGGTCACAT GCTCACGGGC CATCAAGATC TTCCTGGGGG GCCTGTCCGT GGTGCTGGCG
2821 GACAGAAACT ACACGGTCAC CGGGGAGGAG CCCCACGTGC AGCTCGGGGT GACGCCGGGT
2881 GCGCTGAGCC TTGTCGTGGA CATCAGCATC CCCGGGAGGT ACAACCTGAC GCTCATCTGG
2941 AACAGGCACA TGACCATCCT CATCAGGATC GCCCGTGCCT CCCAGGATCC CCTCTGCGGC
3001 TTGTGTGGCA ACTTCAACGG GAACATGAAG GACGACTTCG AGACGCGCAG CAGGTACGTG
3061 GCATCCAGCG AGCTGGAGTT GGTGAACTCG TGGAAGGAGA GCCCGCTGTG CGGGGACGTG
3121 AGCTTCGTGA CAGACCCCTG CAGTCTCAAT GCCTTCCGGC GCTCCTGGGC CGAGCGCAAG
3181 TGCAGCGTCA TCAACAGCCA GACCTTTGCC ACCTGCCACA GCAAGGTATA CCACCTGCCC
3241 TACTACGAGG CCTGCGTGCG CGACGCATGT GGGTGTGACA GTGGCGGGGA CTGTGAGTGT
3301 CTGTGCGATG CCGTGGCTGC CTACGCCCAA GCCTGTCTGG ACAAGGGTGT GTGCGTGGAC
3361 TGGAGGACCC CGGCCTTCTG CCCCATCTAC TGCGGCTTCT ACAACACGCA CACGCAGGAC
3421 GGCCATGGCG AGTACCAGTA CACACAGGAG GCCAACTGCA CGTGGCACTA CCAGCCCTGC
3481 CTCTGCCCCA GCCAGCCACA GAGCGTCCCA GGCAGCAACA TCGAAGGCTG CTACAACTGC
```

```
3541 TCCCAGGATG AGTACTTCGA CCACGAGGAG GGGGTGTGCG TGCCCTGCAT GCCGCCCACC
3601 ACGCCGCAGC CACCCACCAC GCCGCAGCTG CCCACCACAG GCTCACGGCC CACGCAAGTC
3661 TGGCCCATGA CGGGAACCTC CACCACCATC GGGCTTCTCA GCTCCACCGG ACCCTCACCC
3721 AGCTCTAATC ACACCCCTGC CAGCCCCACC CAGACACCCC TCCTTCCAGC CACGCTCACA
3781 TCCTCCAAGC CCACAGCCTC CTCGGGAGAA CCACCTAGAC CAACCACGGC CGTCACCCCA
3841 CAAGCCACAT CAGGGCTGCC TCCCACAGCC ACACTGAGAT CGACAGCCAC AAAACCCACA
3901 GTGACCCAGG CCACAACCAG GGCCACGGCG TCGACCGCCA GCCCAGCCAC GACGTCCACA
3961 GCTCAGTCCA CAACACGGAC CACAATGACA CTACCAACCC CAGCCACATC AGGGACAAGC
4021 CCCACGCTGC CAAAATCGAC CAATCAGGAA CTGCCAGGAA CAACGGCCAC CCAGACGACA
4081 GGCCCACGTC CAACCCCAGC AAGCACCACA GGCCCAACCA CCCCACAGCC AGGACAACCC
4141 ACGAGGCCCA CAGCCACAGA GACCACTCAA ACAAGAACGA CTACTGAATA CACAACGCCC
4201 CAAACCCCAC ACACCACACA CTCCCCGCCT ACGGCGGGGA GTCCCGTCCC TTCCACAGGT
4261 CCTGTCACTG CAACATCTTT CCATGCCACC ACTACCTATC CAACCCCATC ACACCCTGAG
4321 ACCACACTTC CCACTCACGT TCCACCTTTC TCCACCTCCT TGGTGACTCC AAGTACTCAC
4381 ACAGTCATCA CCCCTACCCA CGCACAGATG GCCACATCTG CCTCCAACCA CTCAGCGCCA
4441 ACAGGTACCA TTCCTCCACC AACAACGCTC AAGGCCACAG GGTCCACCCA CACAGCCCCA
4501 CCAATAACGC CGACCACCAG TGGGACCAGC CAAGCCCACA GCTCATTCAG CACAAACAAA
4561 ACACCTACCT CGCTACATTC ACACACTTCC TCCACACACC ATCCTGAAGT CACCCCAACT
4621 TCTACTACCA CGATTACTCC CAACCCCACT AGTACACGCA CCAGAACCCC TGTGGCCCAC
4681 ACCAACTCAG CCACCAGCAG CAGGCCACCA CCACCCTTCA CCACACACTC CCCACCTACA
4741 GGGAGCAGTC CCTTCTCTTC CACAGGTCCC ATGACGGCAA CATCCTTCAA GACCACCACT
4801 ACCTATCCAA CCCCATCACT CCCTCAGACC ACTCTTCTCA CTCACGTTCC ACCTTTCTCA
4861 ACCTCTTTGG TGACTCCAAT TACTCACACA GTCATCACCC CTACCCACCC ACAGATGTCC
4921 ACTTCTGCCT ATATCCACTC AACGCCAACA GGCACGATTG CTCCACCAAC AACAGTTAAG
4981 GCCACAAGGT CCACCTACAC AGCCCCACTA ATGACGGCAA CCACCAGTAG GATCAGCCAA
5041 GCCCACAGCT CAATCAGCAC AGCCAAAACC TCTACATCCC TCCACTCACA TGCTTCCTCC
5101 ACACACCATC CTGAAGTCAC CCCAACTTCT ACCACCAACG TGACTCCCAA GTCCACCAGT
5161 AGAGGCACCA GCACCCCTGT GACCCACACC ACCTCGGCCA CCAGTAGCAG GCCACCCACA
5221 CCCATCACAA CACACTCTTC ACCTACCAGG AGCAGTCCCC TCTCTTCCAC AGGTCGTATG
5281 ACTGCAACAT CTCTCAAGAC CACCACTACC TATCCAACCC CATCACAAGC TCACATCACA
5341 CTTCCCATTC ATGTTCCACC TTTCTCCACC TCATCGGTGA CTCCAAGTAC TCACACAGTC
5401 ATCACCCCAA CCCACCCACA GATGTCCACT TCTGCCTCCA ACCACTCAAC GTCAACAGGC
5461 ACCATTCCTC CACTGACAAC GCTCATGGCC ACAGGGTCCA CACACACAGC CCCACTAATA
5521 ACAGTGACCA CCAGTAGGAC CAGCCAAGTC CACAGCTCCT TCAGCACAGC CAAAACCTCT
5581 ACATCCCTCC TCTCCCATGC TTCCTCCACA CACCATCCAG AAATCACCAC AAATTCTACC
5641 ACCACCATTA CTCCCAACCC CACTAGTACA GGCACCGGAA CCCCTGTGGC CCACACCACC
5701 TCAGCCACCA GCAGCAGGCC ACCACCACCC TTCACCACAC ACTCCCCACC TACAGAGAGC
5761 AGTCCCCTCT CTCCCACAGG TCCTATGACT CCAACATCCT TCAAGACCAC CACTACCTAT
5821 CCAACCACAT CACACCCTCA GACCACACTT CCCACTCACG TTCCACCTTT CTCCAGCTCG
5881 TCAGTGACTC CAAGTACTCA CACAGTCATC ACCCCTACCC ATGCACAGAT GTCCACTTCT
5941 GCCTCGATCC ACTCAACGCC AACAGGTACC ATTCCTCCAC TGACAACGCT CACGGCCACA
6001 GGGTCCACAC ACACAGCCTC ACCAATGACG GGGACAACCA TTCGGACCAC CCAAGCCCAC
6061 AGCTCATTCA GCATAGCCAA AACCTCTACA TCCATCCTCT CACATGCTTC CTCCACACAC
6121 CATCCGGAAA CCACACCAAC TTCTACCACC AACATTACTC CCAAGTCCAC TAGTGCAGGA
6181 ACCAGCACCC CTGTGGCCCA CACCACCTTG GCCACCAGCA GCAGGCCACC CACACCCTTC
6241 ACCACACACT CCCCACCTAC AGGGAGCAGT CCCATCTCTT CCACAGGTCC TATGACTGCA
6301 ACATCCATCA AGACCACCAC GACCTATCCA ACCCCATCAC ACCCTCAGAC CACACTTACC
6361 ACTCATGTTC CACCTTTCTC CACCTCATCA GTCACTCCAA GTACTCACAC AGTCATCACC
6421 CCTACCCACG CACAAATGTC CACTTCTGCT TCGATCCACT CAACGCCAAC AGGCACCGTT
6481 CCTCCACTGA CAACGCGCAT GCCCACAGGG TCGACACACA CAGGCCCACC AATGACGGGG
6541 ACCATTATTC AGACAAGCAA AGCTCACAAC TCATTCAGCA CAGCCAAAAC TTCTACATCT
6601 CTCCACTCAC ATGCTTCCTC CACACACCAT CCTGAAACCA CACCAACTTC TACCACCAAC
6661 ATTACTCCCA AGTCCACTAG TGCAGGAACC AGCACCCCTG TGGCCCACAC CACCTTGGCC
6721 ACCAGCAGCA GGCTACCCAC AACCTTCACC ACACACTTCC CACCTACAGG GAGCAGTCAT
6781 GTCTCTTCCA CAGGTCCTAT GACTGCAACA TCCTCCCAGA CCACCACTAC CCATCCAACC
6841 CCATCACACC CTCAGACCAC ACTTCCCACT CACATTCCAC CTTTCTCCAC CTCCTTGGTG
6901 ACTCCAAGTA CTCACACAGT CATCACCCCT ACCCACGCAC AAGTGTCCAC TTCTGCCTCC
6961 ATCCACTCAA CACCAACAGG CACCATTCCT CCACCTACAA CGGTAAAGGC CACAGGCACA
7021 GGGTCCACGC ACACAGCACC ACGAATGACA GTGACCACCA GCGGGACCAG CCAAGCGCAC
7081 AGCTCTTTCA GCACAGCCAA AACCTCCACA TCCCTACACT CACATGCTTC TTCAACACTC
7141 CATCCTGAAG TCACCCCAAC TTCTACCACC ACCATCACCC CCAACCCCAC CAATACAGGC
```

7201 ATCAGAACGC CTGTGGCAAA CACCACCTCA GCCACCAGCA GCAGGCTAAC CACACCCTTC
7261 ACCACACACT CCCCACCTAC AGGGAGCAGT CCCATCTCTT CCACAGGTCC TATGACTGCA
7321 ACATCCTTCC AGACCACCAC TACATATCCA ACCCCATCAC ACCCTCAGAC CACACTTCCC
7381 ACTCACGTTC CACCTTTCTC CACCTCCTTG GTGACTCCAA GTACTCACAC AGTCATCACC
7441 CCTACCCACG CACAGATGGC CACTTCCGCC TCCATCCATT CAACGCCAAC AGGCACCATT
7501 CCTCCACTGA CAACGCTCAT GAACACAGGG TCCACACACA CAGCCCCACC AGTGACGCCG
7561 ACCACCAGTG GGACGAGCCA AGCCGCGAGC TCATTCAGCA CAGCCAAAAC CTCTACATCC
7621 TTACATTCAC ACACTTCCTC CACACACCAT CCTGAAGTCA CCCCAACTGC TACCACCAAA
7681 ATCACCACCA ACCCCACCAG TATAGGAAGC AGCACACCCA TGGCCCACAC TACCTCAGCC
7741 ACCAGCAGCA GGCTAACTAC ACCATTCACC ACACACTCCC CATCTACAGG GAGCAGTCCT
7801 GTCTCTTCCA CAGGTCCTAT GACTGCAACA TCCTTCCAGA CCACCACTAC CTATCCAACA
7861 CCATCACTCT CTCAGACCAC TCTTCCCACT TACGTTCCAC CTTTCTCCAC CTCCTTGGTG
7921 ACTCCAAGTA CTCACACAGT CATCACCCCT CCCCGCACAC AGATGGCCAC TTCTGCCTCC
7981 ATCCACTCAA CGCCAACAGG CACCATTCCT CCACCGACAA CGCTCAAGGC CACAGGGTCC
8041 ACCCACACAG CGCCAACAAG GACGCTGACC ACCAGCGGGA CCAGCCAAGC CCTGAGCTCA
8101 TTAAACACAG CCAAAACCTC TACATCCCTA CATTCACACA CTTCCTCCAC ACACCATCCT
8161 GAAGCCACCT CAACTTCTAC CACCAACATC ACCCCCAACC CCACCAGTAC AGGAACTGGG
8221 ACACCTGTGG CCCACACCAC CTCAGCCACC AGCAGCAGGC TAACCACACC CTTCACCACA
8281 CACTCCCCAC CTACAGGGAG CACTCCCATC TCTTCCACAG GTCCTGTCAC TGCAACATCC
8341 TTCCATGCCA CCACTACCTA TCCAACACCA TCACACCCTC AGACCACACA TCCCACTCAC
8401 GTTACATCTT TCTCCACCTC CTTGGTGACT CCAAGTACTC ACACAGTCAT CACCCCTACC
8461 CACGCACAGA TGGCCACTTC TGCGTCCATC CACTCAACGC CAACAGGCAC CATTTCTCCA
8521 CCAACAACGC TCAAGGCCAC AGGGTCCATC CACACAGCCC CACCAATGAC GCCGACCACC
8581 AGTGGGACCA GCCAATCCCC AAGCTCATTT AGCATGGCCA AAACTTCTAC ATCCCTACCT
8641 TACCACACTT CCTCCACACA CCATCCTGAA GTCACCCCAA CTTCTACCAC CAACATCACC
8701 CCCAAACACA CCAGTACAGG CACAAGAACC CCTGTGGCCC ACACCACCTC GGCCACCAGC
8761 AGCAAGCTAC CCACACCCTT CACCACACAC TCCCCACCTA CAGGAAGCAG TCCCATCTCT
8821 TCCACAGGTC CTGTCACTGC AACATCCTTC CAGACCACCA CTACCTATCC AACCCCATCA
8881 CACTCTCACA CCACACTTCC CACTCACGTT CCACCTTCCT CCACCTCCTT GGTGACTCCA
8941 AATACTCACA CAGTCATCAC CCATACCCAT GCACAGATGT CCACTTCTGC CTCCATCCAC
9001 TCAACGCCAA CAGGCACCAT TCCTCCACCG ACAACGCTCA AGGCCACAGG GTCCACCCAC
9061 ACAGCCCCAC CAATGACGCC GACCACCAGT GGGACCAGGC AAGCCCCAAG CTCATTCAGC
9121 ACAGCCAAAA CTTCTACATC CCTACATTCA CACACTTCCT CCACACACCA TCCTGCAGTC
9181 ACCCCAACTT CTACCACCAA CATCACCCCC AACCACACCA GTACAGGCAC CAGGACCCCT
9241 GTGGCCCACA CCACTTCGGC CACCAGCAGC AGGCTACCCA CACCCTTCAC CACACACTCC
9301 CCACCGACAG GGAGCAGTCC CATCTCTTCC ACAGCTCCTG TCACTGCAAC ATCCTTCCAG
9361 ACCACCACTA CCTATCCAAC CACATCACAG TCTCAGACCA CACTTCCCAC TCACATTCCA
9421 CCTTTCTCCA CCTCCTTGGT GACTCCAAGT ACACACACAG TCATCACCCC AACCCATCAA
9481 CAGATGGCCA CTTCTGCCTC CATCCACTCA ACGCCAACAG GCACCATTCC TCCACTGACA
9541 ACGCTCAAGG CCACAGGGTC CACCCACACA GCCCCACCAA TGACGCCGAC CACCAGTGGG
9601 ACCAGCCAAG CCCTGAGCTC ATTCAGCACA GCCAAAACCT CTACATCCCT ACATTCACAC
9661 ACTTCCTCCA CACACCATCC TGAAGTCACC CCAACTTCTA CCACCATCAC CCCCAAACCC
9721 ACCAGTACAG AAACCGGCAC CCCTGTGGCC GACACCACGT CAGCCACCAG CAGCAGGCTA
9781 CCCACACCCT TCACCACACA CTCCTTACCT ACAGGGAGCA GTCCCTTCTC TTCCACAGGT
9841 CCTATGACTG CAACATCCTT CCAGACCACT ACTACCTATC CAACCCCATC ACACCCTCAC
9901 ACCACACTTC CCACTCACAT TCCACCTTTC TCCACCTCCT TGGTGACTCC AAGTACTCAC
9961 ACAGTCATCA CCACTACCCA CGCACAGATG TCCACTTCTG CCTCCATCCA CTCAACGCCA
10021 ACAGGCACCA TTCCTCCACC AACAACGCTC AAGGCCACAG GGTCTACCCA CACAGCCCCA
10081 CCAATGACGC CGACCACCAG TGGGACCAGC CAAGCCCCGA GCTCATTCAG CACAGCCAAA
10141 ACCTCTACAT CCTTACATTC ACACACTTCC TCTGCACACC ATCCTGAAGT CACCCCAACT
10201 TCTACCACCA TCACCCCCAA TCCCACCAGT ACAGAAACCG TCACCCCTGT GGCCCACACC
10261 ACCTCAGCCA CCAGCAGCAG GCTAACCACA CCCTTCACCA CACACTCCCC ACCTACAGGG
10321 AGCAGTCCCA TCTCTTCCAC AGGTCCTGTC ACTGCAACAT CCTTCCATGC CACCACTACC
10381 TATCCAACAC CATCACACCC TCAGACCACA CTTCCCACTC ACGTTCCATC TTTCTCCACC
10441 TCCTTGGTGA CTCCAAGTAC TCACACAGTC ATCACCCCTA CCCACGCACA GATGACCACT
10501 TCTGCCTCCA TCCACTCAAT GCCAACAGGC ACCATTCCTC CACCGACAAC GCTGAAGGCC
10561 ACAGGGTCCA CCCACACAGC CCCACCAATG ATGCCAACCA CCAGTGGGAC CAGCCAAGCC
10621 TCAAGCTCAT TCAACACAGC CAAAACCTCT ACATCCCTAC ATTCACACAC TTCCTCCACA
10681 CACCATCCTG AAGTCACCCC AACTTCTATC ACCAACATCA CCCTCAACCC CACCAGTATA
10741 GGAACCTGGA CACCCGTGGC CCACACCACC TCAGCCACCA GCAGCAGGCT AACCACACCC
10801 TTCACCACAC ACTCCCCACC TACAGGGACC ACTCCCATCT CTTCCACAGG TCCTGTCACT

10861 GCAACATCCT TCCATGCCAC CACTACCTAT CCAACACCAT CACACCCTCA GACCACACTT
10921 CCCACTCACG TTCCATCTTT CTCCACCTCC TTGGTGACTC CAAGTACTCA CATAGTCATC
10981 ACCCCTACCC ACGCACAGAT GGCCACTTCT GCCTCCATCC ACTCAATGCA AACAGGCACC
11041 ATTCCTCCAC CGACCACGAT CAAGGCCACA GGGTCCACCC ACACAGCCCC ACCAATGACA
11101 CCGACCACCA GTGGGACCAG CCAATCCCTA AGCTCATTTA GCACGGCCAA AACTTCTACA
11161 TCCCTACCTT ACCACACTTC CTCCACACAC CATCCTGAAG TCACCCCAAC TTCTACCACC
11221 AACATCACCC CCAAACACAC CAGTACAGGC ACCAGAACCC CTGTGGCCCA CACCACCTCG
11281 GCCACCAGCA GCAGACTACC CACACCCTTC ACCACACATT CCCCACCTAC AGGGAGCAGT
11341 CCCATCTCTT CCACAGGTCC TATGACTGCA CCATCCTTTC AGACCACCAC TACCTATCCA
11401 ACCCCATCAC ACCCTCAGAC CACACTTCCC ACTCACATTC CACCTTTCTC CACCTCCTTG
11461 GTGACTCCAA GTACTCACAA GGTCATCACC CCTACCCATG CACAGATGTC CACTTCTGCC
11521 TCCATCCACT CAACGCCAAC AGGCACCATT CCTCCACTAA CAACGCTCAA GGTCACAGGG
11581 TCCACCCACA CAGCCCCACC AATCACAGTG ACCACCAGTG GGACCAGCCC ATCCGCAAGC
11641 TCATTTAGCA CAGGCAAAAC CTCTACATCC TTACATTCAC ACACTTCCTC CACACACTAT
11701 CCTGAAGTCA CCCCAACTTC TACCACCACC ATCACCCCCA ACCACACCAG TACAGGCACC
11761 AGAACCCCTG TGGCCCACAC CACCTCGGCC ACCAGCAGCA GGCTACCCAT ACCCTTCACC
11821 ACACATTCCC CACCTACAGG GAGCAGTCCC ATCTCTTCCA CAGGTCCTAT GACTGCAACA
11881 TCCTTTCAGA CCACCACTAC CTATCCAACC CCATCACACC CTCAGACCAC ACTTCCCACT
11941 CACCTTCCAC CTTTCTCCAC CTCCTTGGTG ACTCCAAGTA CTCACACAGT CATCATCACT
12001 ACCCACACAC AGATGGCCAC TTCTGCCTCC ATCCACTCAA CGCCAACAGG CACCGTTCCT
12061 CCACCAACAA CGCTCAAGGC CACAGGGTCC ACCCACACAG CGCCAACAAT GACGCCGACC
12121 ACCAGCGGGA CGAGCCAAGC CCTGAGCTCA TTCAACACAG CCAAAACCTC TACATCCCTA
12181 CATTCACAAA CTTCCTCCAC ACACCTTCCT GAAGTCACCC CAACTTCTAC CGCCATCACC
12241 CCCAATCCCA CCAGTACAGG AACCGGCACC CCTGTGGCCC ACACCACCTC AGCCACCAGC
12301 AGCAGGCTAA CCACACCCTT CACCACACAC TCCTCACCTA CAGGGAGCAG TCCCTTCTCT
12361 TCCACAGGTC CTATGACTGC AACATCCTTC CAGACCACCA CTACCTATCC AACCCCATCA
12421 CACCCTCAGA CCACACTTCC CACTCACGTT CCACCTTTCT CCACCTCTTT GGTGACTCCA
12481 AGTACTCACA CAGTCATCAC CCCTACCCAT GCACAGATGG CCACTTCTGC CTCCATCCAC
12541 TCAATGCCAA CAGGCACGAT TCCTCCACCG ACAACGCTCA AGGCCACAGG GTCCACCCAC
12601 ACAGCGCCAA CAATGACGCC GACCACCAGC GGGACCAGCC AAGCCCTGAG CTCATTAAAC
12661 ACAGCCAAAA CCTCTACATC CCTACATTCA CACACTTCCT CCACACACCA TGCTGAAGCC
12721 ACCTCAACTT CTACCACCAA CATCACCCCC AACCCCACCA GTACAGGAAC CCCACCAATG
12781 ACAGTGACCA CCAGTGGGAC CAGCCAATCC CGAAGCTCAT TTAGCACGGC CAAAACCTCT
12841 ACATCCCTAC ATTCACACAC TTCCTCCACA CACCATCCTG AAGTCACCTC AACTTCTACC
12901 ACCAGCATCA CCCCCAACCA CACCAGTACA GGCACCAGAA CCCCTGTGGC CCACACCACG
12961 TCGGCCACCA GCAGCAGGCT ACCCACACCC TTCACCACAC ACTCCCCACC TACAGGGACC
13021 ACTCCCATCT CTTCCACAGG TCCTGTCACT GCAACATCCT TCCAGACCAC CACTACCGAT
13081 CCAACCCCAT CACACCCTCA CACCACACTT CCCACTCACG TTCCATCTTT CTCCACCTCC
13141 TTGGTGACTC CAAGTACTCA CATAGTCATC ACCCCTACCC ACGCACAGAT GGCCACTTCT
13201 GCCTCCATCC ACTCAATGCC AACAGGCACT ATTCCTCCAC CGACCACGAT CAAGGCCACA
13261 GGGTCCACCC ACACAGCCCC ACCAATGACG GCAACCACCA GTGGGACCAG CCAATCCCCA
13321 AGCTCATTTA GCACGGCCAA AACTTCTACA TCCCTACATT CACACATTTC CTCAACACAT
13381 CATCCTGAAG TCACCCCAAC TTCTACCACC ACCATCACCC CCAACCACAC CAGTACAGGC
13441 ACCAGAACCC CTGTGGCCCA CACCACCTCG GCCACCAGCA GCAGGCTACC CATACCCTTC
13501 ACCACACATT CCCCACCTAC AGGGAGCAGT CCCATCTCTT CCACAGGTCC TATGACTGCA
13561 ACATCCTTTC AGACCACCAC TACCTATCCA ACCCCATCAC ACCCTCAGAC CACACTTCCC
13621 ACTCACCTTC CACCTTTCTC CACCTCCTTG GTGACTCCAA GTACTCACAC AGTCATCATC
13681 ACTACCCACA CACAGATGGC CACTTCTGCC TCCATCCACT CAACGCCAAC AGGCACCGTT
13741 CCTCCACCAA CAACGCTCAA GGCCACAGGG TCCACCCACA CAGCGCCAAC AATGACGCCG
13801 ACCACCAGCG GGACGAGCCA AGCCCTGAGC TCATTCAACA CAGCCAAAAC CTCTACATCC
13861 CTACATTCAC AAACTTCCTC CACACACCTT CCTGAAGTCA CCCCAACTTC TACCACCATC
13921 ACCCCCAATC CCACCAGTCC AGGAACCGGC ACCCCTGTGG CCCACACCAC CTCAGCCACC
13981 AGCAGCAGGC TAACCACACC CTTCACCACA CACTCCTCAC CTACAGGGAG CAGTCCCTTC
14041 TCTTCCACAG GTCCTATGAC TGCAACATCC TTCAAGACCA CCACTACCTA TCCAACCCCA
14101 TCACACCCTC AGACCACACT TCCCACTCAC GTTCCACCTT TCTCCACCTC TTTGGTGACT
14161 CCAAGTACTC ACACAGTCAT CACCCCTACC CATGCACAGA TGGCCACTTC TGCCTCCATC
14221 CACTCAATGC CAACAGGCAC GATTCCTCCA CCGACAACGC TCAAGGCCAC AGGGTCCACC
14281 CACACAGCGC CAACAATGAC GCTGACCACC AGCGGGACCA GCCAAGCCCT GAGCTCATTA
14341 AACACAGCCA AAACCTCTAC ATCCCTACAT TCACACACTT CCTCCACACA CCATGCTGAA
14401 GCCACCTCAA CTTCTACCAC CAACATCACC CCCAACCCCA CCAGTACAGG AACCCCACCA
14461 ATGACAGTGA CCACCAGTGG GACCAGCCAA TCCCGAAGCT CATTTAGCAC GGCCAAAACC

14521 TCTACATCCC TACATTCACA CACTTCCTCC ACACACCATC CTGAAGTCAC CTCAACTTCT
14581 ACCACCAGCA TCACCCCCAA CCACACCAGT ACAGGCACCA GAACCCCTGT GGCCCACACC
14641 ACGTCGGCCA CCAGCAGCAG GCTACCCACA CCCTTCACCA CACACTCCCC ACCTACAGGG
14701 ACCACTCCCA TCTCTTCCAC AGGTCCTGTC ACTGCAACAT CCTTCCAGAC CACCACTACC
14761 TATCCAACCC CATCACACCC TCACACCACA CTTCCCACTC ACGTTCCATC TTTCTCCACC
14821 TCCTTGGTGA CTCCAAGTAC TCACACGGTC ATCATCCCTA CCCACACACA GATGGCCACT
14881 TCTGCCTCCA TCCACTCAAT GCCAACAGGC ACCATTCCTC CACCGACCAC GATCAAGGCC
14941 ACAGGGTCCA CCCACACAGC CCCACCAATG ACACCGACCA CCAGTGGGAC CAGCCAATCC
15001 CCAAGCTCAT TTAGCACGGC CAAAACTTCT ACATCCCTAC CTTACCACAC TTCCTCAACA
15061 CACCATCCTG AAGTCACCCC AACTTCTACC ACCAACATCA CCCCCAAACA CACCAGTACA
15121 GGCACCAGAA CCCCTGTGGC CCACACCACC TCGGCCAGCA GCAGCAGGCT ACCCACACCC
15181 TTCACCACAC ACTCCCCACC TACAGGGAGC AGTCCCTTCT CTTCCACAGG TCCTATGACT
15241 GCAACATCCT TCCAGACCAC CACTACCTAT CCAACCCCAT CACACCCTCA GACCACACTT
15301 CCCACTCACG TTCCACCTTT CTCCACCTCC TTGGTGACTC CAAGTACTCA CACAGTCATC
15361 ATCACTACCC ACACACAGAT GGCCACTTCT GCCTCCATCC ACTCAACGCC AACAGGCACC
15421 GTTCCTCCAC CAACAACGCT CAAGGCCACA GGGTCCACCC ACACAGCCCC ACCAATGACA
15481 GTGACCACCA GTGGGACCAG CCAAACCCAC AGCTCATTCA GCACAGCTAC AGCCTCTTCT
15541 TCCTTCATAT CCTCCTCGTC TTGGCTGCCT CAGAACTCTA GCTCAAGGCC ACCGTCATCA
15601 CCTATCACCA CACAACTCCC CCACTTGAGT TCTGCAACCA CTCCTGTTTC CACAACTAAT
15661 CAGCTGTCCT CCTCATTTTC TCCCAGTCCT TCTGCCCCCT CTACTGTTTC TTCTTATGTG
15721 CCCTCCTCCC ACTCCTCTCC CCAGACTTCA TCGCCTTCTG TTGGCACATC TTCCTCTTTC
15781 GTGTCCGCCC CCGTGCACTC CACAACCCTG AGCTCGGGGT CACACTCCTC ATTGTCCACT
15841 CATCCCACGA CTGCATCAGT GTCTGCATCT CCTCTTTTTC CTTCTTCTCC AGCTGCCTCT
15901 ACTACCATTA GGGCCACTCT CCCCCACACT ATCTCCTCTC CTTTCACCCT CTCTGCTCTA
15961 CTCCCCATAT CCACTGTTAC CGTGTCTCCC ACCCCATCCA GCCACCTAGC CTCCAGCACC
16021 ATTGCATTTC CGTCCACGCC CAGGACCACG GCCAGCACCC ACACCGCCCC TGCCTTCTCC
16081 TCTCAGTCCA CCACCTCGCG GTCCACTTCT CTCACCACCC GAGTTCCCAC ATCAGGCTTT
16141 GTGTCACTCA CCTCGGGGGT GACGGGTATC CCCACCTCTC CAGTCACCAA CCTTACCACC
16201 AGGCACCCTG GTCCCACCTT GTCGCCTACC ACACGGTTCC TGACCAGCTC CCTCACTGCC
16261 CATGGAAGCA CCCCTGCTTC TGCCCCGGTA TCTTCTCTCG GGACACCTAC GCCCACCTCA
16321 CCCGGGGTCT GCAGTGTGCG GGAGCAGCAG GAGGAGATCA CGTTCAAGGG GTGCATGGCG
16381 AACGTGACGG TAACCCGCTG TGAGGGCGCC TGCATTTCCG CTGCCAGCTT CAACATCATC
16441 ACCCAGCAGG TGGATGCCCG CTGCAGCTGC TGCCGCCCCC TCCACTCCTA TGAGCAGCAG
16501 CTGGAGCTGC CCTGCCCCGA TCCCAGCACG CCTGGCCGGC GGCTCGTACT CACCCTGCAG
16561 GTGTTCAGCC ACTGCGTGTG CAGCTCTGTG GCCTGTGGAG AC

Figure S9.

MUC2 PTS-TR2 units showing common and unique repeats.

Common TR

	n	% (n=98)
PTSTPITTTTTTVTPTPTPTGTQT	13	13.3
PTTTPISTTTTVTPTPTPTGTQT	8	8.2
TTLTPITTTTTTVTPTPTPTGTQT	6	6.1
PTPTPISTTTTVTPTPTPTGTQT	6	6.1
PTTTPITTTTTTVTPTPTPTGTQS	6	6.1
PTPTPITTTTTTVTPTPTPTGTQT	5	5.1
PTMTPITTTTTTVTPTPTPTGTQT	5	5.1
PTPTAITTTTTTVTPTPTPTGTQT	4	4.1
PTTTPITTTTTTVTPTPTPTGTQT	4	4.1
PTTTPITTTTTTVTPTPTPTGTQA	3	3.1
PTTVLITTTTTMTPTPTPTSTKS	2	2.0
PTTTPITTTTTTVTPTPIPTGTQS	2	2.0
TTPTSITTTTTTVTPTPTPTGTQT	2	2.0
Total No.	66	

Unique TR

	TR No.	
	1	PTPTPITTTTTTVTPTPTPTSTQS
	2	TTPTPITTTNTVTPTPTPTGTQT
	3	PTPTPITTTTTMVTPTPTITSTQT
	4	PTPTPITTTT*VTPTPTPTSTQR
	8	LTPTPITTTTTTVTPTPTPTGTQT
	10	PTLTPITTTTTTVTPTPTPTGTQT
	11	PTTTPITTTTTTVTPTPTPTGTKS
	12	TTPTSITTTTMVTPTPPPTGTQT
	15	PTSTPITTNTTVTPTPTPTGTPS
	17	PTSTPISTTTMVTPTPTPTGTQT
	21	PTTTPITTNTTVTPTPTPTGTQT
	23	TTVTPITTTTTTVTPTPTPTGTQS
	24	TTLTPITTTTTTVTPTPTPTGIQT
	27	PTSTPISTTTTVTPTATPTGTQT
	28	PTLTPITTTTTTVTPTPTPTGTKS
	31	PTPTPITTTTTTVTPTPTPTSTQT
	34	PTPTAITTTTTGTPTPTPTGTQT
	39	PTSTPITTTITVTPTPTPTGTQT
	53	PTSTPITTITTVTPTPTPTGTQT
	63	PRSTPITTTTKVTPTPTPTGTQT
	64	PTPTPITTTTTTVTPTPTPTGTQA
	65	PTPAAITTTSTVTPTPTPTGTQT
	69	PTPTPISTTSTVTPTPTPTGTQT
	71	PTTTPISTTTTVTPTPTPTGTQN
	73	PTMTPITTTTTTVTPTPTPTGTQA
	77	PTPIPISTTTTVTPTPTPTGTQT
	80	PTSTPITTTTTTVTPTPIPTGTQT
	84	TTLTPITTTTTTVTPTPTPTSTQT
	88	PTSTPITTTTTTVTPTPTSTGTQT
	90	PTPTAITTTSTVTPTPTPTGTQT
	97	TTVTPITTTTTTVTATPTPTGTQT
	98	PTMIPISTTTTVTPTPTPTTGST
Total No.	32	

Figure S10

MUC6 Tandem repeats.

a

```
SPVPSTGPVTATSFHATTTYPTPSHPETTLPTHVPPFSTSLVTPSTHTVITPTHAQMATSASNHSAPTGTIPPPTTLKA--TGSTHTAPPITPTTSGTSQAHSSFSTNKTPTSLHSHTSSTHHPEVTPTSTTTITPNPTSTRTRTPVAHTNSATSSRPPPPFTTHSPPTGS   169
SPFSSTGPMTATSFKTTTTYPTPSLPQTTLLTHVPPFSTSLVTPITHTVITPTHPQMSTSAYIHSTPTGTIAPPTTVKA--TRSTYTAPLMTATTSRISQAHSSISTAKTSTSLHSHASSTHHPEVTPTSTTNVTPKSTSRGTSTPVTHTTSATSSRPPTPITTHSSPTRS   169
SPLSSTGRMTATSLKTTTTYPTPSQAHITLPIHVPPFSTSSVTPSTHTVITPTHPQMSTSASNHSTSTGTIPPLTTLMA--TGSTHTAPLITVTTSRTSQVHSSFSTAKTSTSLLSHASSTHHPEITTNSTTTITPNPTSTGTGTPVAHTTSATSSRPPPPFTTHSPPTES   169
SPLSPTGPMTPTSFKTTTTYPTTSHPQTTLPTHVPPFSSSSVTPSTHTVITPTHAQMSTSASIHSTPTGTIPPLTTLTA--TGSTHTASPMTGTTIRTTQAHSSFSIAKTSTSILSHASSTHHPETTPTSTTNITPKSTSAGTSTPVAHTTLATSSRPPTPFTTHSPPTGS   169
SPISSTGPMTATSIKTTTTYPTPSHPQTTLTTHVPPFSTSSVTPSTHTVITPTHAQMSTSASIHSTPTGTVPPLTTRMP--TGSTHTGPPMTGTIIQTSKAHNSFSTAKTSTSLHSHASSTHHPETTPTSTTNITPKSTSAGTSTPVAHTTLATSSRLPTTFTTHFPPTGS   169
SHVSSTGPMTATSSQTTTTHPTPSHPQTTLPTHIPPFSTSLVTPSTHTVITPTHAQVSTSASIHSTPTGTIPPPTTVKATGTGSTHTAPRMTVTTSGTSQAHSSFSTAKTSTSLHSHASSTLHPEVTPTSTTTITPNPTNTGIRTPVANTTSATSSRLTTPFTTHSPPTGS   171
SPISSTGPMTATSFQTTTTYPTPSHPQTTLPTHVPPFSTSLVTPSTHTVITPTHAQMATSASIHSTPTGTIPPLTTLMN--TGSTHTAPPVTPTTSGTSQAASSFSTAKTSTSLHSHTSSTHHPEVTPTATTKITTNPTSIGSSTPMAHTTSATSSRLTTPFTTHSPSTGS   169
SPVSSTGPMTATSFQTTTTYPTPSLSQTTLPTYVPPFSTSLVTPSTHTVITPPRTQMATSASIHSTPTGTIPPPTTLKA--TGSTHTAPTRTLTTSGTSQALSSLNTAKTSTSLHSHTSSTHHPEATSTSTTNITPNPTSTGTGTPVAHTTSATSSRLTTPFTTHSPPTGS   169
TPISSTGPVTATSFHATTTYPTPSHPQTTHPTHVTSFSTSLVTPSTHTVITPTHAQMATSASIHSTPTGTISPPTTLKA--TGSIHTAPPMTPTTSGTSQSPSSFSMAKTSTSLPYHTSSTHHPEVTPTSTTNITPKHTSTGTRTPVAHTTSATSSKLPTPFTTHSPPTGS   169
SPISSTGPVTATSFQTTTTYPTPSHSHTTLPTHVPPSSTSLVTPNTHTVITHTHAQMSTSASIHSTPTGTIPPPTTLKA--TGSTHTAPPMTPTTSGTRQAPSSFSTAKTSTSLHSHTSSTHHPAVTPTSTTNITPNHTSTGTRTPVAHTTSATSSRLPTPFTTHSPPTGS   169
SPISSTAPVTATSFQTTTTYPTTSQSQTTLPTHIPPFSTSLVTPSTHTVITPTHQQMATSASIHSTPTGTIPPLTTLKA--TGSTHTAPPMTPTTSGTSQALSSFSTAKTSTSLHSHTSSTHHPEVTPTSTT-ITPKPTSTETGTPVADTTSATSSRLPTPFTTHSLPTGS   168
SPFSSTGPMTATSFQTTTTYPTPSHPHTTLPTHIPPFSTSLVTPSTHTVITTTHAQMSTSASIHSTPTGTIPPPTTLKA--TGSTHTAPPMTPTTSGTSQAPSSFSTAKTSTSLHSHTSSAHHPEVTPTSTT-ITPNPTSTETVTPVAHTTSATSSRLTTPFTTHSPPTGS   168
SPISSTGPVTATSFHATTTYPTPSHPQTTLPTHVPSFSTSLVTPSTHTVITPTHAQMTTSASIHSMPTGTIPPPTTLKA--TGSTHTAPPMMPTTSGTSQASSSFNTAKTSTSLHSHTSSTHHPEVTPTSITNITLNPTSIGTWTPVAHTTSATSSRLTTPFTTHSPPTGT   169
TPISSTGPVTATSFHATTTYPTPSHPQTTLPTHVPSFSTSLVTPSTHIVITPTHAQMATSASIHSMQTGTIPPPTTIKA--TGSTHTAPPMTPTTSGTSQSLSSFSTAKTSTSLPYHTSSTHHPEVTPTSTTNITPKHTSTGTRTPVAHTTSATSSRLPTPFTTHSPPTGS   169
SPISSTGPMTAPSFQTTTTYPTPSHPQTTLPTHIPPFSTSLVTPSTHKVITPTHAQMSTSASIHSTPTGTIPPLTTLKV--TGSTHTAPPITVTTSGTSPSASSFSTGKTSTSLHSHTSSTHYPEVTPTSTTTITPNHTSTGTRTPVAHTTSATSSRLPIPFTTHSPPTGS   169
SPISSTGPMTATSFQTTTTYPTPSHPQTTLPTHLPPFSTSLVTPSTHTVIITTHTQMATSASIHSTPTGTVPPPTTLKA--TGSTHTAPTMTPTTSGTSQALSSFNTAKTSTSLHSQTSSTHLPEVTPTSTA-ITPNPTSTGTGTPVAHTTSATSSRLTTPFTTHSSPTGS   168
SPFSSTGPMTATSFQTTTTYPTPSHPQTTLPTHVPPFSTSLVTPSTHTVITPTHAQMATSASIHSMPTGTIPPPTTLKA--TGSTHTAPTMTPTTSGTSQALSSLNTAKTSTSLHSHTSSTHHAEATSTSTTNITPNPTSTGT   141
                                                                                            PPMTVTTSGTSQSRSSFSTAKTSTSLHSHTSSTHHPEVTSTSTTSITPNHTSTGTRTPVAHTTSATSSRLPTPFTTHSPPTGT    83
TPISSTGPVTATSFQTTTTDPTPSHPHTTLPTHVPSFSTSLVTPSTHIVITPTHAQMATSASIHSMPTGTIPPPTTIKA--TGSTHTAPPMTATTSGTSQSPSSFSTAKTSTSLHSHISSTHHPEVTPTSTTTITPNHTSTGTRTPVAHTTSATSSRLPIPFTTHSPPTGS   169
SPISSTGPMTATSFQTTTTYPTPSHPQTTLPTHLPPFSTSLVTPSTHTVIITTHTQMATSASIHSTPTGTVPPPTTLKA--TGSTHTAPTMTPTTSGTSQALSSFNTAKTSTSLHSQTSSTHLPEVTPTSTT-ITPNPTSPGTGTPVAHTTSATSSRLTTPFTTHSSPTGS   168
SPFSSTGPMTATSFQTTTTYPTPSHPQTTLPTHVPPFSTSLVTPSTHTVITPTHAQMATSASIHSMPTGTIPPPTTLKA--TGSTHTAPTMTLTTSGTSQALSSLNTAKTSTSLHSHTSSTHHAEATSTSTTNITPNPTSTGT   141
                                                                                            PPMTVTTSGTSQSRSSFSTAKTSTSLHSHTSSTHHPEVTSTSTTSITPNHTSTGTRTPVAHTTSATSSRLPTPFTTHSPPTGT    83
TPISSTGPVTATSFQTTTTYPTPSHPHTTLPTHVPSFSTSLVTPSTHTVIIPTHTQMATSASIHSMPTGTIPPPTTIKA--TGSTHTAPPMTPTTSGTSQSPSSFSTAKTSTSLPYHTSSTHHPEVTPTSTTNITPKHTSTGTRTPVAHTTSASSSRLPTPFTTHSPPTGS   169
SPFSSTGPMTATSFQTTTTYPTPSHPQTTLPTHVPPFSTSLVTPSTHTVIITTHTQMATSASIHSTPTGTVPPPTTLKA--TGSTHTAPPMTVTTSGTSQTHSSFSTA                                                                 106
```

b

Reference consensus sequence from all tandem repeats described here:

SPISSTGPMTATSFQTTTTYPTPSHPQTTLPTHVPPFSTSLVTPSTHTVITPTHAQMATSASIHSTPTGTIPPPTTLKA--TGSTHTAPPMTPTTSGTSQAHSSFSTAKTSTSLHSHTSSTHHPEVTPTSTTNITPNPTSTGTRTPVAHTTSATSSRLPTPFTTHSPPTGS
/L

Reference tandem repeat described previously (Toribara et al. J Biol Chem 1993, 268: 5879-5885):

SPFSSTGPMTATSFQTTTTYPTPSHPQTTLPTHVPPFSTSLVTPSTGTVITPTHAQMATSASIHSTPTGTIPPPTTLKA--TGSTHTAPPMTPTTSGTSQAHSSFSTAKTSTSLHSHTSSTHHPEVTPTSTTTITPNPTSTGTSTPVAHTTSATSSRLPTPFTTHSPPTGS

c

Repeat	Homology % against ref.
1	90.5
2	83.4
3	81.7
4	84.6
5	84.6
6	88.9
7	91.7
8	90.5
9	89.9
10	93.5
11	91.7
12	93.5
13	91.1
14	91.7
15	90.5
16	91.7
17 (141)	94.3
18 (83)	91.6
19	91.1
20	91.7
21 (141)	93.6
22 (83)	91.6
23	91.1
24 (106)	94.3

Comments to the sequence of the MUC6 repeat units. **a** All repeats aligned with consensus amino acid at each position are marked by grey shading. None of the full length repeats are the the samebut the 83aa repeats are identical. **b** Consensus sequence as obtained from the present sequencing and compared to the one described by Toribara *et al.* Identical amino acids between the two sequences are bold and different amino acids underlined. **c** Analysis of sequence homology for each repeat compared to the consensus sequence as described here. More than 90% sequence homology were observed between most repeats and the minimal homology to reference consensus repeat is 81.7%.

Figure S11

Comparison of MUC2 PTS-TR2.

Sequences from RP13-807H17 and CH17 -246P12 TR units were compared and coordinated by means of unique (U) shared repeat units (unique referred to as TR´units present one time in both BAC sequences). Shared TR with identical aa sequence are marked in bold and identical DNA sequence highlighted in grey. Fifteen unique non-shared repeat units are marked in green (RP13-870H17) and 16 red (CH17-246P12). Sixty-four of the TR units are identical when comparing both sequences, 18 of these are homologous , but unique repeats (only present one time). Note that some of the repeat units share the aa sequence, but not the DNA sequence (marked in bold).

TR No	98TR from RP13-870H17		TR No	105TR from CH17-246P12	
**1**	**PTPTPITTTTTVTPTPTPTSTQS**	U	**1**	**PTPTPITTTTTVTPTPTPTSTQS**	U
**2**	**TTPTPITTTNTVTPTPTPTGTQT**	U	**2**	**TTPTPITTTNTVTPTPTPTGTQT**	U
**3**	**PTPTPITTTTTMVTPTPTITSTQT**	U	**3**	**PTPTPITTTTTMVTPTPTITSTQT**	U
**4**	**PTPTPITTTT*VTPTPTPTSTQR**	U	**4**	**PTPTPITTTT*VTPTPTPTSTQR**	U
**5**	**TTPTSITTTTTVTPTPTPTGTQT**		**5**	**TTPTSITTTTTVTPTPTPTGTQT**	U
**6**	**PTTTPITTTTTVTPTPTPTGTQT**		**6**	**PTTTPITTTTTVTPTPTPTGTQT**	
7	PTTTPISTTTTVTPTPTPTGTQT		7	PTTTPISTTTMVTPTPTPTGTQT	U
**8**	**LTPTPITTTTTVTPTPTPTGTQT**	U	**8**	**LTPTPITTTTTVTPTPTPTGTQT**	U
9	PTSTPITTTTTVTPTPTPTGTQT		9	PTSTPISTTTTVTPTPTPTGTQT	
**10**	**PTLTPITTTTTVTPTPTPTGTQT**	U	**10**	**PTLTPITTTTTVTPTPTPTGTQT**	
**11**	**PTTTPITTTTTVTPTPTPTGTKS**	U	**11**	**PTTTPITTTTTVTPTPTPTGTKS**	U
**12**	**TTPTSITTTTMVTPTPPPTGTQT**	U	**12**	**TTPTSITTTTMVTPTPPPTGTQT**	U
**13**	**PTTTPITTTTTVTPTPTPTGTQT**		**13**	**PTTTPITTTTTVTPTPTPTGTQT**	
**14**	**PTPTPITTTTTVTPTPTPTGTQT**		**14**	**PTPTPITTTTTVTPTPTPTGTQT**	
**15**	**PTSTPITTNTTVTPTPTPTGTPS**	U	**15**	**PTSTPITTNTTVTPTPTPTGTPS**	U
16	TTLTPITTTTTVTPTPTPTGTQT		16	TTLTPITTTTMVTPTPTPTGTQT	U
17	PTSTPISTTTMVTPTPTPTGTQT	U	17	PTSTPISTTTTVTPTPTPTGTQT	
18	PTPTPISTTTTVTPTPTPTGTQT		18	PTPTPISTTTTVTPTPTPTSTQT	U
			19	PTTTPITTTTTVTPNPTPTGTQT	U
			20	PTTTPITTTTTVTPTPTPTGTQT	
			21	PTTTPISTTTTVTPTPTPTGTQT	
			22	PTTTAITTTTTVTPTPTPTGTQT	U
			23	PTSTPITTTTTVTPTPTPTGTQT	
			24	PTSTPISNTTTVTPTPTPTGTQT	U
			25	PTVTPITTTTTVTPTRTPTGTKS	U
			26	TTPTSITTTTMVTPTPTPTGTHT	U
			27	PTTTPITTTTTVTPTPTPTGTQT	
**19**	**PTPTPITTTTTVTPTPTPTGTQT**		**28**	**PTPTPITTTTTVTPTPTPTGTQT**	
**20**	**PTSTPITTTTTVTPTPTPTGTQT**		**29**	**PTSTPITTTTTVTPTPTPTGTQT**	
**21**	**PTTTPITTNTTVTPTPTPTGTQT**	U	**30**	**PTTTPITTNTTVTPTPTPTGTQT**	U
**22**	**PTTVLITTTTTMTPTPTPTSTKS**		**31**	**PTTVLITTTTTMTPTPTPTSTKS**	
**23**	**TTVTPITTTTTVTPTPTPTGTQS**	U	**32**	**TTVTPITTTTTVTPTPTPTGTQS**	U
24	TTLTPITTTTTVTPTPTPTGIQT	U	33	TTLTPITTTTTVTPTPTPTGTQT	
25	PTTTPISTTTTVTPTPTPTGTQT		34	PTTTPISTTTTVIPTPTPTGTQT	U
**26**	**PTSTPITTTTTVTPTPTPTGTQT**		**35**	**PTSTPITTTTTVTPTPTPTGTQT**	
**27**	**PTSTPISTTTTVTPTATPTGTQT**	U	**36**	**PTSTPISTTTTVTPTATPTGTQT**	U
28	PTLTPITTTTTVTPTPTPTGTKS	U	37	PTLTPITTTTTVTSTPTPTGTQT	U
29	TTPTSITTTTTVTPTPTPTGTQT				
30	PTTTPITTTTTVTPTPTPTGTQT				
**31**	**PTPTPITTTTTVTPTPTPTSTQT**	U	**38**	**PTPTPITTTTTVTPTPTPTSTQT**	U
**32**	**PTSTPITTTTTVTPTPTPTGTQT**		**39**	**PTSTPITTTTTVTPTPTPTGTQT**	
33	PTTTPITTTTTVTPTPTPTGTQA		40	PTTTHITTTTTVTPTPTPTGTQA	U
34	PTPTAITTTTTGTPTPTPTGTQT	U	41	PTPTAITTTTTVTPTPTPTGTQT	
**35**	**PTTTPITTTTTVTPTPTPTGTQS**		**42**	**PTTTPITTTTTVTPTPTPTGTQS**	
**36**	**PTPTAITTTTTVTPTPTPTGTQT**		**43**	**PTPTAITTTTTVTPTPTPTGTQT**	
**37**	**PTTTPITTTTTVTPTPTPTGTQS**		**44**	**PTTTPITTTTTVTPTPTPTGTQS**	
38	TTLTPITTTTTVTPTPTPTGTQT		45	TTLTPITTTTTVTPIPTPTGTQT	U
**39**	**PTSTPITTTITVTPTPTPTGTQT**	U	**46**	**PTSTPITTTITVTPTPTPTGTQT**	U
**40**	**PTPTPISTTTTVTPTPTPTGTQT**		**47**	**PTPTPISTTTTVTPTPTPTGTQT**	
41	PTSTPITTTTTVTPTPTPTGTQT		48	PTTTPITTTTTVTPTPTPTGTQT	
**42**	**PTTTPISTTTTVTPTPTPTGTQT**		**49**	**PTTTPISTTTTVTPTPTPTGTQT**	
43	PTSTPITTTTTVTPTPTPTGTQT				
44	PTTTPISTTTTVTPTPTPTGTQT				
**45**	**PTSTPITTTTTVTPTPTPTGTQT**		**50**	**PTSTPITTTTTVTPTPTPTGTQT**	
**46**	**PTPTPITTTTTVTPTPTPTGTQT**		**51**	**PTPTPITTTTTVTPTPTPTGTQT**	
**47**	**PTSTPITTTTTVTPTPTPTGTQT**		**52**	**PTSTPITTTTTVTPTPTPTGTQT**	
48	PTPTPITTTTTVTPTPTPTGTQT				
**49**	**PTPTPITTTTTVTPTPTPTGTQT**		**53**	**PTPTPITTTTTVTPTPTPTGTQT**	
**50**	**PTSTPITTTTTVTPTPTPTGTQT**		**54**	**PTSTPITTTTTVTPTPTPTGTQT**	
**51**	**PTTTPITTTTTVTPTPTPTGTQS**		**55**	**PTTTPITTTTTVTPTPTPTGTQS**	
**52**	**TTLTPITTTTTVTPTPTPTGTQT**		**56**	**TTLTPITTTTTVTPTPTPTGTQT**	
53	PTSTPITTITTVTPTPTPTGTQT	U	57	PTSTPITTTTTVTPTPT--GTQT	U
**54**	**PTPTPISTTTTVTPTPTPTGTQT**		**58**	**PTPTPISTTTTVTPTPTPTGTQT**	
**55**	**PTMTPITTTTTVTPTPTPTGTQT**		**59**	**PTMTPITTTTTVTPTPTPTGTQT**	
**56**	**PTTTPISTTTTVTPTPTPTGTQT**		**60**	**PTTTPISTTTTVTPTPTPTGTQT**	
**57**	**PTSTPITTTTTVTPTPTPTGTQT**		**61**	**PTSTPITTTTTVTPTPTPTGTQT**	
**58**	**PTTTPITTTTTVTPTPTPTGTQS**		**62**	**PTTTPITTTTTVTPTPTPTGTQS**	
**59**	**TTLTPITTTTTVTPTPTPTGTQT**		**63**	**TTLTPITTTTTVTPTPTPTGTQT**	
**60**	**PTPTPISTTTTVTPTPTPTGTQT**		**64**	**PTPTPISTTTTVTPTPTPTGTQT**	
61	PTMTPITTTTTVTPTPTPTGTQT		65	PTTTPITTTTTVTPTPTPTGTQT	
**62**	**PTTTPISTTTTVTPTPTPTGTQT**		**66**	**PTTTPISTTTTVTPTPTPTGTQT**	
63	PRSTPITTTTKVTPTPTPTGTQT	U	67	PTSTPITTTTTVTPTPTPTGTQT	
64	PTPTPITTTTTVTPTPTPTGTQA	U	68	PTTTPITTTTTVTPTPTPTGTQA	
65	PTPAAITTTSTVTPTPTPTGTQT	U	69	PTPTAITTTTTVTPTPTPTGTQT	
			70	PTTTPITTTTMVTPTPTPTGTQT	U
			71	PTSTPITTTTTVTPTPTPTGTQT	
			72	PTPTPISTTTTVTPTPTPTGTQT	
			73	PTTTPITTTTTVTPTPTPTGTQT	
			74	PTTTPISTTTTVTPTPTPTGTQT	
			75	PTSTPITTTTTVTPTPTPTGTQT	
			76	PTPTPITTTTTVTPTPTPTGTQT	
			77	PTSTPITTTTTVTPTPTPTGTQT	
**66**	**PTTTPITTTTTVTPTPTPTGTQS**		**78**	**PTTTPITTTTTVTPTPTPTGTQS**	
**67**	**TTLTPITTTTTVTPTPTPTGTQT**		**79**	**TTLTPITTTTTVTPTPTPTGTQT**	
**68**	**PTSTPITTTTTVTPTPTPTGTQT**		**80**	**PTSTPITTTTTVTPTPTPTGTQT**	
**69**	**PTPTPISTTSTVTPTPTPTGTQT**	U	**81**	**PTPTPISTTSTVTPTPTPTGTQT**	U
**70**	**PTMTPITTTTTVTPTPTPTGTQT**		**82**	**PTMTPITTTTTVTPTPTPTGTQT**	
71	PTTTPISTTTTVTPTPTPTGTQN	U			
**72**	**PTSTPITTTTTVTPTPTPTGTQT**		**83**	**PTSTPITTTTTVTPTPTPTGTQT**	
**73**	**PTMTPITTTTTVTPTPTPTGTQA**	U	**84**	**PTMTPITTTTTVTPTPTPTGTQA**	U
**74**	**PTPTAITTTTTVTPTPTPTGTQT**		**85**	**PTPTAITTTTTVTPTPTPTGTQT**	
75	PTTTPITTTTTVTPTPIPTGTQS		86	PTTTPITTTTTVTPTPTPTGTQS	
**76**	**TTLTPITTTTTVTPTPTPTGTQT**		**87**	**TTLTPITTTTTVTPTPTPTGTQT**	
77	PTPIPISTTTTVTPTPTPTGTQT	U	88	PTPTPISTTTTVTPTPTPTGTQT	
**78**	**PTMTPITTTTTVTPTPTPTGTQT**		**89**	**PTMTPITTTTTVTPTPTPTGTQT**	
**79**	**PTTTPISTTTTVTPTPTPTGTQT**		**90**	**PTTTPISTTTTVTPTPTPTGTQT**	
80	PTSTPITTTTTVTPTPIPTGTQT	U	91	PTTTPITTTTTVTPTPTPTGTQT	
81	PTTTPITTTTTVTPTPTPTGTQA				
82	PTPTAITTTTTVTPTPTPTGTQT				
83	PTTTPITTTTTVTPTPIPTGTQS				
84	TTLTPITTTTTVTPTPTPTSTQT	U			
85	PTPTPISTTTTVTPTPTPTGTQT		92	PTTTPISTTTTVTPTPTPTGTQT	
86	PTMTPITTTTTVTPTPTPTGTQT		93	PTTTPITTTTTVTPTPTPTGTQT	
**87**	**PTTTPISTTTTVTPTPTPTGTQT**		**94**	**PTTTPISTTTTVTPTPTPTGTQT**	
88	PTSTPITTTTTVTPTPTSTGTQT	U	95	PTSTPITTTTTVTPTPTPTGTQT	
**89**	**PTTTPITTTTTVTPTPTPTGTQA**		**96**	**PTTTPITTTTTVTPTPTPTGTQA**	
**90**	**PTPTAITTTSTVTPTPTPTGTQT**	U	**97**	**PTPTAITTTSTVTPTPTPTGTQT**	U
**91**	**PTTTPITTTTTVTPTPTPTGTQS**		**98**	**PTTTPITTTTTVTPTPTPTGTQS**	
**92**	**PTPTAITTTTTVTPTPTPTGTQT**		**99**	**PTPTAITTTTTVTPTPTPTGTQT**	
93	PTSTPITTTTTVTPTPTPTGTQT		100	PTLTPITTTTTVTPTPTPTGTQT	
**94**	**PTPTPISTTTTVTPTPTPTGTQT**		**101**	**PTPTPISTTTTVTPTPTPTGTQT**	
**95**	**PTTTPITTTTTVTPTPTPTGTQT**		**102**	**PTTTPITTTTTVTPTPTPTGTQT**	
**96**	**PTTVLITTTTTMTPTPTPTSTKS**		**103**	**PTTVLITTTTTMTPTPTPTSTKS**	
**97**	**TTVTPITTTTTVTATPTPTGTQT**	U	**104**	**TTVTPITTTTTVTATPTPTGTQT**	U
**98**	**PTMIPISTTTTVTPTPTPTTGST**	U	**105**	**PTMIPISTTTTVTPTPTPTTGST**	U

Unique repeats only found in 98TR
Unique repeats only found in 105TR
U= unique shared TR units with identical amino acid sequence
* = One aa less
Shared TRs with identical DNA sequence are (marked in GREY)

Figure X1

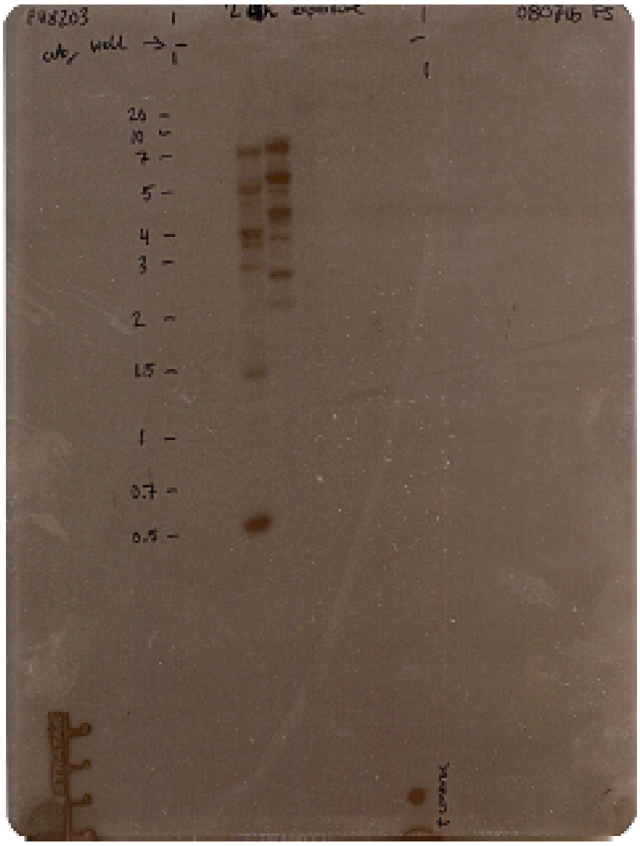

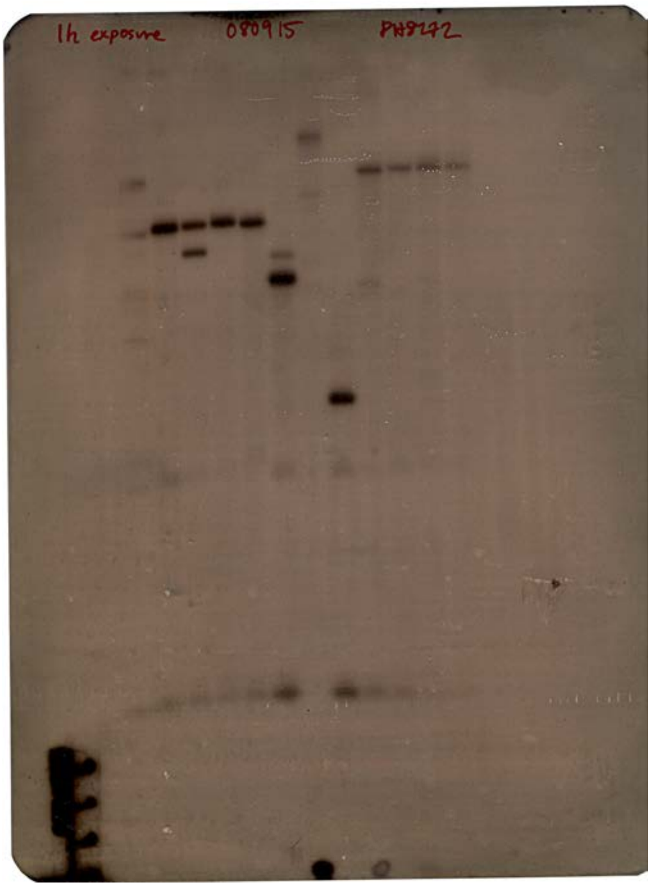

Supplement: Supplementary file 1 — Supplementary Tables and Figures [file 41598_2018_35499_MOESM1_ESM.pdf]
